# Palladium-catalyzed asymmetric hydrogenation of lactones under base-free conditions†

**DOI:** 10.1039/d4sc01890g

**Published:** 2024-06-12

**Authors:** Han Wang, Shan-Shan Xun, Chang-Bin Yu, Yong-Gui Zhou

**Affiliations:** a School of Chemistry, Dalian University of Technology Dalian 116033 P. R. China; b State Key Laboratory of Catalysis, Dalian Institute of Chemical Physics, Chinese Academy of Sciences Dalian 116023 P. R. China ygzhou@dicp.ac.cn

## Abstract

Asymmetric hydrogenation of esters through homogeneous catalysis is a significantly important transformation in organic synthesis. The systems developed so far mainly focused on chiral iridium and ruthenium catalysts, which required a base to facilitate the activity. Herein, we present a palladium-catalyzed asymmetric hydrogenation of lactones under base-free conditions through dynamic kinetic resolution and kinetic resolution. The reaction exhibits high enantioselectivity and excellent functional group tolerance. Remarkably, the hydrogenation proceeds smoothly at the gram scale, and the products can be transformed into several chiral potential building blocks without loss of optical purity. This work provides a new strategy for asymmetric hydrogenation of esters under base-free conditions.

## Abstract 2

We present a palladium-catalyzed asymmetric hydrogenation of lactones under base-free conditions through DKR and KR with high enantioselectivity. This work provides a new strategy for asymmetric hydrogenation of esters under base-free conditions.

## Graphical abstract

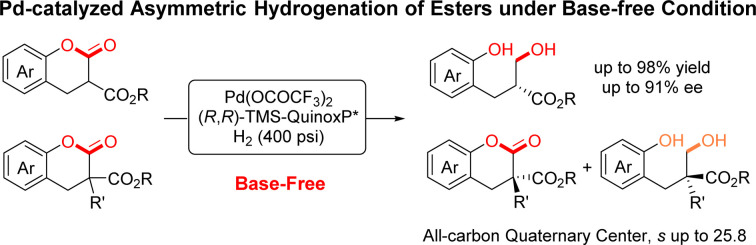

## Introduction

**Scheme 1:**
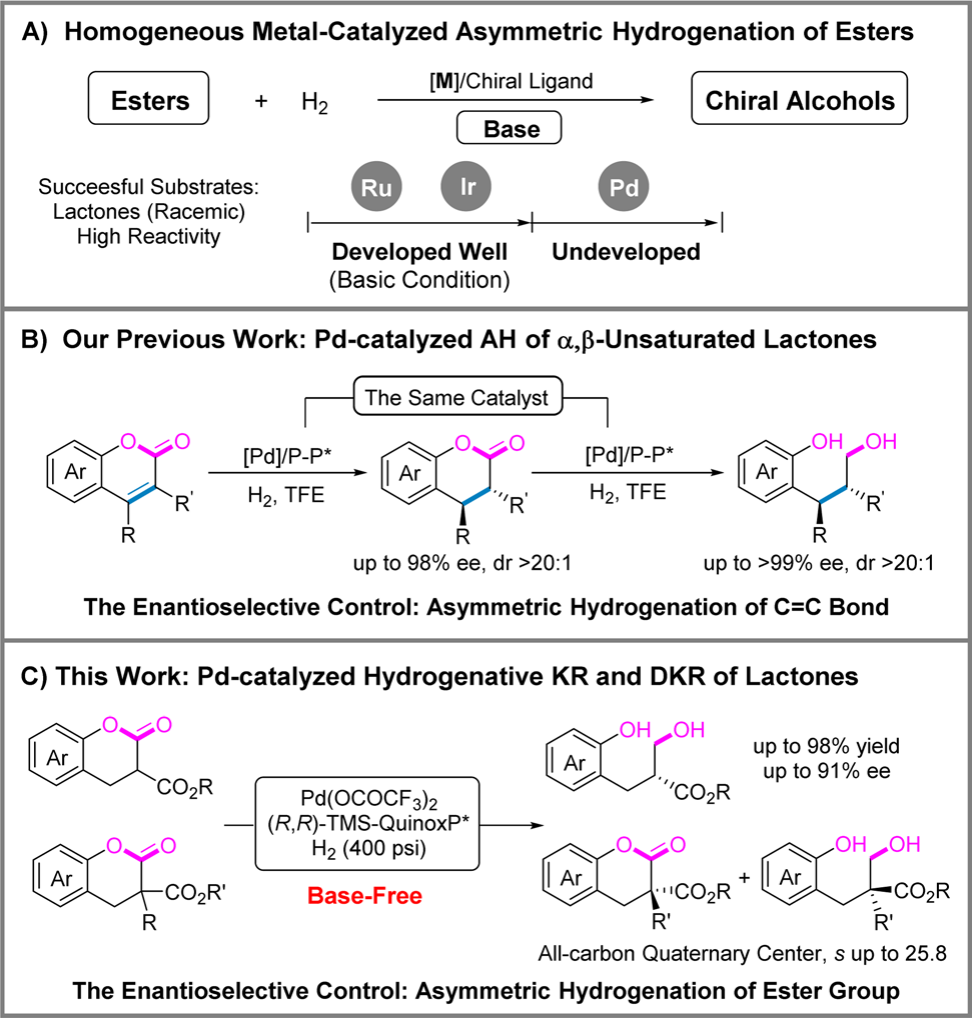
The asymmetric hydrogenation of esters.

The reduction of readily available esters to alcohols has found wide applications in the production of fine chemicals, such as pharmaceuticals, pesticides, and fragrances, representing an important type of reaction in industrial processes.^1–3^ Traditional ester reduction methods typically employ reducing reagents such as lithium aluminum hydride, metal borohydrides, and boranes. However, these methods are characterized by low economic efficiency, safety concerns, and low atom economy. Homogeneous catalytic reduction of esters is known for its operational simplicity, high atom economy and positive environmental friendliness. Although significant advances have been made in homogeneous catalytic reductive systems for esters,^4–7^ the development of asymmetric reduction of esters is relatively disproportionate. The asymmetric reduction of esters, including hydrosilylation,^8–11^ hydroboration,^12^ CBS reduction^13–17^ and hydrogenation,^18–25^ presents specific challenges. This is because the stereocenter in the reduction products typically emerges at the α-position rather than the reaction site, thereby complicating the enantioselective control. Among them, it is widely acknowledged that transition-metal-catalyzed asymmetric hydrogenation stands out as one of the most powerful and versatile tools to achieve chiral alcohols. The currently reported asymmetric hydrogenation of esters is mostly achieved through precious ruthenium or iridium catalysts (Scheme 1A). And the substrates are mostly limited to lactones or hydroxyesters (the *in situ* intramolecular formation of lactones).^6^

In 2011, Ikariya's group disclosed a well-defined Cp*Ru catalyst with chiral modification in the ligand sphere, providing a practical pathway for asymmetric hydrogenation of racemic lactones *via* dynamic kinetic resolution.^18^ Then, Zhou's group introduced an efficient method for kinetic resolution (KR) of racemic hydroxy esters through catalytic hydrogenation of hydroxyl esters, employing an Ir-SpiroPAP catalyst, and achieved a high stereoselectivity factor with an exceptionally low catalyst loading (0.001 mol%).^19^ Based on this achievement, Zhou's group developed a protocol for the Ir-SpiroPAP-catalyzed hydrogenative dynamic kinetic resolution (DKR) of racemic α-substituted lactones,^20^ demonstrating high reactivities and enantioselectivities. In 2018, Zhang's group disclosed an iridium-catalyzed DKR of Bringmann's lactones *via* asymmetric hydrogenation, providing an atom-economic and straightforward approach to valuable axially chiral biaryls.^21^ In 2019, another Ir-SpiroPAP catalyst featuring site-specific modification was developed by Zhou's group to enable asymmetric hydrogenation of both α-arylamino γ-lactones and α-arylamino δ-lactones.^22^ In the same year, Bergens and coworkers unveiled a novel ruthenium catalyst, pioneering the development of the first highly active and enantioselective hydrogenation of acyclic esters with impressive turnover numbers.^23^ In 2022, Zhou's group realized an iridium-catalyzed highly enantioselective hydrogenation of racemic α-aryloxy lactones *via* dynamic kinetic resolution. This achievement was made possible by the rational design of the first chiral ligands containing a C5-substituted chiral oxazoline unit.^24^ In 2023, Ohkuma's group accomplished asymmetric hydrogenation of racemic α-substituted α-amino esters resulting in the production of chiral β-amino alcohols through dynamic kinetic resolution with chiral ruthenabicyclic complexes.^25^ The following mechanistic studies revealed that the reaction proceeded *via* 1,2-hydride migration of the α-amino acetate intermediate into the α-hydroxy imine. Very recently, Xie and Zhou disclosed a novel hydroxy-assisted strategy enabling asymmetric hydrogenation of racemic esters with remote stereocenters *via* kinetic resolution, facilitated by chiral Ir-SpiroPAP catalysts.^26^ This approach demonstrates remarkable efficacy in synthesizing chiral primary alcohols and recovering valuable chiral pharmaceuticals. To the best of our knowledge, all these reported methods could be conducted in the presence of bases. Notably, asymmetric hydrogenation of esters under base-free conditions is rare.

Building on our extensive experience with homogeneous palladium-catalyzed asymmetric hydrogenation,^27–32^ very recently, our group developed a highly enantioselective palladium-catalyzed hydrogenation of α,β-unsaturated lactones *via* auto-tandem catalysis (Scheme 1B), affording chiral alcohols.^33^ It is noteworthy that the enantioselectivity of the product mainly originates from asymmetric hydrogenation of the C=C bond rather than the hydrogenation of the ester group. This is a rare example of hydrogenation of an ester under base-free conditions. Inspired by the above results, we wondered whether palladium-catalyzed asymmetric hydrogenation of the ester group was possible. In this report, we present our latest research on the hydrogenative dynamic kinetic resolution and kinetic resolution of racemic lactones using a chiral palladium catalyst under base-free conditions (Scheme 1C).

## Results and discussion

We selected racemic dihydrocoumarin 1a, a widely used lactone in pharmaceuticals and fragrances, to explore the optimal reaction parameters (Table 1). Initially, different solvents were evaluated. 2,2,2-Trifluoroethanol (TFE) gave 85% ee albeit 13% yield (entry 1). Meanwhile, DCM and toluene exhibited low reactivities (entries 2 & 4). As a result, hexafluoro-2-propanol (HFIP) delivered an 87% yield and 77% ee (entry 3). Besides, to further promote the enantioselectivity, mixed solvents were evaluated (entries 5–8). Among them, HFIP : toluene = 5 : 1 demonstrated the best activity and enantioselectivity (entry 8). After decreasing the reaction temperature to 30 °C, a slight increase of ee was obtained (entry 9). Subsequently, a series of commercially available chiral electron-donating bisphosphine ligands were examined (entries 9–14). The backbone-modified (*R*,*R*)-TMS-QuinoxP* disclosed by Ito's group^34^ exhibited superior activity and enantioselectivity compared to some other ligands, giving a 98% isolated yield and 91% ee (entry 13).

**Table 1:** Optimization of the DKR reaction conditions^a^

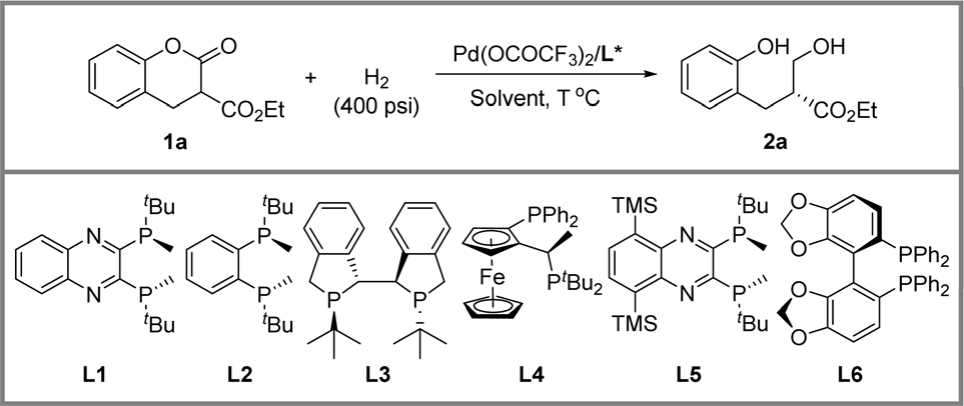					
Entry	Solvent	Ligand	*T* (°C)	2a^b^ (%)	ee^c^ (%)
1	TFE	L1	40	13	85
2	DCM	L1	40	<5	—
3	HFIP	L1	40	87	77
4	Toluene	L1	40	<5	—
5	HFIP/TFE (5/1)	L1	40	70	82
6	HFIP/DCM (5/1)	L1	40	>95	85
7	HFIP/THF (5/1)	L1	40	>95	84
8	HFIP/toluene (5/1)	L1	40	90	87
9	HFIP/toluene (5/1)	L1	30	67	88
10	HFIP/toluene (5/1)	L2	30	>95	78
11	HFIP/toluene (5/1)	L3	30	83	84
12	HFIP/toluene (5/1)	L4	30	>95	58
**13**	**HFIP/toluene (5/1)**	L5	**30**	**98** d	**91**
14	HFIP/toluene (5/1)	L6	30	<5	—

a Lactone 1a (0.2 mmol), 5 mol% palladium catalyst, H_2_ (400 psi), solvent (1 mL), 48 h.

b Determined by NMR using CH_2_Br_2_ as an internal standard.

c The enantiomeric excess of 2a was determined by HPLC.

d Isolated yield.

**Scheme 2:**
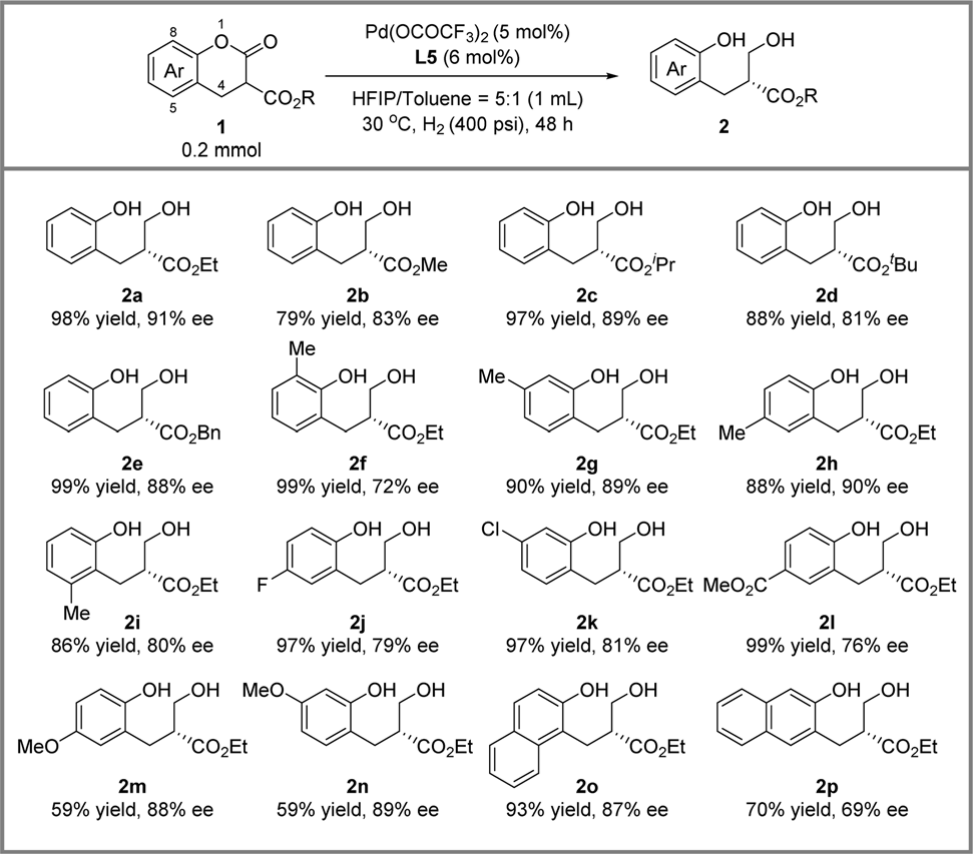
Substrate scope of Pd-catalyzed DKR of lactones.

With the optimized conditions in hand, we conducted the hydrogenative DKR of lactones and the results are presented in Scheme 2. Firstly, we investigated the effect of different R groups. The ethyl (2a), isopropyl (2c) and benzyl (2e) groups achieved good yield and approximately 90% ee. When the R group was smaller, such as methyl (2b), or larger, such as *tert*-butyl (2d), the reaction could only achieve over 80% enantioselectivity. Subsequently, we examined the steric effect of the benzene ring of the lactones. Substrates 1f and 1i bearing a substituent on C8 and C5 smoothly provided the reductive products in moderate ee. Products 2g and 2h with methyl substituents on C7 and C6 positions were obtained with 89% ee and 90% ee, respectively. Furthermore, the electronic effect of the substituents on the benzene ring on reactivity and enantioselectivity was significant. The electron-withdrawing group on the benzene ring had a positive effect on reactivities (2j–2l). Meanwhile, 2m and 2n with electron-donating groups were afforded in moderate yield and with 88 to 89% ee. Finally, we investigated substrates 1o and 1p containing a naphthalene ring, and 2o was obtained with 93% yield and 87% ee and 2p was obtained with 70% yield and 69% ee.

**Scheme 3:**
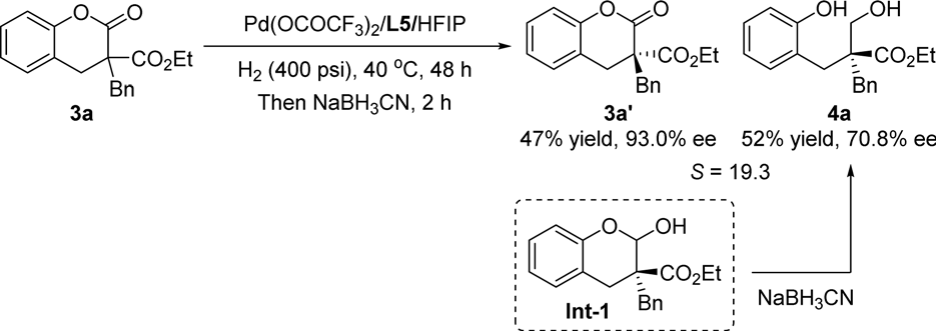
Optimal conditions for hydrogenative KR of lactone 3a.

Encouraged by the success of hydrogenative DKR of lactones, we further developed a palladium-catalyzed hydrogenative KR reaction of lactones with a quaternary stereogenic center. After parameter investigation (see ESI Table S1†), the optimal conditions were identified as shown in Scheme 3: 3a (0.3 mmol), palladium trifluoroacetate (5 mol%), ligand L5 (6 mol%), H_2_ (400 psi), HFIP (1 mL), 40 °C, 48 h, providing a stereoselectivity factor of 19.3. It should be noted that a small amount of hemiacetal intermediate Int-1 could be observed, leading to a deviation in the calculation of the stereoselectivity factor. According to our previous research, the conversion of 3a to Int-1 is the enantio-determining step.^14^ Therefore, the *in situ* addition of a reductant to facilitate the transformation of the Int-1 into final product 4a has no impact on the reaction's stereoselectivity factor. After a series of screenings, the sodium cyanoborohydride was chosen as the reductant.

Next, we began an exploration of the scope of lactones under optimal conditions (Table 2). Firstly, reactions proceeded smoothly with good stereoselectivity factors, when a methyl group (3b) and a fluorine atom (3c) were attached to the aromatic group of R^1^. However, the introduction of a large steric effect group, such as the 2-naphthyl group (3d), resulted in lower reactivity and stereoselectivity factor. Remarkably, a broad range of alkyls were well tolerated. Interestingly, a positive correlation was observed between the length of the alkyl chain and the stereo-selectivity factor of the reaction (3e–3h). Furthermore, when the R^2^ group consisted of different-sized cyclic alkanes, such as cyclopropyl (3i), cyclobutyl (3j), and cyclohexyl (3k), the reaction achieved similar stereoselectivity factors. The substrates (3l, 3m) with a methyl ester or isopropyl ester were also investigated, and a slight erosion of the stereoselectivity factor was observed. Finally, substituents including methyl (3n), chlorine (3o), and methoxy (3p) in the benzene ring were investigated. Among them, a slight decrease in the stereoselectivity factor (*s* = 9.6) was observed for 3o with the electron-withdrawing chlorine atom.

**Table 2:** Substrate scope of Pd-catalyzed KR of lactones^a^

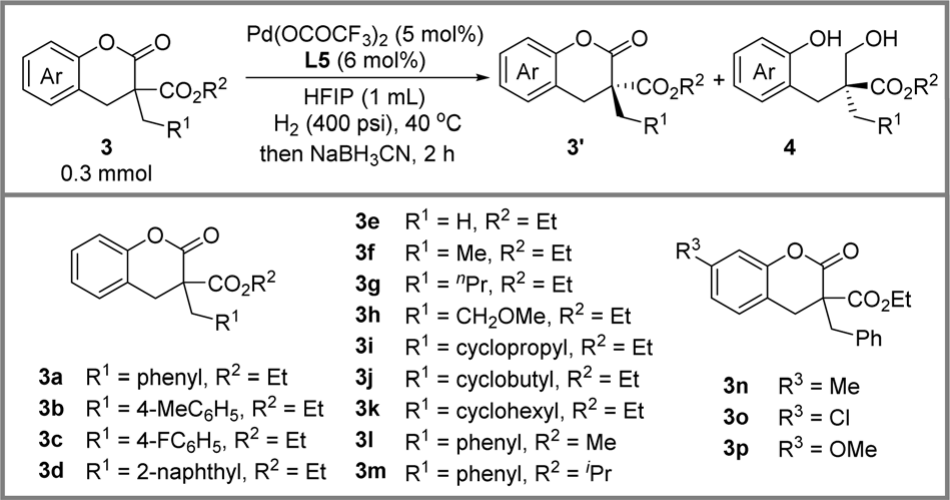							
Entry	3	Time (h)	Conv.^b^ (%)	4^c^ (%)	ee^d^ (%)		*S* e
					3′	4	
1	3a	48	53	52	93.0	70.8	19.3
2	3b	55	56	54	84.1	61.6	10.7
3	3c	72	62	61	98.3	60.1	17.7
4	3d	120	45	45	59.7	67.1	9.2
5	3e	72	65	64	98.4	53.8	14.6
6	3f	60	53	49	88.6	80.5	27.0
7	3g	72	57	57	99.0	75.0	35.5
8	3h	66	47	46	66.4	84.4	23.5
9	3i	72	68	68	98.8	39.8	10.1
10	3j	55	67	67	95.6	48.0	9.9
11	3k	72	64	64	97.0	55.0	13.4
12	3l	50	52	51	72.0	58.6	8.0
13	3m	72	54	53	80.8	71.2	14.6
14	3n	56	50	50	88.1	78.1	23.4
15	3o	72	51	50	82.1	59.3	9.6
16	3p	72	65	65	97.9	49.5	12.1

a Lactones 3 (0.3 mmol), Pd(OCOCF_3_)_2_ (5 mol%), (*R*,*R*)-TMS-QuinoxP* (6 mol%), H_2_ (400 psi), HFIP (1 mL), 40 °C.

b Determined by NMR, using CH_2_Br_2_ as an internal standard.

c Isolated yields.

d The enantiomeric excess of compound 3′ and 4 was determined by HPLC.

e Calculated selectivity factors: *C* = ee of 3′/(ee of 3′ + ee of 4), *s* = ln[(1 − *C*)(1 − ee of 3′)]/ln[(1 − *C*)(1 + ee of 3′)].

**Scheme 4:**
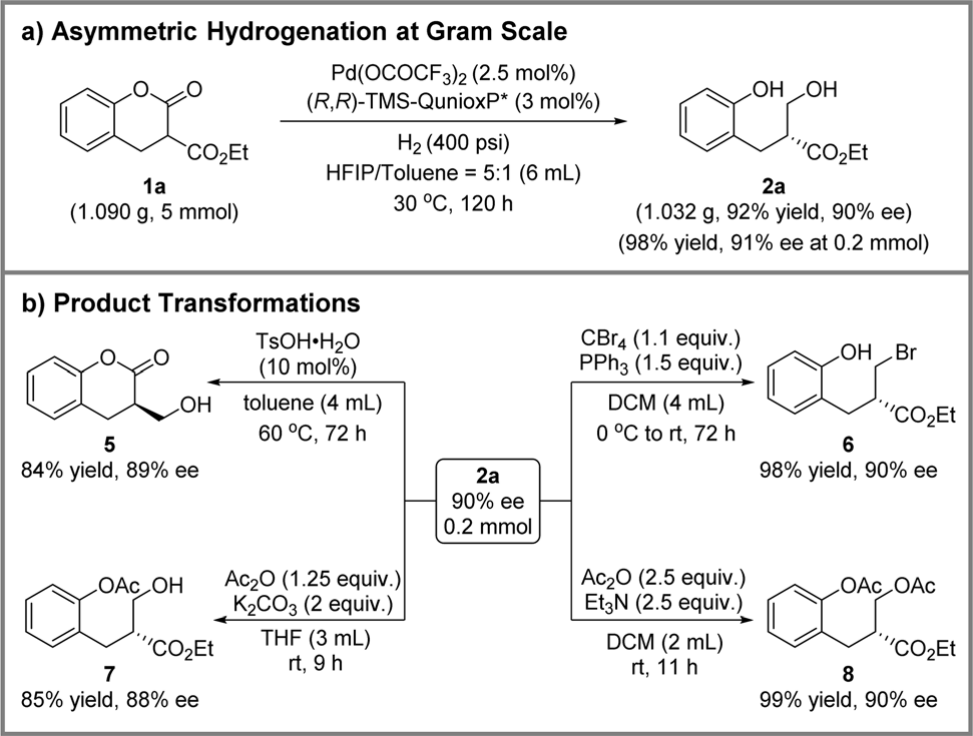
Scale-up and product transformations.

To demonstrated the utility of the above methodology, the palladium-catalyzed asymmetric hydrogenation of ester 1a was conducted at the gram scale (Scheme 4a). As a result, 1.032 g of product 2a could be obtained in 92% isolated yield and 90% ee maintaining both reactivity and enantioselectivity. Then, four product elaborations were performed, and the results are presented in Scheme 4b. In the presence of 4-toluenesulfonic acid monohydrate, transesterification proceeded smoothly at 60 °C, giving the chiral lactone 5 with 84% yield and 89% ee. Additionally, an Appel reaction was carried out to afford the brominated product 6 with 98% yield and 90% ee. Finally, single or double acyl protected products were achieved by employing the different bases. Using potassium carbonate as the base, product 7 with only phenolic hydroxyl group protection was obtained in 85% yield and 88% ee. Simultaneously, product 8 with the protection of both phenolic and alcoholic hydroxyl groups was obtained without any loss of optical purity using triethylamine as base.

## Conclusion

In conclusion, we have successfully developed a palladium-catalyzed asymmetric hydrogenation of lactones under base-free conditions through dynamic kinetic resolution and kinetic resolution. This methodology exhibits good enantioselectivity, reactivity and functional group tolerance. Notably, this reaction could proceed smoothly at the gram scale, and the products can be transformed into several chiral building blocks without loss of optical purity. Additionally, this is the first example of asymmetric hydrogenation of esters under base-free conditions. Ongoing investigations in our laboratory focus on utilizing palladium catalysts for the hydrogenation of acyclic esters and other carboxyl derivatives.

## Data availability

General information, detailed experimental procedures, characterization data for compounds, X-ray crystallographic data, and NMR and HPLC spectra are available in the ESI.† Crystallographic data is available *via* the CCDC.

## Author contributions

Y.-G. Zhou: conception, design, review and editing. C.-B. Yu: conception, review and editing. H. Wang: experiments, purification, analysis and writing. S.-S. Xun: data checking.

## Conflicts of interest

There are no conflicts to declare.

## Supplementary Material

SC-015-D4SC01890G-s001

SC-015-D4SC01890G-s002
