# Supplementary material for: Palladium-catalyzed asymmetric hydrogenation of lactones under base-free conditions
Source: Chem Sci. 2024 Jun 12;15(28):11038–42. doi: 10.1039/d4sc01890g (PMC11253110; doi:10.1039/d4sc01890g)
Supplement: SC-015-D4SC01890G-s001 [file SC-015-D4SC01890G-s001.pdf]

Electronic Supplementary Material (ESI) for Chemical Science.
This journal is © The Royal Society of Chemistry 2024

Supporting Information

Palladium-catalyzed Asymmetric Hydrogenation of Lactones under Base-Free Condition

Han Wang, Shan-Shan Xun, Chang-Bin Yu, and Yong-Gui Zhou**
School of Chemistry, Dalian University of Technology, 2 Linggong Road, Dalian 116024, P. R. China; State Key Laboratory of Catalysis, Dalian Institute of Chemical Physics, ChineseAcademy of Sciences, Dalian 116023, P. R. China
Email: cbyu@dicp.ac.cn; ygzhou@dicp.ac.cn

Table of Contents

1. **General and Materials...S1**
2. **Synthesis of Substrate Lactones...S1-9**
3. **Condition Optimization for Kinetic Resolution...S10**
4. **Pd-catalyzed Hydrogenative DKR and KR of Lactones...S11-22**
5. **Experiment at Gram Scale...S23**
6. **Product Elaboration...S24-26**
7. **Determination of Absolute Configuration...S27-30**
8. **Proposed Mechanism of DKR...S31**
9. **References...S32**
10. **Copy of NMR and HPLC Spectra...S33-211**

1. General and Materials

General: All reactions were carried out under an atmosphere of nitrogen using the standard Schlenk techniques, unless otherwise noted. ^{1}H NMR, ^{13}C NMR and ^{19}F NMR spectra were recorded at room temperature in $CDCl_3$ on 400 MHz instrument with TMS as internal standard. Enantiomeric excess was determined by HPLC analysis, using the chiral column described below in detail. Optical rotations were measured by polarimeter. Flash column chromatography was performed on silica gel (200-300 mesh). The heat source for all heating reactions is the oil bath. High-resolution mass spectrometry (HRMS) was measured on an electrospray ionization (ESI) apparatus using the time-of-flight (TOF) mass spectrometry. All reactions were monitored by TLC analysis.

Materials: Commercially available reagents and anhydrous solvents were used throughout without the further purification.

2. Synthesis of Substrate Lactones

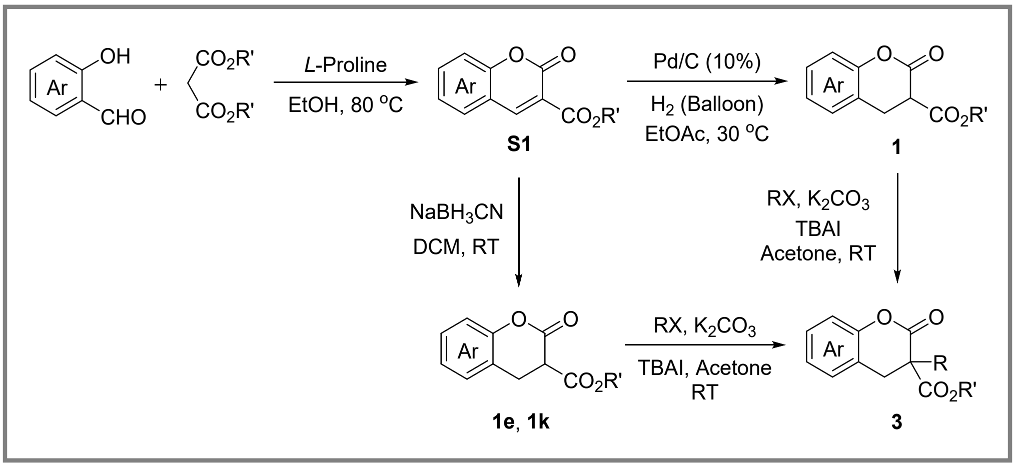

The substrate lactones **1** were prepared from the readily available 2-hydroxyarylaldehydes and malonates over two steps according to the known methods.[1,2] The first step is the synthesis of the intermediate coumarin-3-carboxylates **S1**, which are the known compounds; the second step is Pd/C-catalyzed hydrogenation to afford the substrate lactones. For **1e** and **1k** which is sensitive to Pd/C-catalyzed hydrogenation system, sodium cyanoborohydride was used for reduction. Among them, lactones **1a**, **1b**, **1h**, **1j**, **1m** and **1o** were the known compounds.[1,3]

The substrate lactones **3** with quaternary stereocenter were prepared through the alkylation of above lactones. Among them, lactones **3ae** and **3af** were the known compounds.[4]

2.1. The Synthesis of Alkoxycarbonyl Substituted Lactones

General Procedure for the Synthesis of Intermediate Coumarin-3-carboxylates S1:

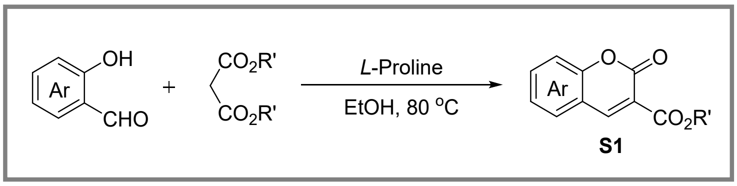

To a mixture of 2-hydroxyarylaldehyde (7.0 mmol), malonate (8.4 mmol) and ethanol (5 mL), *L*-proline (0.350 g, 3 mmol) was added and heated to 80 °C overnight. After the completion of the

reaction (monitored by TLC), the mixture was cooled to room temperature and poured in water (50 mL). After stirred for 10 min, the mixture was filtered and the residue was washed with cool ethanol to give a crude product **S1** for next step without the further purification.

General Procedure for the Synthesis of Lactones 1 with Pd/C:

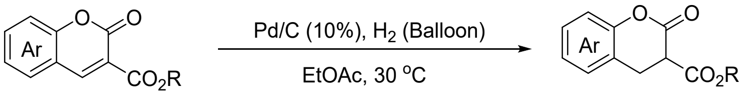

S1 **1**

To a solution of the above coumarin-3-carboxylates **S1** (7.0 mmol) in ethyl acetate (5 mL), 10% palladium on activated charcoal (20% wt of **S1**) was added, then the mixture was charged hydrogen gas (balloon). After the reaction completion (monitored by TLC), the solid was filtered off and the volatiles were removed under the reduced pressure. Then, the residue was purified by silica gel column using hexanes/ethyl acetate (5:1) as eluent to afford the desired alkoxycarbonyl lactones **1**.

***Iso*-Propyl 2-oxochromane-3-carboxylate (1c):**

Reaction conduct under 20 mmol scale. 2.098 g, 45% yield over two steps, white solid, mp 61-62 °C, known compound, R_f = 0.60 (hexanes/ethyl acetate 5/1). ^{1}H NMR (400 MHz, $CDCl_3$) δ 7.28-7.24 (m, 1H), 7.22-7.14 (m, 1H), 7.14-7.01 (m, 2H), 5.10-4.90 (m, 1H), 3.79-3.65 (m, 1H), 3.42-3.33 (m, 1H), 3.21-3.12 (m, 1H), 1.19 (d, *J* = 6.3 Hz, 3H), 1.11 (d, *J* = 6.3 Hz, 3H). ^{13}C NMR (100 MHz, $CDCl_3$) δ 167.1, 165.0, 151.5, 128.7, 128.3, 124.8, 120.8, 116.8, 70.0, 46.6, 27.4, 21.5, 21.4. The HRMS Calculated for $C_{13}H_{15}O_4$ $[M+H]^+$ 235.0965, found: 235.0969.

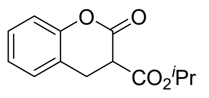

***tert*-Butyl 2-oxochromane-3-carboxylate (1d):**

Reaction conduct under 20 mmol scale. 1.809 g, 36% yield over two steps, white solid, mp 45-46 °C, new compound, R_f = 0.70 (hexanes/ethyl acetate 5/1). ^{1}H NMR (400 MHz, $CDCl_3$) δ 7.23 (m, 1H), 7.21 (d, *J* = 7.3 Hz, 1H), 7.13-7.04 (m, 2H), 3.68 (dd, *J* = 6.8, 6.0 Hz, 1H), 3.33 (dd, *J* = 15.9, 7.0 Hz, 1H), 3.17 (dd, *J* = 15.9, 5.8 Hz, 1H), 1.34 (s, 9H). ^{13}C NMR (100 MHz, $CDCl_3$) δ 166.6, 165.3, 151.6, 128.6, 128.3, 124.7, 121.0, 116.7, 83.2, 47.3, 27.68, 27.65. HRMS Calculated for $C_{14}H_{17}O_4$ $[M+H]^+$ 249.1121, found: 249.1120.

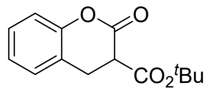

Ethyl 8-methyl-2-oxochromane-3-carboxylate (1f):

0.745 g, 45% yield over two steps, white solid, mp 65-66 °C, new compound, R_f = 0.70 (hexanes/ ethyl acetate 5/1). ^{1}H NMR (400 MHz, $CDCl_3$) δ 7.17-7.07 (m, 1H), 7.07-6.65 (m, 2H), 4.31-4.09 (m, 2H), 3.79-3.63 (m, 1H), 3.44-3.31 (m, 1H), 3.21-3.05 (m, 1H), 2.29 (s, 3H), 1.19 (t, *J* = 7.1 Hz, 3H). ^{13}C NMR (100 MHz, $CDCl_3$) δ 167.7, 165.1, 149.7, 130.2, 126.2, 125.7, 124.3, 120.6, 62.1, 46.4, 27.5, 15.6, 14.0. The HRMS Calculated for $C_{13}H_{15}O_4$ $[M+H]^+$ 235.0965, found: 235.0963.

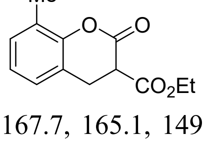

Ethyl 7-methyl-2-oxochromane-3-carboxylate (1g):

0.401 g, 24% yield over two steps, white solid, mp 58-59 °C, new compound, R_f = 0.50 (hexanes/

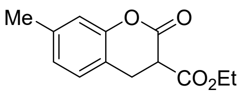

ethyl acetate 5/1). ^1H NMR (400 MHz, $CDCl_3$) δ 7.08-6.93 (m, 2H), 6.93-6.82 (m, 1H), 4.25-4.06 (m, 2H), 3.73-3.59 (m, 1H), 3.35-3.23 (m, 1H), 3.13-3.00 (m, 1H), 2.26 (s, 3H), 1.31-1.08 (m, 3H). ^{13}C NMR (100 MHz, $CDCl_3$) δ 167.6, 165.0, 149.3, 134.4, 129.1, 128.7, 120.4, 116.4, 62.1, 46.4, 27.2, 20.7, 13.9. The HRMS Calculated for $C_{13}H_{15}O_4$ $[M+H]^+$ 235.0965, found: 235.0967.

Ethyl 5-methyl-2-oxochromane-3-carboxylate (1i):

0.820 g, 50% yield over two steps, white solid, mp 61-62 °C, new compound, R_f = 0.55 (hexanes/

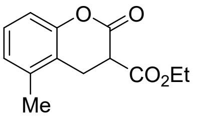

ethyl acetate 5/1). ^1H NMR (400 MHz, $CDCl_3$) δ 7.19-7.07 (m, 1H), 7.03-6.85 (m, 2H), 4.27-4.11 (m, 2H), 3.79-3.66 (m, 1H), 3.39-3.29 (m, 1H), 3.15-3.04 (m, 1H), 2.31 (s, 3H), 1.21 (t, J = 7.1 Hz, 3H). ^{13}C NMR (100 MHz, $CDCl_3$) δ 167.7, 164.9, 151.6, 136.6, 128.1, 126.3, 119.5, 114.6, 62.2, 46.1, 24.3, 19.1, 14.0. The HRMS Calculated for $C_{13}H_{15}O_4$ $[M+H]^+$ 235.0965, found: 235.0975.

3-Ethyl 6-methyl 2-oxochromane-3,6-dicarboxylate (1l):

0.905 g, 46% yield over two steps, white solid, new compound, mp 72-73 °C, R_f = 0.50 (hexanes/

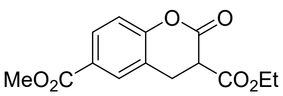

ethyl acetate 5/1). ^1H NMR (400 MHz, $CDCl_3$) δ 8.06 -7.81 (m, 2H), 7.19-7.04 (m, 1H), 4.29-4.12 (m, 2H), 3.92 (s, 3H), 3.86-3.75 (m, 1H), 3.46 (dd, J = 16.1, 8.0 Hz, 1H), 3.25 (dd, J = 16.2, 6.1 Hz, 1H), 1.21 (t, J = 7.1 Hz, 3H). ^{13}C NMR (100 MHz, $CDCl_3$) δ 167.1, 166.0, 164.0, 154.6, 130.5, 130.1, 126.8, 120.8, 116.9, 62.4, 52.3, 46.0, 27.2, 14.0. The HRMS Calculated for $C_{14}H_{15}O_6$ $[M+H]^+$ 279.0863, found: 279.0866.

Ethyl 7-methoxy-2-oxochromane-3-carboxylate (1n):

0.642 g, 37% yield over two steps, white solid, new compound, mp 75-76 °C, R_f = 0.70 (hexanes/

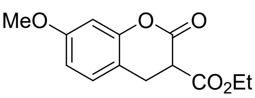

ethyl acetate 5/1). ^1H NMR (400 MHz, $CDCl_3$) δ 7.18-6.98 (m, 1H), 6.82 -6.34 (m, 2H), 4.30-4.09 (m, 2H), 3.94-3.60 (m, 4H), 3.39-3.23 (m, 1H), 3.19-2.99 (m, 1H), 1.20 (t, J = 7.1 Hz, 3H). ^{13}C NMR (100 MHz, $CDCl_3$) δ 167.6, 164.8, 160.0, 152.1, 128.7, 112.4, 110.9, 102.4, 62.1, 55.6, 46.6, 26.7, 14.0. The HRMS Calculated for $C_{13}H_{15}O_5$ $[M+H]^+$ 251.0914, found: 251.0932.

Ethyl 2-oxo-3,4-dihydro-2H-benzo[g]chromene-3-carboxylate (1p):

Reaction conducted at 1.05 mmol scale. 0.151 g, 31% yield over two steps, white solid, new com-

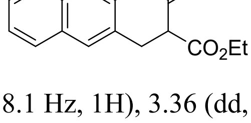

pound, mp 99-100 °C, R_f = 0.60 (hexanes/ethyl acetate 5/1). ^1H NMR (400 MHz, $CDCl_3$) δ 7.79 (d, J = 8.0 Hz, 2H), 7.70 (s, 1H), 7.57-7.39 (m, 3H), 4.26-4.11 (m, 2H), 3.84 (dd, J = 8.0, 5.8 Hz, 1H), 3.58 (dd, J = 15.7, 8.1 Hz, 1H), 3.36 (dd, J = 15.9, 5.6 Hz, 1H), 1.19 (t, J = 7.1 Hz, 3H). ^{13}C NMR (100 MHz, $CDCl_3$) δ 167.4, 164.9, 149.4, 133.2, 130.7, 127.5, 127.44, 127.38, 126.8, 125.8, 121.0, 113.2, 62.3, 46.7, 27.9, 14.0. HRMS Calculated for $C_{16}H_{15}O_4$ $[M+H]^+$ 271.0965, found: 271.0970.

General Procedure for the Synthesis of Lactones 1 with Sodium Cyanoborohydride:

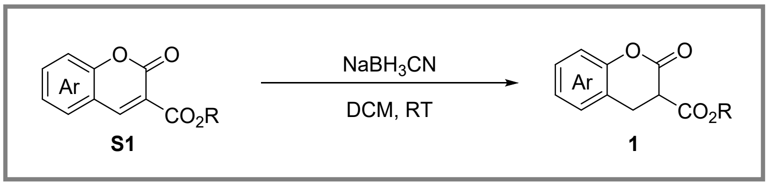

To a solution of the coumarin-3-carboxylates **S1** (7 mmol) in dichloromethane (5 mL), sodium cyanoborohydride (0.660 g, 10.5 mmol) was added slowly at room temperature. Then the mixture was stirred at room temperature until the completion of reduction (monitored by TLC). The volatiles were removed under the reduced pressure. Then, the crude residue was purified by silica gel column using hexanes/ethyl acetate (5:1) as eluent to afford the desired substrate lactones **1**.

Benzyl 2-oxochromane-3-carboxylate (1e):

0.416 g, 18% yield over two steps, white solid, new compound, mp 61-62 °C, R_f = 0.40 (hexanes/

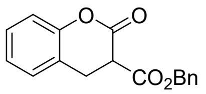

ethyl acetate 5/1). ^{1}H NMR (400 MHz, $CDCl_3$) δ 7.33-7.28 (m, 3H), 7.27-7.20 (m, 3H), 7.19-7.15 (m, 1H), 7.12-7.07 (m, 1H), 7.06-7.02 (m, 1H), 5.17 (q, J = 12.3 Hz, 2H), 3.80 (dd, J = 8.5, 6.1 Hz, 1H), 3.41 (dd, J = 16.0, 8.5 Hz, 1H), 3.17 (dd, J = 16.0, 6.0 Hz, 1H). ^{13}C NMR (100 MHz, $CDCl_3$) δ 167.5, 164.7, 151.4, 134.9, 128.8, 128.6, 128.5, 128.3, 128.1, 124.9, 120.6, 116.9, 67.8, 46.4, 27.3. The HRMS Calculated for $C_{17}H_{15}O_4$ $[M+H]^+$ 283.0965, found: 283.0976.

Ethyl 7-chloro-2-oxochromane-3-carboxylate (1k):

0.366 g, 21% yield over two steps, white solid, new compound, mp 81-82 °C, R_f= 0.60 (hexanes/

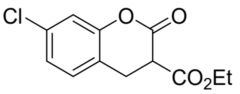

ethyl acetate 5/1). ^{1}H NMR (400 MHz, $CDCl_3$) δ 7.22-6.96 (m, 3H), 4.26-4.15 (m, 2H), 3.76 (dd, J = 8.0, 6.1 Hz), 3.38 (dd, J = 16.1, 8.0 Hz), 3.15 (dd, J = 16.1, 6.1 Hz), 1.21 (t, J = 7.1 Hz). ^{13}C NMR (100 MHz, $CDCl_3$) δ 167.2, 164.0, 151.7, 134.0, 129.2, 125.0, 119.2, 117.3, 62.4, 46.1, 26.9, 14.0. HRMS Calculated for $C_{12}H_{12}ClO_4$ $[M+H]^+$ 255.0419 (^{35}Cl), found: 255.0419 (^{35}Cl). $[M+H]^+$ 257.0353 (^{37}Cl), found: 257.0393 (^{37}Cl).

2.2. Synthesis of (+/-)-Benzyl Substituted Lactones with Quaternary Stereocenter

The substrate lactones **3** with the quaternary stereocenter could be synthesized through direct alkylation of the lactones **1** with halogenated hydrocarbon according to the known methods.[4]

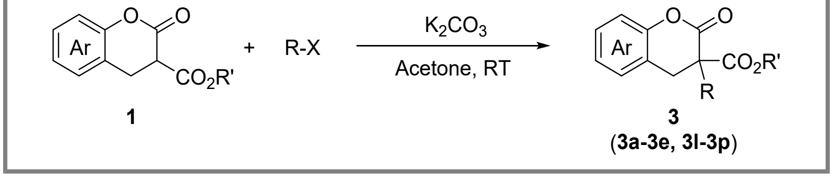

To a mixture of lactone **1** (1.5 mmol), potassium carbonate (0.622 g, 4.5 mmol) and acetone (4 mL), the halogenated hydrocarbon (1.9 mmol) was added. Then, the mixture was stirred under room temperature overnight. After the completion of the reaction (monitored by TLC), the solid was filtered, and the volatiles were removed under the reduced pressure. Then the crude residue was purified by silica gel column using hexanes/ethyl acetate (10:1-5:1) as eluent to afford the

desired products **3**.

(+/-)-Ethyl 3-benzyl-2-oxochromane-3-carboxylate (3a):

Reaction conduct under 6 mmol scale. 1.282 g, 47% yield, white solid, the known compound,[5] R_f = 0.80 (hexanes/ethyl acetate 5/1), ^{1}H NMR (400 MHz, $CDCl_3$) δ 7.31-7.19 (m, 6H), 7.14-7.00 (m, 3H), 4.09-3.97 (m, 2H), 3.50 (d, *J* = 13.8 Hz, 1H), 3.32 (d, *J* = 13.8 Hz, 1H), 3.22 (d, *J* = 15.8 Hz, 1H), 2.87 (d, *J* = 15.8 Hz, 1H), 0.99 (t, *J* = 7.1 Hz, 3H). ^{13}C NMR (100 MHz, $CDCl_3$) δ 169.5, 167.1, 151.0, 135.0, 130.8, 128.5, 128.42, 128.38, 127.4, 124.7, 121.5, 116.4, 62.2, 54.6, 39.3, 32.1, 13.8.

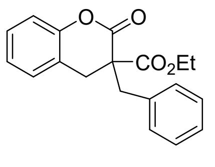

(+/-)-Ethyl 3-(4-methylbenzyl)-2-oxochromane-3-carboxylate (3b):

0.431 g, 89% yield, pale pink solid, mp 110-111 °C, new compound, R_f = 0.80 (hexanes/ethyl acetate 5/1). ^{1}H NMR (400 MHz, $CDCl_3$) δ 7.24-7.00 (m, 8H), 4.11-3.96 (m, 2H), 3.46 (d, *J* = 13.8 Hz, 1H), 3.31-3.18 (m, 2H), 2.87 (d, *J* = 15.8 Hz, 1H), 2.32 (s, 3H), 1.01 (t, *J* = 7.1 Hz, 3H). ^{13}C NMR (100 MHz, $CDCl_3$) δ 169.6, 167.1, 151.0, 137.0, 131.9, 130.6, 129.1, 128.5, 128.4, 124.6, 121.5, 116.4, 62.2, 54.6, 38.9, 32.0, 21.1, 13.8. HRMS Calculated for $C_{20}H_{21}O_4$ $[M+H]^+$ 325.1434, found: 325.1437.

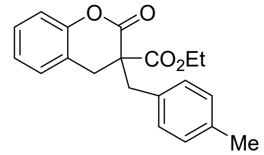

(+/-)-Ethyl 3-(4-fluorobenzyl)-2-oxochromane-3-carboxylate (3c):

0.370 g, 75% yield, colorless oil, new compound, R_f = 0.70 (hexanes/ethyl acetate 5/1). ^{1}H NMR (400 MHz, $CDCl_3$) δ 7.29-7.20 (m, 3H), 7.18-6.90 (m, 5H), 4.14-3.91 (m, 2H), 3.45 (d, *J* = 13.9 Hz, 1H), 3.33-3.17 (m, 2H), 2.87 (d, *J* = 15.7 Hz, 1H), 0.99 (t, *J* = 7.1 Hz, 3H). ^{13}C NMR (100 MHz, $CDCl_3$) δ 169.4, 167.0, 162.2 (d, $^1J_{F\text{-}C}$ = 245.8 Hz). 150.9, 132.3 (d, $^3J_{F\text{-}C}$ = 8.0 Hz), 130.7 (d, $^4J_{F\text{-}C}$ = 3.5 Hz), 128.5 (d, $^3J_{F\text{-}C}$ = 21.9 Hz), 124.7, 121.2, 116.4, 115.2 (d, $^2J_{F\text{-}C}$ = 21.4 Hz), 62.3, 54.6, 38.5, 32.2, 13.7. ^{19}F NMR (376 MHz, $CDCl_3$) δ -115.21. HRMS Calculated for $C_{19}H_{18}FO_4$ $[M+H]^+$ 329.1184, found: 329.1183.

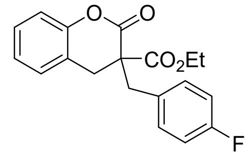

(+/-)-Ethyl 3-(naphthalen-2-ylmethyl)-2-oxochromane-3-carboxylate (3d):

0.476 g, 88% yield, pale yellow solidm, mp 123-124 °C, new compound, R_f = 0.70 (hexanes/ethyl acetate 5/1). ^{1}H NMR (400 MHz, $CDCl_3$) δ 7.84-7.68 (m, 4H), 7.51-7.37 (m, 3H), 7.24-7.17 (m, 1H), 7.13-6.96 (m, 3H), 4.13-3.96 (m, 2H), 3.58 (dd, *J* = 76.6, 13.8 Hz, 2H), 3.26 (d, *J* = 15.8 Hz, 1H), 2.89 (d, *J* = 15.8 Hz, 1H), 0.99 (t, *J* = 7.1 Hz, 3H). ^{13}C NMR (100 MHz, $CDCl_3$) δ 169.6, 167.2, 151.0, 133.3, 132.62, 132.60, 129.7, 128.7, 128.5, 128.4, 127.9, 127.8, 127.6, 126.2, 126.0, 124.7, 121.4, 116.4, 62.3, 54.7, 39.4, 32.1, 13.8. HRMS Calculated for $C_{23}H_{21}O_4$ $[M+H]^+$ 361.1434, found: 361.1431.

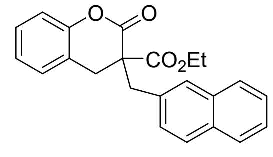

(+/-)-Ethyl 3-methyl-2-oxochromane-3-carboxylate (3e):

0.346 g, 99% yield, white solid, mp 92-93 °C, known compound,[4] R_f = 0.60 (hexanes/ethyl acetate 10/1). ^{1}H NMR (400 MHz, $CDCl_3$) δ 7.29-7.23 (m, 1H), 7.18 (d, *J* = 7.3 Hz, 1H), 7.14-6.98 (m, 2H), 4.14-3.94 (m, 2H), 3.36 (d, *J* =

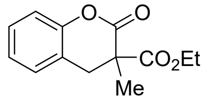

15.8 Hz, 1H), 2.99 (d, J = 15.8 Hz, 1H), 1.62 (s, 3H), 1.00 (t, J = 7.1 Hz, 3H). ^{13}C NMR (100 MHz, $CDCl_3$) δ 170.6, 168.2, 151.5, 128.7, 128.2, 124.6, 121.3, 116.5, 62.1, 49.5, 35.4, 20.9, 13.7.

(+/-)-Methyl 3-benzyl-2-oxochromane-3-carboxylate (3l):

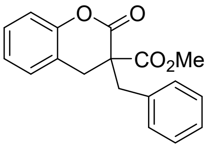

0.275 g, 59% yield, white solid, mp 87-88 °C, new compound, R_f = 0.30 (hexanes/ethyl acetate 30/1). ^{1}H NMR (400 MHz, $CDCl_3$) δ 7.34-7.20 (m, 6H), 7.15-6.98 (m, 3H), 3.60 (s, 3H), 3.40 (dd, J = 41.2, 13.8 Hz, 2H), 3.07 (dd, J = 154.4, 15.9 Hz, 2H). ^{13}C NMR (100 MHz, $CDCl_3$) δ 169.9, 166.9, 150.8, 134.9, 130.7, 128.6, 128.5, 128.4, 127.5, 124.8, 121.3, 116.4, 54.5, 53.1, 39.5, 31.8. HRMS Calculated for $C_{18}H_{17}O_4$ $[M+H]^+$ 297.1121, found: 297.1131.

(+/-)-Isopropyl 3-benzyl-2-oxochromane-3-carboxylate (3m):

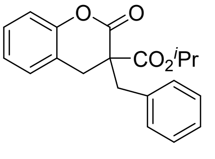

0.396 g, 81% yield, pale yellow solid, mp 101-102 °C, new compound, R_f = 0.75 (hexanes/ethyl acetate 10/1). ^{1}H NMR (400 MHz, $CDCl_3$) δ 7.34-7.26 (m, 5H), 7.24-7.18 (m, 1H), 7.12 (d, J = 7.1 Hz, 1H), 7.09-6.98 (m, 2H), 4.91-4.76 (m, 1H), 3.51 (d, J = 13.8 Hz, 1H), 3.30 (d, J = 13.8 Hz, 1H), 3.19 (d, J = 15.7 Hz, 1H), 2.87 (d, J = 15.7 Hz, 1H), 1.04 (d, J = 6.3 Hz, 3H), 0.87 (d, J = 6.2 Hz, 3H). ^{13}C NMR (100 MHz, $CDCl_3$) δ 169.1, 167.3, 151.1, 135.2, 130.8, 128.5, 128.4, 128.3, 127.3, 124.6, 121.6, 116.3, 70.1, 54.7, 39.1, 32.3, 21.2, 21.1. HRMS Calculated for $C_{20}H_{21}O_4$ $[M+H]^+$ 325.1434, found: 325.1436.

(+/-)-Ethyl 3-benzyl-7-methyl-2-oxochromane-3-carboxylate (3n):

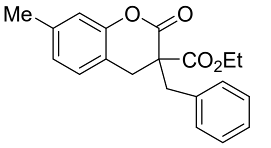

0.311 g, 64% yield, white solid, mp 104-105 °C, new compound, R_f = 0.75 (hexanes/ethyl acetate 5/1). ^{1}H NMR (400 MHz, $CDCl_3$) δ 7.30-7.25 (m, 5H), 7.03-6.95 (m, 1H), 6.93-6.78 (m, 2H), 4.12-3.97 (m, 2H), 3.39 (dd, J = 57.7, 13.8 Hz, 2H), 3.19 (d, J = 15.8 Hz, 1H), 2.82 (d, J = 15.8 Hz, 1H), 2.30 (s, 3H), 1.02 (t, J = 7.1 Hz, 3H). ^{13}C NMR (100 MHz, $CDCl_3$) δ 169.6, 167.3, 150.8, 138.7, 135.1, 130.8, 128.3, 128.1, 127.3, 125.4, 118.2, 116.8, 62.2, 54.7, 39.3, 31.7, 21.2, 13.8. HRMS Calculated for $C_{20}H_{21}O_4$ $[M+H]^+$ 325.1434, found: 325.1440.

(+/-)-Ethyl 3-benzyl-7-chloro-2-oxochromane-3-carboxylate (3o):

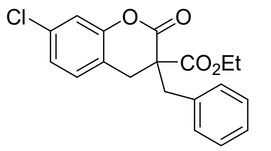

0.378 g, 73% yield, white solid, mp 125-126 °C, new compound, R_f = 0.65 (hexanes/ethyl acetate 10/1). ^{1}H NMR (400 MHz, $CDCl_3$) δ 7.32-7.24 (m, 5H), 7.13-6.94 (m, 3H), 4.18-3.90 (m, 2H), 3.48 (d, J = 13.8 Hz, 1H), 3.31 (d, J = 13.8 Hz, 1H), 3.21 (d, J = 15.9 Hz, 1H), 2.83 (d, J = 15.9 Hz, 1H), 1.05 (t, J = 7.1 Hz, 3H). ^{13}C NMR (100 MHz, $CDCl_3$) δ 169.2, 166.3, 151.3, 134.7, 133.7, 130.7, 129.3, 128.5, 127.5, 124.8, 120.1, 116.8, 62.5, 54.4, 39.4, 31.6, 13.8. HRMS Calculated for $C_{19}H_{17}ClO_4Na$ $[M+Na]^+$ 367.0708 (^{35}Cl), found: 367.0705 (^{35}Cl). $[M+H]^+$ 369.0691 (^{37}Cl), found: 369.0680 (^{37}Cl).

(+/-)-Ethyl 3-benzyl-7-methoxy-2-oxochromane-3-carboxylate (3p):

0.433 g, 85% yield, white solid, mp 127-128 °C, new compound, R_f = 0.70 (hexanes/ethyl acetate 5/1). ^{1}H NMR (400 MHz, $CDCl_3$) δ 7.31-7.23 (m, 5H), 7.00 (d, J = 8.3 Hz, 1H), 6.68-6.50 (m,

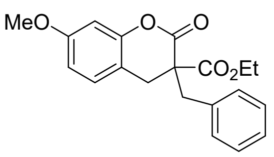

2H), 4.11-3.97 (m, 2H), 3.75 (s, 3H), 3.46 (d, *J* = 13.8 Hz, 1H), 3.31 (d, *J* = 13.8 Hz, 1H), 3.16 (d, *J* = 15.6 Hz, 1H), 2.80 (d, *J* = 15.6 Hz, 1H), 1.03 (t, *J* = 7.1 Hz, 3H). ^{13}C NMR (100 MHz, $CDCl_3$) δ 169.6, 167.1, 159.8, 151.7, 135.1, 130.8, 128.9, 128.3, 127.4, 113.2, 110.7, 102.0, 62.2, 55.5, 54.8, 39.3, 31.4, 13.8. HRMS Calculated for $C_{20}H_{21}O_5$ $[M+H]^+$ 341.1384, found: 341.1385.

2.3. Synthesis of (+/-)-Ethyl 3-(2-Methoxyethyl)-2-oxochromane-3-carboxylate (3h)

The lactone **3h** with quaternary stereogenic center could be conveniently synthesized through alkylation of lactone **1a** with 1-bromo-2-methoxyethane in the presence of potassium carbonate.

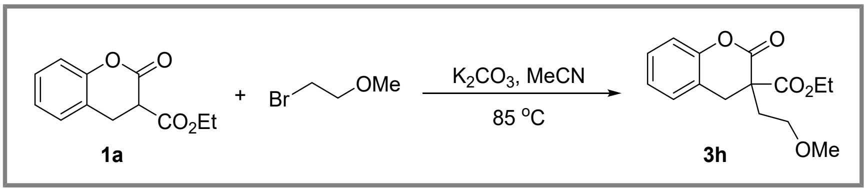

To a mixture of lactone **1a** (0.327 g, 1.5 mmol), potassium carbonate (0.622 g, 4.5 mmol) and acetonitrile (5 mL), 1-bromo-2-methoxyethane (0.18 mL, 1.9 mmol) was added. Then the mixture was refluxed under 85 ^{o}C overnight. After the completion of the alkylation (monitored by TLC), the solid was filtered and the volatiles were removed under the reduced pressure. Then the crude residue was purified by silica gel column using hexanes/ethyl acetate (10:1-5:1) as eluent to afford the desired product **3h**.

0.245 g, 58% yield, colorless oil, new compound, R_f = 0.50 (hexanes/ethyl acetate 5/1). ^{1}H NMR (400 MHz, $CDCl_3$) δ 7.27-7.16 (m, 2H), 7.12-7.01 (m, 2H), 4.12-3.96 (m, 2H), 3.64 (t, *J* = 6.1 Hz, 2H), 3.37 (d, *J* = 15.9 Hz, 1H), 3.29 (s, 3H), 3.11 (d, *J* = 15.8 Hz, 1H), 2.28 (t, *J* = 6.1 Hz, 2H), 1.02 (t, *J* = 7.1 Hz, 3H). ^{13}C NMR (100 MHz, $CDCl_3$) δ 169.6, 167.6, 151.1, 128.6, 128.4, 124.6, 121.5, 116.4, 68.8, 62.1, 58.6, 52.0, 34.1, 33.4, 13.7. HRMS Calculated for $C_{15}H_{19}O_5$ $[M+H]^+$ 279.1227, found: 279.1227.

2.4. Synthesis of (+/-)-Alkyl Substituted Lactones with Quaternary Center

The substrate lactones with quaternary carbon center **3** were synthesized through alkylation of the above lactone **1a** with alkyl halides.

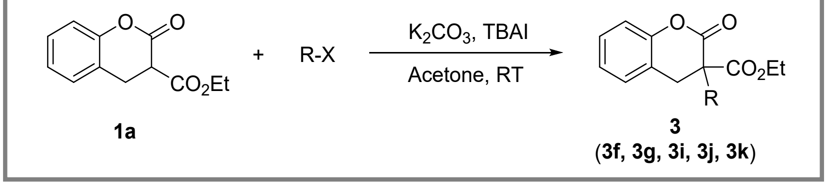

To a mixture of lactone **1a** (0.327 g, 1.5 mmol), potassium carbonate (0.622 g, 4.5 mmol), *tetra*-butylammonium iodide (0.554 g, 1.5 mmol) and acetone (4.0 mL), halogenated hydrocarbons (1.9 mmol) was added. Then the mixture was stirred under room temperature overnight. After the completion of the reaction (monitored by TLC), the solid was filtered, and the volatiles were

removed under the reduced pressure. Then, the crude residue was purified by silica gel column using hexanes/ethyl acetate (10:1-5:1) as eluent to afford the desired products **3**.

(+/-)-Ethyl 3-ethyl-2-oxochromane-3-carboxylate (3f):

0.200 g, 54% yield, colorless oil, known compound,[4] R_f = 0.70 (hexanes/ethyl acetate 10/1). ^{1}H NMR (400 MHz, $CDCl_3$) δ 7.26-7.16 (m, 2H), 7.12-6.96 (m, 2H), 4.14-3.94 (m, 2H), 3.29 (d, *J* = 15.7 Hz, 1H), 3.01 (d, *J* = 15.7 Hz, 1H), 2.13-1.97 (m, 2H), 1.10-0.96 (m, 6H). ^{13}C NMR (100 MHz, $CDCl_3$) δ 169.8, 167.5, 151.2, 128.6, 128.4, 124.6, 121.5, 116.4, 61.9, 53.6, 32.3, 27.4, 13.8, 9.1.

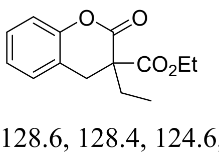

(+/-)-Ethyl 3-methyl-2-oxochromane-3-carboxylate (3g):

0.366 g, 88% yield, colorless oil, new compound, R_f = 0.70 (hexanes/ethyl acetate 10/1). ^{1}H NMR (400 MHz, $CDCl_3$) δ 7.25-7.12 (m, 2H), 7.11-6.96 (m, 2H), 4.12-3.93 (m, 2H), 3.29 (d, *J* = 15.7 Hz, 1H), 3.01 (d, *J* = 15.7 Hz, 1H), 2.05-1.89 (m, 2H), 1.54-1.44 (m, 1H), 1.40-1.28 (m, 3H), 1.00 (t, *J* = 7.1 Hz, 3H), 0.91 (t, *J* = 7.1 Hz, 3H). ^{13}C NMR (100 MHz, $CDCl_3$) δ 169.9, 167.6, 151.2, 128.5, 128.3, 124.6, 121.6, 116.4, 61.9, 53.3, 34.0, 32.7, 26.7, 23.0, 13.9, 13.8. The HRMS Calculated for $C_{16}H_{21}O_4$ $[M+H]^+$ 277.1434, found: 277.1437.

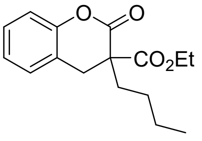

(+/-)-Ethyl 3-(cyclopropylmethyl)-2-oxochromane-3-carboxylate (3i):

0.171 g, 42% yield, colorless oil, new compound, R_f = 0.70 (hexanes/ethyl acetate 10/1). ^{1}H NMR (400 MHz, $CDCl_3$) δ 7.26-7.15 (m, 2H), 7.11-6.97 (m, 2H), 4.12-3.96 (m, 2H), 3.38 (d, *J* = 15.8 Hz, 1H), 3.19 (d, *J* = 15.8 Hz, 1H), 2.00-1.87 (m, 2H), 1.03 (t, *J* = 7.1 Hz, 3H), 0.99-0.85 (m, 1H), 0.55-0.44 (m, 2H), 0.16-0.04 (m, 2H). ^{13}C NMR (100 MHz, $CDCl_3$) δ 169.8, 167.7, 151.1, 128.5, 128.4, 124.6, 121.8, 116.4, 62.0, 53.9, 39.0, 32.7, 13.8, 6.6, 4.9, 4.4. HRMS Calculated for $C_{16}H_{18}O_4K$ $[M+K]^+$ 313.0837, found: 313.0840.

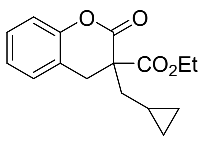

(+/-)-Ethyl 3-(cyclobutylmethyl)-2-oxochromane-3-carboxylate (3j):

0.143 g, 33% yield, colorless oil, new compound, R_f = 0.70 (hexanes/ethyl acetate 10/1). ^{1}H NMR (400 MHz, $CDCl_3$) δ 7.26-7.14 (m, 2H), 7.11-6.96 (m, 2H), 4.10-3.92 (m, 2H), 3.31 (d, *J* = 15.8 Hz, 1H), 2.94 (d, *J* = 15.7 Hz, 1H), 2.67-2.47 (m, 1H), 2.19-1.99 (m, 4H), 1.90-1.66 (m, 4H), 1.02 (t, *J* = 7.1 Hz, 3H). ^{13}C NMR (100 MHz, $CDCl_3$) δ 169.7, 167.6, 151.0, 128.5, 128.4, 124.6, 121.7, 116.4, 62.0, 53.1, 41.0, 32.6, 32.4, 29.9, 29.6, 18.9, 13.8. HRMS Calculated for $C_{17}H_{21}O_4$ $[M+H]^+$ 289.1434, found: 289.1437.

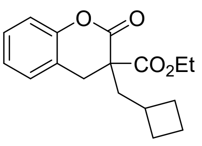

(+/-)-Ethyl 3-(cyclohexylmethyl)-2-oxochromane-3-carboxylate (3k):

0.288 g, 61% yield, colorless oil, new compound, R_f = 0.70 (hexanes/ethyl acetate 10/1). ^{1}H NMR (400 MHz, $CDCl_3$) δ 7.25-7.14 (m, 2H), 7.11-6.98 (m, 2H), 4.10-3.96 (m, 2H), 3.33 (d, *J* = 15.7 Hz, 1H), 3.02 (d, *J* = 15.7 Hz, 1H), 1.96-1.87 (m, 2H), 1.74-1.58 (m, 5H), 1.55-1.43 (m, 1H), 1.26-1.08 (m, 3H), 1.07-0.95 (m, 5H). ^{13}C NMR (100 MHz, $CDCl_3$) δ 170.2, 167.7, 151.1, 128.5, 128.4, 124.6,

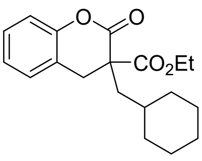

121.6, 116.4, 62.0, 53.1, 40.8, 34.7, 34.4, 34.3, 32.7, 26.3, 26.1, 13.7. HRMS Calculated for $C_{19}H_{25}O_4$ $[M+H]^+$ 317.1747, found: 317.1758.

---

3. Condition Optimization for Kinetic Resolution

General Procedure: The $Pd(OCOCF_3)_2$ (0.015 mmol, 4.8 mg, 5 mol%), ligand (0.018 mmol, 6 mol%) and degassed anhydrous acetone (1.0 mL) were placed in a dried Schlenk tube under nitrogen atmosphere. The mixture was stirred under room temperature for 1 hour, the solvent was removed under vacuum to give the catalyst. Then, this catalyst was taken into a glove box filled with nitrogen gas and dissolved in solvent (1.0 mL). To the lactones **3a** (0.3 mmol, 93.0 mg) added this catalyst solution, and then the mixture was transferred to an autoclave, which was charged hydrogen gas (400 psi). The reaction mixture was stirred at 40 °C for 48 hours. After cooling to room temperature and the release of the hydrogen gas, the autoclave was opened. To the mixture added sodium cyanoborohydride (7.5 mg, 0.12 mmol). The mixture was stirred under room temperature for 2 hours, then the volatiles were removed under the reduced pressure. The crude residue was purified by column chromatography on silica gel using hexanes/ethyl acetate (10:1-5:1) as eluent to give the chiral reductive products **3a'** and **4a**.

The optimal conditions were identified as follow: Lactone **3a** (0.3 mmol), $Pd(OCOCF_3)_2$ (5 mol%), ligand **L5** (6 mol%), H_2 (400 psi), HFIP (1.0 mL), 40 °C, 48 h (entry 8).

Table S1. Optimization of the KR Reaction Conditions

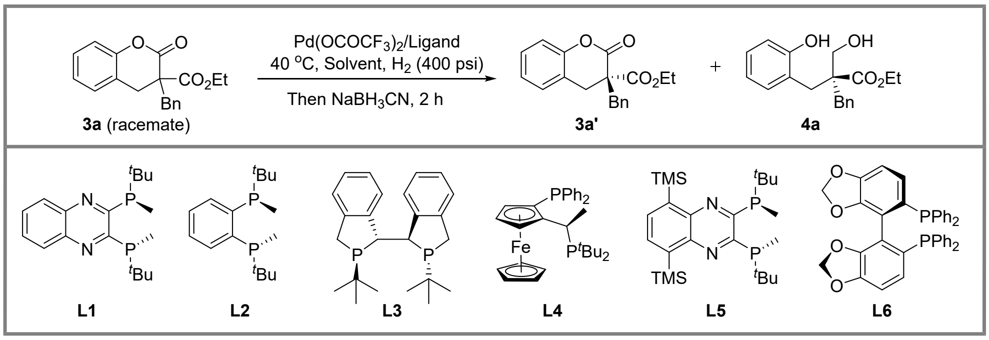

Entry[a]	Ligand	Solvent	Conv. [%][b]	Yield of **4a**[b]	Ee [%][c] 3a'	Ee [%][c] 4a	S[d]
1	**L1**	TFE	13	12	31	77	10.6
2	**L1**	DCM	<5	<5	-	-	-
3	**L1**	HFIP	54	52	89	72	17.6
4	**L1**	Toluene	<5	<5	-	-	-
5	**L2**	HFIP	62	61	96	50	11.3
6	**L3**	HFIP	54	54	95	56	12.3
7	**L4**	HFIP	>95	>95	-	-	-
8	**L5**	HFIP	53	54 (52)[e]	93	71	19.3
9	**L6**	HFIP	<5	<5	-	-	-
10	**L5**	HFIP/Toluene = 5:1	42	42	59	82	18.4
11	**L5**	HFIP/DCM = 5:1	53	52	77	75	16.2
12	**L5**	HFIP/TFE = 5:1	55	54	84	76	18.9

[a] Lactone **3a** (0.3 mmol), $Pd(OCOCF_3)_2$ (5 mol%), chiral ligand (6 mol%), H_2 (400 psi), solvent (1 mL), 40 °C, 48 h. [b]Determined by NMR, using CH_2Br_2 as internal standard. [c]Determined by HPLC. [d]Calculated selectivity factors: C = ee of SM/(ee of SM + ee of TM), s = ln[(1 - C)(1 - ee of SM)]/ln[(1 - C)(1 + ee of SM)]. [e]Isolated yield based on the lactone **3a**.

4. Pd-catalyzed Hydrogenative DKR and KR of Lactones

4.1 Pd-catalyzed Hydrogenative Dynamic Kinetic Resolution of Lactones

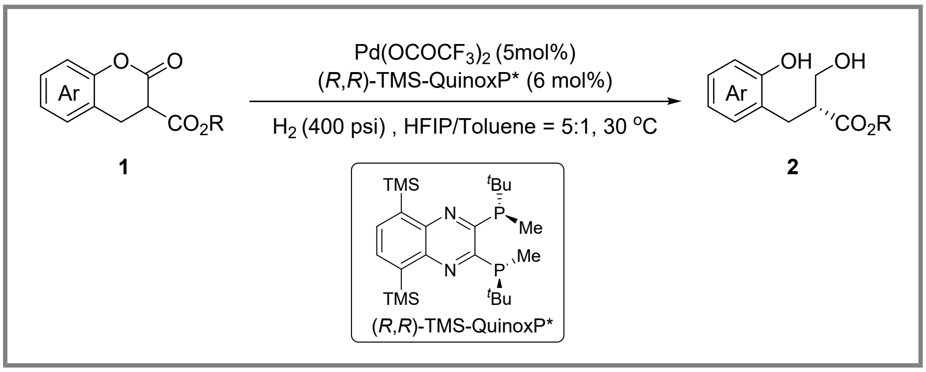

General Procedure: $Pd(OCOCF_3)_2$ (0.01 mmol, 3.2 mg, 5 mol%), chiral ligand (*R,R*)-TMS-QuinoxP* (0.012 mmol, 5.8 mg, 6 mol%) and degassed anhydrous acetone (1.0 mL) were placed in a dried Schlenk tube under nitrogen atmosphere. The mixture was stirred under room temperature for 1 hour, the solvent was removed under vacuum to give the catalyst. Then, this catalyst was taken into a glove box filled with nitrogen and dissolved in solvent (HFIP/toluene = 5:1, 1.0 mL). To the lactones **1** (0.2 mmol) was added the catalyst solution, and the mixture was transferred to an autoclave, which was charged hydrogen gas (400 psi). The reaction mixture was stirred at 30 °C for 48 hours. After release of the hydrogen gas, the autoclave was opened, and the volatiles were removed under the reduced pressure. Then the residue was purified by column chromatography on silica gel using dicholormethane/methanol (40:1) as eluent to give the reductive products **2**.

The racemates were prepared by reduction of lactones **1** with sodium borohydride in methanol.

(+)-Ethyl 3-hydroxy-2-(2-hydroxybenzyl)propanoate (2a):

44 mg, 98% yield, colorless oil, new compound, R_f = 0.30 (dichloromethane/methanol 40/1), 91% ee, $[\alpha]^{20}_D$ = +11.28 (*c* 0.86, $CHCl_3$). ^{1}H NMR (400 MHz, $CDCl_3$) δ 7.54 (brs, 1H), 7.19-6.99 (m, 2H), 6.97-6.70 (m, 2H), 4.31-4.09 (m, 2H), 3.95-3.80 (m, 1H), 3.69-3.58 (m, 1H), 3.40 (brs, 1H), 3.09-2.91 (m, 2H), 2.88-2.74 (m, 1H), 1.26 (t, *J* = 7.1 Hz, 3H). ^{13}C NMR (100 MHz, $CDCl_3$) δ 175.2, 154.9, 131.1, 128.3, 124.6, 120.8, 116.7, 61.5, 61.2, 47.7, 28.1, 14.2. HPLC: Chiralpak AS-H column, 230 nm, 30 °C, *n*-Hexane/*i*-Pr OH = 90/10, flow = 0.8 mL/min, retention time 16.6 min and 19.3 min (major). HRMS Calculated for $C_{12}H_{17}O_4$ $[M+H]^+$ 225.1121, found: 225.1120.

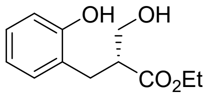

(+)-Methyl 3-hydroxy-2-(2-hydroxybenzyl)propanoate (2b):

33 mg, 79% yield, colorless oil, new compound, R_f = 0.30 (dichloromethane/methanol 40/1), 83% ee, $[\alpha]^{20}_D$ = +9.85 (*c* 0.66, $CHCl_3$). ^{1}H NMR (400 MHz, $CDCl_3$) δ 7.46 (brs, 1H), 7.15-7.06 (m, 2H), 6.90-6.80 (m, 2H), 3.91-3.85 (m, 1H), 3.73 (s, 3H), 3.67-3.60 (m, 1H), 3.36 (brs, 1H), 3.06-2.94 (m, 2H), 2.88-2.80 (m, 1H). ^{13}C NMR (100 MHz, $CDCl_3$) δ 175.6, 154.8, 131.1, 128.3, 124.5, 120.8, 116.7, 61.4, 52.2, 47.6, 28.1. The HPLC: Chiralpak IB column, 230 nm, 30 °C, *n*-Hexane/*i*-PrOH = 90/10, flow = 0.8 mL/min, retention time 17.2 min (major) and 18.7 min. HRMS Calculated for $C_{11}H_{15}O_4$ $[M+H]^+$ 211.0965, found: 211.0970.

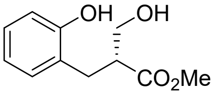

(+)-Isopropyl 3-hydroxy-2-(2-hydroxybenzyl)propanoate (2c):

46 mg, 97% yield, colorless oil, new compound, R_f = 0.35 (dichloromethane/methanol 40/1), 89%

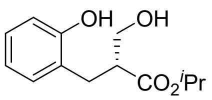

ee, $[\alpha]^{20}_D$ = +13.59 (*c* 0.92, $CHCl_3$). ^1H NMR (400 MHz, $CDCl_3$) δ 7.58 (brs, 1H), 7.16-7.02 (m, 2H), 6.94-6.74 (m, 2H), 5.15-4.96 (m, 1H), 3.88 (dd, *J* = 11.5, 3.8 Hz, 1H), 3.62 (dd, *J* = 11.4, 4.5 Hz, 1H), 3.39 (brs, 1H), 3.08-2.91 (m, 2H), 2.84-2.69 (m, 1H), 1.32-1.16 (m, 6H). ^{13}C NMR (100 MHz, $CDCl_3$) δ 174.8, 154.9, 131.1, 128.3, 124.6, 120.7, 116.8, 68.8, 61.5, 47.8, 28.2, 21.8, 21.7. The HPLC: Chiralpak AS-H column, 230 nm, 30 °C, *n*-Hexane/*i*-PrOH = 90/10, flow = 0.8 mL/min, retention time 12.8 min and 15.2 min (major). HRMS Calculated for $C_{13}H_{19}O_4$ $[M+H]^+$ 239.1278, found: 239.1280.

(+)-*tert*-Butyl 3-hydroxy-2-(2-hydroxybenzyl)propanoate (2d):

44 mg, 87% yield, colorless oil, new compound, R_f = 0.37 (dichloromethane/methanol 40/1), 81%

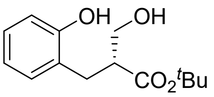

ee, $[\alpha]^{20}_D$ = +13.41 (*c* 0.88, $CHCl_3$). ^1H NMR (400 MHz, $CDCl_3$) δ 7.51 (brs, 1H), 7.20-7.05 (m, 2H), 6.98-6.74 (m, 2H), 3.89 (dd, *J* = 11.5, 3.4 Hz, 1H), 3.55 (dd, *J* = 11.2, 3.2 Hz, 1H), 3.19 (brs, 1H), 3.08-2.88 (m, 2H), 2.78-2.61 (m, 1H), 1.47 (s, 9H). ^{13}C NMR (100 MHz, $CDCl_3$) δ 174.9, 155.2, 131.1, 128.3, 124.7, 120.7, 117.2, 82.1, 61.5, 48.3, 28.3, 28.1. HPLC: Chiralpak AS-H column, 230 nm, 30 °C, *n*-Hexane/*i*-Pr OH = 95/5, flow = 0.8 mL/min, retention time 14.8 min and 16.8 min (major). HRMS Calculated for $C_{14}H_{20}O_4Na$ $[M+Na]^+$ 275.1259, found: 275.1249.

(+)-Benzyl 3-hydroxy-2-(2-hydroxybenzyl)propanoate (2e):

56 mg, 99% yield, white solid, new compound, mp 78-79 °C, R_f = 0.20 (dichloromethane/me-

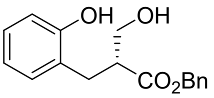

thanol 40/1), 88% ee, $[\alpha]^{20}_D$ = +7.82 (*c* 1.12, $CHCl_3$). ^1H NMR (400 MHz, $CDCl_3$) δ 7.71-7.27 (m, 6H), 7.15-7.04 (m, 2H), 6.91-6.77 (m, 2H), 5.24-5.09 (m, 2H), 3.91 (dd, *J* = 11.4, 3.7 Hz, 1H), 3.66 (dd, *J* = 11.5, 4.5 Hz, 1H), 3.31 (brs, 1H), 3.10-2.98 (m, 2H), 2.93-2.85 (m, 1H). ^{13}C NMR (100 MHz, $CDCl_3$) δ 174.9, 154.8, 135.5, 131.1, 128.7, 128.5, 128.33, 128.30, 124.5, 120.9, 116.7, 66.9, 61.4, 47.7, 28.1. The HPLC: Chiralpak AS-H column, 230 nm, 30 °C, *n*-Hexane/*i*-PrOH = 90/10, flow = 0.8 mL/min, retention time 22.6 min (minor) and 24.4 min (major). HRMS Calculated for $C_{17}H_{19}O_4$ $[M+H]^+$ 287.1279, found: 287.1279.

(+)-Ethyl 3-hydroxy-2-(2-hydroxy-3-methylbenzyl)propanoate (2f):

47 mg, 99% yield, pale yellow oil, new compound, R_f = 0.40 (dichloromethane/methanol 40/1),

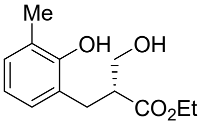

72% ee, $[\alpha]^{20}_D$ = +8.08 (*c* 0.94, $CHCl_3$). ^1H NMR (400 MHz, $CDCl_3$) δ 7.24 (brs, 1H), 7.00 (dd, *J* = 21.8, 7.4 Hz, 2H), 6.83-6.71 (m, 1H), 4.22 (q, *J* = 7.1 Hz, 2H), 3.93 (dd, *J* = 11.5, 3.3 Hz, 1H), 3.58 (d, *J* = 8.9 Hz, 1H), 3.11 (brs, 1H), 3.07-2.96 (m, 2H), 2.79-2.72 (m, 1H), 2.26 (s, 3H), 1.30 (t, *J* = 7.1 Hz, 3H). ^{13}C NMR (100 MHz, $CDCl_3$) δ 175.5, 153.4, 129.6, 128.5, 125.9, 124.0, 120.3, 61.31, 61.26, 47.7, 28.3, 16.5, 14.2. HPLC: Chiralpak AS-H column, 230 nm, 30 °C, *n*-Hexane/*i*-PrOH = 90/10, flow = 0.8 mL/min, retention time 10.0 min and 10.6 min (major). The HRMS Calculated for $C_{13}H_{19}O_4$ $[M+H]^+$ 239.1278, found: 239.1274.

(+)-Ethyl 3-hydroxy-2-(2-hydroxy-4-methylbenzyl)propanoatee (2g):

43 mg, 90% yield, colorless oil, new compound, R_f = 0.30 (DCM/methanol 40/1), 89% ee, $[\alpha]^{20}_D$ = +9.77 (*c* 0.86, $CHCl_3$). ^{1}H NMR (400 MHz, $CDCl_3$) δ 7.45 (brs, 1H), 6.97 (d, *J* = 7.4 Hz, 1H), 6.77-6.57 (m, 2H), 4.19 (q, *J* = 7.1 Hz, 2H), 3.88 (dd, *J* = 11.4, 3.6 Hz, 1H), 3.64 (d, *J* = 7.9 Hz, 1H), 3.40 (brs, 1H), 3.05-2.89 (m, 2H), 2.85-2.73 (m, 1H), 2.26 (s, 3H), 1.27 (t, *J* = 7.1 Hz, 3H). ^{13}C NMR (100 MHz, $CDCl_3$) δ 175.3, 154.7, 138.3, 130.8, 121.6, 121.4, 117.3, 61.4, 61.2, 47.8, 27.8, 21.1, 14.2. HPLC: Chiralpak IB, 230 nm, 30 °C, *n*-Hexane/*i*-PrOH = 90/10, flow = 0.8 mL/min, retention time 21.2 min (major) and 22.8 min. HRMS Calculated for $C_{13}H_{19}O_4$ $[M+H]^+$ 239.1278, found: 239.1282.

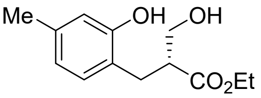

(+)-Ethyl 3-hydroxy-2-(2-hydroxy-5-methylbenzyl)propanoate (2h):

42 mg, 88% yield, colorless oil, new compound, R_f = 0.40 (dichloromethane/methanol 40/1), 90% ee, $[\alpha]^{20}_D$ = +8.33 (*c* 0.84, $CHCl_3$). ^{1}H NMR (400 MHz, $CDCl_3$) δ 6.99-6.86 (m, 2H), 6.76 (d, *J* = 8.8 Hz, 1H), 4.25-4.13 (m, 2H), 3.89 (dd, *J* = 11.5, 3.7 Hz, 1H), 3.62 (dd, *J* = 11.6, 4.5 Hz, 1H), 3.05-2.92 (m, 2H), 2.83-2.74 (m, 1H), 2.24 (s, 3H), 1.27 (t, *J* = 7.1 Hz, 3H). ^{13}C NMR (100 MHz, $CDCl_3$) δ 175.3, 152.6, 131.5, 129.9, 128.8, 124.3, 116.6, 61.4, 61.2, 47.7, 28.1, 20.5, 14.2. HPLC: Chiralpak AS-H column, 230 nm, 30 °C, *n*-Hexane/*i*-PrOH = 95/5, flow = 0.8 mL/min, retention time 25.2 min and 27.6 min (major). HRMS Calculated for $C_{13}H_{19}O_4$ $[M+H]^+$ 239.1278, found: 239.1278.

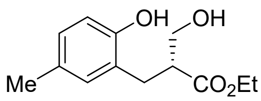

(+)-Ethyl 3-hydroxy-2-(2-hydroxy-6-methylbenzyl)propanoate (2i):

41 mg, 86% yield, colorless oil, new compound, R_f = 0.40 (dichloromethane/methanol 40/1), 80% ee, $[\alpha]^{20}_D$ = +17.19 (*c* 0.82, $CHCl_3$). ^{1}H NMR (400 MHz, $CDCl_3$) δ 7.58 (brs, 1H), 7.08-6.95 (m, 1H), 6.75 (d, *J* = 7.8 Hz, 2H), 4.22 (q, *J* = 7.1 Hz, 2H), 4.04-3.87 (m, 1H), 3.68-3.38 (m, 2H), 3.14-2.96 (m, 2H), 2.81-2.65 (m, 1H), 2.31 (s, 3H), 1.29 (t, *J* = 7.1 Hz, 3H). ^{13}C NMR (100 MHz, $CDCl_3$) δ 175.5, 155.2, 138.1, 127.6, 123.4, 122.8, 114.5, 61.6, 61.3, 45.9, 24.4, 19.4, 14.2. HPLC: Chiral pak AS-H column, 230 nm, 30 °C, *n*-Hexane/*i*-PrOH = 90/10, flow = 0.8 mL/min, retention time 13.2 min and 16.6 min (major). HRMS Calculated for $C_{13}H_{19}O_4$ $[M+H]^+$ 239.1278, found: 239.1272.

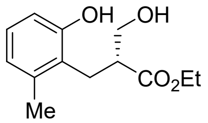

(+)-Ethyl 2-(5-fluoro-2-hydroxybenzyl)-3-hydroxypropanoate (2j):

47 mg, 97% yield, pale yellow oil, new compound, R_f = 0.30 (dichloromethane/methanol 40/1), 79% ee, $[\alpha]^{20}_D$ = +12.73 (*c* 0.94, $CHCl_3$). ^{1}H NMR (400 MHz, $CDCl_3$) δ 7.38 (brs, 1H), 6.96-6.73 (m, 3H), 4.20 (q, *J* = 7.1 Hz, 2H), 3.91 (dd, *J* = 11.5, 3.6 Hz, 1H), 3.60 (dd, *J* = 11.5, 4.1 Hz, 1H), 3.21 (brs, 1H), 3.05-2.91 (m, 2H), 2.85-2.73 (m, 1H), 1.28 (t, *J* = 7.1 Hz, 3H).^{13}C NMR (100 MHz, $CDCl_3$) δ 175.0, 157.0 (d, $^1J_{F-C}$ = 238.1 Hz), 151.0 (d, $^4J_{F-C}$ = 2.0 Hz), 126.1 (d, $^3J_{F-C}$ = 7.2 Hz), 118.0 (d, $^3J_{F-C}$ = 8.1 Hz), 116.9 (d, $^2J_{F-C}$ = 22.6 Hz), 114.7 (d, $^2J_{F-C}$ = 22.7 Hz), 61.4, 61.3, 47.4, 28.2, 14.1. ^{19}F NMR (376 MHz, $CDCl_3$) δ -124.01. HPLC: Chiralpak IB column, 230 nm, 30 °C, *n*-Hexane/*i*-Pr OH = 90/10, flow = 0.8 mL/min, retention time 10.2 min and 11.5 min (major). HRMS Calculated for $C_{12}H_{16}FO_4$ $[M+H]^+$ 243.1027, found: 243.1027.

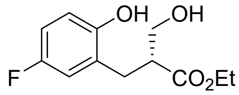

(+)-Ethyl 2-(4-chloro-2-hydroxybenzyl)-3-hydroxypropanoate (2k):

50 mg, 97% yield, colorless oil, new compound, R_f = 0.40 (dichloromethane/methanol 40/1), 81% ee, $[\alpha]^{20}_D$ = +9.97 (*c* 1.00, $CHCl_3$). ^1H NMR (400 MHz, $CDCl_3$) δ 7.85 (brs, 1H), 7.01 (d, *J* = 8.1 Hz, 1H), 6.91-6.72 (m, 2H), 4.19 (q, *J* = 7.1 Hz, 2H), 3.89 (dd, *J* = 11.5, 3.7 Hz, 1H), 3.63 (dd, *J* = 11.4, 4.4 Hz, 1H), 3.33 (brs, 1H), 3.04-2.89 (m, 2H), 2.84-2.73 (m, 1H), 1.27 (t, *J* = 7.1 Hz, 3H). ^{13}C NMR (100 MHz, $CDCl_3$) δ 175.1, 155.7, 133.2, 131.8, 123.2, 120.8, 117.1, 61.44, 61.41, 47.5, 27.8, 14.2. HPLC: Chiralpak AS-H column, 230 nm, 30 °C, *n*-Hexane/*i*-PrOH = 90/10, flow = 0.8 mL/min, retention time 14.4 min and 16.2 min (major). HRMS Calculated for $C_{12}H_{16}ClO_4$ $[M+H]^+$ Cl^{35} 259.0732, found: 259.0746. $[M+H]^+$ Cl^{37} 261.0706, found: 261.0716.

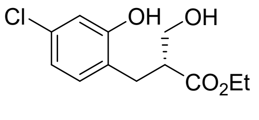

(+)-Methyl 3-(3-ethoxy-2-(hydroxymethyl)-3-oxopropyl)-4-hydroxybenzoate (2l):

56 mg, 99% yield, colorless oil, new compound, R_f = 0.35 (dichloromethane/methanol 40/1), 76% ee, $[\alpha]^{20}_D$ = +31.16 (*c* 1.12, $CHCl_3$). ^1H NMR (400 MHz, $CDCl_3$) δ 7.91-7.77 (m, 2H), 6.90 (d, *J* = 8.4 Hz, 1H), 4.26-4.14 (m, 2H), 3.92 (dd, *J* = 11.6, 3.8 Hz, 1H), 3.88 (s, 3H), 3.63 (dd, *J* = 11.6, 4.6 Hz, 1H), 3.07-2.99 (m, 2H), 2.87-2.81 (m, 1H), 1.29 (t, *J* = 7.1 Hz, 3H). ^{13}C NMR (100 MHz, $CDCl_3$) δ 175.1, 167.1, 159.7, 133.2, 130.4, 124.5, 122.4, 116.8, 61.4, 61.4, 52.0, 47.4, 28.1, 14.1. HPLC: Chiralpak AS-H column, 254 nm, 30 °C, *n*-Hexane/*i*-PrOH = 90/10, flow = 0.8 mL/ min, retention time 22.5 min and 26.0 min (major). The HRMS Calculated for $C_{14}H_{18}O_6K$ $[M+K]^+$ 321.0735, found: 321.0728.

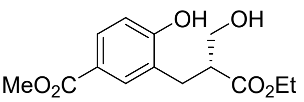

(+)-Ethyl 3-hydroxy-2-(2-hydroxy-5-methoxybenzyl)propanoate (2m):

30 mg, 59% yield, colorless oil, new compound, R_f = 0.40 (dichloromethane/methanol 40/1), 89% ee, $[\alpha]^{20}_D$ = +4.83 (*c* 0.60, $CHCl_3$). ^1H NMR (400 MHz, $CDCl_3$) δ 7.05 (brs, 1H), 6.81 (d, *J* = 8.6 Hz, 1H), 6.78-6.54 (m, 2H), 4.20 (q, *J* = 7.1 Hz, 2H), 3.91 (dd, *J* = 11.4, 3.2 Hz, 1H), 3.74 (s, 3H), 3.58 (d, *J* = 10.9 Hz, 1H), 3.16 (brs, 1H), 3.08-2.91 (m, 2H), 2.85-2.72 (m, 1H), 1.28 (t, *J* = 7.1 Hz, 3H). ^{13}C NMR (100 MHz, $CDCl_3$) δ 175.2, 153.7, 148.9, 125.6, 117.8, 116.0, 113.5, 61.33, 61.29, 55.7, 47.6, 28.3, 14.2. HPLC: Chiralpak AS-H column, 230 nm, 30 °C, *n*-Hexane/*i*-PrOH = 90/10, flow = 0.8 mL/ min, retention time 19.5 min and 21.1 min (major). The HRMS Calculated for $C_{13}H_{19}O_5$ $[M+H]^+$ 255.1227, found: 255.1237.

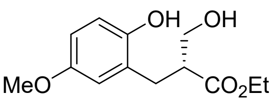

(+)-Ethyl 3-hydroxy-2-(2-hydroxy-4-methoxybenzyl)propanoate (2n):

46 mg, 91% yield, colorless oil, new compound, R_f = 0.40 (dichloromethane/methanol 40/1), 88% ee, $[\alpha]^{20}_D$ = +12.17 (*c* 0.92, $CHCl_3$). ^1H NMR (400 MHz, $CDCl_3$) δ 7.64 (brs, 1H), 6.98 (d, *J* = 8.2 Hz, 1H), 6.57-6.25 (m, 2H), 4.19 (q, *J* = 7.1 Hz, 2H), 3.88 (dd, *J* = 11.5, 3.8 Hz, 1H), 3.74 (s, 3H), 3.62 (dd, *J* = 11.5, 4.5 Hz, 1H), 3.33 (brs, 1H), 3.02-2.86 (m, 2H), 2.82-2.68 (m, 1H), 1.27 (t, *J* = 7.1 Hz, 3H). ^{13}C NMR (100 MHz, $CDCl_3$) δ 175.3, 159.8, 155.9, 131.5, 116.7, 106.6, 102.5, 61.4, 61.2, 55.3, 47.8, 27.6, 14.2. HPLC: Chiralpak IB column, 230 nm, 30 °C, *n*-Hexane/*i*-PrOH = 95/5, flow = 0.8 mL/min, retention time 32.7 min (major) and 35.2 min. The HRMS Calculated for $C_{13}H_{19}O_5$

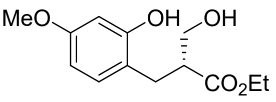

[M+H]$^+$ 255.1227, found: 255.1234.

(+)-Ethyl (*R*)-3-hydroxy-2-((2-hydroxynaphthalen-1-yl)methyl)propanoate (2o):

51 mg, 93% yield, white solid, new compound, mp 82-83 °C, R_f = 0.60 (dichloromethane/methanol 40/1), 87% ee, $[\alpha]^{20}_D$ = +10.11 (*c* 1.00, $CHCl_3$). ^{1}H NMR (400 MHz, $CDCl_3$) δ 8.08 (brs, 1H), 7.90 (d, *J* = 8.5 Hz, 1H), 7.79 (d, *J* = 8.1 Hz, 1H), 7.69 (d, *J* = 8.8 Hz, 1H), 7.52 (t, *J* = 7.7 Hz, 1H), 7.42-7.30 (m, 1H), 7.21 (d, *J* = 8.8 Hz, 1H), 4.28 (q, *J* = 7.1 Hz, 2H), 4.09-3.95 (m, 1H), 3.72-3.27 (m, 4H), 3.00-2.93 (m, 1H), 1.34 (t, *J* = 7.1 Hz, 3H). ^{13}C NMR (100 MHz, $CDCl_3$) δ 175.8, 153.2, 133.2, 129.5, 128.92, 128.85, 126.7, 123.2, 122.2, 119.6, 115.6, 61.5, 61.4, 45.9, 23.6, 14.2. HPLC: Chiralpak AS-H column, 230 nm, 30 °C, *n*-Hexane/*i*-PrOH = 90/10, flow = 0.8 mL/ min, retention time 13.1 min (minor) and 15.6 min (major). HRMS Calculated for $C_{16}H_{19}O_4$ [M+H]$^+$ 275.1278, found: 275.1285.

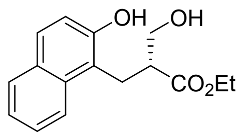

(+)-Ethyl 3-hydroxy-2-((3-hydroxynaphthalen-2-yl)methyl)propanoate (2p):

38 mg, 70% yield, colorless oil, new compound, R_f = 0.40 (dichloromethane/methanol 40/1), 69% ee, $[\alpha]^{20}_D$ = +12.55 (*c* 0.76, $CHCl_3$). ^{1}H NMR (400 MHz, $CDCl_3$) δ 7.82-7.52 (m, 3H), 7.41-7.35 (m, 1H), 7.33-7.28 (m, 1H), 7.23 (s, 1H), 4.21 (q, *J* = 7.1 Hz, 2H), 3.93 (dd, *J* = 11.5, 3.6 Hz, 1H), 3.66 (dd, *J* = 11.5, 4.7 Hz, 1H), 3.24-3.14 (m, 2H), 2.97-2.90 (m, 1H), 1.28 (t, *J* = 7.1 Hz, 3H). ^{13}C NMR (100 MHz, $CDCl_3$) δ 175.2, 153.2, 134.0, 130.1, 129.0, 127.3, 127.2, 126.1, 126.0, 123.7, 111.3, 61.6, 61.3, 47.9, 28.7, 14.2. HPLC: Chiralpak IB column, 230 nm, 30 °C, *n*-Hexane/*i*-PrOH = 90/10, flow = 0.8 mL/min, retention time 14.9 min (major) and 17.8 min. The HRMS Calculated for $C_{16}H_{19}O_4$ [M+H]$^+$ 275.1278, found: 275.1279.

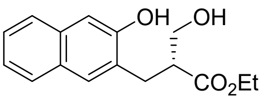

4.2 Pd-catalyzed Hydrogenative Kinetic Resolution of Lactones with Qutarnary Centers

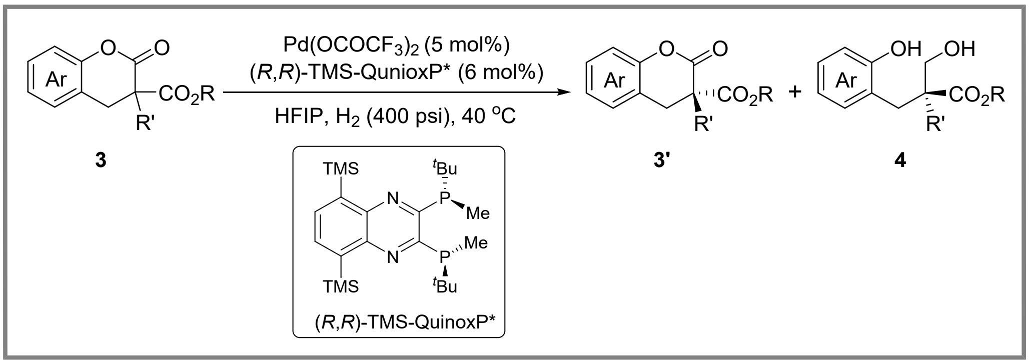

General Procedure: The $Pd(OCOCF_3)_2$ (0.015 mmol, 4.8 mg, 5 mol%), ligand (*R,R*)-TMS-QuinoxP* (0.018 mmol, 8.6 mg, 6 mol%) and degassed anhydrous acetone (1.0 mL) were placed in a dried Schlenk tube under nitrogen atmosphere. The mixture was stirred under room temperature for 1 hour, the solvent was removed under vacuum to give the catalyst. Then, this catalyst was taken into a glove box filled with nitrogen gas and dissolved in 1,1,1,3,3,3-hexafluoro-2-propanol (HFIP, 1.0 mL). To the lactones **3** (0.3 mmol) added this catalyst solution, and then the mixture was transferred to an autoclave, which was charged hydrogen gas (400 psi). The reaction mixture was stirred at 40 °C for 55-120 hours. After cooling to room temperature and the release of the hydrogen gas, the autoclave was opened. To the mixture added sodium cyanoborohydride (7.5 mg, 0.12 mmol). The mixture was stirred under room temperature for 2 hours, then the volatiles were removed under the reduced pressure. The crude residue was purified by column chromatography on silica gel using hexanes/ethyl acetate (10:1-5:1) as eluent to give the chiral reductive products **3'** and **4**.

The racemates were prepared by reduction of lactones **3** with sodium borohydride in methanol.

(+)-Ethyl 2-benzyl-3-hydroxy-2-(2-hydroxybenzyl)propanoate (4a):

React for 55 h. 48 mg, 52% yield, colorless oil, new compound, R_f = 0.40 (hexanes/ethyl acetate

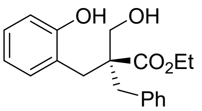

5/1), 70.8% ee, $[\alpha]^{20}_D$ = +4.90 (*c* 0.96, $CHCl_3$). ^{1}H NMR (400 MHz, $CDCl_3$) δ 7.26-7.02 (m, 7H), 7.01-6.80 (m, 2H), 4.27-4.06 (m, 2H), 3.71 (d, *J* = 11.7 Hz, 1H), 3.39 (d, *J* = 11.7 Hz, 1H), 3.26-3.17 (m, 2H), 3.01 (d, *J* = 14.1 Hz, 1H), 2.86 (d, *J* = 13.5 Hz, 1H), 1.27 (t, *J* = 7.2 Hz, 3H). ^{13}C NMR (100 MHz, $CDCl_3$) δ 175.2, 155.6, 136.6, 132.5, 129.8, 128.7, 128.4, 126.9, 122.5, 120.5, 117.2, 61.7, 61.1, 53.6, 40.7, 34.7, 14.1. HPLC: Chiral-pak IB column, 230 nm, 30 °C, *n*-Hexane/*i*-PrOH = 95/5, flow = 0.8 mL/min, retention time 9.6 min and 10.3 min (major). HRMS Calculated for $C_{19}H_{22}O_4Na$ $[M+H]^+$ 337.1410, found: 337.1416.

(+)-Ethyl (*S*)-3-benzyl-2-oxochromane-3-carboxylate (3a'):

Kinetic resolution from hydrogenation of **3a**. 44 mg, 47% yield, 93.0% ee, $[\alpha]^{20}_D$ = +34.18 (*c* 0.88, $CHCl_3$). HPLC: Chiralpak AS-H column, 230 nm, 30 °C, *n*-Hexane/*i*-PrOH = 90/10, flow = 0.8 mL/min, retention time 9.5 min (major) and 10.7 min.

(+)-Ethyl 3-hydroxy-2-(2-hydroxybenzyl)-2-(4-methylbenzyl)propanoate (4b):

React for 55 h. 52 mg, 54% yield, colorless oil, new compound, R_f = 0.37 (hexanes/ethyl acetate

5/1), 61.6% ee, $[\alpha]^{20}_{D}$ = +7.31 (*c* 1.04, $CHCl_3$). ^{1}H NMR (400 MHz, $CDCl_3$) δ 8.20 (brs, 1H),

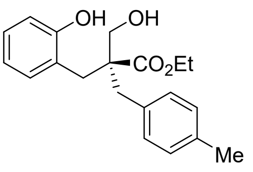

7.19-7.11 (m, 2H), 7.05 (d, *J* = 7.8 Hz, 2H), 6.97 (d, *J* = 7.9 Hz, 2H), 6.93-6.82 (m, 2H), 4.25-4.09 (m, 2H), 3.69 (d, *J* = 11.3 Hz, 1H), 3.40 (d, *J* = 11.7 Hz, 2H), 3.25-3.14 (m, 2H), 3.02 (d, *J* = 14.1 Hz, 1H), 2.85 (d, *J* = 13.5 Hz, 1H), 2.30 (s, 3H), 1.28 (t, *J* = 7.2 Hz, 3H). ^{13}C NMR (100 MHz, $CDCl_3$) δ 175.4, 155.7, 136.4, 133.4, 132.5, 129.6, 129.1, 128.7, 122.5, 120.4, 117.3, 61.9, 61.1, 53.5, 40.2, 34.7, 21.1, 14.2. HPLC: Chiralpak IB column, 230 nm, 30 °C, *n*-Hexane/*i*-PrOH = 95/5, flow = 0.8 mL/min, retention time 9.1 min and 10.3 min (major). HRMS Calculated for $C_{20}H_{25}O_4$ $[M+H]^+$ 329.1747, found: 329.1755.

(+)-Ethyl 3-(4-methylbenzyl)-2-oxochromane-3-carboxylate (3b'):

Kinetic resolution from hydrogenation of **3b**. 43 mg, 44% yield, 84.1% ee, $[\alpha]^{20}_{D}$ = +35.11 (*c* 0.86, $CHCl_3$). HPLC: Chiralpak AS-H column, 230 nm, 30 °C, *n*-Hexane/*i*-PrOH = 90/10, flow = 0.8 mL/min, retention time 8.0 min (major) and 9.1 min.

(+)-Ethyl 2-(4-fluorobenzyl)-3-hydroxy-2-(2-hydroxybenzyl)propanoate (4c):

React for 72 h. 61 mg, 61% yield, colorless oil, new compound, R_f = 0.30 (hexanes/ethyl acetate

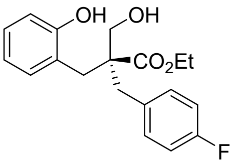

5/1), 60.1% ee, $[\alpha]^{20}_{D}$ = +5.66 (*c* 1.22, $CHCl_3$). ^{1}H NMR (400 MHz, $CDCl_3$) δ 8.41 (brs, 1H), 7.19-7.05 (m, 4H), 6.97-6.77 (m, 4H), 4.27-3.82 (m, 3H), 3.63 (d, *J* = 11.8 Hz, 1H), 3.44 (d, *J* = 11.7 Hz, 1H), 3.21-2.91 (m, 4H), 1.23 (t, *J* = 7.2 Hz, 3H). ^{13}C NMR (100 MHz, $CDCl_3$) δ 174.8, 161.8 (d, $^{1}J_{F\text{-}C}$ = 245.0 Hz), 155.2, 132.4 (d, $^{4}J_{F\text{-}C}$ = 3.0 Hz), 132.3, 131.3 (d, $^{3}J_{F\text{-}C}$ = 7.8 Hz), 128.7, 122.8 , 120.7, 116.7, 115.2 (d, $^{2}J_{F\text{-}C}$ = 21.1 Hz), 61.0, 60.9, 53.8, 39.9, 34.6, 14.1. ^{19}F NMR (376 MHz, $CDCl_3$) δ -115.88. HPLC: Chiralpak IB column, 230 nm, 30 °C, *n*-Hexane/*i*-PrOH = 95/5, flow = 0.8 mL/min, retention time 10.2 min and 10.7 min (major). HRMS Calculated for $C_{19}H_{22}FO_4$ $[M+H]^+$ 333.1497, found: 333.1498.

(+)-Ethyl 3-(4-fluorobenzyl)-2-oxochromane-3-carboxylate (3c'):

Kinetic resolution from hydrogenation of **3c**. 37 mg, 38% yield, 98.3% ee, $[\alpha]^{20}_{D}$ = +33.17 (*c* 0.74, $CHCl_3$). HPLC: Chiralpak AS-H column, 230 nm, 30 °C, *n*-Hexane/*i*-PrOH = 90/10, flow = 0.8 mL/min, retention time 9.3 min (major) and 11.2 min.

(+)-Ethyl 3-hydroxy-2-(2-hydroxybenzyl)-2-(naphthalen-2-ylmethyl)propanoate (4d):

React for 120 h. 49 mg, 45% yield, colorless oil, new compound, R_f = 0.30 (hexanes/ethyl acetate 5/1), 67.1% ee, $[\alpha]^{20}_{D}$ = +3.74 (*c* 0.98, $CHCl_3$). ^{1}H NMR (400 MHz, $CDCl_3$) δ 8.33 (brs, 1H),

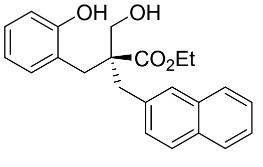

7.79-7.66 (m, 3H), 7.56 (s, 1H), 7.46-7.37 (m, 2H), 7.22 (d, *J* = 7.7 Hz, 1H), 7.17-7.08 (m, 2H), 6.95-6.80 (m, 2H), 4.14 (q, *J* = 7.1 Hz, 2H), 3.68 (d, *J* = 11.7 Hz, 2H), 3.48 (d, *J* = 11.7 Hz, 1H), 3.34 (d, *J* = 13.4 Hz, 1H), 3.24 (d, *J* = 14.1 Hz, 1H), 3.16-3.02 (m, 2H), 1.21 (t, *J* = 7.1 Hz, 3H). ^{13}C NMR (100 MHz, $CDCl_3$) δ 175.2, 155.5, 134.3, 133.4, 132.5, 132.4, 128.7, 128.6, 128.1, 127.9, 127.62, 127.59, 126.1, 125.7, 122.7, 120.6, 117.0, 61.5, 61.1, 53.9, 40.8, 34.8, 14.2. HPLC: Chiralpak IA column, 230 nm, 30 °C, *n*-Hexane/*i*-PrOH = 95/5, flow = 0.8 mL/min, retention time 18.7 min (minor) and 26.5 min (major). HRMS Calculated for $C_{23}H_{25}O_4$ $[M+H]^+$ 365.1747, found: 365.1750.

(+)-Ethyl 3-(naphthalen-2-ylmethyl)-2-oxochromane-3-carboxylate (3d'):

Kinetic resolution from hydrogenation of **3d**. 60 mg, 55% yield, 59.7% ee, $[\alpha]^{20}_{D}$ = +14.32 (*c* 1.20, $CHCl_3$). HPLC: Chiralpak AS-H column, 230 nm, 30 °C, *n*-Hexane/*i*-PrOH = 90/10, flow = 0.8 mL/min, retention time 11.4 min (major) and 13.5 min.

(+)-Ethyl 3-hydroxy-2-(2-hydroxybenzyl)-2-methylpropanoate (4e):

React for 72 h. 45 mg, 64% yield, colorless oil, new compound, R_f = 0.34 (hexanes/ethyl acetate 5/1), 53.8% ee, $[\alpha]^{20}_{D}$ = +6.88 (*c* 0.90, $CHCl_3$). ^{1}H NMR (400 MHz, $CDCl_3$) δ 8.20 (brs, 1H), 7.23-7.09 (m, 1H), 7.05 (d, *J* = 6.5 Hz, 1H), 6.93-6.67 (m, 2H), 4.22 (q, *J* = 7.1 Hz, 2H), 3.78 (d, *J* = 11.7 Hz, 1H), 3.31-3.08 (m, 2H), 2.79 (d, *J* = 14.1 Hz, 1H), 1.30 (t, *J* = 7.1 Hz, 3H), 1.14 (s, 3H). ^{13}C NMR (100 MHz, $CDCl_3$) δ 177.6, 155.8, 132.9, 128.6, 122.2, 120.2, 117.2, 65.6, 61.3, 48.0, 34.0, 19.3, 14.2. HPLC: Chiralpak IB column, 230 nm, 30 °C, *n*-Hexane/*i*-PrOH = 95/5, flow = 0.8 mL/min, retention time 12.3 min and 13.9 min (major). HRMS Calculated for $C_{13}H_{19}O_4$ $[M+H]^+$ 239.1278, found: 239.1271.

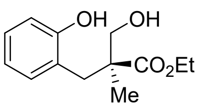

(+)-Ethyl 3-methyl-2-oxochromane-3-carboxylate (3e'):

Kinetic resolution from hydrogenation of **3e**. 25 mg, 35% yield, 98.4% ee, $[\alpha]^{20}_{D}$ = +11.34 (*c* 0.50, $CHCl_3$). HPLC: Chiralpak AS-H column, 230 nm, 30 °C, *n*-Hexane/*i*-PrOH = 90/10, flow = 0.8 mL/min, retention time 9.8 min (major) and 11.0 min.

(+)-Ethyl 2-(2-hydroxy-5-methylbenzyl)-2-(hydroxymethyl)butanoate (4f):

React for 60 h. 36 mg, 49% yield, colorless oil, new compound, R_f = 0.38 (hexanes/ethyl acetate 5/1), 80.5% ee, $[\alpha]^{20}_{D}$ = +7.71 (*c* 0.72, $CHCl_3$). ^{1}H NMR (400 MHz, $CDCl_3$) δ 8.33 (brs, 1H), 7.18-7.10 (m, 1H), 7.08-7.00 (m, 1H), 6.93-6.79 (m, 2H), 4.23 (q, *J* = 7.1 Hz, 2H), 3.86 (d, *J* = 11.6 Hz, 2H), 3.32 (d, *J* = 11.6 Hz, 1H), 3.18 (d, *J* = 14.2 Hz, 1H), 2.85 (d, *J* = 14.2 Hz, 1H), 1.80-1.67 (m, 1H), 1.60-1.49 (m, 1H), 1.30 (t, *J* = 7.1 Hz, 3H), 0.87 (t, *J* = 7.5 Hz, 3H). ^{13}C NMR (100 MHz, $CDCl_3$) δ 176.3, 155.6, 132.5, 128.5, 122.6, 120.3, 117.0, 63.1, 61.1, 52.7, 33.0, 26.6, 14.3, 8.9. The HPLC: Chiralpak IB column, 230 nm, 30 °C, *n*-Hexane/*i*-PrOH = 90/10, flow = 0.8 mL/min, retention time 9.8 min and 11.2 min (major). HRMS Calculated for $C_{14}H_{20}O_4Na$ $[M+Na]^+$ 275.1254, found: 275.1253.

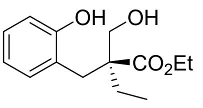

(+)-Ethyl 3-ethyl-6-methyl-2-oxochromane-3-carboxylate (3f'):

Kinetic resolution from hydrogenation of substrate lactone **3f**. 35 mg, 47% yield, 88.6% ee, $[\alpha]^{20}_{D}$ = +14.91 (*c* 0.70, $CHCl_3$). The HPLC: Chiralpak AS-H column, 230 nm, 30 °C, *n*-Hexane/*i*-PrOH = 90/10, flow = 0.8 mL/min, retention time 7.9 min (major) and 9.7 min (minor).

(+)-Ethyl 2-(2-hydroxybenzyl)-2-(hydroxymethyl)pentanoate (4g):

React for 72 h. 47 mg, 57% yield, colorless oil, new compound, R_f = 0.40 (hexanes/ethyl acetate 5/1), 75.0% ee, $[\alpha]^{20}_{D}$ = +10.32 (*c* 0.94, $CHCl_3$). ^{1}H NMR (400 MHz, $CDCl_3$) δ 8.30 (brs, 1H), 7.19-7.09 (m, 1H), 7.08-6.99 (m, 1H), 6.94-6.74 (m, 2H), 4.31-4.15 (m, 2H), 3.82 (t, *J* = 29.3 Hz, 2H), 3.30 (d, *J* = 11.7 Hz, 1H), 3.18 (d, *J* = 14.2 Hz, 1H), 2.84 (d, *J* = 14.2 Hz, 1H), 1.74-1.64 (m, 1H),

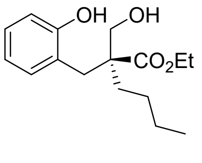

1.53-1.43 (m, 1H), 1.33-1.18 (m, 7H), 0.89 (t, J = 7.2 Hz, 3H). ^{13}C NMR (100 MHz, $CDCl_3$) δ 176.5, 155.7, 132.6, 128.5, 122.6, 120.2, 117.1, 63.5, 61.0, 52.2, 33.5, 33.3, 26.7, 23.2, 14.3, 13.9. HPLC: Chi- ralpak IB column, 230 nm, 30 °C, *n*-Hexane/*i*-PrOH = 95/5, flow = 0.8 mL/min, retention time 8.9 min and 9.4 min (major). HRMS Calculated for $C_{16}H_{24}O_4Na$ $[M+Na]^+$ 303.1567, found: 303.1572.

(+)-Ethyl 2-oxo-3-propylchromane-3-carboxylate (3g'):

Kinetic resolution from hydrogenation of **3g**. 36 mg, 43% yield, 99.0% ee, $[\alpha]^{20}_D$ = +15.97 (*c* 0.72, $CHCl_3$). HPLC: Chiralpak AS-H column, 230 nm, 30 °C, *n*-Hexane/*i*-PrOH = 90/10, flow = 0.8 mL/min, retention time 7.1 min (major) and 8.6 min.

(+)-Ethyl 2-(2-hydroxybenzyl)-2-(hydroxymethyl)-4-methoxybutanoate (4h):

React for 66 h. 39 mg, 46% yield, colorless oil, new compound, R_f = 0.20 (hexanes/ethyl acetate 5/1), 84.4% ee, $[\alpha]^{20}_D$ = +20.99 (*c* 0.78, $CHCl_3$). ^{1}H NMR (400 MHz, $CDCl_3$) δ 7.16-7.09 (m, 1H), 7.05-6.97 (m, 1H), 6.89 (d, J = 7.9 Hz, 1H), 6.84-6.74 (m, 1H), 4.16 (q, J = 7.1 Hz, 2H), 3.77 (d, J = 12.1 Hz, 1H), 3.59-3.37 (m, 3H), 3.33 (s, 3H), 2.99 (dd, J = 35.6, 14.1 Hz, 2H), 2.19-2.07 (m, 1H), 1.94-1.85 (m, 1H), 1.25 (t, J = 7.1 Hz, 3H). ^{13}C NMR (100 MHz, $CDCl_3$) δ 175.6, 155.9, 132.2, 128.6, 122.5, 120.2, 117.3, 69.0, 62.8, 61.1, 58.8, 51.4, 34.6, 33.9, 14.1. HPLC: Chiralpak IB column, 230 nm, 30 °C, *n*-Hexane/*i*-PrOH = 95/5, flow = 0.8 mL/min, retention time 14.1 min and 17.5 min (major). HRMS Calculated for $C_{15}H_{22}O_5Na$ $[M+Na]^+$ 303.1359, found: 303.1364.

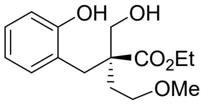

(+)-Ethyl 3-(2-methoxyethyl)-2-oxochromane-3-carboxylate (3h'):

Kinetic resolution from hydrogenation of **3h**. 44 mg, 53% yield, 66.4% ee, $[\alpha]^{20}_D$ = +17.33 (*c* 0.88, $CHCl_3$). HPLC: Chiralpak AS-H column, 230 nm, 30 °C, *n*-Hexane/*i*-PrOH = 90/10, flow = 0.8 mL/min, retention time 9.2 min (major) and 10.1 min.

(+)-Ethyl 3-cyclopropyl-2-(2-hydroxybenzyl)-2-(hydroxymethyl)propanoate (4i):

React for 72 h. 56 mg, 68% yield, colorless oil, new compound, R_f = 0.30 (hexanes/ethyl acetate 5/1), 39.8% ee, $[\alpha]^{20}_D$ = +9.10 (*c* 1.12, $CHCl_3$). ^{1}H NMR (400 MHz, $CDCl_3$) δ 8.30 (brs, 1H), 7.20-7.11 (m, 1H), 7.07-6.95 (m, 1H), 6.95-6.74 (m, 2H), 4.33-4.16 (m, 2H), 4.09 (d, J = 11.7 Hz, 1H), 3.39 (d, J = 11.7 Hz, 2H), 3.18 (d, J = 14.2 Hz, 1H), 2.83 (d, J = 14.2 Hz, 1H), 1.90-1.80 (m, 1H), 1.33 (t, J = 7.1 Hz, 3H), 0.65-0.51 (m, 1H), 0.49-0.36 (m, 2H), 0.08-0.03 (m, 2H). ^{13}C NMR (100 MHz, $CDCl_3$) δ 176.7, 155.9, 132.6, 128.6, 122.3, 120.2, 117.4, 63.4, 61.1, 52.8, 39.0, 34.0, 14.2, 6.5, 5.3, 4.1. HPLC: Chiralpak IB column, 230 nm, 30 °C, *n*-Hexane/*i*-PrOH = 95/5, flow = 0.8 mL/min, retention time 9.9 min and 11.4 min (major). HRMS Calculated for $C_{16}H_{23}O_4$ $[M+H]^+$ 279.1591, found: 279.1597.

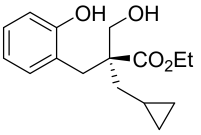

(+)-Ethyl 3-(cyclopropylmethyl)-2-oxochromane-3-carboxylate (3i'):

Kinetic resolution from hydrogenation of **3i**. 26 mg, 32% yield, 98.8% ee, $[\alpha]^{20}_D$ = +32.66 (*c* 0.52, $CHCl_3$). HPLC: Chiralpak AS-H column, 230 nm, 30 °C, *n*-Hexane/*i*-PrOH = 90/10, flow = 0.8 mL/min, retention time 7.6 min (major) and 9.6 min.

(+)-Ethyl 3-cyclobutyl-2-(2-hydroxybenzyl)-2-(hydroxymethyl)propanoate (4j):

React for 55 h under 50 °C. 58 mg, 67% yield, colorless oil, new compound, R_f = 0.25 (hexanes/ethyl acetate 5/1), 48.0% ee, $[\alpha]^{20}_D$ = +2.33 (*c* 1.16, $CHCl_3$). ^1H NMR (400 MHz, $CDCl_3$) δ 8.44 (brs, 1H), 7.17-7.10 (m, 1H), 7.08-6.98 (m, 1H), 6.91-6.73 (m, 2H), 4.24-4.12 (m, 2H), 3.82 (d, *J* = 11.7 Hz, 2H), 3.29 (d, *J* = 11.7 Hz, 1H), 3.13 (d, *J* = 14.1 Hz, 1H), 2.83 (d, *J* = 14.2 Hz, 1H), 2.37-2.26 (m, 1H), 2.02-1.92 (m, 2H), 1.89-1.76 (m, 2H), 1.74-1.54 (m, 4H), 1.30 (t, *J* = 7.1 Hz, 3H). ^{13}C NMR (100 MHz, $CDCl_3$) δ 176.4, 155.7, 132.6, 128.5, 122.6, 120.3, 117.0, 63.1, 61.0, 51.9, 41.2, 33.7, 32.5, 30.1, 29.2, 18.8, 14.2. HPLC: Chiralpak IB column, 230 nm, 30 °C, *n*-Hexane/*i*-PrOH = 95/5, flow = 0.8 mL/min, retention time 9.1 min and 9.9 min (major). HRMS Calculated for $C_{17}H_{24}O_4Na$ $[M+Na]^+$ 315.1567, found: 315.1567.

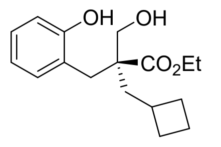

(+)-Ethyl 3-(cyclobutylmethyl)-2-oxochromane-3-carboxylate (3j'):

Kinetic resolution from hydrogenation of **3j**. 28 mg, 33% yield, 95.6% ee, $[\alpha]^{20}_D$ = +24.69 (*c* 0.56, $CHCl_3$). HPLC: Chiralpak AS-H column, 230 nm, 30 °C, *n*-Hexane/*i*-PrOH = 90/10, flow = 0.8 mL/min, retention time 7.6 min (major) and 8.9 min.

(+)-Ethyl 3-cyclohexyl-2-(2-hydroxybenzyl)-2-(hydroxymethyl)propanoate (4k):

React for 72 h. 62 mg, 64% yield, colorless oil, new compound, R_f = 0.30 (hexanes/ethyl acetate 5/1), 55.0% ee, $[\alpha]^{20}_D$ = +6.51 (*c* 1.04, $CHCl_3$). ^1H NMR (400 MHz, $CDCl_3$) δ 8.27 (brs, 1H), 7.20-7.10 (m, 1H), 7.07-7.00 (m, 1H), 6.98-6.87 (m, 1H), 6.86-6.76 (m, 1H), 4.22 (q, *J* = 7.1 Hz, 2H), 3.96 (d, *J* = 11.7 Hz, 1H), 3.44 (brs, 1H), 3.26 (d, *J* = 11.9 Hz, 1H), 3.16 (d, *J* = 14.1 Hz, 1H), 2.75 (d, *J* = 14.1 Hz, 1H), 1.79-1.70 (m, 1H), 1.67-1.59 (m, 4H), 1.52 (d, *J* = 13.2 Hz, 1H), 1.47-1.40 (m, 1H), 1.32 (t, *J* = 7.1 Hz, 3H), 1.25-1.08 (m, 4H), 0.98-0.85 (m, 2H). ^{13}C NMR (100 MHz, $CDCl_3$) δ 177.2, 155.8, 132.7, 128.5, 122.5, 120.2, 117.2, 63.2, 61.0, 51.4, 41.3, 34.9, 34.7, 34.1, 33.7, 26.4, 26.3, 26.1, 14.1. HPLC: Chiralpak AS-H column, 230 nm, 30 °C, *n*-Hexane/*i*-PrOH = 90/10, flow = 0.8 mL/min, retention time 6.2 min (major) and 7.2 min. HRMS Calculated for $C_{19}H_{28}O_4Na$ $[M+Na]^+$ 339.1567, found: 339.1534.

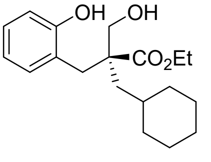

(+)-Ethyl 3-(cyclohexylmethyl)-2-oxochromane-3-carboxylate (3k'):

Kinetic resolution from hydrogenation of **3k**. 34 mg, 36% yield, 97.0% ee, $[\alpha]^{20}_D$ = +14.87 (*c* 0.68, $CHCl_3$). HPLC: Chiralcel OD-H column, 230 nm, 30 °C, *n*-Hexane/*i*-PrOH = 90/10, flow = 0.8 mL/min, retention time 5.6 min (major) and 6.2 min.

(+)-Methyl 2-benzyl-3-hydroxy-2-(2-hydroxybenzyl)propanoate (4l):

React for 50 h. 46 mg, 51% yield, colorless oil, new compound, R_f = 0.35 (hexanes/ethyl acetate 5/1), 77.4% ee, $[\alpha]^{20}_D$ = +13.75 (*c* 0.64, $CHCl_3$). ^1H NMR (400 MHz, $CDCl_3$) δ 8.08 (brs, 1H), 7.27-7.04 (m, 7H), 6.93-6.81 (m, 2H), 3.69 (s, 3H), 3.68-3.28 (m, 3H), 3.27-3.14 (m, 2H), 3.04 (d, *J* = 14.1 Hz, 1H), 2.90 (d, *J* = 13.5 Hz, 1H). ^{13}C NMR (100 MHz, $CDCl_3$) δ 175.6, 155.6, 136.6, 132.4, 129.7, 128.7, 128.5, 126.9, 122.4, 120.5, 117.2, 61.6, 53.8, 51.8, 40.7, 34.7. HPLC: Chiralpak IB column, 230 nm, 30 °C, *n*-

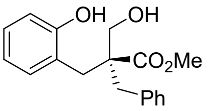

Hexane/*i*-PrOH = 97/3, flow = 1.0 mL/ min, retention time 13.8 min and 15.0 min (major). HRMS Calculated for $C_{18}H_{20}O_4Na$ $[M+Na]^+$ 323.1254, found: 323.1268.

(+)-Methyl 3-benzyl-2-oxochromane-3-carboxylate (3l'):

Kinetic resolution from hydrogenation of **3l**. 45 mg, 48% yield, 72.0% ee, $[\alpha]^{20}_D$ = +32.00 (*c* 0.60, $CHCl_3$). HPLC: Chiralpak AS-H column, 230 nm, 30 °C, *n*-Hexane/*i*-PrOH = 90/10, flow = 0.8 mL/min, retention time 12.0 min and 23.5 min (major).

(+)-Isopropyl 2-benzyl-3-hydroxy-2-(2-hydroxybenzyl)propanoate (4m):

React for 72 h. 52 mg, 53% yield, colorless oil, new compound, R_f = 0.35 (hexanes/ethyl acetate

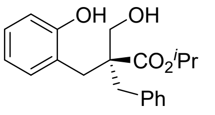

5/1), 71.2% ee, $[\alpha]^{20}_D$ = +7.61 (*c* 1.04, $CHCl_3$). ^{1}H NMR (400 MHz, $CDCl_3$) δ 8.41 (brs, 1H), 7.26-7.07 (m, 7H), 6.94-6.80 (m, 2H), 5.09-4.89 (m, 1H), 3.70 (t, *J* = 36.4 Hz, 2H), 3.41 (d, *J* = 11.7 Hz, 1H), 3.25-3.11 (m, 2H), 3.03 (d, *J* = 14.1 Hz, 1H), 2.91 (d, *J* = 13.5 Hz, 1H), 1.24-1.05 (m, 6H). ^{13}C NMR (100 MHz, $CDCl_3$) δ 174.8, 155.5, 136.7, 132.6, 130.0, 128.7, 128.3, 126.8, 122.7, 120.4, 117.1, 69.0, 61.5, 53.5, 40.6, 34.8, 21.81, 21.75. HPLC: Chiralpak IB column, 230 nm, 30 °C, *n*-Hexane/*i*-PrOH = 95/5, flow = 0.8 mL/ min, retention time 8.6 min and 9.6 min (major). HRMS Calculated for $C_{20}H_{24}O_4Na$ $[M+Na]^+$ 351.1567, found: 351.1574.

(+)-Isopropyl 3-benzyl-2-oxochromane-3-carboxylate (3m'):

Kinetic resolution from hydrogenation of **3l**. 45 mg, 46% yield, 80.8% ee, $[\alpha]^{20}_D$ = +7.62 (*c* 0.90, $CHCl_3$). HPLC: Chiralpak AS-H column, 230 nm, 30 °C, *n*-Hexane/*i*-PrOH = 90/10, flow = 0.8 mL/min, retention time 7.6 min (major) and 8.1 min.

(+)-Ethyl 2-benzyl-3-hydroxy-2-(2-hydroxy-4-methylbenzyl)propanoate (4n):

React for 56 h. 50 mg, 50% yield, colorless oil, new compound, R_f = 0.40 (hexanes/ethyl acetate

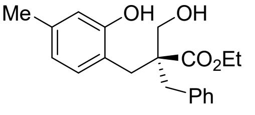

5/1), 78.1% ee, $[\alpha]^{20}_D$ = +9.79 (*c* 1.00, $CHCl_3$). ^{1}H NMR (400 MHz, $CDCl_3$) δ 8.05 (brs, 1H), 7.26-7.18 (m, 3H), 7.12-6.96 (m, 3H), 6.82-6.58 (m, 2H), 4.24-4.08 (m, 2H), 3.68 (d, *J* = 11.5 Hz, 1H), 3.39 (d, *J* = 11.8 Hz, 1H), 3.18 (d, *J* = 13.8 Hz, 2H), 3.01-2.82 (m, 2H), 2.27 (s, 3H), 1.25 (t, *J* = 7.1 Hz, 3H). ^{13}C NMR (100 MHz, $CDCl_3$) δ 175.4, 155.5, 138.7, 136.7, 132.3, 129.8, 128.4, 126.9, 121.3, 119.2, 117.8, 61.8, 61.0, 53.5, 40.6, 34.5, 21.1, 14.2. The HPLC: Chiralpak IA column, 230 nm, 30 °C, *n*-Hexane/*i*-PrOH = 95/5, flow = 0.8 mL/min, retention time 14.9 min and 18.1 min (major). HRMS Calculated for $C_{20}H_{24}O_4Na$ $[M+Na]^+$ 351.1567, found: 351.1568.

(+)-Ethyl 3-benzyl-7-methyl-2-oxochromane-3-carboxylate (3n'):

Kinetic resolution from hydrogenation of **3m**. 49 mg, 50% yield, 88.1% ee, $[\alpha]^{20}_D$ = +29.00 (*c* 0.98, $CHCl_3$). HPLC: Chiralpak IB column, 230 nm, 30 °C, *n*-Hexane/*i*-PrOH = 97/3, flow = 0.8 mL/min, retention time 9.0 min (major) and 10.6 min.

(+)-Ethyl 2-benzyl-2-(4-chloro-2-hydroxybenzyl)-3-hydroxypropanoate (4o):

React for 72 h. 51 mg, 50% yield, colorless oil, new compound, R_f = 0.40 (hexanes/ethyl acetate 5/1), 59.3% ee, $[\alpha]^{20}_D$ = +10.33 (*c* 1.02, $CHCl_3$). ^{1}H NMR (400 MHz, $CDCl_3$) δ 8.52 (brs, 1H),

7.26-7.18 (m, 3H), 7.13-7.00 (m, 3H), 6.91 (d, J = 2.1 Hz, 1H), 6.86-6.77 (m, 1H), 4.18-4.09 (m, 2H), 3.64 (d, J = 11.7 Hz, 1H), 3.41 (d, J = 11.7 Hz, 1H), 3.16 (d, J = 13.5 Hz, 2H), 2.98 (d, J = 14.2 Hz, 1H), 2.87 (d, J = 13.5 Hz, 1H), 1.23 (t, J = 7.2 Hz, 3H). ^{13}C NMR (100 MHz, $CDCl_3$) δ 175.0, 156.5, 136.4, 133.7, 133.2, 129.8, 128.4, 127.0, 121.2, 120.6, 117.5, 61.6, 61.2, 53.5, 40.7, 34.2, 14.1. HPLC: Chiralpak IB column, 230 nm, 30 °C, *n*-Hexane/*i*-PrOH = 95/5, flow = 0.8 mL/min, retention time 14.2 min (major) and 15.5 min. HRMS Calculated for $C_{19}H_{21}ClO_4Na$ $[M+Na]^+$ 371.1021 (^{35}Cl), found: 371.1027 (^{35}Cl). $[M+H]^+$ 373.1004 (^{37}Cl), found: 373.1003 (^{37}Cl).

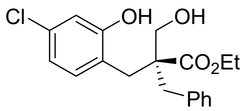

(+)-Ethyl 3-benzyl-7-chloro-2-oxochromane-3-carboxylate (3o'):

Kinetic resolution from hydrogenation of **3n**. 51 mg, 49% yield, 82.1% ee, $[\alpha]^{20}_D$ = +32.32 (*c* 1.02, $CHCl_3$). HPLC: Chiralpak AS-H column, 230 nm, 30 °C, *n*-Hexane/*i*-PrOH = 97/3, flow = 0.8 mL/min, retention time 9.3 min (major) and 10.0 min.

(+)-Ethyl 2-benzyl-3-hydroxy-2-(2-hydroxy-4-methoxybenzyl)propanoate (4p):

React for 72 h. 67 mg, 65% yield, colorless oil, new compound, R_f = 0.25 (hexanes/ethyl acetate 5/1), 49.5% ee, $[\alpha]^{20}_D$ = +14.74 (*c* 1.34, $CHCl_3$). ^{1}H NMR (400 MHz, $CDCl_3$) δ 8.47 (brs, 1H), 7.24-7.15 (m, 3H), 7.12-7.05 (m, 2H), 7.00 (d, J = 8.4 Hz, 1H), 6.53-6.36 (m, 2H), 4.18-4.06 (m, 2H), 3.72 (d, J = 2.2 Hz, 3H), 3.65 (d, J = 11.6 Hz, 1H), 3.42 (d, J = 11.6 Hz, 1H), 3.20-3.09 (m, 2H), 3.01-2.84 (m, 2H), 1.22 (t, J = 7.2 Hz, 3H). ^{13}C NMR (100 MHz, $CDCl_3$) δ 175.3, 160.0, 156.5, 136.8, 132.9, 129.8, 128.4, 126.8, 114.8, 106.7, 102.4, 61.5, 61.0, 55.3, 53.7, 40.7, 34.1, 14.1. HPLC: Chiralpak IC column, 230 nm, 30 °C, *n*-Hexane/*i*-PrOH = 80/20, flow = 0.8 mL/min, retention time 9.0 min (major) and 11.3 min. HRMS Calculated for $C_{20}H_{25}O_5$ $[M+H]^+$ 345.1697, found: 345.1697.

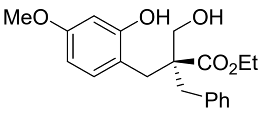

(+)-Ethyl 3-benzyl-7-methoxy-2-oxochromane-3-carboxylate (3p'):

Kinetic resolution from hydrogenation of **3o**. 36 mg, 35% yield, 97.9% ee, $[\alpha]^{20}_D$ = +42.97 (*c* 0.72, $CHCl_3$). HPLC: Chiralcel OD-H column, 230 nm, 30 °C, *n*-Hexane/*i*-PrOH = 90/10, flow = 0.8 mL/min, retention time 9.6 min (major) and 10.8 min.

---

5. Experiment at Gram Scale

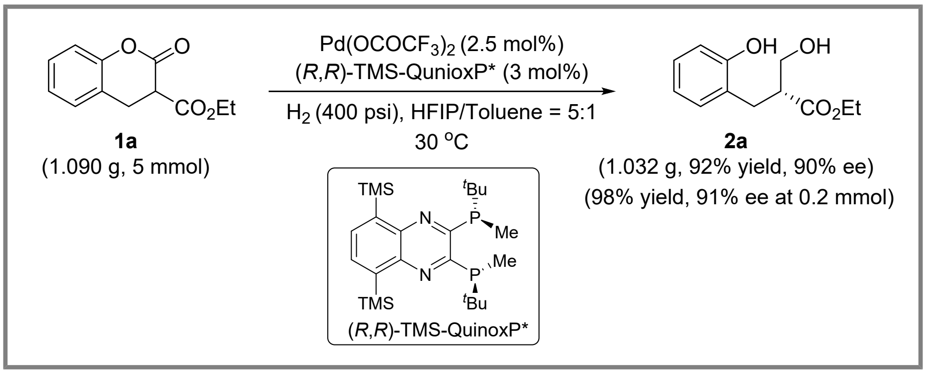

The metal precursor $Pd(OCOCF_3)_2$ (0.125 mmol, 40.3 mg, 2.5 mol%), chiral ligand (*R,R*)-TMS-QuinoxP* (0.15 mmol, 71.9 mg, 3 mol%) and degassed anhydrous acetone (10 mL) were placed in a dried Schlenk tube under nitrogen atmosphere. The mixture was stirred under room temperature for 2 hour. The solvent was removed under vacuum to give the chiral catalyst. This catalyst was taken into a glove box filled with nitrogen and dissolved in solvent (HFIP/toluene = 5:1, 6.0 mL). To the mixture of lactone **1a** (5 mmol, 1.090 g) and solvent (HFIP/toluene = 5:1, 6.0 mL) was added the above catalyst solution, and the mixture was transferred to an autoclave, which was charged hydrogen gas (400 psi). The reaction mixture was stirred at 30 °C for 120 hours. After the release of hydrogen gas, the autoclave was opened and the volatiles were removed under the reduced pressure. Then, the residue was purified by column chromatography on silica gel using using dicholormethane/methanol (40:1) as eluent to give the pure reductive product **2a** (1.032 g, 92% yield, 90% ee).

The enantiomeric excess of the reductive product **2a** was determined by the chiral HPLC with the Chiralpak AS-H column.

6. Product Elaboration

6.1 Transesterification

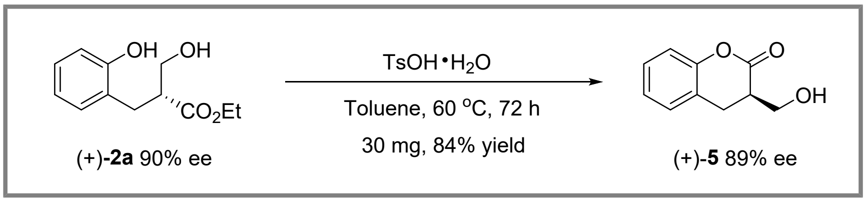

To a Schlenk tube, reductive product ethyl 3-hydroxy-2-(2-hydroxybenzyl)propanoate (+)-**2a** (90% ee, 44.8 mg, 0.2 mmol), toluene (4.0 mL) and *p*-toluenesulfonic acid monohydrate (3.8 mg, 0.02 mmol) were added under nitrogen gas. Then the mixture was stirred for 72 hours under 60 °C. After completion of reaction (monitored by TLC), water (10 mL) and ethyl acetate (5 mL) were added, and the water layer was extracted with ethyl acetate three times. The combined organic layers were dried over anhydrous sodium sulfate, filtered and concentrated under the reduced pressure. The crude residue was purified by flash column chromatography on silica gel using dichloromethane/methanol (40:1) as eluent to give the chiral transesterification product (-)-**5** without any loss of optical purity.

(-)-3-(Hydroxymethyl)chroman-2-one (5)

30 mg, 84% yield, viscous oil, new compound, R_f = 0.60 (dichloromethane/methanol 40/1), 89% ee, $[\alpha]^{20}_D$ = -14.09 (*c* 0.44, $CHCl_3$). ^1H NMR (400 MHz, $CDCl_3$) δ 7.28-7.20 (m, 2H), 7.17-7.02 (m, 2H), 4.17-3.84 (m, 2H), 3.04 (t, *J* = 14.3 Hz, 1H), 2.97-2.79 (m, 2H), 2.63 (t, *J* = 6.5 Hz, 1H). ^{13}C NMR (100 MHz, $CDCl_3$) δ 171.0, 151.2, 128.4, 128.2, 124.7, 122.5, 116.8, 61.8, 41.6, 26.3. HPLC: Chiralpak AS-H column, 230 nm, 30 °C, *n*-Hexane/*i*-PrOH = 90/10, flow = 0.8 mL/min, retention time 31.1 min (major) and 32.8. HRMS Calculated for $C_{10}H_{11}O_3$ $[M+H]^+$ 179.0703, found: 179.0697.

6.2 Appel Reaction

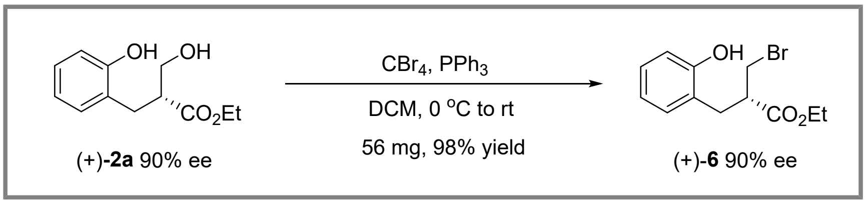

To a Schlenk tube, reductive product ethyl 3-hydroxy-2-(2-hydroxybenzyl)propanoate (+)-**2a** (90% ee, 44.8 mg, 0.2 mmol), dichloromethane (2.0 mL) and carbon tetrabromide (73.0 mg, 0.22 mmol) were added under nitrogen gas. Then triphenylphosphine (78.7 mg, 0.3 mmol) in dichloromethane (2.0 mL) was added dropwise at 0 °C. The mixture was allowed to reach room temperature and stirred for 72 hours. After completion of reaction (monitored by TLC), water (10 mL) and ethyl acetate (5 mL) were added, and the water layer was extracted with ethyl acetate three times. The combined organic layers were dried over anhydrous sodium sulfate, filtered and concentrated under the reduced pressure. The crude residue was purified by flash column chromatography on silica gel using hexanes/ethyl acetate (10/1) as eluent to give the chiral brominated product (-)-**6** without any loss of optical purity.

(-)-Ethyl 3-bromo-2-(2-hydroxybenzyl)propanoate (6)

56 mg, 98% yield, viscous oil, new compound, R_f = 0.40 (hexanes/ethyl acetate 10/1), 90% ee, $[\alpha]^{20}_D$ = -1.52 (*c* 1.12, $CHCl_3$). ^1H NMR (400 MHz, $CDCl_3$) δ 7.17-7.07 (m, 2H), 6.93-6.71 (m, 2H), 6.21 (brs, 1H), 4.27-4.12 (m, 2H), 3.60 (d, *J* = 5.7 Hz, 2H), 3.29-3.18 (m, 1H), 3.13 (dd, *J* = 13.8, 7.9 Hz, 1H), 2.88 (dd, *J* = 13.8, 6.0 Hz, 1H), 1.23 (t, *J* = 7.1 Hz, 3H). ^{13}C NMR (100 MHz, $CDCl_3$) δ 173.7, 154.1, 131.3, 128.4, 124.4, 120.9, 116.4, 61.6, 48.1, 33.0, 31.2, 14.1. The HPLC: Chiralcel OD-H column, 230 nm, 30 °C, *n*-Hexane/*i*-PrOH = 75/25, flow = 0.8 mL/min, retention time 9.9 min and 12.2 (major). HRMS Calculated for $C_{12}H_{16}BrO_3$ $[M+H]^+$ 287.0283 (^{79}Br), found: 287.0278 (^{79}Br). $[M+H]^+$ 289.0263 (^{81}Br), found: 289.0257 (^{81}Br).

6.3 Single Acyl Protection

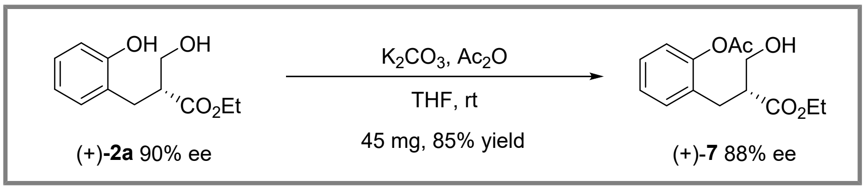

To a Schlenk tube, reductive product ethyl 3-hydroxy-2-(2-hydroxybenzyl)propanoate (+)-**2a** (90% ee, 44.8 mg, 0.2 mmol), tetrahydrofuran (3.0 mL) and potassium carbonate (56.0 mg, 0.4 mmol) were added under nitrogen gas. Then acetic anhydride (23 μL, 0.25 mmol) in was slowly added and stirred for 9 hours. After completion of reaction (monitored by TLC), water (10 mL) and ethyl acetate (5 mL) were added, and the water layer was extracted with ethyl acetate three times. The combined organic layers were dried over anhydrous sodium sulfate, filtered and concentrated under the reduced pressure. The crude residue was purified by flash column chromatography on silica gel using dicholormethane/methanol (40:1) as eluent to give the single protected product (+)-**7** without any loss of optical purity.

(+)-Ethyl 2-(2-acetoxybenzyl)-3-hydroxypropanoate (7)

45 mg, 85% yield, viscous oil, new compound, R_f = 0.60 (dicholormethane/methanol 40/1), 88% ee, $[\alpha]^{20}_D$ = +7.83 (*c* 0.90, $CHCl_3$). ^1H NMR (400 MHz, $CDCl_3$) δ 7.27 (s, 2H), 7.22-7.13 (m, 1H), 7.05 (d, *J* = 7.9 Hz, 1H), 4.16 (q, *J* = 7.0 Hz, 2H), 3.85-3.71 (m, 1H), 3.69-3.61 (m, 1H), 3.02-2.89 (m, 1H), 2.89-2.71 (m, 2H), 2.34 (s, 4H), 1.23 (t, *J* = 7.1 Hz, 3H). ^{13}C NMR (100 MHz, $CDCl_3$) δ 174.6, 169.6, 149.1, 131.1, 130.7, 128.0, 126.2, 122.6, 61.9, 60.9, 47.6, 28.9, 21.0, 14.2. HPLC: Chiralcel OJ-H column, 230 nm, 30 °C, *n*-Hexane/*i*-PrOH = 70/30, flow = 0.8 mL/min, retention time 6.9 min (major) and 8.0. HRMS Calculated for$C_{14}H_{19}O_5$ $[M+H]^+$ 267.1227, found: 267.1242.

6.4 Double Acyl Protection

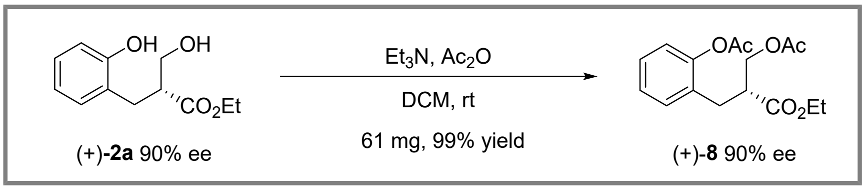

To a Schlenk tube, reductive product ethyl 3-hydroxy-2-(2-hydroxybenzyl)propanoate (+)-**2a**

(90% ee, 44.8 mg, 0.2 mmol), dichloromethane (2.0 mL) and triethylamine (69 μL, 0.5 mmol) were added under nitrogen gas. Then acetic anhydride (47 μL, 0.5 mmol) in was slowly added and stirred overnight. After completion of reaction (monitored by TLC), water (10 mL) and ethyl acetate (5 mL) were added, and the water layer was extracted with ethyl acetate three times. The combined organic layers were dried over anhydrous sodium sulfate, filtered and concentrated under the reduced pressure. The crude residue was purified by flash column chromatography on silica gel using hexanes/ethyl acetate (5/1) as eluent to give the double acyl protected product (-)-**8** without any loss of optical purity.

(-)-Ethyl 3-acetoxy-2-(2-acetoxybenzyl)propanoate (8)

61 mg, 99% yield, viscous oil, new compound, R_f = 0.50 (hexanes/ethyl acetate 5/1), 90% ee, $[\alpha]^{20}_D$ = -2.75 (*c* 1.20, $CHCl_3$). ^{1}H NMR (400 MHz, $CDCl_3$) δ 7.29-7.03 (m, 4H), 4.28-4.16 (m, 2H), 4.16-4.04 (m, 2H), 3.04-2.87 (m, 2H), 2.81-2.68 (m, 1H), 2.34 (s, 3H), 2.02 (s, 3H), 1.17 (t, *J* = 7.1 Hz, 3H). ^{13}C NMR (100 MHz, $CDCl_3$) δ 172.7, 170.6, 169.3, 149.2, 130.9, 130.1, 128.1, 126.2, 122.6, 64.0, 60.9, 45.1, 29.7, 20.9, 20.8, 14.1. HPLC: Chiralcel OD-H column, 254 nm, 30 ^{o}C, *n*-Hexane/*i*-PrOH = 95/5, flow = 0.8 mL/min, retention time 13.3 min and 14.0 (major). The HRMS Calculated for$C_{16}H_{21}O_6$ $[M+H]^+$ 309.1333, found: 309.1337.

---

7. Determination of Absolute Configuration

7.1 Absolute Configuration of Product (+)-2o

To determine the absolute configuration of (+)-ethyl 3-hydroxy-2-((2-hydroxynaphthalen-1-yl) methyl)propanoate (**2o**, >99% ee): (+)-**2o** was completely dissolved in ethyl acetate (1.0 mL), *n*-hexane (5.0 mL) was added slowly at room temperature.The solvent diffused slowly, and the single crystal was obtained after 14 days. The structure in **Figure S1** showed the absolute configuration is (*R*). The CCDC number is 2247354. These details can be obtained free of charge *via* www.ccdc.com.ac.uk/data_request/cif from the Cambridge Crystallographic Data Centre.

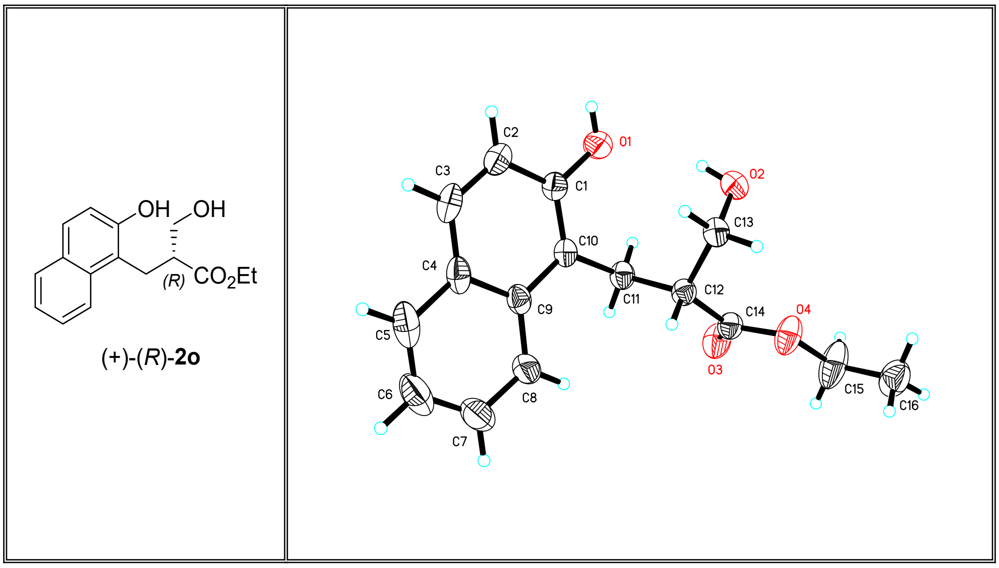

Figure S1. X-Ray Structure of Chiral Reductive Product (+)-(*R*)-**2o**

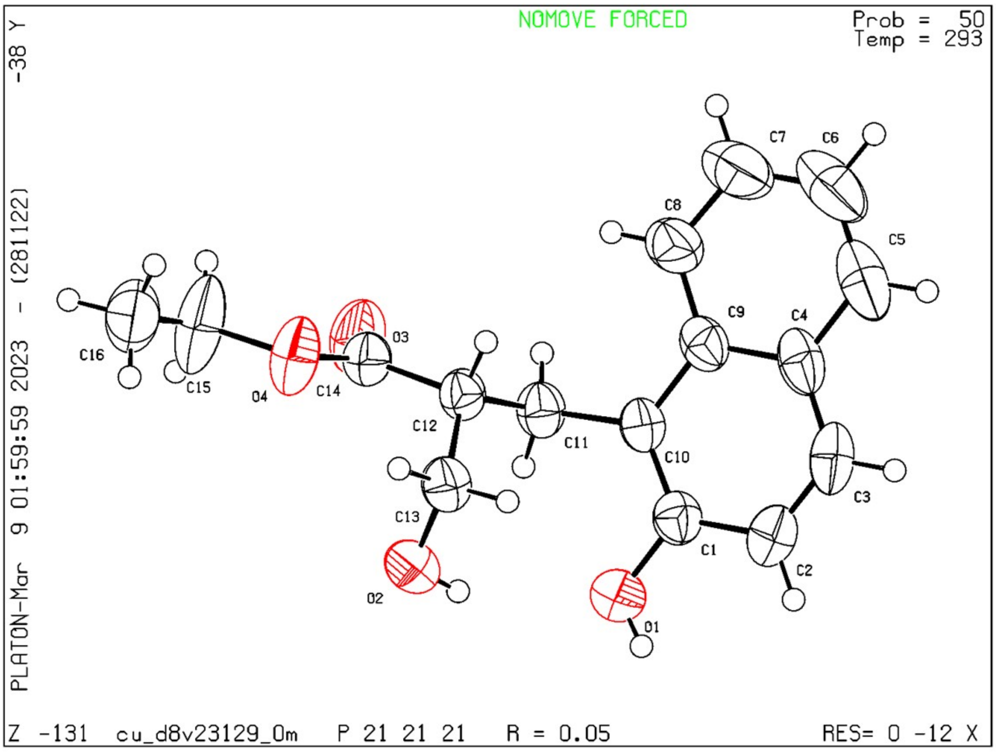

Crystal Data and Structure Refinement for cu_d8v21614_0m for (+)-(*R*)-2n

Identification code	cu_d8v23129_0m	
Empirical formula	C16 H18 O4	
Formula weight	274.30	
Temperature	293(2) K	
Wavelength	1.54178 Å	
Crystal system	Orthorhombic	
Space group	P 21 21 21	
Unit cell dimensions	a = 7.0614(3) Å	α = 90°
	b = 10.6993(4) Å	β = 90°
	c = 18.6749(8) Å	γ = 90°
Volume	1410.93(10) $Å^3$	
Z	4	
Density (calculated)	1.291 Mg/m^3	
Absorption coefficient	0.756 mm^{-1}	
F(000)	584	
Crystal size	0.180 x 0.140 x 0.100 mm^3	
Theta range for data collection	4.763 to 67.464°.	
Index ranges	-8<=h<=8, -12<=k<=12, -22<=l<=20	
Reflections collected	9206	
Independent reflections	2510 [R(int) = 0.0598]	
Completeness to theta = 67.679°	98.1 %	
Absorption correction	Semi-empirical from equivalents	
Max. and min. transmission	0.7533 and 0.4628	
Refinement method	Full-matrix least-squares on F^2	
Data / restraints / parameters	2510 / 0 / 185	
Goodness-of-fit on F^2	1.073	
Final R indices [I>2sigma(I)]	R1 = 0.0464, wR2 = 0.1241	
R indices (all data)	R1 = 0.0519, wR2 = 0.1296	
Absolute structure parameter	0.05(15)	
Extinction coefficient	0.029(5)	
Largest diff. peak and hole	0.158 and -0.140 e.$Å^{-3}$	

7.2 Absolute Configuration of Product (+)-3a’

To determine the absolute configuration of (+)-ethyl 3-benzyl-2-oxochromane-3-carboxylate (**3a’**, >99% ee): (+)-**3a’** was completely dissolved in dicholormethane (0.1 mL), *n*-hexane (0.5 mL) was added slowly at room temperature.The solvent diffused slowly, and the single crystal was obtained after two days. The structure in **Figure S2** showed the absolute configuration is (*S*). The CCDC number is 2247355. These details can be obtained free of charge *via* www.ccdc.com.ac.uk/ data_request/ cif from the Cambridge Crystallographic Data Centre.

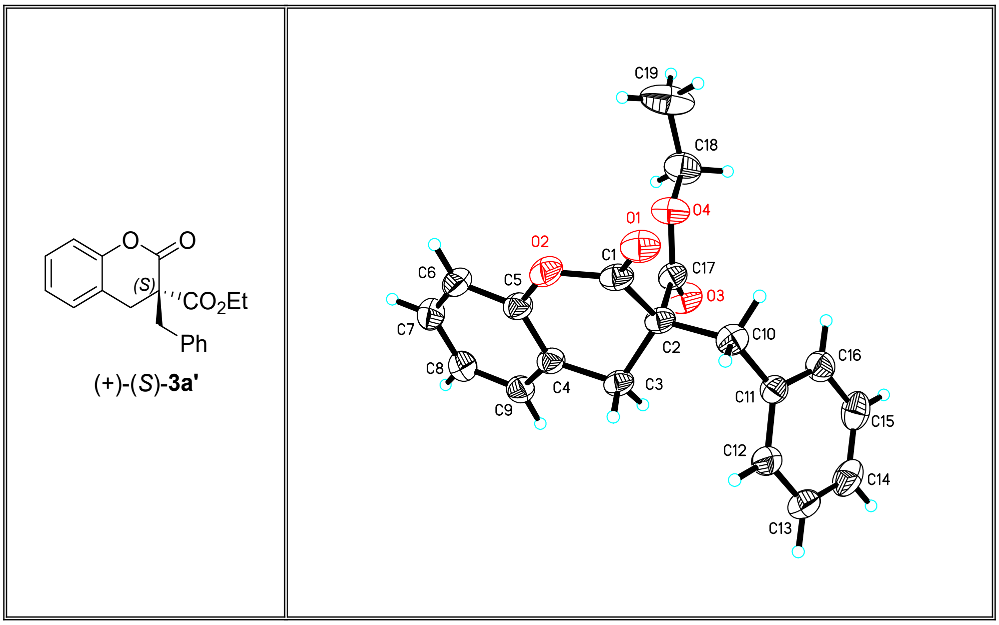

Figure S2.X-Ray Structure of Chiral Compound (+)-(*S*)-**3a’**

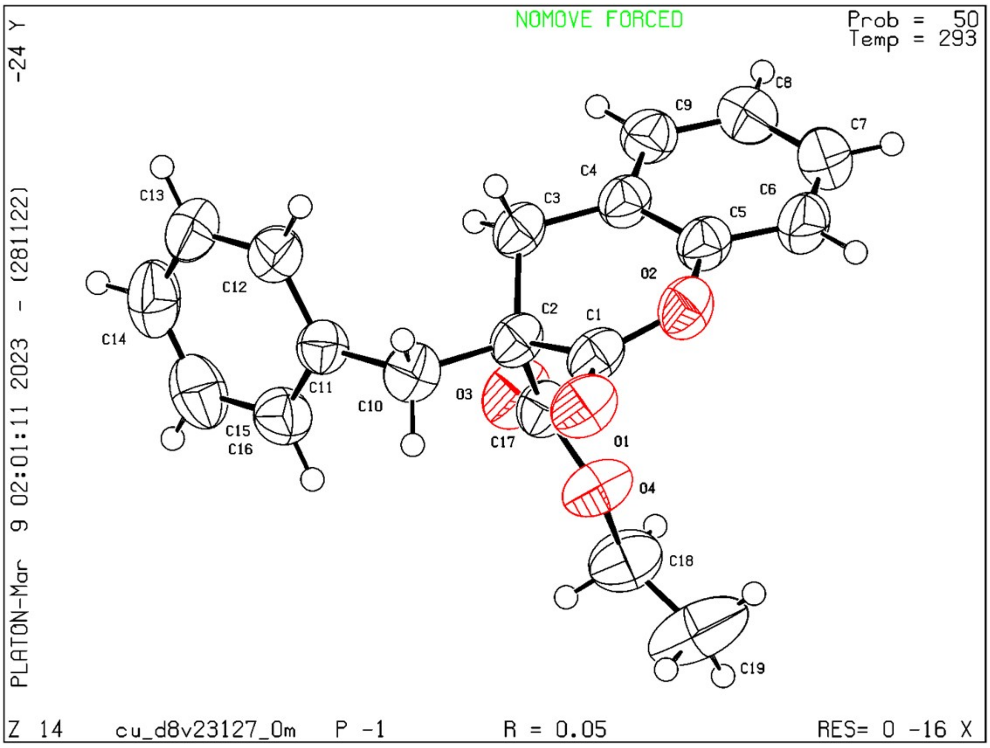

Crystal Data and Structure Refinement for cu_d8v21614_0m for (+)-(*S*)-3a'

Identification code	cu_d8v23127_0m	
Empirical formula	C19 H18 O4	
Formula weight	310.33	
Temperature	293(2) K	
Wavelength	1.54178 Å	
Crystal system	Triclinic	
Space group	P -1	
Unit cell dimensions	a = 8.2353(3) Å	α = 82.2410(10)°
	b = 9.8484(4) Å	β = 70.296(2)°
	c = 11.7427(5) Å	γ = 66.5120(10)°
Volume	822.33(6) $Å^3$	
Z	2	
Density (calculated)	1.253 Mg/m^3	
Absorption coefficient	0.714 mm^{-1}	
F(000)	328	
Crystal size	0.200 x 0.150 x 0.120 mm^3	
Theta range for data collection	3.999 to 67.492°.	
Index ranges	-9<=h<=9, -11<=k<=11, -14<=l<=14	
Reflections collected	28232	
Independent reflections	2856 [R(int) = 0.0623]	
Completeness to theta = 67.679°	96.4 %	
Absorption correction	Semi-empirical from equivalents	
Max. and min. transmission	0.7456 and 0.5300	
Refinement method	Full-matrix least-squares on F^2	
Data / restraints / parameters	2856 / 0 / 210	
Goodness-of-fit on F^2	1.046	
Final R indices [I>2sigma(I)]	R1 = 0.0489, wR2 = 0.1336	
R indices (all data)	R1 = 0.0505, wR2 = 0.1352	
Extinction coefficient	0.059(11)	
Largest diff. peak and hole	0.209 and -0.127 e.$Å^{-3}$	

8. Proposed Mechanism of DKR

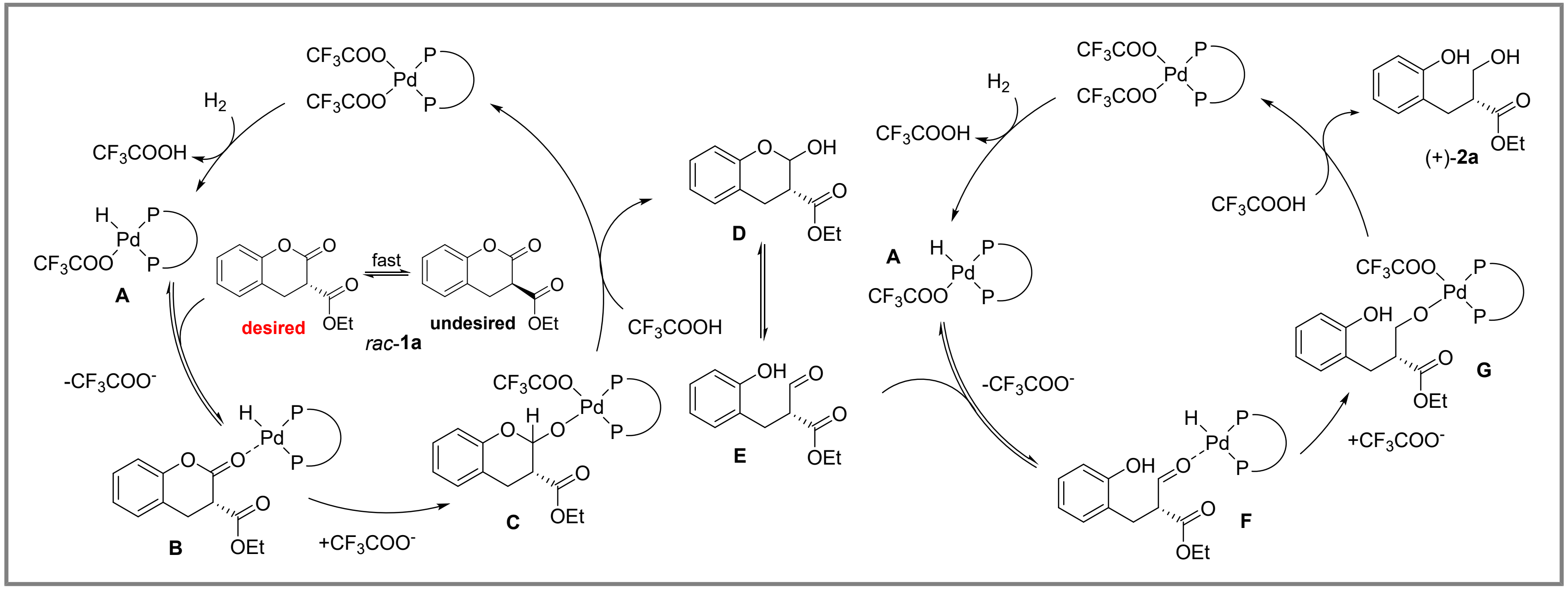

.

9. References

1. Suljic, S; Pietruszka, J. Synthesis of 3-Arylated 3,4-Dihydrocoumarins: Combining Continuous Flow Hydrogenation with Laccase-catalysed Oxidations. *Adv. Synth. Catal.* **2014**, *356*, 1007-1020.
2. Velasco, M.; Romero-Ceronio, N.; Torralba, R.; Abreu, O. H.; Vilchis-Reyes, M. A.; Alarcón-Matus, E.; Ramos-Rivera, E. M.; Aparicio, D. M.; Jiménez, J.; García, E. A.; Cruz, D. C.; Gómez, C. V.; Alvarado, C. Piperidine-iodine as Efficient Dual Catalyst for the One-pot, Three-component Synthesis of Coumarin-3-carboxamides. *Molecules* **2022**, *27*, 4659-4668.
3. Ramachary, D. B.; Kishor, M.; Reddy, Y. V. Development of Pharmaceutical Drugs, Drug Intermediates and Ingredients by Using Direct Organo-click Reactions. *Eur. J. Org. Chem.* **2008**, 975-993.
4. Mitra, A. K.; Misra, S. K.; Patra, A. New Synthesis of 3-Alkyl Coumarins. *Synth. Commun.* **1980**, *10*, 915-919.
5. Liu, Z.; Liu, Q.; Zhang, W.; Mu, R.; Yang, L.; Liu, Z.-L.; Yu, W. Selective Reduction of the Endocyclic Double Bond of 3-Substituted Coumarins by Hantzsch 1,4-Dihydropyridine. *Synthesis* **2006**, *5*, 771-774.

10. Copy of NMR and HPLC Spectra

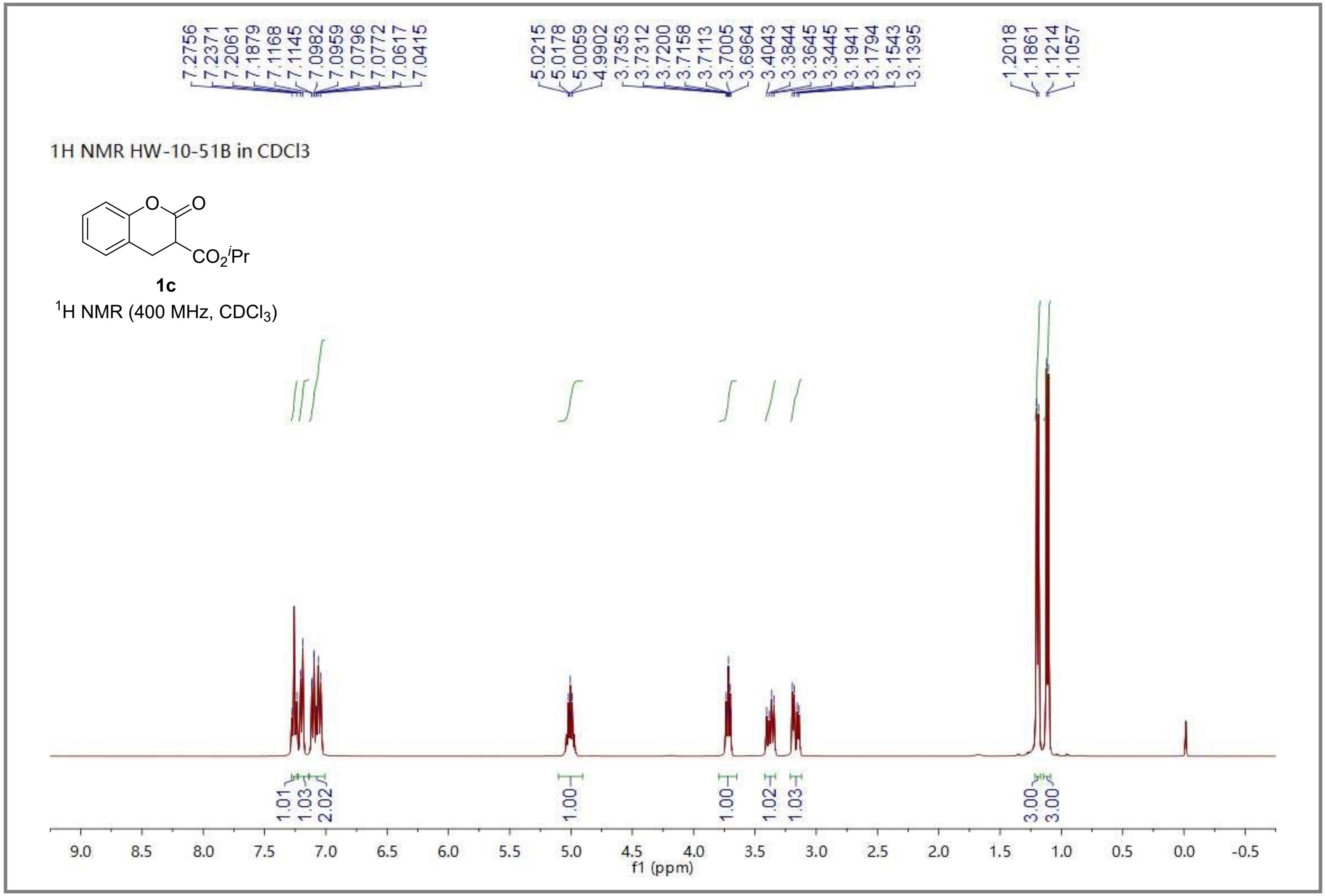

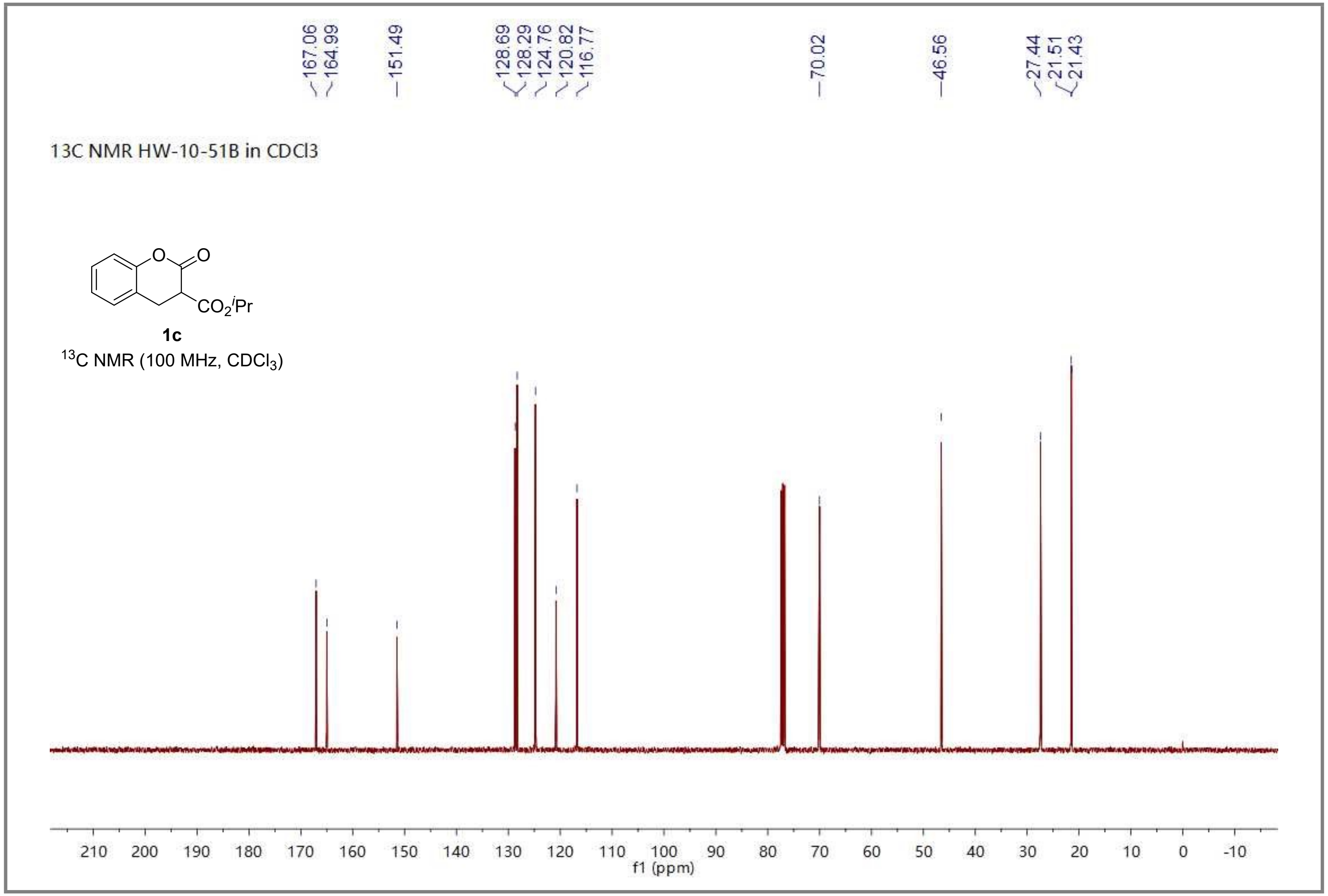
167.06
164.99
151.49
128.69
128.29
124.76
120.82
116.77
70.02
46.56
27.44
21.51
21.43
13C NMR HW-10-51B in CDCl3
O
O
$CO_2{}^iPr$
1c
^{13}C NMR (100 MHz, $CDCl_3$)
210 200 190 180 170 160 150 140 130 120 110 100 90 80 70 60 50 40 30 20 10 0 -10
f1 (ppm)

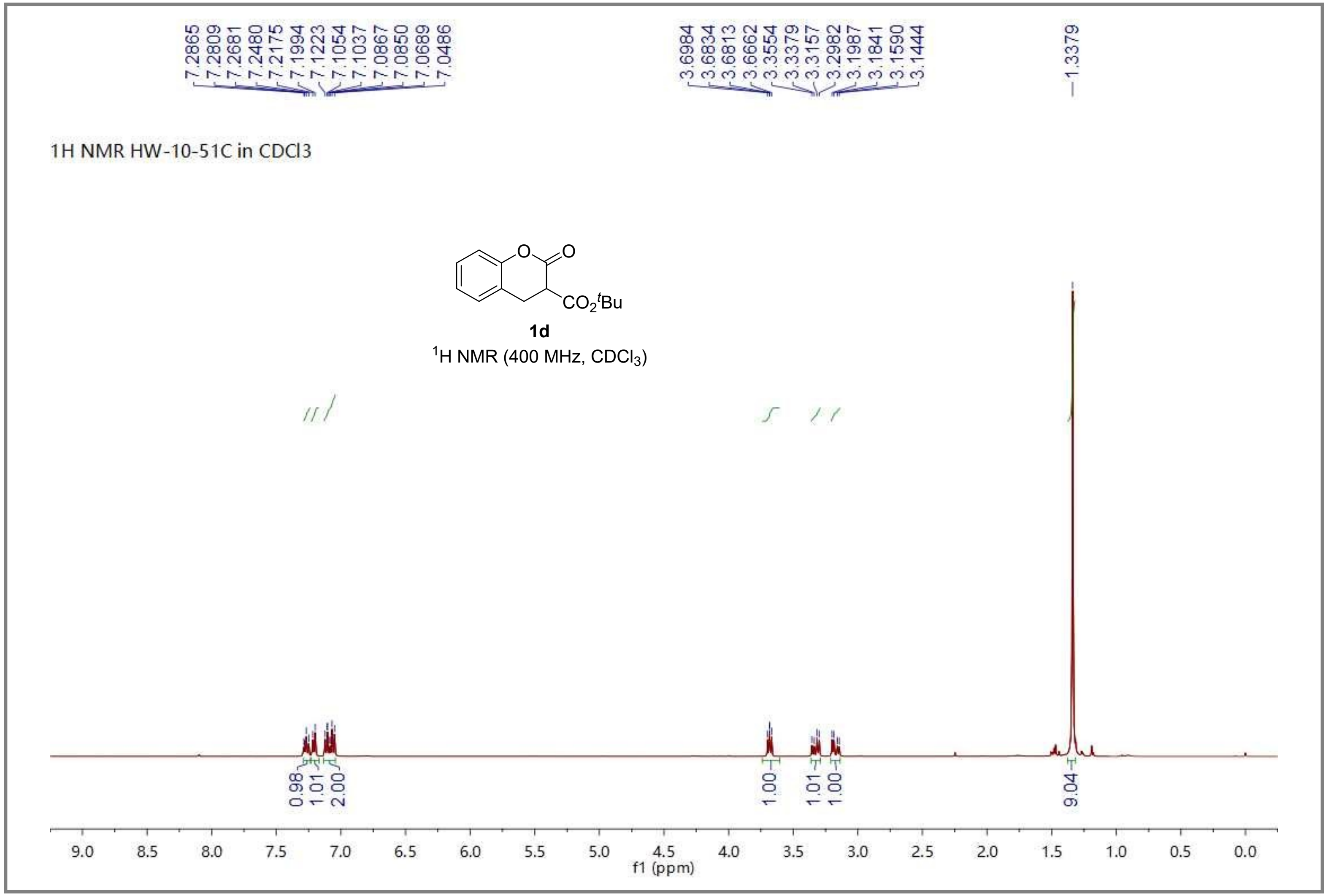
7.2865
7.2809
7.2681
7.2480
7.2175
7.1994
7.1223
7.1054
7.1037
7.0867
7.0850
7.0689
7.0486
3.6984
3.6834
3.6813
3.6662
3.3554
3.3379
3.3157
3.2982
3.1987
3.1841
3.1590
3.1444
1.3379
1H NMR HW-10-51C in CDCl3
$CO_2{}^tBu$
1d
^1H NMR (400 MHz, $CDCl_3$)
0.98
1.01
2.00
1.00
1.01
1.00
9.04
9.0
8.5
8.0
7.5
7.0
6.5
6.0
5.5
5.0
4.5
4.0
3.5
3.0
2.5
2.0
1.5
1.0
0.5
0.0
f1 (ppm)

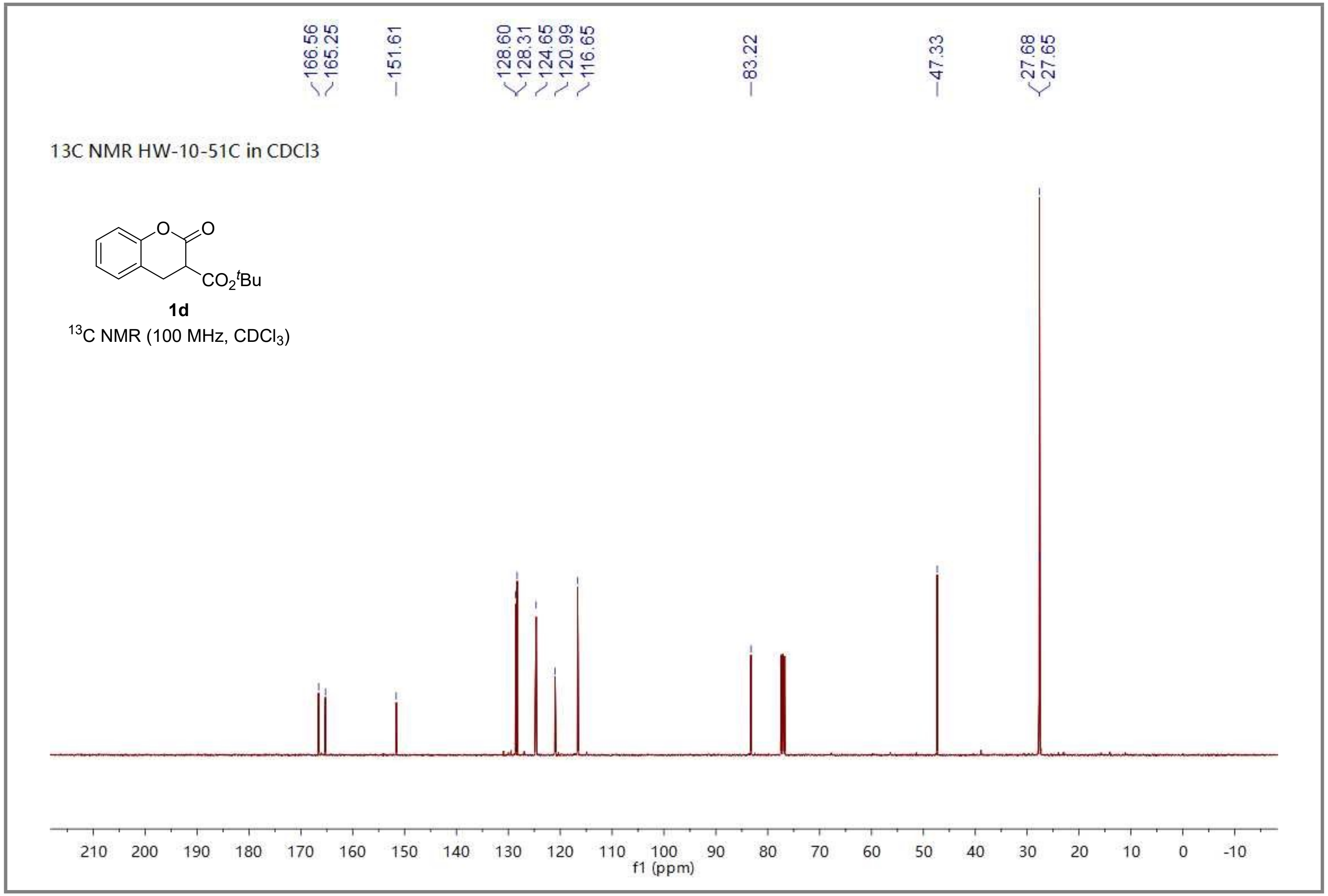

166.56
165.25
151.61
128.60
128.31
124.65
120.99
116.65
83.22
47.33
27.68
27.65
13C NMR HW-10-51C in CDCl3
CO2tBu
1d
13C NMR (100 MHz, CDCl3)
210 200 190 180 170 160 150 140 130 120 110 100 90 80 70 60 50 40 30 20 10 0 -10
f1 (ppm)

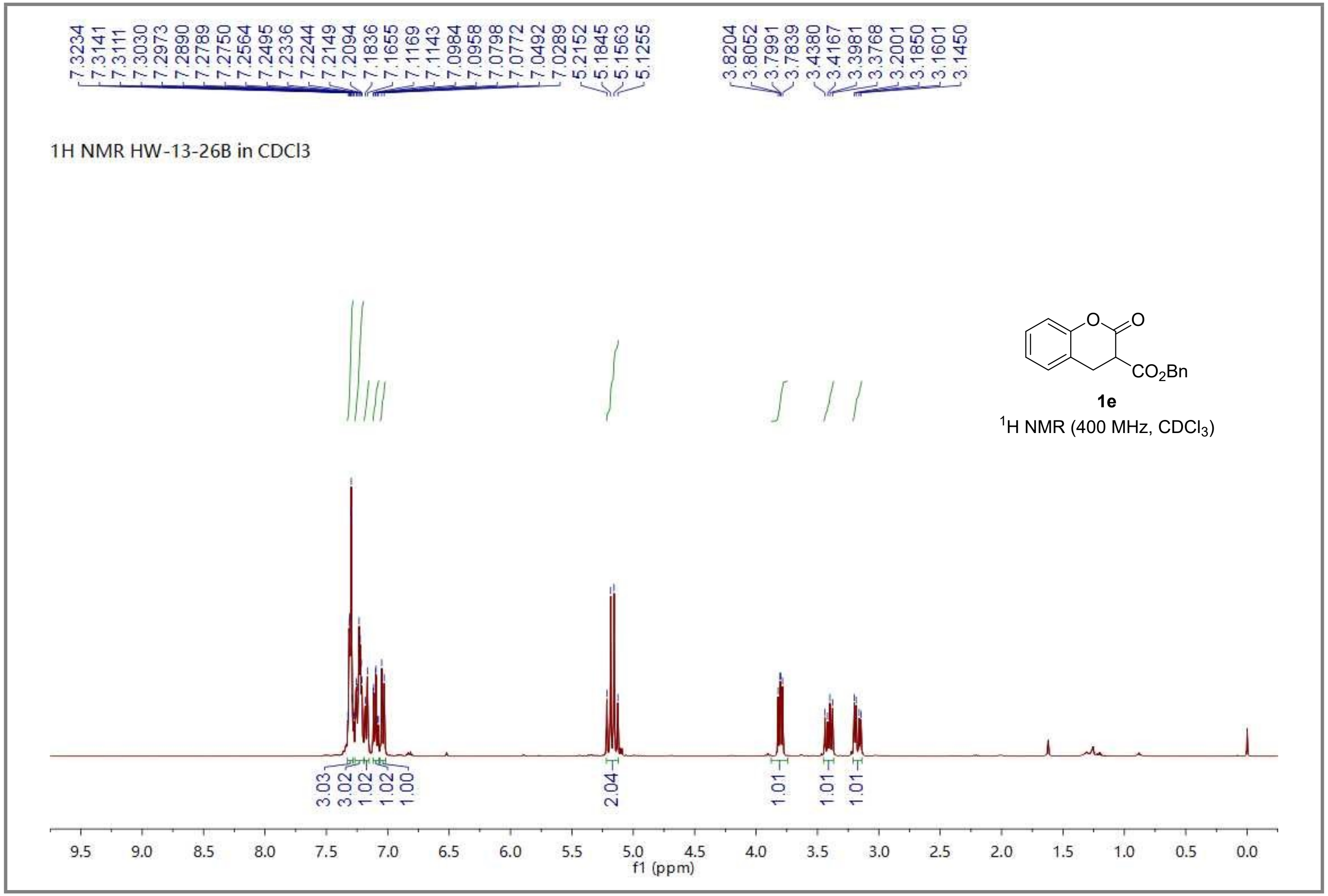

7.3234
7.3141
7.3111
7.3030
7.2973
7.2890
7.2789
7.2750
7.2564
7.2495
7.2336
7.2244
7.2149
7.2094
7.1836
7.1655
7.1169
7.1143
7.0984
7.0958
7.0798
7.0772
7.0492
7.0289
5.2152
5.1845
5.1563
5.1255
3.8204
3.8052
3.7991
3.7839
3.4380
3.4167
3.3981
3.3768
3.2001
3.1850
3.1601
3.1450
1H NMR HW-13-26B in CDCl3
O
O
CO2Bn
1e
1H NMR (400 MHz, CDCl3)
3.03
3.02
1.02
1.02
1.00
2.04
1.01
1.01
1.01
9.5
9.0
8.5
8.0
7.5
7.0
6.5
6.0
5.5
5.0
4.5
4.0
3.5
3.0
2.5
2.0
1.5
1.0
0.5
0.0
f1 (ppm)

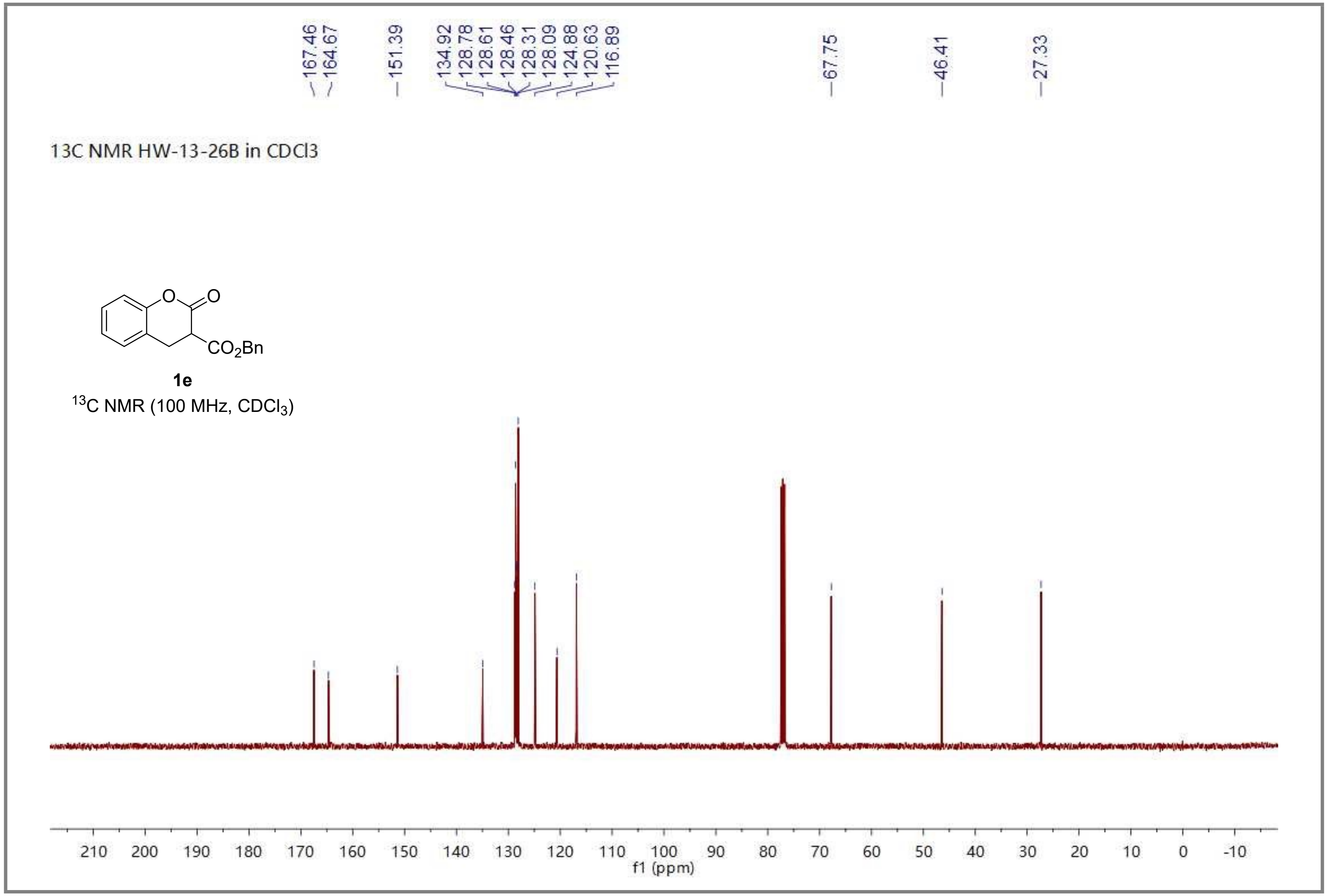
167.46
164.67
151.39
134.92
128.78
128.61
128.46
128.31
128.09
124.88
120.63
116.89
67.75
46.41
27.33
13C NMR HW-13-26B in CDCl3
O
O
CO2Bn
1e
13C NMR (100 MHz, CDCl3)
210 200 190 180 170 160 150 140 130 120 110 100 90 80 70 60 50 40 30 20 10 0 -10
f1 (ppm)

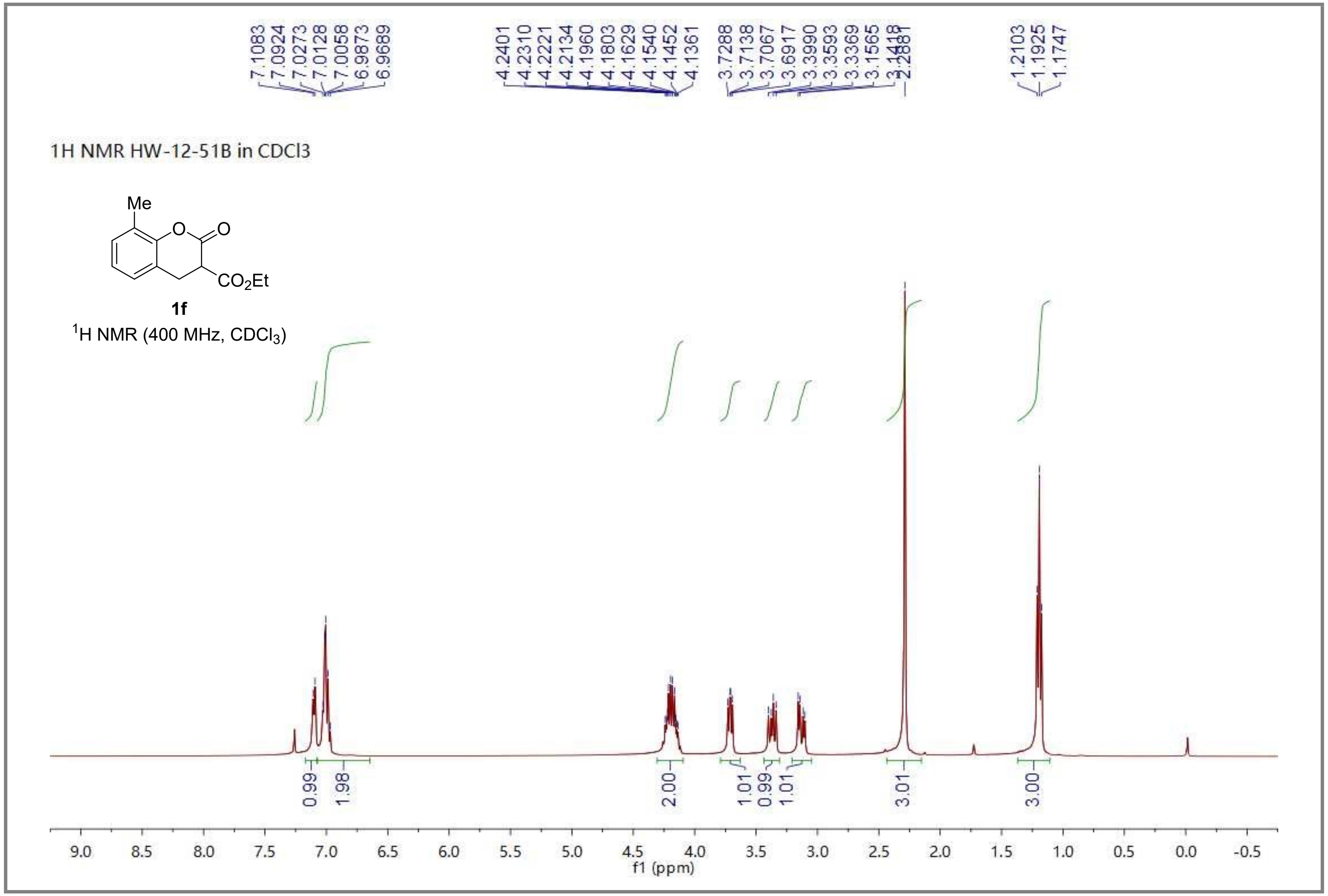

7.1083
7.0924
7.0273
7.0128
7.0058
6.9873
6.9689
4.2401
4.2310
4.2221
4.2134
4.1960
4.1803
4.1629
4.1540
4.1452
4.1361
3.7288
3.7138
3.7067
3.6917
3.3990
3.3593
3.3369
3.1565
3.1418
2.2881
1.2103
1.1925
1.1747
1H NMR HW-12-51B in CDCl3
Me
O
O
CO2Et
1f
1H NMR (400 MHz, CDCl3)
0.99
1.98
2.00
1.01
0.99
1.01
3.01
3.00
9.0
8.5
8.0
7.5
7.0
6.5
6.0
5.5
5.0
4.5
4.0
3.5
3.0
2.5
2.0
1.5
1.0
0.5
0.0
-0.5
f1 (ppm)

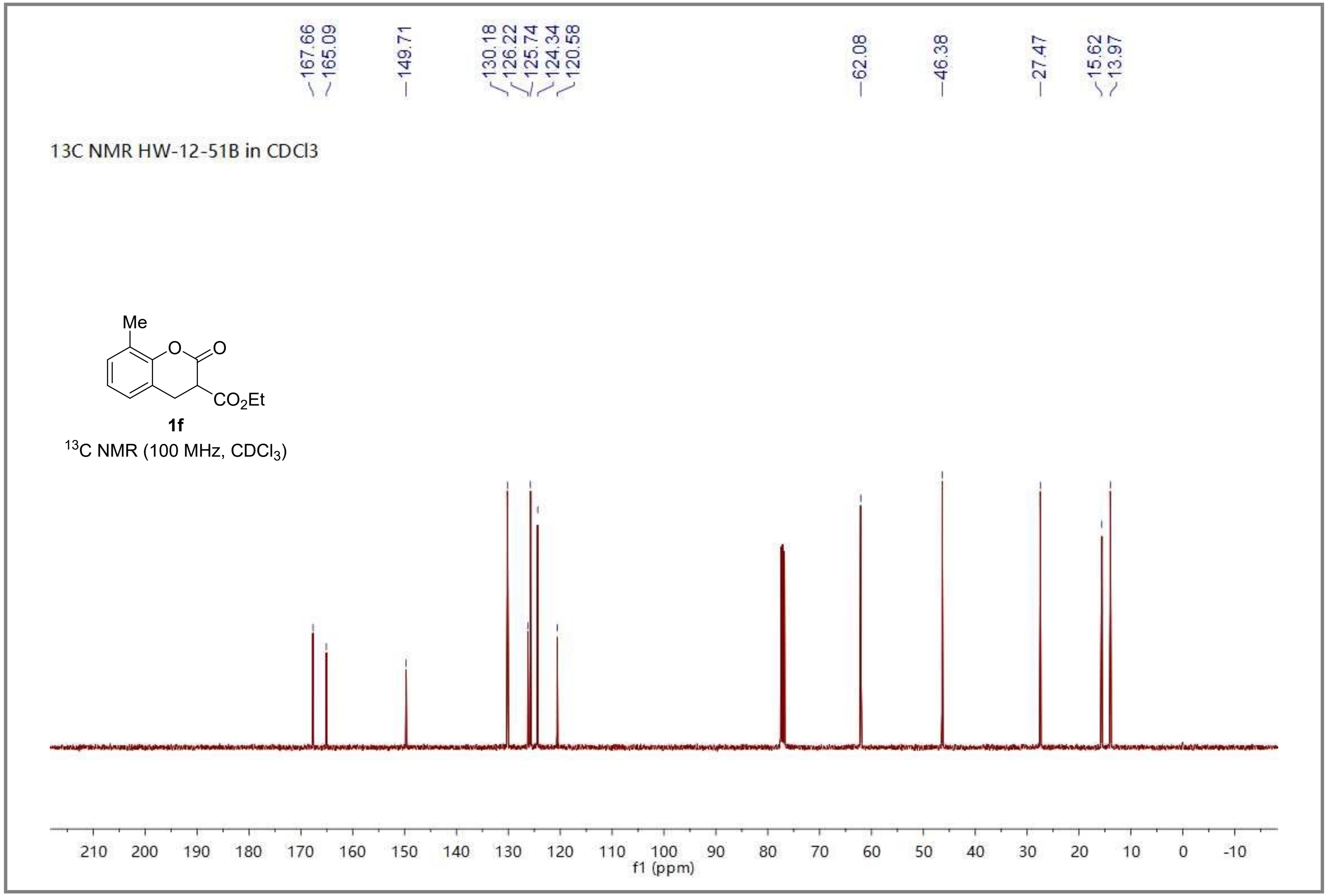

167.66
165.09
149.71
130.18
126.22
125.74
124.34
120.58
62.08
46.38
27.47
15.62
13.97
13C NMR HW-12-51B in CDCl3
Me
O
O
CO2Et
1f
13C NMR (100 MHz, CDCl3)
210 200 190 180 170 160 150 140 130 120 110 100 90 80 70 60 50 40 30 20 10 0 -10
f1 (ppm)

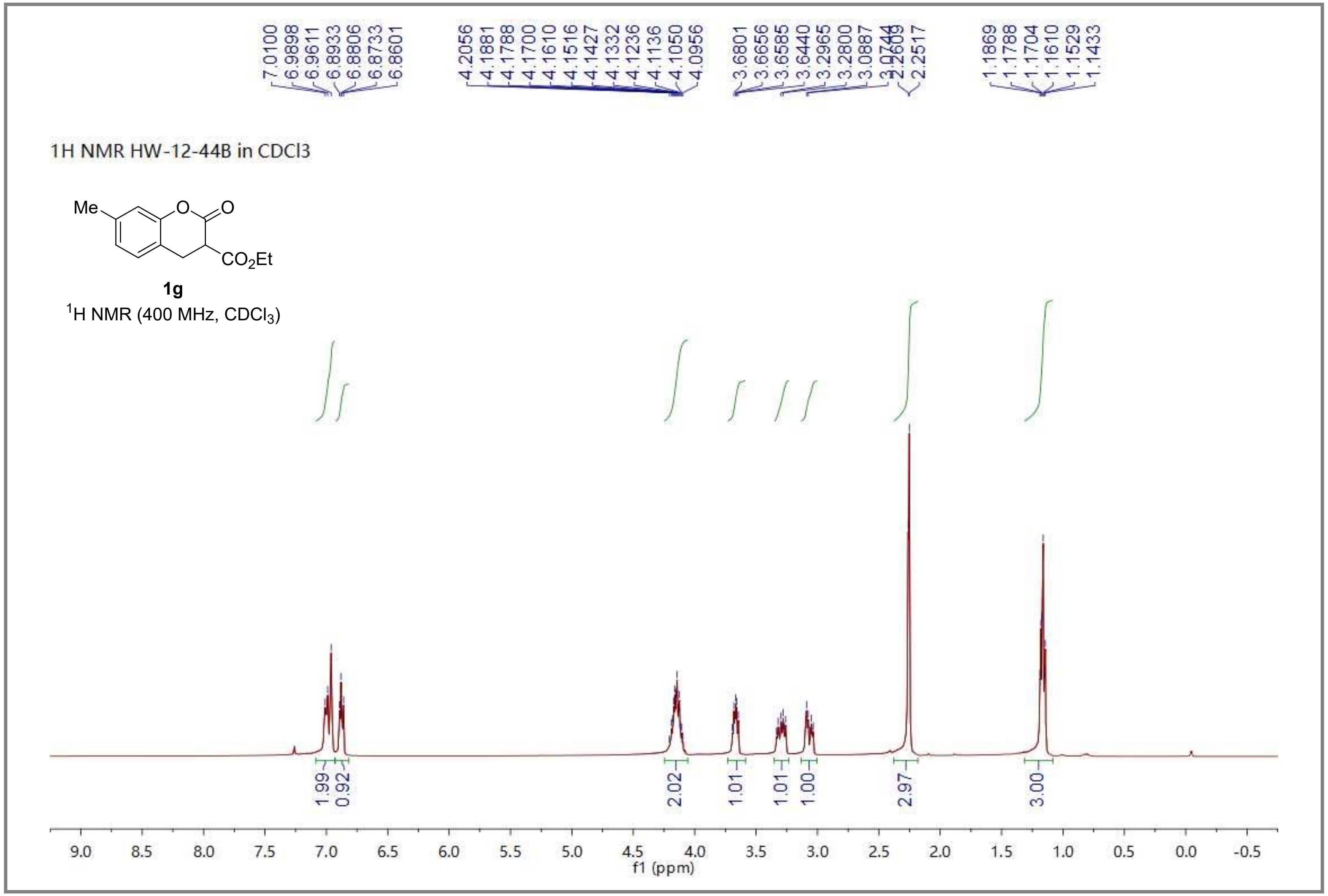
7.0100
6.9898
6.9611
6.8933
6.8806
6.8733
6.8601
4.2056
4.1881
4.1788
4.1700
4.1610
4.1516
4.1427
4.1332
4.1236
4.1136
4.1050
4.0956
3.6801
3.6656
3.6585
3.6440
3.2965
3.2800
3.0887
2.2609
2.2517
1.1869
1.1788
1.1704
1.1610
1.1529
1.1433
1H NMR HW-12-44B in CDCl3
Me
O
O
CO2Et
1g
1H NMR (400 MHz, CDCl3)
1.99
0.92
2.02
1.01
1.01
1.00
2.97
3.00
9.0 8.5 8.0 7.5 7.0 6.5 6.0 5.5 5.0 4.5 4.0 3.5 3.0 2.5 2.0 1.5 1.0 0.5 0.0 -0.5
f1 (ppm)

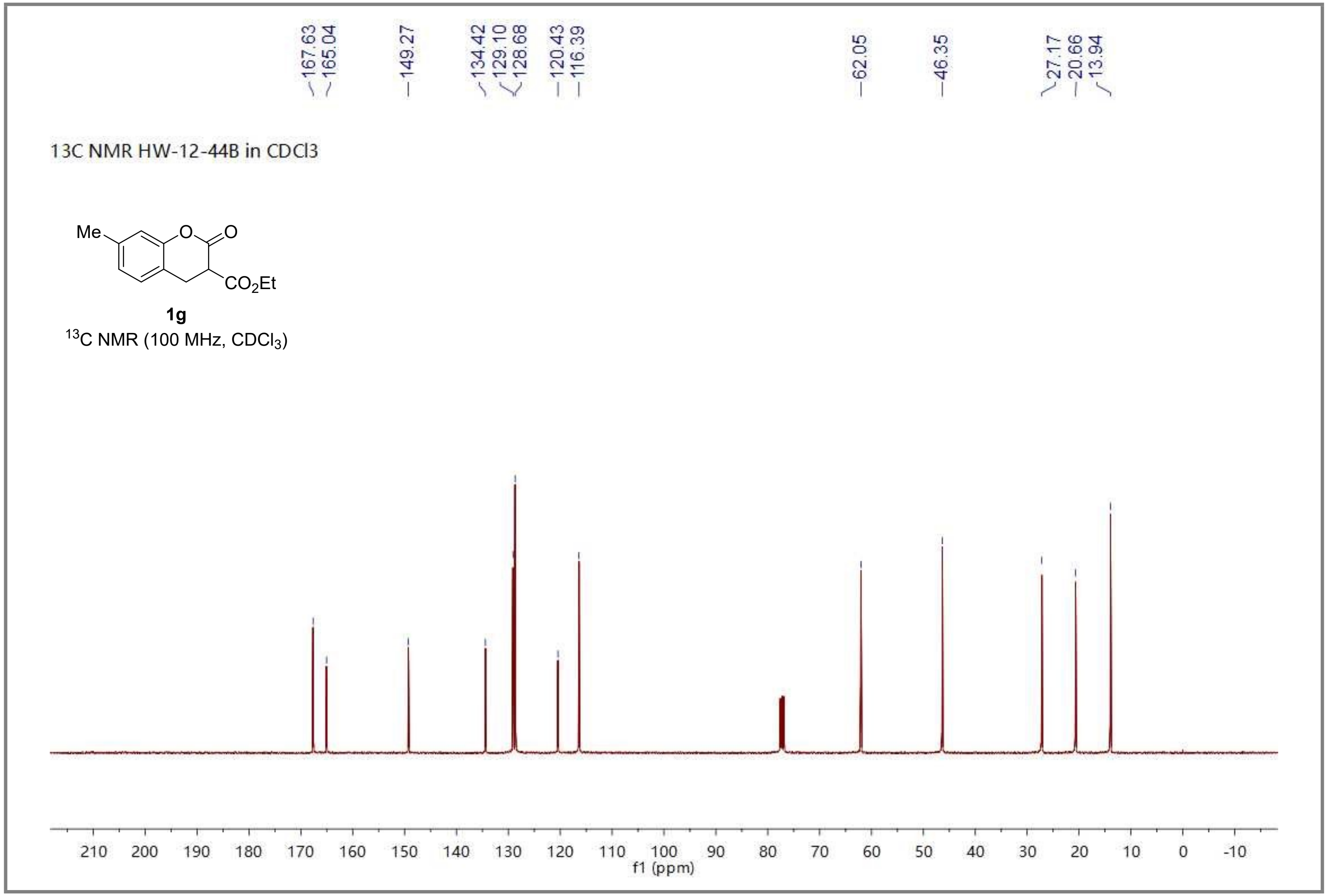
167.63
165.04
149.27
134.42
129.10
128.68
120.43
116.39
62.05
46.35
27.17
20.66
13.94
13C NMR HW-12-44B in CDCl3
Me
O
O
CO2Et
1g
13C NMR (100 MHz, CDCl3)
210 200 190 180 170 160 150 140 130 120 110 100 90 80 70 60 50 40 30 20 10 0 -10
f1 (ppm)

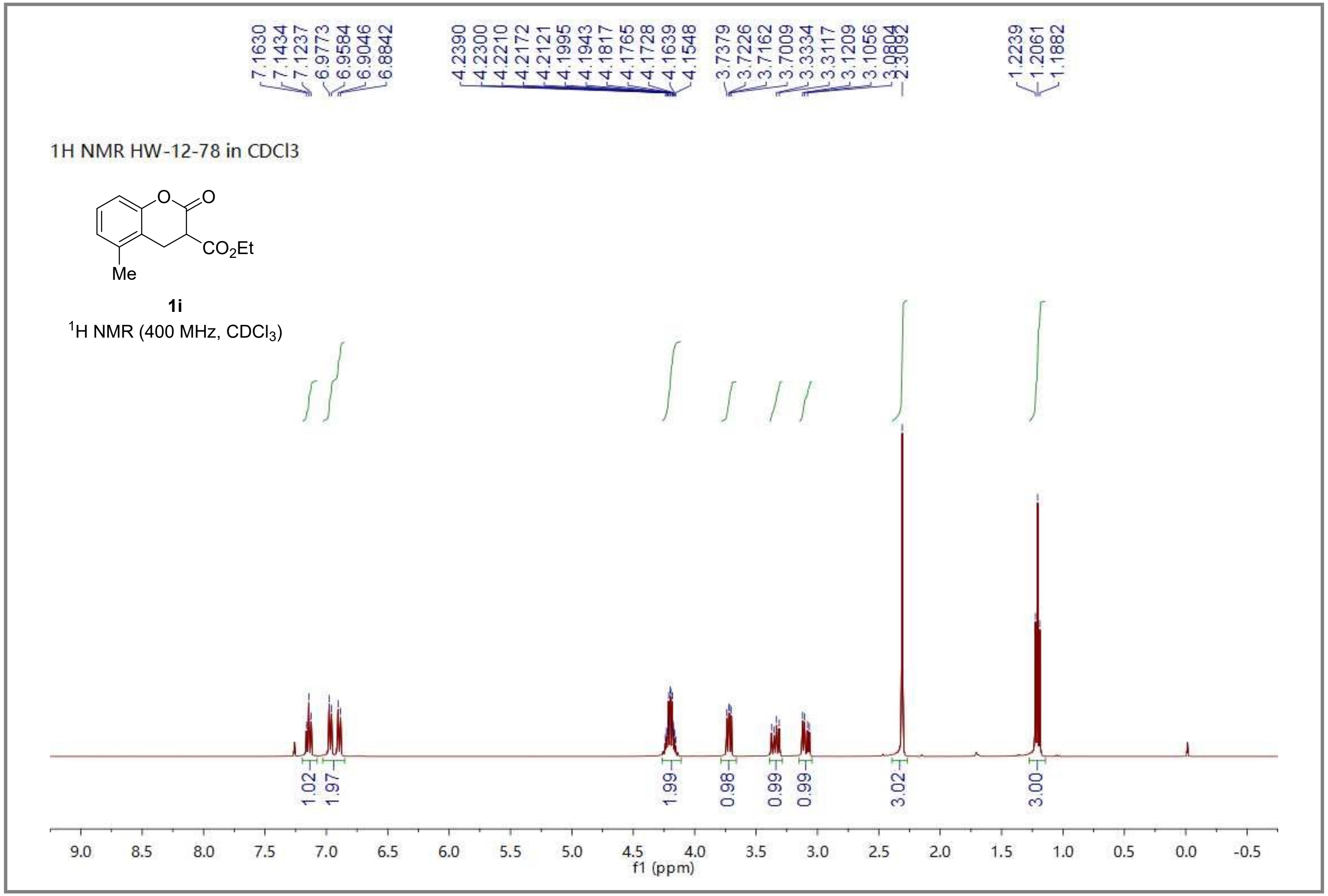

7.1630
7.1434
7.1237
6.9773
6.9584
6.9046
6.8842
4.2390
4.2300
4.2210
4.2172
4.2121
4.1995
4.1943
4.1817
4.1765
4.1728
4.1639
4.1548
3.7379
3.7226
3.7162
3.7009
3.3334
3.3117
3.1209
3.1056
3.0804
2.3092
1.2239
1.2061
1.1882
1H NMR HW-12-78 in CDCl3
CO2Et
Me
1i
1H NMR (400 MHz, CDCl3)
1.02
1.97
1.99
0.98
0.99
0.99
3.02
3.00
9.0
8.5
8.0
7.5
7.0
6.5
6.0
5.5
5.0
4.5
4.0
3.5
3.0
2.5
2.0
1.5
1.0
0.5
0.0
-0.5
f1 (ppm)

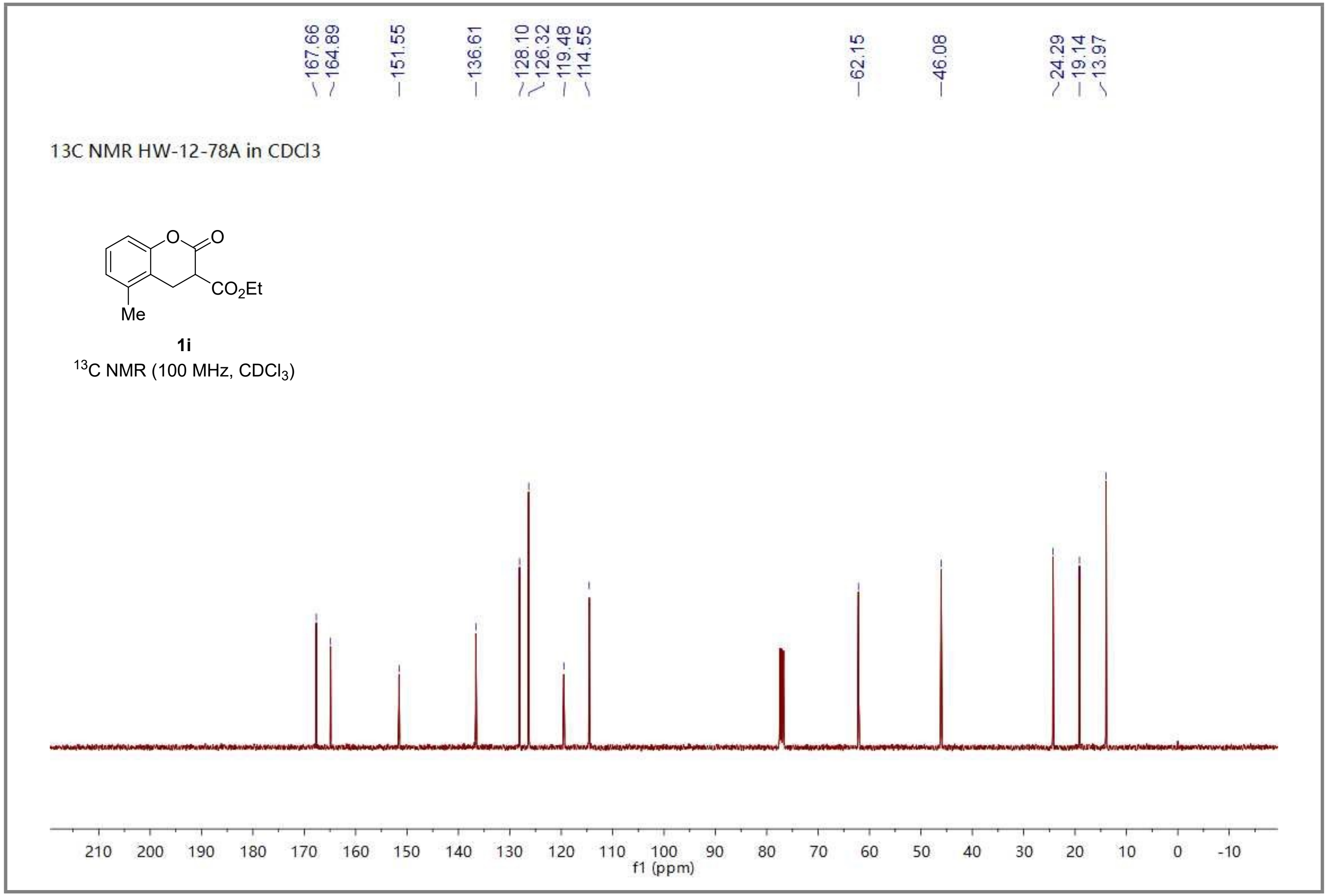

167.66
164.89
151.55
136.61
128.10
126.32
119.48
114.55
62.15
46.08
24.29
19.14
13.97
13C NMR HW-12-78A in CDCl3
O
O
CO2Et
Me
1i
13C NMR (100 MHz, CDCl3)
210 200 190 180 170 160 150 140 130 120 110 100 90 80 70 60 50 40 30 20 10 0 -10
f1 (ppm)

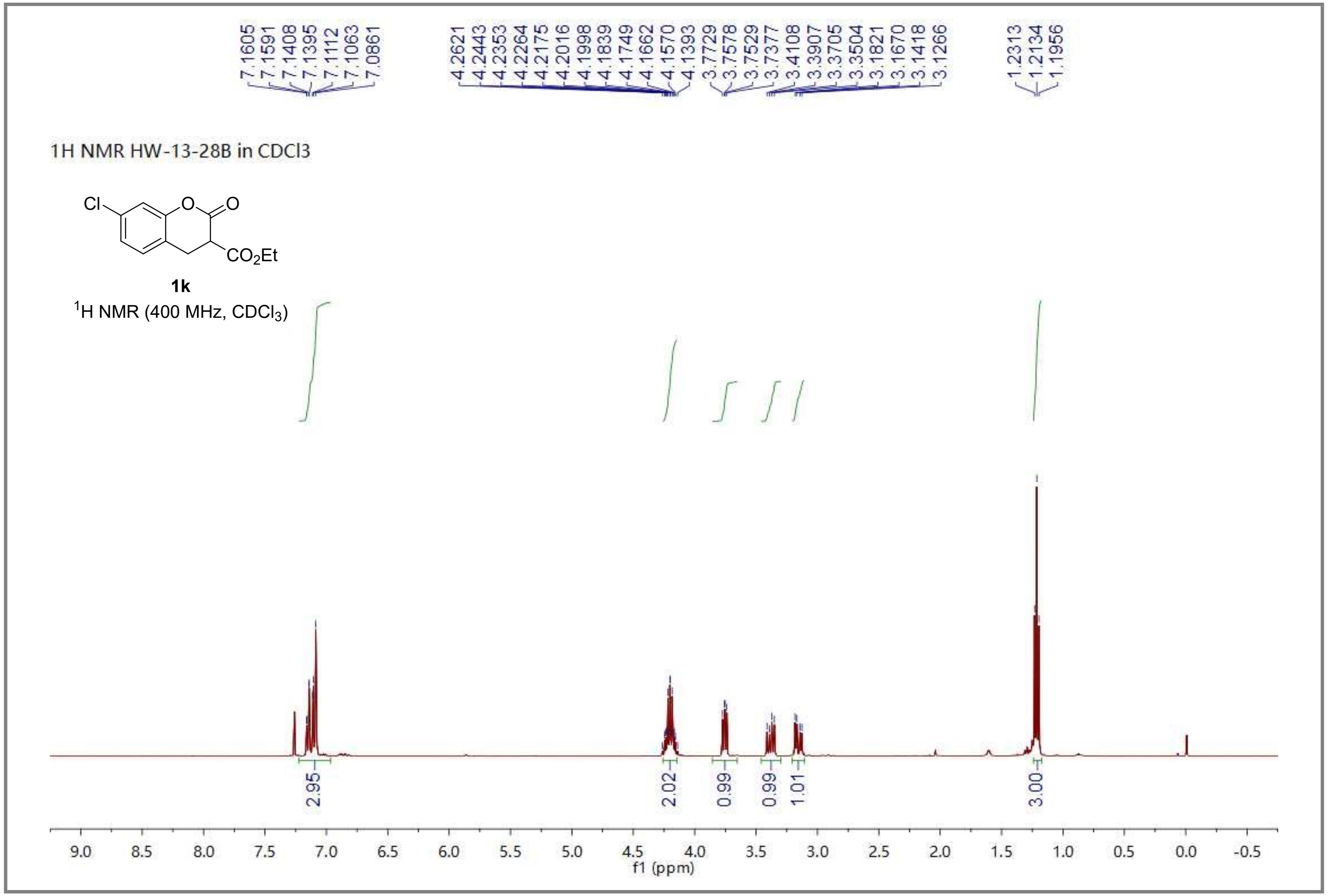
7.1605
7.1591
7.1408
7.1395
7.1112
7.1063
7.0861
4.2621
4.2443
4.2353
4.2264
4.2175
4.2016
4.1998
4.1839
4.1749
4.1662
4.1570
4.1393
3.7729
3.7578
3.7529
3.7377
3.4108
3.3907
3.3705
3.3504
3.1821
3.1670
3.1418
3.1266
1.2313
1.2134
1.1956
1H NMR HW-13-28B in CDCl3
Cl
O
O
CO2Et
1k
1H NMR (400 MHz, CDCl3)
2.95
2.02
0.99
0.99
1.01
3.00
9.0
8.5
8.0
7.5
7.0
6.5
6.0
5.5
5.0
4.5
4.0
3.5
3.0
2.5
2.0
1.5
1.0
0.5
0.0
-0.5
f1 (ppm)

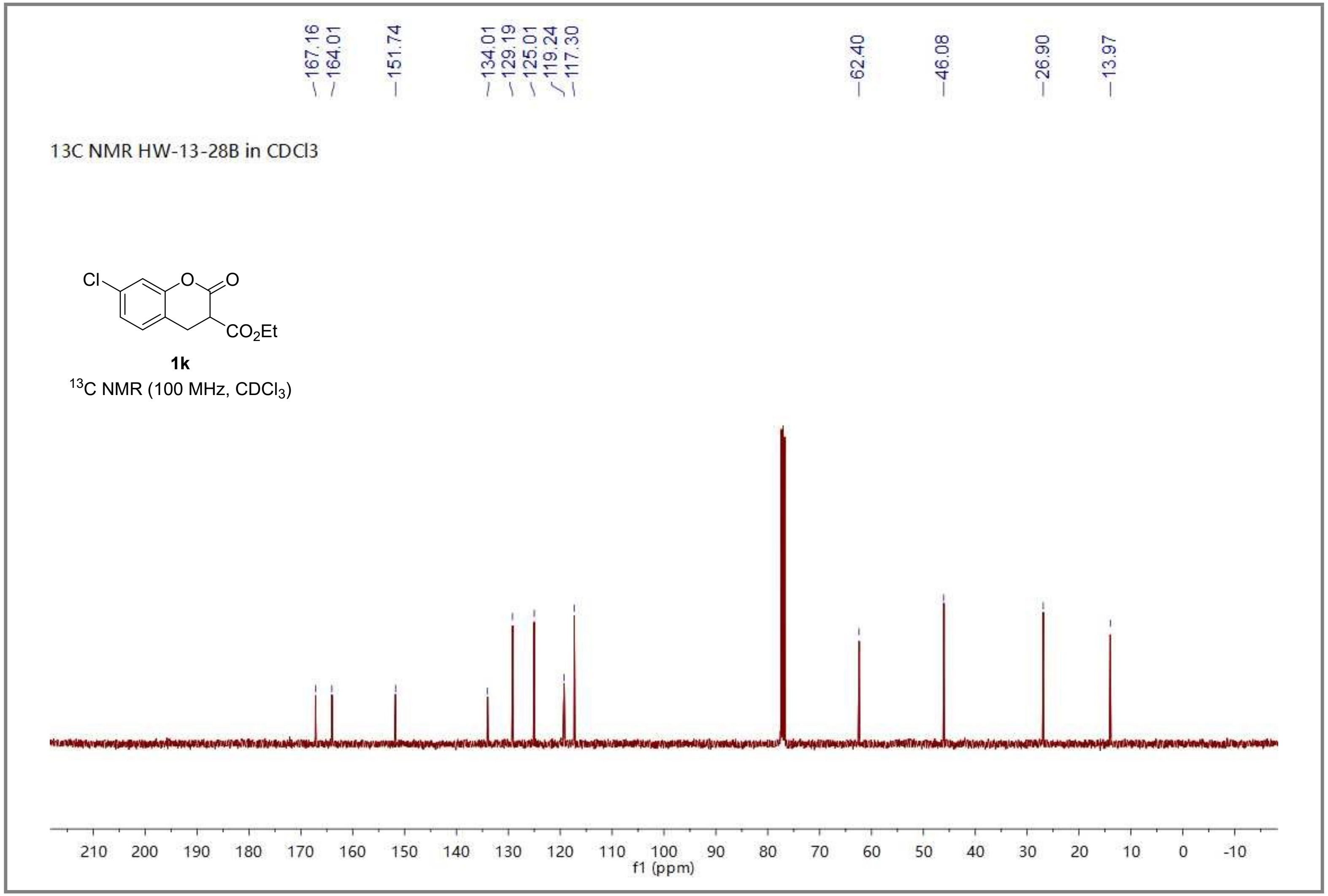
167.16
164.01
151.74
134.01
129.19
125.01
119.24
117.30
62.40
46.08
26.90
13.97
13C NMR HW-13-28B in CDCl3
Cl
O
O
CO2Et
1k
13C NMR (100 MHz, CDCl3)
210 200 190 180 170 160 150 140 130 120 110 100 90 80 70 60 50 40 30 20 10 0 -10
f1 (ppm)

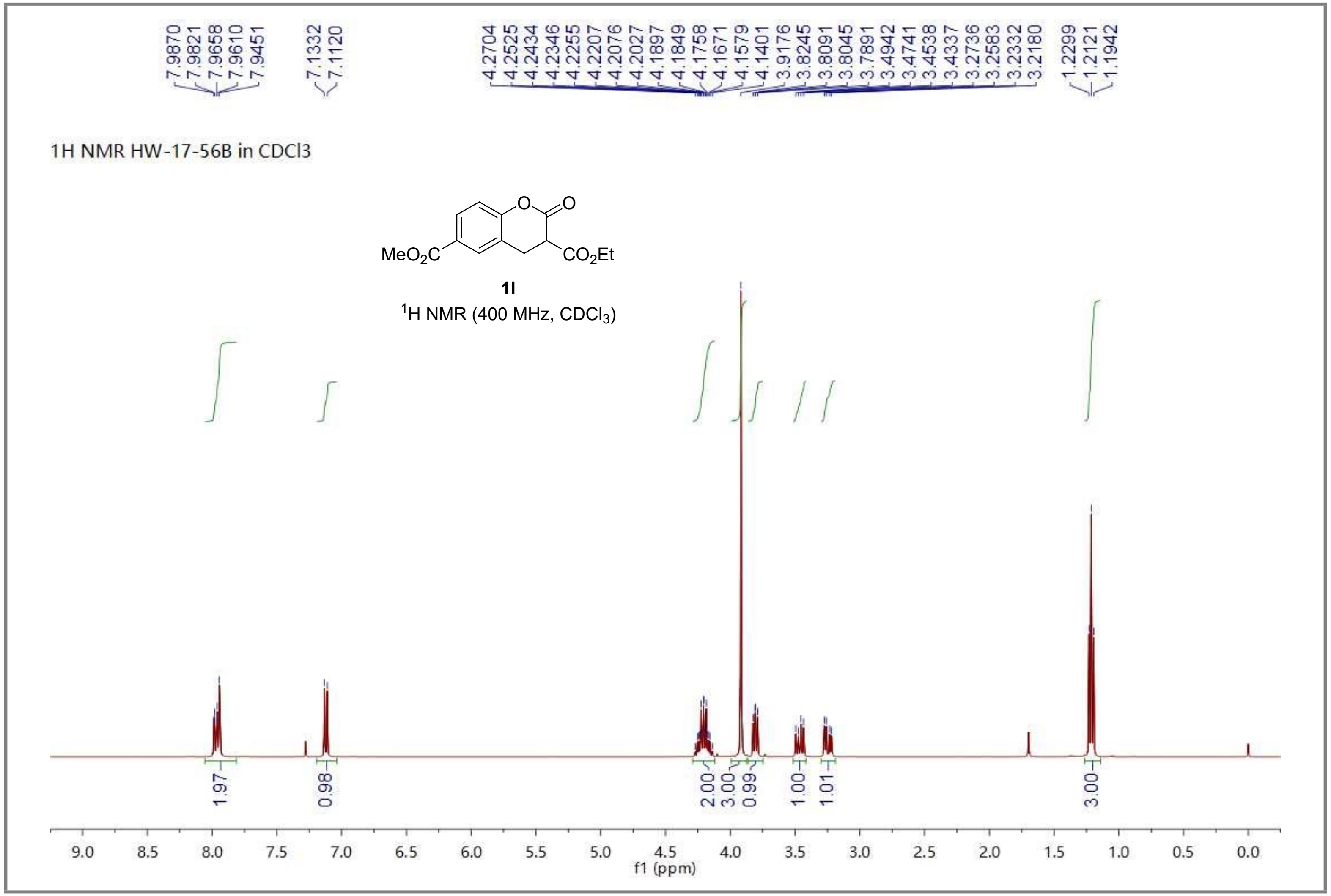

7.9870
7.9821
7.9658
7.9610
7.9451
7.1332
7.1120
4.2704
4.2525
4.2434
4.2346
4.2255
4.2207
4.2076
4.2027
4.1897
4.1849
4.1758
4.1671
4.1579
4.1401
3.9176
3.8245
3.8091
3.8045
3.7891
3.4942
3.4741
3.4538
3.4337
3.2736
3.2583
3.2332
3.2180
1.2299
1.2121
1.1942
1H NMR HW-17-56B in CDCl3
MeO2C
CO2Et
O
O
1l
1H NMR (400 MHz, CDCl3)
1.97
0.98
2.00
3.00
0.99
1.00
1.01
3.00
9.0
8.5
8.0
7.5
7.0
6.5
6.0
5.5
5.0
4.5
4.0
3.5
3.0
2.5
2.0
1.5
1.0
0.5
0.0
f1 (ppm)

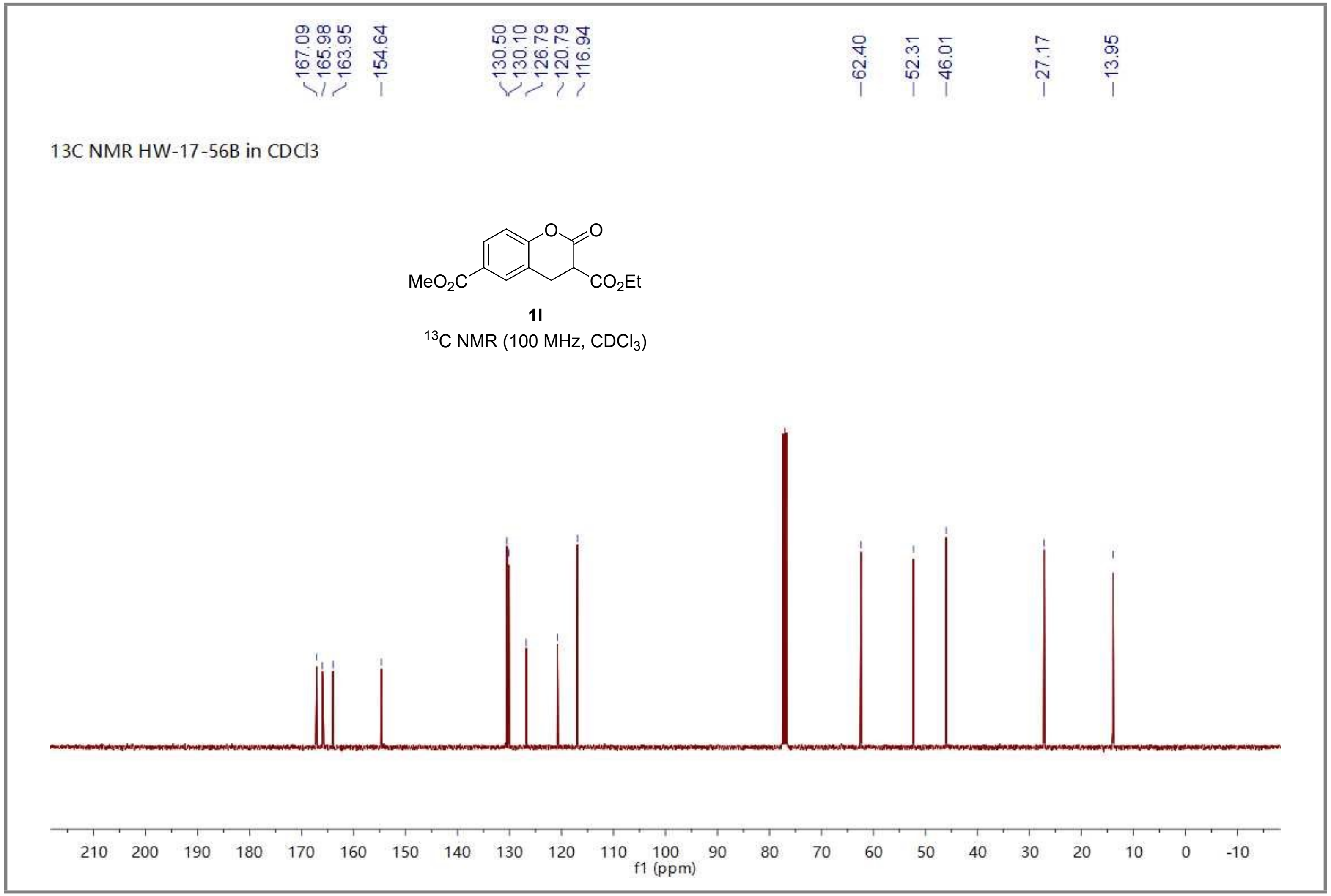

167.09
165.98
163.95
154.64
130.50
130.10
126.79
120.79
116.94
62.40
52.31
46.01
27.17
13.95
13C NMR HW-17-56B in CDCl3
MeO2C
CO2Et
O
O
1l
13C NMR (100 MHz, CDCl3)
210 200 190 180 170 160 150 140 130 120 110 100 90 80 70 60 50 40 30 20 10 0 -10
f1 (ppm)

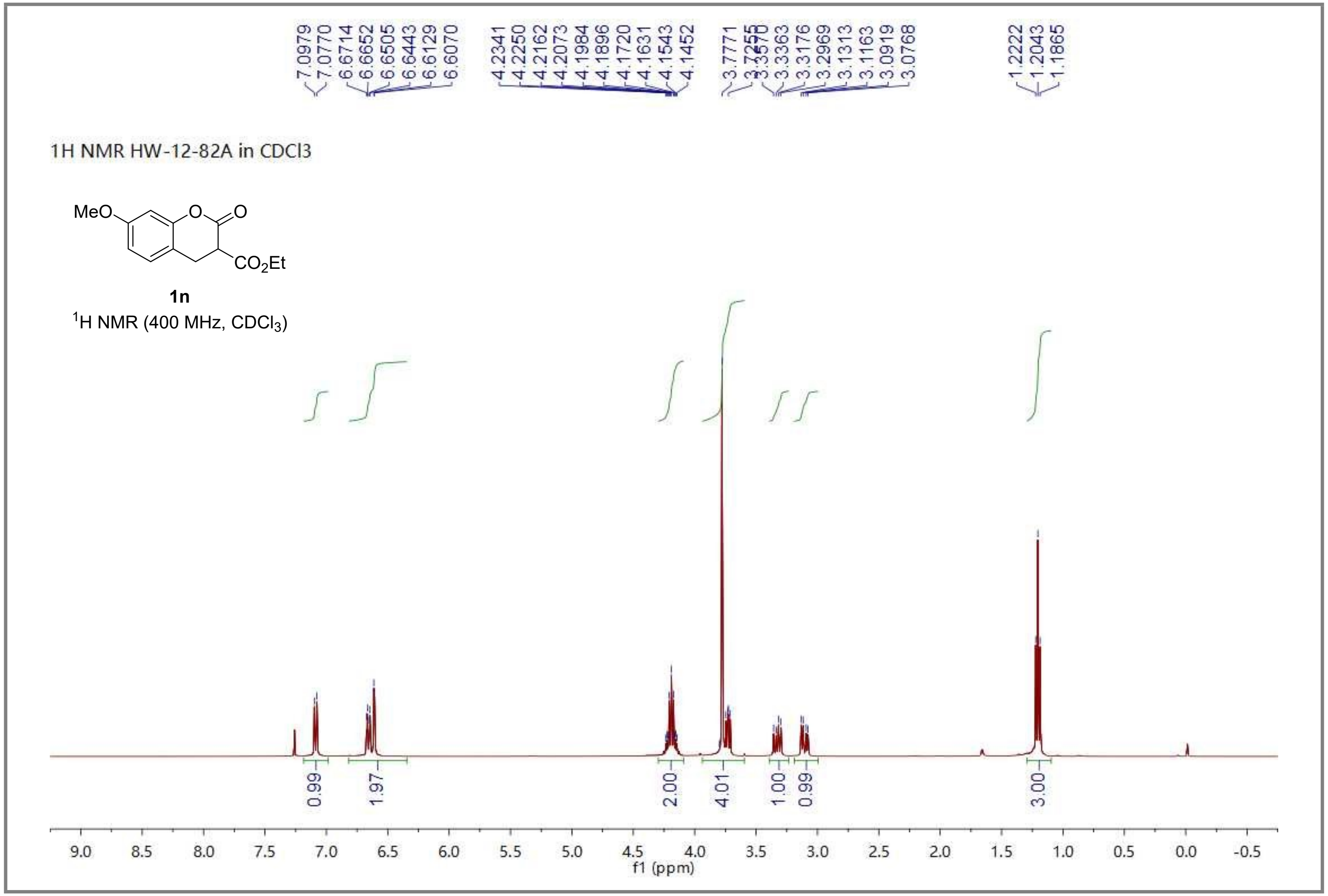
7.0979
7.0770
6.6714
6.6652
6.6505
6.6443
6.6129
6.6070
4.2341
4.2250
4.2162
4.2073
4.1984
4.1896
4.1720
4.1631
4.1543
4.1452
3.7771
3.7255
3.3570
3.3363
3.3176
3.2969
3.1313
3.1163
3.0919
3.0768
1.2222
1.2043
1.1865
1H NMR HW-12-82A in CDCl3
MeO
O
O
CO2Et
1n
1H NMR (400 MHz, CDCl3)
0.99
1.97
2.00
4.01
1.00
0.99
3.00
9.0
8.5
8.0
7.5
7.0
6.5
6.0
5.5
5.0
4.5
4.0
3.5
3.0
2.5
2.0
1.5
1.0
0.5
0.0
-0.5
f1 (ppm)

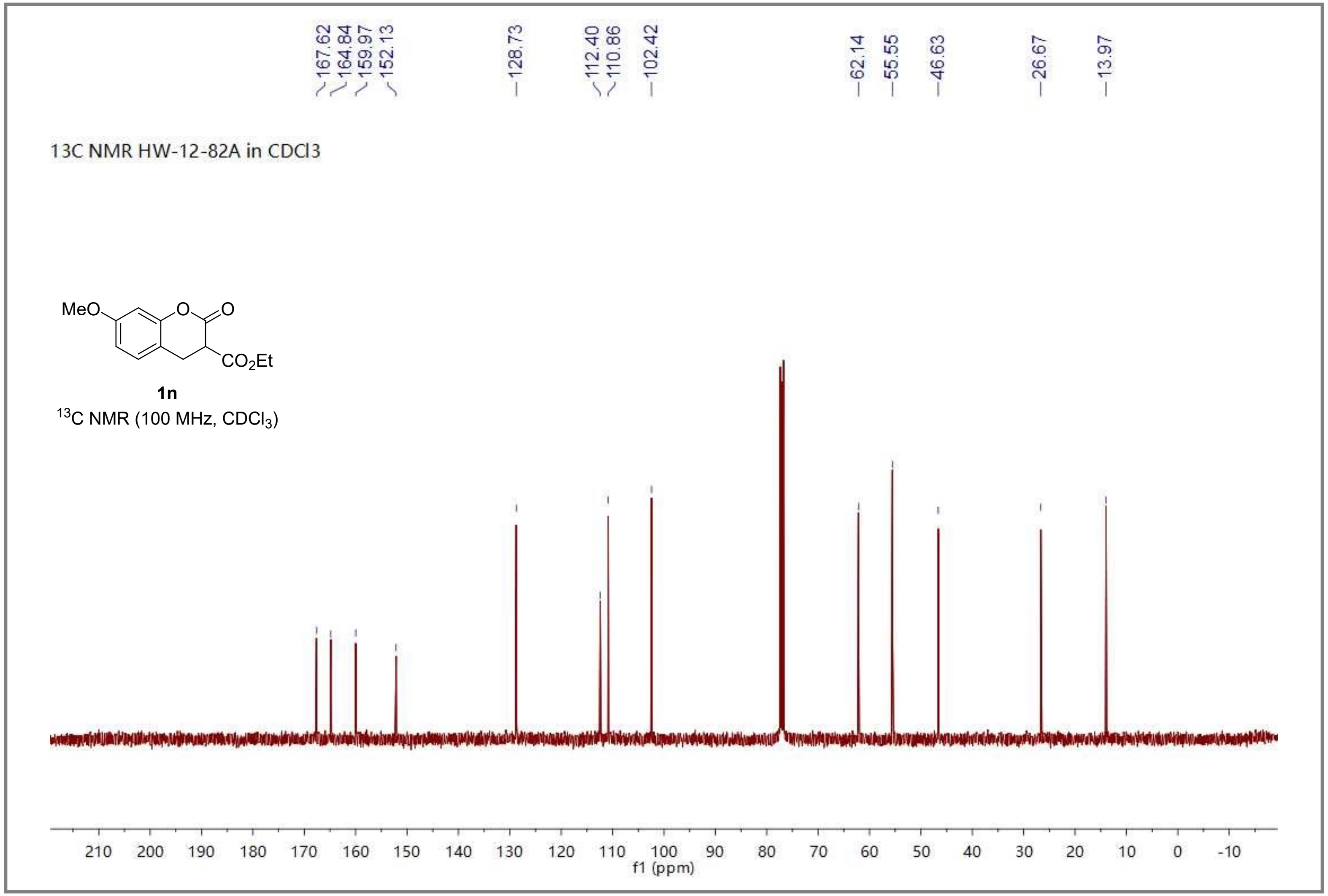

1n

^{13}C NMR (100 MHz, $CDCl_3$)

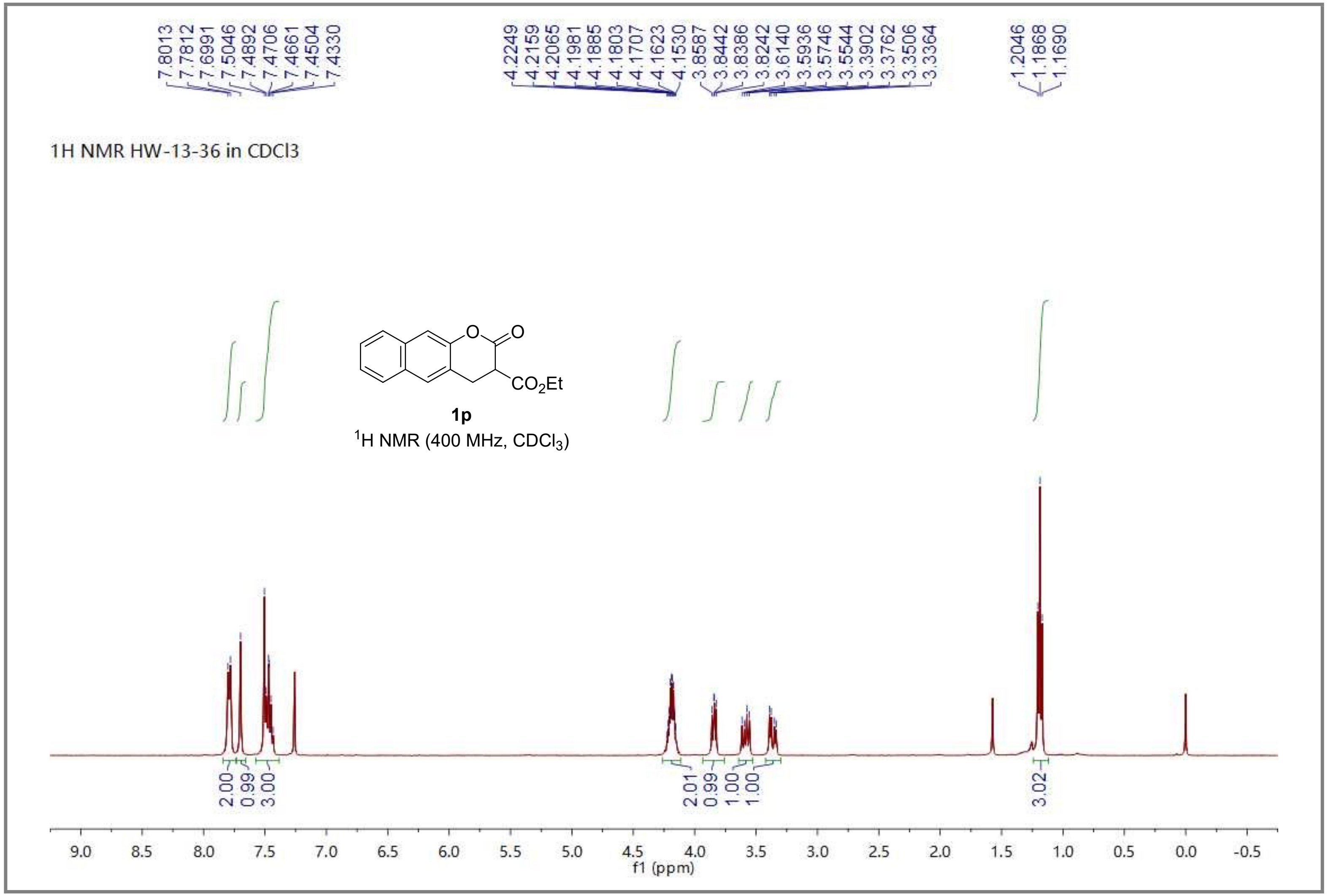
7.8013
7.7812
7.6991
7.5046
7.4892
7.4706
7.4661
7.4504
7.4330
4.2249
4.2159
4.2065
4.1981
4.1885
4.1803
4.1707
4.1623
4.1530
3.8587
3.8442
3.8386
3.8242
3.6140
3.5936
3.5746
3.5544
3.3902
3.3762
3.3506
3.3364
1.2046
1.1868
1.1690
1H NMR HW-13-36 in CDCl3
O
O
CO2Et
1p
1H NMR (400 MHz, CDCl3)
2.00
0.99
3.00
2.01
0.99
1.00
1.00
3.02
9.0
8.5
8.0
7.5
7.0
6.5
6.0
5.5
5.0
4.5
4.0
3.5
3.0
2.5
2.0
1.5
1.0
0.5
0.0
-0.5
f1 (ppm)

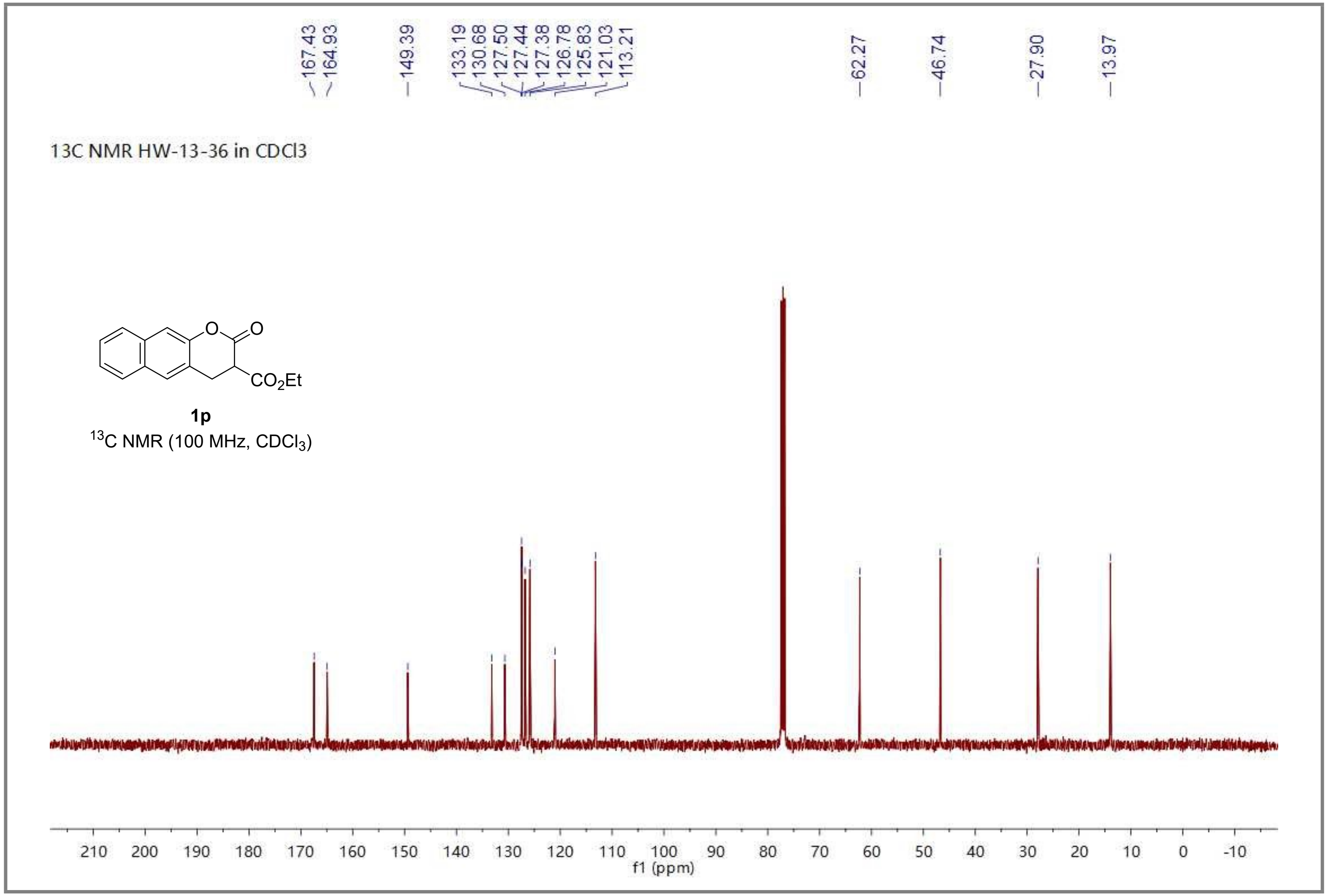

1p

^{13}C NMR (100 MHz, $CDCl_3$)

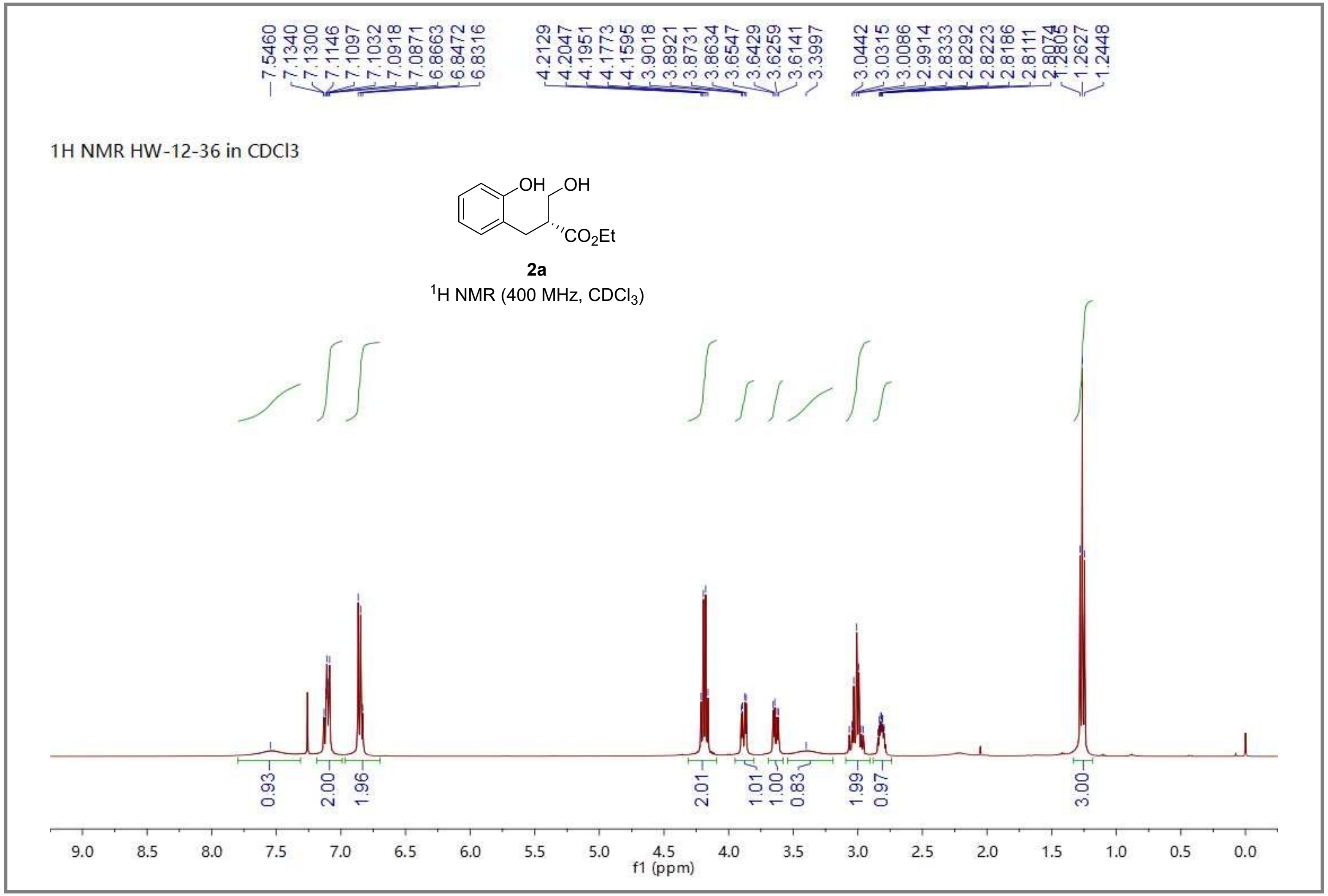

7.5460
7.1340
7.1300
7.1146
7.1097
7.1032
7.0918
7.0871
6.8663
6.8472
6.8316
4.2129
4.2047
4.1951
4.1773
4.1595
3.9018
3.8921
3.8731
3.8634
3.6547
3.6429
3.6259
3.6141
3.3997
3.0442
3.0315
3.0086
2.9914
2.8333
2.8292
2.8223
2.8186
2.8111
1.2627
1.2448
1H NMR HW-12-36 in CDCl3
OH
OH
CO2Et
2a
1H NMR (400 MHz, CDCl3)
0.93
2.00
1.96
2.01
1.01
1.00
0.83
1.99
0.97
3.00
9.0
8.5
8.0
7.5
7.0
6.5
6.0
5.5
5.0
4.5
4.0
3.5
3.0
2.5
2.0
1.5
1.0
0.5
0.0
f1 (ppm)

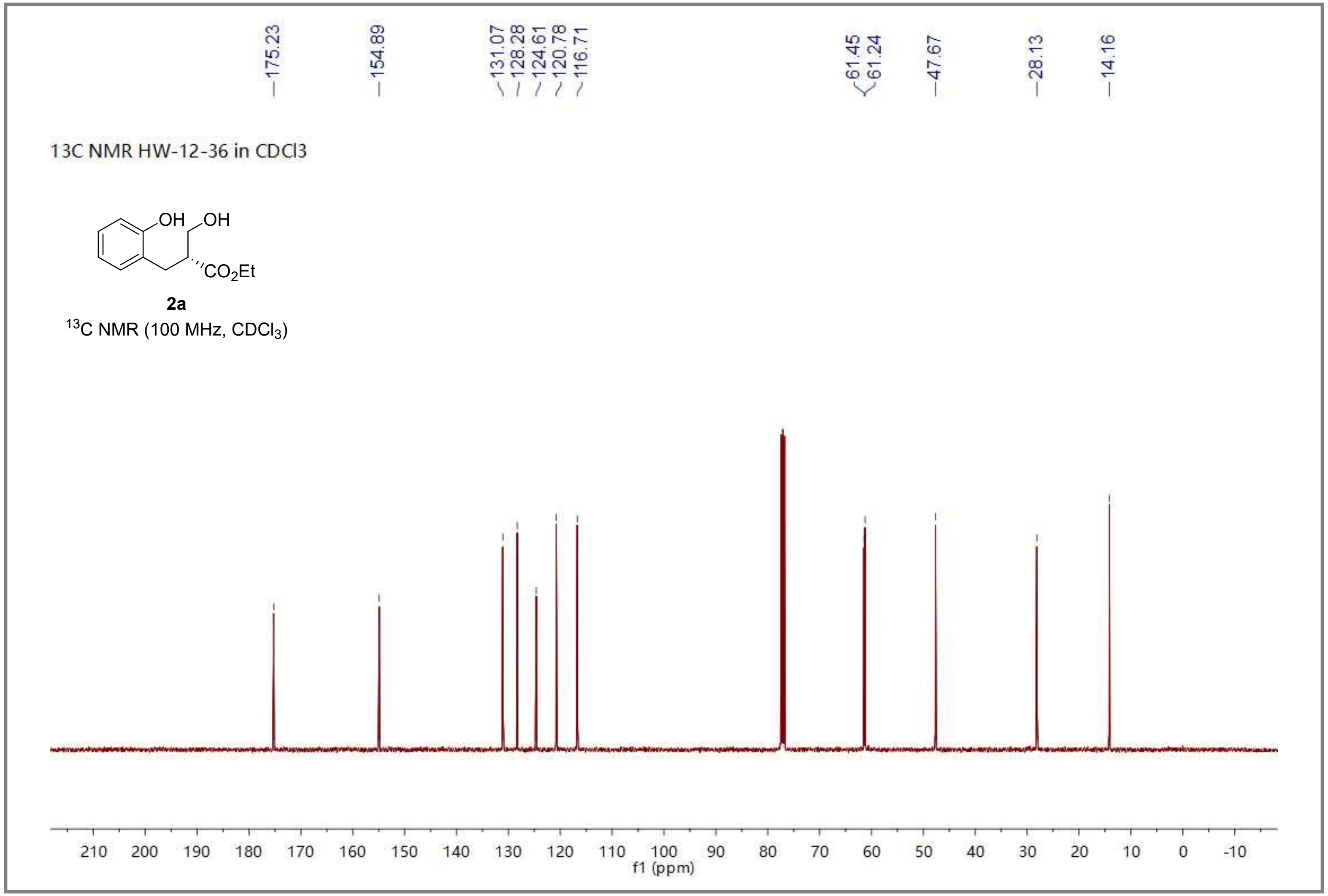
175.23
154.89
131.07
128.28
124.61
120.78
116.71
61.45
61.24
47.67
28.13
14.16
13C NMR HW-12-36 in CDCl3
OH
OH
CO2Et
2a
13C NMR (100 MHz, CDCl3)
210 200 190 180 170 160 150 140 130 120 110 100 90 80 70 60 50 40 30 20 10 0 -10
f1 (ppm)

Data File C:\USERS\PUBLIC\DOCUMENTS\CHEMSTATION\1\DATA\2021\YZH001126 19-54-53.D
Sample Name: HW-10-27+/-

===

Acq. Operator : SYSTEM
Sample Operator : SYSTEM
Acq. Instrument : HPLC1260 II Location : 1
Injection Date : 12/23/2021 7:54:53 PM Inj : 1
Inj Volume : No inj
Acq. Method : C:\USERS\PUBLIC\DOCUMENTS\CHEMSTATION\1\METHODS\def_LC1.M
Last changed : 12/23/2021 7:19:09 PM by SYSTEM
(modified after loading)
Analysis Method : C:\USERS\PUBLIC\DOCUMENTS\CHEMSTATION\1\METHODS\def_LC1.M
Last changed : 10/17/2023 10:24:47 AM by SYSTEM
(modified after loading)
Sample Info : AS-H, Hexane/i-PrOH = 90/10, 0.8 mL/min, 30 oC, 230 nm

Additional Info : Peak(s) manually integrated

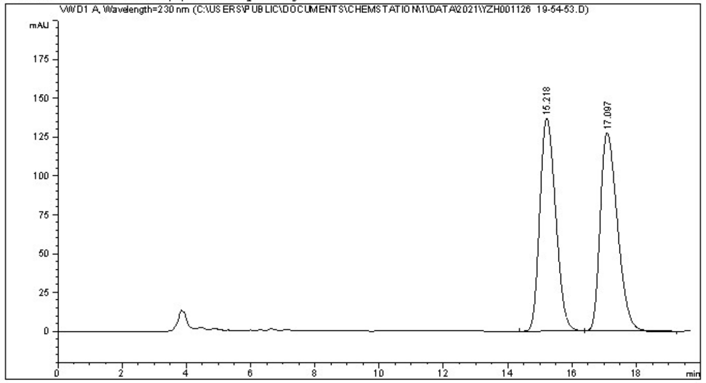

===
Area Percent Report
===

Sorted By : Signal
Multiplier : 1.0000
Dilution : 1.0000
Do not use Multiplier & Dilution Factor with ISTDs

Signal 1: VWD1 A, Wavelength=230 nm

Peak #	RetTime [min]	Type	Width [min]	Area [mAU*s]	Height [mAU]	Area %
1	15.218	BB	0.5413	4693.18555	137.10939	49.8009
2	17.097	BB	0.5803	4730.71143	127.37619	50.1991

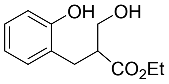

(+/-)-2a

HPLC1260 II 10/17/2023 10:24:52 AM SYSTEM

Page 1 of 2

Data File C:\USERS\PUBLIC\DOCUMENTS\CHEMSTATION\1\DATA\2022\YZH002566 20-01-01.D
Sample Name: HW-12-36

===

Acq. Operator : SYSTEM
Sample Operator : SYSTEM
Acq. Instrument : HPLC1260 II Location : 1
Injection Date : 10/21/2022 8:01:02 PM Inj : 1
Inj Volume : No inj
Acq. Method : C:\USERS\PUBLIC\DOCUMENTS\CHEMSTATION\1\METHODS\def_LC1.M
Last changed : 10/21/2022 7:49:42 PM by SYSTEM
(modified after loading)
Analysis Method : C:\USERS\PUBLIC\DOCUMENTS\CHEMSTATION\1\METHODS\def_LC1.M
Last changed : 10/17/2023 10:26:34 AM by SYSTEM
(modified after loading)
Sample Info : AS-H, Hexane/i-PrOH = 90/10, 0.8 mL/min, 30 oC, 230 nm

Additional Info : Peak(s) manually integrated

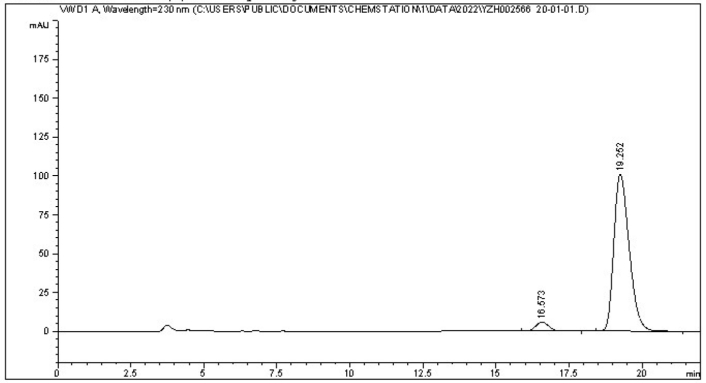

===
Area Percent Report
===

Sorted By : Signal
Multiplier : 1.0000
Dilution : 1.0000
Do not use Multiplier & Dilution Factor with ISTDs

Signal 1: VWD1 A, Wavelength=230 nm

Peak #	RetTime [min]	Type	Width [min]	Area [mAU*s]	Height [mAU]	Area %
1	16.573	BB	0.4750	181.65741	5.90364	4.7155
2	19.252	BB	0.5613	3670.72803	101.16671	95.2845

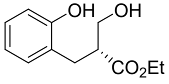

(+)-2a

HPLC1260 II 10/17/2023 10:26:41 AM SYSTEM

Page 1 of 2

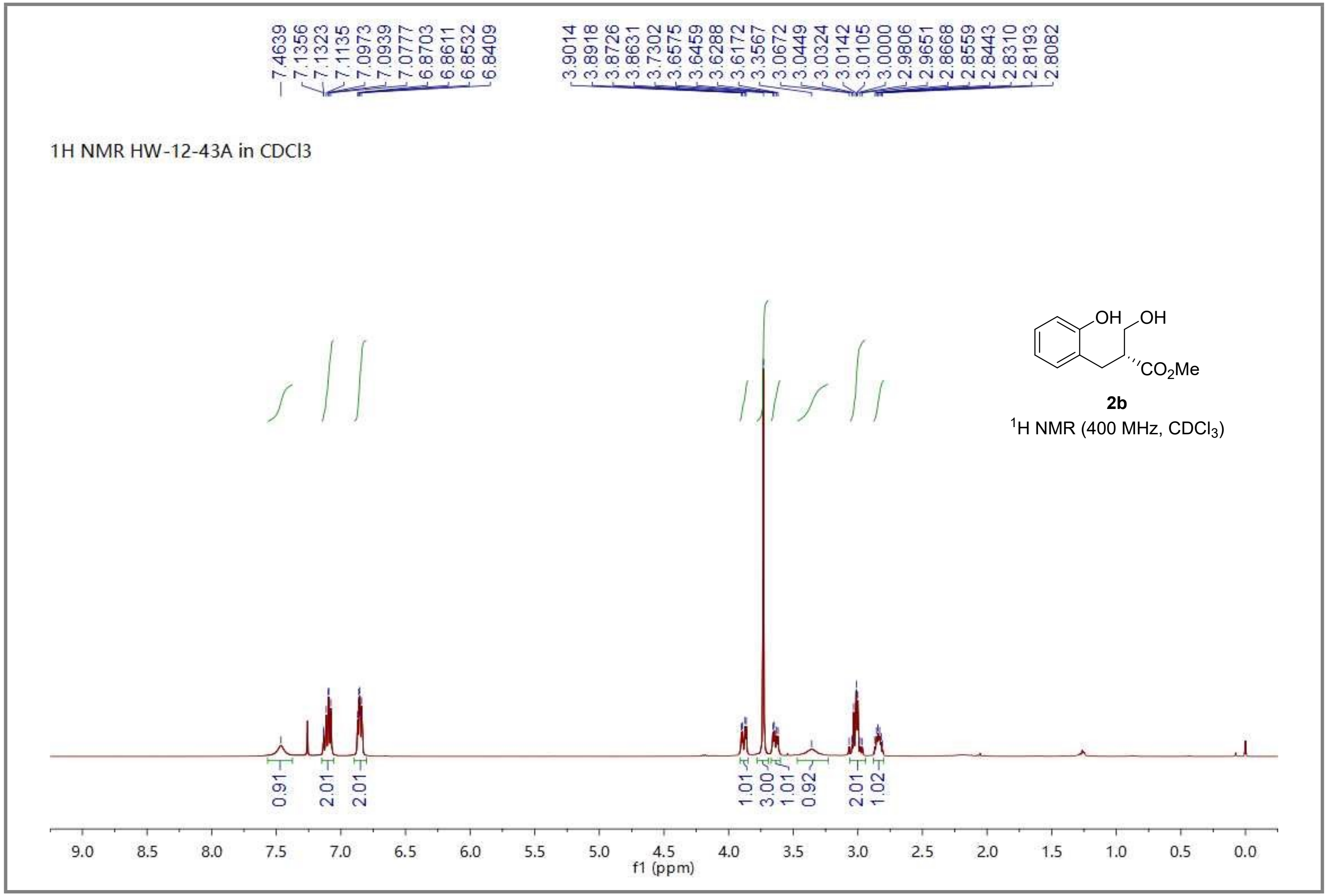

2b

^{1}H NMR (400 MHz, $CDCl_3$)

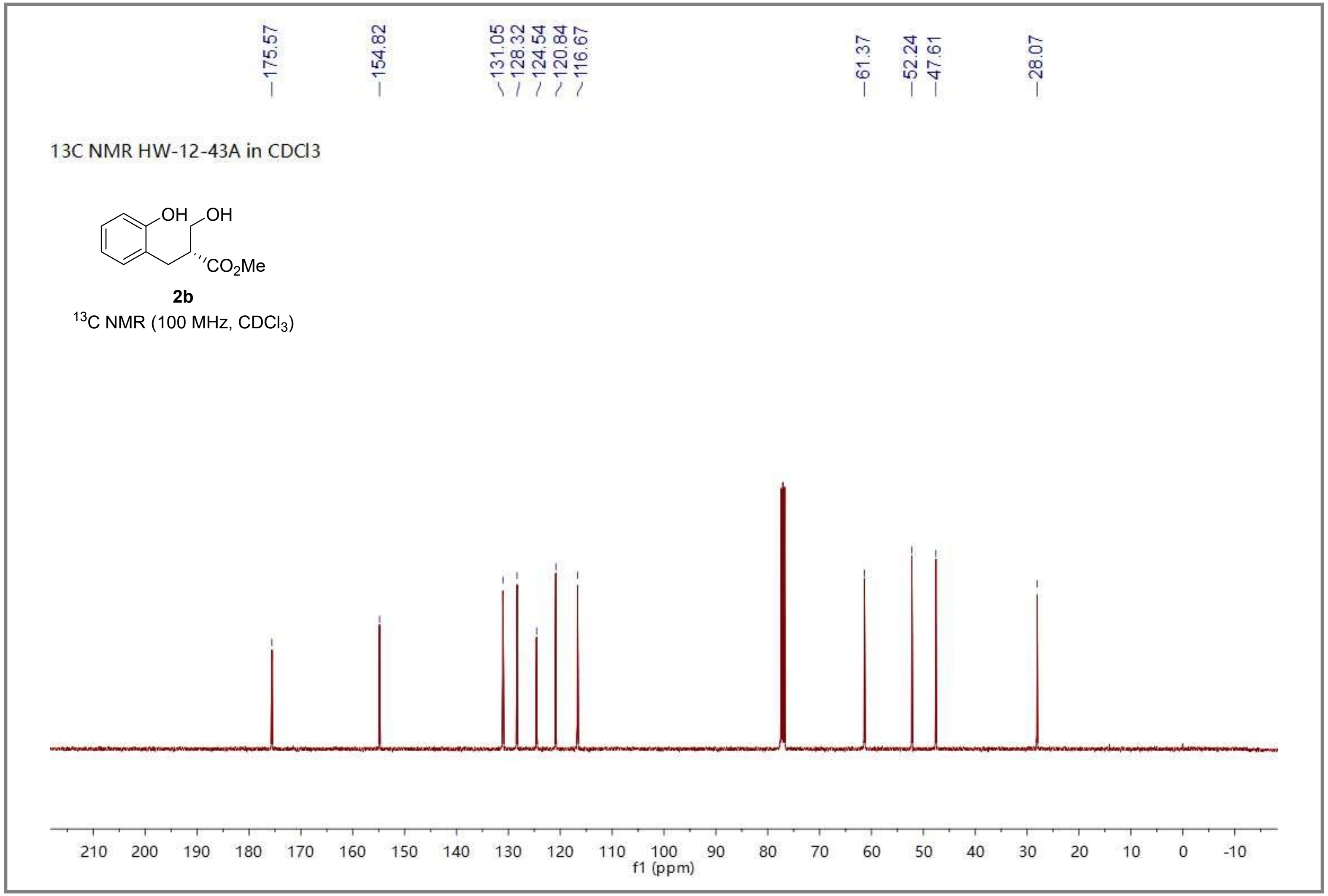

175.57
154.82
131.05
128.32
124.54
120.84
116.67
61.37
52.24
47.61
28.07
13C NMR HW-12-43A in CDCl3
OH
OH
CO2Me
2b
13C NMR (100 MHz, CDCl3)
210 200 190 180 170 160 150 140 130 120 110 100 90 80 70 60 50 40 30 20 10 0 -10
f1 (ppm)

Data File C:\USERS\PUBLIC\DOCUMENTS\CHEMSTATION\1\DATA\2022\YZH002669 10-08-05.D
Sample Name: HW-12-43A+/-

===

Acq. Operator : SYSTEM
Sample Operator : SYSTEM
Acq. Instrument : HPLC1260 II Location : 1
Injection Date : 10/31/2022 10:08:06 AM Inj : 1
Inj Volume : No inj
Acq. Method : C:\USERS\PUBLIC\DOCUMENTS\CHEMSTATION\1\METHODS\def_LC1.M
Last changed : 10/31/2022 9:54:39 AM by SYSTEM
(modified after loading)
Analysis Method : C:\USERS\PUBLIC\DOCUMENTS\CHEMSTATION\1\METHODS\def_LC1.M
Last changed : 10/17/2023 10:28:02 AM by SYSTEM
(modified after loading)
Sample Info : IB, Hexane/i-PrOH = 90/10, 0.8 mL/min, 30 oC, 230 nm

Additional Info : Peak(s) manually integrated

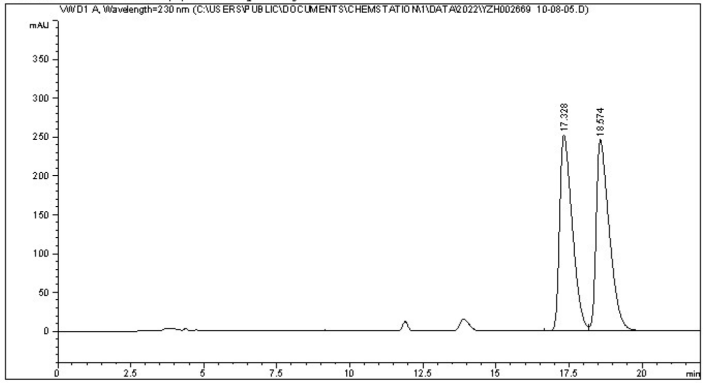

===

Area Percent Report

===

Sorted By : Signal
Multiplier : 1.0000
Dilution : 1.0000
Do not use Multiplier & Dilution Factor with ISTDs

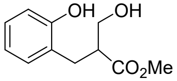

(+/-)-2b

Signal 1: VWD1 A, Wavelength=230 nm

Peak #	RetTime [min]	Type	Width [min]	Area [mAU*s]	Height [mAU]	Area %
1	17.328	BV	0.4606	7565.42383	252.47827	49.4359
2	18.574	VB	0.4692	7738.06689	246.54932	50.5641

HPLC1260 II 10/17/2023 10:28:06 AM SYSTEM

Page 1 of 2

Data File C:\USERS\PUBLIC\DOCUMENTS\CHEMSTATION\1\DATA\2022\YZH002670 10-40-03.D
Sample Name: HW-12-43A

===

Acq. Operator : SYSTEM
Sample Operator : SYSTEM
Acq. Instrument : HPLC1260 II Location : 1
Injection Date : 10/31/2022 10:40:03 AM Inj : 1
Inj Volume : No inj
Acq. Method : C:\USERS\PUBLIC\DOCUMENTS\CHEMSTATION\1\METHODS\def_LC1.M
Last changed : 10/31/2022 9:54:39 AM by SYSTEM
(modified after loading)
Analysis Method : C:\USERS\PUBLIC\DOCUMENTS\CHEMSTATION\1\METHODS\def_LC1.M
Last changed : 10/17/2023 10:28:02 AM by SYSTEM
(modified after loading)
Sample Info : IB, Hexane/i-PrOH = 90/10, 0.8 mL/min, 30 oC, 230 nm

Additional Info : Peak(s) manually integrated

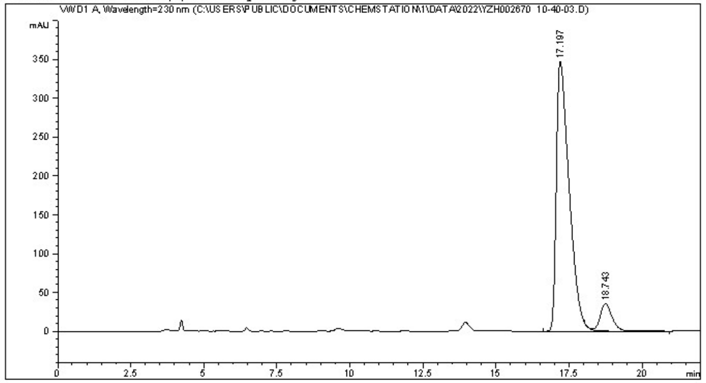

===

Area Percent Report

===

Sorted By : Signal
Multiplier : 1.0000
Dilution : 1.0000
Do not use Multiplier & Dilution Factor with ISTDs

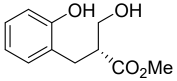

(+)-2b

Signal 1: VWD1 A, Wavelength=230 nm

Peak #	RetTime [min]	Type	Width [min]	Area [mAU*s]	Height [mAU]	Area %
1	17.197	BV R	0.4631	1.06331e4	347.43600	91.3184
2	18.743	VB E	0.4324	1010.87579	35.37531	8.6816

HPLC1260 II 10/17/2023 10:28:49 AM SYSTEM

Page 1 of 2

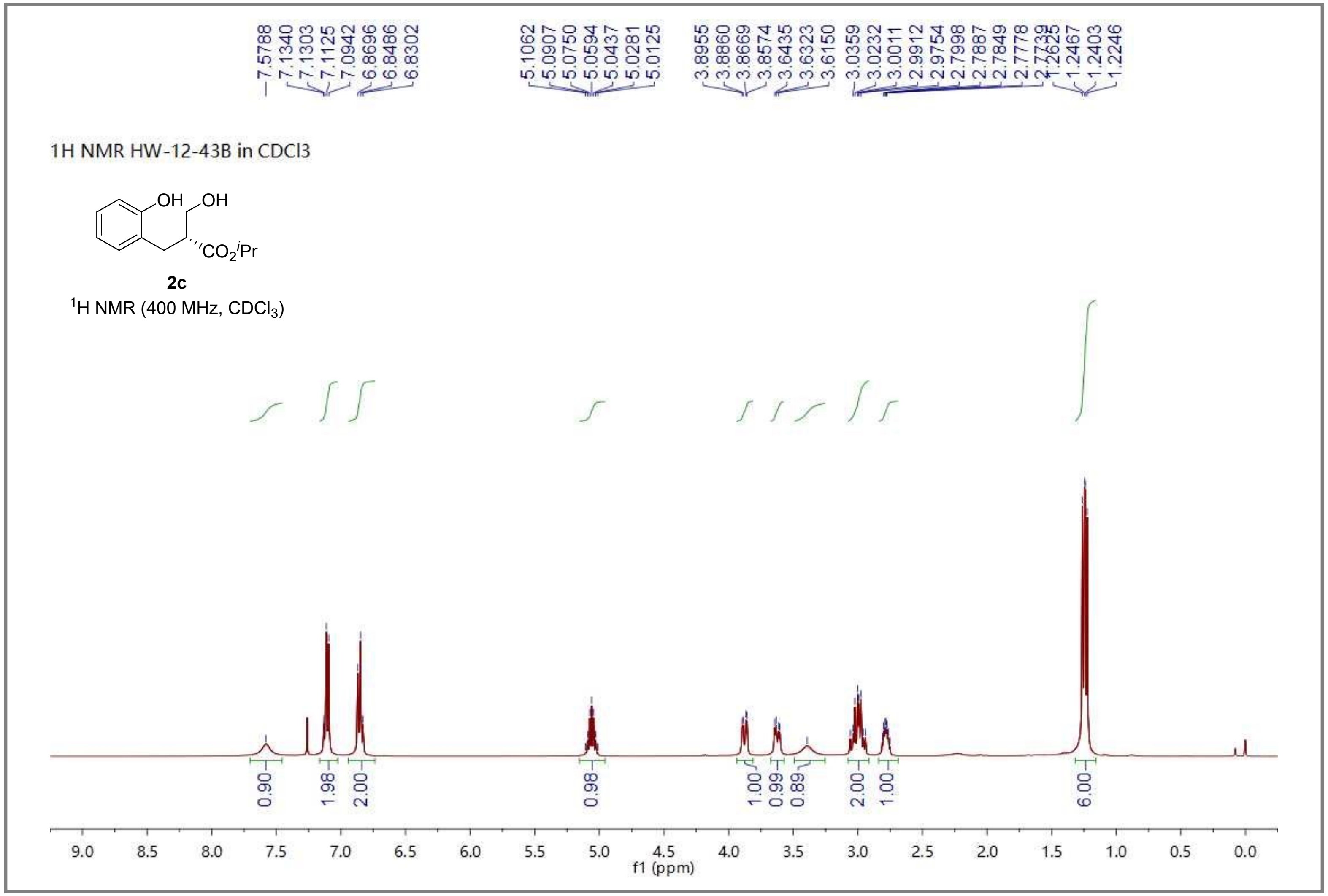
7.5788
7.1340
7.1303
7.1125
7.0942
6.8696
6.8486
6.8302
5.1062
5.0907
5.0750
5.0594
5.0437
5.0281
5.0125
3.8955
3.8860
3.8669
3.8574
3.6435
3.6323
3.6150
3.0359
3.0232
3.0011
2.9912
2.9754
2.7998
2.7887
2.7849
2.7778
2.7739
1.2625
1.2467
1.2403
1.2246
1H NMR HW-12-43B in CDCl3
OH
OH
CO2iPr
2c
1H NMR (400 MHz, CDCl3)
0.90
1.98
2.00
0.98
1.00
0.99
0.89
2.00
1.00
6.00
9.0
8.5
8.0
7.5
7.0
6.5
6.0
5.5
5.0
4.5
4.0
3.5
3.0
2.5
2.0
1.5
1.0
0.5
0.0
f1 (ppm)

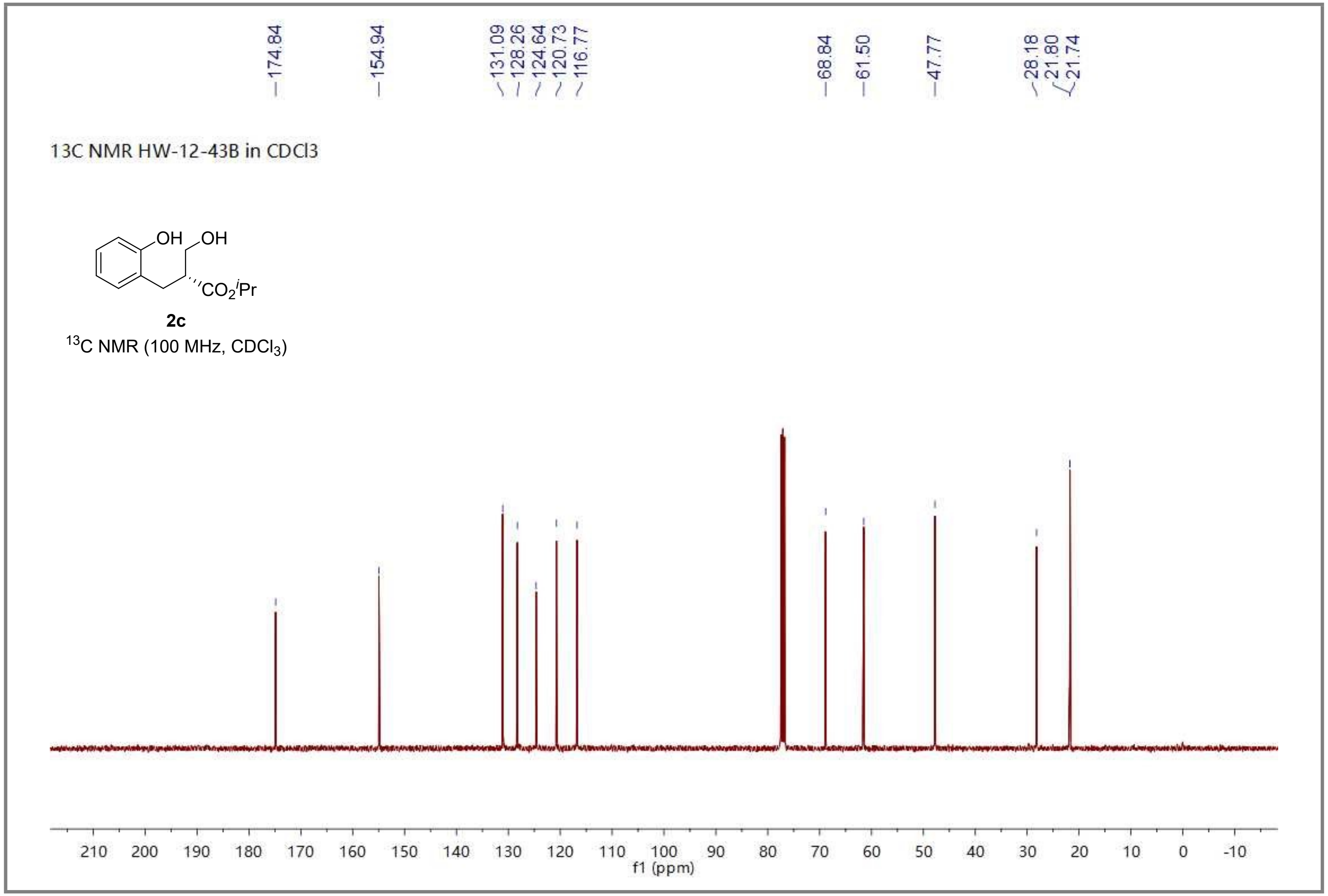
174.84
154.94
131.09
128.26
124.64
120.73
116.77
68.84
61.50
47.77
28.18
21.80
21.74
13C NMR HW-12-43B in CDCl3
OH
OH
CO2iPr
2c
13C NMR (100 MHz, CDCl3)
210 200 190 180 170 160 150 140 130 120 110 100 90 80 70 60 50 40 30 20 10 0 -10
f1 (ppm)

```
Data File C:\CHEM32\1\DATA\ZHOU-22\YZN021922.D
Sample Name: HW-12-43B+/-

=====================================================================
Acq. Operator   :
Acq. Instrument : Instrument 1                    Location :   -
Injection Date  : 10/30/2022 10:20:46 PM
Acq. Method     : C:\CHEM32\1\METHODS\DEF_LC.M
Last changed    : 10/30/2022 10:20:15 PM
                  (modified after loading)
Analysis Method : C:\CHEM32\1\METHODS\DEF_LC.M
Last changed    : 10/17/2023 10:18:18 PM
                  (modified after loading)
Sample Info     : AS-H, Hexane/i-PrOH = 90/10, 0.8 mL/min, 30 oC, 230 nm
```

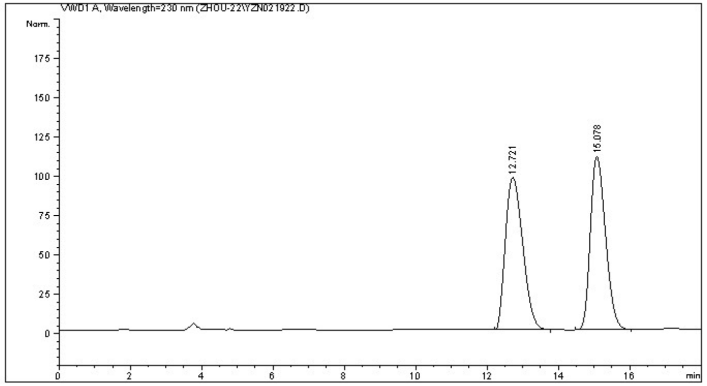


```
=====================================================================
                         Area Percent Report
=====================================================================

Sorted By             :      Signal
Multiplier:                  :      1.0000
Dilution:                    :      1.0000
Use Multiplier & Dilution Factor with ISTDs


Signal 1: VWD1 A, Wavelength=230 nm

Peak RetTime Type  Width     Area      Height     Area
  #   [min]        [min]  mAU   *s    [mAU  ]       %
----|-------|----|-------|----------|----------|--------|
   1  12.721 BB    0.5415 3272.87427   96.79517  50.1478
   2  15.078 BB    0.4621 3253.57910  110.28246  49.8522

Totals :                  6526.45337  207.07764

=====================================================================
                         *** End of Report ***
```

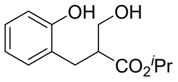

(+/-)-2c

Instrument 1 10/17/2023 10:18:28 PM   Page 1 of 1

```
Data File C:\CHEM32\1\DATA\ZHOU-22\YZN021921.D
Sample Name: HW-12-43B

=====================================================================
Acq. Operator   :
Acq. Instrument : Instrument 1                    Location :   -
Injection Date  : 10/30/2022 9:58:49 PM
Acq. Method     : C:\CHEM32\1\METHODS\DEF_LC.M
Last changed    : 10/30/2022 9:47:11 PM
                  (modified after loading)
Analysis Method : C:\CHEM32\1\METHODS\DEF_LC.M
Last changed    : 10/17/2023 10:20:10 PM
                  (modified after loading)
Sample Info     : AS-H, Hexane/i-PrOH = 90/10, 0.8 mL/min, 30 oC, 230 nm
```

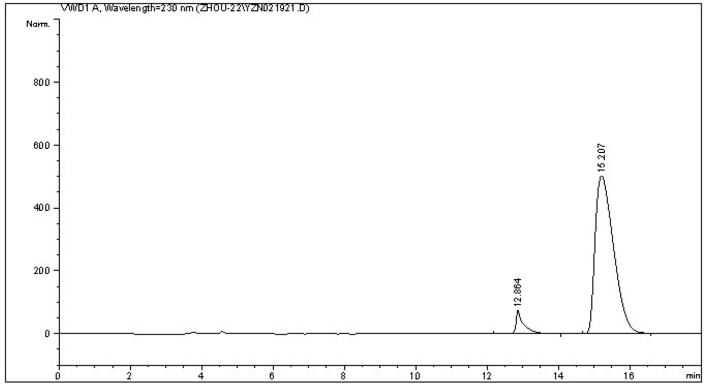


```
=====================================================================
                         Area Percent Report
=====================================================================

Sorted By             :      Signal
Multiplier:                  :      1.0000
Dilution:                    :      1.0000
Use Multiplier & Dilution Factor with ISTDs


Signal 1: VWD1 A, Wavelength=230 nm

Peak RetTime Type  Width     Area      Height     Area
  #   [min]        [min]  mAU   *s    [mAU  ]       %
----|-------|----|-------|----------|----------|--------|
   1  12.864 BB    0.1941 1091.21497   73.91610   5.7253
   2  15.207 BB    0.5657 1.79683e4   502.47910  94.2747

Totals :                  1.90595e4   576.39520

=====================================================================
                         *** End of Report ***
```

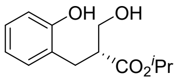

(+)-2c

Instrument 1 10/17/2023 10:20:13 PM   Page 1 of 1

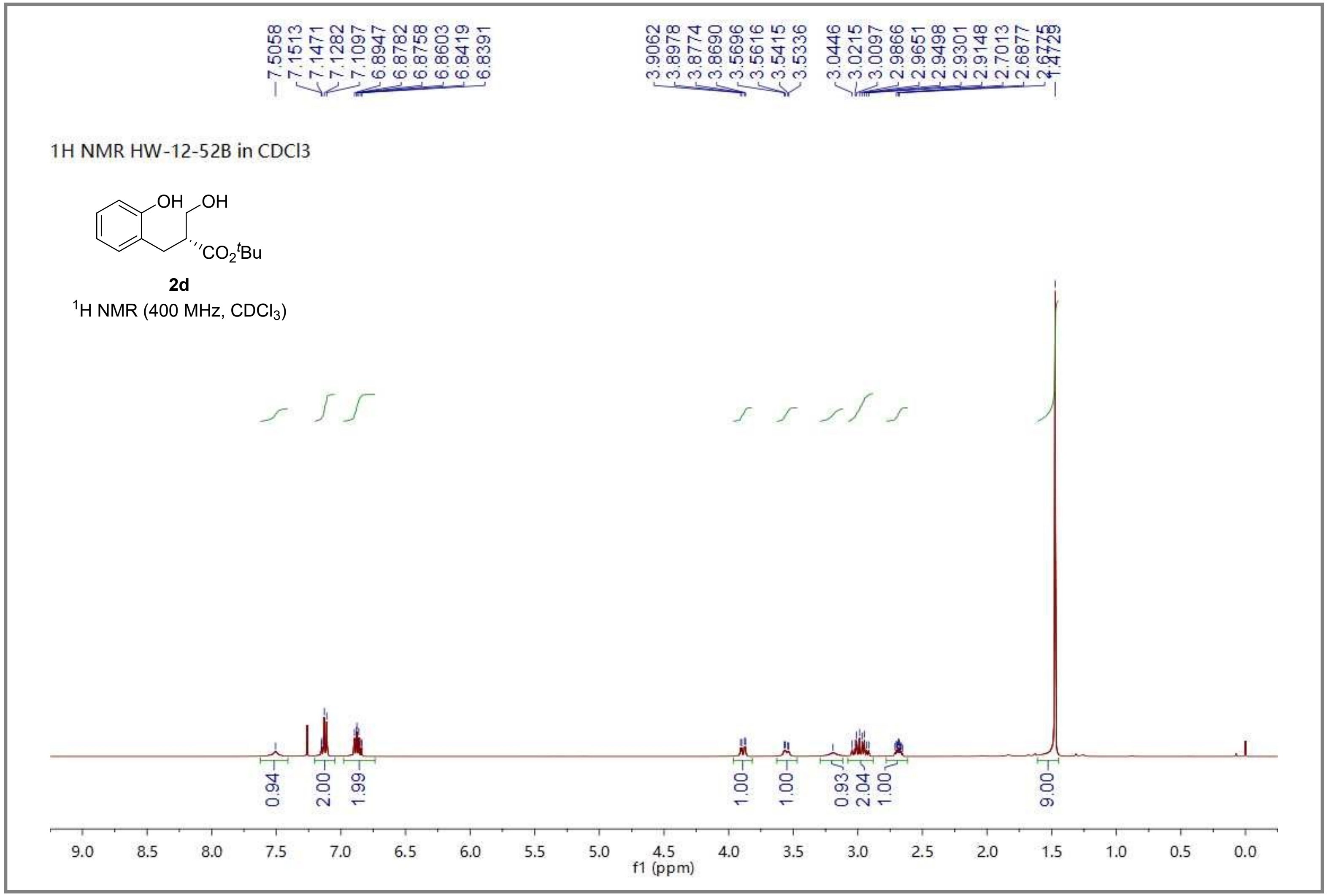

7.5058
7.1513
7.1471
7.1282
7.1097
6.8947
6.8782
6.8758
6.8603
6.8419
6.8391
3.9062
3.8978
3.8774
3.8690
3.5696
3.5616
3.5415
3.5336
3.0446
3.0215
3.0097
2.9866
2.9651
2.9498
2.9301
2.9148
2.7013
2.6877
2.6775
1.4729
1H NMR HW-12-52B in CDCl3
OH
OH
CO2tBu
2d
1H NMR (400 MHz, CDCl3)
0.94
2.00
1.99
1.00
1.00
0.93
2.04
1.00
9.00
9.0
8.5
8.0
7.5
7.0
6.5
6.0
5.5
5.0
4.5
4.0
3.5
3.0
2.5
2.0
1.5
1.0
0.5
0.0
f1 (ppm)

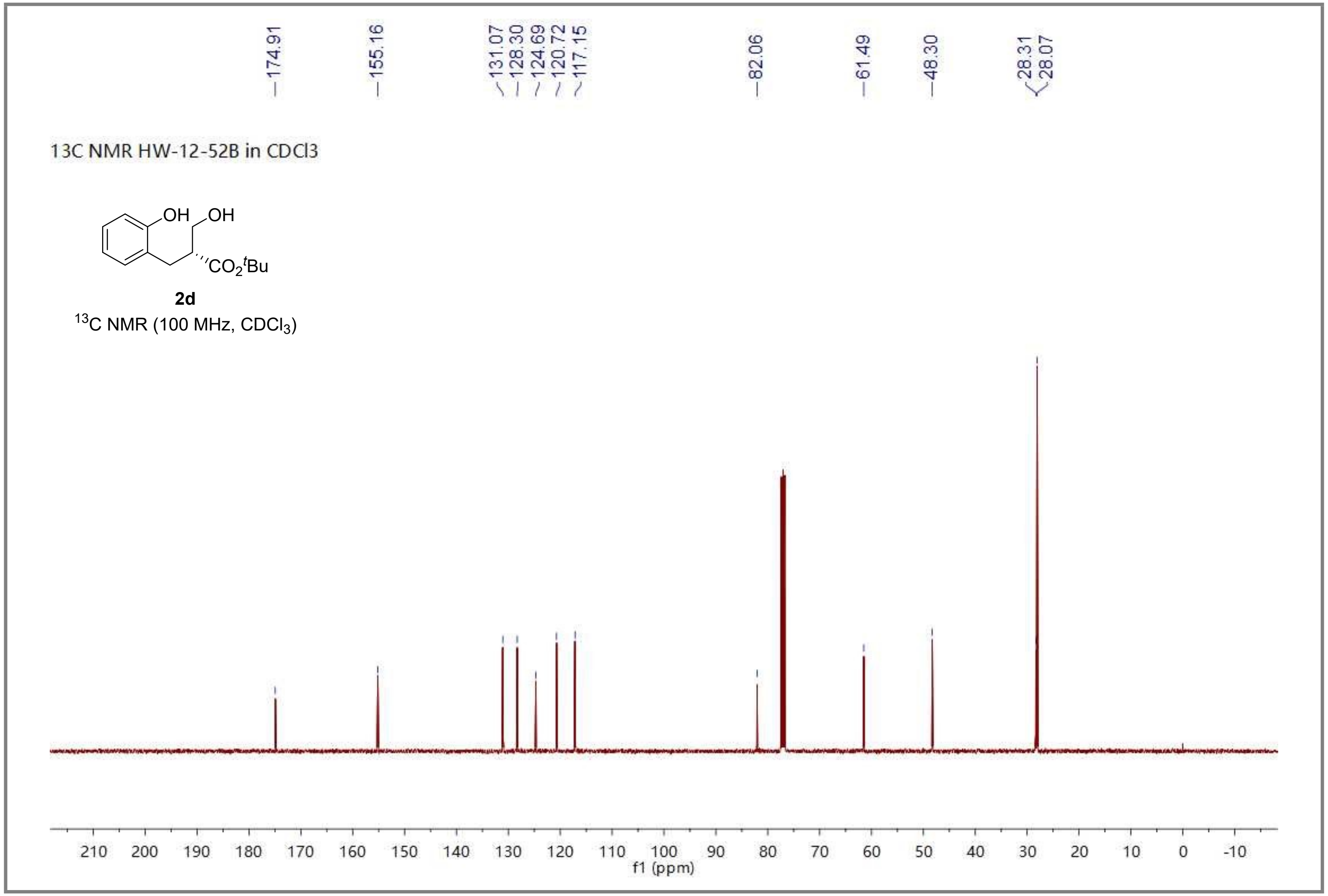

174.91
155.16
131.07
128.30
124.69
120.72
117.15
82.06
61.49
48.30
28.31
28.07
13C NMR HW-12-52B in CDCl3
OH
OH
CO2tBu
2d
13C NMR (100 MHz, CDCl3)
210 200 190 180 170 160 150 140 130 120 110 100 90 80 70 60 50 40 30 20 10 0 -10
f1 (ppm)

```
Data File D:\USERS\CHEMSTATION\1\DATA\ZHOU2022\YZ3005557  15-16-34.D
Sample Name: HW-12-52B+/-

    =====================================================================
    Acq. Operator   : SYSTEM
    Sample Operator : SYSTEM
    Acq. Instrument : 1260II                          Location :     -
    Injection Date  : 11/3/2022 3:16:34 PM                 Inj :   1
                                                    Inj Volume : No inj
    Acq. Method     : C:\USERS\PUBLIC\DOCUMENTS\CHEMSTATION\1\METHODS\lsm-1-58.M
    Last changed    : 11/3/2022 3:15:39 PM by SYSTEM
                      (modified after loading)
    Analysis Method : C:\Users\Public\Documents\ChemStation\1\Methods\def_LC.M
    Last changed    : 10/17/2023 10:47:36 AM by SYSTEM
                      (modified after loading)
    Sample Info     : AS-H, n-Hexane/i-PrOH = 95/5, 0.8 mL/min, 30 oC, 230 nm

    Additional Info : Peak(s) manually integrated
```

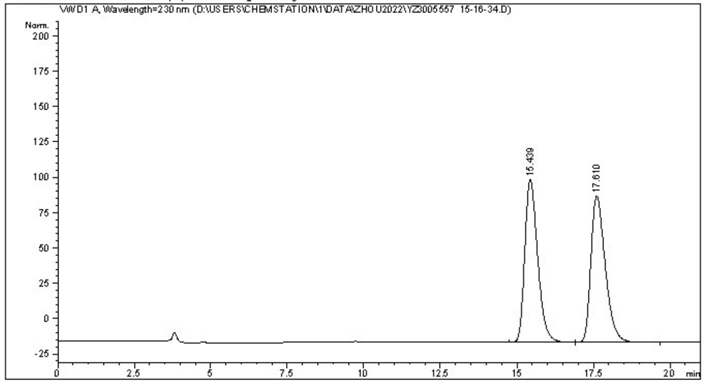


```
    =====================================================================
                          Area Percent Report
    =====================================================================

    Sorted By             :      Signal
    Multiplier            :      1.0000
    Dilution              :      1.0000
    Do not use Multiplier & Dilution Factor with ISTDs

    Signal 1: VWD1 A, Wavelength=230 nm
```

Peak #	RetTime [min]	Type	Width [min]	Area [mAU*s]	Height [mAU]	Area %
1	15.439	BB	0.4512	2422.44336	82.83044	49.9052
2	17.610	BB	0.5026	2431.65088	74.54190	50.0948

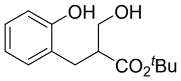

(+/-)-2d

```
1260II 10/17/2023 10:47:38 AM SYSTEM                                Page 1 of 2
```

```
Data File D:\USERS\CHEMSTATION\1\DATA\ZHOU2022\YZ3005558  15-59-50.D
Sample Name: HW-12-52B

    =====================================================================
    Acq. Operator   : SYSTEM
    Sample Operator : SYSTEM
    Acq. Instrument : 1260II                          Location :     -
    Injection Date  : 11/3/2022 3:59:50 PM                 Inj :   1
                                                    Inj Volume : No inj
    Acq. Method     : C:\USERS\PUBLIC\DOCUMENTS\CHEMSTATION\1\METHODS\lsm-1-58.M
    Last changed    : 11/3/2022 3:59:23 PM by SYSTEM
                      (modified after loading)
    Analysis Method : C:\Users\Public\Documents\ChemStation\1\Methods\def_LC.M
    Last changed    : 10/17/2023 10:48:28 AM by SYSTEM
                      (modified after loading)
    Sample Info     : AS-H, n-Hexane/i-PrOH = 95/5, 0.8 mL/min, 30 oC, 230 nm

    Additional Info : Peak(s) manually integrated
```

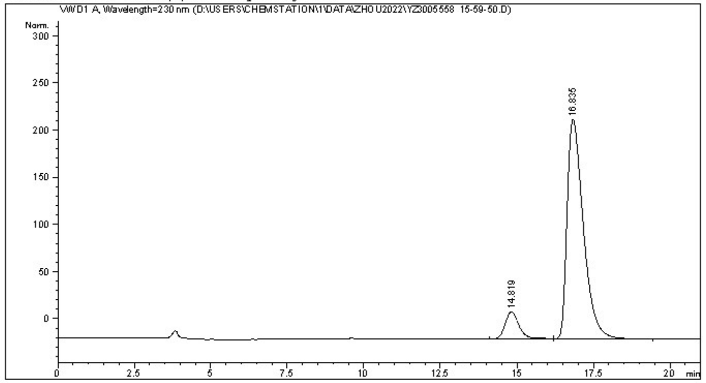


```
    =====================================================================
                          Area Percent Report
    =====================================================================

    Sorted By             :      Signal
    Multiplier            :      1.0000
    Dilution              :      1.0000
    Do not use Multiplier & Dilution Factor with ISTDs

    Signal 1: VWD1 A, Wavelength=230 nm
```

Peak #	RetTime [min]	Type	Width [min]	Area [mAU*s]	Height [mAU]	Area %
1	14.819	BB	0.4734	732.06317	23.63449	9.4616
2	16.835	BB	0.5639	7005.12598	190.55113	90.5384

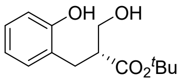

(+)-2d

```
1260II 10/17/2023 10:48:30 AM SYSTEM                                Page 1 of 2
```

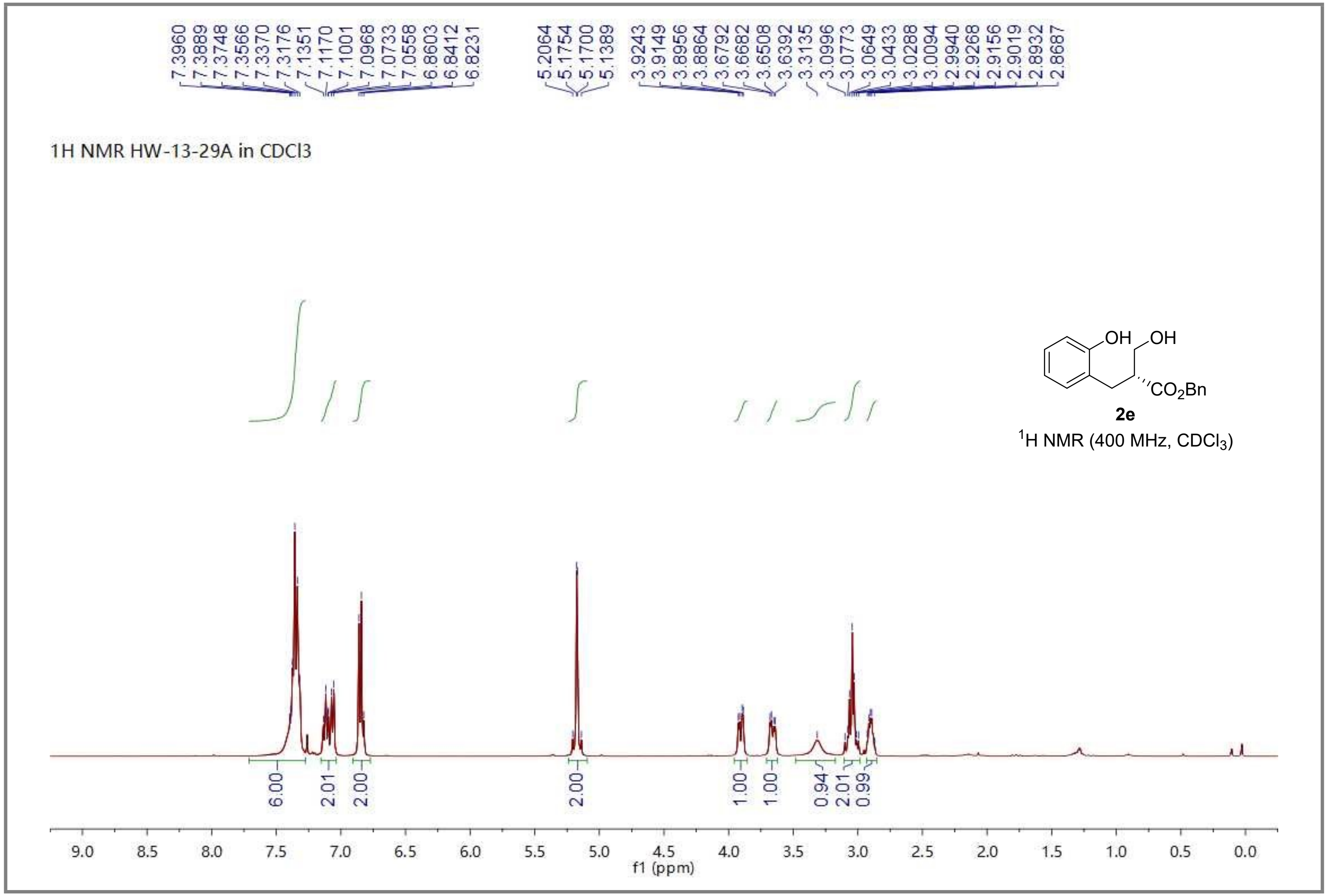
7.3960
7.3889
7.3748
7.3566
7.3370
7.3176
7.1351
7.1170
7.1001
7.0968
7.0733
7.0558
6.8603
6.8412
6.8231
5.2064
5.1754
5.1700
5.1389
3.9243
3.9149
3.8956
3.8864
3.6792
3.6682
3.6508
3.6392
3.3135
3.0996
3.0773
3.0649
3.0433
3.0288
3.0094
2.9940
2.9268
2.9156
2.9019
2.8932
2.8687
1H NMR HW-13-29A in CDCl3
OH
OH
CO2Bn
2e
1H NMR (400 MHz, CDCl3)
6.00
2.01
2.00
2.00
1.00
1.00
0.94
2.01
0.99
9.0
8.5
8.0
7.5
7.0
6.5
6.0
5.5
5.0
4.5
4.0
3.5
3.0
2.5
2.0
1.5
1.0
0.5
0.0
f1 (ppm)

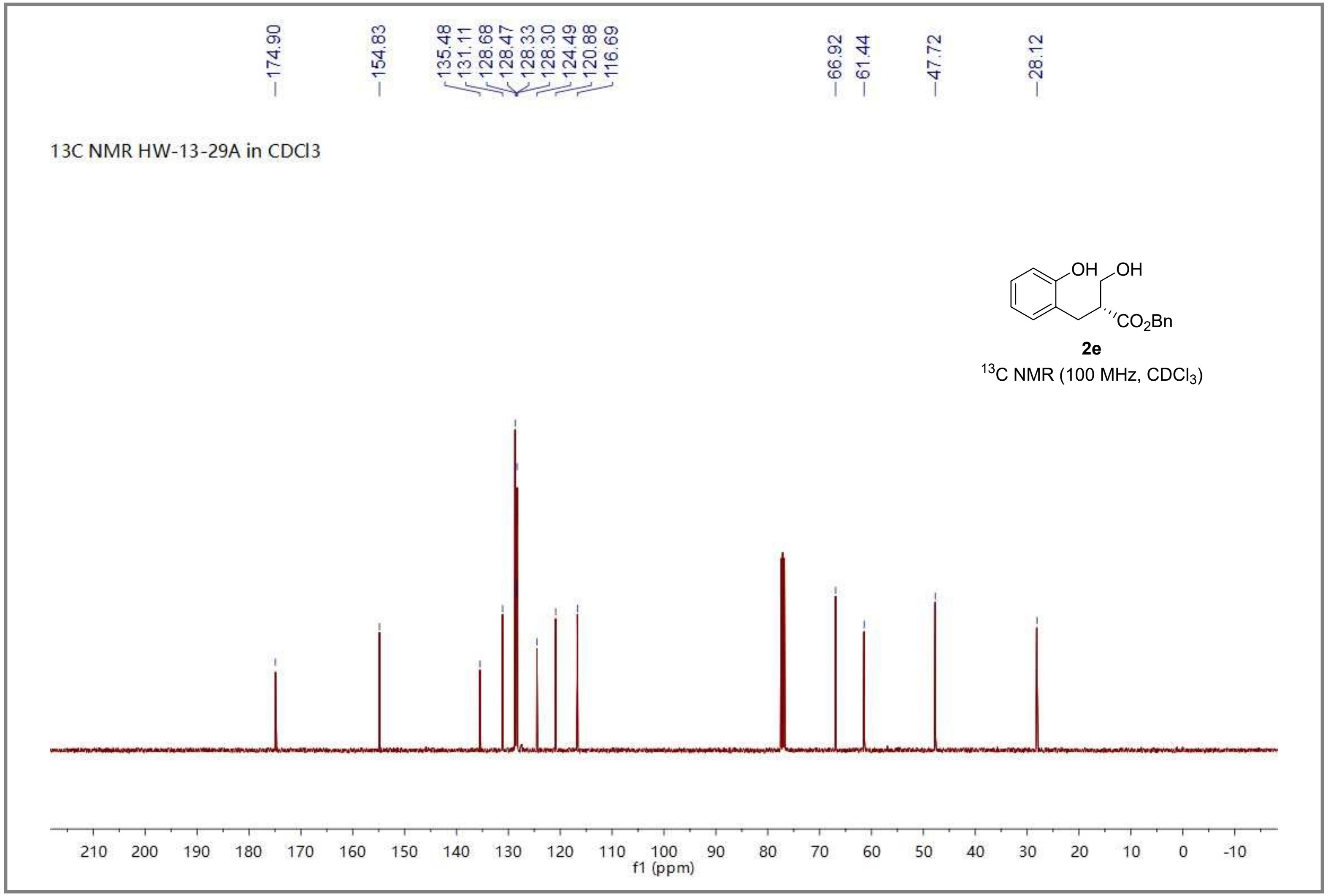

174.90
154.83
135.48
131.11
128.68
128.47
128.33
128.30
124.49
120.88
116.69
66.92
61.44
47.72
28.12
13C NMR HW-13-29A in CDCl3
OH
OH
CO2Bn
2e
13C NMR (100 MHz, CDCl3)
210 200 190 180 170 160 150 140 130 120 110 100 90 80 70 60 50 40 30 20 10 0 -10
f1 (ppm)

Data File C:\CHEM32\1\DATA\ZHOU-22\YZN022312.D
Sample Name: HW-13-29A+/-

===

Acq. Operator :
Acq. Instrument : Instrument 1 Location : -
Injection Date : 1/4/2023 2:43:50 AM
Acq. Method : C:\CHEM32\1\METHODS\DEF_LC.M
Last changed : 1/4/2023 2:41:14 AM
(modified after loading)
Analysis Method : C:\CHEM32\1\METHODS\DEF_LC.M
Last changed : 10/17/2023 10:22:19 PM
(modified after loading)
Sample Info : AS-H, Hexane/i-PrOH = 90/10, 0.8 mL/min, 30 oC, 230 nm

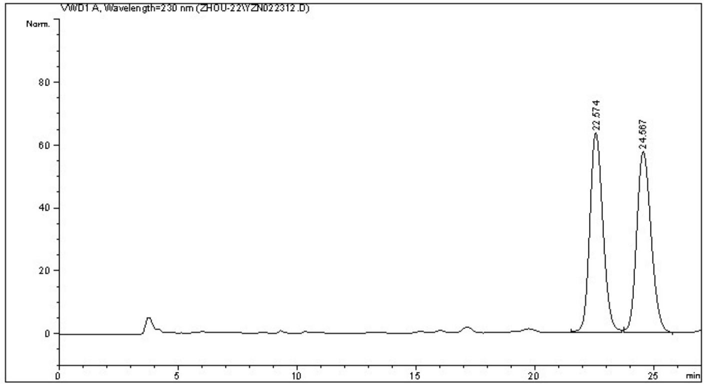

===
Area Percent Report
===

Sorted By : Signal
Multiplier: : 1.0000
Dilution: : 1.0000
Use Multiplier & Dilution Factor with ISTDs

Signal 1: VWD1 A, Wavelength=230 nm

Peak #	RetTime [min]	Type	Width [min]	Area mAU *s	Height [mAU]	Area %
1	22.574	BB	0.5937	2422.07251	63.27313	50.1355
2	24.567	BB	0.6518	2408.98291	57.50860	49.8645
Totals :				4831.05542	120.78173	

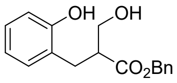

(+/-)-2e

===
*** End of Report ***

Instrument 1 10/17/2023 10:22:22 PM Page 1 of 1

Data File C:\CHEM32\1\DATA\ZHOU-22\YZN022313.D
Sample Name: HW-13-29A

===

Acq. Operator :
Acq. Instrument : Instrument 1 Location : -
Injection Date : 1/4/2023 3:12:47 AM
Acq. Method : C:\CHEM32\1\METHODS\DEF_LC.M
Last changed : 1/4/2023 3:12:07 AM
(modified after loading)
Analysis Method : C:\CHEM32\1\METHODS\DEF_LC.M
Last changed : 10/17/2023 10:23:20 PM
(modified after loading)
Sample Info : AS-H, Hexane/i-PrOH = 90/10, 0.8 mL/min, 30 oC, 230 nm

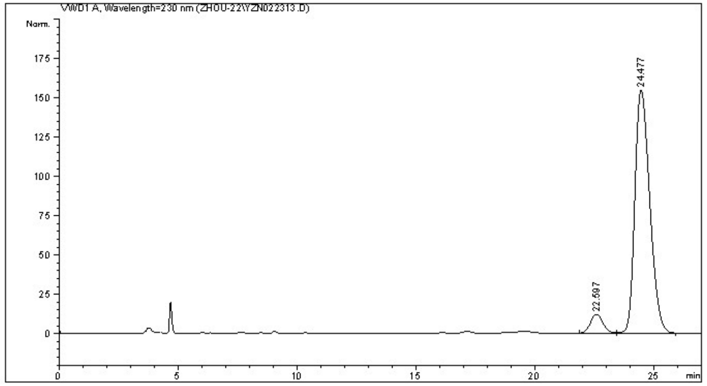

===
Area Percent Report
===

Sorted By : Signal
Multiplier: : 1.0000
Dilution: : 1.0000
Use Multiplier & Dilution Factor with ISTDs

Signal 1: VWD1 A, Wavelength=230 nm

Peak #	RetTime [min]	Type	Width [min]	Area mAU *s	Height [mAU]	Area %
1	22.597	BV	0.5685	436.08621	11.94907	6.1070
2	24.477	VB	0.6742	6704.71338	154.81198	93.8930
Totals :				7140.79959	166.76106	

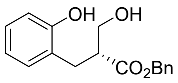

(+)-2e

===
*** End of Report ***

Instrument 1 10/17/2023 10:23:25 PM Page 1 of 1

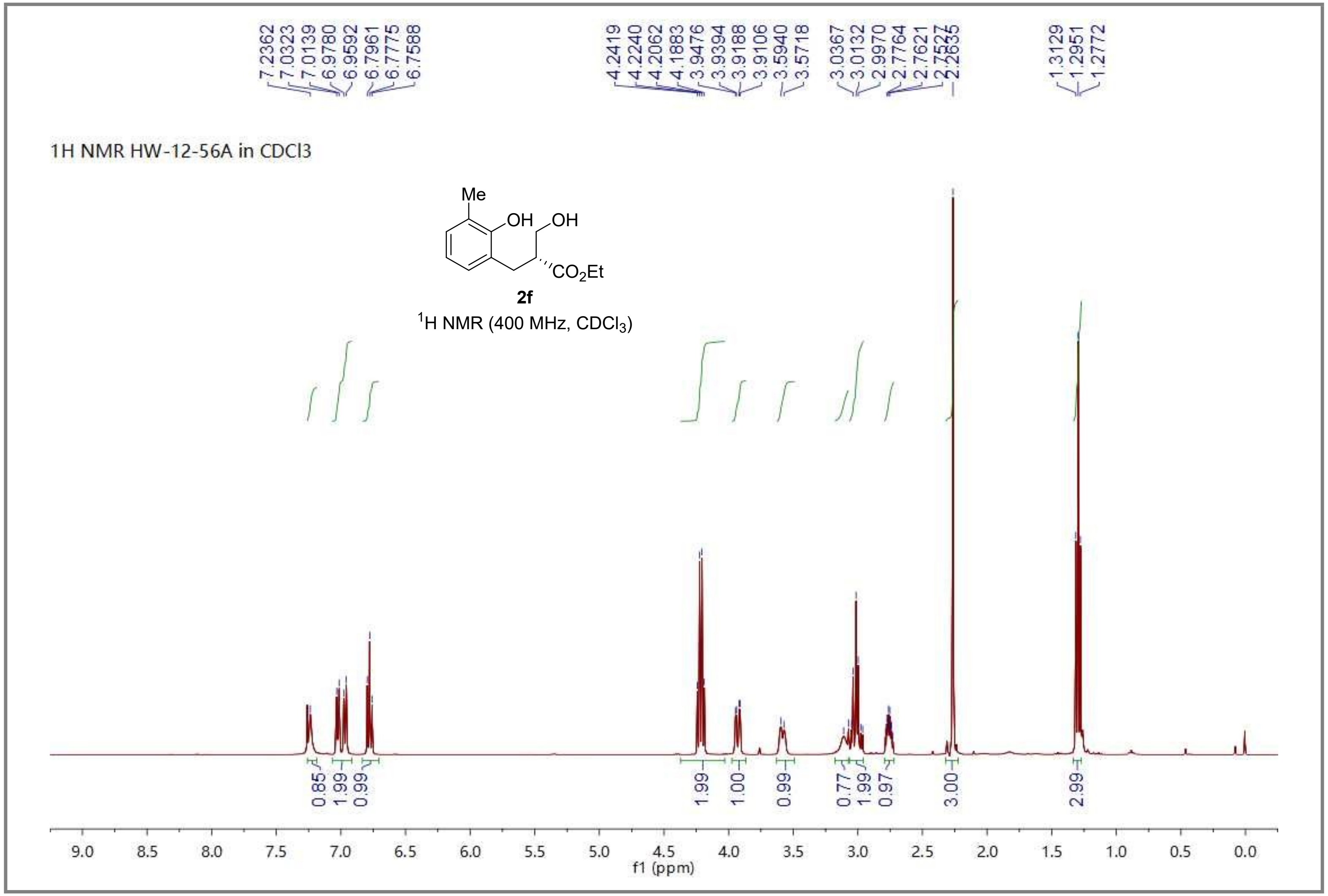
7.2362
7.0323
7.0139
6.9780
6.9592
6.7961
6.7775
6.7588
4.2419
4.2240
4.2062
4.1883
3.9476
3.9394
3.9188
3.9106
3.5940
3.5718
3.0367
3.0132
2.9970
2.7764
2.7621
2.7527
2.2635
1.3129
1.2951
1.2772
1H NMR HW-12-56A in CDCl3
Me
OH
OH
CO2Et
2f
1H NMR (400 MHz, CDCl3)
0.85
1.99
0.99
1.99
1.00
0.99
0.77
1.99
0.97
3.00
2.99
9.0
8.5
8.0
7.5
7.0
6.5
6.0
5.5
5.0
4.5
4.0
3.5
3.0
2.5
2.0
1.5
1.0
0.5
0.0
f1 (ppm)

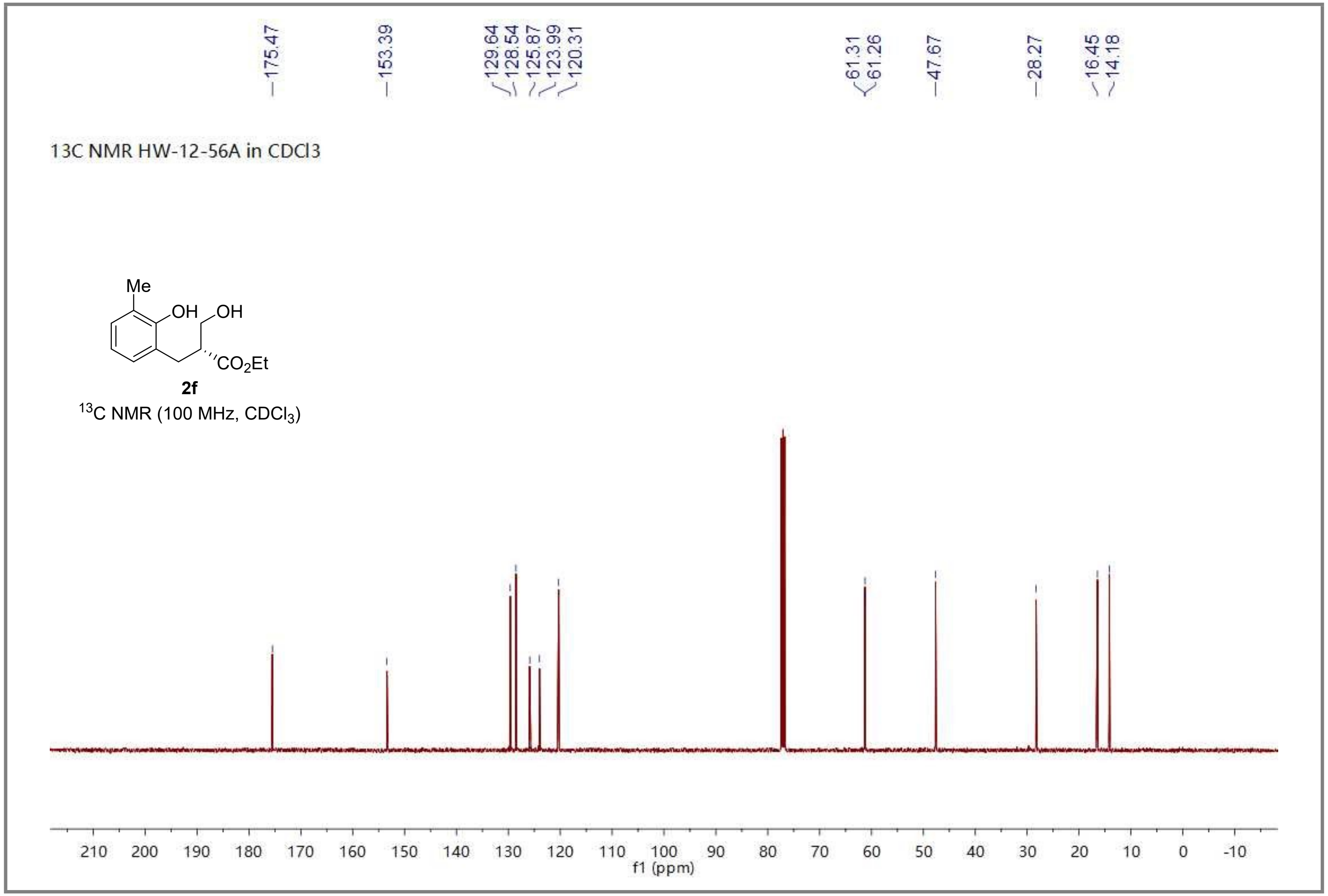

175.47
153.39
129.64
128.54
125.87
123.99
120.31
61.31
61.26
47.67
28.27
16.45
14.18
13C NMR HW-12-56A in CDCl3
Me
OH
OH
CO2Et
2f
13C NMR (100 MHz, CDCl3)
210
200
190
180
170
160
150
140
130
120
110
100
90
80
70
60
50
40
30
20
10
0
-10
f1 (ppm)

Data File C:\USERS\PUBLIC\DOCUMENTS\CHEMSTATION\1\DATA\2022\YZH002733 09-05-03.D
Sample Name: HW-12-56A+/-

===

Acq. Operator : SYSTEM
Sample Operator : SYSTEM
Acq. Instrument : HPLC1260 II Location : 1
Injection Date : 11/7/2022 9:05:03 AM Inj : 1
Inj Volume : No inj
Acq. Method : C:\USERS\PUBLIC\DOCUMENTS\CHEMSTATION\1\METHODS\def_LC1.M
Last changed : 11/7/2022 8:39:09 AM by SYSTEM
(modified after loading)
Analysis Method : C:\USERS\PUBLIC\DOCUMENTS\CHEMSTATION\1\METHODS\def_LC1.M
Last changed : 10/17/2023 10:39:05 AM by SYSTEM
(modified after loading)
Sample Info : AS-H, Hexane/i-PrOH = 90/10, 0.8 mL/min, 30 oC, 230 nm

Additional Info : Peak(s) manually integrated

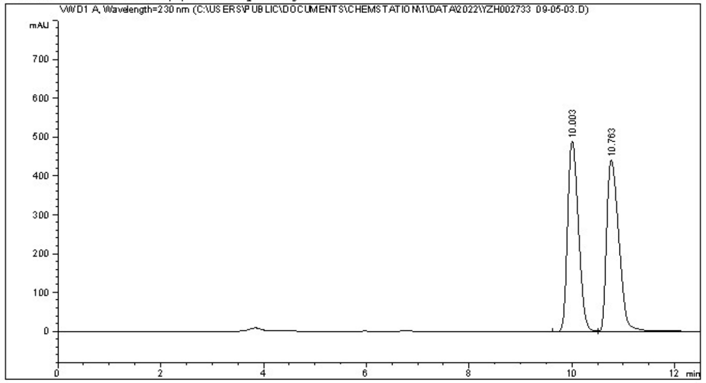

===
Area Percent Report
===

Sorted By : Signal
Multiplier : 1.0000
Dilution : 1.0000
Do not use Multiplier & Dilution Factor with ISTDs

Signal 1: VWD1 A, Wavelength=230 nm

Peak #	RetTime [min]	Type	Width [min]	Area [mAU*s]	Height [mAU]	Area %
1	10.003	BV	0.2300	7136.70947	489.74524	49.3328
2	10.763	VB	0.2606	7329.75830	441.75406	50.6672

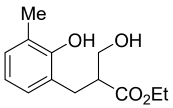

(+/-)-2f

HPLC1260 II 10/17/2023 10:39:09 AM SYSTEM

Page 1 of 2

Data File C:\USERS\PUBLIC\DOCUMENTS\CHEMSTATION\1\DATA\2022\YZH002734 09-27-08.D
Sample Name: HW-12-56A

===

Acq. Operator : SYSTEM
Sample Operator : SYSTEM
Acq. Instrument : HPLC1260 II Location : 1
Injection Date : 11/7/2022 9:27:09 AM Inj : 1
Inj Volume : No inj
Acq. Method : C:\USERS\PUBLIC\DOCUMENTS\CHEMSTATION\1\METHODS\def_LC1.M
Last changed : 11/7/2022 8:39:09 AM by SYSTEM
(modified after loading)
Analysis Method : C:\USERS\PUBLIC\DOCUMENTS\CHEMSTATION\1\METHODS\def_LC1.M
Last changed : 10/17/2023 10:39:05 AM by SYSTEM
(modified after loading)
Sample Info : AS-H, Hexane/i-PrOH = 90/10, 0.8 mL/min, 30 oC, 230 nm

Additional Info : Peak(s) manually integrated

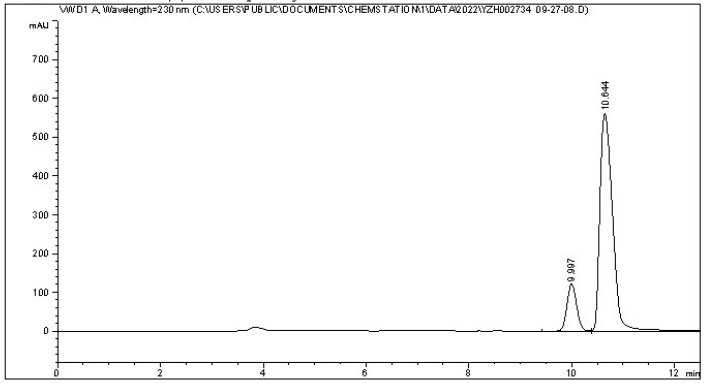

===
Area Percent Report
===

Sorted By : Signal
Multiplier : 1.0000
Dilution : 1.0000
Do not use Multiplier & Dilution Factor with ISTDs

Signal 1: VWD1 A, Wavelength=230 nm

Peak #	RetTime [min]	Type	Width [min]	Area [mAU*s]	Height [mAU]	Area %
1	9.997	BV	0.2035	1571.79883	121.00585	14.2329
2	10.644	VB	0.2770	9471.63477	560.87994	85.7671

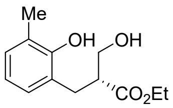

(+)-2f

HPLC1260 II 10/17/2023 10:39:44 AM SYSTEM

Page 1 of 2

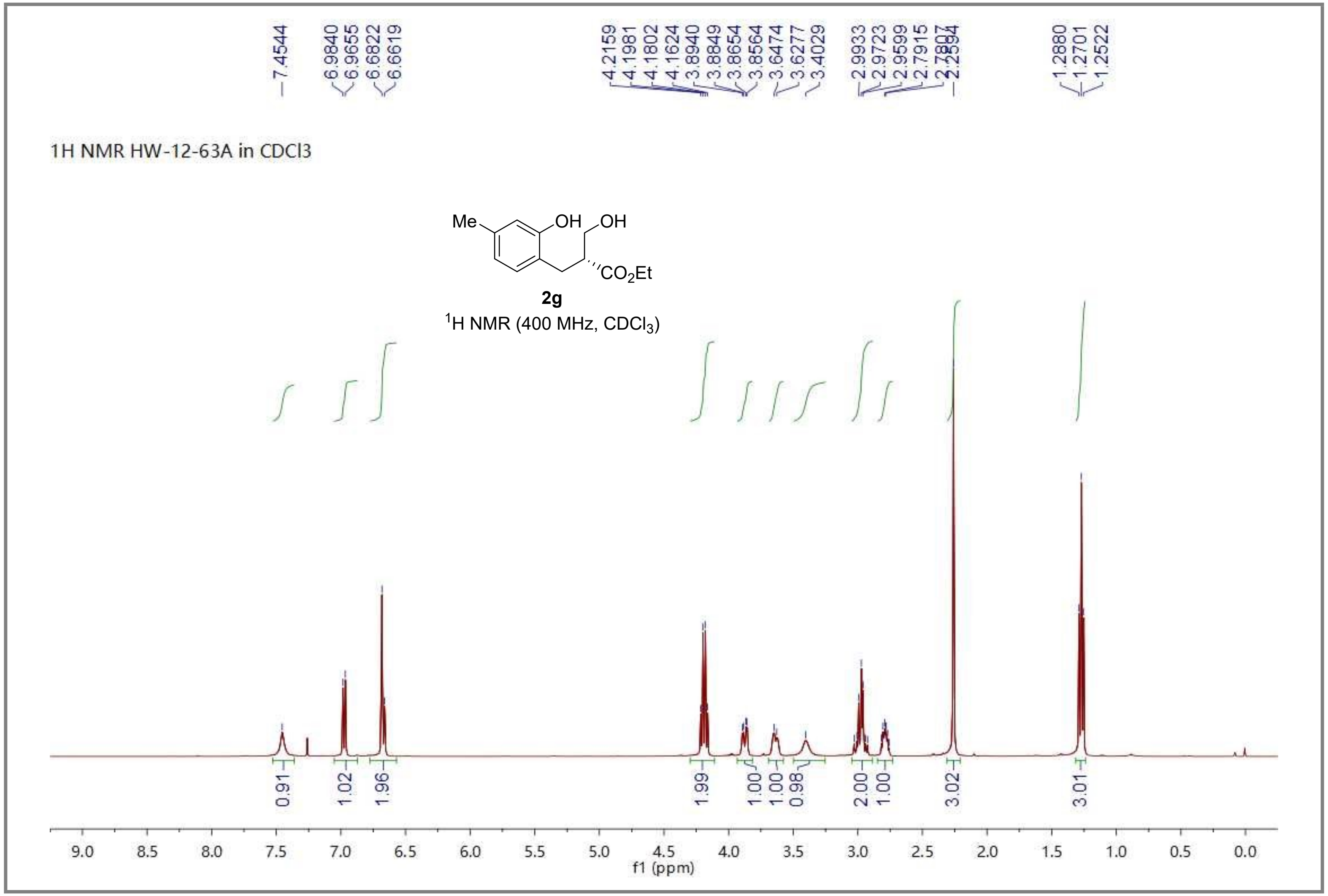
1H NMR HW-12-63A in CDCl3
Me
OH
OH
CO2Et
2g
1H NMR (400 MHz, CDCl3)
f1 (ppm)

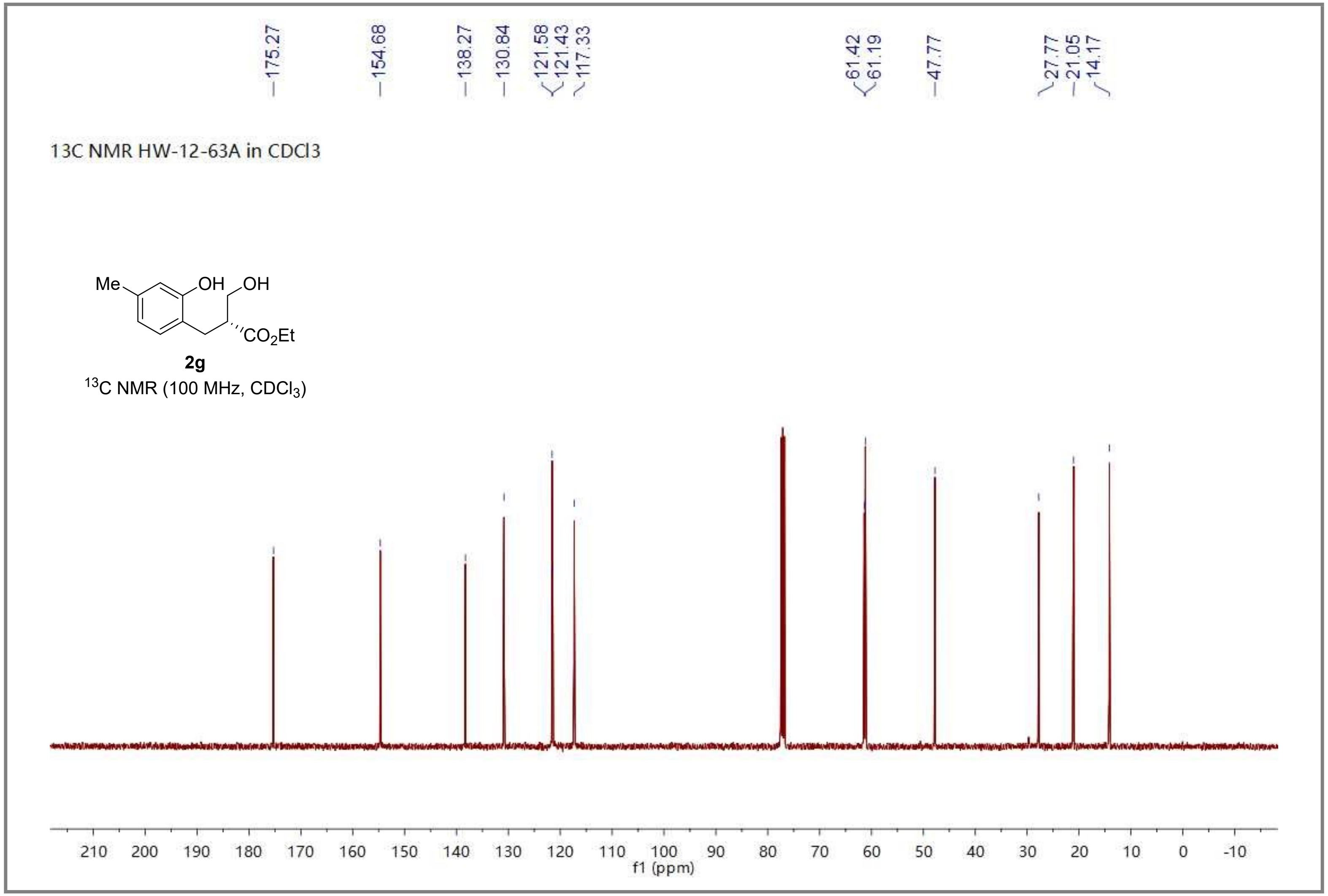

175.27
154.68
138.27
130.84
121.58
121.43
117.33
61.42
61.19
47.77
27.77
21.05
14.17
13C NMR HW-12-63A in CDCl3
Me
OH
OH
CO2Et
2g
13C NMR (100 MHz, CDCl3)
210 200 190 180 170 160 150 140 130 120 110 100 90 80 70 60 50 40 30 20 10 0 -10
f1 (ppm)

Data File C:\CHEM32\1\DATA\ZHOU-22\YZN022050.D
Sample Name: HW-12-63A+/-

===

Acq. Operator :
Acq. Instrument : Instrument 1 Location : -
Injection Date : 11/15/2022 10:36:57 PM
Acq. Method : C:\CHEM32\1\METHODS\DEF_LC.M
Last changed : 11/15/2022 10:36:31 PM
(modified after loading)
Analysis Method : C:\CHEM32\1\METHODS\DEF_LC.M
Last changed : 10/17/2023 10:32:22 PM
(modified after loading)
Sample Info : IB, Hexane/i-PrOH = 95/5, 0.8 mL/min, 30 oC, 230 nm

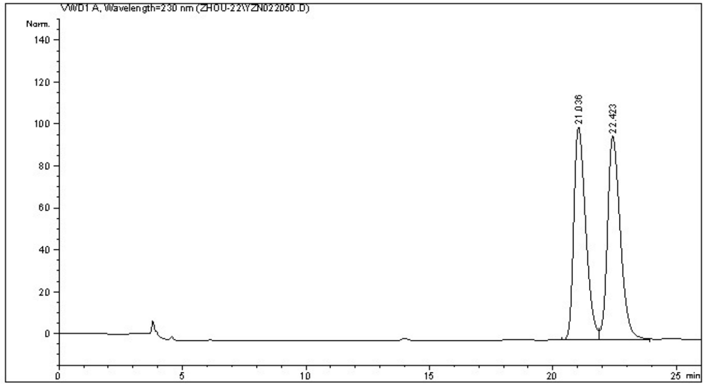

===
Area Percent Report
===

Sorted By : Signal
Multiplier: : 1.0000
Dilution: : 1.0000
Use Multiplier & Dilution Factor with ISTDs

Signal 1: VWD1 A, Wavelength=230 nm

Peak #	RetTime [min]	Type	Width [min]	Area mAU *s	Height [mAU]	Area %
1	21.036	BV	0.5132	3400.93555	101.50028	49.4587
2	22.423	VB	0.5518	3475.38354	97.02937	50.5413

Totals : 6876.31909 198.52966

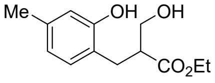

(+/-)-2g

===
*** End of Report ***

Instrument 1 10/17/2023 10:32:25 PM Page 1 of 1

Data File C:\CHEM32\1\DATA\ZHOU-22\YZN022052.D
Sample Name: HW-12-63A

===

Acq. Operator :
Acq. Instrument : Instrument 1 Location : -
Injection Date : 11/15/2022 11:36:05 PM
Acq. Method : C:\CHEM32\1\METHODS\DEF_LC.M
Last changed : 11/15/2022 11:34:22 PM
(modified after loading)
Analysis Method : C:\CHEM32\1\METHODS\DEF_LC.M
Last changed : 10/17/2023 10:33:11 PM
(modified after loading)
Sample Info : IB, Hexane/i-PrOH = 95/5, 0.8 mL/min, 30 oC, 230 nm

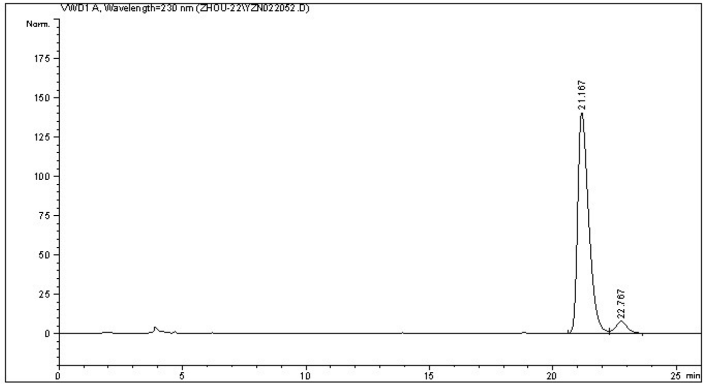

===
Area Percent Report
===

Sorted By : Signal
Multiplier: : 1.0000
Dilution: : 1.0000
Use Multiplier & Dilution Factor with ISTDs

Signal 1: VWD1 A, Wavelength=230 nm

Peak #	RetTime [min]	Type	Width [min]	Area mAU *s	Height [mAU]	Area %
1	21.167	BV	0.4709	4340.50195	140.55257	94.6579
2	22.767	VB	0.4975	244.96194	7.55889	5.3421

Totals : 4585.46390 148.11145

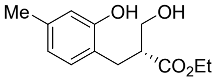

(+)-2g

===
*** End of Report ***

Instrument 1 10/17/2023 10:33:14 PM Page 1 of 1

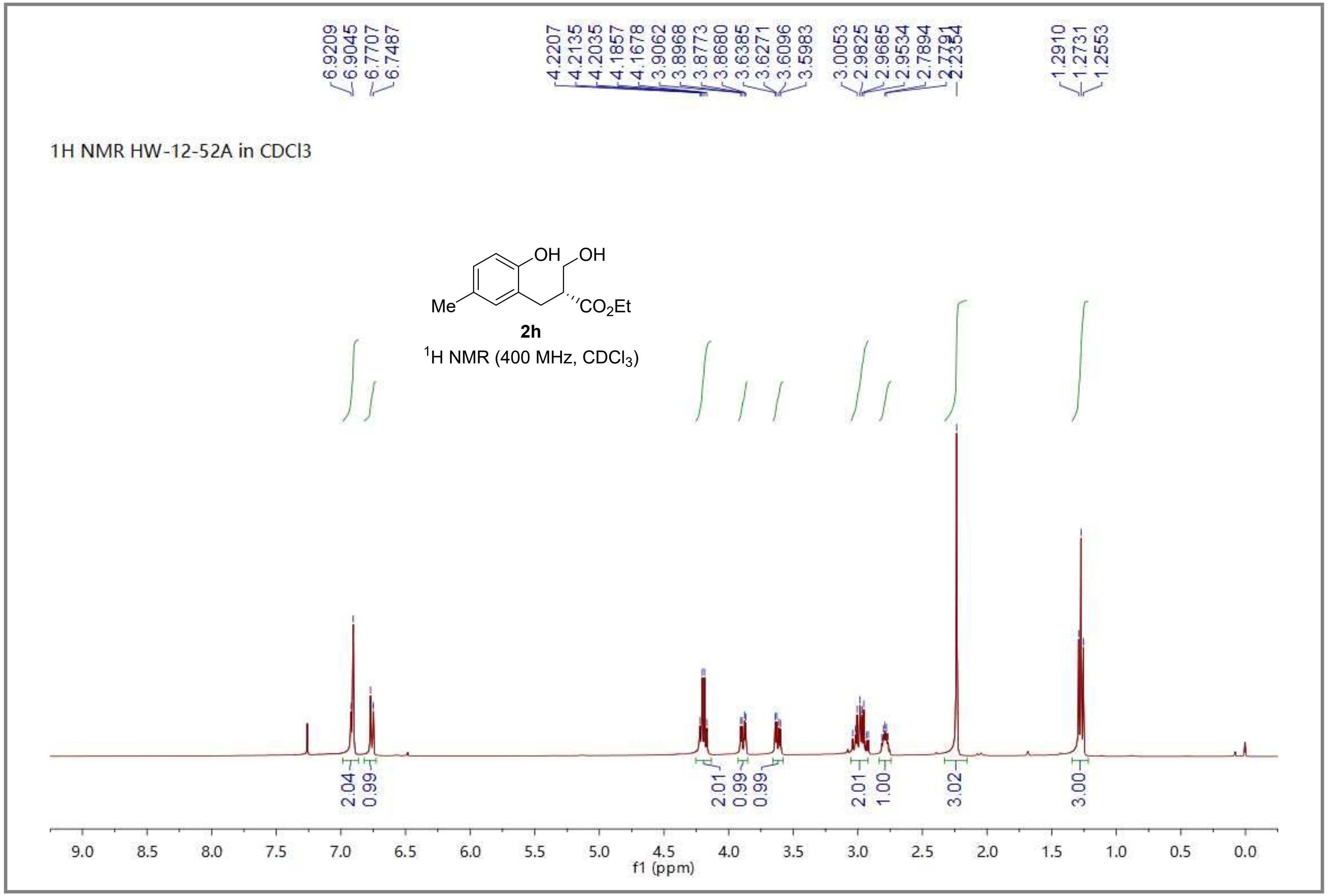
1H NMR HW-12-52A in CDCl3
OH
OH
Me
CO2Et
2h
1H NMR (400 MHz, CDCl3)
6.9209
6.9045
6.7707
6.7487
4.2207
4.2135
4.2035
4.1857
4.1678
3.9062
3.8968
3.8773
3.8680
3.6385
3.6271
3.6096
3.5983
3.0053
2.9825
2.9685
2.9534
2.7894
2.7791
2.2354
1.2910
1.2731
1.2553
2.04
0.99
2.01
0.99
0.99
2.01
1.00
3.02
3.00
f1 (ppm)

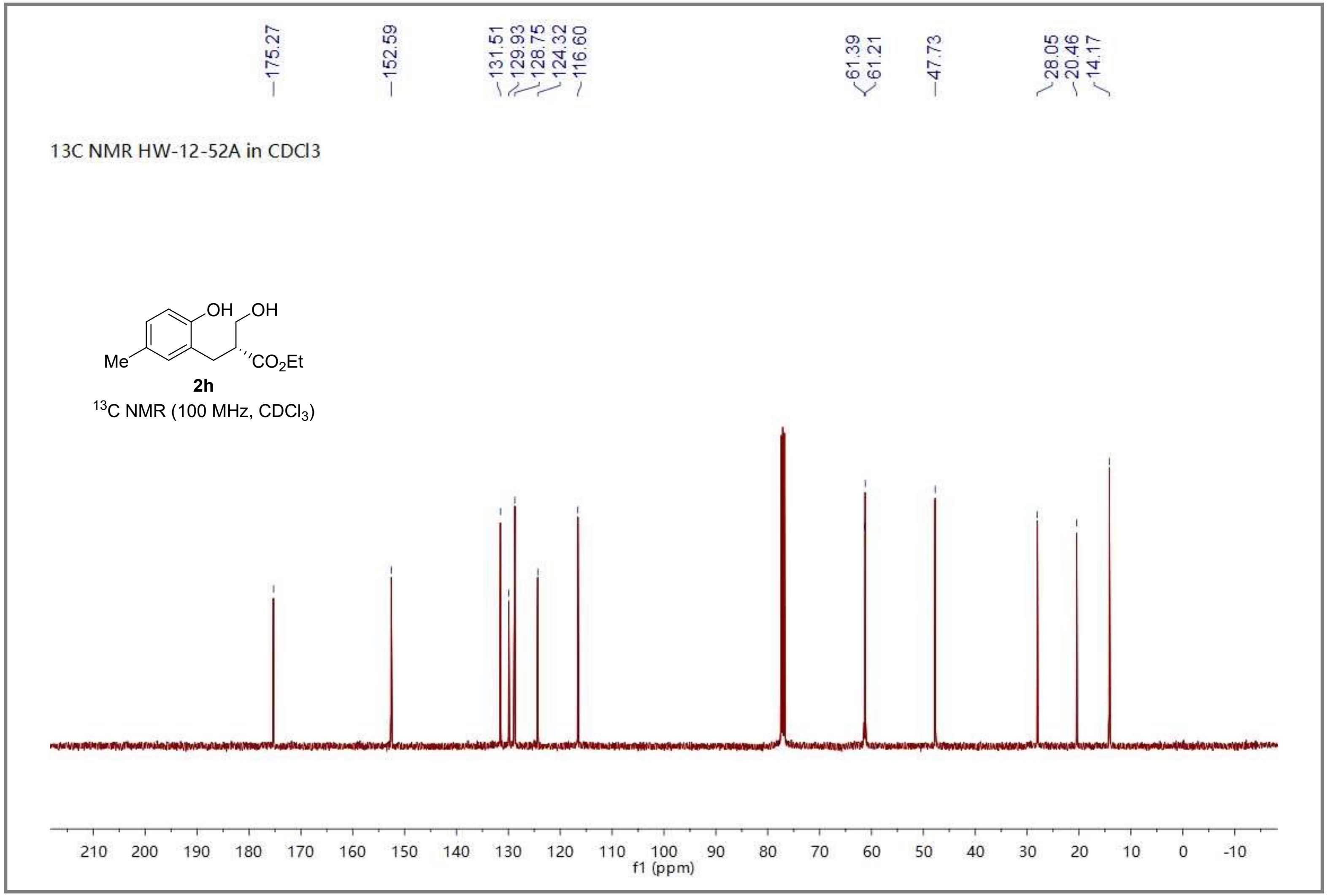

175.27
152.59
131.51
129.93
128.75
124.32
116.60
61.39
61.21
47.73
28.05
20.46
14.17
13C NMR HW-12-52A in CDCl3
OH
OH
Me
CO2Et
2h
13C NMR (100 MHz, CDCl3)
210 200 190 180 170 160 150 140 130 120 110 100 90 80 70 60 50 40 30 20 10 0 -10
f1 (ppm)

Data File C:\USERS\PUBLIC\DOCUMENTS\CHEMSTATION\1\DATA\2023\YZH005564 18-51-45.D
Sample Name: HW-12-52A+/-

===

Acq. Operator : SYSTEM
Sample Operator : SYSTEM
Acq. Instrument : HPLC1260 II Location : 1
Injection Date : 11/28/2023 6:51:45 PM Inj : 1
Inj Volume : No inj
Acq. Method : C:\USERS\PUBLIC\DOCUMENTS\CHEMSTATION\1\METHODS\def_LC1.M
Last changed : 11/28/2023 6:48:19 PM by SYSTEM
(modified after loading)
Analysis Method : C:\USERS\PUBLIC\DOCUMENTS\CHEMSTATION\1\METHODS\def_LC1.M
Last changed : 11/29/2023 11:05:26 AM by SYSTEM
(modified after loading)
Sample Info : AS-H, Hexane/i-PrOH = 95/5, 0.8 mL/min, 30 oC, 230 nm

Additional Info : Peak(s) manually integrated

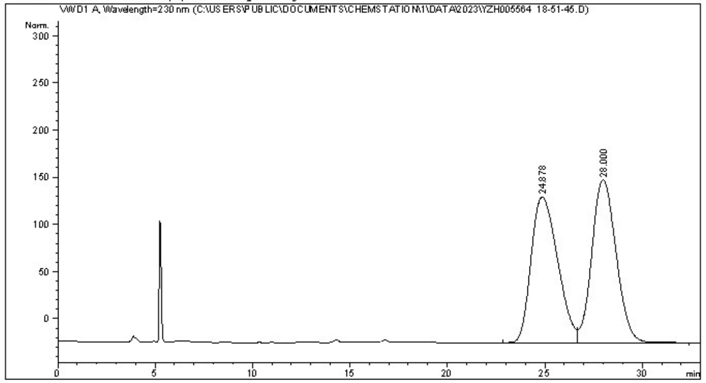

===
Area Percent Report
===

Sorted By : Signal
Multiplier : 1.0000
Dilution : 1.0000
Sample Amount: : 1.00000 [ng/ul] (not used in calc.)
Do not use Multiplier & Dilution Factor with ISTDs

Signal 1: VWD1 A, Wavelength=230 nm

Peak #	RetTime [min]	Type	Width [min]	Area [mAU*s]	Height [mAU]	Area %
1	24.878	BV	1.4839	1.05224e4	111.00722	49.4465
2	28.000	VB	1.3391	1.07580e4	123.88742	50.5535

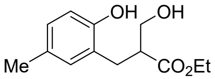

(+/-)-2h

HPLC1260 II 11/29/2023 11:05:30 AM SYSTEM

Page 1 of 2

Data File C:\USERS\PUBLIC\DOCUMENTS\CHEMSTATION\1\DATA\2023\YZH005565 19-26-57.D
Sample Name: HW-12-52A

===

Acq. Operator : SYSTEM
Sample Operator : SYSTEM
Acq. Instrument : HPLC1260 II Location : 1
Injection Date : 11/28/2023 7:26:57 PM Inj : 1
Inj Volume : No inj
Acq. Method : C:\USERS\PUBLIC\DOCUMENTS\CHEMSTATION\1\METHODS\def_LC1.M
Last changed : 11/28/2023 6:48:19 PM by SYSTEM
(modified after loading)
Analysis Method : C:\USERS\PUBLIC\DOCUMENTS\CHEMSTATION\1\METHODS\def_LC1.M
Last changed : 11/29/2023 11:06:40 AM by SYSTEM
(modified after loading)
Sample Info : AS-H, Hexane/i-PrOH = 95/5, 0.8 mL/min, 30 oC, 230 nm

Additional Info : Peak(s) manually integrated

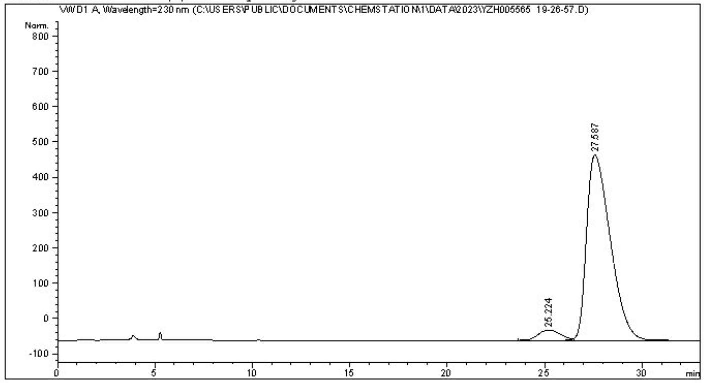

===
Area Percent Report
===

Sorted By : Signal
Multiplier : 1.0000
Dilution : 1.0000
Sample Amount: : 1.00000 [ng/ul] (not used in calc.)
Do not use Multiplier & Dilution Factor with ISTDs

Signal 1: VWD1 A, Wavelength=230 nm

Peak #	RetTime [min]	Type	Width [min]	Area [mAU*s]	Height [mAU]	Area %
1	25.224	BV E	1.2266	1771.88013	22.62739	4.9326
2	27.587	VB R	1.3056	3.41502e4	402.61203	95.0674

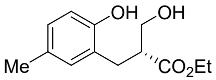

(+)-2h

HPLC1260 II 11/29/2023 11:06:46 AM SYSTEM

Page 1 of 2

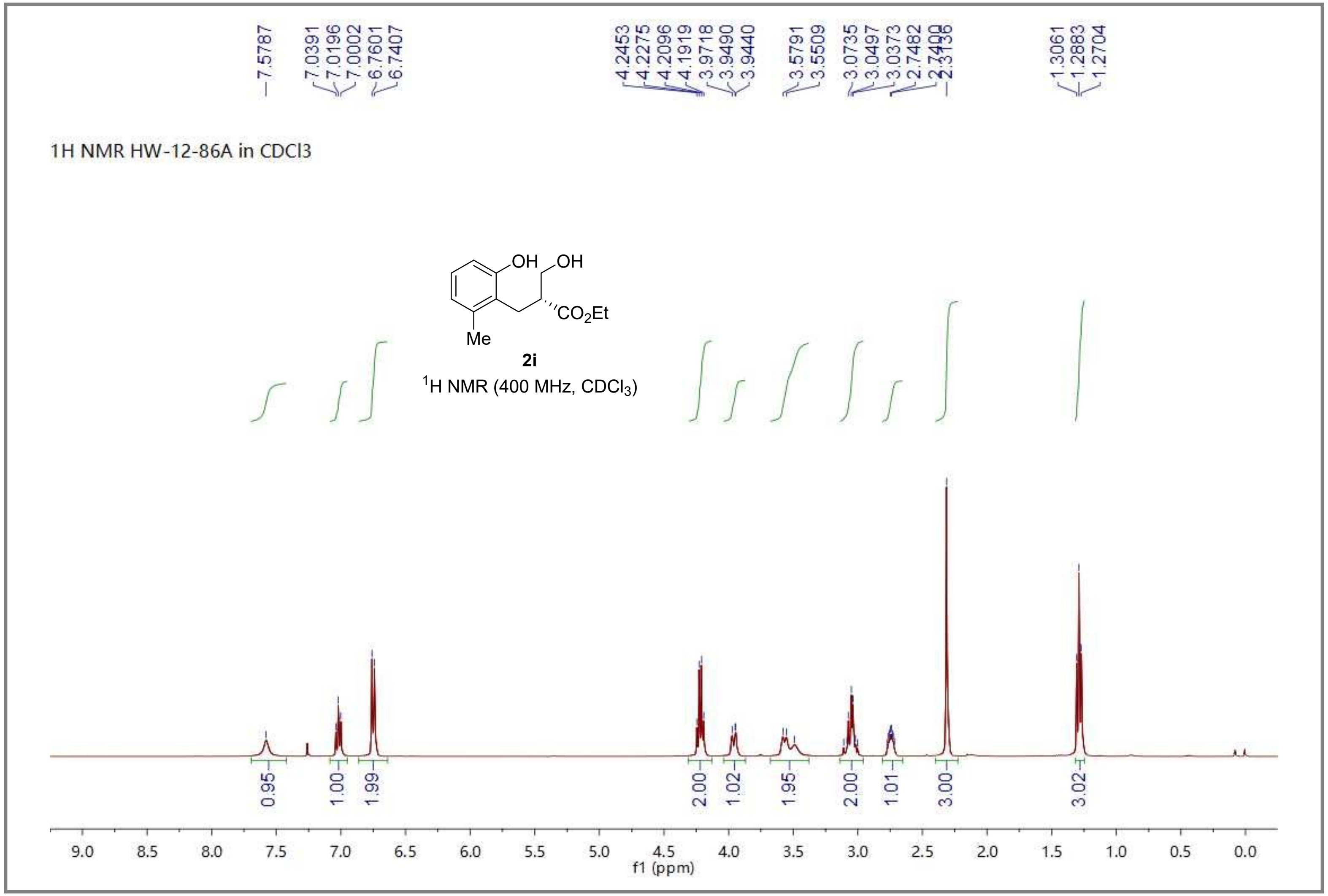

7.5787
7.0391
7.0196
7.0002
6.7601
6.7407
4.2453
4.2275
4.2096
4.1919
3.9718
3.9490
3.9440
3.5791
3.5509
3.0735
3.0497
3.0373
2.7482
2.7400
2.3136
1.3061
1.2883
1.2704
1H NMR HW-12-86A in CDCl3
OH
OH
CO2Et
Me
2i
1H NMR (400 MHz, CDCl3)
0.95
1.00
1.99
2.00
1.02
1.95
2.00
1.01
3.00
3.02
9.0
8.5
8.0
7.5
7.0
6.5
6.0
5.5
5.0
4.5
4.0
3.5
3.0
2.5
2.0
1.5
1.0
0.5
0.0
f1 (ppm)

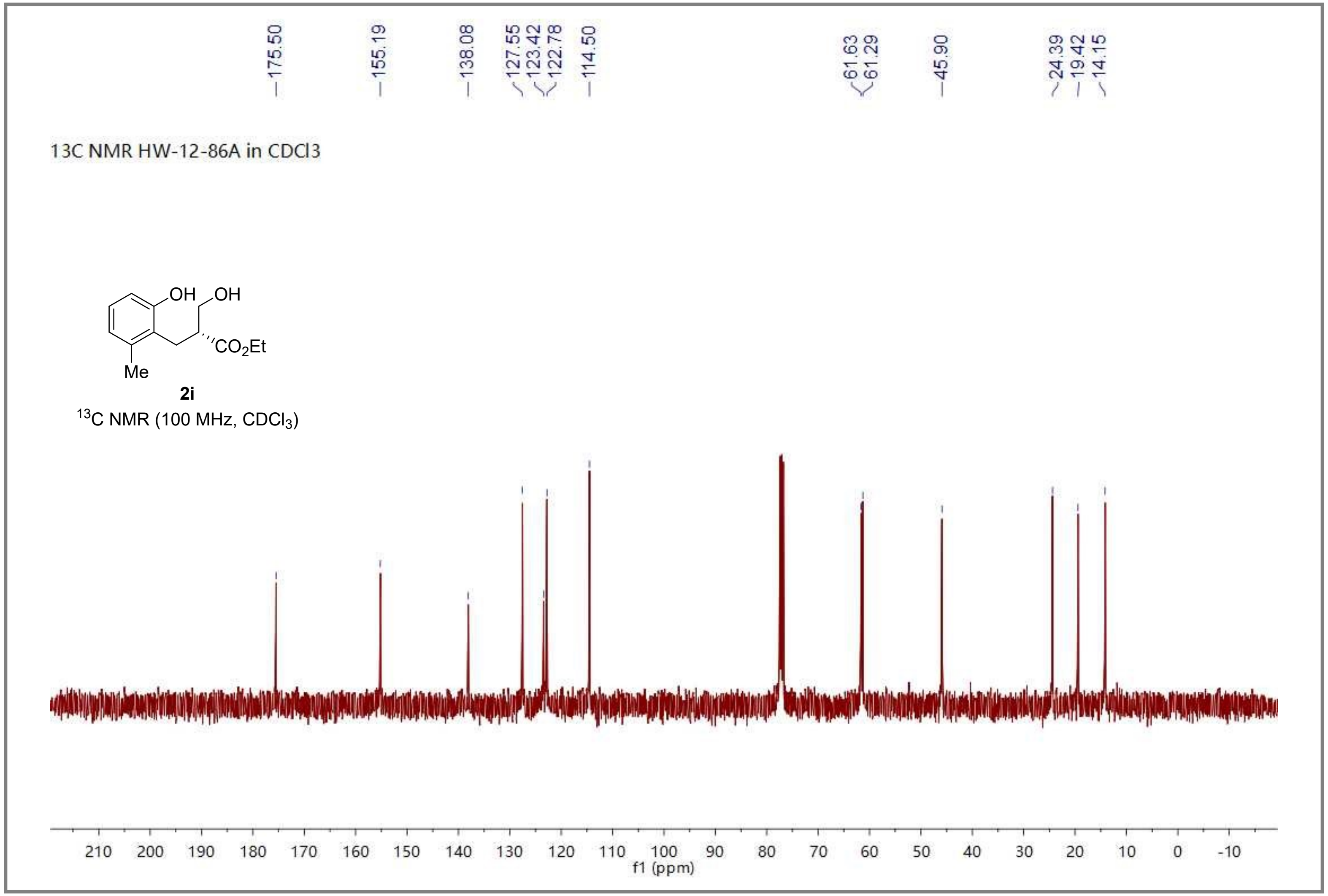
175.50
155.19
138.08
127.55
123.42
122.78
114.50
61.63
61.29
45.90
24.39
19.42
14.15
13C NMR HW-12-86A in CDCl3
OH
OH
CO2Et
Me
2i
13C NMR (100 MHz, CDCl3)
210 200 190 180 170 160 150 140 130 120 110 100 90 80 70 60 50 40 30 20 10 0 -10
f1 (ppm)

```
Data File D:\USERS\CHEMSTATION\1\DATA\ZHOU2022\YZ3005839  20-17-56.D
Sample Name: HW-12-86A+/-

    =====================================================================
    Acq. Operator   : SYSTEM
    Sample Operator : SYSTEM
    Acq. Instrument : 1260II                     Location :     -
    Injection Date  : 12/6/2022 8:17:56 PM                 Inj :   1
                                              Inj Volume : No inj
    Acq. Method     : C:\USERS\PUBLIC\DOCUMENTS\CHEMSTATION\1\METHODS\lsm-1-58.M
    Last changed    : 12/6/2022 8:16:54 PM by SYSTEM
                      (modified after loading)
    Analysis Method : C:\Users\Public\Documents\ChemStation\1\Methods\def_LC.M
    Last changed    : 10/17/2023 10:49:33 AM by SYSTEM
                      (modified after loading)
    Sample Info     : AS-H, n-Hexane/i-PrOH = 90/10, 0.8  mL/min, 30 oC, 230 nm

    Additional Info : Peak(s) manually integrated
```

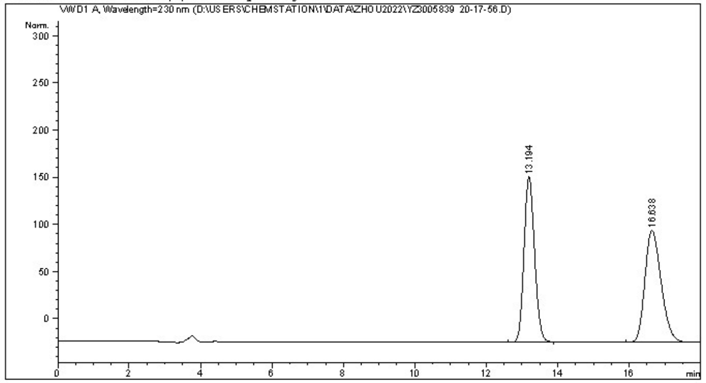


```
    =====================================================================
                         Area Percent Report
    =====================================================================

    Sorted By             :      Signal
    Multiplier            :      1.0000
    Dilution              :      1.0000
    Do not use Multiplier & Dilution Factor with ISTDs


    Signal 1: VWD1 A, Wavelength=230 nm

    Peak RetTime Type  Width     Area      Height     Area
      #   [min]        [min]   [mAU*s]     [mAU]        %
    ----|-------|----|-------|----------|----------|--------|
       1  13.194 BB    0.3200 2606.14819  127.19192  48.9720
       2  16.638 BB    0.4914 2715.56299   85.98973  51.0280
```

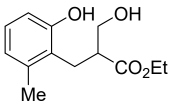

(+/-)-2i

```
1260II 10/17/2023 10:49:36 AM SYSTEM                              Page 1 of 2
```

```
Data File D:\USERS\CHEMSTATION\1\DATA\ZHOU2022\YZ3005840  20-38-28.D
Sample Name: HW-12-86A

    =====================================================================
    Acq. Operator   : SYSTEM
    Sample Operator : SYSTEM
    Acq. Instrument : 1260II                     Location :     -
    Injection Date  : 12/6/2022 8:38:28 PM                 Inj :   1
                                              Inj Volume : No inj
    Acq. Method     : C:\USERS\PUBLIC\DOCUMENTS\CHEMSTATION\1\METHODS\lsm-1-58.M
    Last changed    : 12/6/2022 8:16:54 PM by SYSTEM
                      (modified after loading)
    Analysis Method : C:\Users\Public\Documents\ChemStation\1\Methods\def_LC.M
    Last changed    : 10/17/2023 10:50:22 AM by SYSTEM
                      (modified after loading)
    Sample Info     : AS-H, n-Hexane/i-PrOH = 90/10, 0.8  mL/min, 30 oC, 230 nm

    Additional Info : Peak(s) manually integrated
```

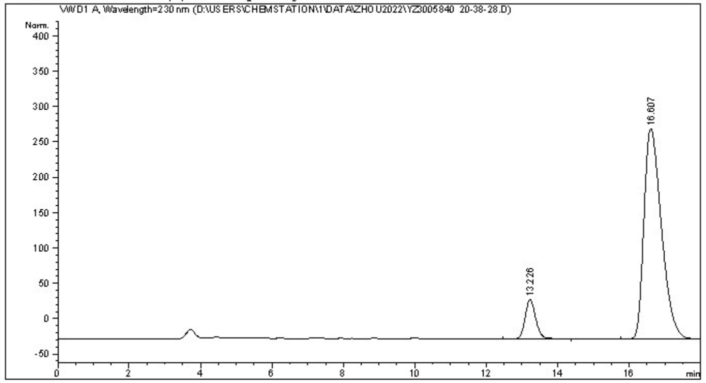


```
    =====================================================================
                         Area Percent Report
    =====================================================================

    Sorted By             :      Signal
    Multiplier            :      1.0000
    Dilution              :      1.0000
    Do not use Multiplier & Dilution Factor with ISTDs


    Signal 1: VWD1 A, Wavelength=230 nm

    Peak RetTime Type  Width     Area      Height     Area
      #   [min]        [min]   [mAU*s]     [mAU]        %
    ----|-------|----|-------|----------|----------|--------|
       1  13.226 BB    0.3164  922.83313   44.96306  10.1848
       2  16.607 BB    0.5258 8138.02637  239.87434  89.8152
```

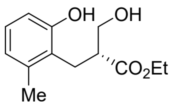

(+)-2i

```
1260II 10/17/2023 10:50:25 AM SYSTEM                              Page 1 of 2
```

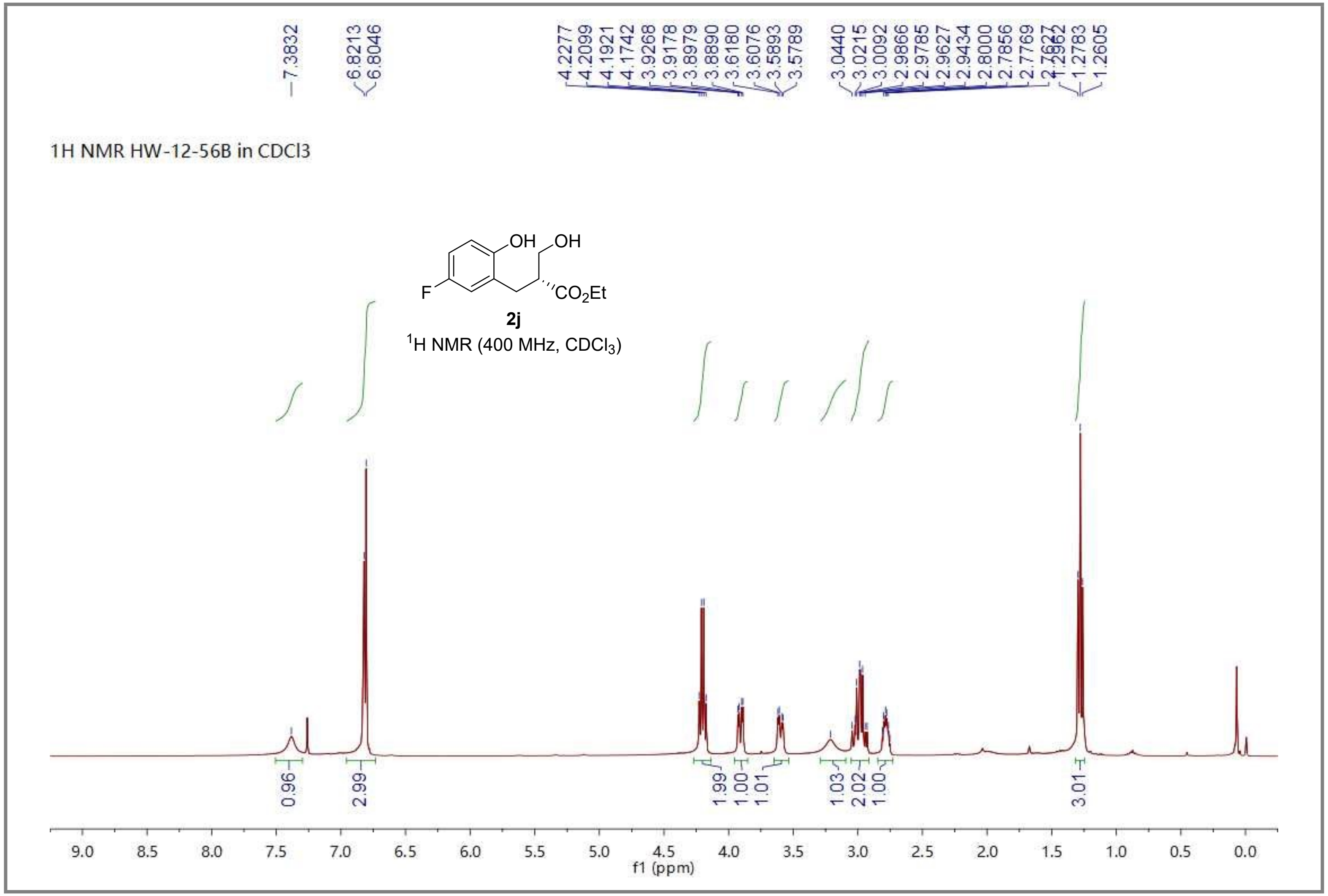

7.3832
6.8213
6.8046
4.2277
4.2099
4.1921
4.1742
3.9268
3.9178
3.8979
3.8890
3.6180
3.6076
3.5893
3.5789
3.0440
3.0215
3.0092
2.9866
2.9785
2.9627
2.9434
2.8000
2.7856
2.7769
2.7627
1.2962
1.2783
1.2605
1H NMR HW-12-56B in CDCl3
OH
OH
F
CO2Et
2j
1H NMR (400 MHz, CDCl3)
0.96
2.99
1.99
1.00
1.01
1.03
2.02
1.00
3.01
9.0
8.5
8.0
7.5
7.0
6.5
6.0
5.5
5.0
4.5
4.0
3.5
3.0
2.5
2.0
1.5
1.0
0.5
0.0
f1 (ppm)

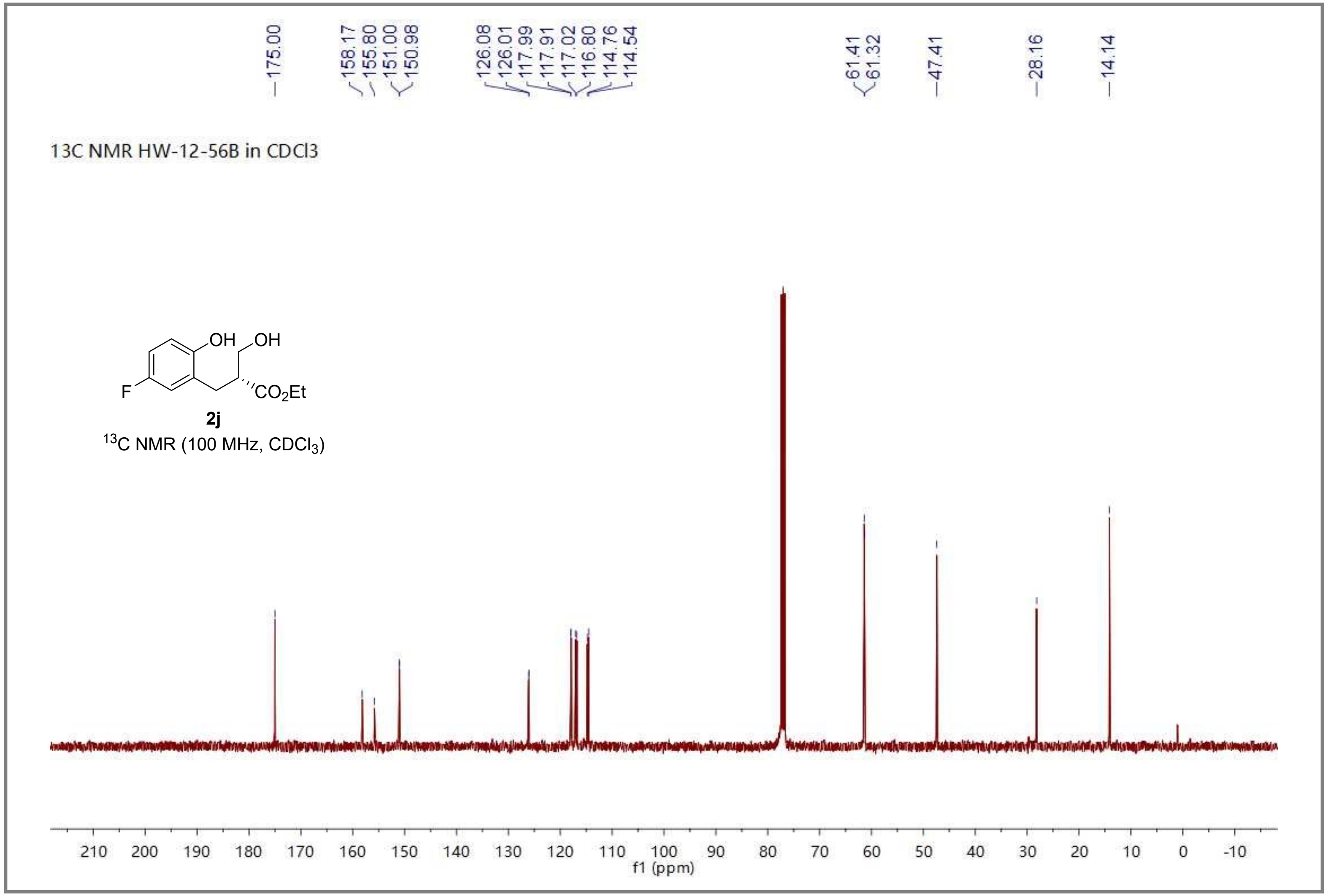
175.00
158.17
155.80
151.00
150.98
126.08
126.01
117.99
117.91
117.02
116.80
114.76
114.54
61.41
61.32
47.41
28.16
14.14
13C NMR HW-12-56B in CDCl3
OH
OH
F
CO2Et
2j
13C NMR (100 MHz, CDCl3)
210 200 190 180 170 160 150 140 130 120 110 100 90 80 70 60 50 40 30 20 10 0 -10
f1 (ppm)

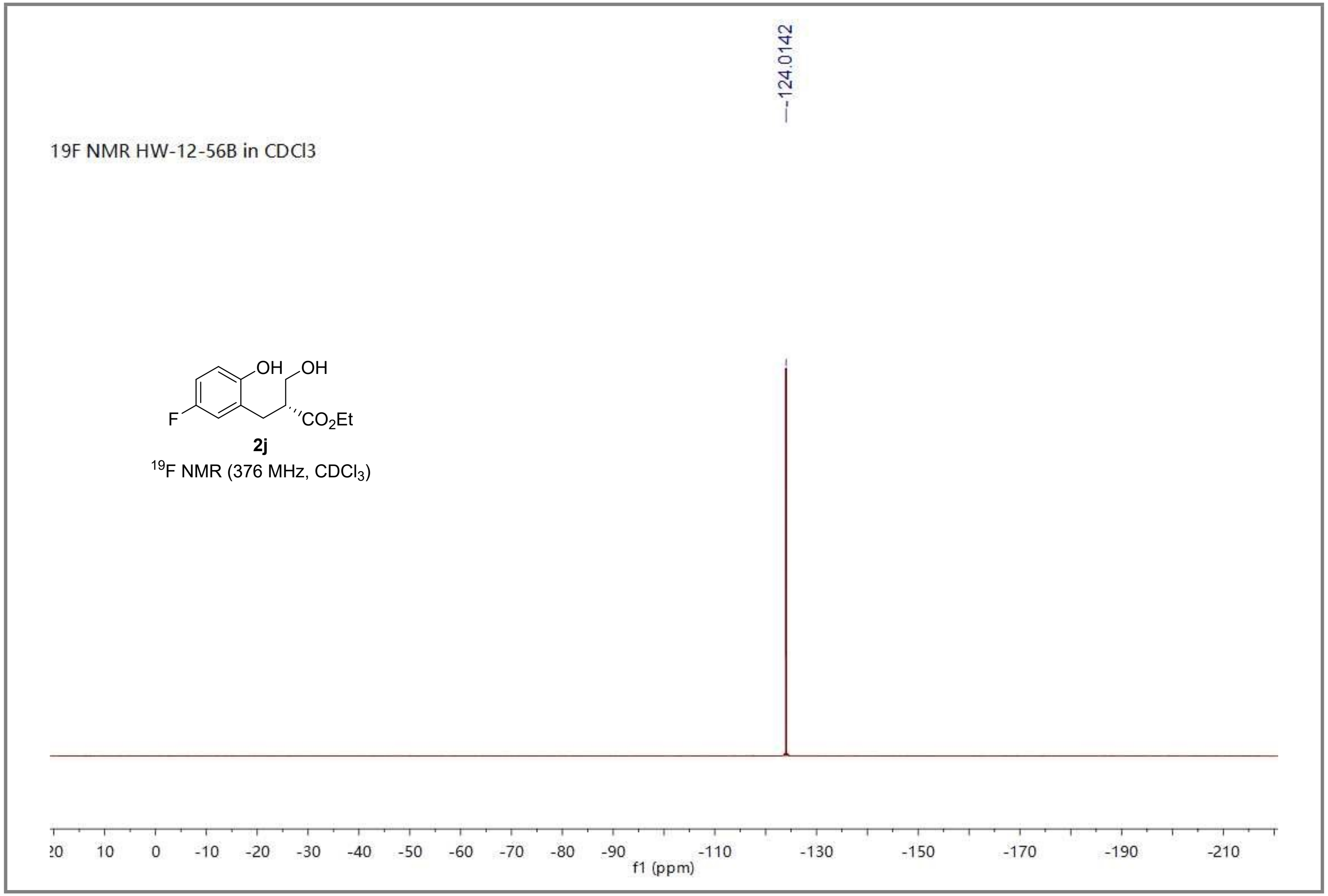

-124.0142
19F NMR HW-12-56B in CDCl3
OH
OH
F
CO2Et
2j
19F NMR (376 MHz, CDCl3)
20 10 0 -10 -20 -30 -40 -50 -60 -70 -80 -90 -110 -130 -150 -170 -190 -210
f1 (ppm)

Data File C:\USERS\PUBLIC\DOCUMENTS\CHEMSTATION\1\DATA\2022\YZH002749 10-18-57.D
Sample Name: HW-12-56B+/-

===

Acq. Operator : SYSTEM
Sample Operator : SYSTEM
Acq. Instrument : HPLC1260 II Location : 1
Injection Date : 11/8/2022 10:18:58 AM Inj : 1
Inj Volume : No inj
Acq. Method : C:\USERS\PUBLIC\DOCUMENTS\CHEMSTATION\1\METHODS\def_LC1.M
Last changed : 11/8/2022 10:03:25 AM by SYSTEM
(modified after loading)
Analysis Method : C:\USERS\PUBLIC\DOCUMENTS\CHEMSTATION\1\METHODS\def_LC1.M
Last changed : 11/29/2023 9:59:58 PM by SYSTEM
(modified after loading)
Sample Info : IB, Hexane/i-PrOH = 90/10, 0.8mL/min, 30 oC, 230 nm

Additional Info : Peak(s) manually integrated

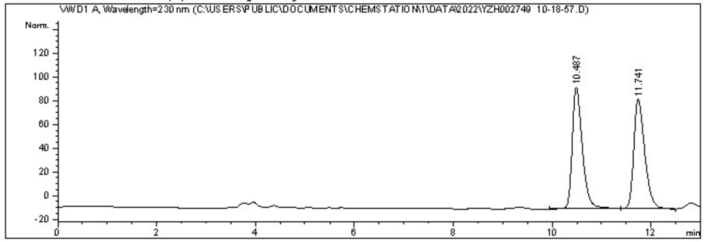

===
Area Percent Report
===

Sorted By : Signal
Multiplier : 1.0000
Dilution : 1.0000
Do not use Multiplier & Dilution Factor with ISTDs

Signal 1: VWD1 A, Wavelength=230 nm

Peak #	RetTime [min]	Type	Width [min]	Area [mAU*s]	Height [mAU]	Area %
1	10.487	BB	0.2155	1166.01672	82.20879	50.6514
2	11.741	BB	0.2330	1136.02637	74.04032	49.3486
Totals :				2302.04309	156.24911	

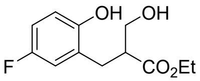

(+/-)-2j

===
*** End of Report ***

HPLC1260 II 11/29/2023 10:00:02 PM SYSTEM Page 1 of 1

Data File C:\USERS\PUBLIC\DOCUMENTS\CHEMSTATION\1\DATA\2022\YZH002750 10-42-31.D
Sample Name: HW-12-56B

===

Acq. Operator : SYSTEM
Sample Operator : SYSTEM
Acq. Instrument : HPLC1260 II Location : 1
Injection Date : 11/8/2022 10:42:32 AM Inj : 1
Inj Volume : No inj
Acq. Method : C:\USERS\PUBLIC\DOCUMENTS\CHEMSTATION\1\METHODS\def_LC1.M
Last changed : 11/8/2022 10:03:25 AM by SYSTEM
(modified after loading)
Analysis Method : C:\USERS\PUBLIC\DOCUMENTS\CHEMSTATION\1\METHODS\def_LC1.M
Last changed : 11/29/2023 9:50:01 PM by SYSTEM
(modified after loading)
Sample Info : IB, Hexane/i-PrOH = 90/10, 0.8mL/min, 30 oC, 230 nm

Additional Info : Peak(s) manually integrated

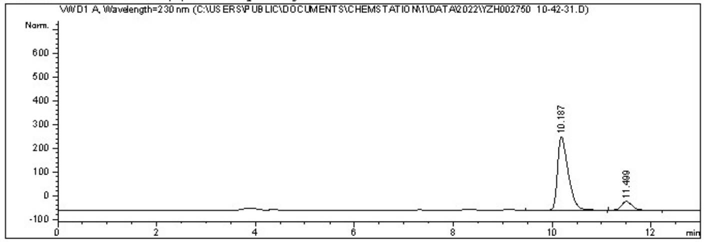

===
Area Percent Report
===

Sorted By : Signal
Multiplier : 1.0000
Dilution : 1.0000
Do not use Multiplier & Dilution Factor with ISTDs

Signal 1: VWD1 A, Wavelength=230 nm

Peak #	RetTime [min]	Type	Width [min]	Area [mAU*s]	Height [mAU]	Area %
1	10.187	VB R	0.2271	2805.53760	186.94838	89.4030
2	11.499	BB	0.2315	332.54401	21.98408	10.5970
Totals :				3138.08160	208.93246	

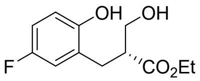

(+)-2j

===
*** End of Report ***

HPLC1260 II 11/29/2023 9:50:05 PM SYSTEM Page 1 of 1

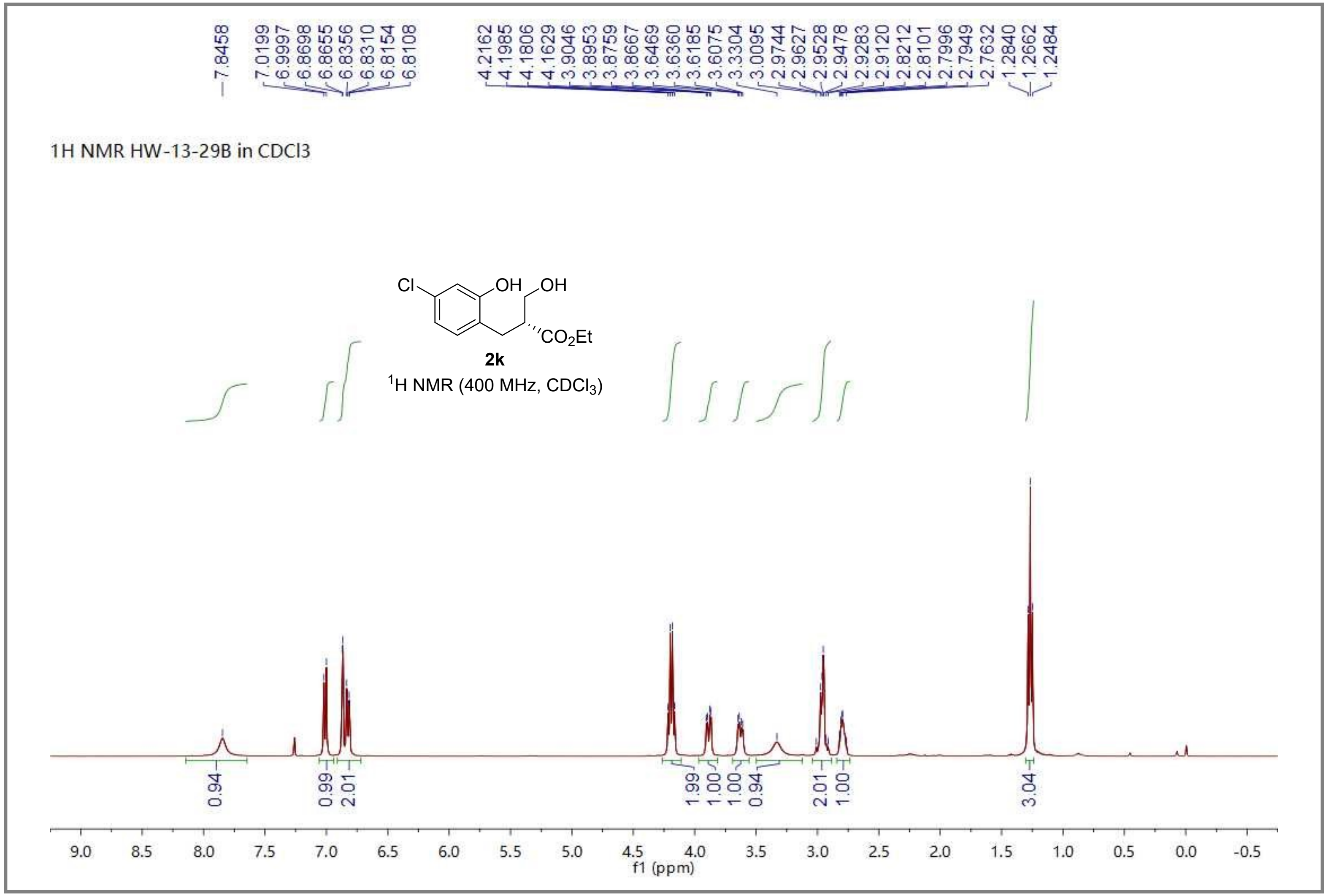

1H NMR HW-13-29B in CDCl3
2k
^{1}H NMR (400 MHz, $CDCl_3$)
7.8458
7.0199
6.9997
6.8698
6.8655
6.8356
6.8310
6.8154
6.8108
4.2162
4.1985
4.1806
4.1629
3.9046
3.8953
3.8759
3.8667
3.6469
3.6360
3.6185
3.6075
3.3304
3.0095
2.9744
2.9627
2.9528
2.9478
2.9283
2.9120
2.8212
2.8101
2.7996
2.7949
2.7632
1.2840
1.2662
1.2484
0.94
0.99
2.01
1.99
1.00
1.00
0.94
2.01
1.00
3.04
f1 (ppm)

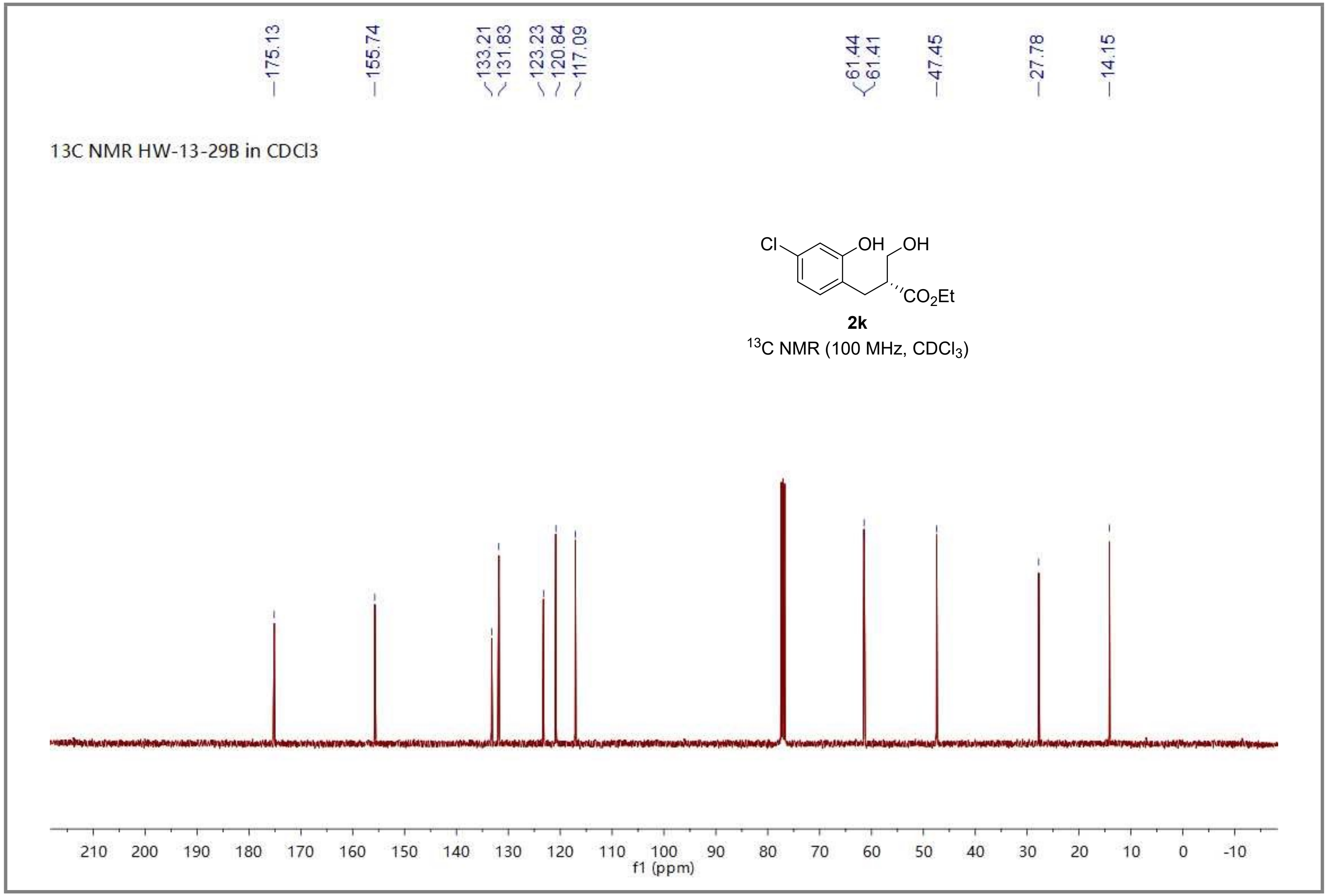
175.13
155.74
133.21
131.83
123.23
120.84
117.09
61.44
61.41
47.45
27.78
14.15
13C NMR HW-13-29B in CDCl3
Cl
OH
OH
CO2Et
2k
13C NMR (100 MHz, CDCl3)
210 200 190 180 170 160 150 140 130 120 110 100 90 80 70 60 50 40 30 20 10 0 -10
f1 (ppm)

Data File C:\CHEM32\1\DATA\ZHOU-22\YZN022314.D
Sample Name: HW-13-29B+/-

===

Acq. Operator :
Acq. Instrument : Instrument 1 Location : -
Injection Date : 1/4/2023 3:46:49 AM
Acq. Method : C:\CHEM32\1\METHODS\DEF_LC.M
Last changed : 1/4/2023 3:44:39 AM
(modified after loading)
Analysis Method : C:\CHEM32\1\METHODS\DEF_LC.M
Last changed : 11/30/2023 8:56:32 AM
(modified after loading)
Sample Info : AS-H, Hexane/i-PrOH = 90/10, 0.8 mL/min, 30 oC, 230 nm

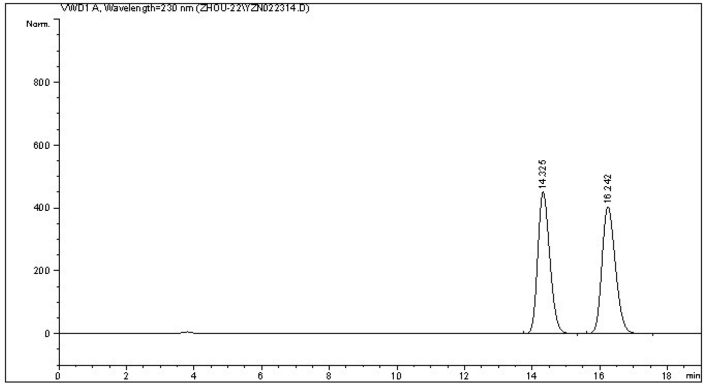

===
Area Percent Report
===

Sorted By : Signal
Multiplier: : 1.0000
Dilution: : 1.0000
Use Multiplier & Dilution Factor with ISTDs

Signal 1: VWD1 A, Wavelength=230 nm

Peak #	RetTime [min]	Type	Width [min]	Area mAU *s	Height [mAU]	Area %
1	14.325	BB	0.3834	1.10519e4	448.83185	49.8610
2	16.242	BB	0.4294	1.11136e4	403.14838	50.1390

Totals : 2.21655e4 851.98022

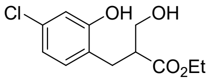

(+/-)-2k

===
*** End of Report ***

Instrument 1 11/30/2023 8:56:36 AM Page 1 of 1

Data File C:\CHEM32\1\DATA\ZHOU-22\YZN022315.D
Sample Name: HW-13-29B

===

Acq. Operator :
Acq. Instrument : Instrument 1 Location : -
Injection Date : 1/4/2023 4:16:05 AM
Acq. Method : C:\CHEM32\1\METHODS\DEF_LC.M
Last changed : 1/4/2023 4:15:23 AM
(modified after loading)
Analysis Method : C:\CHEM32\1\METHODS\DEF_LC.M
Last changed : 10/17/2023 10:25:01 PM
(modified after loading)
Sample Info : AS-H, Hexane/i-PrOH = 90/10, 0.8 mL/min, 30 oC, 230 nm

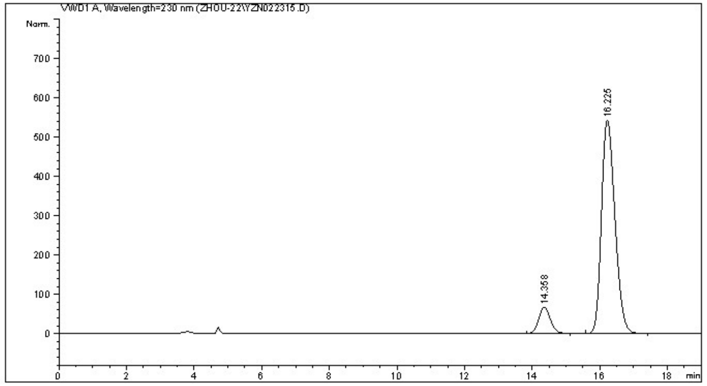

===
Area Percent Report
===

Sorted By : Signal
Multiplier: : 1.0000
Dilution: : 1.0000
Use Multiplier & Dilution Factor with ISTDs

Signal 1: VWD1 A, Wavelength=230 nm

Peak #	RetTime [min]	Type	Width [min]	Area mAU *s	Height [mAU]	Area %
1	14.358	BB	0.3654	1571.00635	66.62236	9.5166
2	16.225	BB	0.4290	1.49370e4	542.43024	90.4834

Totals : 1.65081e4 609.05260

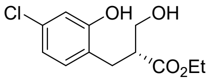

(+)-2k

===
*** End of Report ***

Instrument 1 10/17/2023 10:25:04 PM Page 1 of 1

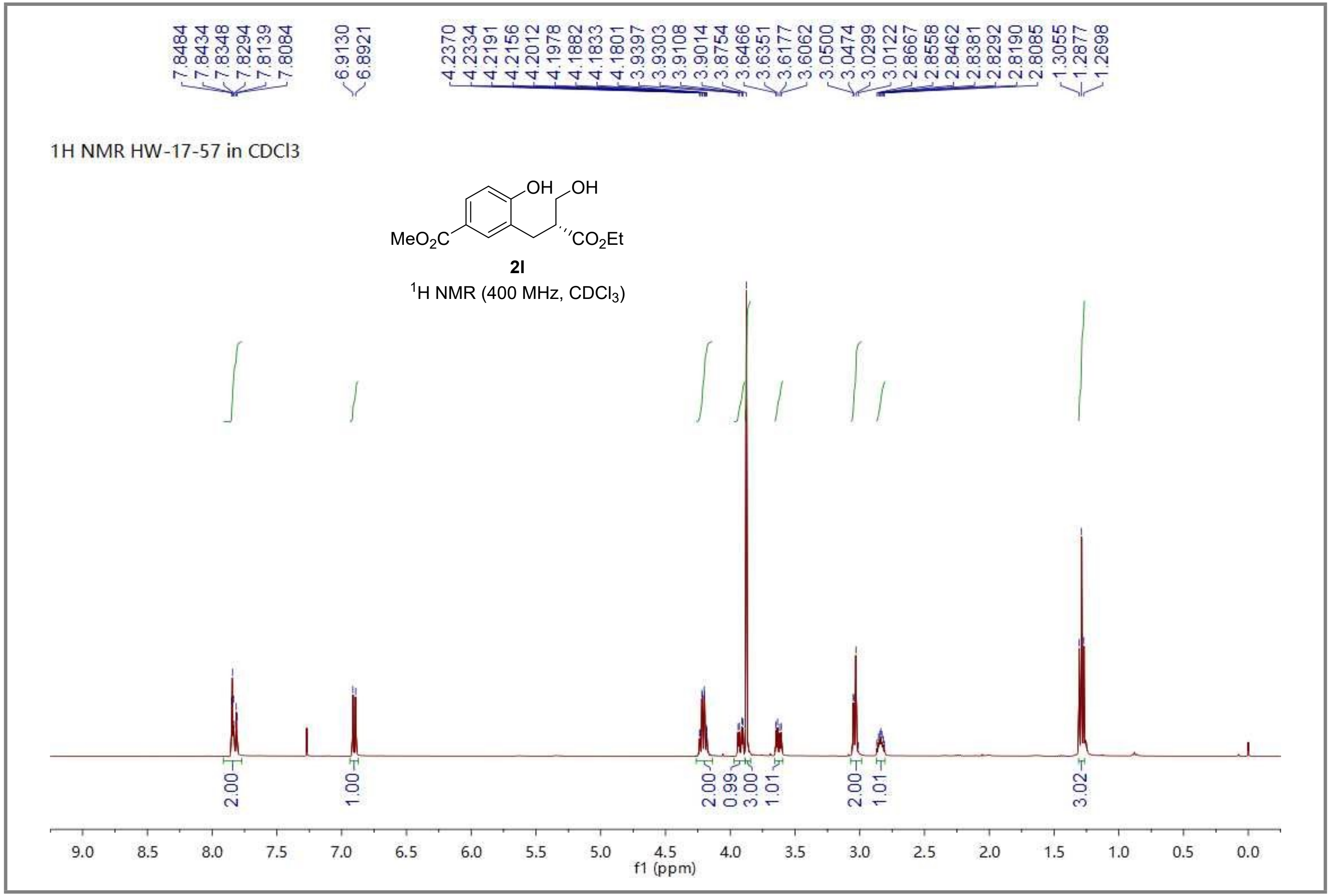
7.8484
7.8434
7.8348
7.8294
7.8139
7.8084
6.9130
6.8921
4.2370
4.2334
4.2191
4.2156
4.2012
4.1978
4.1882
4.1833
4.1801
3.9397
3.9303
3.9108
3.9014
3.8754
3.6466
3.6351
3.6177
3.6062
3.0500
3.0474
3.0299
3.0122
2.8667
2.8558
2.8462
2.8381
2.8292
2.8190
2.8085
1.3055
1.2877
1.2698
1H NMR HW-17-57 in CDCl3
OH
OH
MeO2C
CO2Et
2l
1H NMR (400 MHz, CDCl3)
2.00
1.00
2.00
0.99
3.00
1.01
2.00
1.01
3.02
9.0
8.5
8.0
7.5
7.0
6.5
6.0
5.5
5.0
4.5
4.0
3.5
3.0
2.5
2.0
1.5
1.0
0.5
0.0
f1 (ppm)

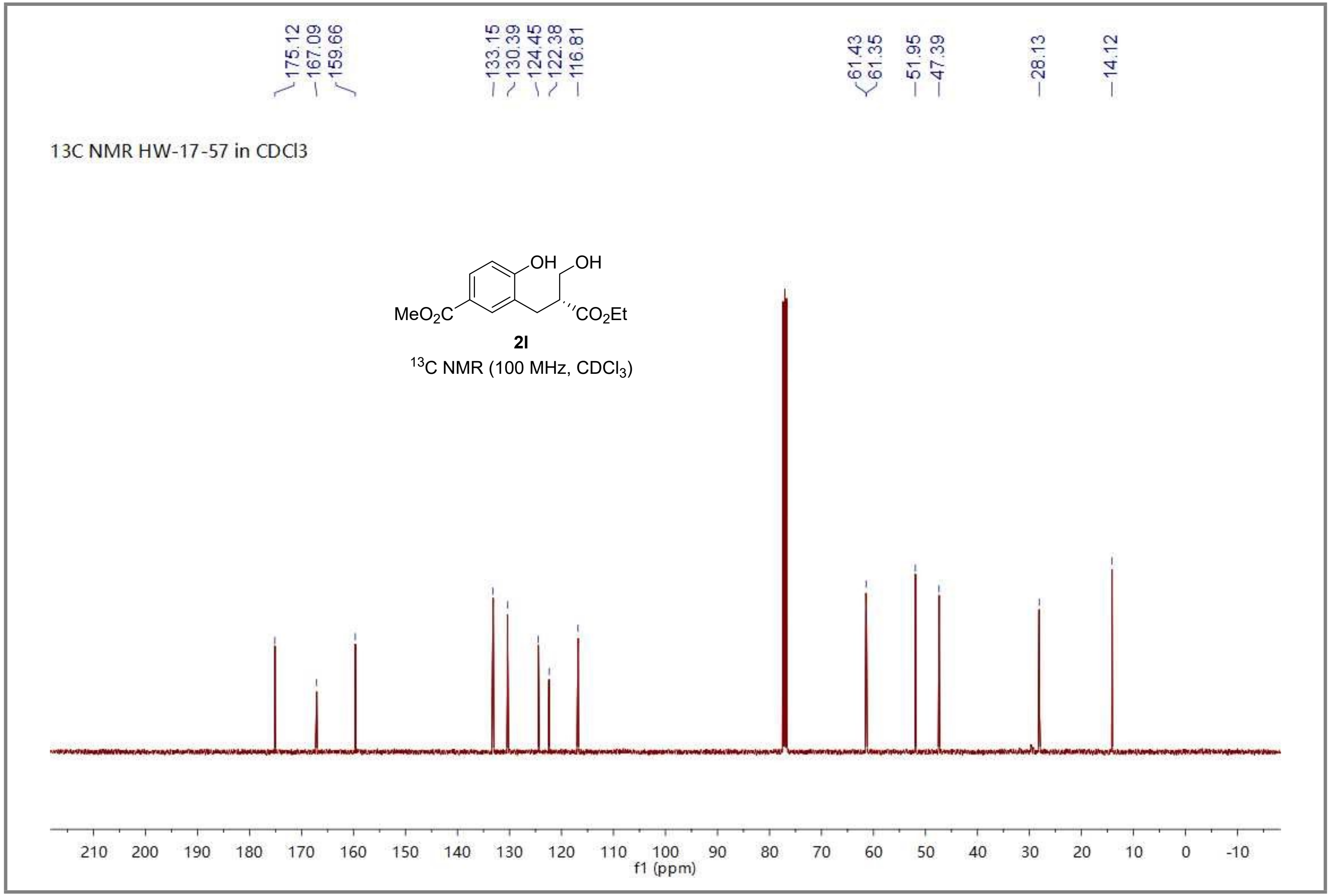
175.12
167.09
159.66
133.15
130.39
124.45
122.38
116.81
61.43
61.35
51.95
47.39
28.13
14.12
13C NMR HW-17-57 in CDCl3
OH
OH
MeO2C
CO2Et
2l
13C NMR (100 MHz, CDCl3)
210 200 190 180 170 160 150 140 130 120 110 100 90 80 70 60 50 40 30 20 10 0 -10
f1 (ppm)

Data File C:\CHEM32\1\DATA\ZHOU-24\YZN025805.D
Sample Name: HW-17-57+/-

===

Acq. Operator :
Acq. Instrument : Instrument 1 Location : -
Injection Date : 5/9/2024 8:51:28 AM
Acq. Method : C:\CHEM32\1\METHODS\DEF_LC.M
Last changed : 5/9/2024 8:50:44 AM
(modified after loading)
Analysis Method : C:\CHEM32\1\METHODS\DEF_LC.M
Last changed : 5/9/2024 9:31:41 AM
(modified after loading)
Sample Info : AS-H, Hexane/iPrOH = 90/10, 0.8 mL/ min, 30 oC, 254 nm.

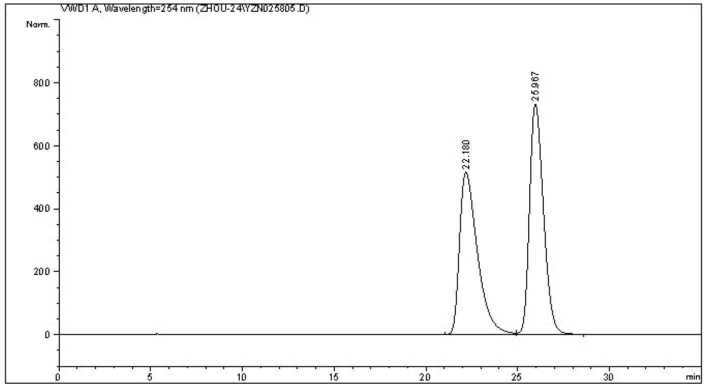

===
Area Percent Report
===

Sorted By : Signal
Multiplier: : 1.0000
Dilution: : 1.0000
Sample Amount: : 1.00000 [ng/ul] (not used in calc.)
Use Multiplier & Dilution Factor with ISTDs

Signal 1: VWD1 A, Wavelength=254 nm

Peak #	RetTime [min]	Type	Width [min]	Area mAU *s	Height [mAU]	Area %
1	22.180	BV	1.0273	3.49369e4	515.97540	47.6093
2	25.967	VB	0.8152	3.84456e4	731.93768	52.3907
Totals :				7.33825e4	1247.91309	

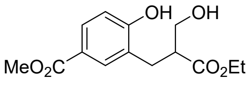

(+/-)-2l

===
*** End of Report ***

Instrument 1 5/9/2024 9:31:44 AM Page 1 of 1

Data File C:\CHEM32\1\DATA\ZHOU-24\YZN025804.D
Sample Name: HW-17-57

===

Acq. Operator :
Acq. Instrument : Instrument 1 Location : -
Injection Date : 5/9/2024 8:14:25 AM
Acq. Method : C:\CHEM32\1\METHODS\DEF_LC.M
Last changed : 5/9/2024 8:06:52 AM
(modified after loading)
Analysis Method : C:\CHEM32\1\METHODS\DEF_LC.M
Last changed : 5/9/2024 9:31:16 AM
(modified after loading)
Sample Info : AS-H, Hexane/iPrOH = 90/10, 0.8 mL/ min, 30 oC, 254 nm.

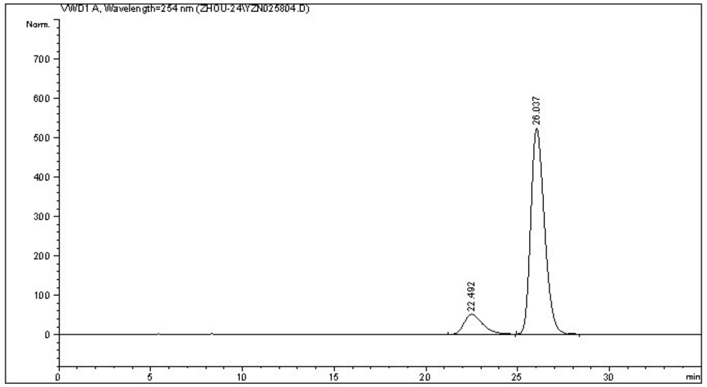

===
Area Percent Report
===

Sorted By : Signal
Multiplier: : 1.0000
Dilution: : 1.0000
Sample Amount: : 1.00000 [ng/ul] (not used in calc.)
Use Multiplier & Dilution Factor with ISTDs

Signal 1: VWD1 A, Wavelength=254 nm

Peak #	RetTime [min]	Type	Width [min]	Area mAU *s	Height [mAU]	Area %
1	22.492	BB	1.0688	3635.62256	51.19103	11.8524
2	26.037	BB	0.8015	2.70386e4	522.73462	88.1476
Totals :				3.06742e4	573.92565	

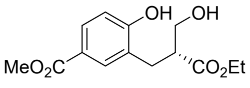

(+)-2l

===
*** End of Report ***

Instrument 1 5/9/2024 9:31:20 AM Page 1 of 1

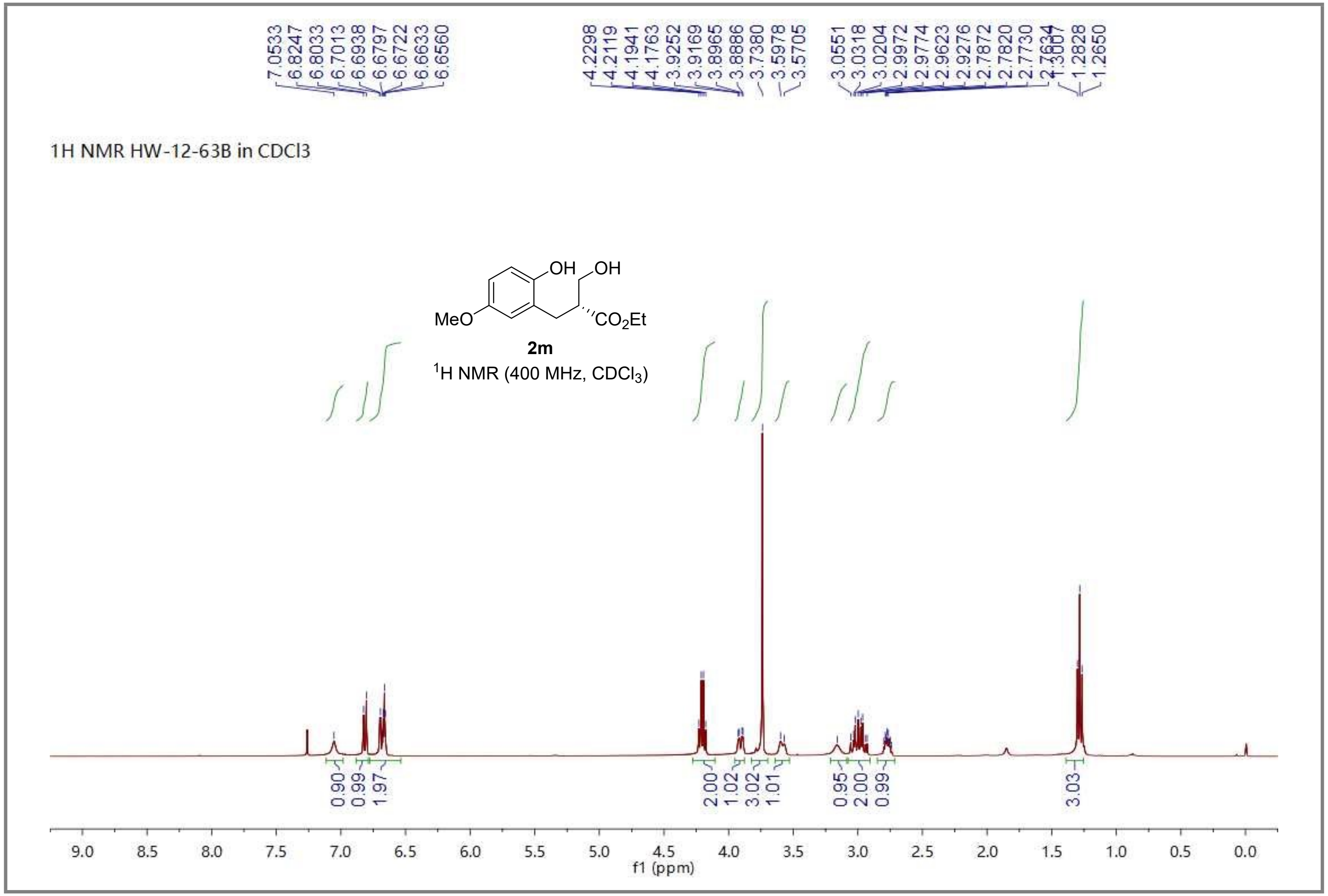

7.0533
6.8247
6.8033
6.7013
6.6938
6.6797
6.6722
6.6633
6.6560
4.2298
4.2119
4.1941
4.1763
3.9252
3.9169
3.8965
3.8886
3.7380
3.5978
3.5705
3.0551
3.0318
3.0204
2.9972
2.9774
2.9623
2.9276
2.7872
2.7820
2.7730
2.7634
1.3007
1.2828
1.2650
1H NMR HW-12-63B in CDCl3
OH
OH
MeO
CO2Et
2m
1H NMR (400 MHz, CDCl3)
0.90
0.99
1.97
2.00
1.02
3.02
1.01
0.95
2.00
0.99
3.03
9.0
8.5
8.0
7.5
7.0
6.5
6.0
5.5
5.0
4.5
4.0
3.5
3.0
2.5
2.0
1.5
1.0
0.5
0.0
f1 (ppm)

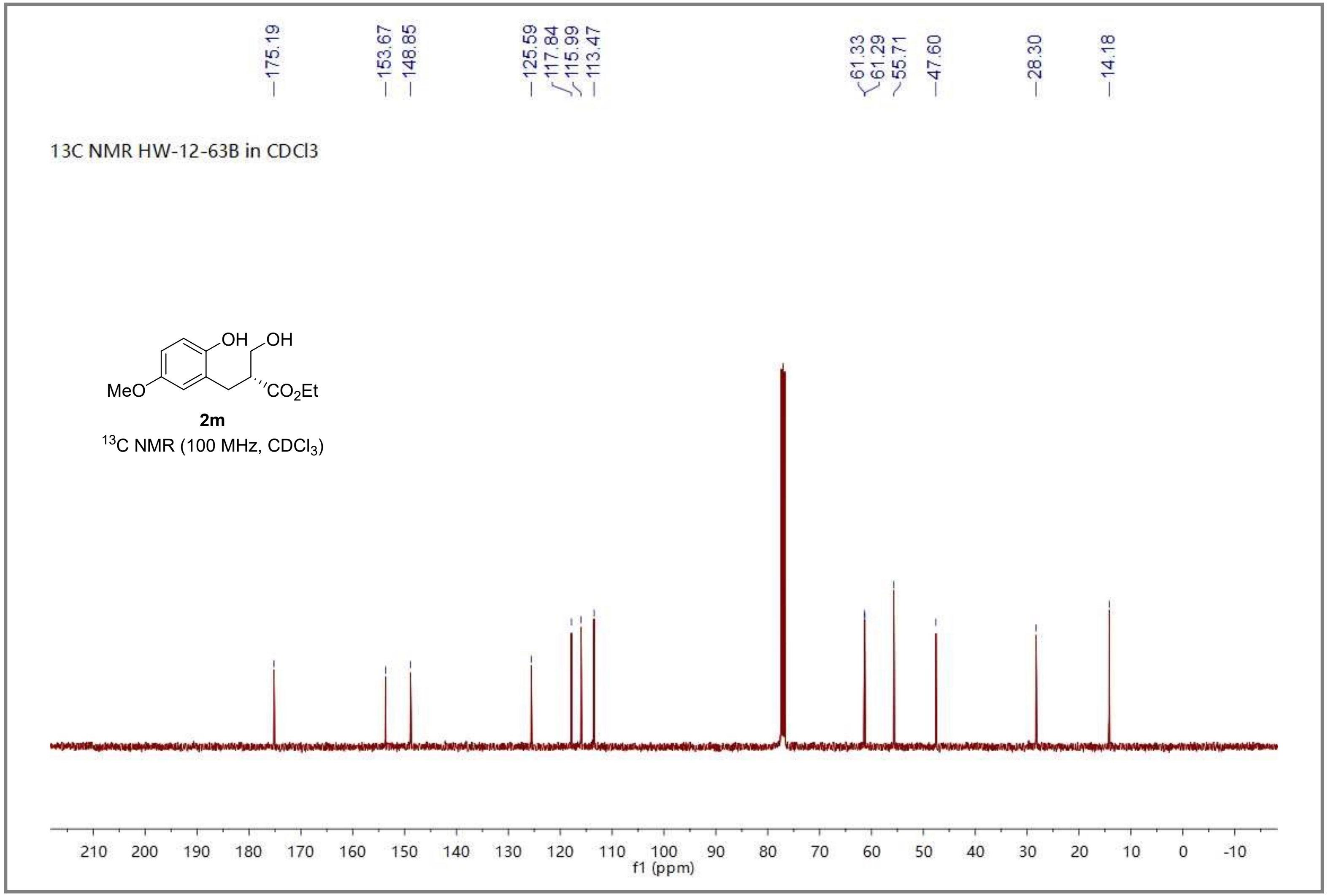
175.19
153.67
148.85
125.59
117.84
115.99
113.47
61.33
61.29
55.71
47.60
28.30
14.18
13C NMR HW-12-63B in CDCl3
OH
OH
MeO
CO2Et
2m
13C NMR (100 MHz, CDCl3)
210 200 190 180 170 160 150 140 130 120 110 100 90 80 70 60 50 40 30 20 10 0 -10
f1 (ppm)

Data File C:\USERS\PUBLIC\DOCUMENTS\CHEMSTATION\1\DATA\2022\YZH002818 20-41-43.D
Sample Name: HW-12-63B+/-

===

Acq. Operator : SYSTEM
Sample Operator : SYSTEM
Acq. Instrument : HPLC1260 II Location : 1
Injection Date : 11/14/2022 8:41:43 PM Inj : 1
Inj Volume : No inj
Acq. Method : C:\USERS\PUBLIC\DOCUMENTS\CHEMSTATION\1\METHODS\def_LC1.M
Last changed : 11/14/2022 8:05:07 PM by SYSTEM
(modified after loading)
Analysis Method : C:\USERS\PUBLIC\DOCUMENTS\CHEMSTATION\1\METHODS\def_LC1.M
Last changed : 10/17/2023 10:42:41 AM by SYSTEM
(modified after loading)
Sample Info : AS-H, Hexane/i-PrOH = 90/10, 0.8 mL/min, 30 oC, 230 nm

Additional Info : Peak(s) manually integrated

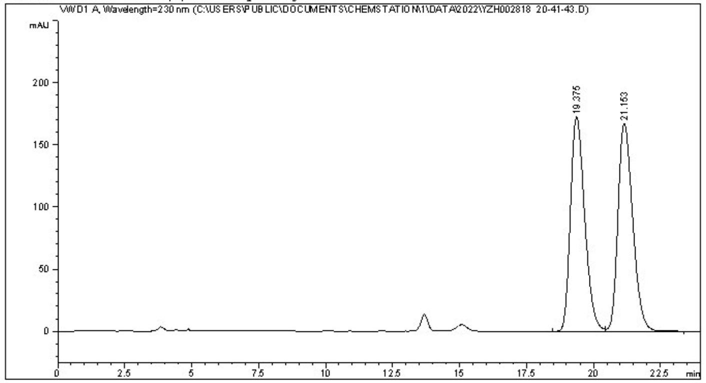

===
Area Percent Report
===

Sorted By : Signal
Multiplier : 1.0000
Dilution : 1.0000
Do not use Multiplier & Dilution Factor with ISTDs

Signal 1: VWD1 A, Wavelength=230 nm

Peak #	RetTime [min]	Type	Width [min]	Area [mAU*s]	Height [mAU]	Area %
1	19.375	BV	0.5713	6360.90137	172.46037	49.8071
2	21.153	VB	0.5951	6410.17627	167.28079	50.1929

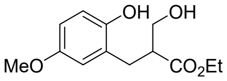

(+/-)-2m

HPLC1260 II 10/17/2023 10:42:46 AM SYSTEM Page 1 of 2

Data File C:\USERS\PUBLIC\DOCUMENTS\CHEMSTATION\1\DATA\2022\YZH002819 21-06-43.D
Sample Name: HW-12-63B

===

Acq. Operator : SYSTEM
Sample Operator : SYSTEM
Acq. Instrument : HPLC1260 II Location : 1
Injection Date : 11/14/2022 9:06:43 PM Inj : 1
Inj Volume : No inj
Acq. Method : C:\USERS\PUBLIC\DOCUMENTS\CHEMSTATION\1\METHODS\def_LC1.M
Last changed : 11/14/2022 8:05:07 PM by SYSTEM
(modified after loading)
Analysis Method : C:\USERS\PUBLIC\DOCUMENTS\CHEMSTATION\1\METHODS\def_LC1.M
Last changed : 10/17/2023 10:42:41 AM by SYSTEM
(modified after loading)
Sample Info : AS-H, Hexane/i-PrOH = 90/10, 0.8 mL/min, 30 oC, 230 nm

Additional Info : Peak(s) manually integrated

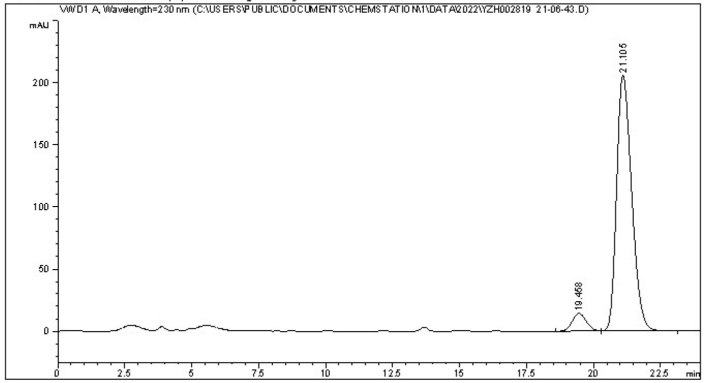

===
Area Percent Report
===

Sorted By : Signal
Multiplier : 1.0000
Dilution : 1.0000
Do not use Multiplier & Dilution Factor with ISTDs

Signal 1: VWD1 A, Wavelength=230 nm

Peak #	RetTime [min]	Type	Width [min]	Area [mAU*s]	Height [mAU]	Area %
1	19.458	BB	0.5670	513.02692	14.18275	6.0063
2	21.105	BB	0.6060	8028.38330	205.41820	93.9937

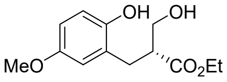

(+)-2m

HPLC1260 II 10/17/2023 10:43:23 AM SYSTEM Page 1 of 2

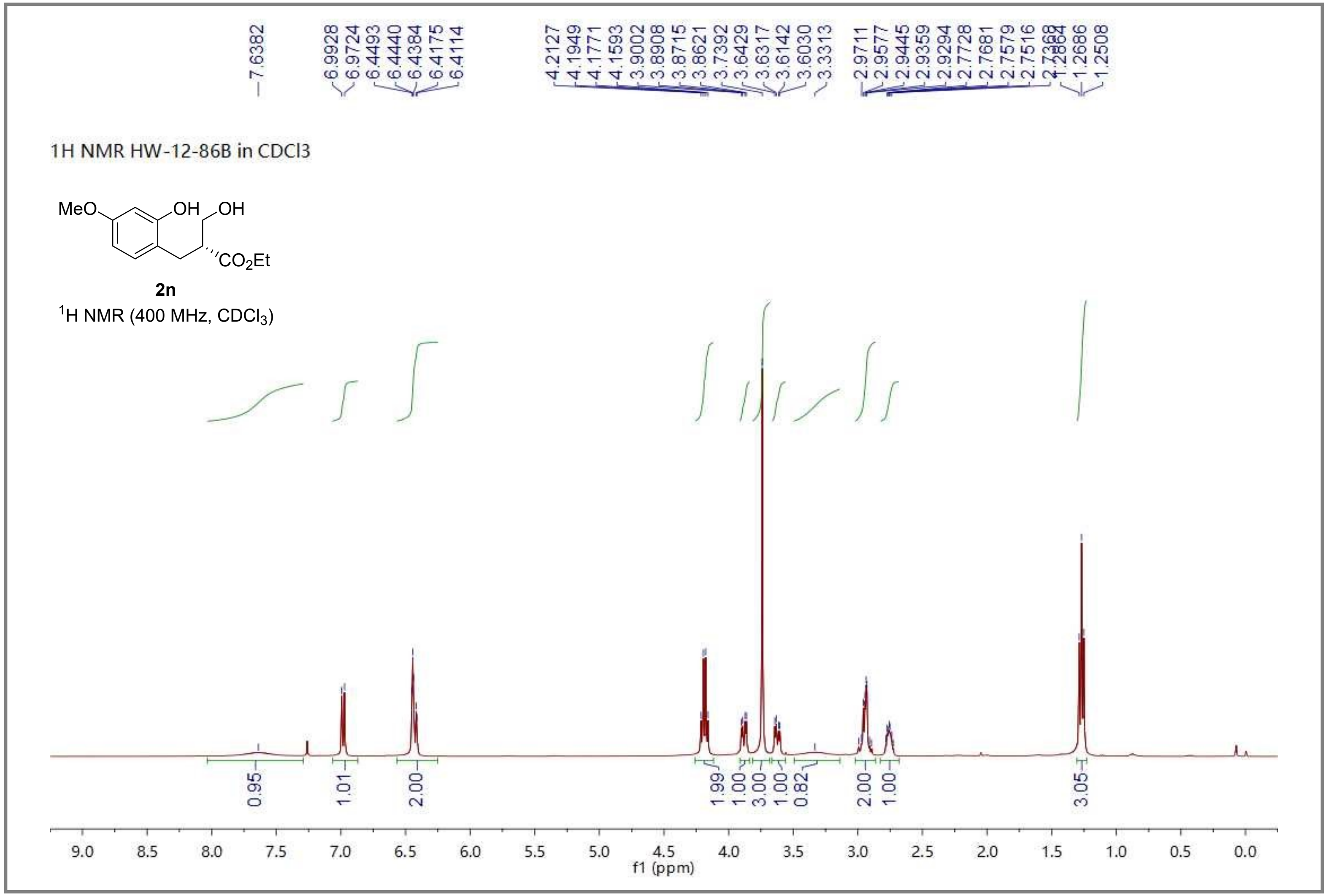
1H NMR HW-12-86B in CDCl3
MeO
OH
OH
CO2Et
2n
1H NMR (400 MHz, CDCl3)
f1 (ppm)

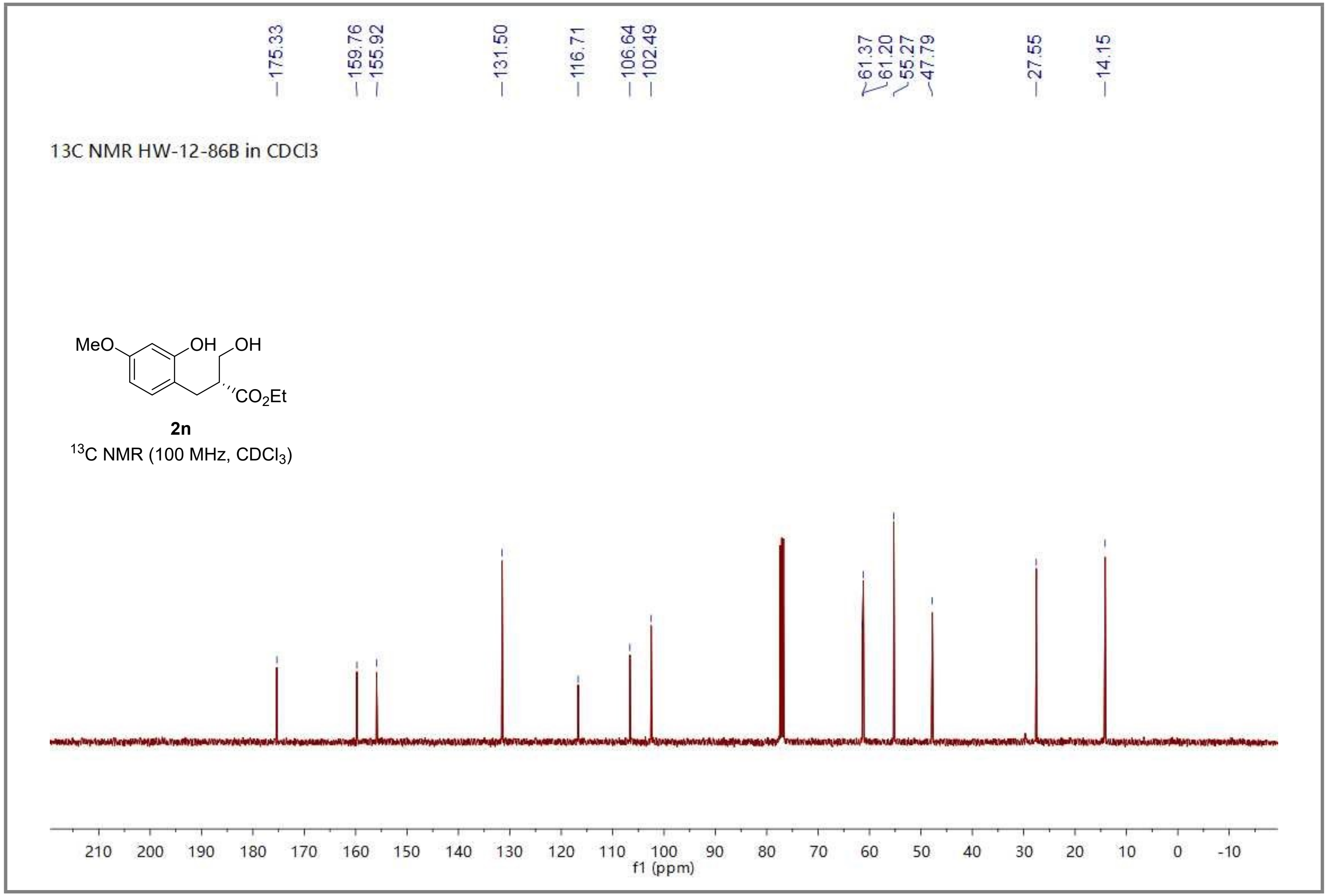
175.33
159.76
155.92
131.50
116.71
106.64
102.49
61.37
61.20
55.27
47.79
27.55
14.15
13C NMR HW-12-86B in CDCl3
MeO
OH
OH
CO2Et
2n
13C NMR (100 MHz, CDCl3)
210 200 190 180 170 160 150 140 130 120 110 100 90 80 70 60 50 40 30 20 10 0 -10
f1 (ppm)

```
Data File C:\CHEM32\1\DATA\ZHOU-22\YZN022169.D
Sample Name: HW-12-86B+/-

    =====================================================================
    Acq. Operator   :
    Acq. Instrument : Instrument 1                    Location :   -
    Injection Date  : 12/5/2022 9:44:16 PM
    Acq. Method     : C:\CHEM32\1\METHODS\DEF_LC.M
    Last changed    : 12/5/2022 9:43:45 PM
                      (modified after loading)
    Analysis Method : C:\CHEM32\1\METHODS\DEF_LC.M
    Last changed    : 10/17/2023 10:35:13 PM
                      (modified after loading)
    Sample Info     : IB, Hexane/i-PrOH = 95/5, 0.8 mL/min, 30 oC, 230 nm
```

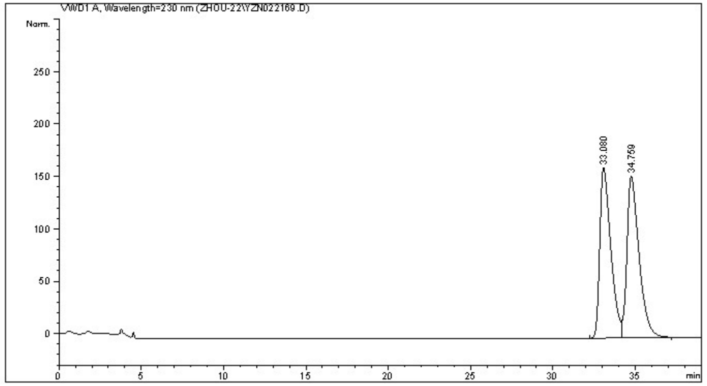


```
    =====================================================================
                         Area Percent Report
    =====================================================================

    Sorted By             :      Signal
    Multiplier:                  :      1.0000
    Dilution:                    :      1.0000
    Use Multiplier & Dilution Factor with ISTDs


    Signal 1: VWD1 A, Wavelength=230 nm

    Peak RetTime Type  Width     Area      Height     Area
      #   [min]        [min]  mAU   *s    [mAU  ]      %
    ----|-------|----|-------|----------|----------|--------|
       1  33.080 BV    0.6963 7529.39307  162.53297  48.7565
       2  34.759 VB    0.7740 7913.44580  154.42337  51.2435

    Totals :                  1.54428e4   316.95634

    =====================================================================
                          *** End of Report ***
```

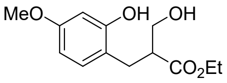

(+/-)-2n

Instrument 1 10/17/2023 10:36:31 PM Page 1 of 1

```
Data File C:\CHEM32\1\DATA\ZHOU-22\YZN022170.D
Sample Name: HW-12-86

    =====================================================================
    Acq. Operator   :
    Acq. Instrument : Instrument 1                    Location :   -
    Injection Date  : 12/5/2022 10:24:58 PM
    Acq. Method     : C:\CHEM32\1\METHODS\DEF_LC.M
    Last changed    : 12/5/2022 10:24:08 PM
                      (modified after loading)
    Analysis Method : C:\CHEM32\1\METHODS\DEF_LC.M
    Last changed    : 10/17/2023 10:39:39 PM
                      (modified after loading)
    Sample Info     : IB, Hexane/i-PrOH = 95/5, 0.8 mL/min, 30 oC, 230 nm
```

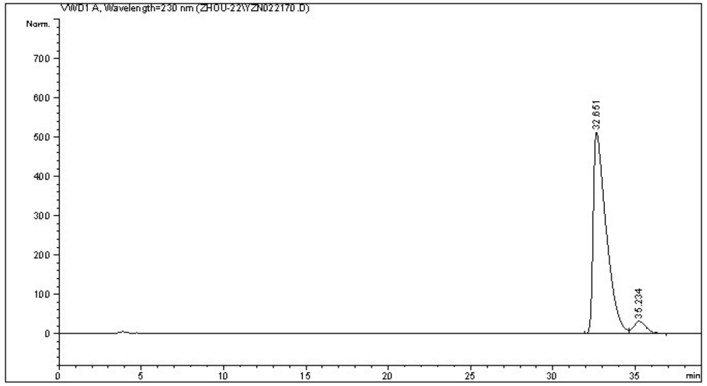


```
    =====================================================================
                         Area Percent Report
    =====================================================================

    Sorted By             :      Signal
    Multiplier:                  :      1.0000
    Dilution:                    :      1.0000
    Use Multiplier & Dilution Factor with ISTDs


    Signal 1: VWD1 A, Wavelength=230 nm

    Peak RetTime Type  Width     Area      Height     Area
      #   [min]        [min]  mAU   *s    [mAU  ]      %
    ----|-------|----|-------|----------|----------|--------|
       1  32.651 BV    0.7691 2.80348e4  512.62573  94.5243
       2  35.234 VB    0.7861 1624.01208   31.21306   5.4757

    Totals :                  2.96588e4   543.83879

    =====================================================================
                          *** End of Report ***
```

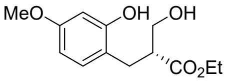

(+)-2n

Instrument 1 10/17/2023 10:39:42 PM Page 1 of 1

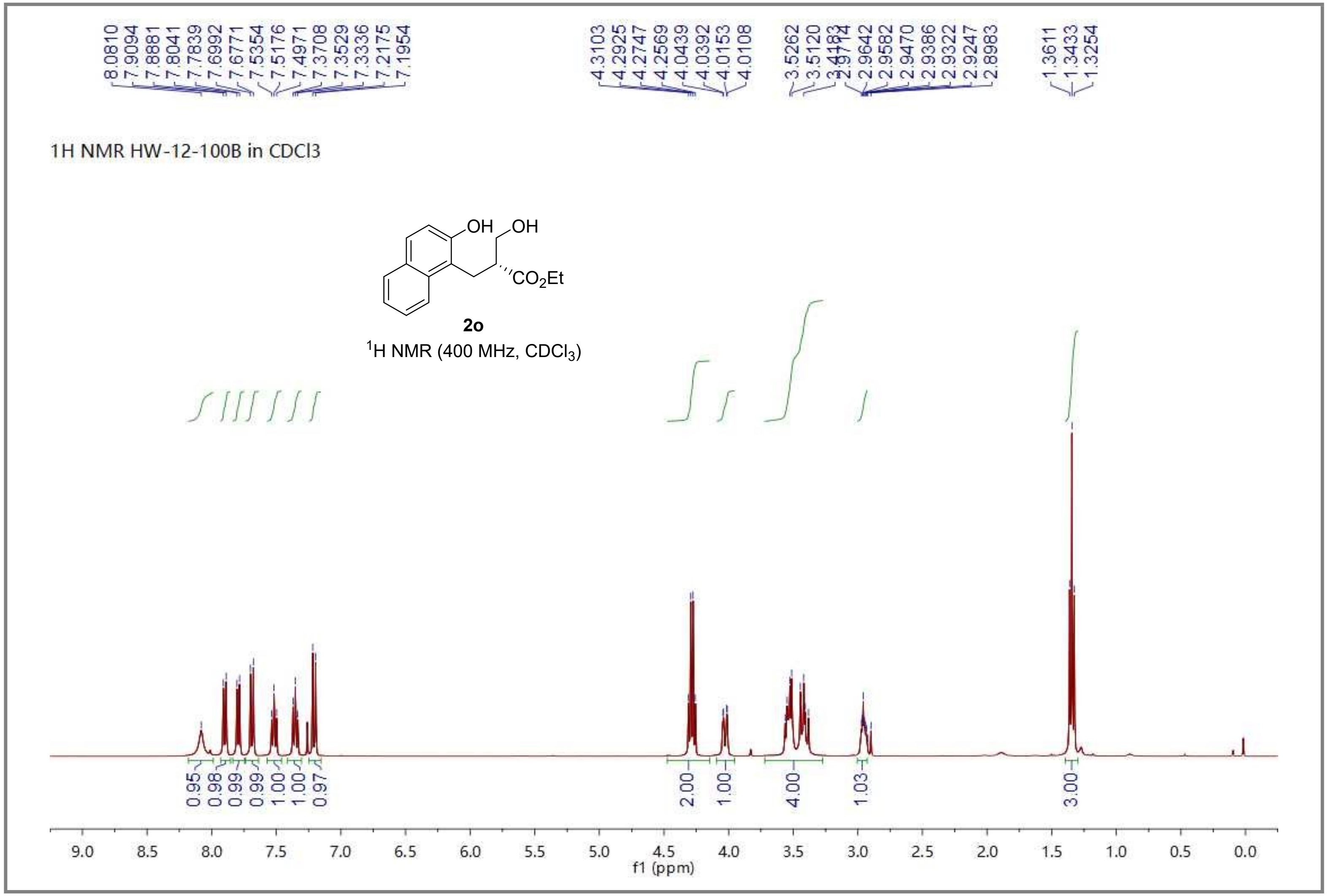

8.0810
7.9094
7.8881
7.8041
7.7839
7.6992
7.6771
7.5354
7.5176
7.4971
7.3708
7.3529
7.3336
7.2175
7.1954
4.3103
4.2925
4.2747
4.2569
4.0439
4.0392
4.0153
4.0108
3.5262
3.5120
2.9642
2.9582
2.9470
2.9386
2.9322
2.9247
2.8983
1.3611
1.3433
1.3254
1H NMR HW-12-100B in CDCl3
OH
OH
CO_2Et
2o
^1H NMR (400 MHz, $CDCl_3$)
0.95
0.98
0.99
0.99
1.00
1.00
0.97
2.00
1.00
4.00
1.03
3.00
9.0
8.5
8.0
7.5
7.0
6.5
6.0
5.5
5.0
4.5
4.0
3.5
3.0
2.5
2.0
1.5
1.0
0.5
0.0
f1 (ppm)

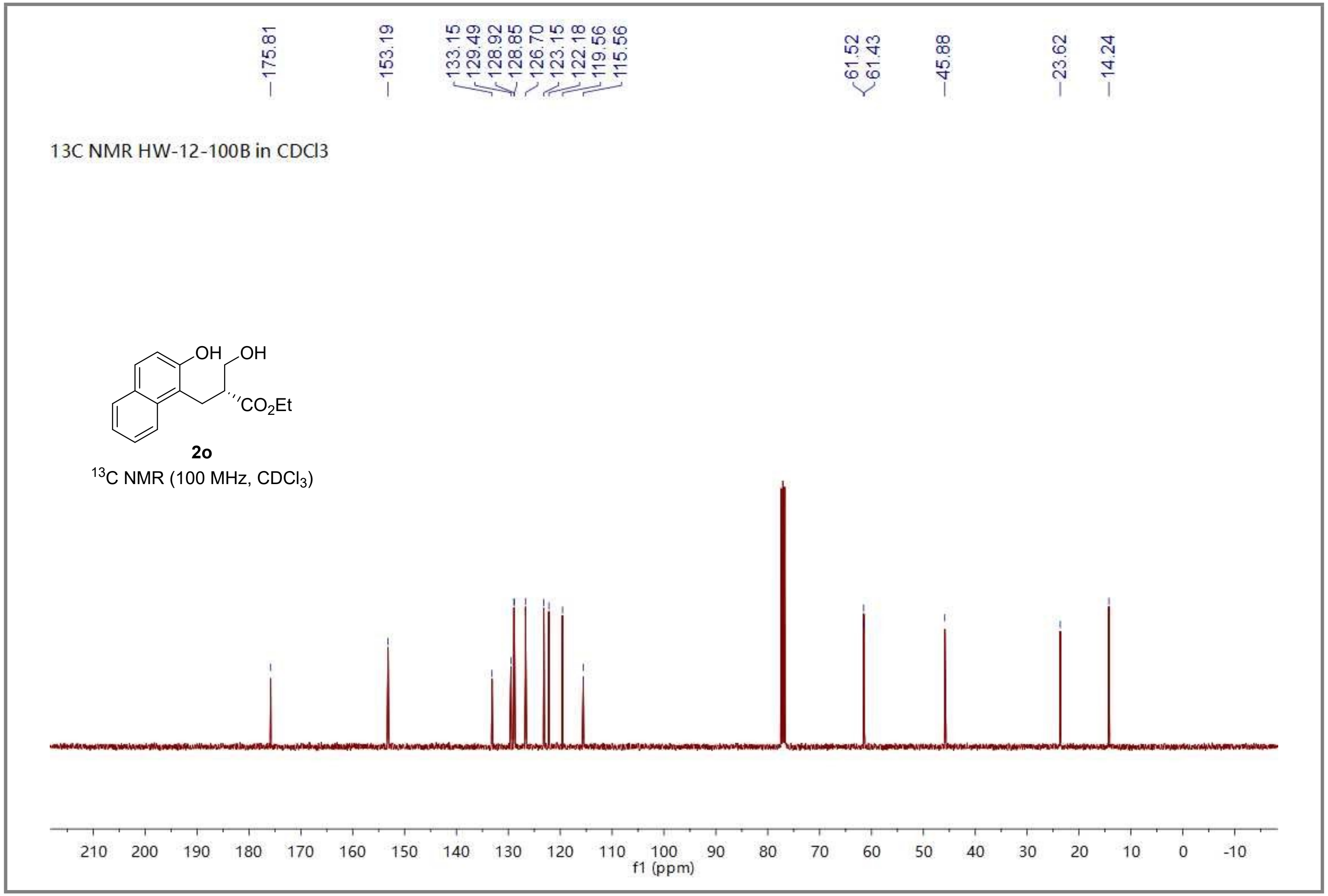

175.81
153.19
133.15
129.49
128.92
128.85
126.70
123.15
122.18
119.56
115.56
61.52
61.43
45.88
23.62
14.24
13C NMR HW-12-100B in CDCl3
OH
OH
CO2Et
2o
13C NMR (100 MHz, CDCl3)
210 200 190 180 170 160 150 140 130 120 110 100 90 80 70 60 50 40 30 20 10 0 -10
f1 (ppm)

Data File C:\USERS\PUBLIC\DOCUMENTS\CHEMSTATION\1\DATA\2022\YZH003090 17-00-20.D
Sample Name: HW-12-100B+/-

===

Acq. Operator : SYSTEM
Sample Operator : SYSTEM
Acq. Instrument : HPLC1260 II Location : 1
Injection Date : 12/20/2022 5:00:20 PM Inj : 1
Inj Volume : No inj
Acq. Method : C:\USERS\PUBLIC\DOCUMENTS\CHEMSTATION\1\METHODS\def_LC1.M
Last changed : 12/20/2022 3:36:44 PM by SYSTEM
(modified after loading)
Analysis Method : C:\USERS\PUBLIC\DOCUMENTS\CHEMSTATION\1\METHODS\def_LC1.M
Last changed : 10/17/2023 10:56:22 AM by SYSTEM
(modified after loading)
Sample Info : AS-H, Hexane/i-PrOH = 90/10, 0.8 mL/min, 30 oC, 230 nm

Additional Info : Peak(s) manually integrated

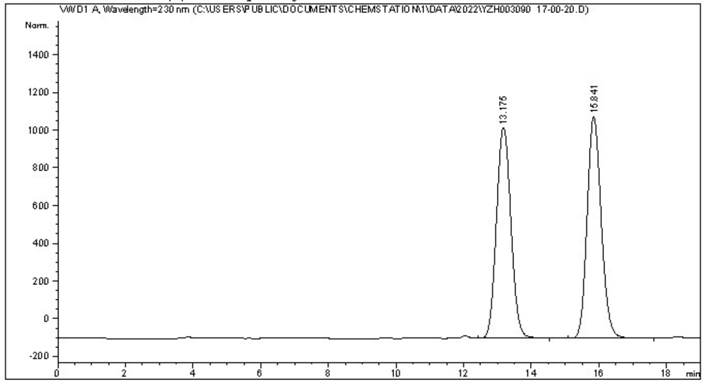

===
Area Percent Report
===

Sorted By : Signal
Multiplier : 1.0000
Dilution : 1.0000
Do not use Multiplier & Dilution Factor with ISTDs

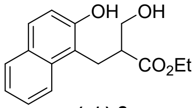

(+/-)-2o

Signal 1: VWD1 A, Wavelength=230 nm

Peak #	RetTime [min]	Type	Width [min]	Area [mAU*s]	Height [mAU]	Area %
1	13.175	BB	0.4682	2.73494e4	918.88074	49.3684
2	15.841	BB	0.4508	2.80491e4	965.84302	50.6316

Data File C:\USERS\PUBLIC\DOCUMENTS\CHEMSTATION\1\DATA\2022\YZH003092 18-42-40.D
Sample Name: HW-12-100B

===

Acq. Operator : SYSTEM
Sample Operator : SYSTEM
Acq. Instrument : HPLC1260 II Location : 1
Injection Date : 12/20/2022 6:42:40 PM Inj : 1
Inj Volume : No inj
Acq. Method : C:\USERS\PUBLIC\DOCUMENTS\CHEMSTATION\1\METHODS\def_LC1.M
Last changed : 12/20/2022 6:41:56 PM by SYSTEM
(modified after loading)
Analysis Method : C:\USERS\PUBLIC\DOCUMENTS\CHEMSTATION\1\METHODS\def_LC1.M
Last changed : 10/17/2023 10:57:18 AM by SYSTEM
(modified after loading)
Sample Info : AS-H, Hexane/i-PrOH = 90/10, 0.8 mL/min, 30 oC, 230 nm

Additional Info : Peak(s) manually integrated

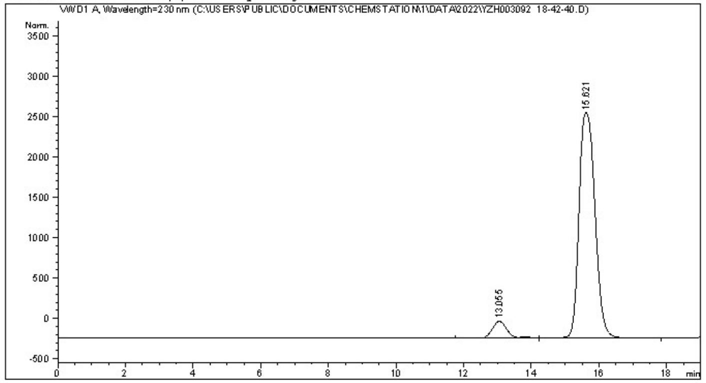

===
Area Percent Report
===

Sorted By : Signal
Multiplier : 1.0000
Dilution : 1.0000
Do not use Multiplier & Dilution Factor with ISTDs

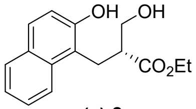

(+)-2o

Signal 1: VWD1 A, Wavelength=230 nm

Peak #	RetTime [min]	Type	Width [min]	Area [mAU*s]	Height [mAU]	Area %
1	13.055	VV R	0.4646	5408.42383	172.75548	6.6842
2	15.621	VB	0.5206	7.55050e4	2302.19604	93.3158

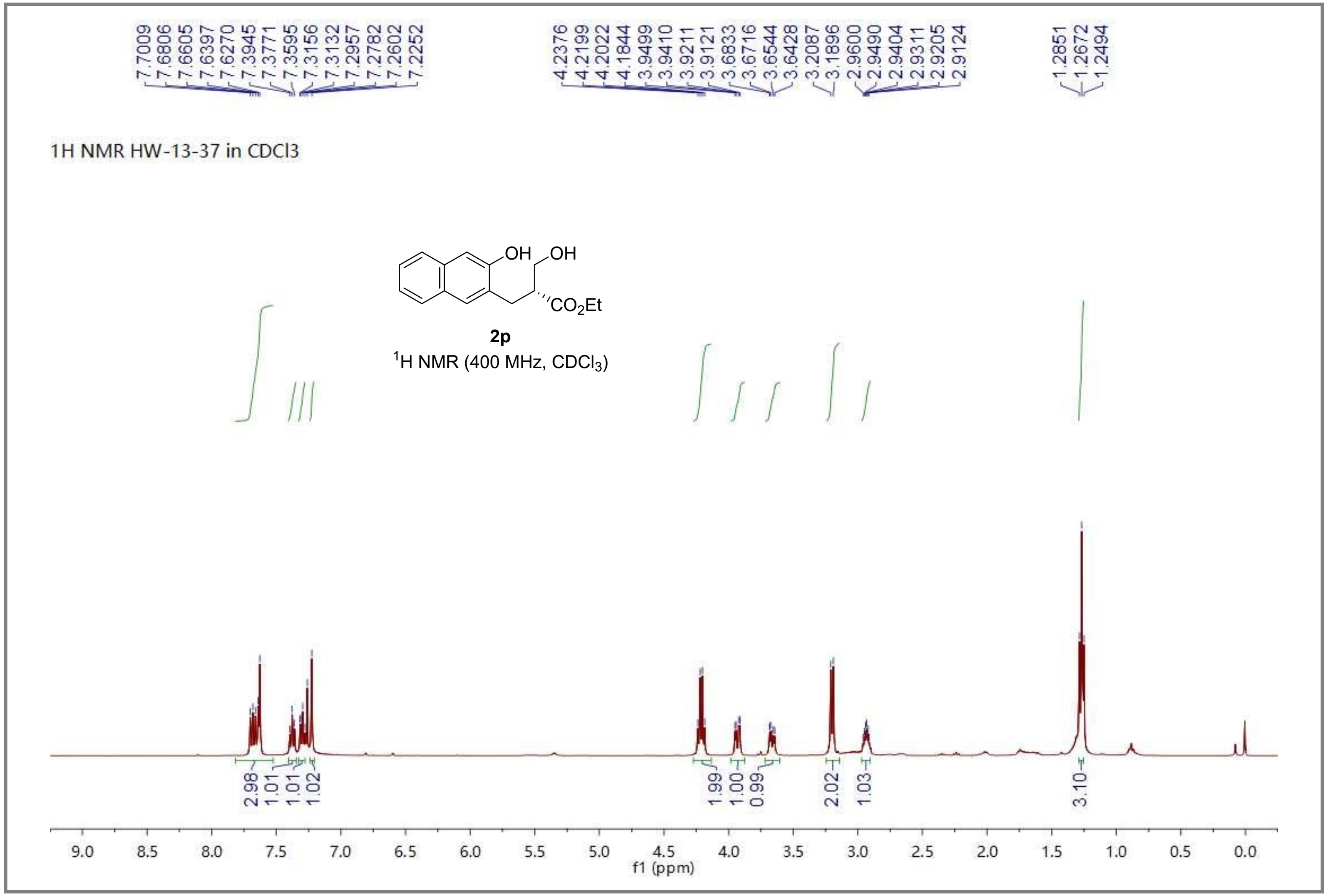
7.7009
7.6806
7.6605
7.6397
7.6270
7.3945
7.3771
7.3595
7.3156
7.3132
7.2957
7.2782
7.2602
7.2252
4.2376
4.2199
4.2022
4.1844
3.9499
3.9410
3.9211
3.9121
3.6833
3.6716
3.6544
3.6428
3.2087
3.1896
2.9600
2.9490
2.9404
2.9311
2.9205
2.9124
1.2851
1.2672
1.2494
1H NMR HW-13-37 in CDCl3
OH
OH
CO2Et
2p
1H NMR (400 MHz, CDCl3)
2.98
1.01
1.01
1.02
1.99
1.00
0.99
2.02
1.03
3.10
9.0
8.5
8.0
7.5
7.0
6.5
6.0
5.5
5.0
4.5
4.0
3.5
3.0
2.5
2.0
1.5
1.0
0.5
0.0
f1 (ppm)

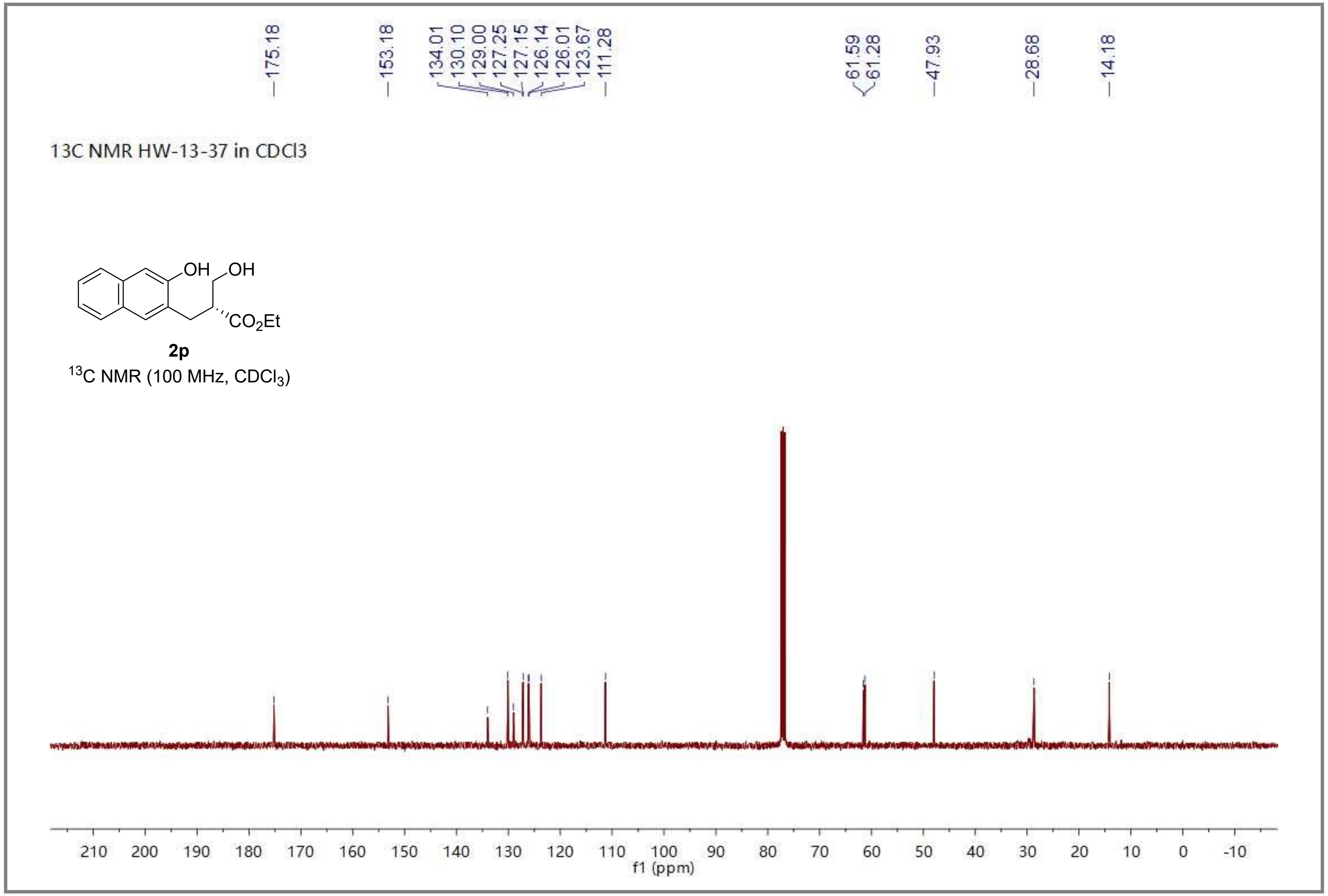

175.18
153.18
134.01
130.10
129.00
127.25
127.15
126.14
126.01
123.67
111.28
61.59
61.28
47.93
28.68
14.18
13C NMR HW-13-37 in CDCl3
OH
OH
CO2Et
2p
13C NMR (100 MHz, CDCl3)
210 200 190 180 170 160 150 140 130 120 110 100 90 80 70 60 50 40 30 20 10 0 -10
f1 (ppm)

Data File D:\USERS\CHEMSTATION\1\DATA\ZHOU2022\YZ3006114 19-22-43.D
Sample Name: HW-13-37+/-

===

Acq. Operator : SYSTEM
Sample Operator : SYSTEM
Acq. Instrument : 1260II   Location : -
Injection Date : 1/9/2023 7:22:43 PM   Inj : 1
Inj Volume : No inj
Acq. Method : C:\Users\Public\Documents\ChemStation\1\Methods\def_LC.M
Last changed : 1/9/2023 7:07:57 PM by SYSTEM
(modified after loading)
Analysis Method : C:\Users\Public\Documents\ChemStation\1\Methods\def_LC.M
Last changed : 10/17/2023 10:51:55 AM by SYSTEM
(modified after loading)
Sample Info : IB, n-Hexane/i-PrOH = 90/10, 0.8 mL/min, 30 oC, 230 nm

Additional Info : Peak(s) manually integrated

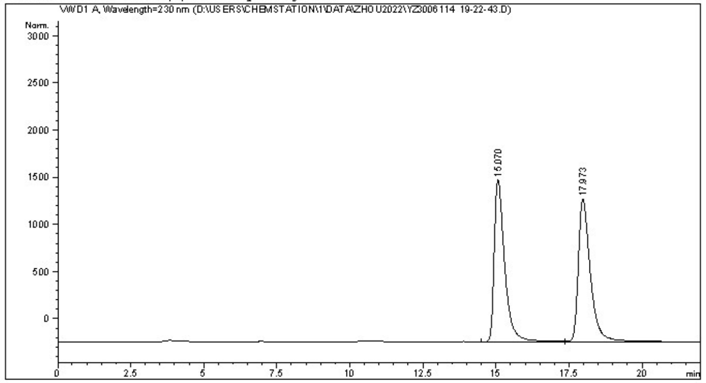

===
Area Percent Report
===

Sorted By : Signal
Multiplier : 1.0000
Dilution : 1.0000
Do not use Multiplier & Dilution Factor with ISTDs

Signal 1: VWD1 A, Wavelength=230 nm

Peak #	RetTime [min]	Type	Width [min]	Area [mAU*s]	Height [mAU]	Area %
1	15.070	BV	0.3706	3.05775e4	1231.83813	49.7197
2	17.973	VB	0.4277	3.09223e4	1084.37146	50.2803

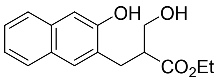

(+/-)-2p

1260II 10/17/2023 10:52:25 AM SYSTEM

Page 1 of 2

Data File D:\USERS\CHEMSTATION\1\DATA\ZHOU2022\YZ3006115 19-47-36.D
Sample Name: HW-13-37

===

Acq. Operator : SYSTEM
Sample Operator : SYSTEM
Acq. Instrument : 1260II   Location : -
Injection Date : 1/9/2023 7:47:36 PM   Inj : 1
Inj Volume : No inj
Acq. Method : C:\Users\Public\Documents\ChemStation\1\Methods\def_LC.M
Last changed : 1/9/2023 7:07:57 PM by SYSTEM
(modified after loading)
Analysis Method : C:\Users\Public\Documents\ChemStation\1\Methods\def_LC.M
Last changed : 10/17/2023 10:51:55 AM by SYSTEM
(modified after loading)
Sample Info : IB, n-Hexane/i-PrOH = 90/10, 0.8 mL/min, 30 oC, 230 nm

Additional Info : Peak(s) manually integrated

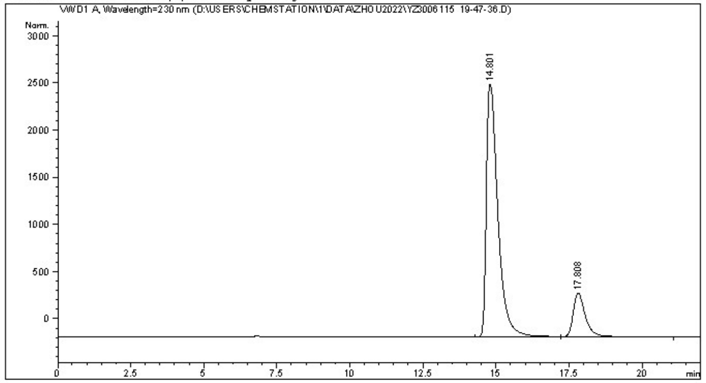

===
Area Percent Report
===

Sorted By : Signal
Multiplier : 1.0000
Dilution : 1.0000
Do not use Multiplier & Dilution Factor with ISTDs

Signal 1: VWD1 A, Wavelength=230 nm

Peak #	RetTime [min]	Type	Width [min]	Area [mAU*s]	Height [mAU]	Area %
1	14.801	BV	0.4165	6.33097e4	2297.26587	84.3860
2	17.808	VB	0.4372	1.17143e4	400.56366	15.6140

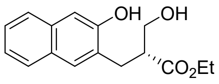

(+)-2p

1260II 10/17/2023 10:52:00 AM SYSTEM

Page 1 of 2

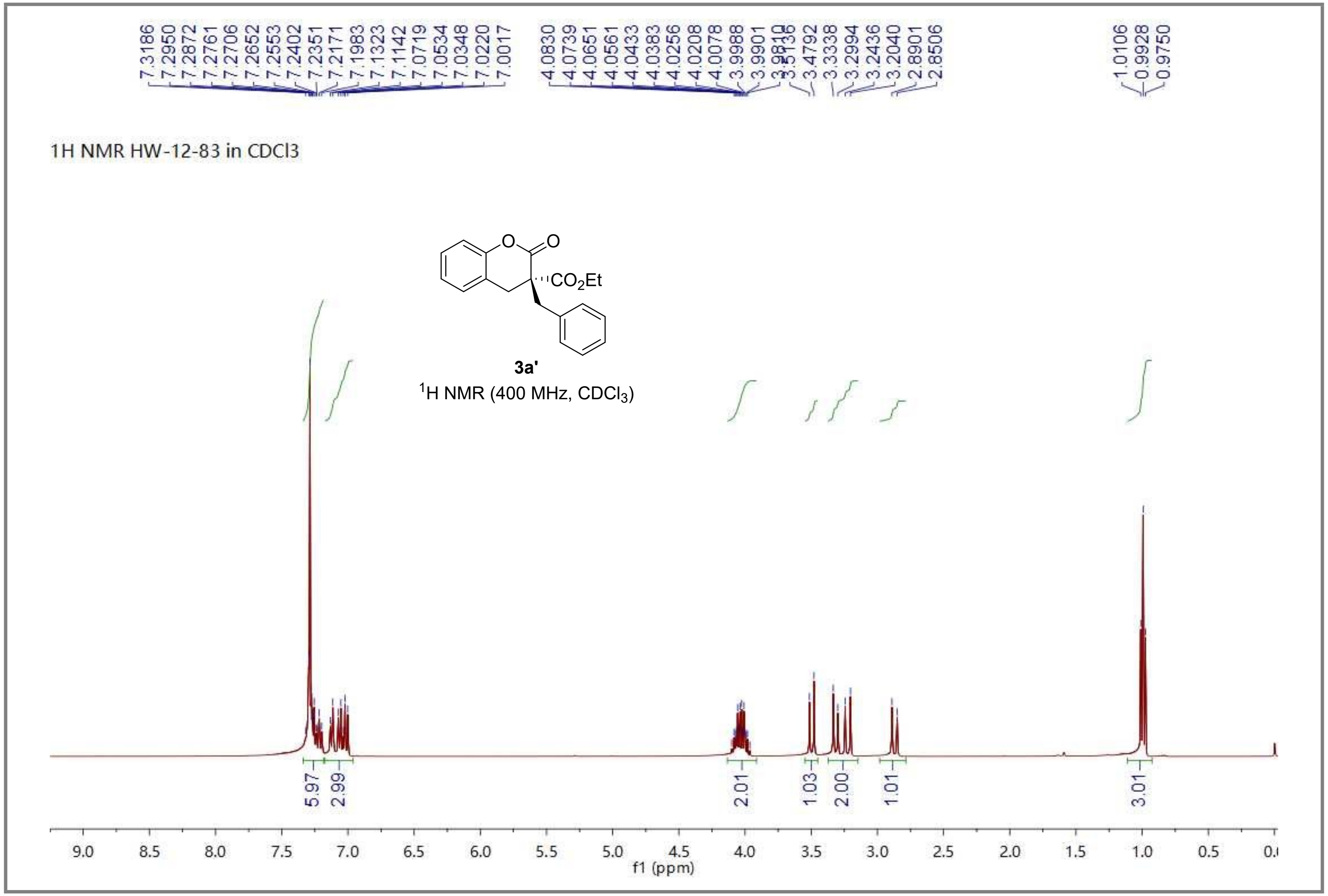

7.3186
7.2950
7.2872
7.2761
7.2706
7.2652
7.2553
7.2402
7.2351
7.2171
7.1983
7.1323
7.1142
7.0719
7.0534
7.0348
7.0220
7.0017
4.0830
4.0739
4.0651
4.0561
4.0433
4.0383
4.0256
4.0208
4.0078
3.9988
3.9901
3.9810
3.5136
3.4792
3.3338
3.2994
3.2436
3.2040
2.8901
2.8506
1.0106
0.9928
0.9750
1H NMR HW-12-83 in CDCl3
CO2Et
3a'
1H NMR (400 MHz, CDCl3)
5.97
2.99
2.01
1.03
2.00
1.01
3.01
9.0
8.5
8.0
7.5
7.0
6.5
6.0
5.5
5.0
4.5
4.0
3.5
3.0
2.5
2.0
1.5
1.0
0.5
f1 (ppm)

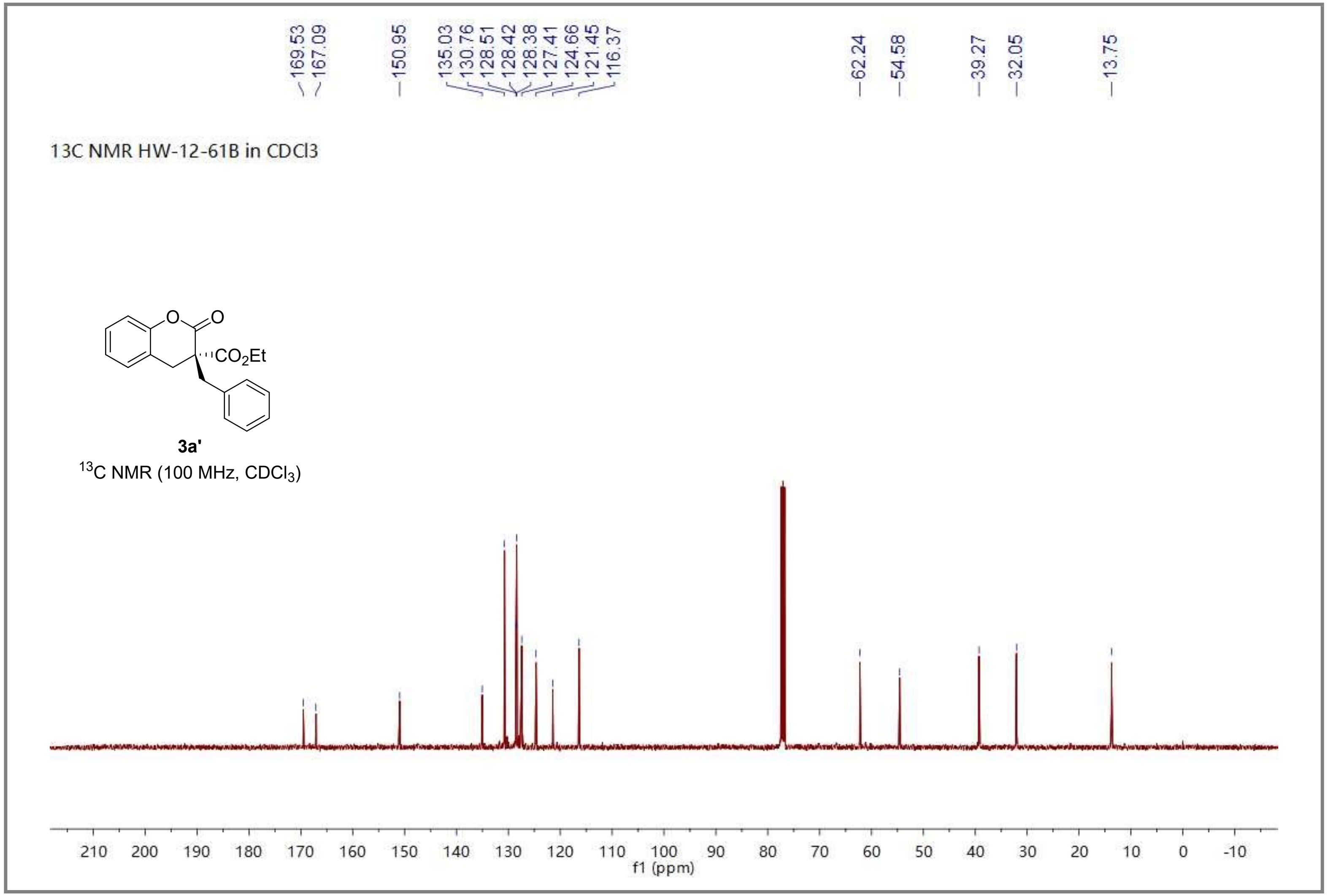
169.53
167.09
150.95
135.03
130.76
128.51
128.42
128.38
127.41
124.66
121.45
116.37
62.24
54.58
39.27
32.05
13.75
13C NMR HW-12-61B in CDCl3
O
O
CO2Et
3a'
13C NMR (100 MHz, CDCl3)
210 200 190 180 170 160 150 140 130 120 110 100 90 80 70 60 50 40 30 20 10 0 -10
f1 (ppm)

Data File C:\USERS\PUBLIC\DOCUMENTS\CHEMSTATION\1\DATA\2022\YZH002824 14-08-21.D
Sample Name: HW-12-64C SM+/-

===

Acq. Operator : SYSTEM
Sample Operator : SYSTEM
Acq. Instrument : HPLC1260 II   Location : 1
Injection Date : 11/15/2022 2:08:21 PM   Inj : 1
Inj Volume : No inj
Acq. Method : C:\USERS\PUBLIC\DOCUMENTS\CHEMSTATION\1\METHODS\def_LC1.M
Last changed : 11/15/2022 1:49:03 PM by SYSTEM
(modified after loading)
Analysis Method : C:\USERS\PUBLIC\DOCUMENTS\CHEMSTATION\1\METHODS\def_LC1.M
Last changed : 10/16/2023 8:53:54 PM by SYSTEM
(modified after loading)
Sample Info : AS-H, Hexane/i-PrOH = 90/10, 0.8 mL/min, 30 oC, 230 nm

Additional Info : Peak(s) manually integrated

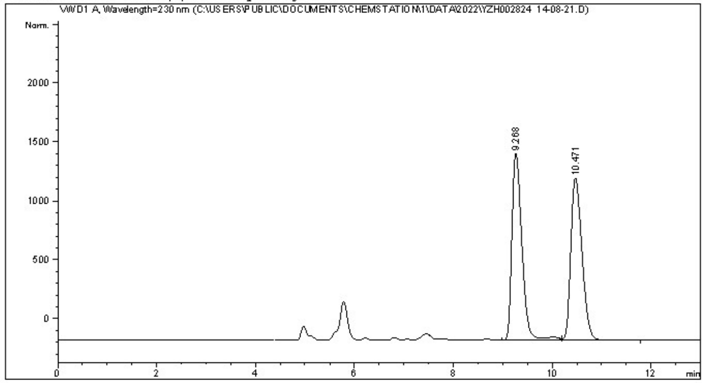

===
Area Percent Report
===

Sorted By : Signal
Multiplier : 1.0000
Dilution : 1.0000
Do not use Multiplier & Dilution Factor with ISTDs

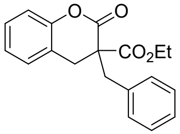

(+/-)-3a

Signal 1: VWD1 A, Wavelength=230 nm

Peak #	RetTime [min]	Type	Width [min]	Area [mAU*s]	Height [mAU]	Area %
1	9.268	BV R	0.2125	1.71476e4	1216.52478	50.7952
2	10.471	VB	0.2456	1.66107e4	1055.36694	49.2048

Data File C:\CHEM32\1\DATA\ZHOU-22\YZN022696.D
Sample Name: HW-14-8A SM'

===

Acq. Operator :
Acq. Instrument : Instrument 1   Location : -
Injection Date : 3/22/2023 8:46:31 AM
Acq. Method : C:\CHEM32\1\METHODS\DEF_LC.M
Last changed : 3/22/2023 8:34:55 AM
(modified after loading)
Analysis Method : C:\CHEM32\1\METHODS\DEF_LC.M
Last changed : 10/18/2023 4:00:41 AM
(modified after loading)
Sample Info : AS-H, Hexane/i-PrOH = 90/10, 0.8 mL/min, 30 oC, 230 nm

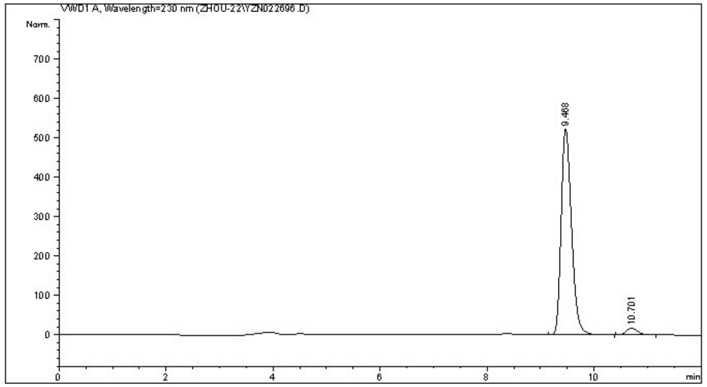

===
Area Percent Report
===

Sorted By : Signal
Multiplier: : 1.0000
Dilution: : 1.0000
Use Multiplier & Dilution Factor with ISTDs

Signal 1: VWD1 A, Wavelength=230 nm

Peak #	RetTime [min]	Type	Width [min]	Area mAU *s	Height [mAU]	Area %
1	9.468	VB	0.2098	7061.64648	523.32916	96.4830
2	10.701	BB	0.2273	257.40836	17.46237	3.5170
Totals :				7319.05484	540.79154	

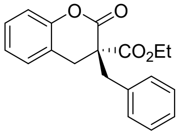

(+)-3a'

===
*** End of Report ***

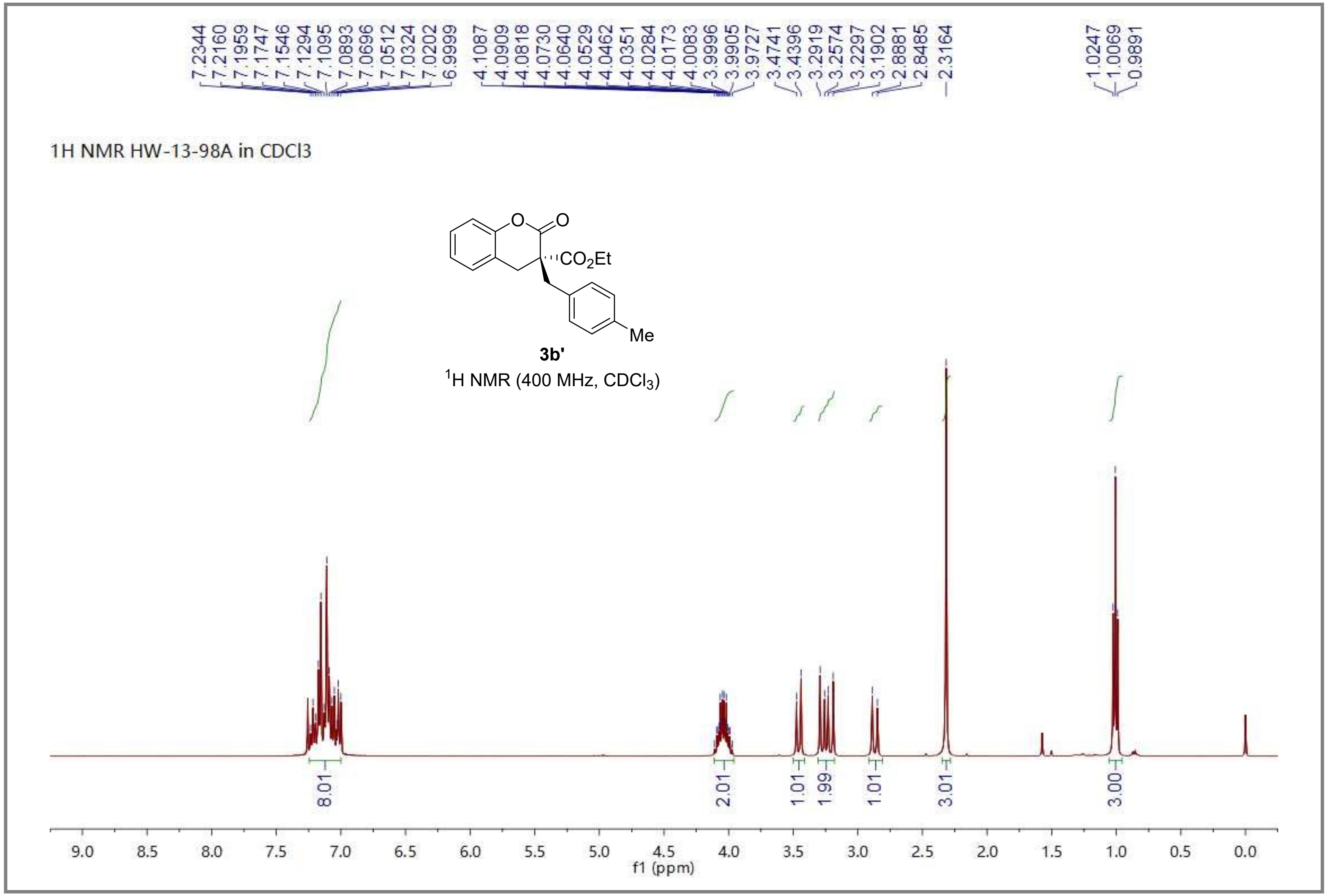

7.2344
7.2160
7.1959
7.1747
7.1546
7.1294
7.1095
7.0893
7.0696
7.0512
7.0324
7.0202
6.9999
4.1087
4.0909
4.0818
4.0730
4.0640
4.0529
4.0462
4.0351
4.0284
4.0173
4.0083
3.9996
3.9905
3.9727
3.4741
3.4396
3.2919
3.2574
3.2297
3.1902
2.8881
2.8485
2.3164
1.0247
1.0069
0.9891
1H NMR HW-13-98A in CDCl3
O
O
CO2Et
Me
3b'
1H NMR (400 MHz, CDCl3)
8.01
2.01
1.01
1.99
1.01
3.01
3.00
9.0
8.5
8.0
7.5
7.0
6.5
6.0
5.5
5.0
4.5
4.0
3.5
3.0
2.5
2.0
1.5
1.0
0.5
0.0
f1 (ppm)

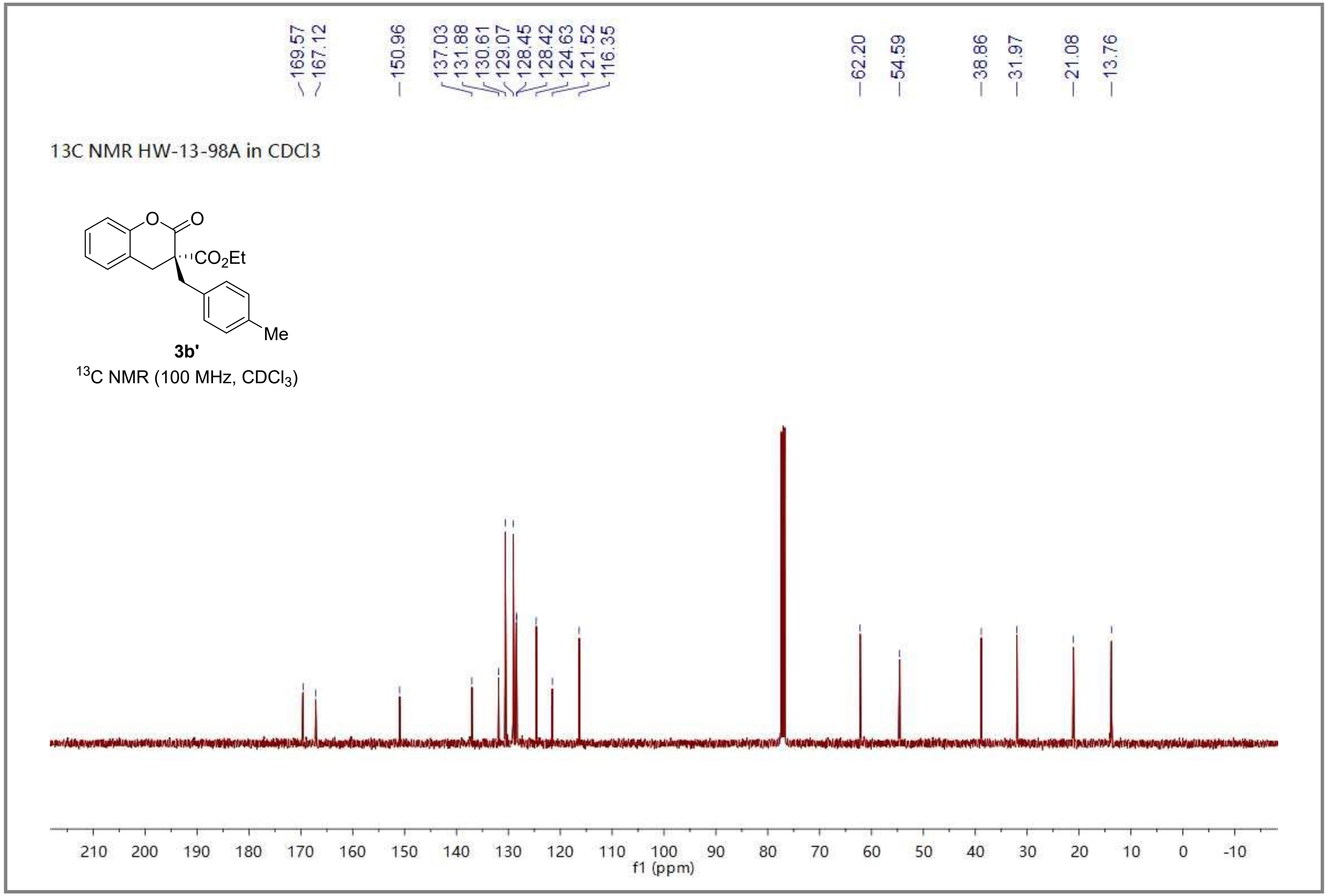
169.57
167.12
150.96
137.03
131.88
130.61
129.07
128.45
128.42
124.63
121.52
116.35
62.20
54.59
38.86
31.97
21.08
13.76
13C NMR HW-13-98A in CDCl3
O
O
CO2Et
Me
3b'
13C NMR (100 MHz, CDCl3)
210 200 190 180 170 160 150 140 130 120 110 100 90 80 70 60 50 40 30 20 10 0 -10
f1 (ppm)

Data File C:\CHEM32\1\DATA\ZHOU-22\YZN022666.D
Sample Name: HW-13-98A+/-

Acq. Operator :
Acq. Instrument : Instrument 1 Location : -
Injection Date : 3/18/2023 4:50:21 AM
Acq. Method : C:\CHEM32\1\METHODS\DEF_LC.M
Last changed : 3/18/2023 4:49:11 AM
(modified after loading)
Analysis Method : C:\CHEM32\1\METHODS\DEF_LC.M
Last changed : 10/17/2023 8:47:42 AM
(modified after loading)
Sample Info : AS-H, Hexane/i-PrOH = 90/10, 0.8 mL/min, 30 oC, 230nm

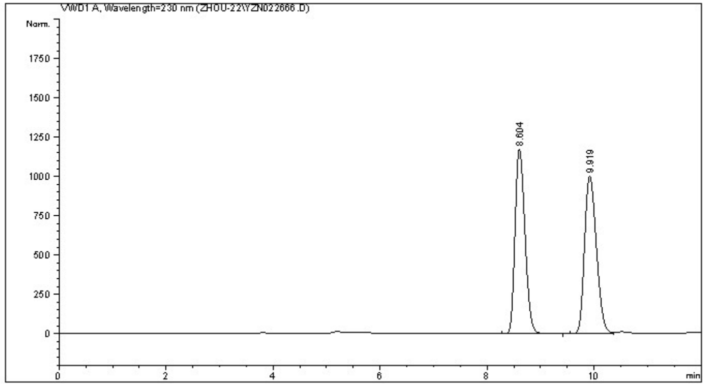

Area Percent Report

Sorted By : Signal
Multiplier: : 1.0000
Dilution: : 1.0000
Use Multiplier & Dilution Factor with ISTDs

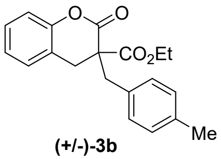

(+/-)-3b

Signal 1: VWD1 A, Wavelength=230 nm

Peak #	RetTime [min]	Type	Width [min]	Area mAU *s	Height [mAU]	Area %
1	8.604	BB	0.2026	1.51038e4	1173.28320	49.4957
2	9.919	BV	0.2419	1.54116e4	1002.69934	50.5043
Totals :				3.05154e4	2175.98254	

*** End of Report ***

Instrument 1 10/17/2023 8:47:46 AM Page 1 of 1

Data File C:\CHEM32\1\DATA\ZHOU-22\YZN022697.D
Sample Name: HW-14-8B SM'

Acq. Operator :
Acq. Instrument : Instrument 1 Location : -
Injection Date : 3/22/2023 9:09:22 AM
Acq. Method : C:\CHEM32\1\METHODS\DEF_LC.M
Last changed : 3/22/2023 9:06:42 AM
(modified after loading)
Analysis Method : C:\CHEM32\1\METHODS\DEF_LC.M
Last changed : 10/17/2023 8:49:46 AM
(modified after loading)
Sample Info : AS-H, Hexane/i-PrOH = 90/10, 0.8 mL/min, 30 oC, 230 nm

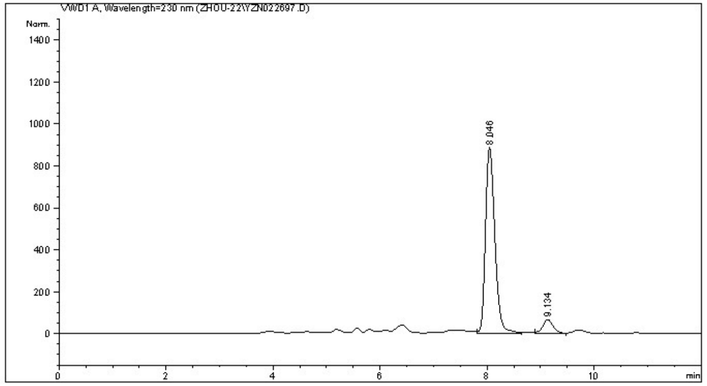

Area Percent Report

Sorted By : Signal
Multiplier: : 1.0000
Dilution: : 1.0000
Use Multiplier & Dilution Factor with ISTDs

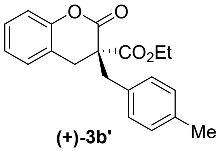

(+)-3b'

Signal 1: VWD1 A, Wavelength=230 nm

Peak #	RetTime [min]	Type	Width [min]	Area mAU *s	Height [mAU]	Area %
1	8.046	VV	0.1888	1.08113e4	886.86145	92.0598
2	9.134	VV	0.2106	932.47943	66.90748	7.9402
Totals :				1.17438e4	953.76893	

*** End of Report ***

Instrument 1 10/17/2023 8:49:50 AM Page 1 of 1

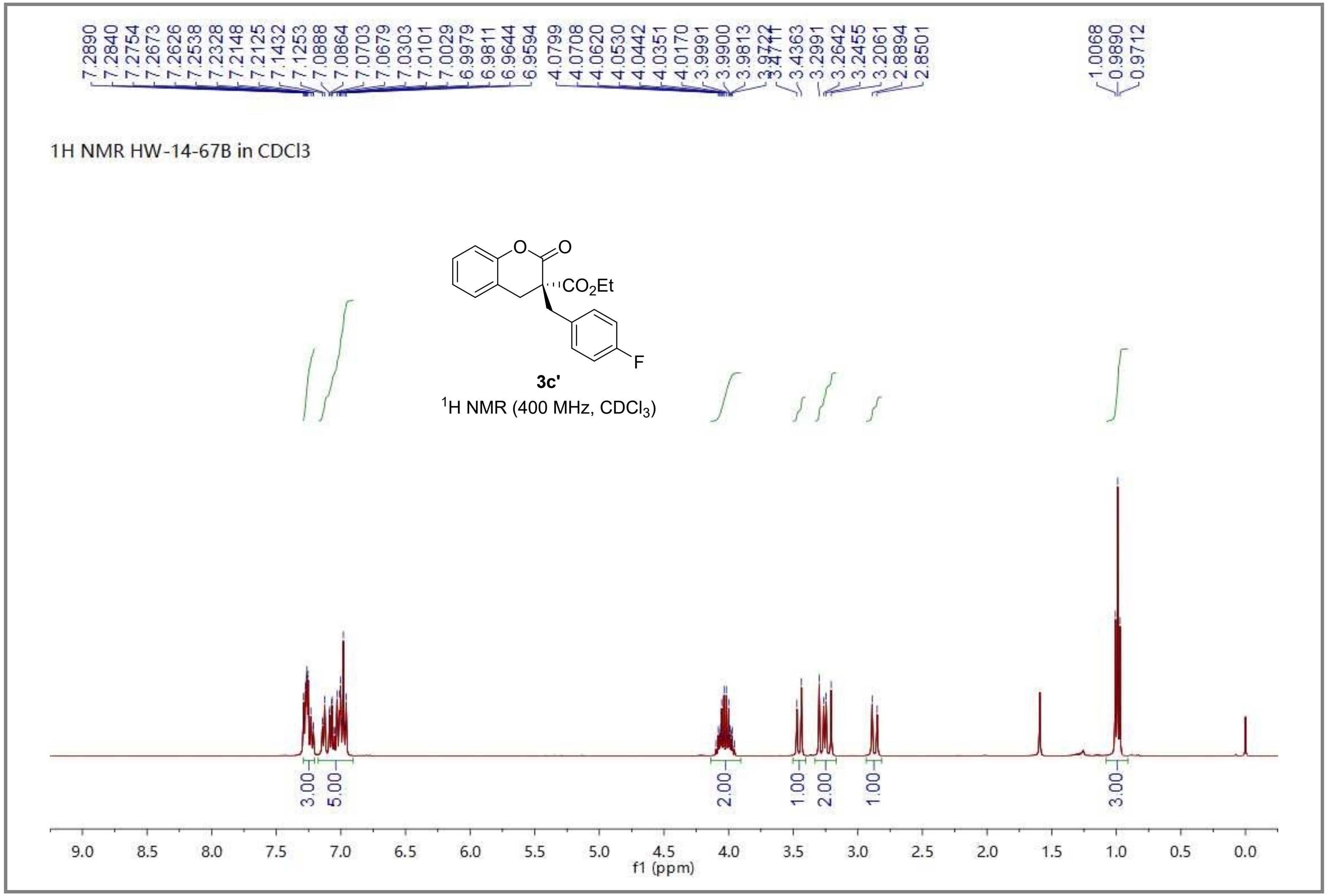

1H NMR HW-14-67B in CDCl3
3c'
1H NMR (400 MHz, CDCl3)
f1 (ppm)

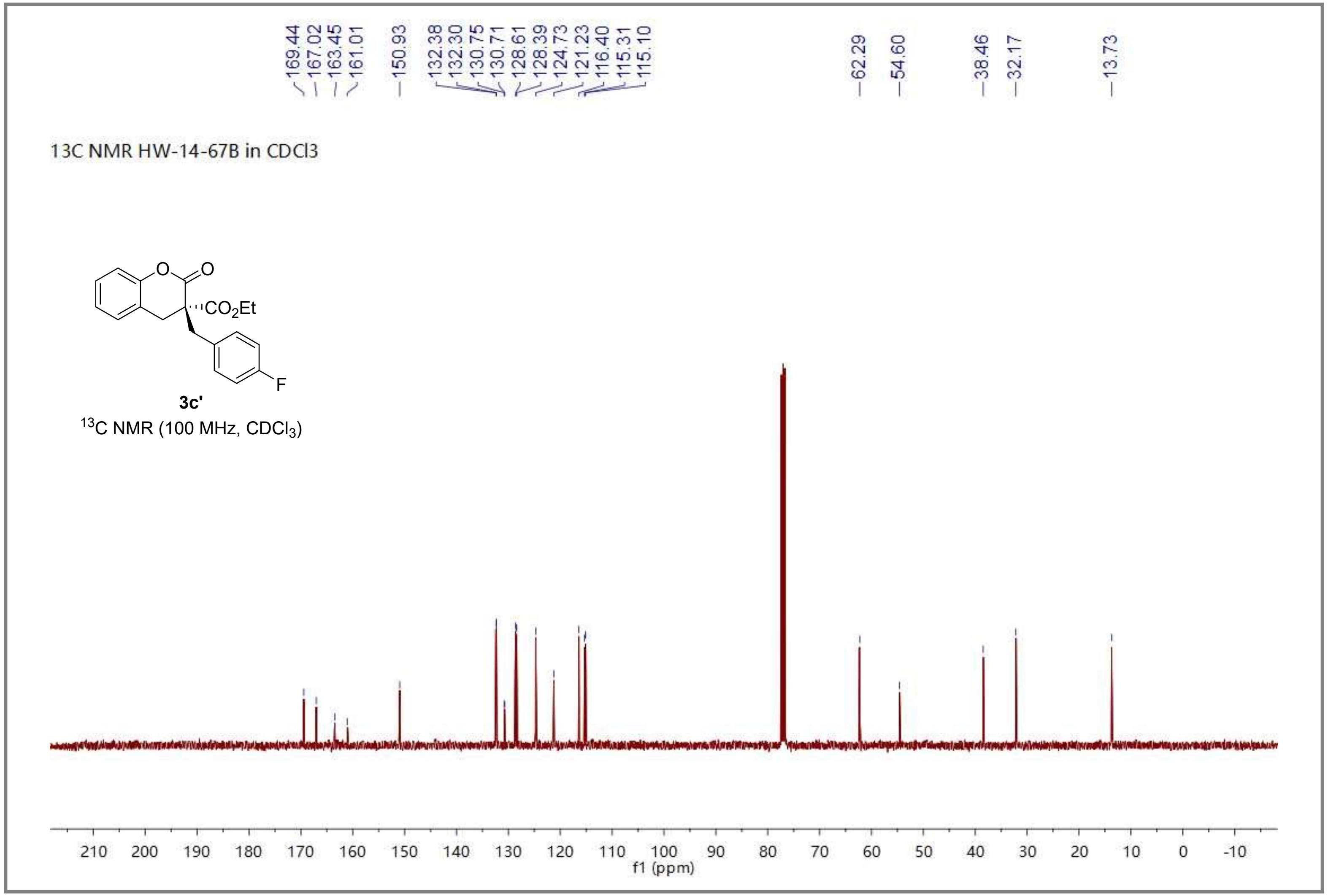
169.44
167.02
163.45
161.01
150.93
132.38
132.30
130.75
130.71
128.61
128.39
124.73
121.23
116.40
115.31
115.10
62.29
54.60
38.46
32.17
13.73
13C NMR HW-14-67B in CDCl3
O
O
CO2Et
F
3c'
13C NMR (100 MHz, CDCl3)
210 200 190 180 170 160 150 140 130 120 110 100 90 80 70 60 50 40 30 20 10 0 -10
f1 (ppm)

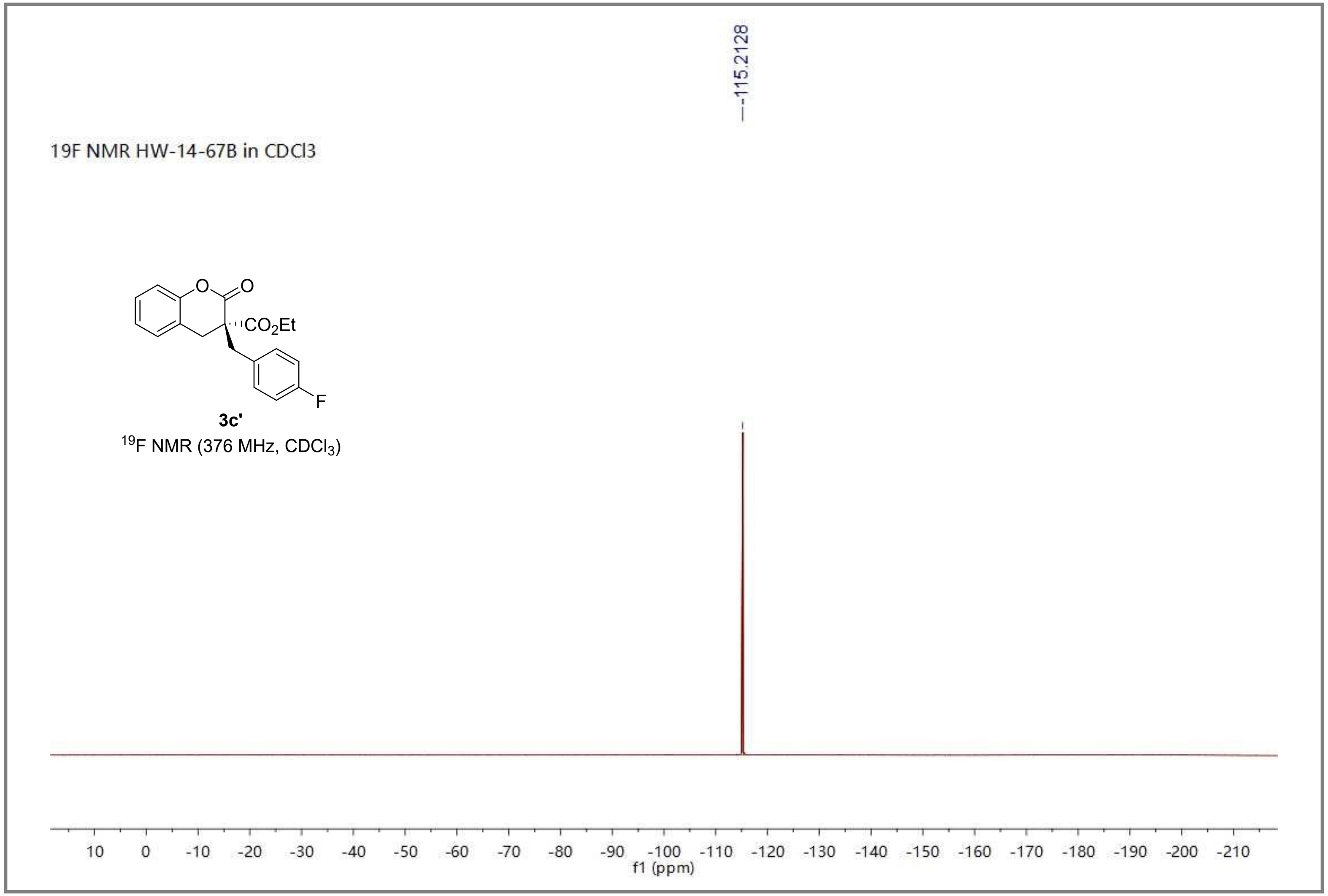
-115.2128
19F NMR HW-14-67B in CDCl3
CO2Et
F
3c'
19F NMR (376 MHz, CDCl3)
10 0 -10 -20 -30 -40 -50 -60 -70 -80 -90 -100 -110 -120 -130 -140 -150 -160 -170 -180 -190 -200 -210
f1 (ppm)

```
Data File C:\CHEM32\1\DATA\ZHOU-22\YZH005562  17-30-03.D
Sample Name: HW-14-67B+/-

    =====================================================================
    Acq. Operator    : SYSTEM
    Acq. Instrument  : HPLC1260 II                   Location : Vial 1
    Injection Date   : 11/28/2023 5:30:03 PM
                                                  Inj Volume : No inj
    Acq. Method      : C:\USERS\PUBLIC\DOCUMENTS\CHEMSTATION\1\METHODS\def_LC1.M
    Last changed     : 11/28/2023 5:27:19 PM by SYSTEM
                       (modified after loading)
    Analysis Method  : C:\CHEM32\1\METHODS\DEF_LC.M
    Last changed     : 11/30/2023 8:58:34 AM
                       (modified after loading)
```

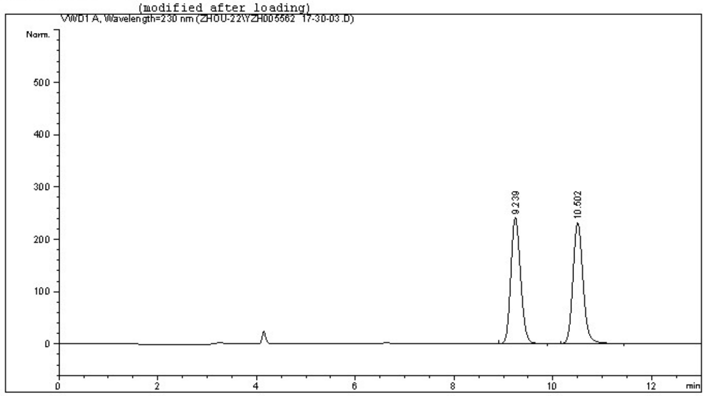


```
    =====================================================================
                             Area Percent Report
    =====================================================================

    Sorted By             :      Signal
    Multiplier:                   :      1.0000
    Dilution:                     :      1.0000
    Use Multiplier & Dilution Factor with ISTDs


    Signal 1: VWD1 A, Wavelength=230 nm

    Peak RetTime Type  Width     Area      Height     Area
      #   [min]        [min]   [mAU*s]     [mAU]        %
    ----|-------|----|-------|----------|----------|--------|
       1   9.239 BB    0.2119 3275.86230  240.48276  50.0812
       2  10.502 BB    0.2191 3265.23950  230.76292  49.9188

    Totals :                  6541.10181  471.24568

    =====================================================================
                             *** End of Report ***
```

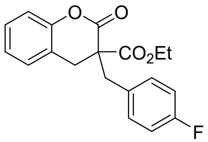

(+/-)-3c

Instrument 1 11/30/2023 8:58:44 AM   Page 1 of 1

```
Data File C:\CHEM32\1\DATA\ZHOU-23\YZN023214.D
Sample Name: HW-14-73B SM

    =====================================================================
    Acq. Operator    :
    Acq. Instrument  : Instrument 1                  Location :   -
    Injection Date   : 5/30/2023 4:58:20 AM
    Acq. Method      : C:\CHEM32\1\METHODS\DEF_LC.M
    Last changed     : 5/30/2023 4:57:54 AM
                       (modified after loading)
    Analysis Method  : C:\CHEM32\1\METHODS\DEF_LC.M
    Last changed     : 10/17/2023 8:58:26 AM
                       (modified after loading)
    Sample Info      : AS-H, Hexane/i-PrOH = 90/10, 0.8 mL/min, 30 oC, 230 nm
```

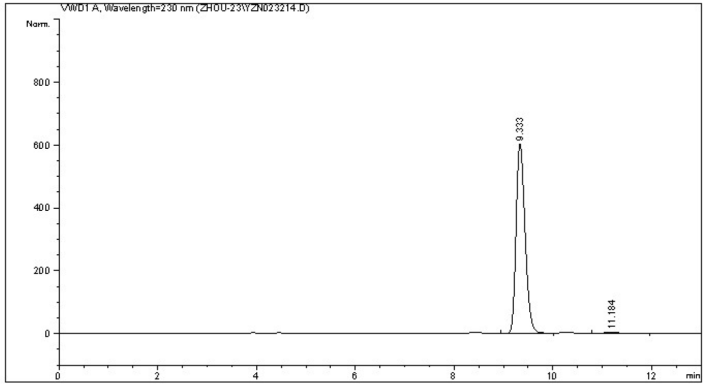


```
    =====================================================================
                             Area Percent Report
    =====================================================================

    Sorted By             :      Signal
    Multiplier:                   :      1.0000
    Dilution:                     :      1.0000
    Use Multiplier & Dilution Factor with ISTDs


    Signal 1: VWD1 A, Wavelength=230 nm

    Peak RetTime Type  Width     Area      Height     Area
      #   [min]        [min]  mAU   *s   [mAU   ]      %
    ----|-------|----|-------|----------|----------|--------|
       1   9.333 BV    0.1972 7633.78906  603.07947  99.1347
       2  11.184 VB    0.3151   66.63427    3.07884   0.8653

    Totals :                  7700.42333  606.15831

    =====================================================================
                             *** End of Report ***
```

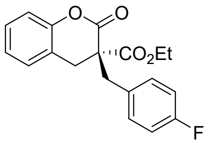

(+)-3c'

Instrument 1 10/17/2023 8:58:36 AM   Page 1 of 1

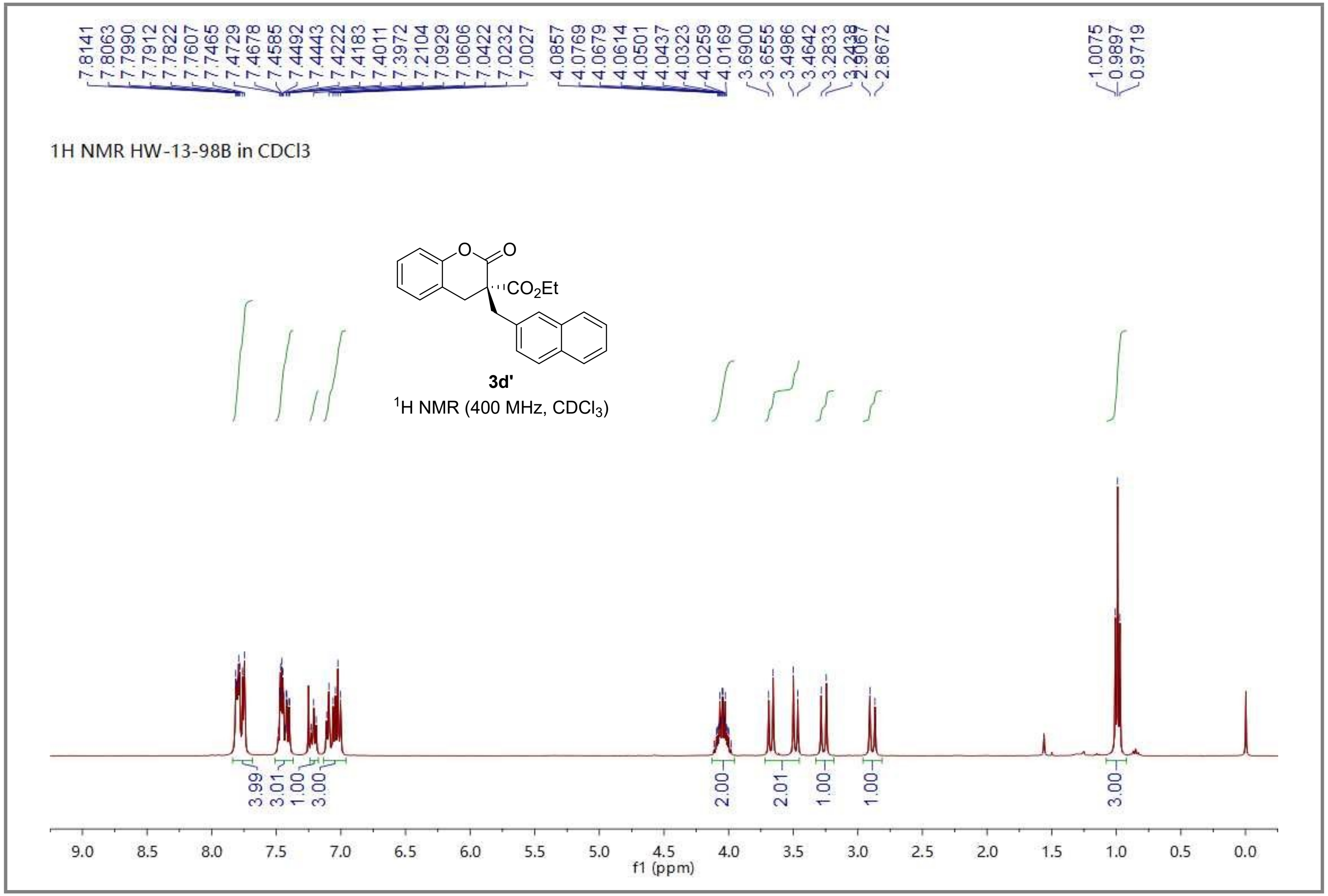

7.8141
7.8063
7.7990
7.7912
7.7822
7.7607
7.7465
7.4729
7.4678
7.4585
7.4492
7.4443
7.4222
7.4183
7.4011
7.3972
7.2104
7.0929
7.0606
7.0422
7.0232
7.0027
4.0857
4.0769
4.0679
4.0614
4.0501
4.0437
4.0323
4.0259
4.0169
3.6900
3.6555
3.4986
3.4642
3.2833
3.2439
2.9057
2.8672
1.0075
0.9897
0.9719
1H NMR HW-13-98B in CDCl3
CO2Et
3d'
1H NMR (400 MHz, CDCl3)
3.99
3.01
1.00
3.00
2.00
2.01
1.00
1.00
3.00
9.0 8.5 8.0 7.5 7.0 6.5 6.0 5.5 5.0 4.5 4.0 3.5 3.0 2.5 2.0 1.5 1.0 0.5 0.0
f1 (ppm)

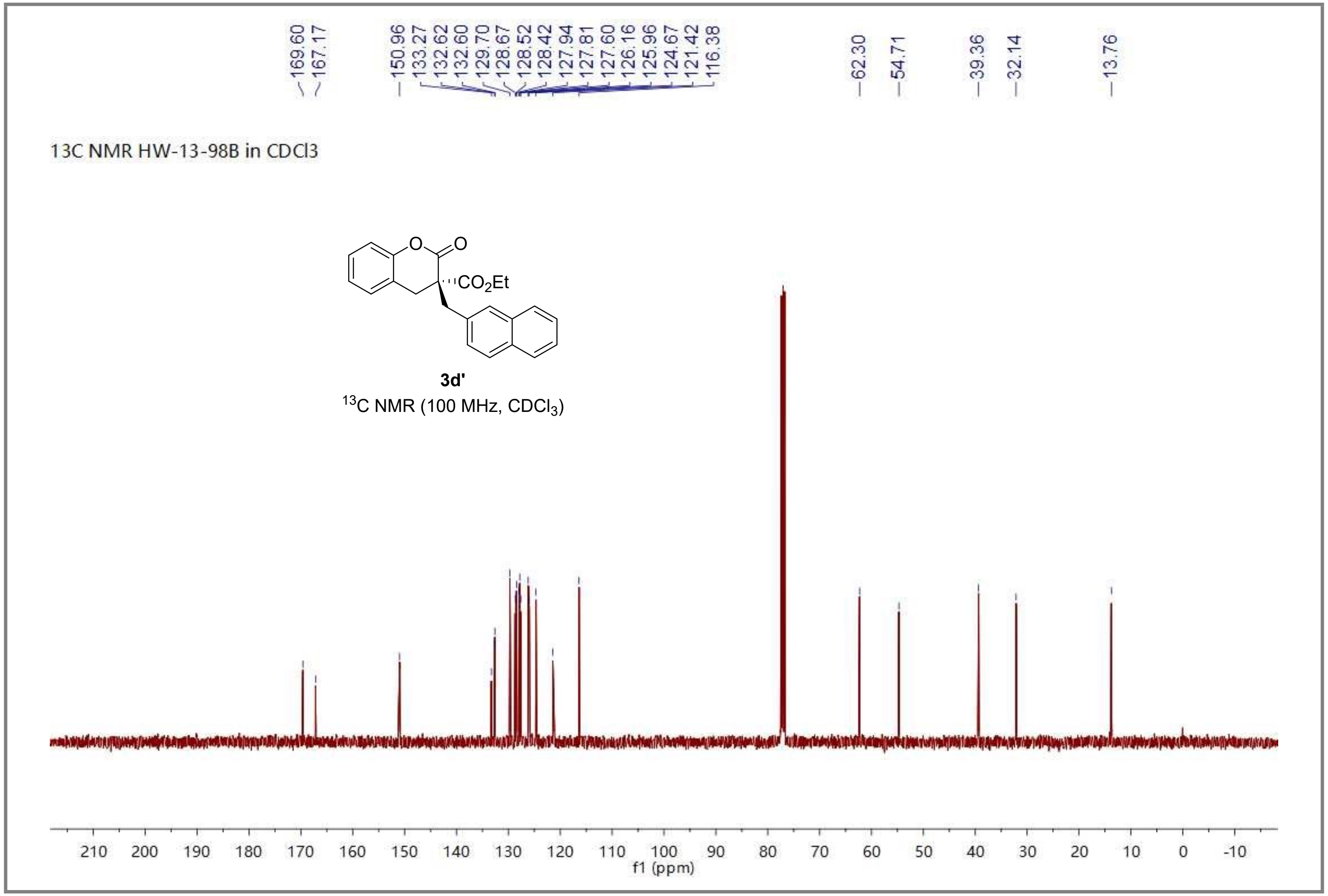
169.60
167.17
150.96
133.27
132.62
132.60
129.70
128.67
128.52
128.42
127.94
127.81
127.60
126.16
125.96
124.67
121.42
116.38
62.30
54.71
39.36
32.14
13.76
13C NMR HW-13-98B in CDCl3
CO2Et
O
O
3d'
13C NMR (100 MHz, CDCl3)
f1 (ppm)
210
200
190
180
170
160
150
140
130
120
110
100
90
80
70
60
50
40
30
20
10
0
-10

```
Data File C:\CHEM32\1\DATA\ZHOU-23\YZH005556  15-34-07.D
Sample Name: HW-13-98B +/-

   =====================================================================
   Acq. Operator   : SYSTEM
   Acq. Instrument : HPLC1260 II                 Location : Vial 1
   Injection Date  : 11/28/2023 3:34:08 PM
                                               Inj Volume : No inj
   Acq. Method     : C:\USERS\PUBLIC\DOCUMENTS\CHEMSTATION\1\METHODS\def_LC1.M
   Last changed    : 11/28/2023 3:17:25 PM by SYSTEM
                     (modified after loading)
   Analysis Method : C:\CHEM32\1\METHODS\DEF_LC.M
   Last changed    : 11/30/2023 8:07:56 AM
                     (modified after loading)
```

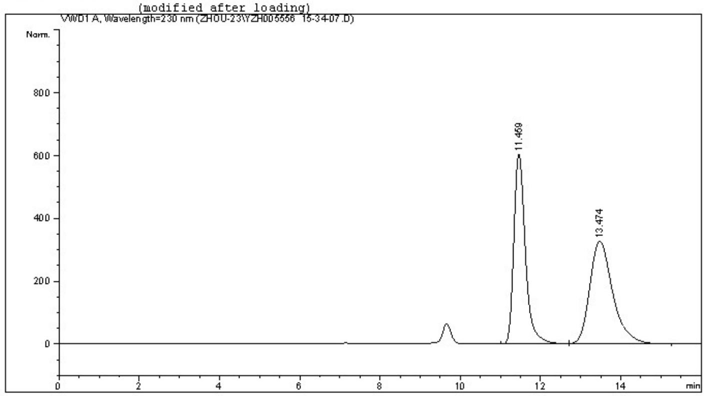


```
   =====================================================================
                        Area Percent Report
   =====================================================================

   Sorted By             :      Signal
   Multiplier:                  :      1.0000
   Dilution:                    :      1.0000
   Use Multiplier & Dilution Factor with ISTDs


   Signal 1: VWD1 A, Wavelength=230 nm
```

Peak #	RetTime [min]	Type	Width [min]	Area [mAU*s]	Height [mAU]	Area %
1	11.459	BV	0.3069	1.20354e4	602.91223	48.0585
2	13.474	VB	0.6067	1.30079e4	327.30435	51.9415
Totals :				2.50433e4	930.21658	

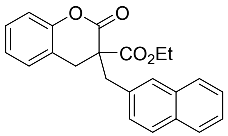

(+/-)-3d

```
   =====================================================================
                        *** End of Report ***
```

Instrument 1 11/30/2023 8:08:17 AM Page 1 of 1

```
Data File C:\USERS\PUBLIC\DOCUMENTS\CHEMSTATION\1\DATA\2023\YZH004326  14-10-08.D
Sample Name: HW-14-76SM

   =====================================================================
   Acq. Operator   : SYSTEM
   Sample Operator : SYSTEM
   Acq. Instrument : HPLC1260 II                Location :   1
   Injection Date  : 6/5/2023 2:10:08 PM             Inj :   1
                                              Inj Volume : No inj
   Acq. Method     : C:\USERS\PUBLIC\DOCUMENTS\CHEMSTATION\1\METHODS\def_LC1.M
   Last changed    : 6/5/2023 1:48:31 PM by SYSTEM
                     (modified after loading)
   Analysis Method : C:\USERS\PUBLIC\DOCUMENTS\CHEMSTATION\1\METHODS\def_LC1.M
   Last changed    : 11/29/2023 9:29:35 PM by SYSTEM
                     (modified after loading)
   Sample Info     : AS-H, Hexane/i-PrOH = 90/10, 0.8 mL/min, 30 oC, 230 nm

   Additional Info : Peak(s) manually integrated
```

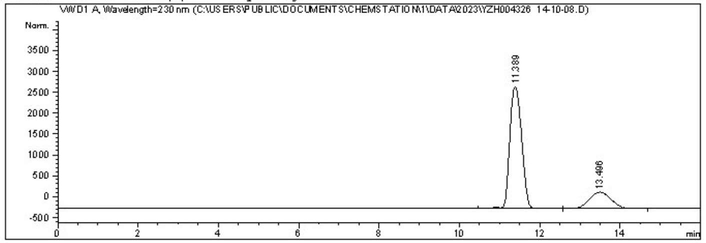


```
   =====================================================================
                        Area Percent Report
   =====================================================================

   Sorted By             :      Signal
   Multiplier            :      1.0000
   Dilution              :      1.0000
   Sample Amount:        :      1.00000  [ng/ul]   (not used in calc.)
   Do not use Multiplier & Dilution Factor with ISTDs


   Signal 1: VWD1 A, Wavelength=230 nm
```

Peak #	RetTime [min]	Type	Width [min]	Area [mAU*s]	Height [mAU]	Area %
1	11.389	VV R	0.3307	4.77353e4	2337.02051	79.8645
2	13.496	BB	0.5983	1.20351e4	316.75845	20.1355
Totals :				5.97704e4	2653.77896	

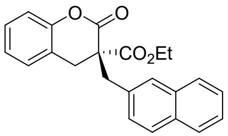

(+)-3d'

```
   =====================================================================
                        *** End of Report ***
```

HPLC1260 II 11/29/2023 9:29:43 PM SYSTEM Page 1 of 1

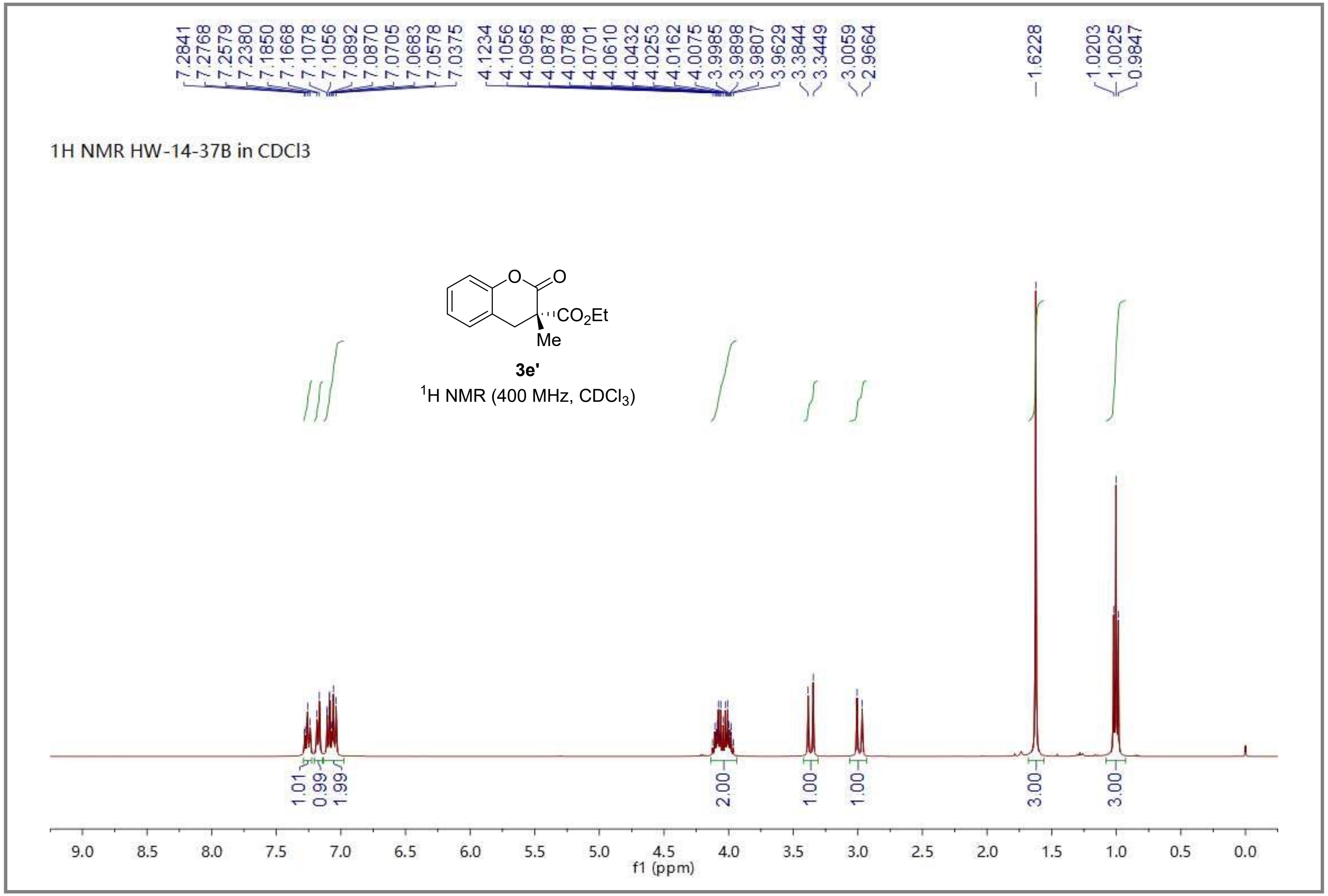
1H NMR HW-14-37B in CDCl3
3e'
1H NMR (400 MHz, CDCl3)
CO2Et
Me
7.2841
7.2768
7.2579
7.2380
7.1850
7.1668
7.1078
7.1056
7.0892
7.0870
7.0705
7.0683
7.0578
7.0375
4.1234
4.1056
4.0965
4.0878
4.0788
4.0701
4.0610
4.0432
4.0253
4.0162
4.0075
3.9985
3.9898
3.9807
3.9629
3.3844
3.3449
3.0059
2.9664
1.6228
1.0203
1.0025
0.9847
1.01
0.99
1.99
2.00
1.00
1.00
3.00
3.00
f1 (ppm)

13C NMR HW-14-37B in CDCl3

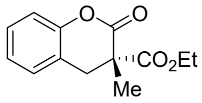

3e'

^{13}C NMR (100 MHz, $CDCl_3$)

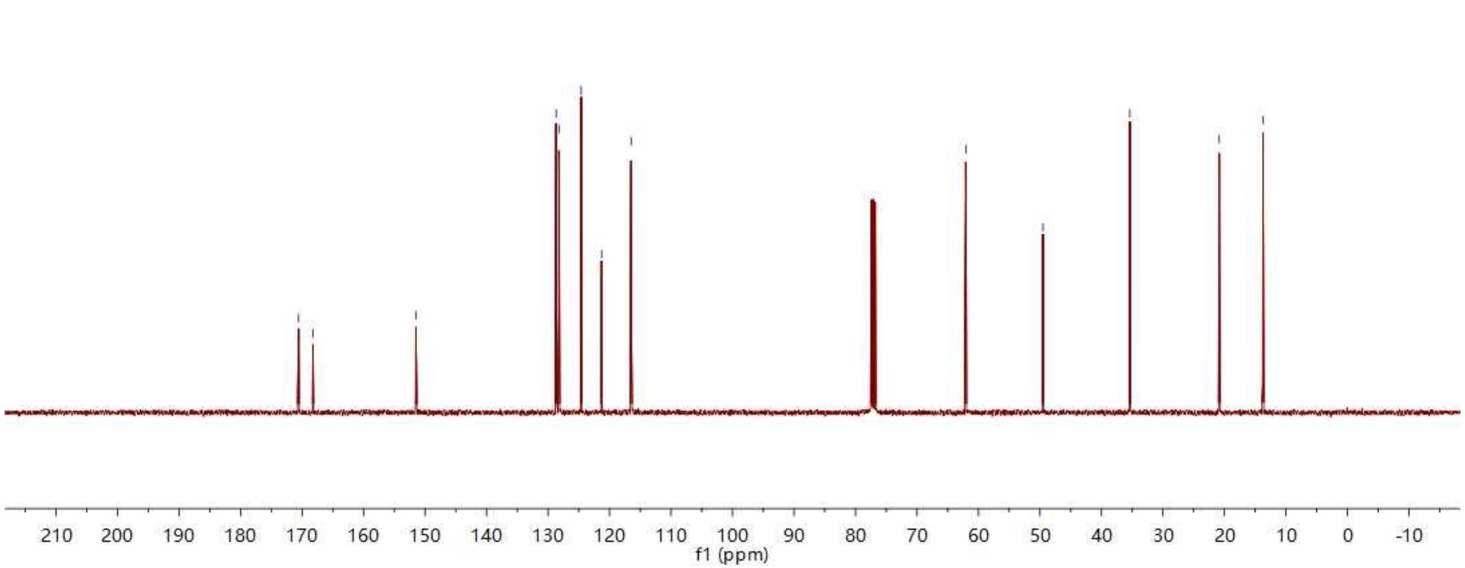

```
Data File C:\CHEM32\1\DATA\ZHOU-23\YZN022942.D
Sample Name: HW-14-41B SM+/-

    =====================================================================
    Acq. Operator    :
    Acq. Instrument  : Instrument 1                    Location :   -
    Injection Date   : 4/22/2023 9:08:35 AM
    Acq. Method      : C:\CHEM32\1\METHODS\DEF_LC.M
    Last changed     : 4/22/2023 9:08:11 AM
                       (modified after loading)
    Analysis Method  : C:\CHEM32\1\METHODS\DEF_LC.M
    Last changed     : 10/17/2023 9:15:12 AM
                       (modified after loading)
    Sample Info      : AS-H, Hexane/i-PrOH = 90/10, 0.8mL/min, 30 oC, 230 nm
```

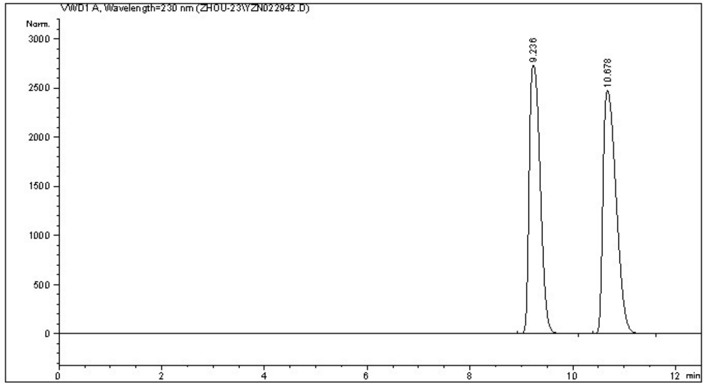


```
    =====================================================================
                             Area Percent Report
    =====================================================================

    Sorted By             :      Signal
    Multiplier:                   :      1.0000
    Dilution:                     :      1.0000
    Use Multiplier & Dilution Factor with ISTDs


    Signal 1: VWD1 A, Wavelength=230 nm

    Peak RetTime Type  Width     Area      Height     Area
      #   [min]        [min]  mAU   *s   [mAU   ]      %
    ----|-------|----|-------|----------|----------|--------|
       1   9.236 BB    0.2377 4.03326e4  2733.45776  48.1167
       2  10.678 BB    0.2808 4.34900e4  2474.20898  51.8833

    Totals :                  8.38226e4  5207.66675

    =====================================================================
                              *** End of Report ***
```

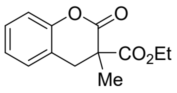

(+/-)-3e

Instrument 1 10/17/2023 9:15:16 AM   Page 1 of 1

```
Data File C:\CHEM32\1\DATA\ZHOU-23\YZN022973.D
Sample Name: HW-14-41B SM'

    =====================================================================
    Acq. Operator    :
    Acq. Instrument  : Instrument 1                    Location :   -
    Injection Date   : 4/26/2023 3:50:09 AM
    Acq. Method      : C:\CHEM32\1\METHODS\DEF_LC.M
    Last changed     : 4/26/2023 3:46:00 AM
                       (modified after loading)
    Analysis Method  : C:\CHEM32\1\METHODS\DEF_LC.M
    Last changed     : 10/17/2023 9:16:21 AM
                       (modified after loading)
    Sample Info      : AS-H, Hexane/i-PrOH = 90/10, 0.8 mL/min, 30 oC, 230 nm
```

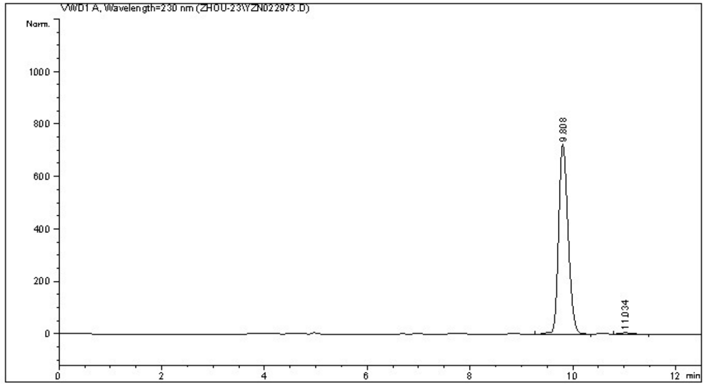


```
    =====================================================================
                             Area Percent Report
    =====================================================================

    Sorted By             :      Signal
    Multiplier:                   :      1.0000
    Dilution:                     :      1.0000
    Use Multiplier & Dilution Factor with ISTDs


    Signal 1: VWD1 A, Wavelength=230 nm

    Peak RetTime Type  Width     Area      Height     Area
      #   [min]        [min]  mAU   *s   [mAU   ]      %
    ----|-------|----|-------|----------|----------|--------|
       1   9.808 BV    0.1985 9239.00488  723.36456  99.1812
       2  11.034 VB    0.2306   76.27379    5.07842   0.8188

    Totals :                  9315.27867  728.44298

    =====================================================================
                              *** End of Report ***
```

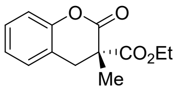

(+)-3e'

Instrument 1 10/17/2023 9:16:29 AM   Page 1 of 1

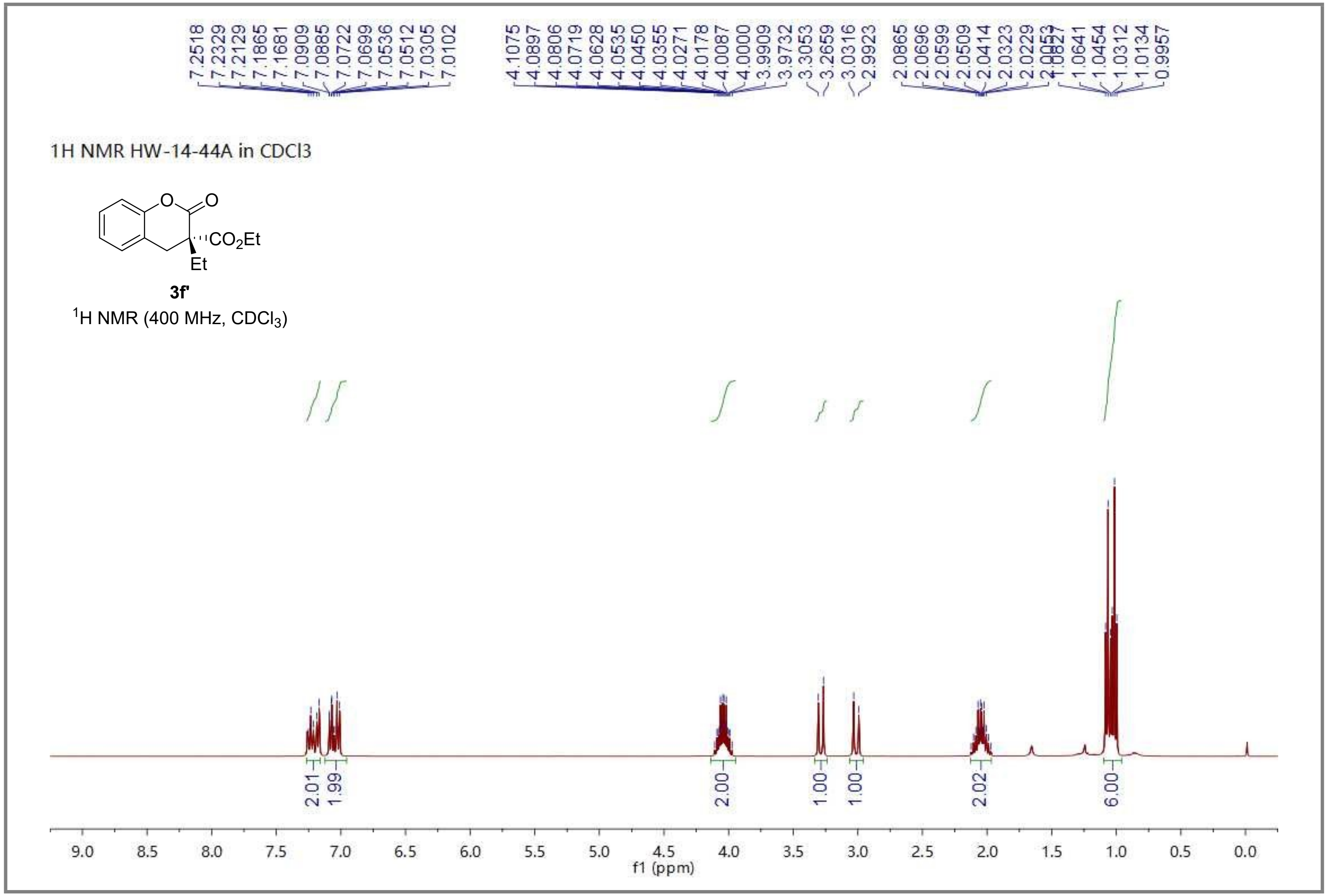
7.2518
7.2329
7.2129
7.1865
7.1681
7.0909
7.0885
7.0722
7.0699
7.0536
7.0512
7.0305
7.0102
4.1075
4.0897
4.0806
4.0719
4.0628
4.0535
4.0450
4.0355
4.0271
4.0178
4.0087
4.0000
3.9909
3.9732
3.3053
3.2659
3.0316
2.9923
2.0865
2.0696
2.0599
2.0509
2.0414
2.0323
2.0229
1.0641
1.0454
1.0312
1.0134
0.9957
1H NMR HW-14-44A in CDCl3
O
O
CO2Et
Et
3f'
1H NMR (400 MHz, CDCl3)
2.01
1.99
2.00
1.00
1.00
2.02
6.00
9.0
8.5
8.0
7.5
7.0
6.5
6.0
5.5
5.0
4.5
4.0
3.5
3.0
2.5
2.0
1.5
1.0
0.5
0.0
f1 (ppm)

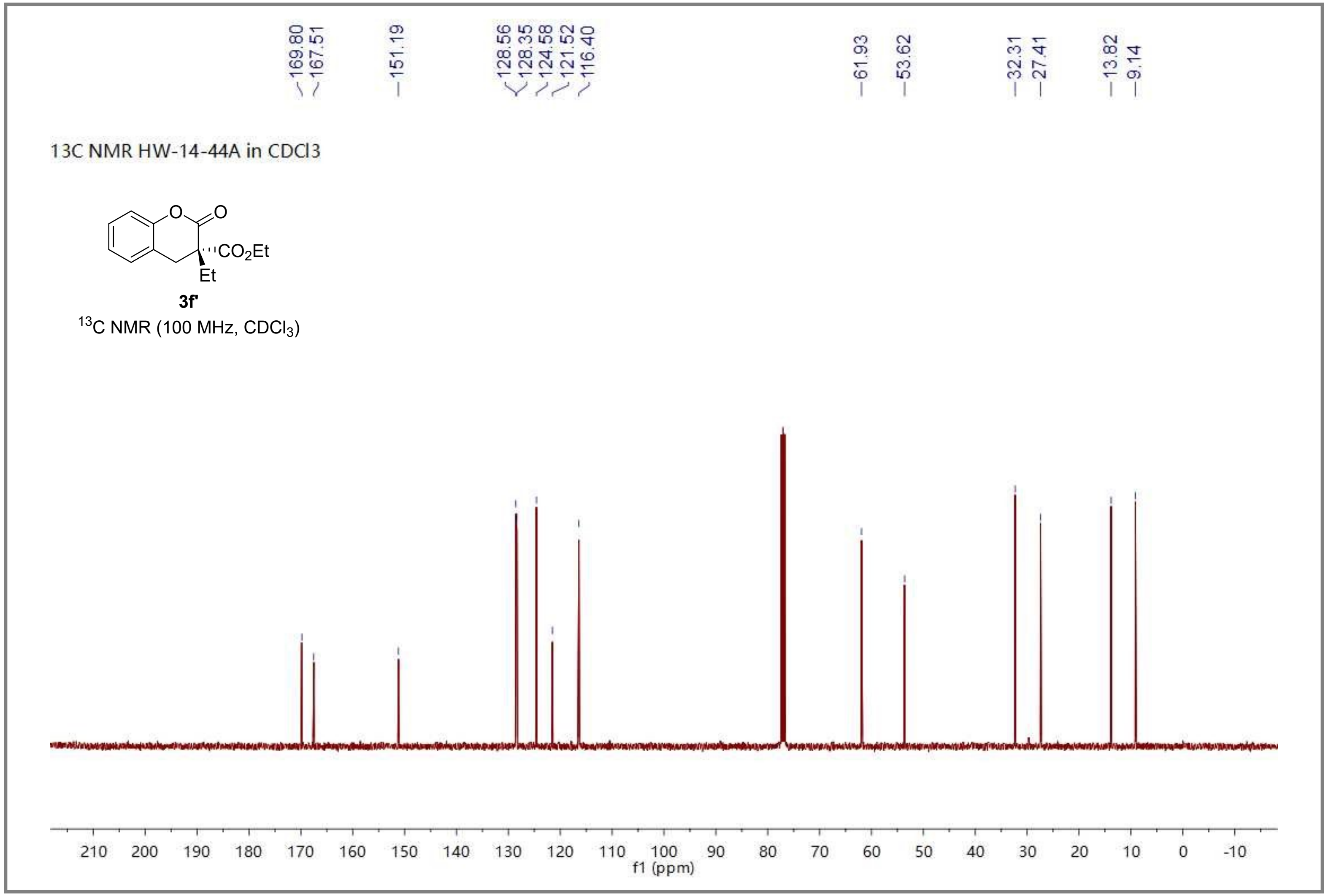
169.80
167.51
151.19
128.56
128.35
124.58
121.52
116.40
61.93
53.62
32.31
27.41
13.82
9.14
13C NMR HW-14-44A in CDCl3
O
O
CO2Et
Et
3f'
13C NMR (100 MHz, CDCl3)
210 200 190 180 170 160 150 140 130 120 110 100 90 80 70 60 50 40 30 20 10 0 -10
f1 (ppm)

Data File C:\CHEM32\1\DATA\ZHOU-23\YZN022940.D
Sample Name: HW-14-34A SM+/-

===

Acq. Operator :
Acq. Instrument : Instrument 1 Location : -
Injection Date : 4/22/2023 8:40:11 AM
Acq. Method : C:\CHEM32\1\METHODS\DEF_LC.M
Last changed : 4/22/2023 8:35:28 AM
(modified after loading)
Analysis Method : C:\CHEM32\1\METHODS\DEF_LC.M
Last changed : 10/17/2023 9:24:39 AM
(modified after loading)
Sample Info : AS-H, Hexane/i-PrOH = 90/10, 0.8mL/min, 30 oC, 230 nm

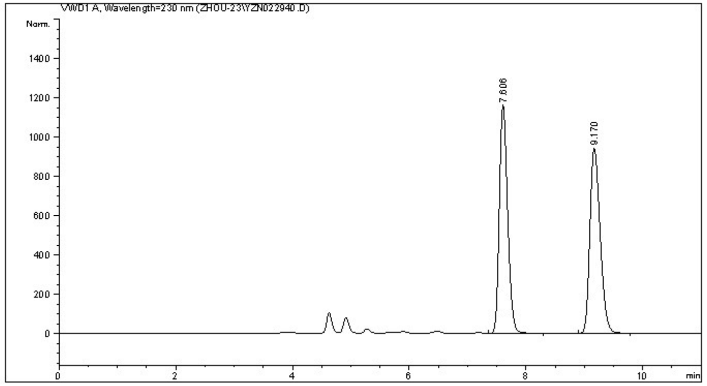

===
Area Percent Report
===

Sorted By : Signal
Multiplier: : 1.0000
Dilution: : 1.0000
Use Multiplier & Dilution Factor with ISTDs

Signal 1: VWD1 A, Wavelength=230 nm

Peak #	RetTime [min]	Type	Width [min]	Area mAU *s	Height [mAU]	Area %
1	7.606	VB	0.1526	1.14103e4	1160.53186	49.8726
2	9.170	VV	0.1903	1.14687e4	940.75964	50.1274

Totals : 2.28790e4 2101.29150

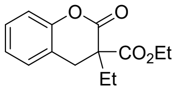

(+/-)-3f

===
*** End of Report ***

Instrument 1 10/17/2023 9:24:42 AM Page 1 of 1

Data File C:\CHEM32\1\DATA\ZHOU-23\YZN022947.D
Sample Name: HW-14-32A SM

===

Acq. Operator :
Acq. Instrument : Instrument 1 Location : -
Injection Date : 4/23/2023 1:27:16 AM
Acq. Method : C:\CHEM32\1\METHODS\DEF_LC.M
Last changed : 4/23/2023 1:16:53 AM
(modified after loading)
Analysis Method : C:\CHEM32\1\METHODS\DEF_LC.M
Last changed : 1/12/2024 3:53:05 AM
(modified after loading)
Sample Info : AS-H, Hexane/i-PrOH = 90/10, 0.8 mL/min, 30 oC, 230 nm

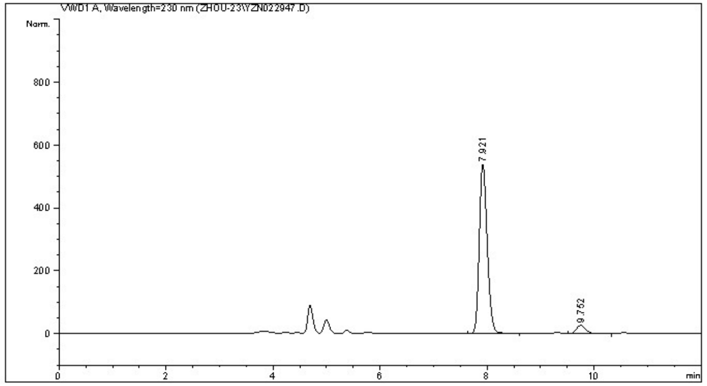

===
Area Percent Report
===

Sorted By : Signal
Multiplier: : 1.0000
Dilution: : 1.0000
Use Multiplier & Dilution Factor with ISTDs

Signal 1: VWD1 A, Wavelength=230 nm

Peak #	RetTime [min]	Type	Width [min]	Area mAU *s	Height [mAU]	Area %
1	7.921	VV	0.1605	5609.98291	540.15234	94.2767
2	9.752	VV	0.1929	340.56915	27.16190	5.7233

Totals : 5950.55206 567.31424

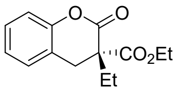

(+)-3f'

===
*** End of Report ***

Instrument 1 1/12/2024 3:53:20 AM Page 1 of 1

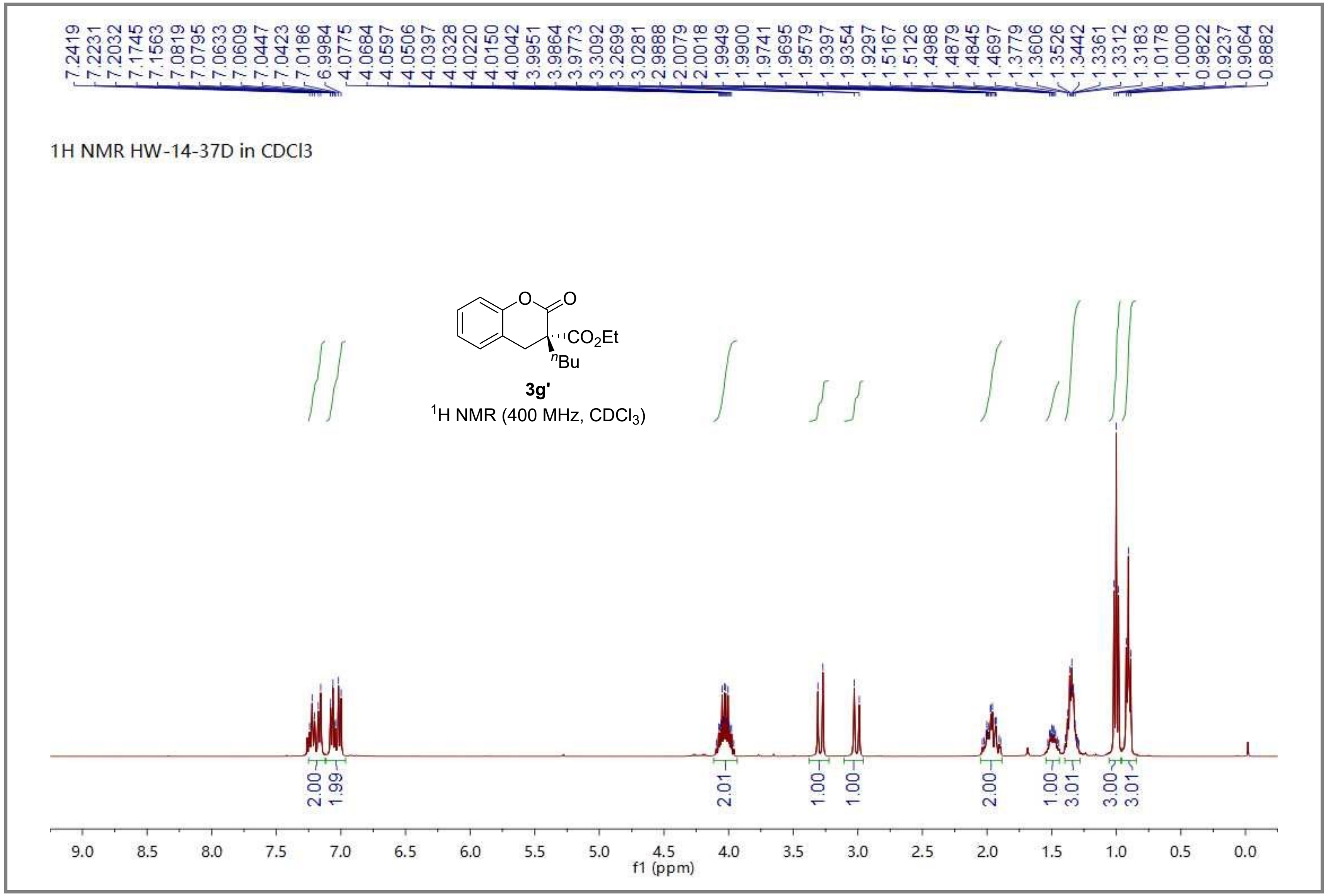

1H NMR HW-14-37D in CDCl3
3g'
^{1}H NMR (400 MHz, CDCl$_3$)
f1 (ppm)

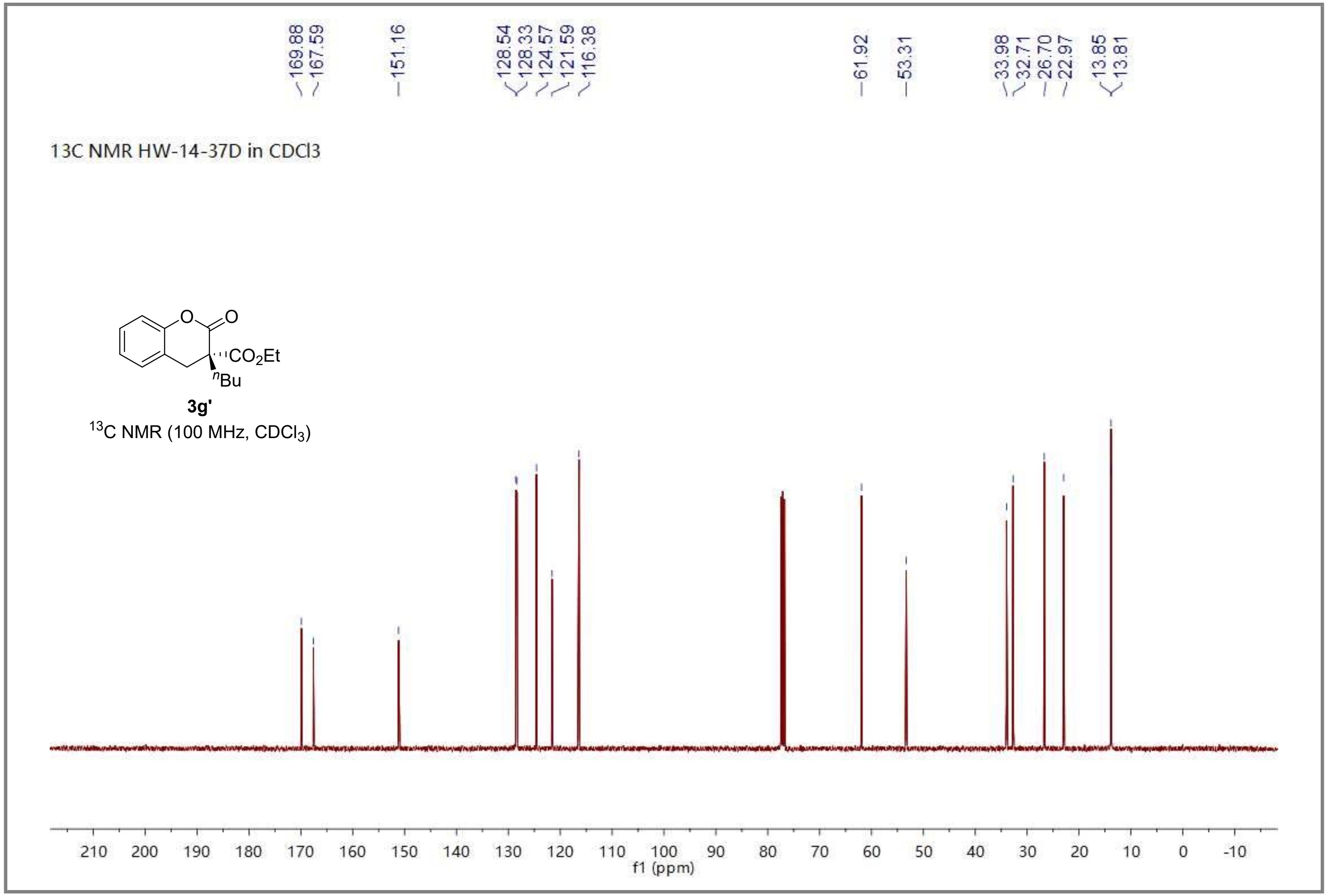
169.88
167.59
151.16
128.54
128.33
124.57
121.59
116.38
61.92
53.31
33.98
32.71
26.70
22.97
13.85
13.81
13C NMR HW-14-37D in CDCl3
O
O
CO2Et
nBu
3g'
13C NMR (100 MHz, CDCl3)
210 200 190 180 170 160 150 140 130 120 110 100 90 80 70 60 50 40 30 20 10 0 -10
f1 (ppm)

Data File C:\CHEM32\1\DATA\ZHOU-23\YZN022943.D
Sample Name: HW-14-41C SM+/-

===

Acq. Operator :
Acq. Instrument : Instrument 1 Location : -
Injection Date : 4/22/2023 9:33:01 AM
Acq. Method : C:\CHEM32\1\METHODS\DEF_LC.M
Last changed : 4/22/2023 9:32:44 AM
(modified after loading)
Analysis Method : C:\CHEM32\1\METHODS\DEF_LC.M
Last changed : 10/17/2023 9:19:47 AM
(modified after loading)
Sample Info : AS-H, Hexane/i-PrOH = 90/10, 0.8mL/min, 30 oC, 230 nm

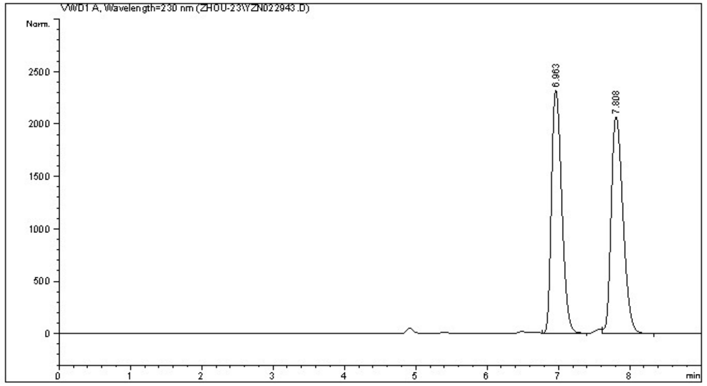

===
Area Percent Report
===

Sorted By : Signal
Multiplier: : 1.0000
Dilution: : 1.0000
Use Multiplier & Dilution Factor with ISTDs

Signal 1: VWD1 A, Wavelength=230 nm

Peak #	RetTime [min]	Type	Width [min]	Area mAU *s	Height [mAU]	Area %
1	6.963	VV	0.1572	2.32125e4	2325.45605	49.3297
2	7.808	VV	0.1810	2.38433e4	2069.13452	50.6703

Totals : 4.70558e4 4394.59058

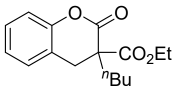

(+/-)-3g

===
*** End of Report ***

Instrument 1 10/17/2023 9:19:50 AM Page 1 of 1

Data File C:\CHEM32\1\DATA\ZHOU-23\YZN022974.D
Sample Name: HW-14-41C SM'

===

Acq. Operator :
Acq. Instrument : Instrument 1 Location : -
Injection Date : 4/26/2023 4:05:12 AM
Acq. Method : C:\CHEM32\1\METHODS\DEF_LC.M
Last changed : 4/26/2023 4:04:48 AM
(modified after loading)
Analysis Method : C:\CHEM32\1\METHODS\DEF_LC.M
Last changed : 10/17/2023 9:20:58 AM
(modified after loading)
Sample Info : AS-H, Hexane/i-PrOH = 90/10, 0.8 mL/min, 30 oC, 230 nm

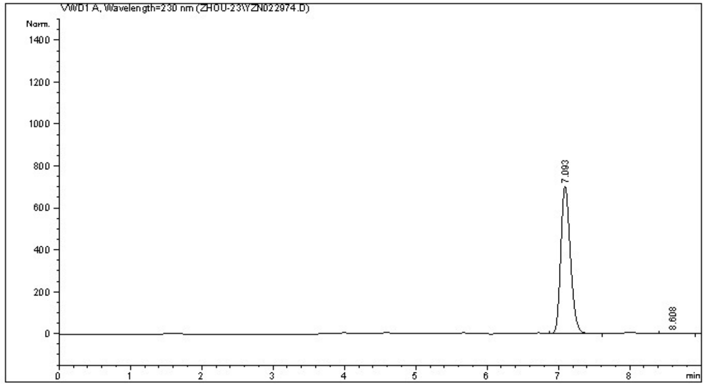

===
Area Percent Report
===

Sorted By : Signal
Multiplier: : 1.0000
Dilution: : 1.0000
Use Multiplier & Dilution Factor with ISTDs

Signal 1: VWD1 A, Wavelength=230 nm

Peak #	RetTime [min]	Type	Width [min]	Area mAU *s	Height [mAU]	Area %
1	7.093	VV	0.1444	6612.80273	704.59741	99.4928
2	8.608	VB	0.1508	33.71329	3.43738	0.5072

Totals : 6646.51603 708.03480

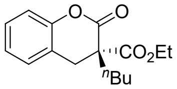

(+)-3g'

===
*** End of Report ***

Instrument 1 10/17/2023 9:21:04 AM Page 1 of 1

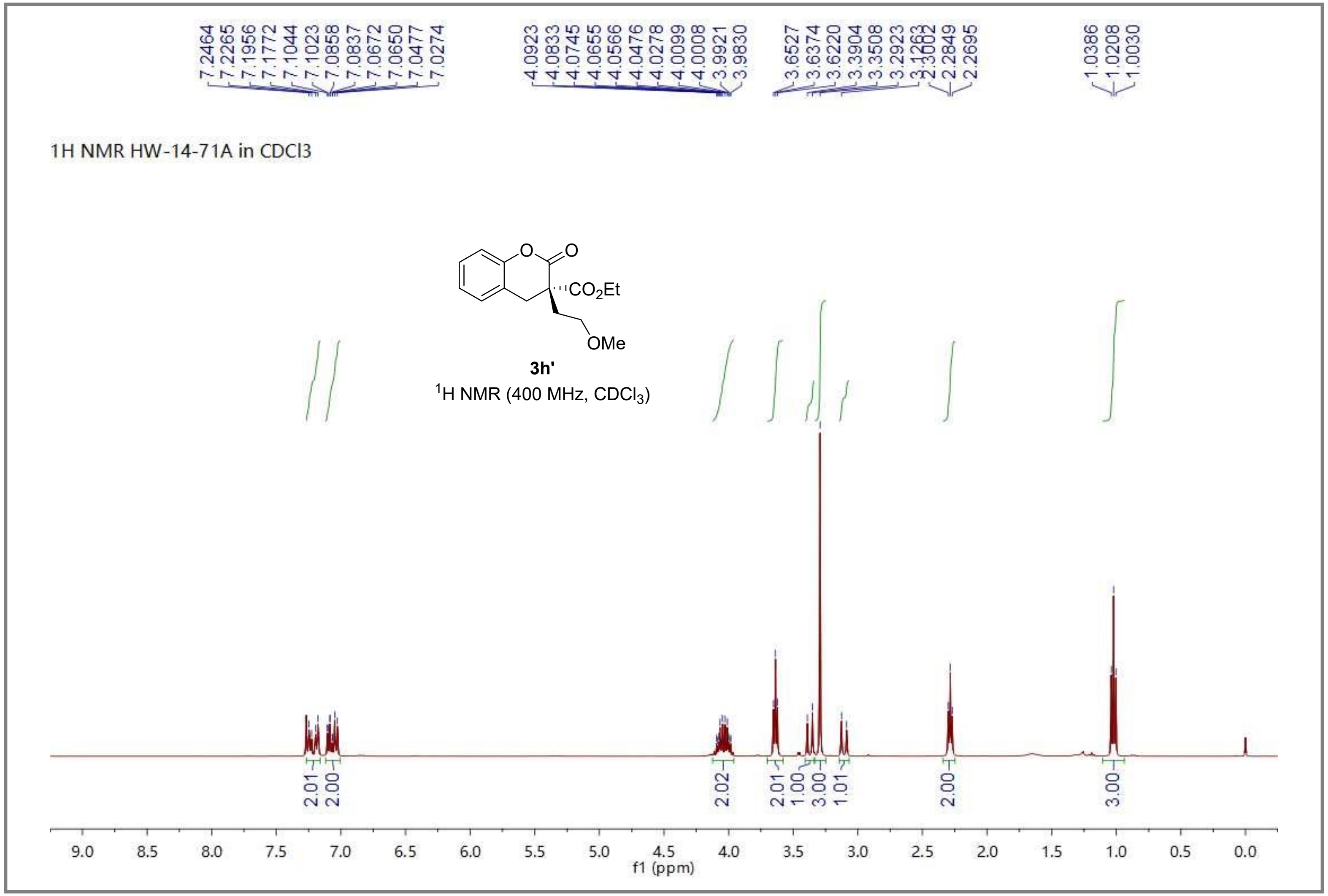

7.2464
7.2265
7.1956
7.1772
7.1044
7.1023
7.0858
7.0837
7.0672
7.0650
7.0477
7.0274
4.0923
4.0833
4.0745
4.0655
4.0566
4.0476
4.0278
4.0099
4.0008
3.9921
3.9830
3.6527
3.6374
3.6220
3.3904
3.3508
3.2923
3.1263
3.3002
2.2849
2.2695
1.0386
1.0208
1.0030
1H NMR HW-14-71A in CDCl3
CO2Et
OMe
3h'
1H NMR (400 MHz, CDCl3)
2.01
2.00
2.02
2.01
1.00
3.00
1.01
2.00
3.00
9.0
8.5
8.0
7.5
7.0
6.5
6.0
5.5
5.0
4.5
4.0
3.5
3.0
2.5
2.0
1.5
1.0
0.5
0.0
f1 (ppm)

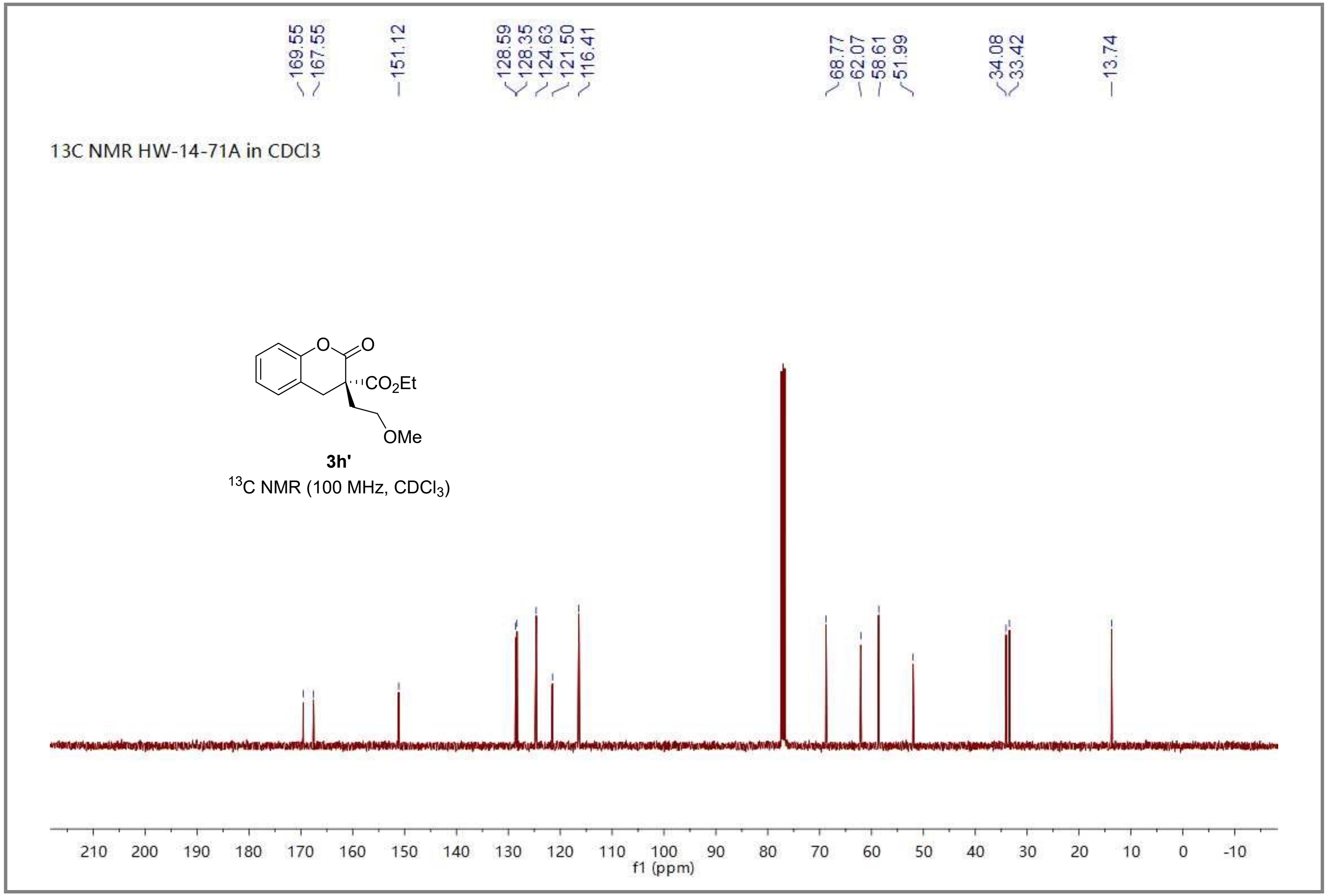
169.55
167.55
151.12
128.59
128.35
124.63
121.50
116.41
68.77
62.07
58.61
51.99
34.08
33.42
13.74
13C NMR HW-14-71A in CDCl3
CO2Et
OMe
3h'
13C NMR (100 MHz, CDCl3)
210 200 190 180 170 160 150 140 130 120 110 100 90 80 70 60 50 40 30 20 10 0 -10
f1 (ppm)

Data File C:\CHEM32\1\DATA\ZHOU-23\YZN023196.D
Sample Name: HW-14-70A +/-

===

Acq. Operator :
Acq. Instrument : Instrument 1 Location : -
Injection Date : 5/26/2023 4:31:55 AM
Acq. Method : C:\CHEM32\1\METHODS\DEF_LC.M
Last changed : 5/26/2023 4:31:26 AM
(modified after loading)
Analysis Method : C:\CHEM32\1\METHODS\DEF_LC.M
Last changed : 10/17/2023 9:28:03 AM
(modified after loading)
Sample Info : AS-H, Hexane/i-PrOH = 90/10, 0.8 mL/min, 30 oC, 230 nm

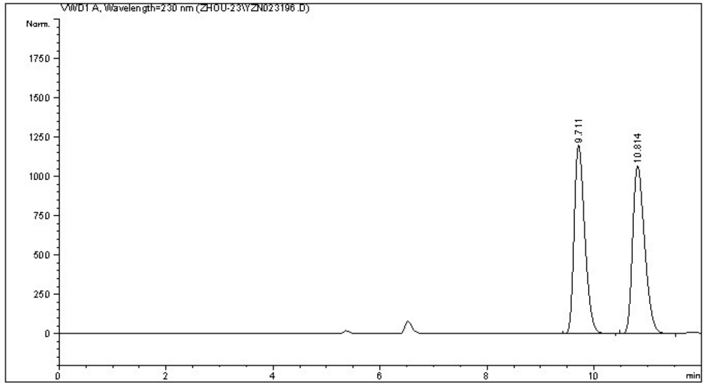

===

Area Percent Report

===

Sorted By : Signal
Multiplier: : 1.0000
Dilution: : 1.0000
Use Multiplier & Dilution Factor with ISTDs

Signal 1: VWD1 A, Wavelength=230 nm

Peak #	RetTime [min]	Type	Width [min]	Area mAU *s	Height [mAU]	Area %
1	9.711	BB	0.2129	1.64655e4	1196.65088	49.8354
2	10.814	BV	0.2412	1.65743e4	1065.27185	50.1646
Totals :				3.30399e4	2261.92273	

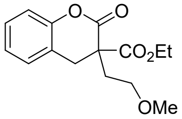

(+/-)-3h

===

*** End of Report ***

Instrument 1 10/17/2023 9:28:06 AM Page 1 of 1

Data File C:\CHEM32\1\DATA\ZHOU-23\YZN023302.D
Sample Name: HW-14-80 SM

===

Acq. Operator :
Acq. Instrument : Instrument 1 Location : -
Injection Date : 6/13/2023 7:49:12 AM
Acq. Method : C:\CHEM32\1\METHODS\DEF_LC.M
Last changed : 6/13/2023 7:44:25 AM
(modified after loading)
Analysis Method : C:\CHEM32\1\METHODS\DEF_LC.M
Last changed : 11/30/2023 9:02:08 AM
(modified after loading)
Sample Info : AS-H, Hexane/i-PrOH = 90/10, 0.8 mL/min, 30 oC, 230 nm

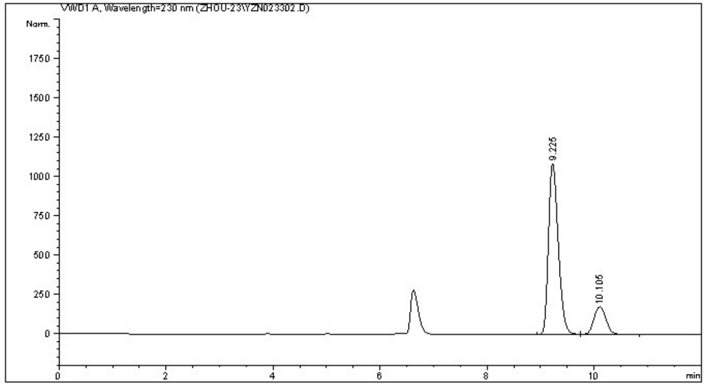

===

Area Percent Report

===

Sorted By : Signal
Multiplier: : 1.0000
Dilution: : 1.0000
Use Multiplier & Dilution Factor with ISTDs

Signal 1: VWD1 A, Wavelength=230 nm

Peak #	RetTime [min]	Type	Width [min]	Area mAU *s	Height [mAU]	Area %
1	9.225	VV	0.1976	1.38067e4	1087.73401	83.2211
2	10.105	VB	0.2539	2783.67700	175.13556	16.7789
Totals :				1.65904e4	1262.86957	

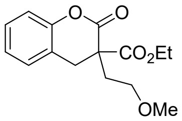

(+)-3h'

===

*** End of Report ***

Instrument 1 11/30/2023 9:02:11 AM Page 1 of 1

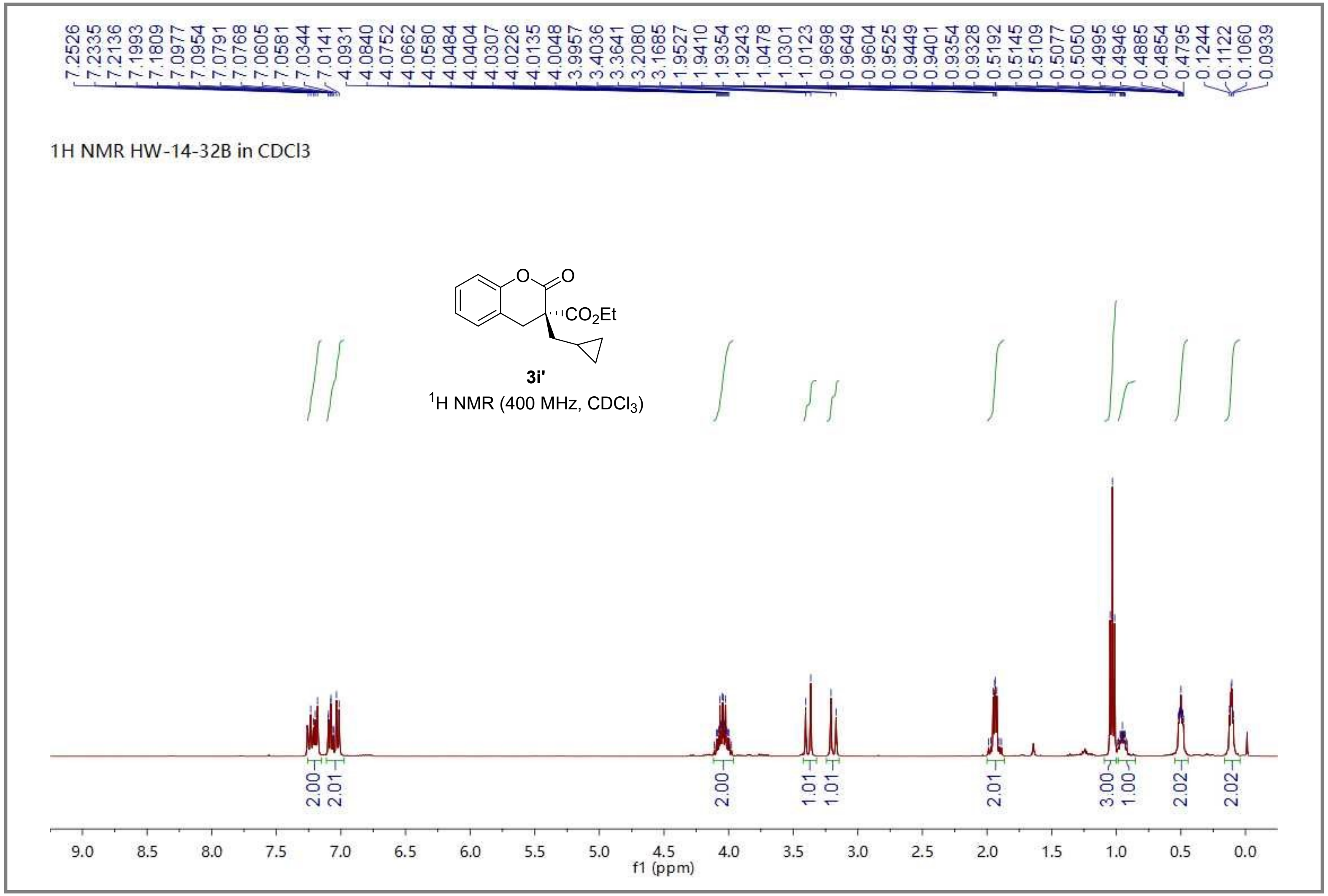

7.2526
7.2335
7.2136
7.1993
7.1809
7.0977
7.0954
7.0791
7.0768
7.0605
7.0581
7.0344
7.0141
4.0931
4.0840
4.0752
4.0662
4.0580
4.0484
4.0404
4.0307
4.0226
4.0135
4.0048
3.9957
3.4036
3.3641
3.2080
3.1685
1.9527
1.9410
1.9354
1.9243
1.0478
1.0301
1.0123
0.9698
0.9649
0.9604
0.9525
0.9449
0.9401
0.9354
0.9328
0.5192
0.5145
0.5109
0.5077
0.5050
0.4995
0.4946
0.4885
0.4854
0.4795
0.1244
0.1122
0.1060
0.0939
1H NMR HW-14-32B in CDCl3
O
O
CO2Et
3i'
1H NMR (400 MHz, CDCl3)
2.00
2.01
2.00
1.01
1.01
2.01
3.00
1.00
2.02
2.02
9.0
8.5
8.0
7.5
7.0
6.5
6.0
5.5
5.0
4.5
4.0
3.5
3.0
2.5
2.0
1.5
1.0
0.5
0.0
f1 (ppm)

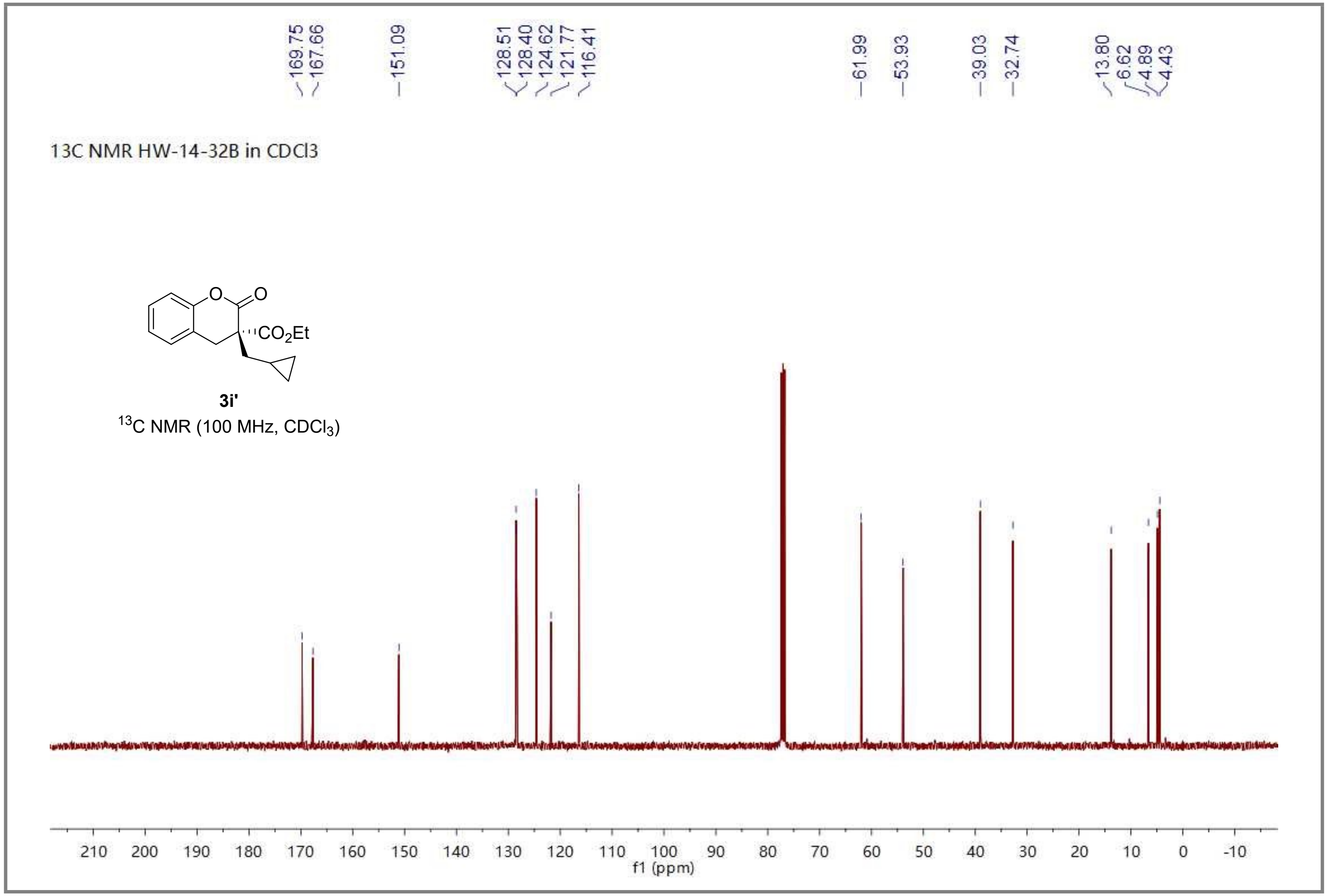
169.75
167.66
151.09
128.51
128.40
124.62
121.77
116.41
61.99
53.93
39.03
32.74
13.80
6.62
4.89
4.43
13C NMR HW-14-32B in CDCl3
O
O
CO2Et
3i'
13C NMR (100 MHz, CDCl3)
210 200 190 180 170 160 150 140 130 120 110 100 90 80 70 60 50 40 30 20 10 0 -10
f1 (ppm)

Data File C:\CHEM32\1\DATA\ZHOU-23\YZN022941.D
Sample Name: HW-14-34B SM+/-

===

Acq. Operator :
Acq. Instrument : Instrument 1 Location : -
Injection Date : 4/22/2023 8:53:22 AM
Acq. Method : C:\CHEM32\1\METHODS\DEF_LC.M
Last changed : 4/22/2023 8:52:29 AM
(modified after loading)
Analysis Method : C:\CHEM32\1\METHODS\DEF_LC.M
Last changed : 10/17/2023 9:32:09 AM
(modified after loading)
Sample Info : AS-H, Hexane/i-PrOH = 90/10, 0.8mL/min, 30 oC, 230 nm

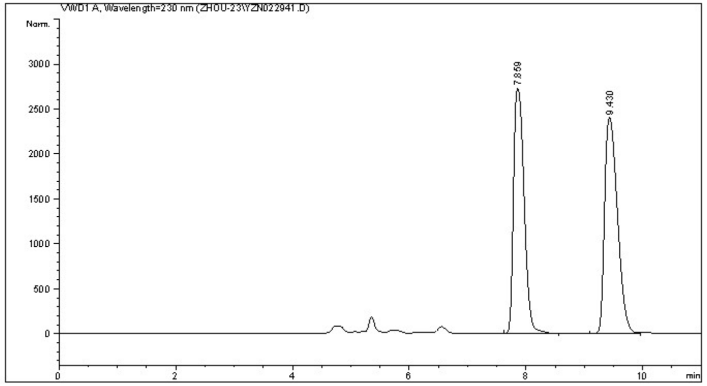

===
Area Percent Report
===

Sorted By : Signal
Multiplier: : 1.0000
Dilution: : 1.0000
Use Multiplier & Dilution Factor with ISTDs

Signal 1: VWD1 A, Wavelength=230 nm

Peak #	RetTime [min]	Type	Width [min]	Area mAU *s	Height [mAU]	Area %
1	7.859	VV	0.1994	3.43748e4	2727.70850	48.4090
2	9.430	VV	0.2419	3.66344e4	2403.72900	51.5910
Totals :				7.10092e4	5131.43750	

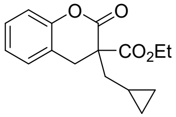

(+/-)-3i

===
*** End of Report ***

Instrument 1 10/17/2023 9:32:12 AM Page 1 of 1

Data File C:\CHEM32\1\DATA\ZHOU-23\YZN023007.D
Sample Name: HW-14-47A SM

===

Acq. Operator :
Acq. Instrument : Instrument 1 Location : -
Injection Date : 5/5/2023 3:58:08 AM
Acq. Method : C:\CHEM32\1\METHODS\DEF_LC.M
Last changed : 5/5/2023 3:57:05 AM
(modified after loading)
Analysis Method : C:\CHEM32\1\METHODS\DEF_LC.M
Last changed : 10/17/2023 9:34:49 AM
(modified after loading)
Sample Info : AS-H, Hexane/i-PrOH = 90/10, 0.8 mL/min, 30 oC, 230nm

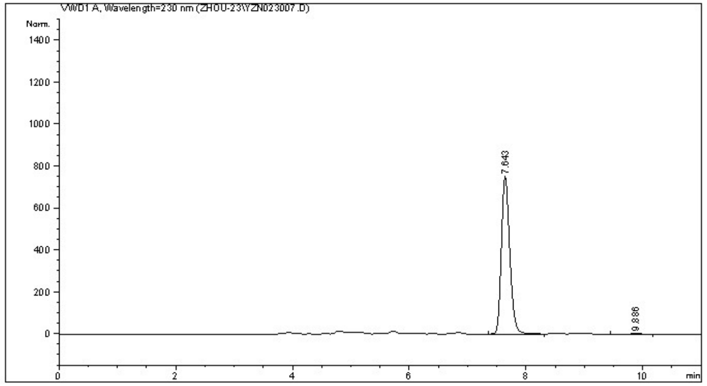

===
Area Percent Report
===

Sorted By : Signal
Multiplier: : 1.0000
Dilution: : 1.0000
Use Multiplier & Dilution Factor with ISTDs

Signal 1: VWD1 A, Wavelength=230 nm

Peak #	RetTime [min]	Type	Width [min]	Area mAU *s	Height [mAU]	Area %
1	7.643	VV	0.1588	7590.19287	750.01428	99.3829
2	9.886	VV	0.2269	47.12951	2.95362	0.6171
Totals :				7637.32238	752.96790	

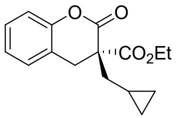

(+)-3i'

===
*** End of Report ***

Instrument 1 10/17/2023 9:34:51 AM Page 1 of 1

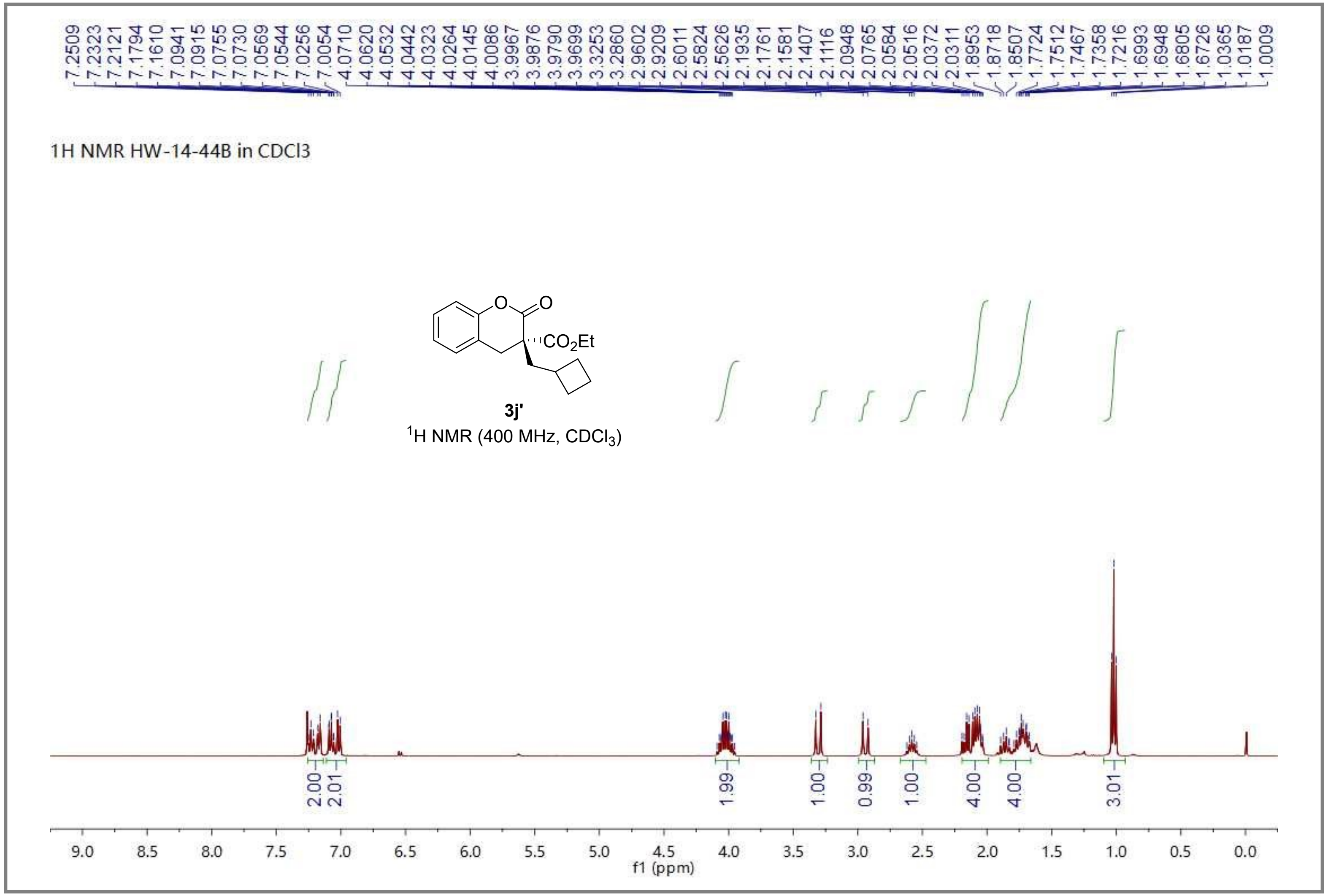

7.2509 7.2323 7.2121 7.1794 7.1610 7.0941 7.0915 7.0755 7.0730 7.0569 7.0544 7.0256 7.0054 4.0710 4.0620 4.0532 4.0442 4.0323 4.0264 4.0145 4.0086 3.9967 3.9876 3.9790 3.9699 3.3253 3.2860 2.9602 2.9209 2.6011 2.5824 2.5626 2.1935 2.1761 2.1581 2.1407 2.1116 2.0948 2.0765 2.0584 2.0516 2.0372 2.0311 1.8953 1.8718 1.8507 1.7724 1.7512 1.7467 1.7358 1.7216 1.6993 1.6948 1.6805 1.6726 1.0365 1.0187 1.0009
1H NMR HW-14-44B in CDCl3
CO2Et
O
O
3j'
1H NMR (400 MHz, CDCl3)
2.00 2.01 1.99 1.00 0.99 1.00 4.00 4.00 3.01
9.0 8.5 8.0 7.5 7.0 6.5 6.0 5.5 5.0 4.5 4.0 3.5 3.0 2.5 2.0 1.5 1.0 0.5 0.0
f1 (ppm)

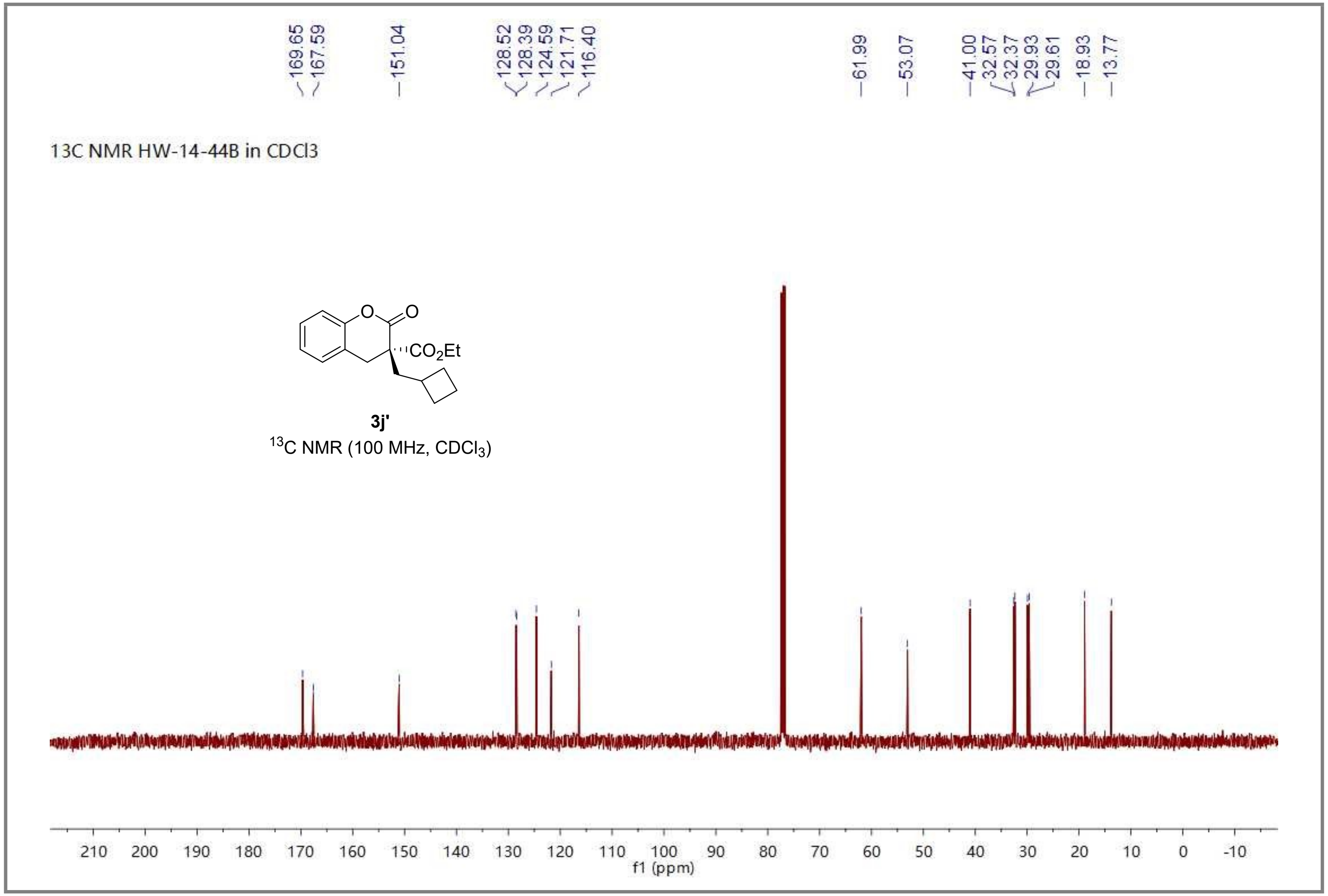

169.65
167.59
151.04
128.52
128.39
124.59
121.71
116.40
61.99
53.07
41.00
32.57
32.37
29.93
29.61
18.93
13.77
13C NMR HW-14-44B in CDCl3
CO2Et
O
O
3j'
13C NMR (100 MHz, CDCl3)
210 200 190 180 170 160 150 140 130 120 110 100 90 80 70 60 50 40 30 20 10 0 -10
f1 (ppm)

Data File C:\CHEM32\1\DATA\ZHOU-23\YZN023005.D
Sample Name: HW-14-47B SM+/-

===

Acq. Operator :
Acq. Instrument : Instrument 1 Location : -
Injection Date : 5/5/2023 3:26:16 AM
Acq. Method : C:\CHEM32\1\METHODS\DEF_LC.M
Last changed : 5/5/2023 3:17:54 AM
(modified after loading)
Analysis Method : C:\CHEM32\1\METHODS\DEF_LC.M
Last changed : 10/17/2023 9:39:16 AM
(modified after loading)
Sample Info : AS-H, Hexane/i-PrOH = 90/10, 0.8 mL/min, 30 oC, 230nm

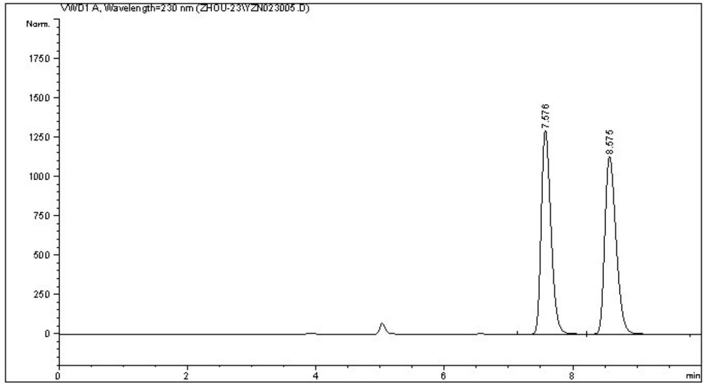

===

Area Percent Report

===

Sorted By : Signal
Multiplier: : 1.0000
Dilution: : 1.0000
Use Multiplier & Dilution Factor with ISTDs

Signal 1: VWD1 A, Wavelength=230 nm

Peak #	RetTime [min]	Type	Width [min]	Area mAU *s	Height [mAU]	Area %
1	7.576	VV	0.1639	1.36363e4	1291.87915	49.7430
2	8.575	VV	0.1891	1.37773e4	1127.55725	50.2570
Totals :				2.74136e4	2419.43640	

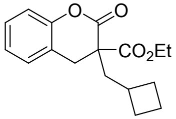

(+/-)-3j

===

*** End of Report ***

Instrument 1 10/17/2023 9:39:19 AM Page 1 of 1

Data File C:\CHEM32\1\DATA\ZHOU-23\YZN023132.D
Sample Name: HW-14-56A SM

===

Acq. Operator :
Acq. Instrument : Instrument 1 Location : -
Injection Date : 5/20/2023 8:16:59 AM
Acq. Method : C:\CHEM32\1\METHODS\DEF_LC.M
Last changed : 5/20/2023 7:53:15 AM
(modified after loading)
Analysis Method : C:\CHEM32\1\METHODS\DEF_LC.M
Last changed : 10/17/2023 9:40:58 AM
(modified after loading)
Sample Info : AS-H, Hexane/i-PrOH = 90/10, 0.8 mL/min, 30 oC, 230 nm

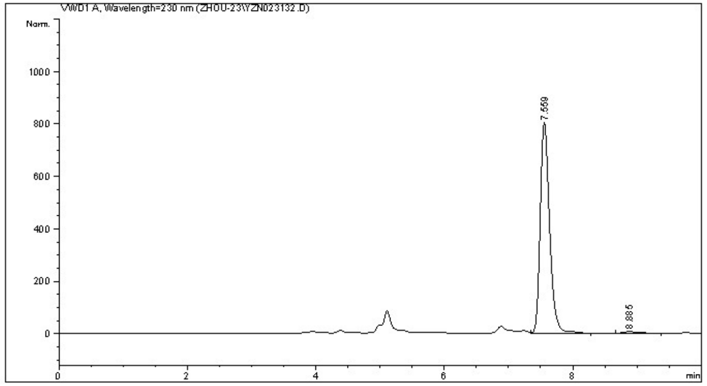

===

Area Percent Report

===

Sorted By : Signal
Multiplier: : 1.0000
Dilution: : 1.0000
Use Multiplier & Dilution Factor with ISTDs

Signal 1: VWD1 A, Wavelength=230 nm

Peak #	RetTime [min]	Type	Width [min]	Area mAU *s	Height [mAU]	Area %
1	7.559	VV	0.1608	8269.88477	803.69092	97.7994
2	8.885	VV	0.3327	186.08423	7.74540	2.2006
Totals :				8455.96899	811.43632	

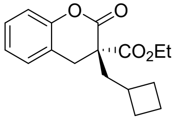

(+)-3j'

===

*** End of Report ***

Instrument 1 10/17/2023 9:41:01 AM Page 1 of 1

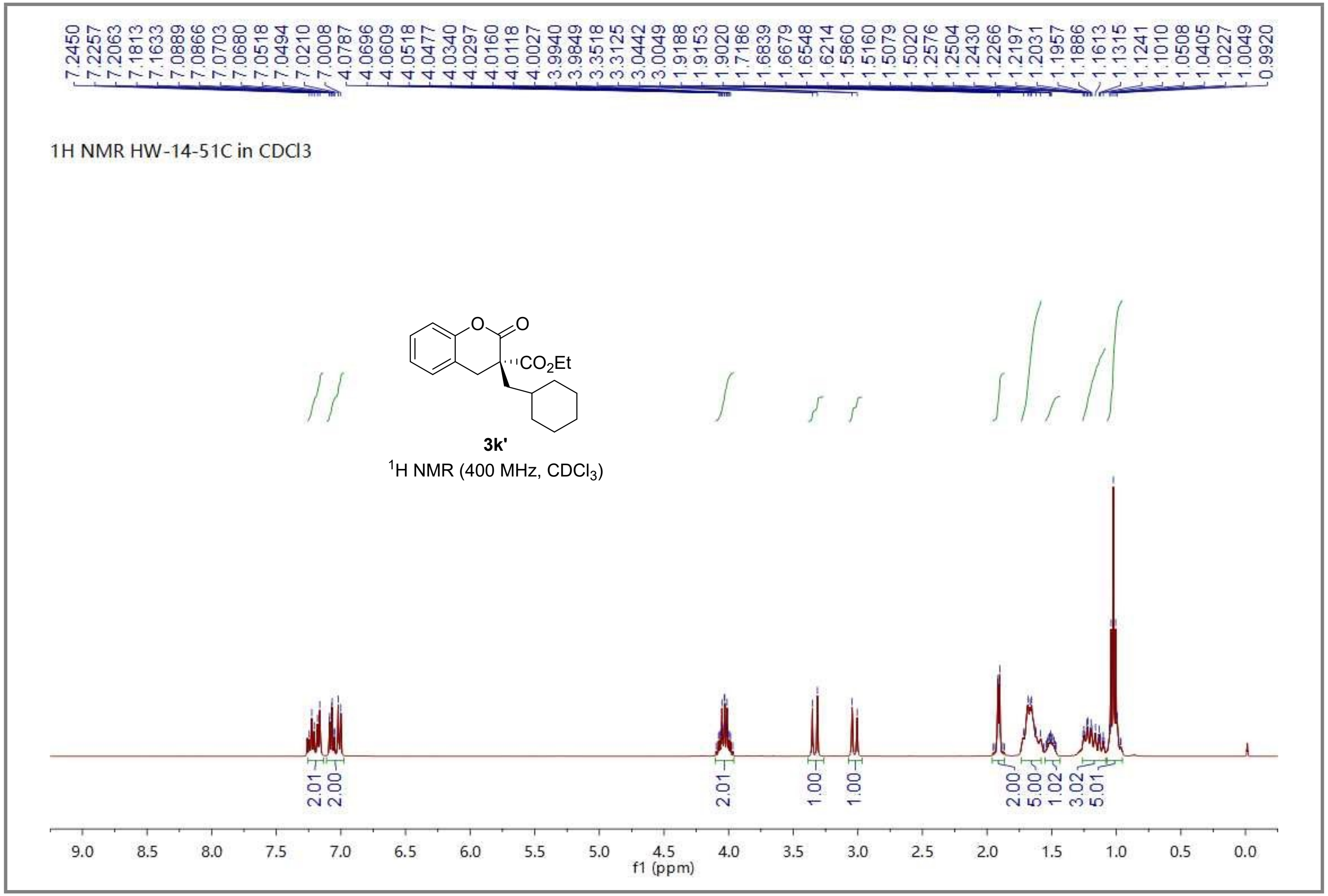

1H NMR HW-14-51C in CDCl3
3k'
1H NMR (400 MHz, CDCl3)
f1 (ppm)

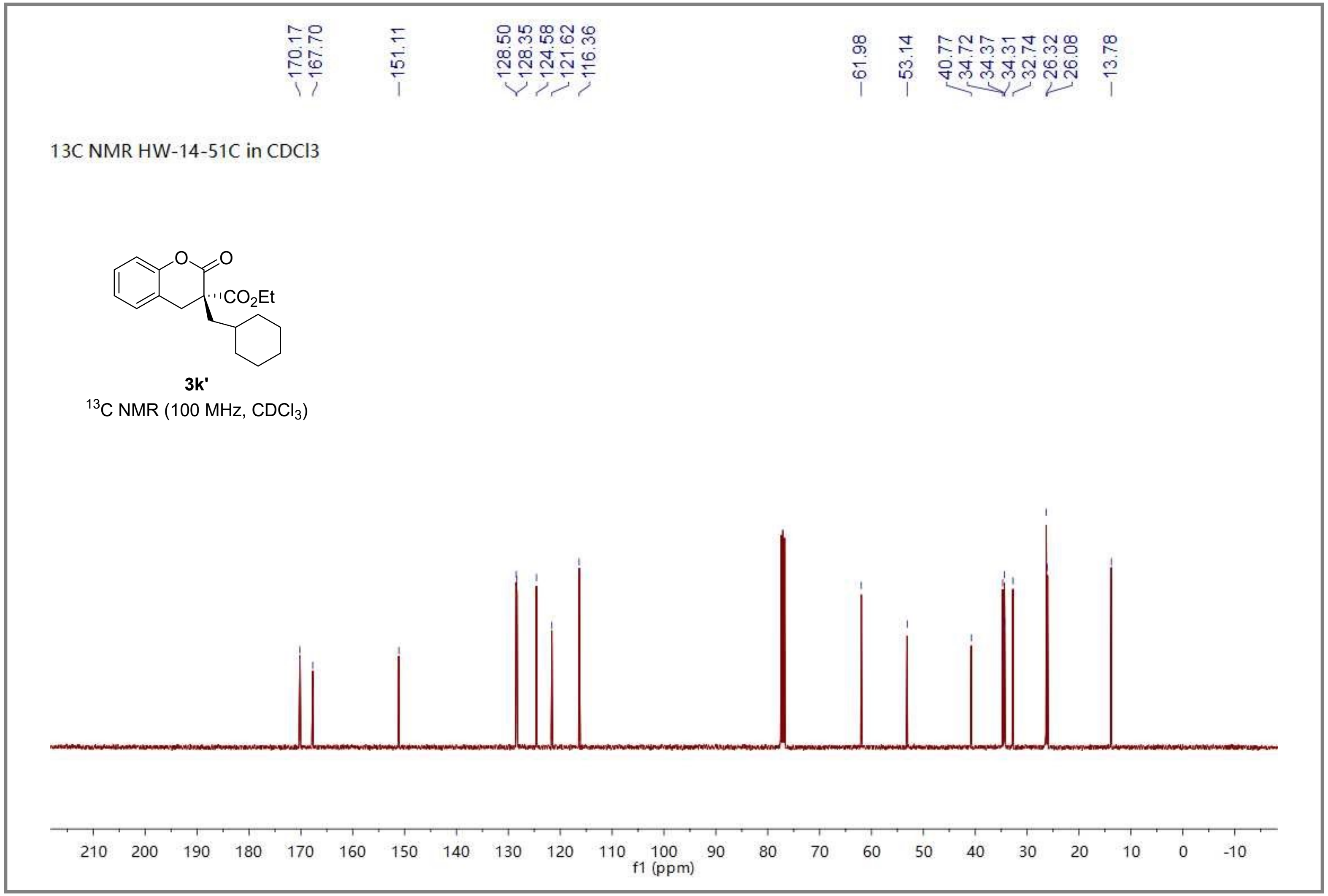
170.17
167.70
151.11
128.50
128.35
124.58
121.62
116.36
61.98
53.14
40.77
34.72
34.37
34.31
32.74
26.32
26.08
13.78
13C NMR HW-14-51C in CDCl3
O
O
CO2Et
3k'
13C NMR (100 MHz, CDCl3)
210 200 190 180 170 160 150 140 130 120 110 100 90 80 70 60 50 40 30 20 10 0 -10
f1 (ppm)

Data File C:\CHEM32\1\DATA\ZHOU-23\YZN023146.D
Sample Name: HW-14-56B SM+/-

===

Acq. Operator :
Acq. Instrument : Instrument 1 Location : -
Injection Date : 5/21/2023 1:46:25 AM
Acq. Method : C:\CHEM32\1\METHODS\DEF_LC.M
Last changed : 5/21/2023 1:37:19 AM
(modified after loading)
Analysis Method : C:\CHEM32\1\METHODS\DEF_LC.M
Last changed : 10/17/2023 9:44:31 AM
(modified after loading)
Sample Info : OD-H, Hexane/i-PrOH = 90/10, 0.8 mL/min, 30 oC, 230 nm

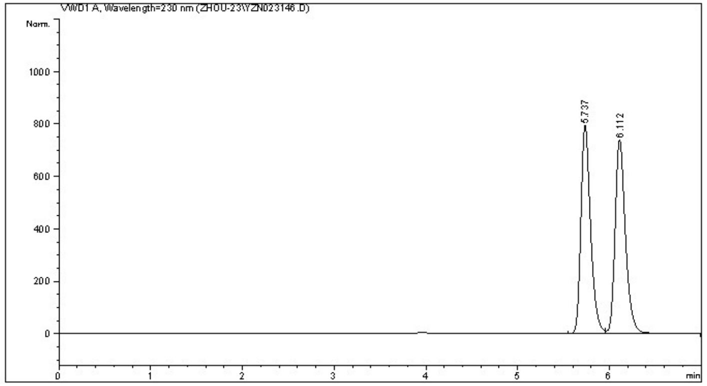

===
Area Percent Report
===

Sorted By : Signal
Multiplier: : 1.0000
Dilution: : 1.0000
Use Multiplier & Dilution Factor with ISTDs

Signal 1: VWD1 A, Wavelength=230 nm

Peak #	RetTime [min]	Type	Width [min]	Area mAU *s	Height [mAU]	Area %
1	5.737	VV	0.1148	5801.85840	789.97021	49.8961
2	6.112	VV	0.1271	5826.03174	737.68420	50.1039
Totals :				1.16279e4	1527.65442	

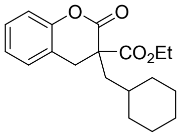

(+/-)-3k

===
*** End of Report ***

Instrument 1 10/17/2023 9:44:35 AM Page 1 of 1

Data File C:\CHEM32\1\DATA\ZHOU-23\YZN023218.D
Sample Name: HW-14-73C SM

===

Acq. Operator :
Acq. Instrument : Instrument 1 Location : -
Injection Date : 5/30/2023 9:35:19 AM
Acq. Method : C:\CHEM32\1\METHODS\DEF_LC.M
Last changed : 5/30/2023 9:35:01 AM
(modified after loading)
Analysis Method : C:\CHEM32\1\METHODS\DEF_LC.M
Last changed : 10/17/2023 9:45:28 AM
(modified after loading)
Sample Info : OD-H, Hexane/i-PrOH = 90/10, 0.8 mL/min, 30 oC, 230 nm

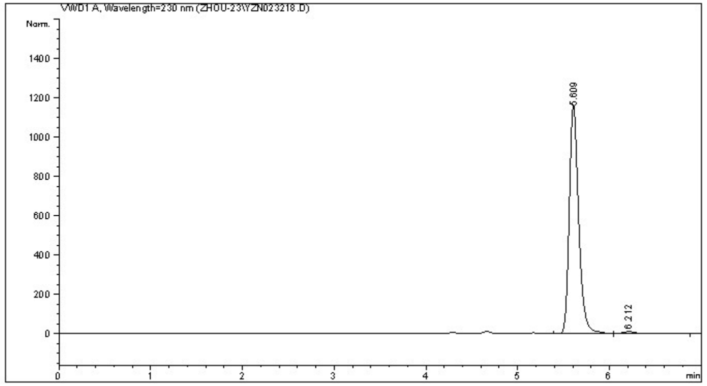

===
Area Percent Report
===

Sorted By : Signal
Multiplier: : 1.0000
Dilution: : 1.0000
Use Multiplier & Dilution Factor with ISTDs

Signal 1: VWD1 A, Wavelength=230 nm

Peak #	RetTime [min]	Type	Width [min]	Area mAU *s	Height [mAU]	Area %
1	5.609	VV	0.1078	8286.52246	1143.56421	98.4766
2	6.212	VV	0.1696	128.19243	11.10791	1.5234
Totals :				8414.71489	1154.67212	

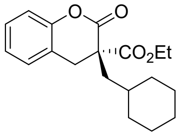

(+)-3k'

===
*** End of Report ***

Instrument 1 10/17/2023 9:45:31 AM Page 1 of 1

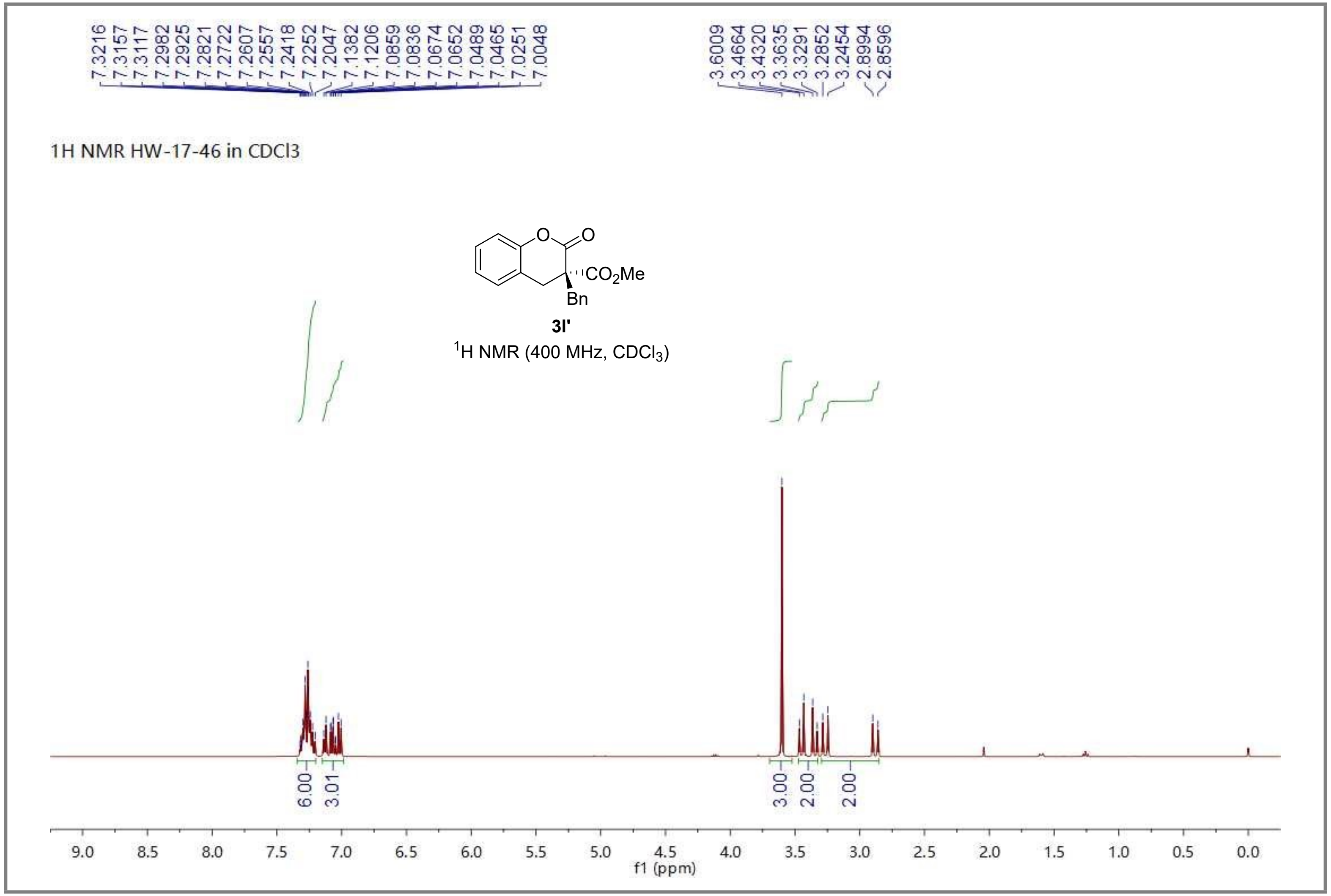
7.3216
7.3157
7.3117
7.2982
7.2925
7.2821
7.2722
7.2607
7.2557
7.2418
7.2252
7.2047
7.1382
7.1206
7.0859
7.0836
7.0674
7.0652
7.0489
7.0465
7.0251
7.0048
3.6009
3.4664
3.4320
3.3635
3.3291
3.2852
3.2454
2.8994
2.8596
1H NMR HW-17-46 in CDCl3
O
O
CO2Me
Bn
3l'
1H NMR (400 MHz, CDCl3)
6.00
3.01
3.00
2.00
2.00
9.0
8.5
8.0
7.5
7.0
6.5
6.0
5.5
5.0
4.5
4.0
3.5
3.0
2.5
2.0
1.5
1.0
0.5
0.0
f1 (ppm)

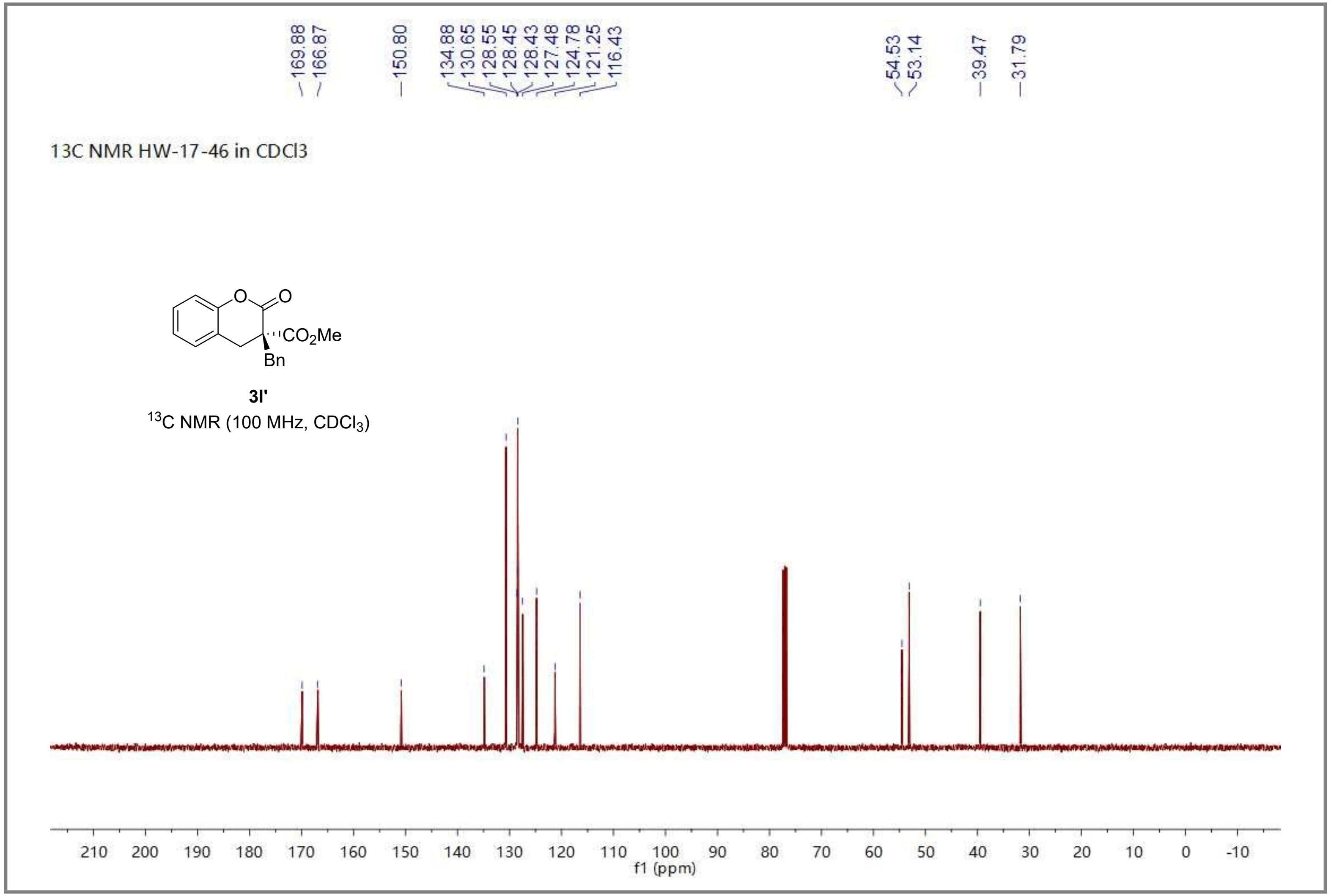

169.88
166.87
150.80
134.88
130.65
128.55
128.45
128.43
127.48
124.78
121.25
116.43
54.53
53.14
39.47
31.79
13C NMR HW-17-46 in CDCl3
O
O
CO2Me
Bn
3l'
13C NMR (100 MHz, CDCl3)
210 200 190 180 170 160 150 140 130 120 110 100 90 80 70 60 50 40 30 20 10 0 -10
f1 (ppm)

```
Data File D:\USERS\CHEMSTATION\1\DATA\ZHOU2024\YZ3009332  14-49-04.D
Sample Name: HW-17-48SM+/-

    =====================================================================
    Acq. Operator   : SYSTEM
    Sample Operator : SYSTEM
    Acq. Instrument : 1260II                        Location :     -
    Injection Date  : 4/27/2024 2:49:04 PM                Inj :    1
                                                  Inj Volume : No inj
    Acq. Method     : C:\Users\Public\Documents\ChemStation\1\Methods\def_LC.M
    Last changed    : 4/27/2024 2:41:24 PM by SYSTEM
                      (modified after loading)
    Analysis Method : C:\Users\Public\Documents\ChemStation\1\Methods\def_LC.M
    Last changed    : 4/27/2024 3:47:20 PM by SYSTEM
                      (modified after loading)
    Sample Info     : AS-H, n-Hexane/i-PrOH = 90/10, 0.8 mL/min, 30 oC, 230 nm

    Additional Info : Peak(s) manually integrated
```

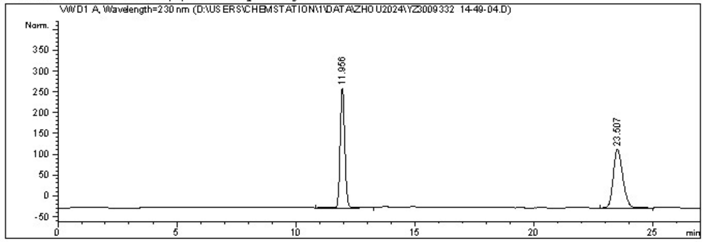


```
    =====================================================================
                             Area Percent Report
    =====================================================================

    Sorted By             :      Signal
    Multiplier            :      1.0000
    Dilution              :      1.0000
    Do not use Multiplier & Dilution Factor with ISTDs

    Signal 1: VWD1 A, Wavelength=230 nm
```

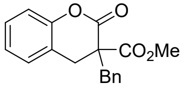

(+/-)-3l

Peak #	RetTime [min]	Type	Width [min]	Area [mAU*s]	Height [mAU]	Area %
1	11.956	VB R	0.2089	3111.81250	229.70885	50.1791
2	23.507	BV	0.4288	3089.59863	112.37350	49.8209

Totals : 6201.41113 342.08234

```
    =====================================================================
                             *** End of Report ***
```



```
Data File D:\USERS\CHEMSTATION\1\DATA\ZHOU2024\YZ3009333  15-20-32.D
Sample Name: HW-17-48SM

    =====================================================================
    Acq. Operator   : SYSTEM
    Sample Operator : SYSTEM
    Acq. Instrument : 1260II                        Location :     -
    Injection Date  : 4/27/2024 3:20:32 PM                Inj :    1
                                                  Inj Volume : No inj
    Acq. Method     : C:\Users\Public\Documents\ChemStation\1\Methods\def_LC.M
    Last changed    : 4/27/2024 2:41:24 PM by SYSTEM
                      (modified after loading)
    Analysis Method : C:\Users\Public\Documents\ChemStation\1\Methods\def_LC.M
    Last changed    : 4/27/2024 3:49:04 PM by SYSTEM
                      (modified after loading)
    Sample Info     : AS-H, n-Hexane/i-PrOH = 90/10, 0.8 mL/min, 30 oC, 230 nm

    Additional Info : Peak(s) manually integrated
```

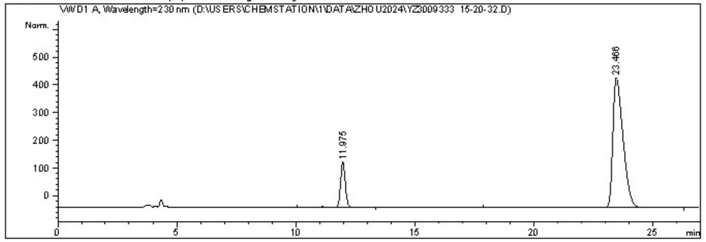


```
    =====================================================================
                             Area Percent Report
    =====================================================================

    Sorted By             :      Signal
    Multiplier            :      1.0000
    Dilution              :      1.0000
    Do not use Multiplier & Dilution Factor with ISTDs

    Signal 1: VWD1 A, Wavelength=230 nm
```

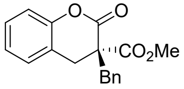

(+)-3l'

Peak #	RetTime [min]	Type	Width [min]	Area [mAU*s]	Height [mAU]	Area %
1	11.975	VV R	0.2160	1858.51172	133.44449	14.0102
2	23.466	VV R	0.4520	1.14069e4	382.56509	85.9898

Totals : 1.32655e4 516.00958

```
    =====================================================================
                             *** End of Report ***
```

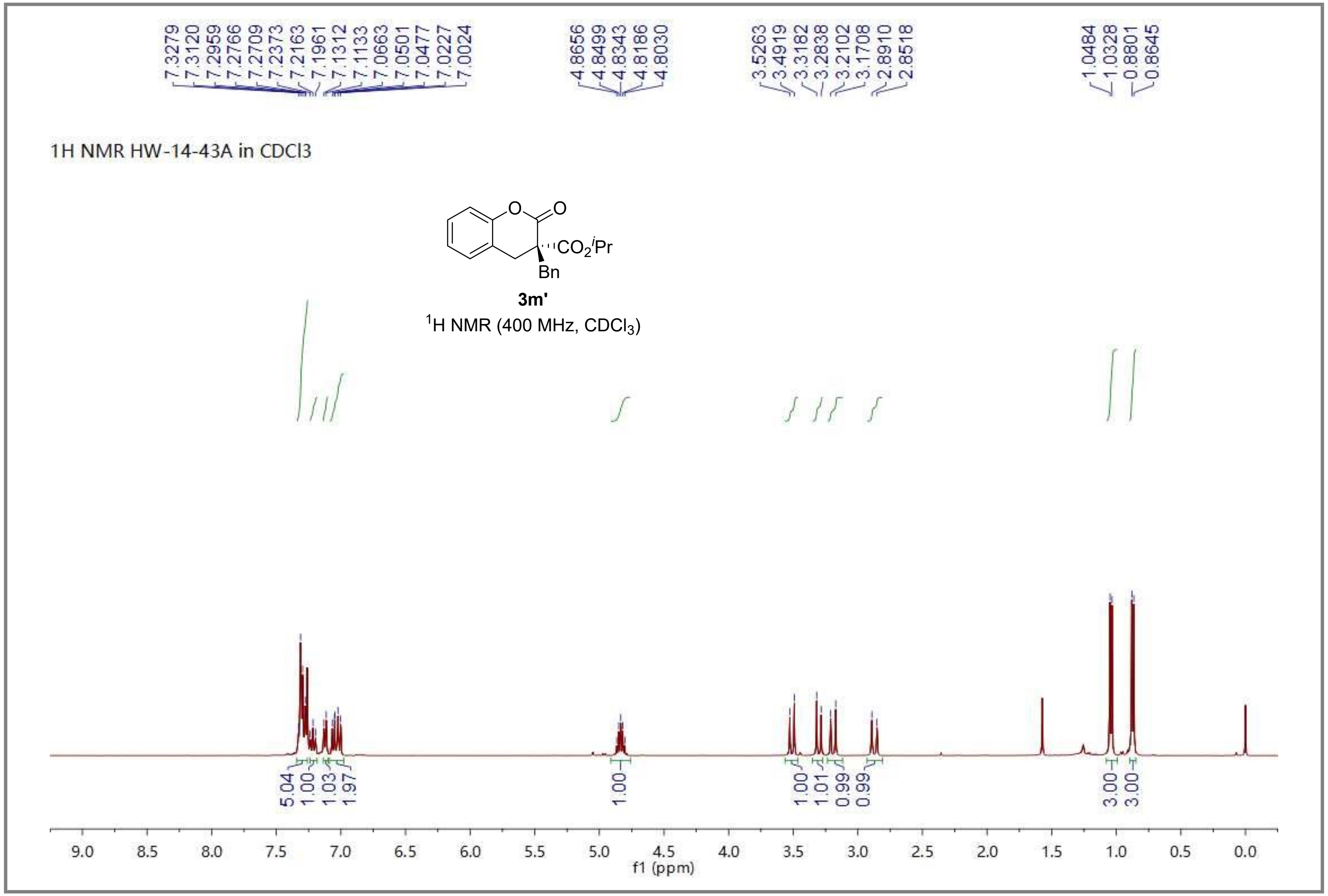

7.3279
7.3120
7.2959
7.2766
7.2709
7.2373
7.2163
7.1961
7.1312
7.1133
7.0663
7.0501
7.0477
7.0227
7.0024
4.8656
4.8499
4.8343
4.8186
4.8030
3.5263
3.4919
3.3182
3.2838
3.2102
3.1708
2.8910
2.8518
1.0484
1.0328
0.8801
0.8645
1H NMR HW-14-43A in CDCl3
O
O
CO2iPr
Bn
3m'
1H NMR (400 MHz, CDCl3)
5.04
1.00
1.03
1.97
1.00
1.00
1.01
0.99
0.99
3.00
3.00
9.0
8.5
8.0
7.5
7.0
6.5
6.0
5.5
5.0
4.5
4.0
3.5
3.0
2.5
2.0
1.5
1.0
0.5
0.0
f1 (ppm)

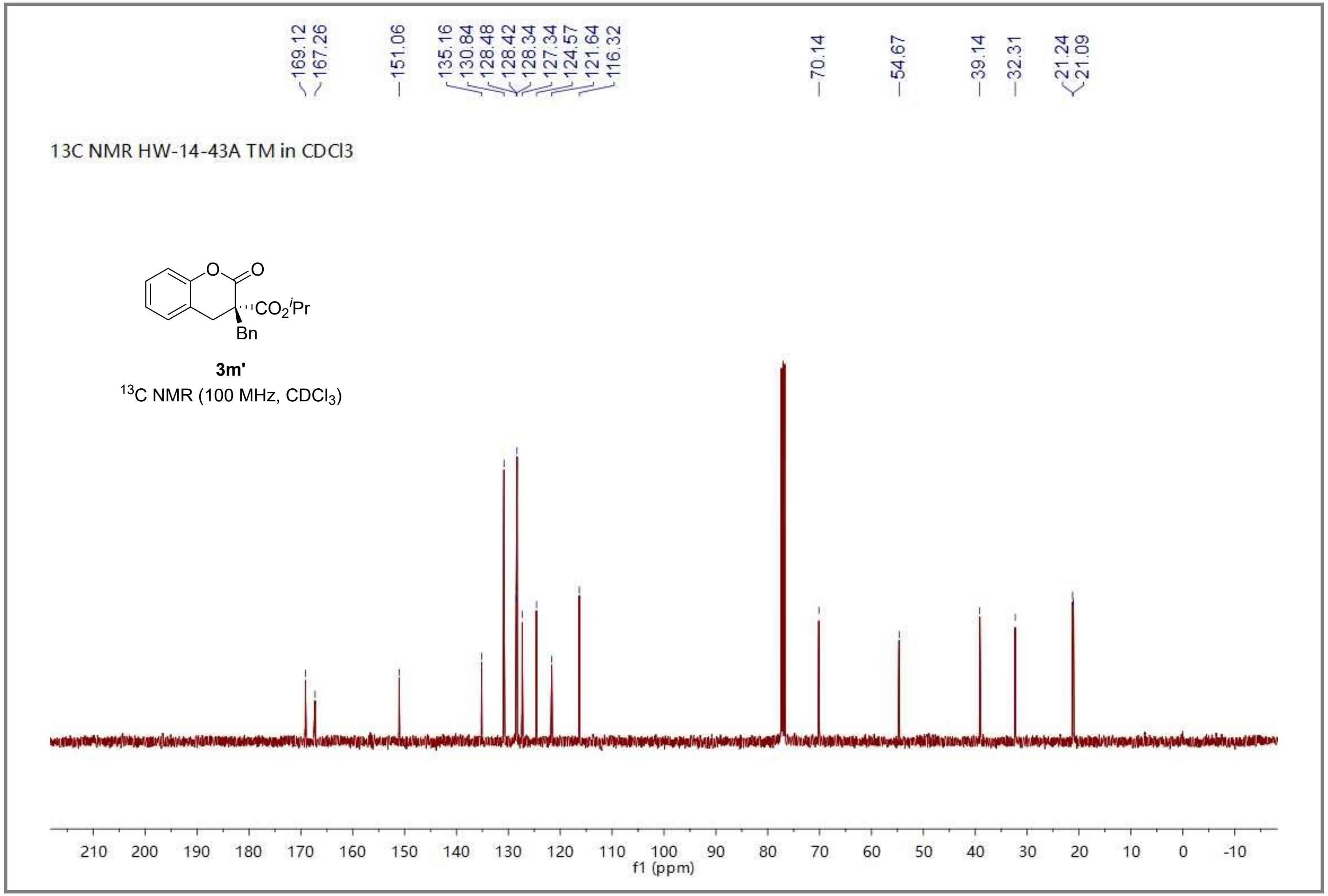
169.12
167.26
151.06
135.16
130.84
128.48
128.42
128.34
127.34
124.57
121.64
116.32
70.14
54.67
39.14
32.31
21.24
21.09
13C NMR HW-14-43A TM in CDCl3
O
O
CO2iPr
Bn
3m'
13C NMR (100 MHz, CDCl3)
210 200 190 180 170 160 150 140 130 120 110 100 90 80 70 60 50 40 30 20 10 0 -10
f1 (ppm)

Data File C:\CHEM32\1\DATA\ZHOU-23\YZN023011.D
Sample Name: HW-14-48A SM+/-

===

Acq. Operator :
Acq. Instrument : Instrument 1 Location : -
Injection Date : 5/5/2023 4:56:07 AM
Acq. Method : C:\CHEM32\1\METHODS\DEF_LC.M
Last changed : 5/5/2023 4:55:47 AM
(modified after loading)
Analysis Method : C:\CHEM32\1\METHODS\DEF_LC.M
Last changed : 10/17/2023 9:49:24 AM
(modified after loading)
Sample Info : AS-H, Hexane/i-PrOH = 90/10, 0.8 mL/min, 30 oC, 230nm

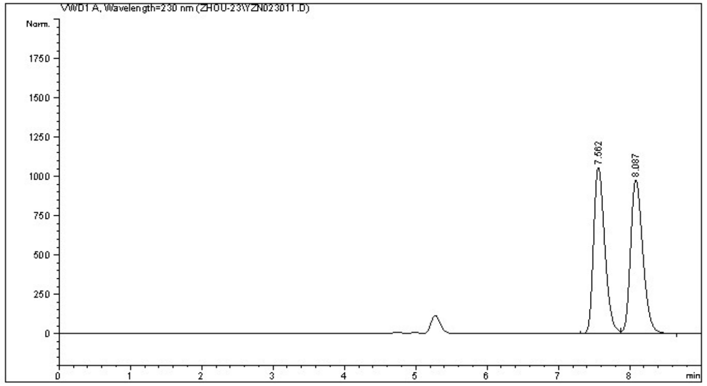

===
Area Percent Report
===

Sorted By : Signal
Multiplier: : 1.0000
Dilution: : 1.0000
Use Multiplier & Dilution Factor with ISTDs

Signal 1: VWD1 A, Wavelength=230 nm

Peak #	RetTime [min]	Type	Width [min]	Area mAU *s	Height [mAU]	Area %
1	7.562	VV	0.1686	1.15752e4	1056.39905	49.6820
2	8.087	VV	0.1863	1.17234e4	978.81940	50.3180

Totals : 2.32986e4 2035.21844

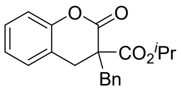

(+/-)-3m

===
*** End of Report ***

Instrument 1 10/17/2023 9:49:26 AM Page 1 of 1

Data File C:\CHEM32\1\DATA\ZHOU-23\YZN023009.D
Sample Name: HW-14-48A SM

===

Acq. Operator :
Acq. Instrument : Instrument 1 Location : -
Injection Date : 5/5/2023 4:23:58 AM
Acq. Method : C:\CHEM32\1\METHODS\DEF_LC.M
Last changed : 5/5/2023 4:23:20 AM
(modified after loading)
Analysis Method : C:\CHEM32\1\METHODS\DEF_LC.M
Last changed : 10/17/2023 9:48:45 AM
(modified after loading)
Sample Info : AS-H, Hexane/i-PrOH = 90/10, 0.8 mL/min, 30 oC, 230nm

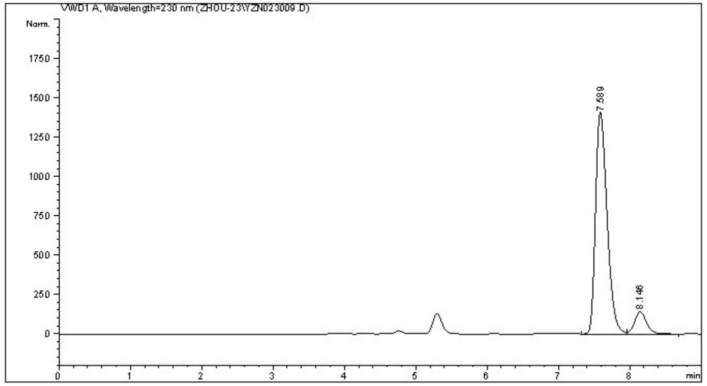

===
Area Percent Report
===

Sorted By : Signal
Multiplier: : 1.0000
Dilution: : 1.0000
Use Multiplier & Dilution Factor with ISTDs

Signal 1: VWD1 A, Wavelength=230 nm

Peak #	RetTime [min]	Type	Width [min]	Area mAU *s	Height [mAU]	Area %
1	7.589	VV	0.1784	1.58565e4	1403.31604	90.3405
2	8.146	VV	0.1865	1695.42773	141.34738	9.6595

Totals : 1.75519e4 1544.66342

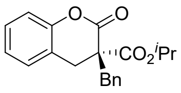

(+)-3m'

===
*** End of Report ***

Instrument 1 10/17/2023 9:48:48 AM Page 1 of 1

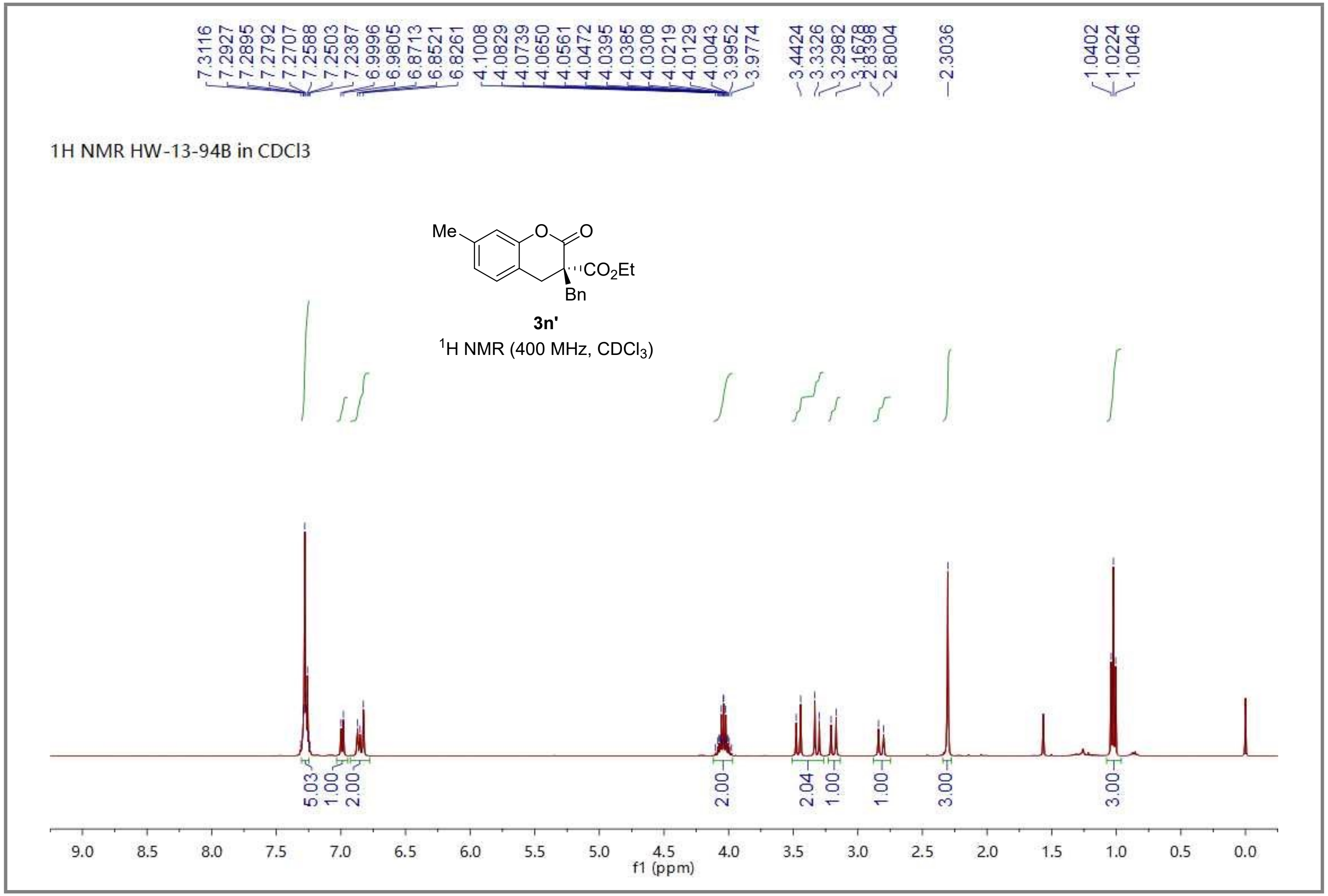

7.3116
7.2927
7.2895
7.2792
7.2707
7.2588
7.2503
7.2387
6.9996
6.9805
6.8713
6.8521
6.8261
4.1008
4.0829
4.0739
4.0650
4.0561
4.0472
4.0395
4.0385
4.0308
4.0219
4.0129
4.0043
3.9952
3.9774
3.4424
3.3326
3.2982
3.1678
2.8398
2.8004
2.3036
1.0402
1.0224
1.0046
1H NMR HW-13-94B in CDCl3
Me
O
O
CO2Et
Bn
3n'
1H NMR (400 MHz, CDCl3)
5.03
1.00
2.00
2.00
2.04
1.00
1.00
3.00
3.00
9.0
8.5
8.0
7.5
7.0
6.5
6.0
5.5
5.0
4.5
4.0
3.5
3.0
2.5
2.0
1.5
1.0
0.5
0.0
f1 (ppm)

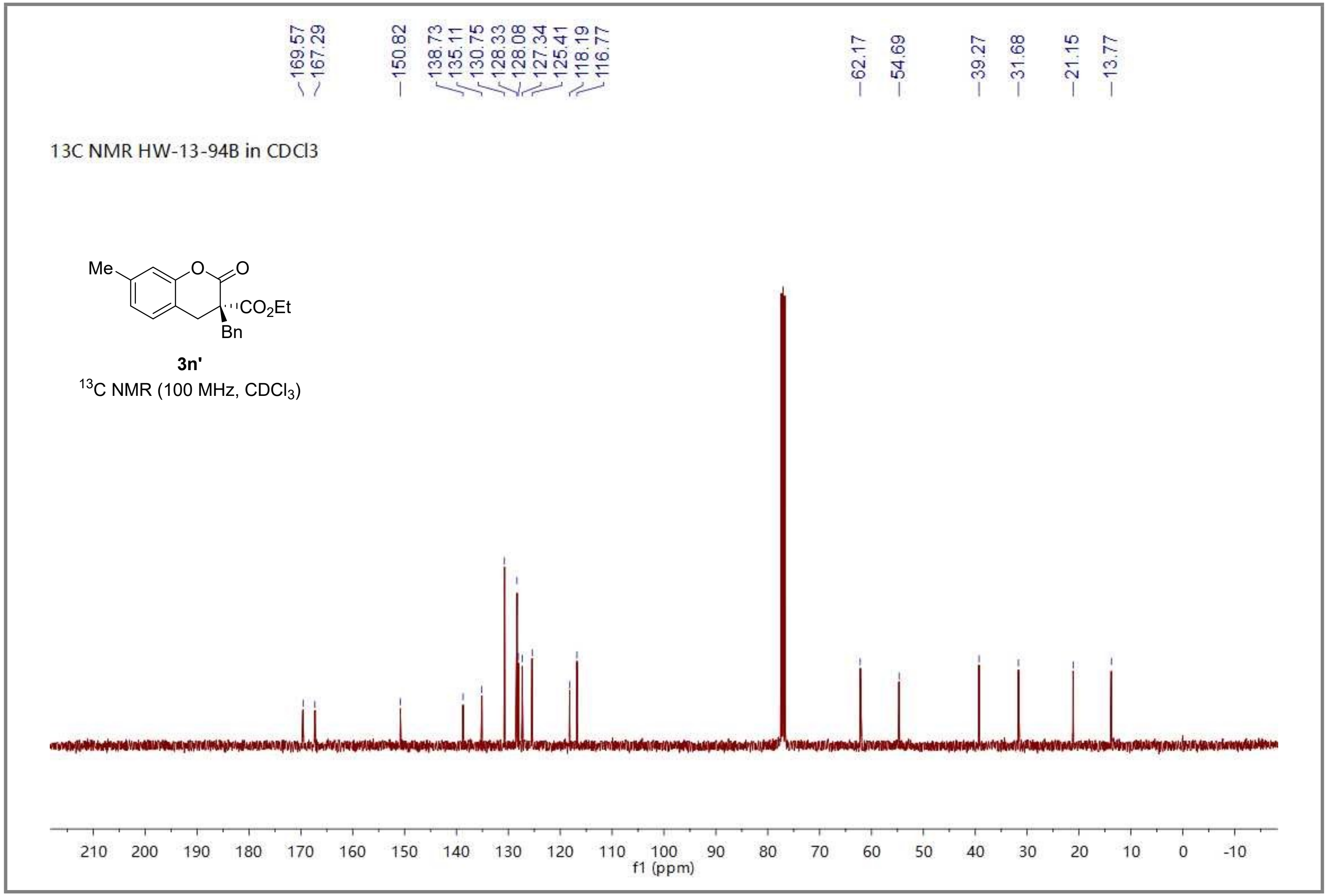
169.57
167.29
150.82
138.73
135.11
130.75
128.33
128.08
127.34
125.41
118.19
116.77
62.17
54.69
39.27
31.68
21.15
13.77
13C NMR HW-13-94B in CDCl3
Me
O
O
CO2Et
Bn
3n'
13C NMR (100 MHz, CDCl3)
210 200 190 180 170 160 150 140 130 120 110 100 90 80 70 60 50 40 30 20 10 0 -10
f1 (ppm)

Data File C:\USERS\PUBLIC\DOCUMENTS\CHEMSTATION\1\DATA\2023\YZH005554 14-43-18.D
Sample Name: HW-13-94B+/-

===

Acq. Operator : SYSTEM
Sample Operator : SYSTEM
Acq. Instrument : HPLC1260 II Location : 1
Injection Date : 11/28/2023 2:43:18 PM Inj : 1
Inj Volume : No inj
Acq. Method : C:\USERS\PUBLIC\DOCUMENTS\CHEMSTATION\1\METHODS\def_LC1.M
Last changed : 11/28/2023 2:29:47 PM by SYSTEM
(modified after loading)
Analysis Method : C:\USERS\PUBLIC\DOCUMENTS\CHEMSTATION\1\METHODS\def_LC1.M
Last changed : 11/29/2023 11:00:00 AM by SYSTEM
(modified after loading)
Sample Info : IB, Hexane/i-PrOH = 97/3, 0.8 mL/min, 30 oC, 230 nm

Additional Info : Peak(s) manually integrated

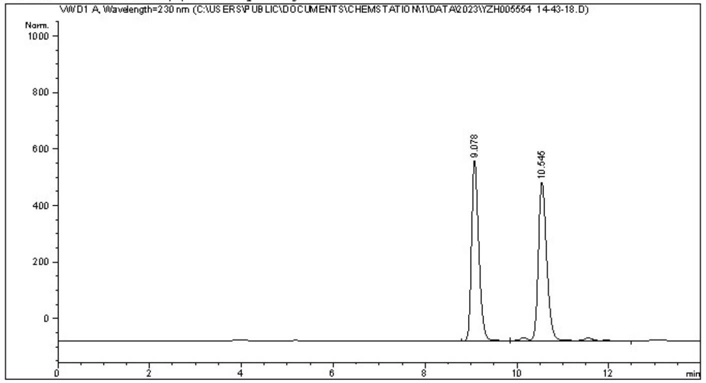

===
Area Percent Report
===

Sorted By : Signal
Multiplier : 1.0000
Dilution : 1.0000
Sample Amount: : 1.00000 [ng/ul] (not used in calc.)
Do not use Multiplier & Dilution Factor with ISTDs

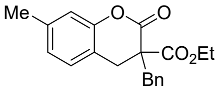

(+/-)-3n

Signal 1: VWD1 A, Wavelength=230 nm

Peak #	RetTime [min]	Type	Width [min]	Area [mAU*s]	Height [mAU]	Area %
1	9.078	BB	0.1677	5274.76465	481.92596	48.8818
2	10.545	VV R	0.1919	5516.08203	423.66296	51.1182

HPLC1260 II 11/29/2023 11:00:04 AM SYSTEM

Page 1 of 2

Data File C:\USERS\PUBLIC\DOCUMENTS\CHEMSTATION\1\DATA\2023\YZH005555 14-59-36.D
Sample Name: HW-14-12B SM'

===

Acq. Operator : SYSTEM
Sample Operator : SYSTEM
Acq. Instrument : HPLC1260 II Location : 1
Injection Date : 11/28/2023 2:59:36 PM Inj : 1
Inj Volume : No inj
Acq. Method : C:\USERS\PUBLIC\DOCUMENTS\CHEMSTATION\1\METHODS\def_LC1.M
Last changed : 11/28/2023 2:29:47 PM by SYSTEM
(modified after loading)
Analysis Method : C:\USERS\PUBLIC\DOCUMENTS\CHEMSTATION\1\METHODS\def_LC1.M
Last changed : 11/29/2023 11:01:10 AM by SYSTEM
(modified after loading)
Sample Info : IB, Hexane/i-PrOH = 97/3, 0.8 mL/min, 30 oC, 230 nm

Additional Info : Peak(s) manually integrated

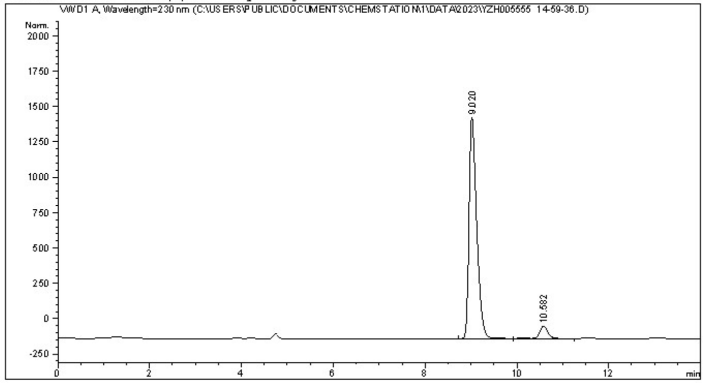

===
Area Percent Report
===

Sorted By : Signal
Multiplier : 1.0000
Dilution : 1.0000
Sample Amount: : 1.00000 [ng/ul] (not used in calc.)
Do not use Multiplier & Dilution Factor with ISTDs

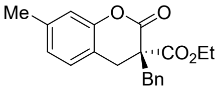

(+)-3n'

Signal 1: VWD1 A, Wavelength=230 nm

Peak #	RetTime [min]	Type	Width [min]	Area [mAU*s]	Height [mAU]	Area %
1	9.020	BB	0.1740	1.48492e4	1282.79663	94.0383
2	10.582	VB R	0.1873	941.38684	72.44398	5.9617

HPLC1260 II 11/29/2023 11:01:17 AM SYSTEM

Page 1 of 2

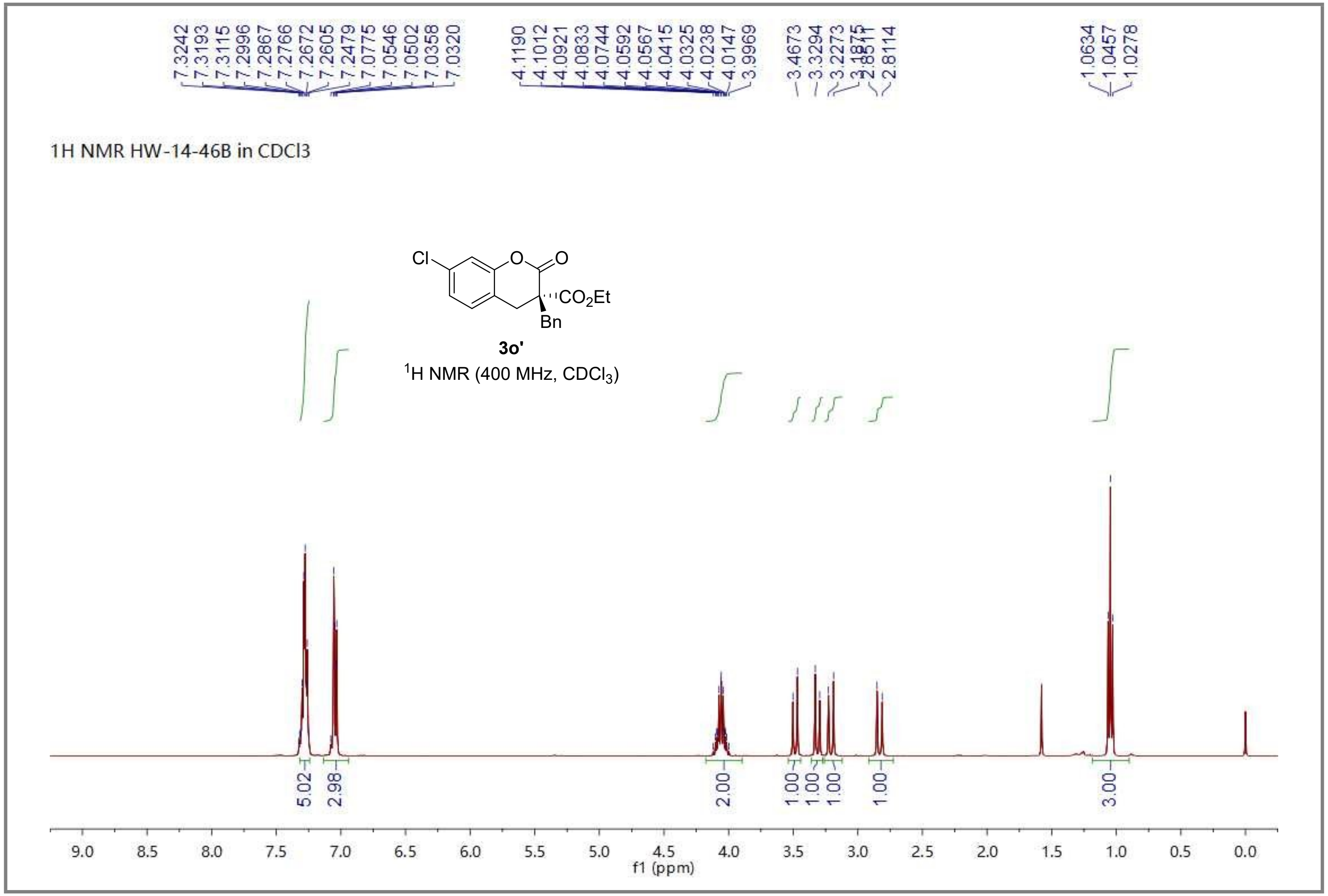

7.3242
7.3193
7.3115
7.2996
7.2867
7.2766
7.2672
7.2605
7.2479
7.0775
7.0546
7.0502
7.0358
7.0320
4.1190
4.1012
4.0921
4.0833
4.0744
4.0592
4.0567
4.0415
4.0325
4.0238
4.0147
3.9969
3.4673
3.3294
3.2273
3.1875
2.8511
2.8114
1.0634
1.0457
1.0278
1H NMR HW-14-46B in CDCl3
Cl
O
O
CO2Et
Bn
3o'
1H NMR (400 MHz, CDCl3)
5.02
2.98
2.00
1.00
1.00
1.00
1.00
3.00
9.0
8.5
8.0
7.5
7.0
6.5
6.0
5.5
5.0
4.5
4.0
3.5
3.0
2.5
2.0
1.5
1.0
0.5
0.0
f1 (ppm)

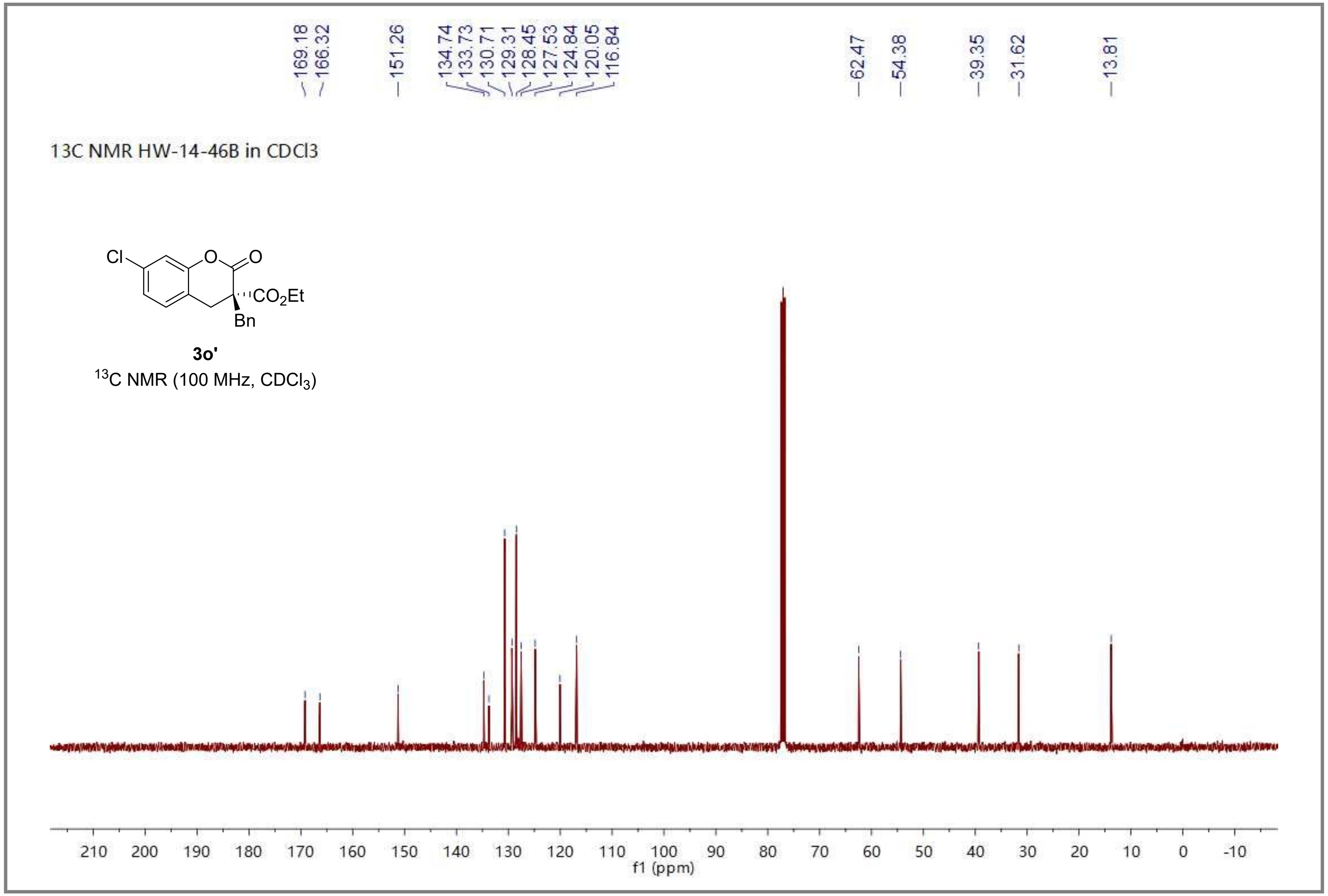
169.18
166.32
151.26
134.74
133.73
130.71
129.31
128.45
127.53
124.84
120.05
116.84
62.47
54.38
39.35
31.62
13.81
13C NMR HW-14-46B in CDCl3
Cl
O
O
CO2Et
Bn
3o'
13C NMR (100 MHz, CDCl3)
210 200 190 180 170 160 150 140 130 120 110 100 90 80 70 60 50 40 30 20 10 0 -10
f1 (ppm)

Data File C:\USERS\PUBLIC\DOCUMENTS\CHEMSTATION\1\DATA\2023\YZH004521 16-19-57.D
Sample Name: HW-14-92SM +/-

===

Acq. Operator : SYSTEM
Sample Operator : SYSTEM
Acq. Instrument : HPLC1260 II Location : 1
Injection Date : 7/10/2023 4:19:57 PM Inj : 1
Inj Volume : No inj
Acq. Method : C:\USERS\PUBLIC\DOCUMENTS\CHEMSTATION\1\METHODS\def_LC1.M
Last changed : 7/10/2023 4:05:56 PM by SYSTEM
(modified after loading)
Analysis Method : C:\USERS\PUBLIC\DOCUMENTS\CHEMSTATION\1\METHODS\def_LC1.M
Last changed : 10/16/2023 10:14:17 PM by SYSTEM
(modified after loading)
Sample Info : AS-H, Hexane/i-PrOH = 93/7, 0.8 mL/min, 30 oC, 230 nm

Additional Info : Peak(s) manually integrated

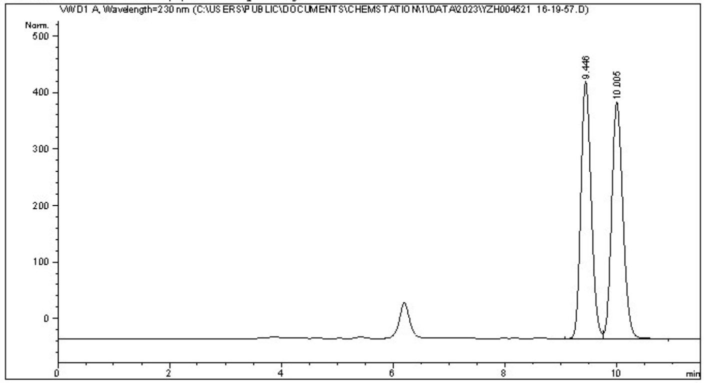

===

Area Percent Report

===

Sorted By : Signal
Multiplier : 1.0000
Dilution : 1.0000
Sample Amount: : 1.00000 [ng/ul] (not used in calc.)
Do not use Multiplier & Dilution Factor with ISTDs

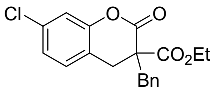

(+/-)-3o

Signal 1: VWD1 A, Wavelength=230 nm

Peak #	RetTime [min]	Type	Width [min]	Area [mAU*s]	Height [mAU]	Area %
1	9.446	BV	0.1987	4970.99463	390.02411	49.7473
2	10.005	VB	0.2174	5021.50635	358.63422	50.2527

HPLC1260 II 10/16/2023 10:14:21 PM SYSTEM Page 1 of 2

Data File C:\USERS\PUBLIC\DOCUMENTS\CHEMSTATION\1\DATA\2023\YZH004522 16-41-25.D
Sample Name: HW-14-92SM

===

Acq. Operator : SYSTEM
Sample Operator : SYSTEM
Acq. Instrument : HPLC1260 II Location : 1
Injection Date : 7/10/2023 4:41:25 PM Inj : 1
Inj Volume : No inj
Acq. Method : C:\USERS\PUBLIC\DOCUMENTS\CHEMSTATION\1\METHODS\def_LC1.M
Last changed : 7/10/2023 4:05:56 PM by SYSTEM
(modified after loading)
Analysis Method : C:\USERS\PUBLIC\DOCUMENTS\CHEMSTATION\1\METHODS\def_LC1.M
Last changed : 10/16/2023 10:15:20 PM by SYSTEM
(modified after loading)
Sample Info : AS-H, Hexane/i-PrOH = 93/7, 0.8 mL/min, 30 oC, 230 nm

Additional Info : Peak(s) manually integrated

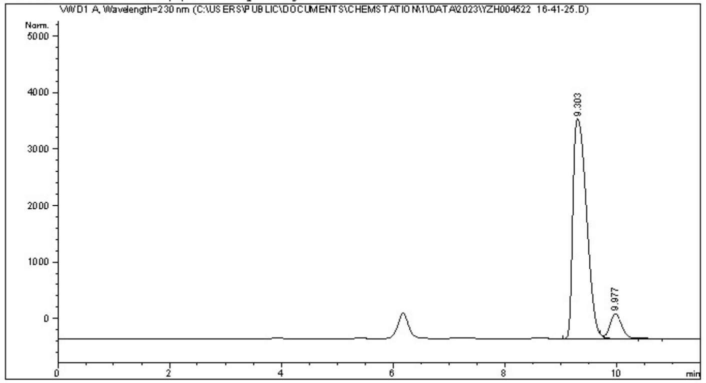

===

Area Percent Report

===

Sorted By : Signal
Multiplier : 1.0000
Dilution : 1.0000
Sample Amount: : 1.00000 [ng/ul] (not used in calc.)
Do not use Multiplier & Dilution Factor with ISTDs

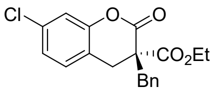

(+)-3o'

Signal 1: VWD1 A, Wavelength=230 nm

Peak #	RetTime [min]	Type	Width [min]	Area [mAU*s]	Height [mAU]	Area %
1	9.303	BV R	0.2700	5.37358e4	3178.20581	91.0710
2	9.977	VV E	0.2255	5268.52686	362.52792	8.9290

HPLC1260 II 10/16/2023 10:15:24 PM SYSTEM Page 1 of 2

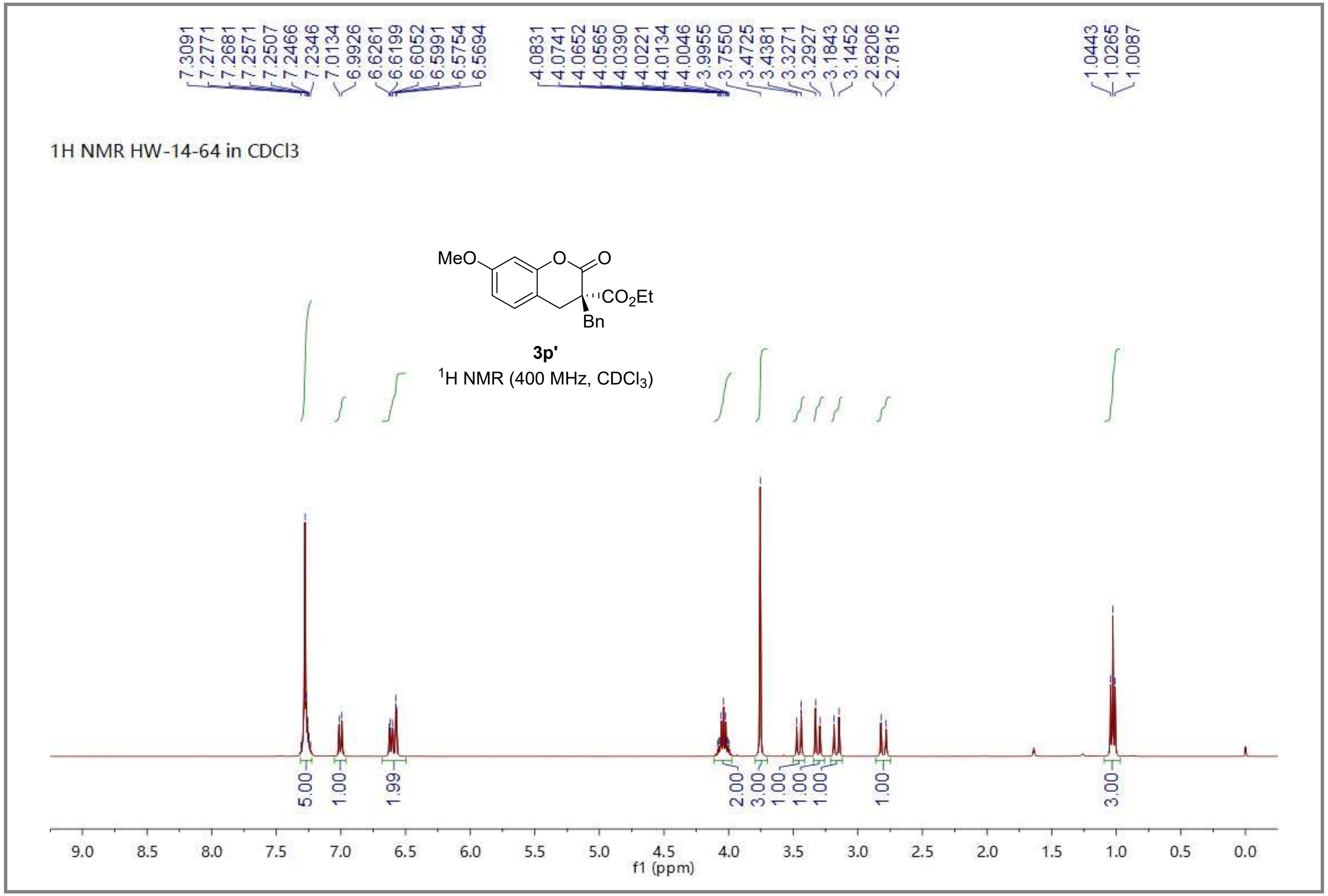

7.3091
7.2771
7.2681
7.2571
7.2507
7.2466
7.2346
7.0134
6.9926
6.6261
6.6199
6.6052
6.5991
6.5754
6.5694
4.0831
4.0741
4.0652
4.0565
4.0390
4.0221
4.0134
4.0046
3.9955
3.7550
3.4725
3.4381
3.3271
3.2927
3.1843
3.1452
2.8206
2.7815
1.0443
1.0265
1.0087
1H NMR HW-14-64 in CDCl3
MeO
O
O
CO2Et
Bn
3p'
1H NMR (400 MHz, CDCl3)
5.00
1.00
1.99
2.00
3.00
1.00
1.00
1.00
1.00
3.00
9.0 8.5 8.0 7.5 7.0 6.5 6.0 5.5 5.0 4.5 4.0 3.5 3.0 2.5 2.0 1.5 1.0 0.5 0.0
f1 (ppm)

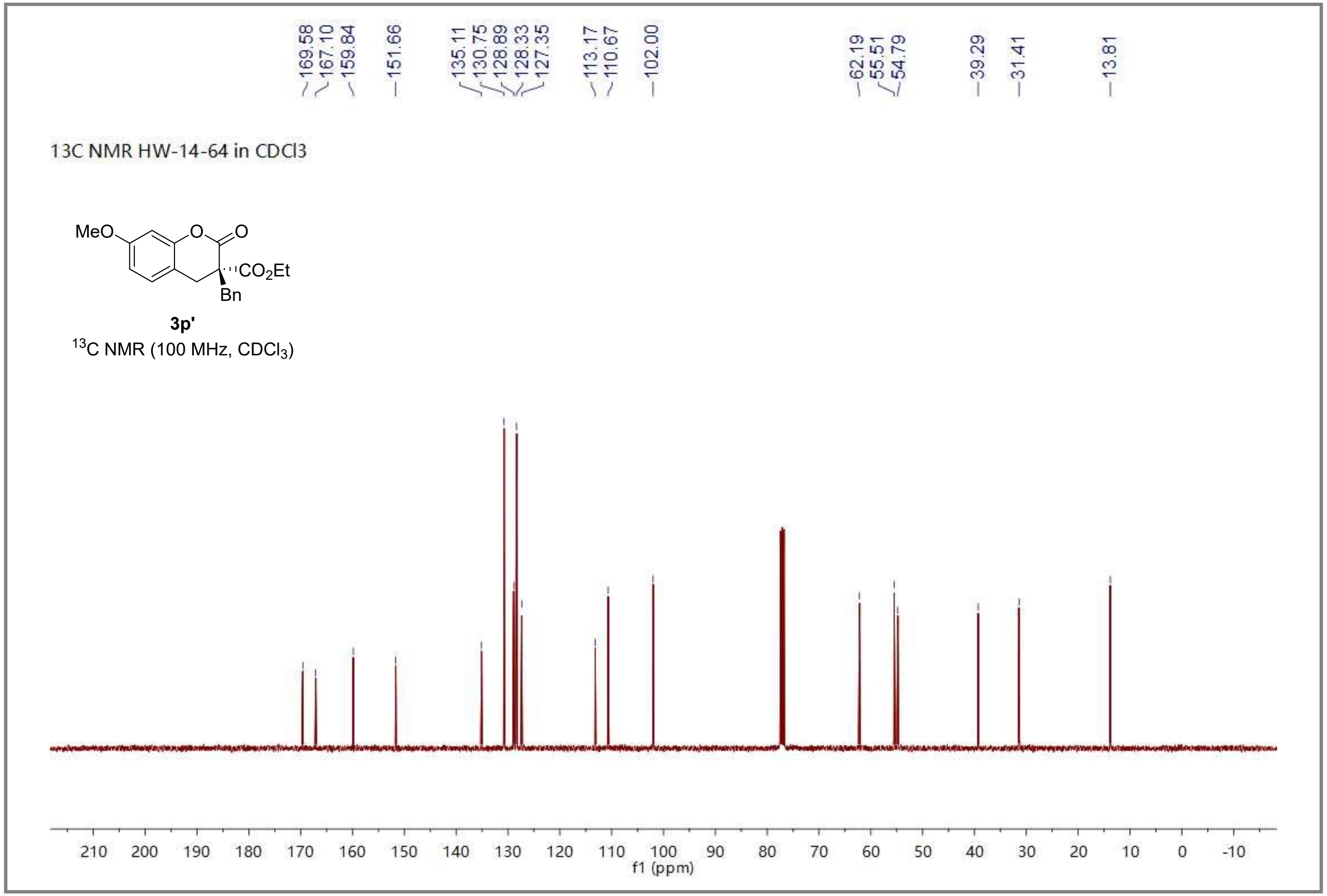

169.58
167.10
159.84
151.66
135.11
130.75
128.89
128.33
127.35
113.17
110.67
102.00
62.19
55.51
54.79
39.29
31.41
13.81
13C NMR HW-14-64 in CDCl3
MeO
O
O
CO2Et
Bn
3p'
13C NMR (100 MHz, CDCl3)
210
200
190
180
170
160
150
140
130
120
110
100
90
80
70
60
50
40
30
20
10
0
-10
f1 (ppm)

Data File C:\CHEM32\1\DATA\ZHOU-23\YZN023216.D
Sample Name: HW-14-73A SM+/-

===

Acq. Operator :
Acq. Instrument : Instrument 1 Location : -
Injection Date : 5/30/2023 8:44:18 AM
Acq. Method : C:\CHEM32\1\METHODS\DEF_LC.M
Last changed : 5/30/2023 8:35:12 AM
(modified after loading)
Analysis Method : C:\CHEM32\1\METHODS\DEF_LC.M
Last changed : 10/17/2023 10:08:16 AM
(modified after loading)
Sample Info : od-H, Hexane/i-PrOH = 90/10, 0.8 mL/min, 30 oC, 230 nm

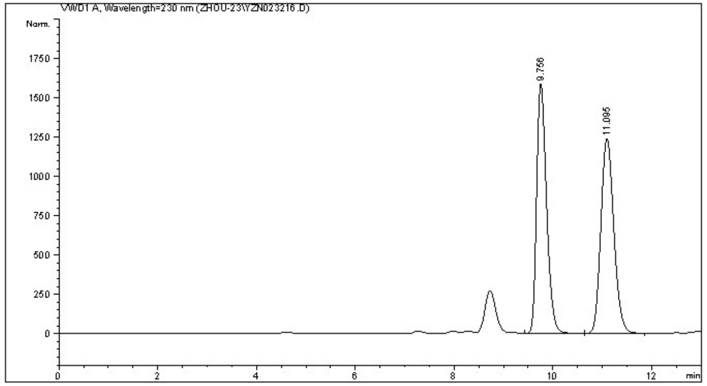

===
Area Percent Report
===

Sorted By : Signal
Multiplier: : 1.0000
Dilution: : 1.0000
Use Multiplier & Dilution Factor with ISTDs

Signal 1: VWD1 A, Wavelength=230 nm

Peak #	RetTime [min]	Type	Width [min]	Area mAU *s	Height [mAU]	Area %
1	9.756	VV	0.2160	2.22832e4	1589.47205	49.4825
2	11.095	VV	0.2899	2.27492e4	1238.88599	50.5175

Totals : 4.50324e4 2828.35803

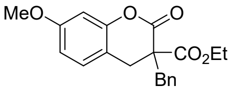

(+/-)-3p

===
*** End of Report ***

Instrument 1 10/17/2023 10:08:19 AM Page 1 of 1

Data File C:\CHEM32\1\DATA\ZHOU-23\YZN023217.D
Sample Name: HW-14-73A SM

===

Acq. Operator :
Acq. Instrument : Instrument 1 Location : -
Injection Date : 5/30/2023 9:17:42 AM
Acq. Method : C:\CHEM32\1\METHODS\DEF_LC.M
Last changed : 5/30/2023 9:17:10 AM
(modified after loading)
Analysis Method : C:\CHEM32\1\METHODS\DEF_LC.M
Last changed : 10/17/2023 10:09:09 AM
(modified after loading)
Sample Info : OD-H, Hexane/i-PrOH = 90/10, 0.8 mL/min, 30 oC, 230 nm

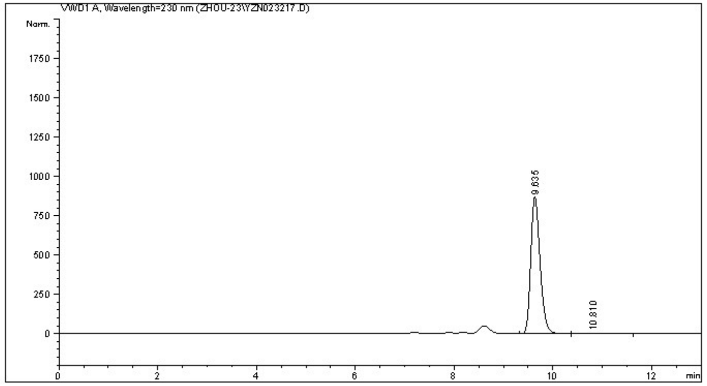

===
Area Percent Report
===

Sorted By : Signal
Multiplier: : 1.0000
Dilution: : 1.0000
Use Multiplier & Dilution Factor with ISTDs

Signal 1: VWD1 A, Wavelength=230 nm

Peak #	RetTime [min]	Type	Width [min]	Area mAU *s	Height [mAU]	Area %
1	9.635	VV	0.2048	1.13250e4	867.21967	98.9115
2	10.810	VV	0.4614	124.63101	4.14512	1.0885

Totals : 1.14497e4 871.36478

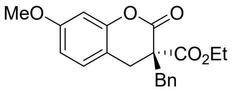

(+)-3p'

===
*** End of Report ***

Instrument 1 10/17/2023 10:09:12 AM Page 1 of 1

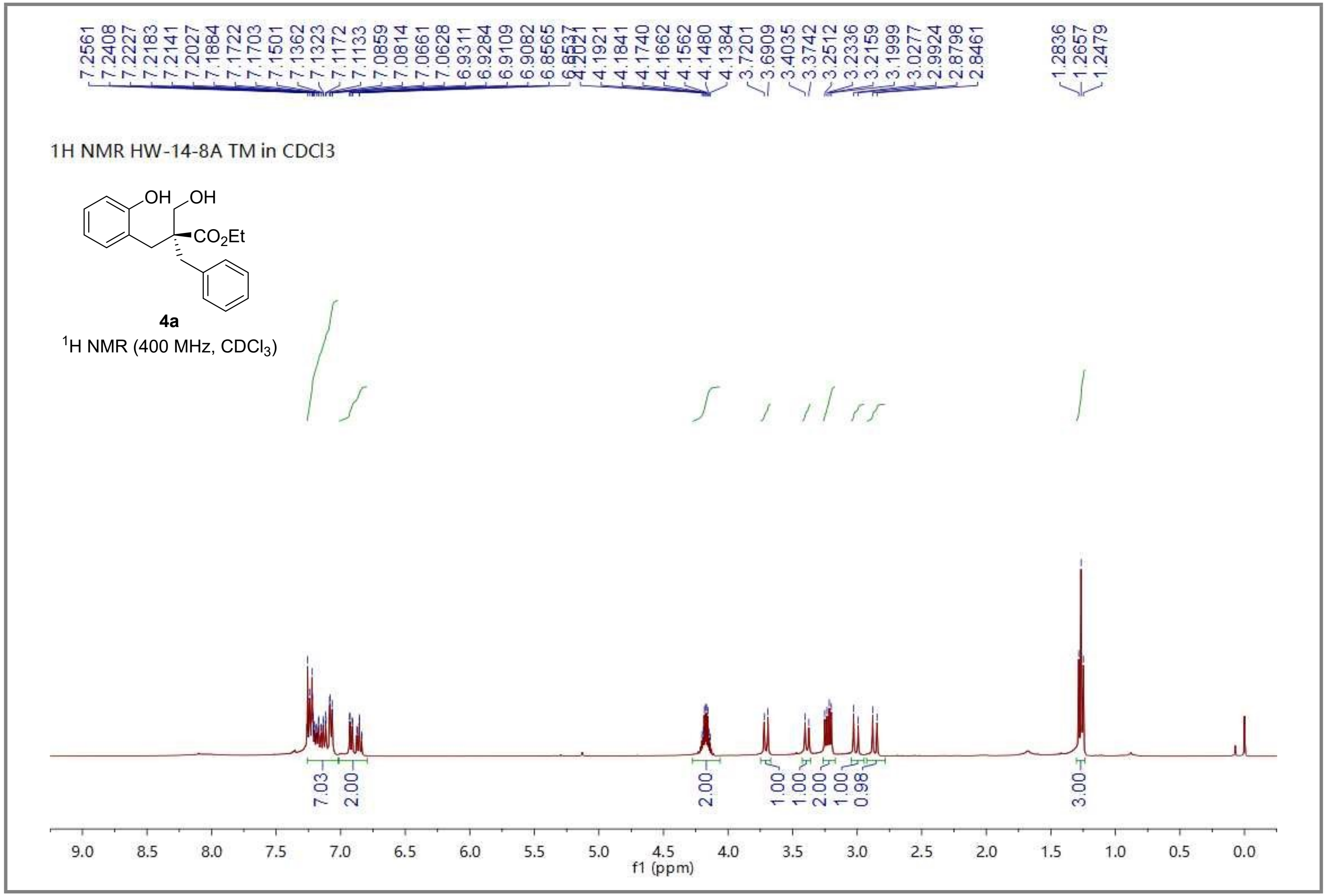
1H NMR HW-14-8A TM in CDCl3
OH
OH
CO2Et
4a
1H NMR (400 MHz, CDCl3)
7.03
2.00
2.00
1.00
1.00
2.00
1.00
0.98
3.00
f1 (ppm)

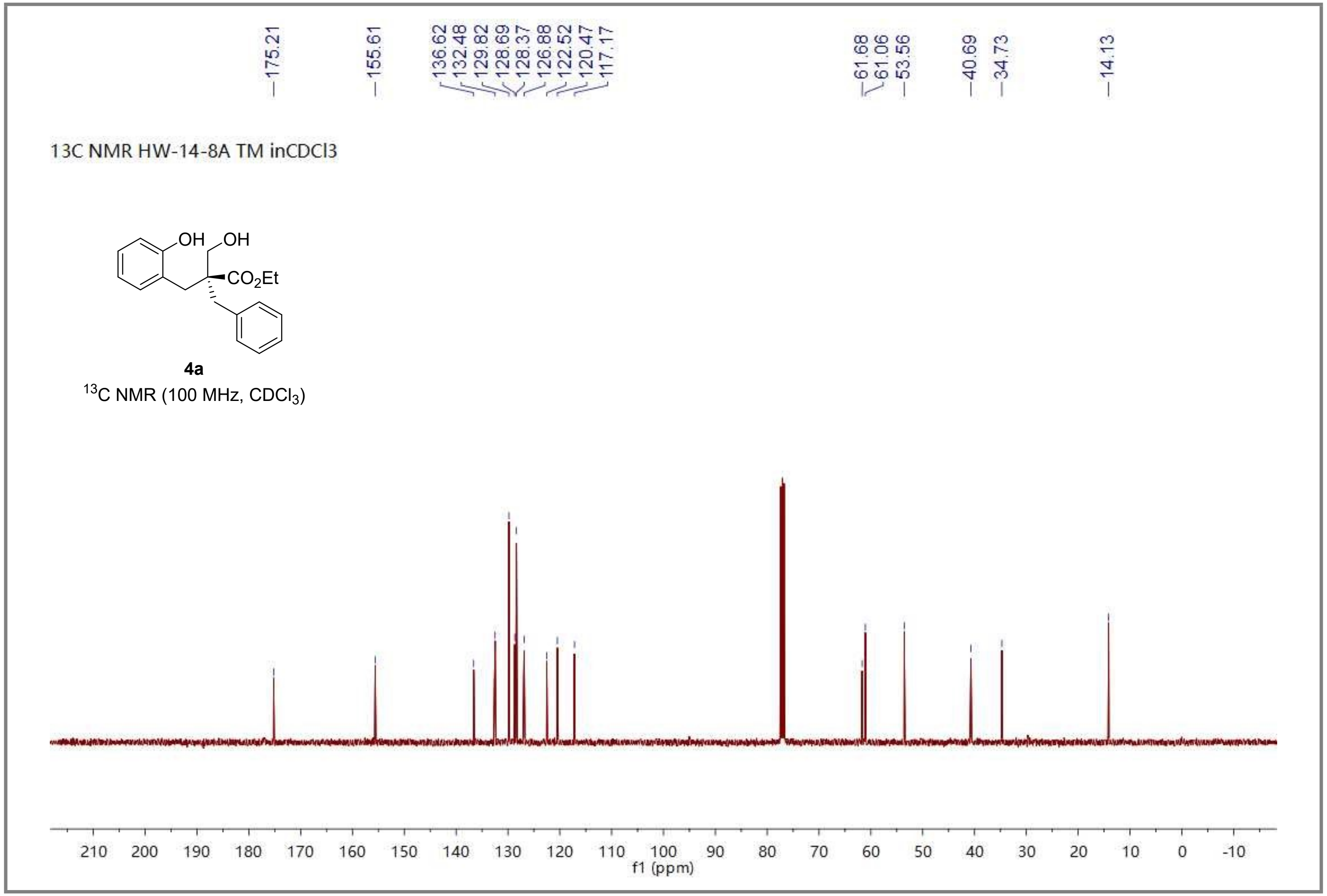
175.21
155.61
136.62
132.48
129.82
128.69
128.37
126.88
122.52
120.47
117.17
61.68
61.06
53.56
40.69
34.73
14.13
13C NMR HW-14-8A TM inCDCl3
OH
OH
CO2Et
4a
13C NMR (100 MHz, CDCl3)
210 200 190 180 170 160 150 140 130 120 110 100 90 80 70 60 50 40 30 20 10 0 -10
f1 (ppm)

Data File C:\CHEM32\1\DATA\ZHOU-22\YZN022298.D
Sample Name: HW-12-84+/-

===

Acq. Operator :
Acq. Instrument : Instrument 1 Location : -
Injection Date : 12/30/2022 10:22:40 PM
Acq. Method : C:\CHEM32\1\METHODS\DEF_LC.M
Last changed : 12/30/2022 10:22:16 PM
(modified after loading)
Analysis Method : C:\CHEM32\1\METHODS\DEF_LC.M
Last changed : 10/17/2023 8:35:45 AM
(modified after loading)
Sample Info : IB, Hexane/i-PrOH = 95/5, 0.8 mL/min, 30 oC, 230 nm

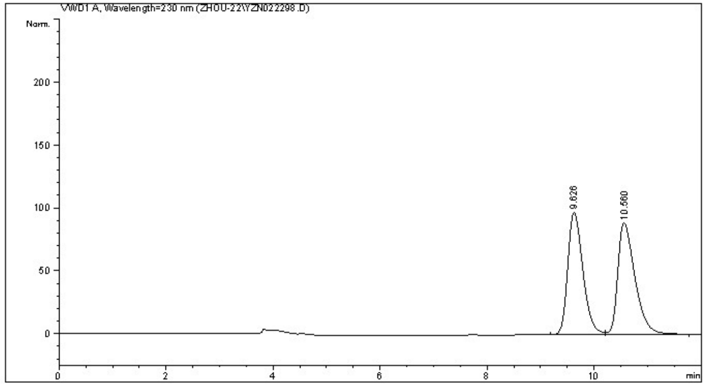

===
Area Percent Report
===

Sorted By : Signal
Multiplier: : 1.0000
Dilution: : 1.0000
Use Multiplier & Dilution Factor with ISTDs

Signal 1: VWD1 A, Wavelength=230 nm

Peak #	RetTime [min]	Type	Width [min]	Area mAU *s	Height [mAU]	Area %
1	9.626	BV	0.3044	1919.77893	97.30987	49.1682
2	10.560	VB	0.3395	1984.73413	88.77587	50.8318

Totals : 3904.51306 186.08574

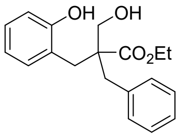

(+/-)-4a

===
*** End of Report ***

Instrument 1 10/17/2023 8:35:50 AM Page 1 of 1

Data File C:\USERS\PUBLIC\DOCUMENTS\CHEMSTATION\1\DATA\2023\YZH003727 20-08-27.D
Sample Name: HW-14-8A TM

===

Acq. Operator : SYSTEM
Sample Operator : SYSTEM
Acq. Instrument : HPLC1260 II Location : 1
Injection Date : 3/22/2023 8:08:27 PM Inj : 1
Inj Volume : No inj
Acq. Method : C:\USERS\PUBLIC\DOCUMENTS\CHEMSTATION\1\METHODS\def_LC1.M
Last changed : 3/22/2023 7:43:25 PM by SYSTEM
(modified after loading)
Analysis Method : C:\USERS\PUBLIC\DOCUMENTS\CHEMSTATION\1\METHODS\def_LC1.M
Last changed : 10/16/2023 8:57:56 PM by SYSTEM
(modified after loading)
Sample Info : IB, Hexane/i-PrOH = 95/5, 0.8 mL/min, 30 oC, 230 nm

Additional Info : Peak(s) manually integrated

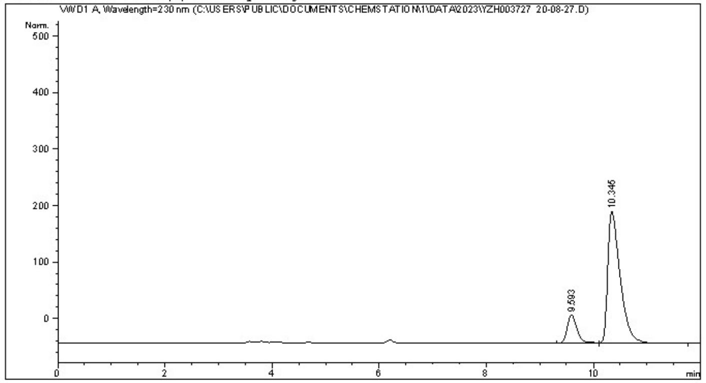

===
Area Percent Report
===

Sorted By : Signal
Multiplier : 1.0000
Dilution : 1.0000
Sample Amount: : 1.00000 [ng/ul] (not used in calc.)
Do not use Multiplier & Dilution Factor with ISTDs

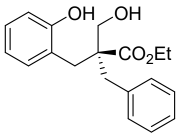

(+)-4a

Signal 1: VWD1 A, Wavelength=230 nm

Peak #	RetTime [min]	Type	Width [min]	Area [mAU*s]	Height [mAU]	Area %
1	9.593	BV	0.1944	404.16336	31.55659	14.6134
2	10.345	VB	0.2394	2361.54614	146.99408	85.3866

HPLC1260 II 10/16/2023 8:58:00 PM SYSTEM Page 1 of 2

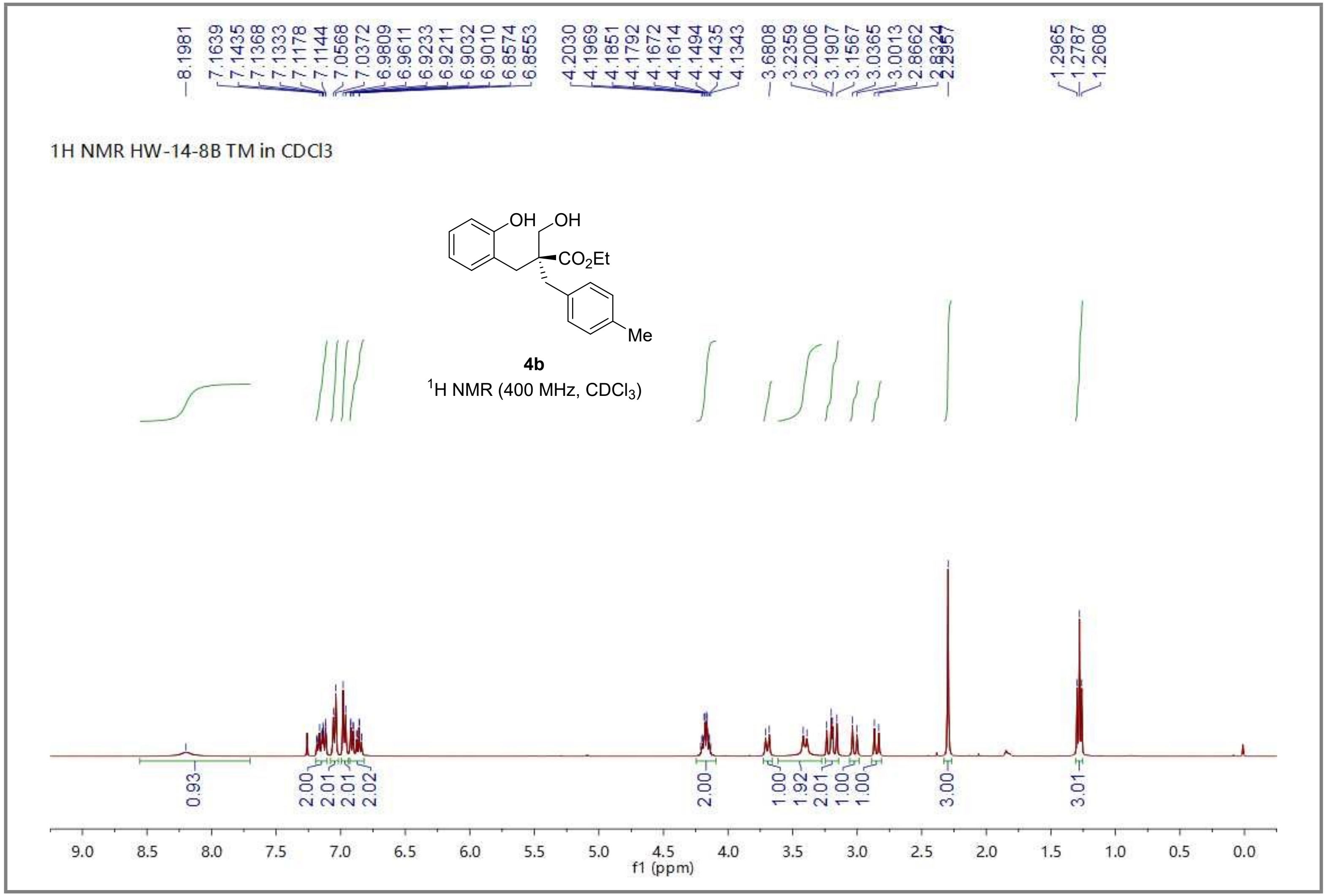
1H NMR HW-14-8B TM in CDCl3
OH
OH
CO2Et
Me
4b
1H NMR (400 MHz, CDCl3)
f1 (ppm)

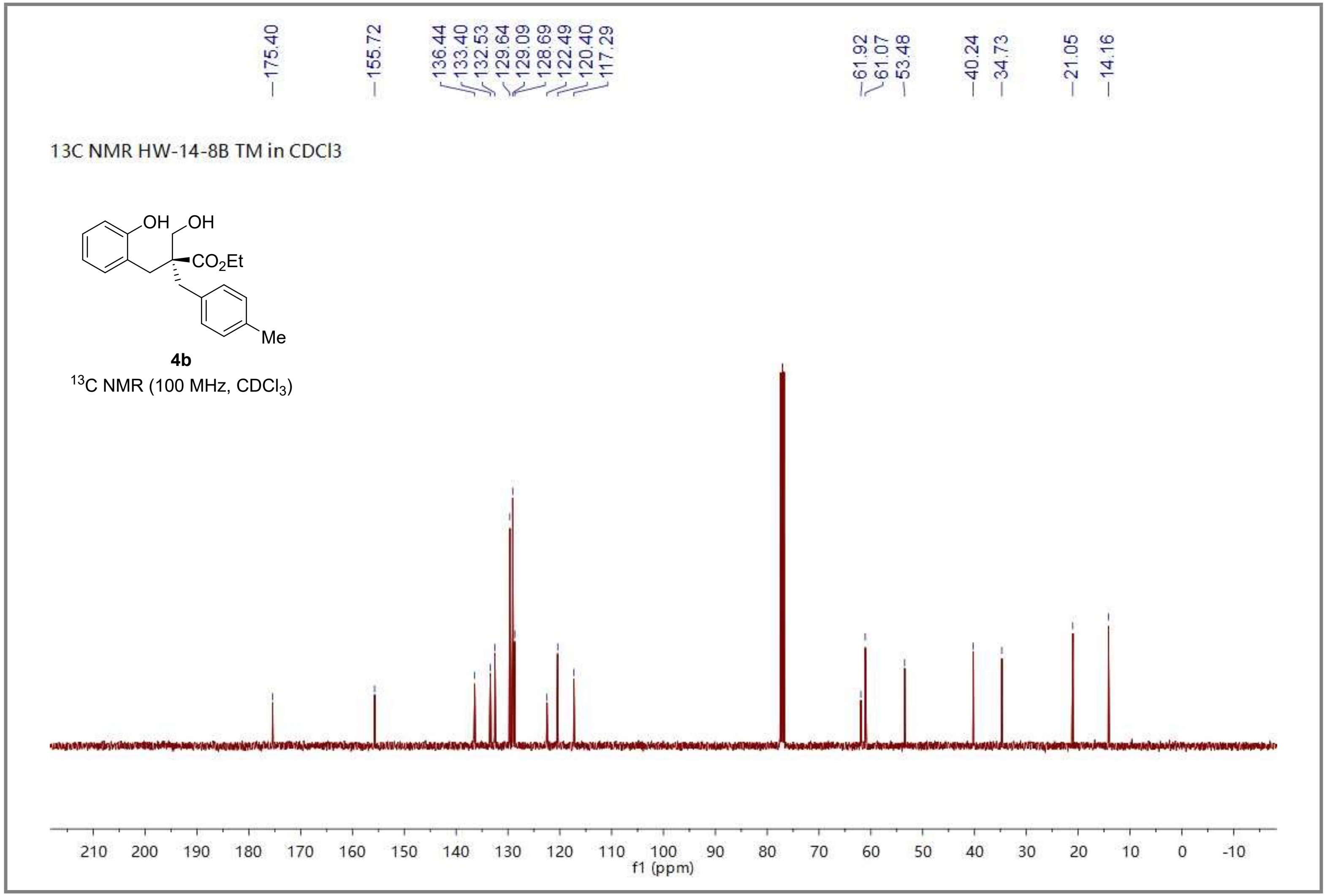

175.40
155.72
136.44
133.40
132.53
129.64
129.09
128.69
122.49
120.40
117.29
61.92
61.07
53.48
40.24
34.73
21.05
14.16
13C NMR HW-14-8B TM in CDCl3
OH
OH
CO2Et
Me
4b
13C NMR (100 MHz, CDCl3)
210
200
190
180
170
160
150
140
130
120
110
100
90
80
70
60
50
40
30
20
10
0
-10
f1 (ppm)

```
Data File C:\USERS\PUBLIC\DOCUMENTS\CHEMSTATION\1\DATA\2023\YZH003731  21-31-16.D
Sample Name: HW-14-8C TM+/-

    =====================================================================
    Acq. Operator   : SYSTEM
    Sample Operator : SYSTEM
    Acq. Instrument : HPLC1260 II                    Location :   1
    Injection Date  : 3/22/2023 9:31:16 PM                 Inj :   1
                                                Inj Volume : No inj
    Acq. Method     : C:\USERS\PUBLIC\DOCUMENTS\CHEMSTATION\1\METHODS\def_LC1.M
    Last changed    : 3/22/2023 7:43:25 PM by SYSTEM
                      (modified after loading)
    Analysis Method : C:\USERS\PUBLIC\DOCUMENTS\CHEMSTATION\1\METHODS\def_LC1.M
    Last changed    : 10/16/2023 9:05:43 PM by SYSTEM
                      (modified after loading)
    Sample Info     : IB, Hexane/i-PrOH = 95/5, 0.8 mL/min, 30 oC, 230 nm

    Additional Info : Peak(s) manually integrated
```

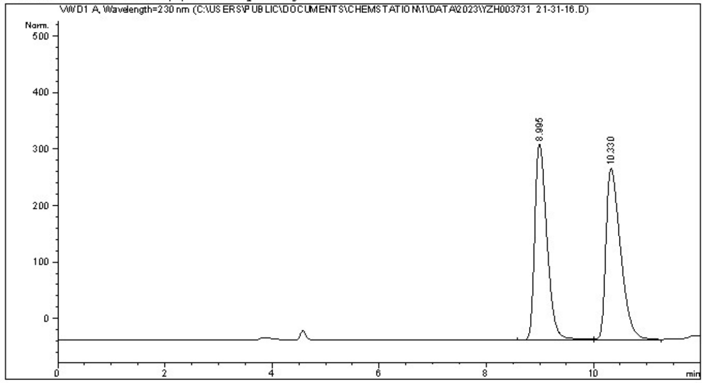


```
    =====================================================================
                              Area Percent Report
    =====================================================================

    Sorted By             :      Signal
    Multiplier            :      1.0000
    Dilution              :      1.0000
    Sample Amount:        :      1.00000  [ng/ul]   (not used in calc.)
    Do not use Multiplier & Dilution Factor with ISTDs


    Signal 1: VWD1 A, Wavelength=230 nm
```

Peak #	RetTime [min]	Type	Width [min]	Area [mAU*s]	Height [mAU]	Area %
1	8.995	BV R	0.2435	4275.72021	271.88998	48.8896
2	10.330	VB	0.2896	4469.95020	237.51076	51.1104

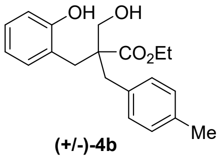

(+/-)-4b

HPLC1260 II 10/16/2023 9:05:48 PM SYSTEM

Page 1 of 2

```
Data File C:\USERS\PUBLIC\DOCUMENTS\CHEMSTATION\1\DATA\2023\YZH003732  21-59-01.D
Sample Name: HW-14-8C TM

    =====================================================================
    Acq. Operator   : SYSTEM
    Sample Operator : SYSTEM
    Acq. Instrument : HPLC1260 II                    Location :   1
    Injection Date  : 3/22/2023 9:59:02 PM                 Inj :   1
                                                Inj Volume : No inj
    Acq. Method     : C:\USERS\PUBLIC\DOCUMENTS\CHEMSTATION\1\METHODS\def_LC1.M
    Last changed    : 3/22/2023 7:43:25 PM by SYSTEM
                      (modified after loading)
    Analysis Method : C:\USERS\PUBLIC\DOCUMENTS\CHEMSTATION\1\METHODS\def_LC1.M
    Last changed    : 10/16/2023 9:04:43 PM by SYSTEM
                      (modified after loading)
    Sample Info     : IB, Hexane/i-PrOH = 95/5, 0.8 mL/min, 30 oC, 230 nm

    Additional Info : Peak(s) manually integrated
```

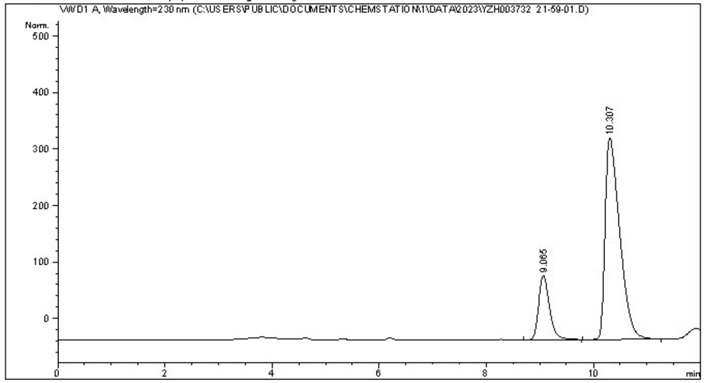


```
    =====================================================================
                              Area Percent Report
    =====================================================================

    Sorted By             :      Signal
    Multiplier            :      1.0000
    Dilution              :      1.0000
    Sample Amount:        :      1.00000  [ng/ul]   (not used in calc.)
    Do not use Multiplier & Dilution Factor with ISTDs


    Signal 1: VWD1 A, Wavelength=230 nm
```

Peak #	RetTime [min]	Type	Width [min]	Area [mAU*s]	Height [mAU]	Area %
1	9.065	BB	0.2145	1265.45081	90.30990	19.1985
2	10.307	BB	0.2875	5325.93701	283.06808	80.8015

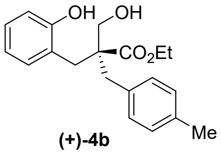

(+)-4b

HPLC1260 II 10/16/2023 9:04:46 PM SYSTEM

Page 1 of 2

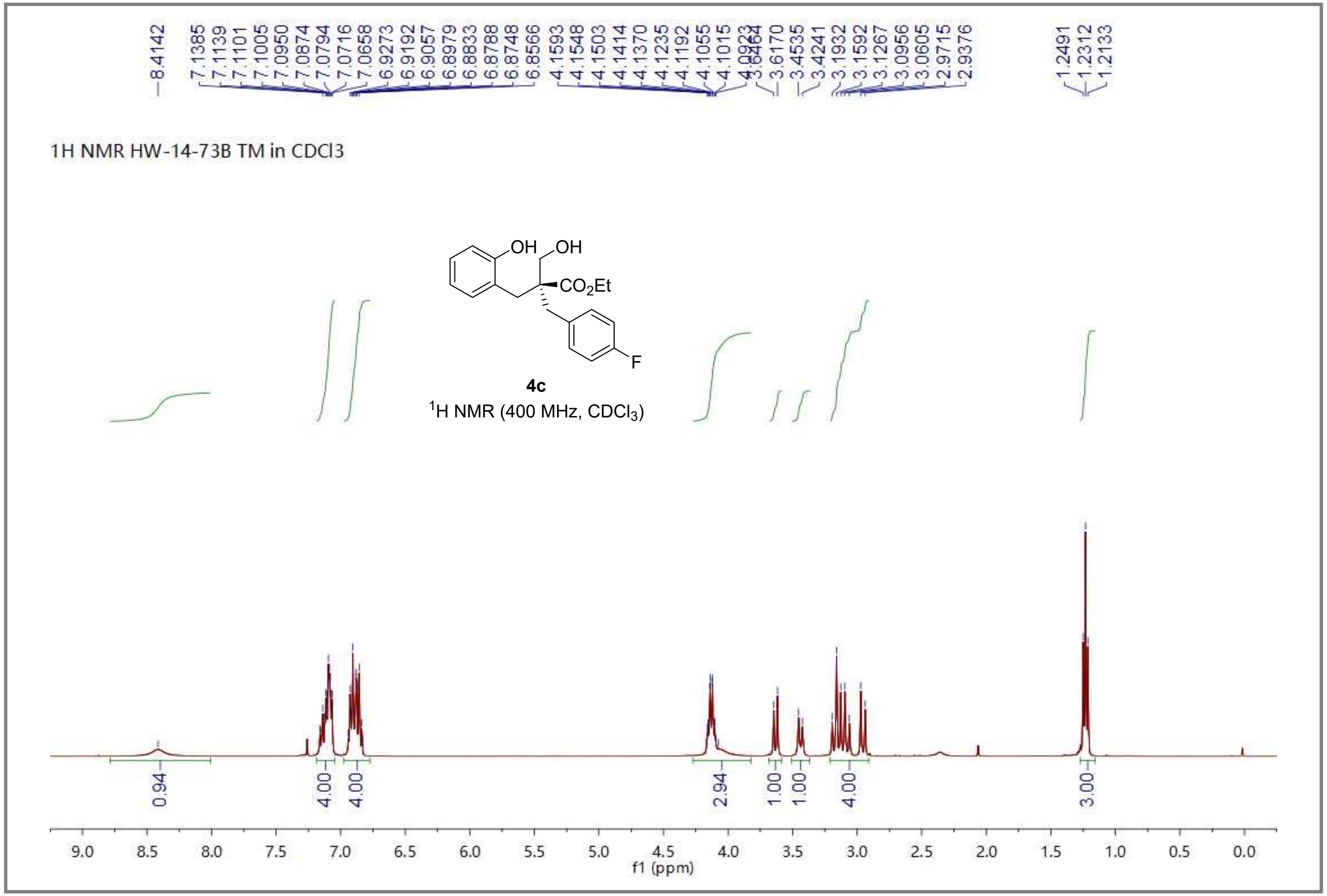
1H NMR HW-14-73B TM in CDCl3
OH
OH
CO2Et
F
4c
1H NMR (400 MHz, CDCl3)
f1 (ppm)

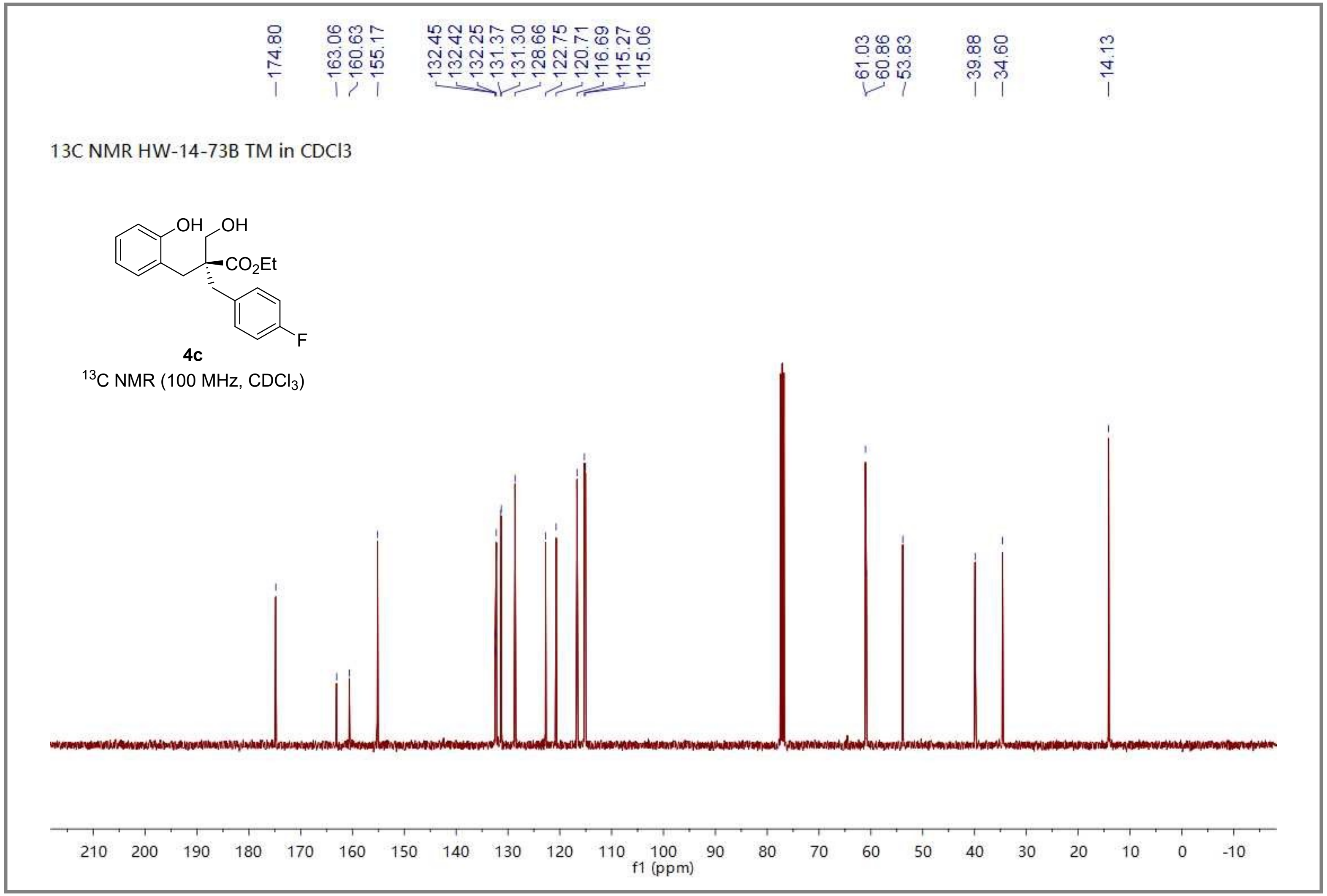

174.80
163.06
160.63
155.17
132.45
132.42
132.25
131.37
131.30
128.66
122.75
120.71
116.69
115.27
115.06
61.03
60.86
53.83
39.88
34.60
14.13
13C NMR HW-14-73B TM in CDCl3
OH
OH
CO2Et
F
4c
13C NMR (100 MHz, CDCl3)
210 200 190 180 170 160 150 140 130 120 110 100 90 80 70 60 50 40 30 20 10 0 -10
f1 (ppm)

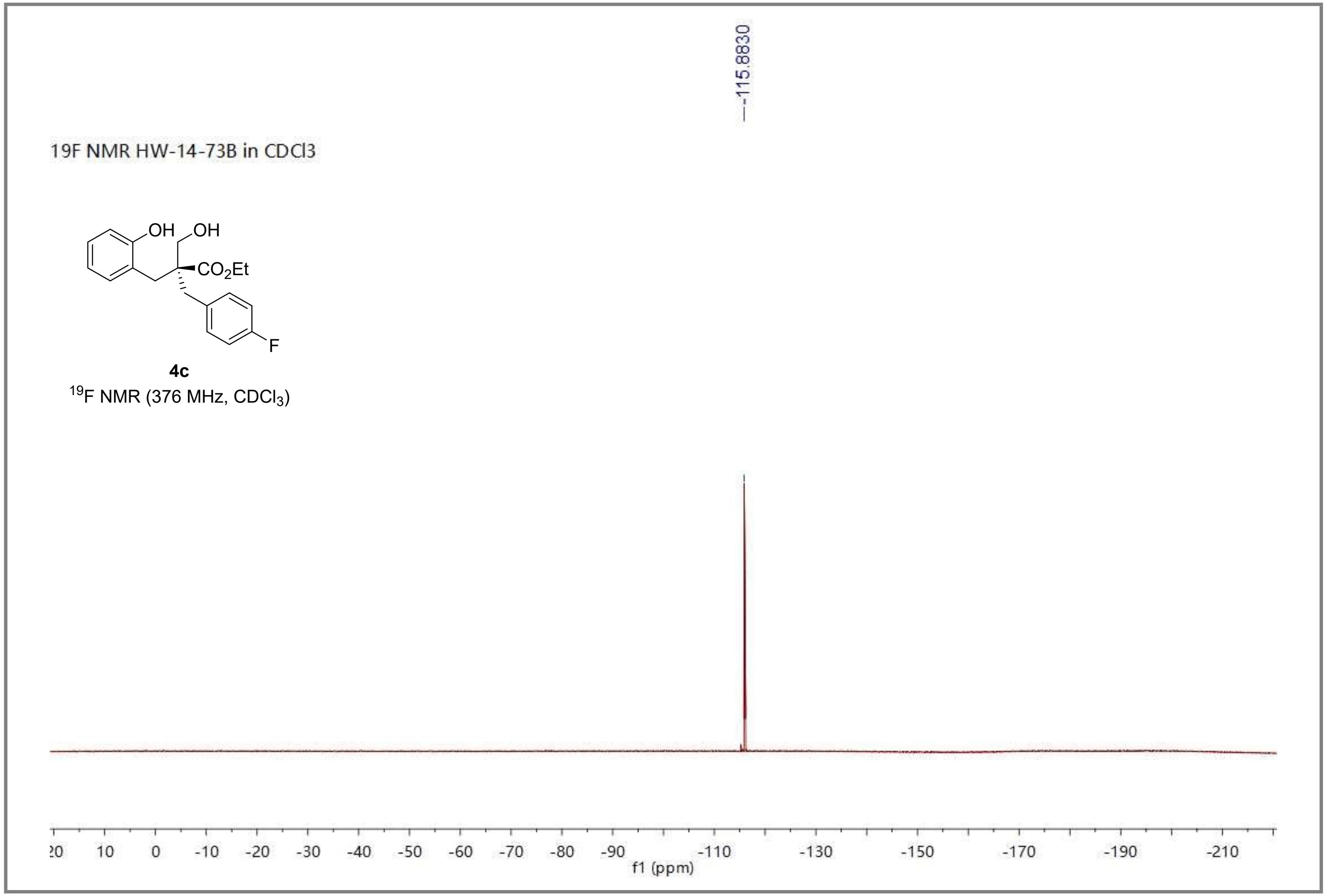

--115.8830
19F NMR HW-14-73B in CDCl3
OH
OH
CO2Et
F
4c
19F NMR (376 MHz, CDCl3)
20
10
0
-10
-20
-30
-40
-50
-60
-70
-80
-90
-110
-130
-150
-170
-190
-210
f1 (ppm)

Data File C:\CHEM32\1\DATA\ZHOU-23\YZN023231.D
Sample Name: HW-14-73CTM+/-

===

Acq. Operator :
Acq. Instrument : Instrument 1 Location : -
Injection Date : 5/31/2023 5:07:35 AM
Acq. Method : C:\CHEM32\1\METHODS\DEF_LC.M
Last changed : 5/31/2023 5:07:01 AM
(modified after loading)
Analysis Method : C:\CHEM32\1\METHODS\DEF_LC.M
Last changed : 10/17/2023 9:00:02 AM
(modified after loading)
Sample Info : IB, Hexane/i-PrOH = 95/5, 0.8 mL/min, 30 oC, 230 nm

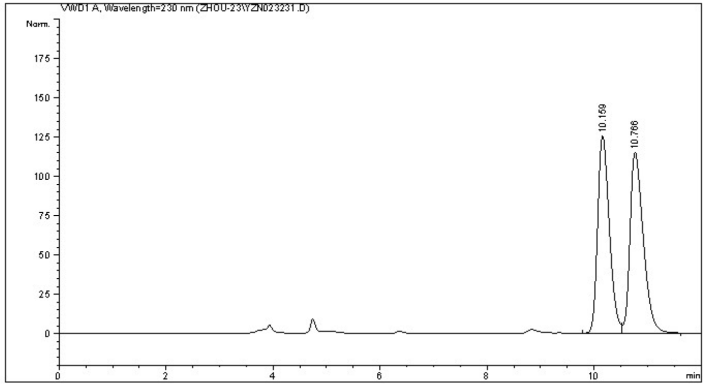

===
Area Percent Report
===

Sorted By : Signal
Multiplier: : 1.0000
Dilution: : 1.0000
Use Multiplier & Dilution Factor with ISTDs

Signal 1: VWD1 A, Wavelength=230 nm

Peak #	RetTime [min]	Type	Width [min]	Area mAU *s	Height [mAU]	Area %
1	10.159	BV	0.2354	1904.47192	125.44061	48.9206
2	10.766	VB	0.2642	1988.51709	115.10017	51.0794

Totals : 3892.98901 240.54079

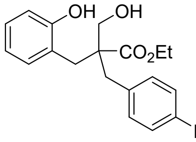

(+/-)-4c

===
*** End of Report ***

Instrument 1 10/17/2023 9:00:10 AM Page 1 of 1

Data File C:\CHEM32\1\DATA\ZHOU-23\YZN023232.D
Sample Name: HW-14-73CTM

===

Acq. Operator :
Acq. Instrument : Instrument 1 Location : -
Injection Date : 5/31/2023 5:22:40 AM
Acq. Method : C:\CHEM32\1\METHODS\DEF_LC.M
Last changed : 5/31/2023 5:22:17 AM
(modified after loading)
Analysis Method : C:\CHEM32\1\METHODS\DEF_LC.M
Last changed : 10/17/2023 9:01:10 AM
(modified after loading)
Sample Info : IB, Hexane/i-PrOH = 95/5, 0.8 mL/min, 30 oC, 230 nm

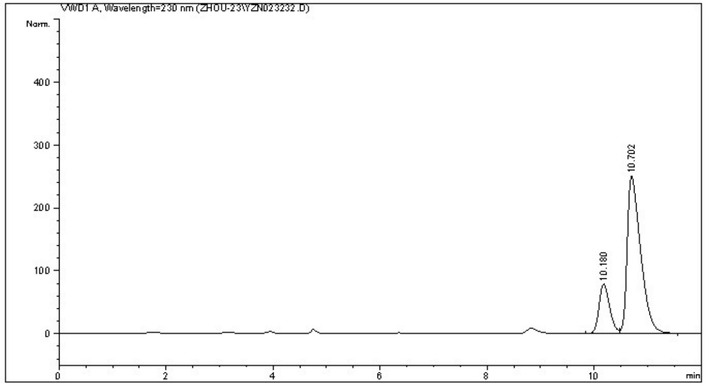

===
Area Percent Report
===

Sorted By : Signal
Multiplier: : 1.0000
Dilution: : 1.0000
Use Multiplier & Dilution Factor with ISTDs

Signal 1: VWD1 A, Wavelength=230 nm

Peak #	RetTime [min]	Type	Width [min]	Area mAU *s	Height [mAU]	Area %
1	10.180	BV	0.2112	1080.59412	78.68358	19.9336
2	10.702	VB	0.2638	4340.38330	249.85710	80.0664

Totals : 5420.97742 328.54068

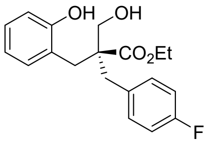

(+)-4c

===
*** End of Report ***

Instrument 1 10/17/2023 9:01:14 AM Page 1 of 1

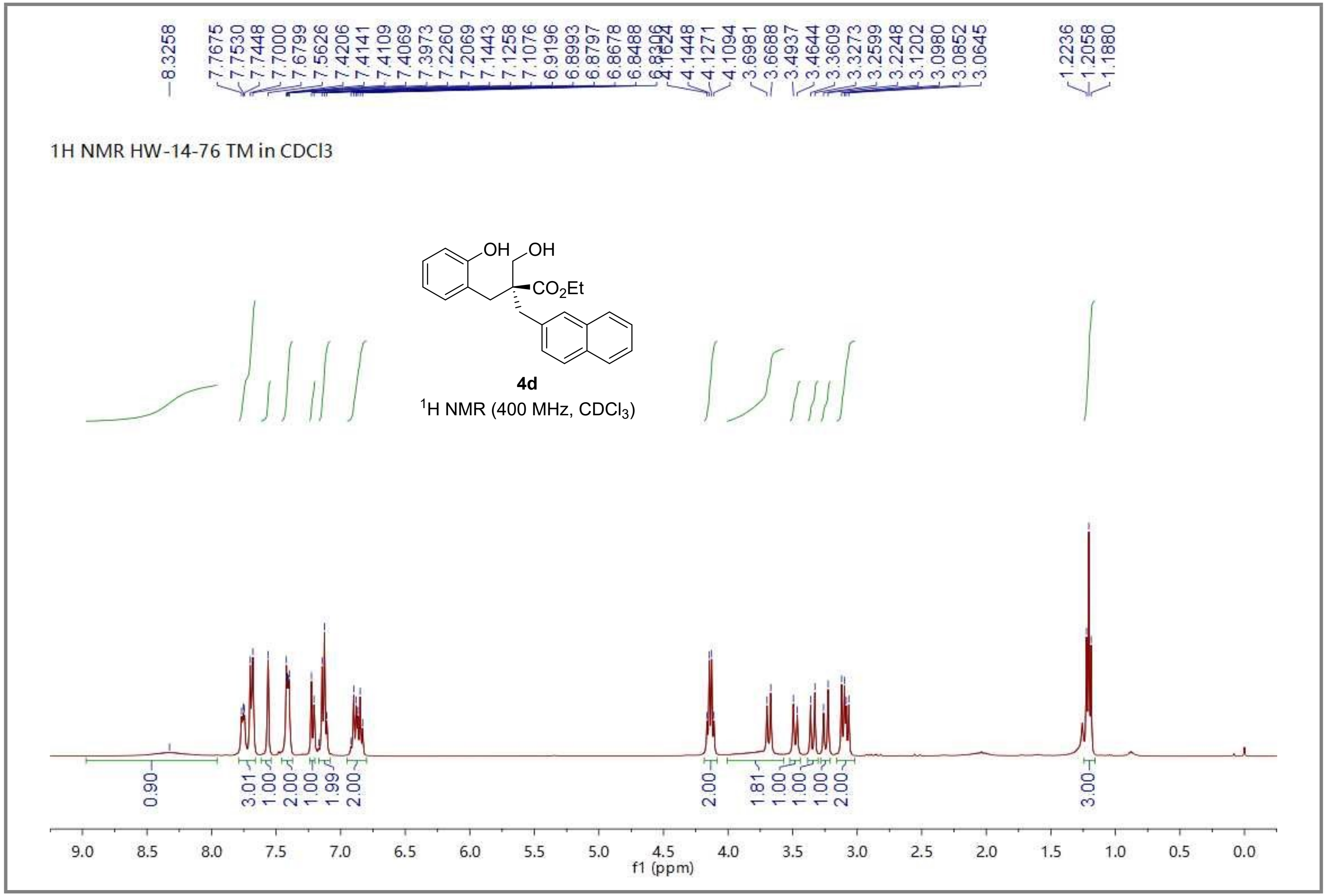
1H NMR HW-14-76 TM in CDCl3
8.3258
7.7675
7.7530
7.7448
7.7000
7.6799
7.5626
7.4206
7.4141
7.4109
7.4069
7.3973
7.2260
7.2069
7.1443
7.1258
7.1076
6.9196
6.8993
6.8797
6.8678
6.8488
6.8306
4.1624
4.1448
4.1271
4.1094
3.6981
3.6688
3.4937
3.4644
3.3609
3.3273
3.2599
3.2248
3.1202
3.0980
3.0852
3.0645
1.2236
1.2058
1.1880
OH
OH
CO2Et
4d
1H NMR (400 MHz, CDCl3)
0.90
3.01
1.00
2.00
1.00
1.99
2.00
2.00
1.81
1.00
1.00
1.00
2.00
3.00
9.0
8.5
8.0
7.5
7.0
6.5
6.0
5.5
5.0
4.5
4.0
3.5
3.0
2.5
2.0
1.5
1.0
0.5
0.0
f1 (ppm)

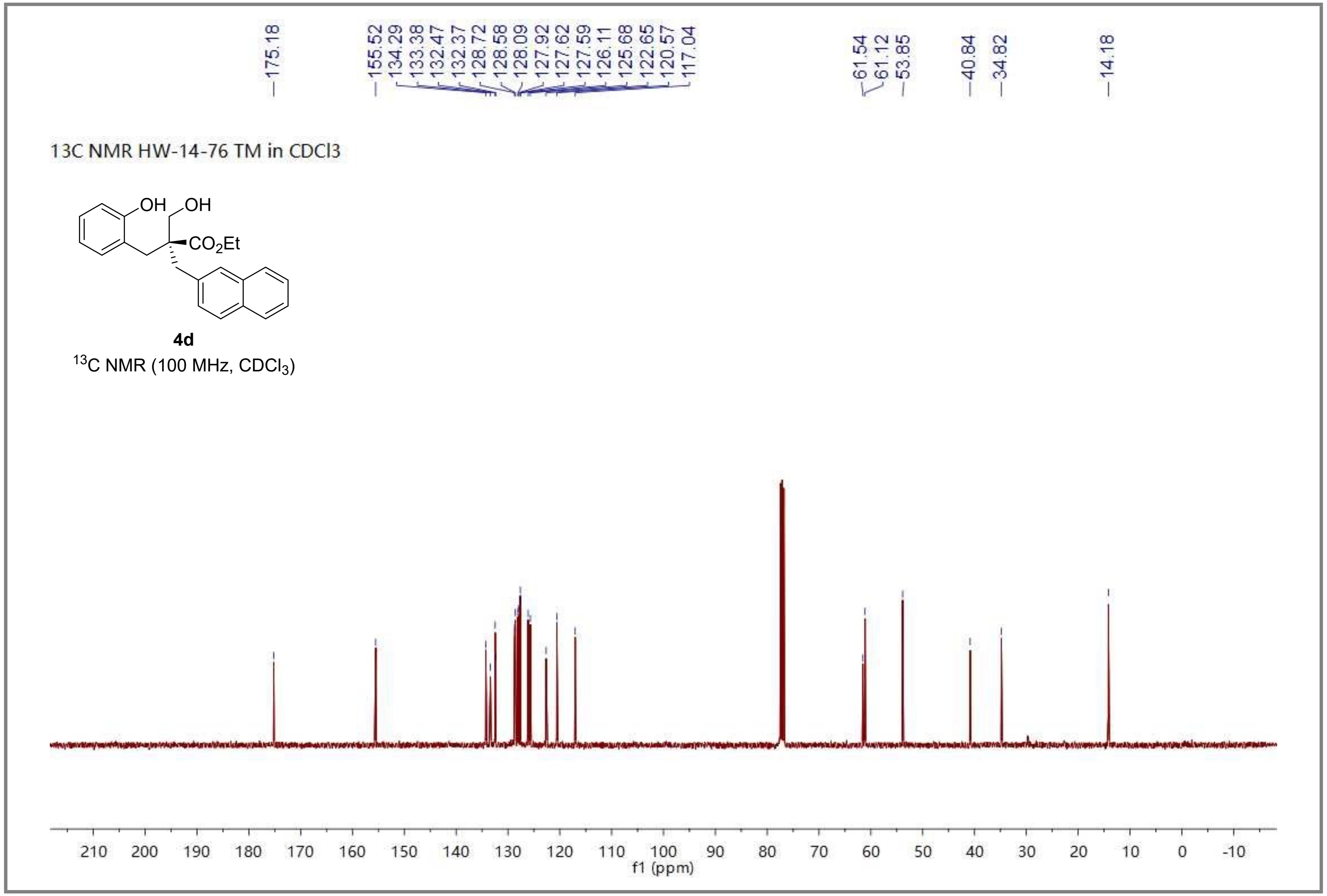
175.18
155.52
134.29
133.38
132.47
132.37
128.72
128.58
128.09
127.92
127.62
127.59
126.11
125.68
122.65
120.57
117.04
61.54
61.12
53.85
40.84
34.82
14.18
13C NMR HW-14-76 TM in CDCl3
OH
OH
CO2Et
4d
13C NMR (100 MHz, CDCl3)
210 200 190 180 170 160 150 140 130 120 110 100 90 80 70 60 50 40 30 20 10 0 -10
f1 (ppm)

Data File C:\USERS\PUBLIC\DOCUMENTS\CHEMSTATION\1\DATA\2023\YZH003753 15-55-23.D
Sample Name: HW-14-8C TM+/-

===
Acq. Operator : SYSTEM
Sample Operator : SYSTEM
Acq. Instrument : HPLC1260 II Location : 1
Injection Date : 3/24/2023 3:55:23 PM Inj : 1
Inj Volume : No inj
Acq. Method : C:\USERS\PUBLIC\DOCUMENTS\CHEMSTATION\1\METHODS\def_LC1.M
Last changed : 3/24/2023 2:09:49 PM by SYSTEM
(modified after loading)
Analysis Method : C:\USERS\PUBLIC\DOCUMENTS\CHEMSTATION\1\METHODS\def_LC1.M
Last changed : 10/16/2023 9:21:23 PM by SYSTEM
(modified after loading)
Sample Info : IA, Hexane/i-PrOH = 95/5, 0.8 mL/min, 30 oC, 230 nm

Additional Info : Peak(s) manually integrated

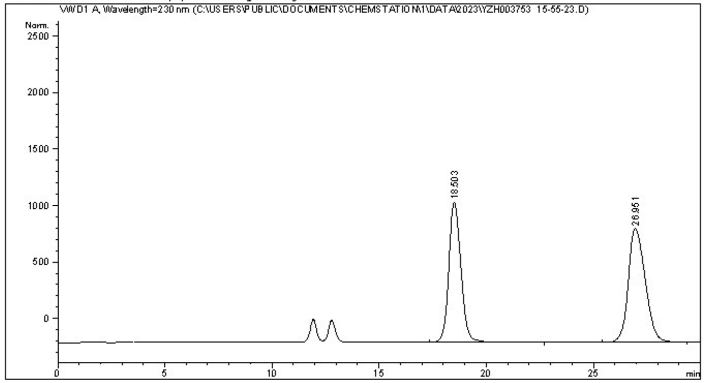

===
Area Percent Report
===

Sorted By : Signal
Multiplier : 1.0000
Dilution : 1.0000
Sample Amount: : 1.00000 [ng/ul] (not used in calc.)
Do not use Multiplier & Dilution Factor with ISTDs

Signal 1: VWD1 A, Wavelength=230 nm

Peak #	RetTime [min]	Type	Width [min]	Area [mAU*s]	Height [mAU]	Area %
1	18.503	BB	0.6219	3.20917e4	812.29633	46.9001
2	26.951	BB	0.8646	3.63340e4	662.75378	53.0999

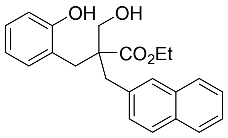

(+/-)-4d

HPLC1260 II 10/16/2023 9:21:27 PM SYSTEM Page 1 of 2

Data File C:\USERS\PUBLIC\DOCUMENTS\CHEMSTATION\1\DATA\2023\YZH004328 10-47-19.D
Sample Name: HW-14-76TM

===
Acq. Operator : SYSTEM
Sample Operator : SYSTEM
Acq. Instrument : HPLC1260 II Location : 1
Injection Date : 6/6/2023 10:47:19 AM Inj : 1
Inj Volume : No inj
Acq. Method : C:\USERS\PUBLIC\DOCUMENTS\CHEMSTATION\1\METHODS\def_LC1.M
Last changed : 6/6/2023 10:41:04 AM by SYSTEM
(modified after loading)
Analysis Method : C:\USERS\PUBLIC\DOCUMENTS\CHEMSTATION\1\METHODS\def_LC1.M
Last changed : 10/16/2023 9:20:30 PM by SYSTEM
(modified after loading)
Sample Info : IA, Hexane/i-PrOH = 95/5, 0.8 mL/min, 30 oC, 230 nm

Additional Info : Peak(s) manually integrated

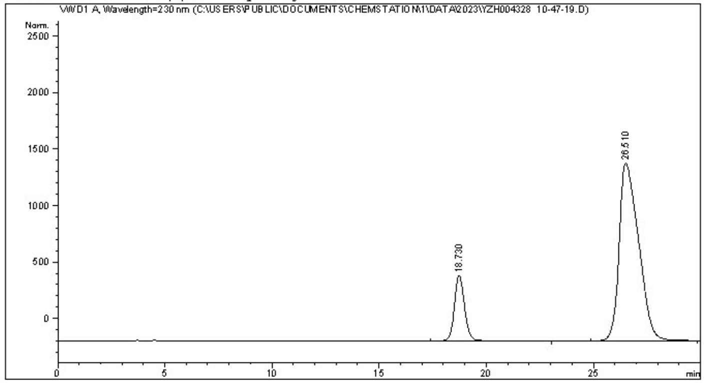

===
Area Percent Report
===

Sorted By : Signal
Multiplier : 1.0000
Dilution : 1.0000
Sample Amount: : 1.00000 [ng/ul] (not used in calc.)
Do not use Multiplier & Dilution Factor with ISTDs

Signal 1: VWD1 A, Wavelength=230 nm

Peak #	RetTime [min]	Type	Width [min]	Area [mAU*s]	Height [mAU]	Area %
1	18.730	BB	0.5175	1.46792e4	437.40421	16.4315
2	26.510	BB	1.0464	7.46569e4	1182.81482	83.5685

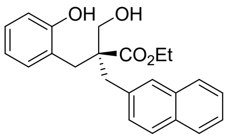

(+)-4d

HPLC1260 II 10/16/2023 9:20:35 PM SYSTEM Page 1 of 2

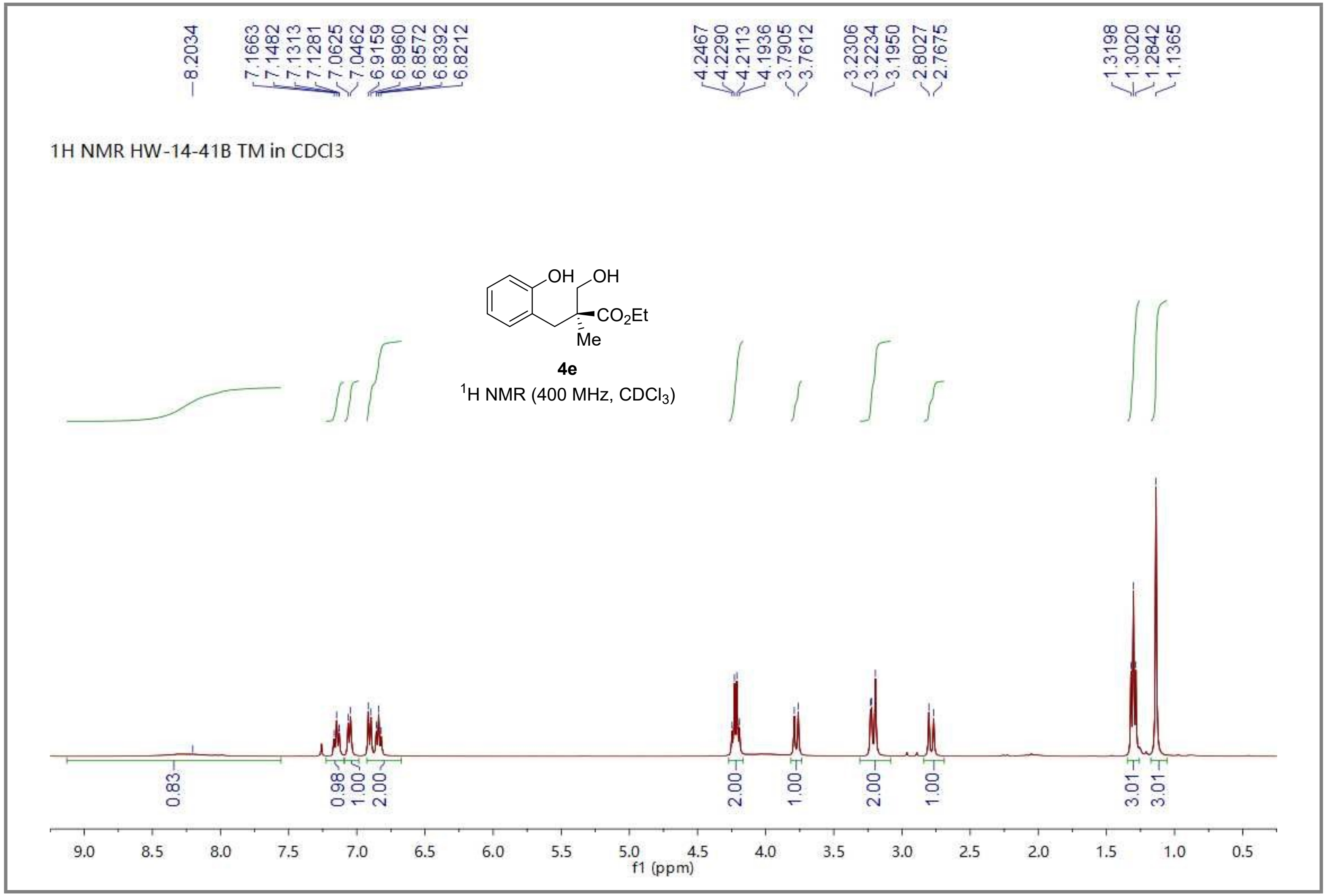

8.2034
7.1663
7.1482
7.1313
7.1281
7.0625
7.0462
6.9159
6.8960
6.8572
6.8392
6.8212
4.2467
4.2290
4.2113
4.1936
3.7905
3.7612
3.2306
3.2234
3.1950
2.8027
2.7675
1.3198
1.3020
1.2842
1.1365
1H NMR HW-14-41B TM in CDCl3
OH
OH
CO2Et
Me
4e
1H NMR (400 MHz, CDCl3)
0.83
0.98
1.00
2.00
2.00
1.00
2.00
1.00
3.01
3.01
9.0
8.5
8.0
7.5
7.0
6.5
6.0
5.5
5.0
4.5
4.0
3.5
3.0
2.5
2.0
1.5
1.0
0.5
f1 (ppm)

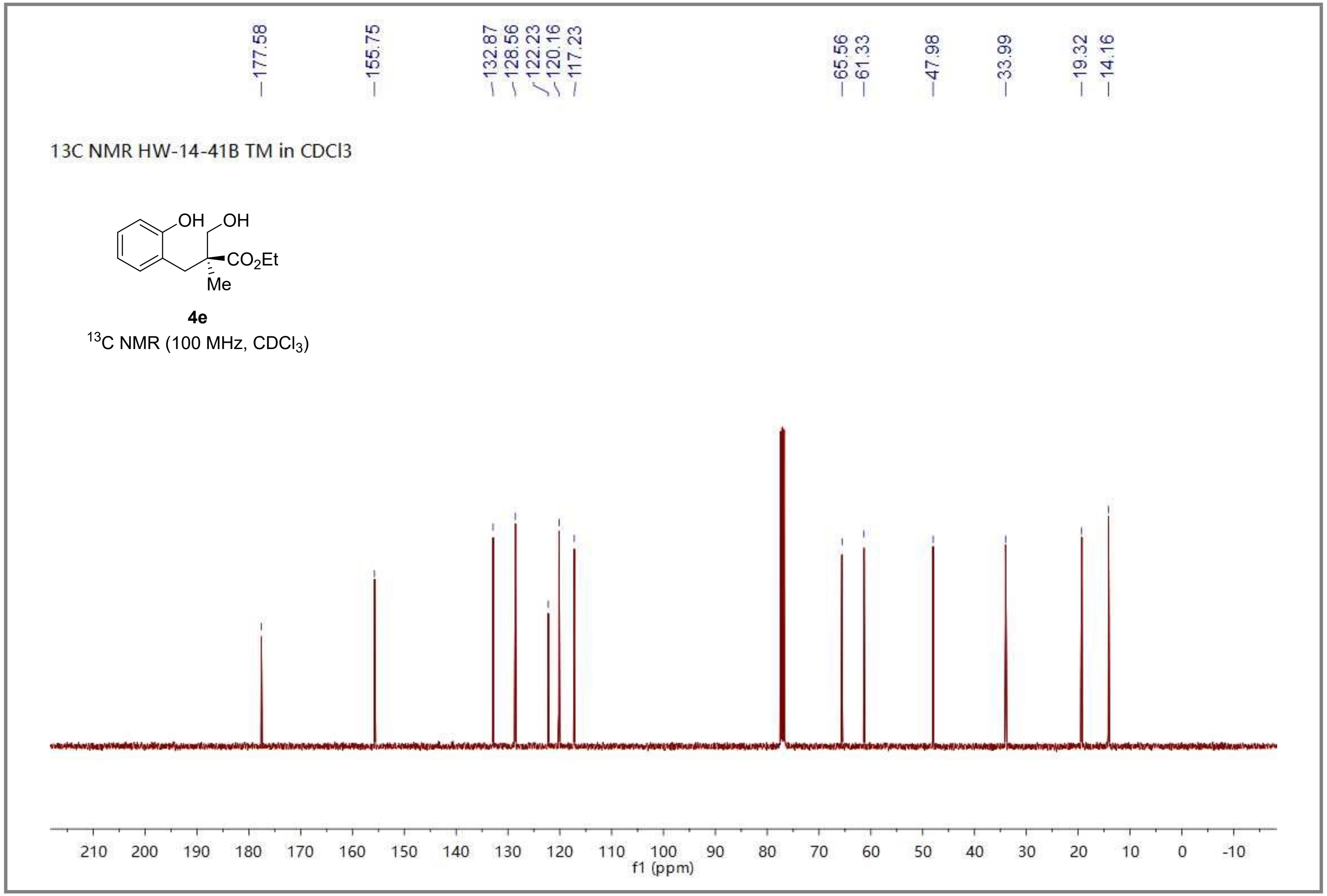
177.58
155.75
132.87
128.56
122.23
120.16
117.23
65.56
61.33
47.98
33.99
19.32
14.16
13C NMR HW-14-41B TM in CDCl3
OH
OH
CO2Et
Me
4e
13C NMR (100 MHz, CDCl3)
210 200 190 180 170 160 150 140 130 120 110 100 90 80 70 60 50 40 30 20 10 0 -10
f1 (ppm)

Data File C:\CHEM32\1\DATA\ZHOU-23\YZN022975.D
Sample Name: HW-14-41B TM+/-

===

Acq. Operator :
Acq. Instrument : Instrument 1 Location : -
Injection Date : 4/26/2023 4:21:26 AM
Acq. Method : C:\CHEM32\1\METHODS\DEF_LC.M
Last changed : 4/26/2023 4:19:55 AM
(modified after loading)
Analysis Method : C:\CHEM32\1\METHODS\DEF_LC.M
Last changed : 10/17/2023 9:17:42 AM
(modified after loading)
Sample Info : IB, Hexane/i-PrOH = 95/5, 0.8 mL/min, 30 oC, 230 nm

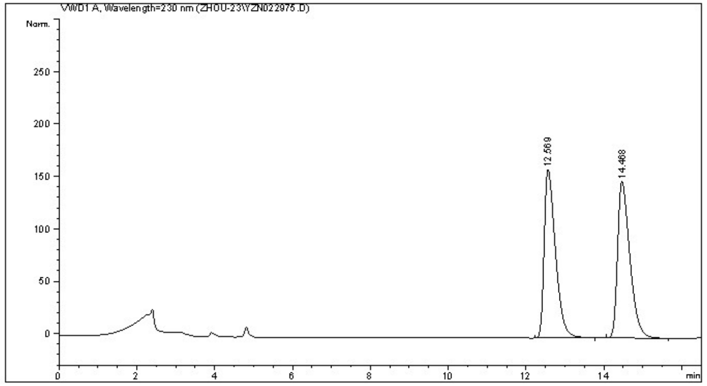

===
Area Percent Report
===

Sorted By : Signal
Multiplier: : 1.0000
Dilution: : 1.0000
Use Multiplier & Dilution Factor with ISTDs

Signal 1: VWD1 A, Wavelength=230 nm

Peak #	RetTime [min]	Type	Width [min]	Area mAU *s	Height [mAU]	Area %
1	12.569	BB	0.3056	3226.27905	160.67441	49.6838
2	14.468	BB	0.3342	3267.35059	149.16887	50.3162
Totals :				6493.62964	309.84328	

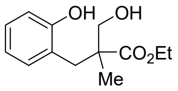

(+/-)-4e

===
*** End of Report ***

Instrument 1 10/17/2023 9:17:46 AM Page 1 of 1

Data File C:\CHEM32\1\DATA\ZHOU-23\YZN022976.D
Sample Name: HW-14-41B TM

===

Acq. Operator :
Acq. Instrument : Instrument 1 Location : -
Injection Date : 4/26/2023 4:42:32 AM
Acq. Method : C:\CHEM32\1\METHODS\DEF_LC.M
Last changed : 4/26/2023 4:41:54 AM
(modified after loading)
Analysis Method : C:\CHEM32\1\METHODS\DEF_LC.M
Last changed : 10/17/2023 9:17:42 AM
(modified after loading)
Sample Info : IB, Hexane/i-PrOH = 95/5, 0.8 mL/min, 30 oC, 230 nm

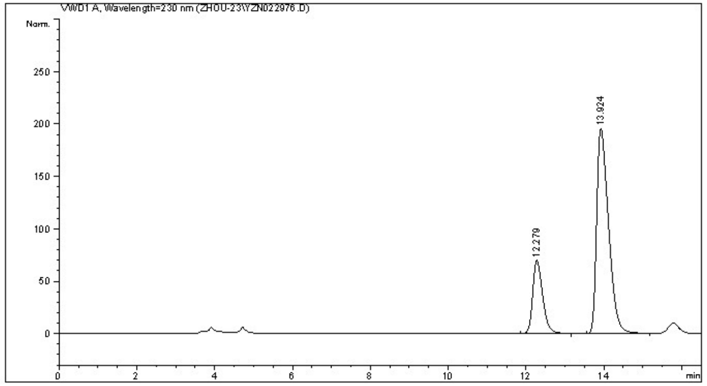

===
Area Percent Report
===

Sorted By : Signal
Multiplier: : 1.0000
Dilution: : 1.0000
Use Multiplier & Dilution Factor with ISTDs

Signal 1: VWD1 A, Wavelength=230 nm

Peak #	RetTime [min]	Type	Width [min]	Area mAU *s	Height [mAU]	Area %
1	12.279	BB	0.2725	1238.63635	69.80949	23.0816
2	13.924	BB	0.3279	4127.69531	195.49022	76.9184
Totals :				5366.33167	265.29971	

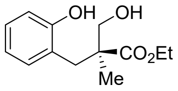

(+)-4e

===
*** End of Report ***

Instrument 1 10/17/2023 9:18:41 AM Page 1 of 1

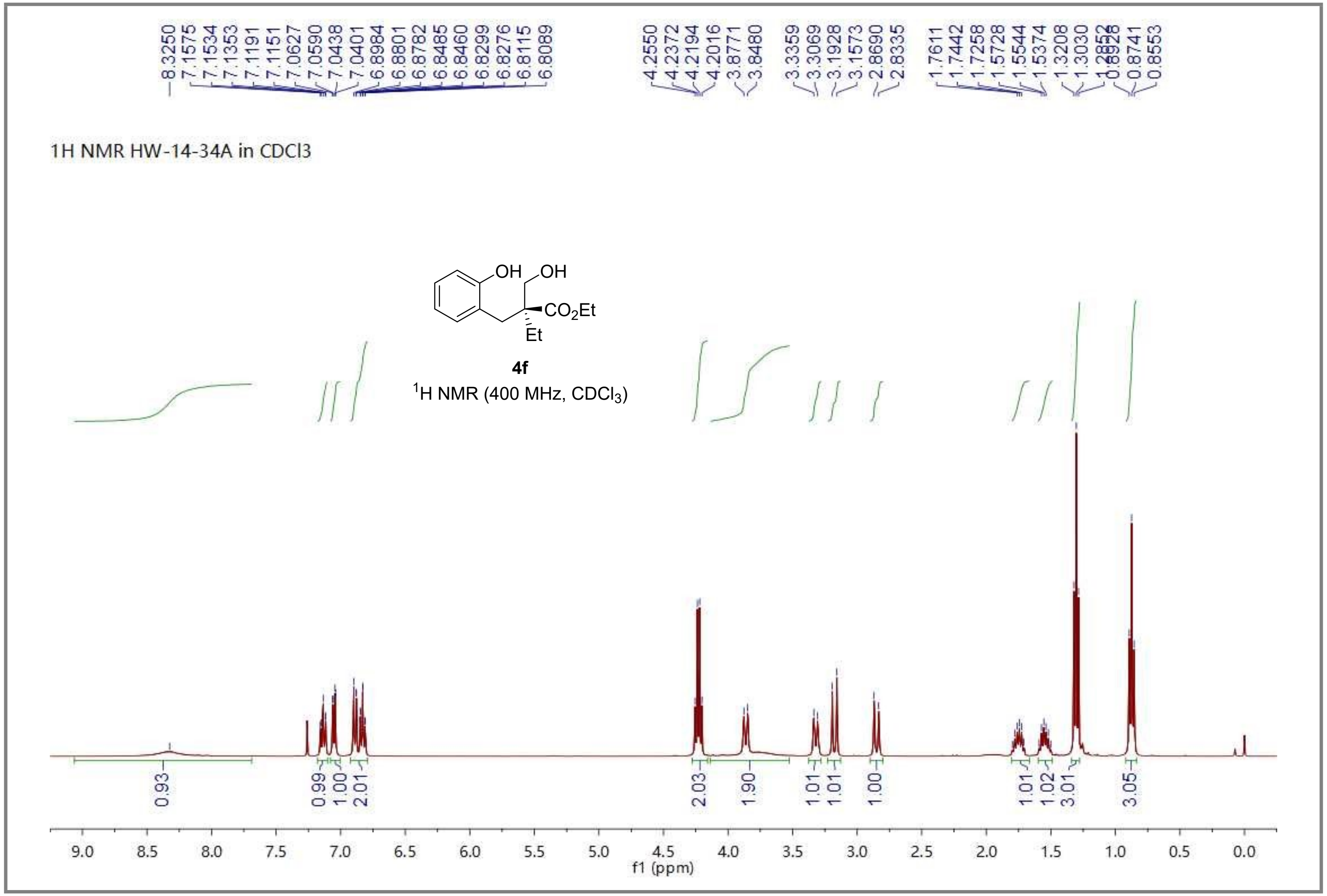

8.3250
7.1575
7.1534
7.1353
7.1191
7.1151
7.0627
7.0590
7.0438
7.0401
6.8984
6.8801
6.8782
6.8485
6.8460
6.8299
6.8276
6.8115
6.8089
4.2550
4.2372
4.2194
4.2016
3.8771
3.8480
3.3359
3.3069
3.1928
3.1573
2.8690
2.8335
1.7611
1.7442
1.7258
1.5728
1.5544
1.5374
1.3208
1.3030
1.2852
0.8928
0.8741
0.8553
1H NMR HW-14-34A in CDCl3
OH
OH
CO2Et
Et
4f
1H NMR (400 MHz, CDCl3)
0.93
0.99
1.00
2.01
2.03
1.90
1.01
1.01
1.00
1.01
1.02
3.01
3.05
9.0
8.5
8.0
7.5
7.0
6.5
6.0
5.5
5.0
4.5
4.0
3.5
3.0
2.5
2.0
1.5
1.0
0.5
0.0
f1 (ppm)

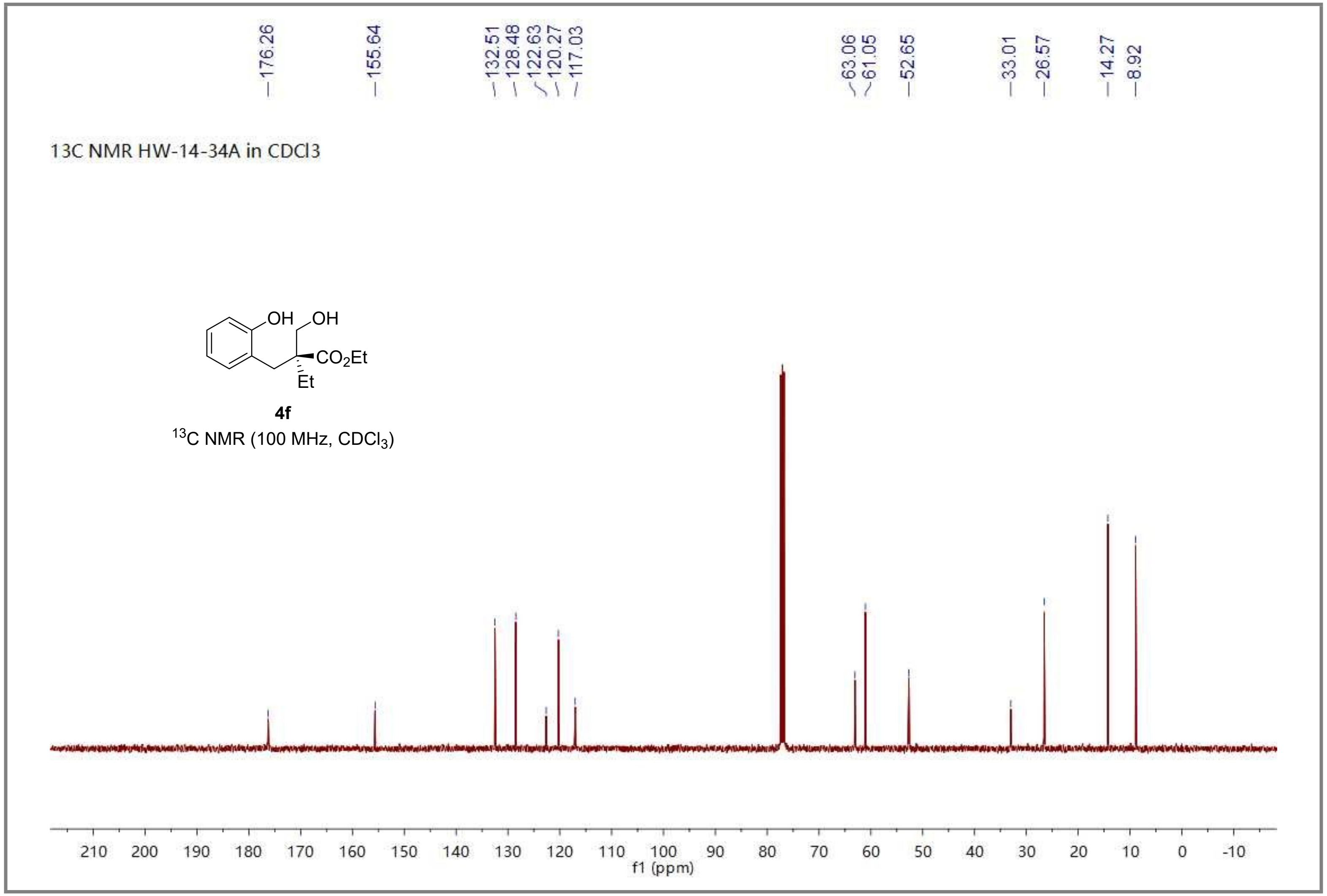
176.26
155.64
132.51
128.48
122.63
120.27
117.03
63.06
61.05
52.65
33.01
26.57
14.27
8.92
13C NMR HW-14-34A in CDCl3
OH
OH
CO2Et
Et
4f
13C NMR (100 MHz, CDCl3)
210 200 190 180 170 160 150 140 130 120 110 100 90 80 70 60 50 40 30 20 10 0 -10
f1 (ppm)

Data File C:\CHEM32\1\DATA\ZHOU-23\YZN022949.D
Sample Name: HW-14-32A TM+/-

===
Acq. Operator :
Acq. Instrument : Instrument 1 Location : -
Injection Date : 4/23/2023 2:11:37 AM
Acq. Method : C:\CHEM32\1\METHODS\DEF_LC.M
Last changed : 4/23/2023 1:59:02 AM
(modified after loading)
Analysis Method : C:\CHEM32\1\METHODS\DEF_LC.M
Last changed : 10/18/2023 8:07:03 AM
(modified after loading)
Sample Info : IB, Hexane/i-PrOH = 95/5, 0.8 mL/min, 30 oC, 230 nm

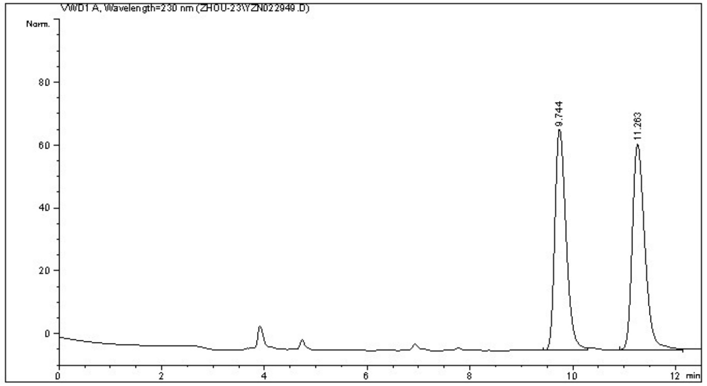

===
Area Percent Report
===

Sorted By : Signal
Multiplier: : 1.0000
Dilution: : 1.0000
Use Multiplier & Dilution Factor with ISTDs

Signal 1: VWD1 A, Wavelength=230 nm

Peak #	RetTime [min]	Type	Width [min]	Area mAU *s	Height [mAU]	Area %
1	9.744	BV	0.2347	1063.41235	70.32623	49.1143
2	11.263	BB	0.2591	1101.76733	65.43362	50.8857
Totals :				2165.17969	135.75985	

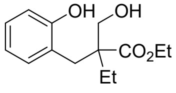

(+/-)-4f

===
*** End of Report ***

Instrument 1 10/18/2023 8:07:08 AM Page 1 of 1

Data File C:\CHEM32\1\DATA\ZHOU-23\YZN022950.D
Sample Name: HW-14-32A TM

===
Acq. Operator :
Acq. Instrument : Instrument 1 Location : -
Injection Date : 4/23/2023 2:26:56 AM
Acq. Method : C:\CHEM32\1\METHODS\DEF_LC.M
Last changed : 4/23/2023 2:25:56 AM
(modified after loading)
Analysis Method : C:\CHEM32\1\METHODS\DEF_LC.M
Last changed : 10/18/2023 8:08:15 AM
(modified after loading)
Sample Info : IB, Hexane/i-PrOH = 95/5, 0.8 mL/min, 30 oC, 230 nm

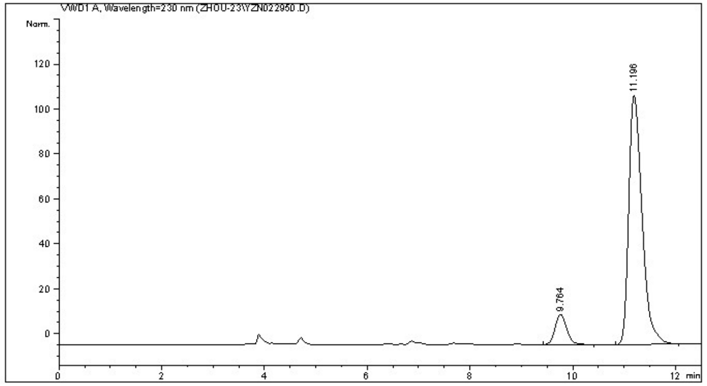

===
Area Percent Report
===

Sorted By : Signal
Multiplier: : 1.0000
Dilution: : 1.0000
Use Multiplier & Dilution Factor with ISTDs

Signal 1: VWD1 A, Wavelength=230 nm

Peak #	RetTime [min]	Type	Width [min]	Area mAU *s	Height [mAU]	Area %
1	9.764	BB	0.2472	215.87962	13.53471	9.7641
2	11.196	BB	0.2783	1995.07495	110.88701	90.2359
Totals :				2210.95457	124.42172	

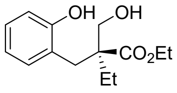

(+)-4f

===
*** End of Report ***

Instrument 1 10/18/2023 8:08:18 AM Page 1 of 1

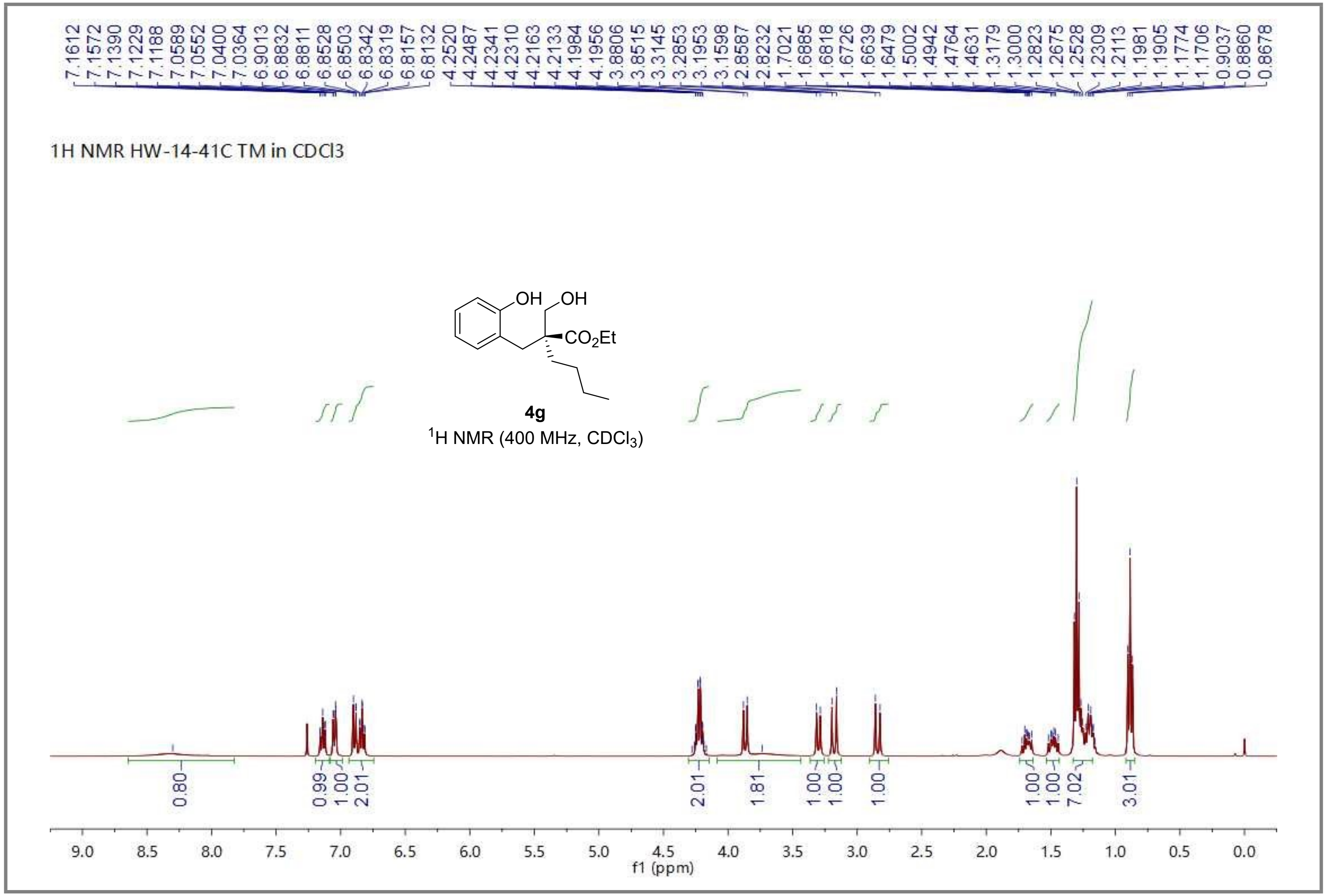
1H NMR HW-14-41C TM in CDCl3
OH
OH
CO2Et
4g
1H NMR (400 MHz, CDCl3)
f1 (ppm)

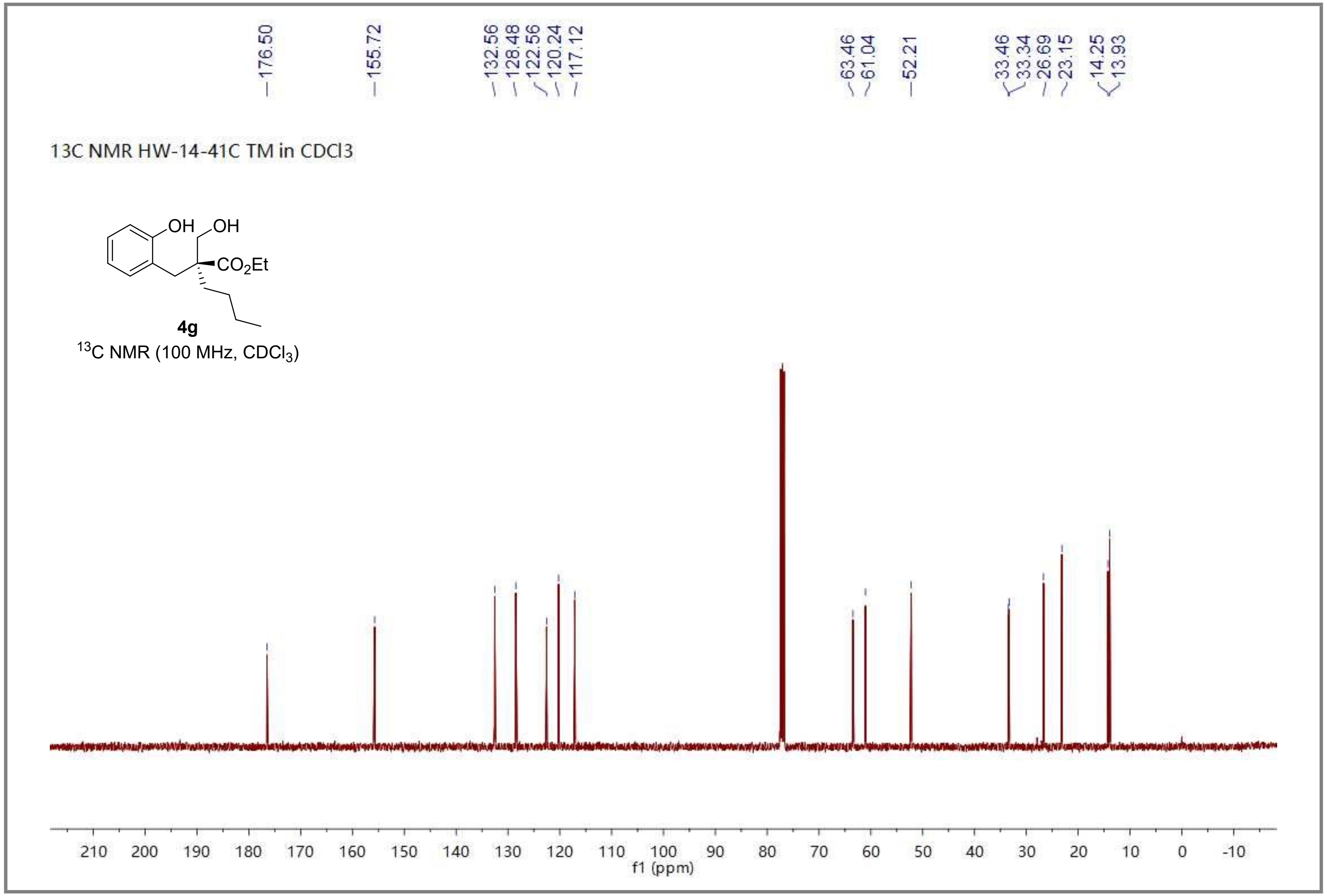

176.50
155.72
132.56
128.48
122.56
120.24
117.12
63.46
61.04
52.21
33.46
33.34
26.69
23.15
14.25
13.93
13C NMR HW-14-41C TM in CDCl3
OH
OH
CO2Et
4g
13C NMR (100 MHz, CDCl3)
210
200
190
180
170
160
150
140
130
120
110
100
90
80
70
60
50
40
30
20
10
0
-10
f1 (ppm)

Data File C:\CHEM32\1\DATA\ZHOU-23\YZN022977.D
Sample Name: HW-14-41C TM+/-

===

Acq. Operator :
Acq. Instrument : Instrument 1 Location : -
Injection Date : 4/26/2023 7:45:21 AM
Acq. Method : C:\CHEM32\1\METHODS\DEF_LC.M
Last changed : 4/26/2023 7:29:44 AM
(modified after loading)
Analysis Method : C:\CHEM32\1\METHODS\DEF_LC.M
Last changed : 10/17/2023 9:22:15 AM
(modified after loading)
Sample Info : IB, Hexane/i-PrOH = 95/5, 0.8 mL/min, 30 oC, 230 nm

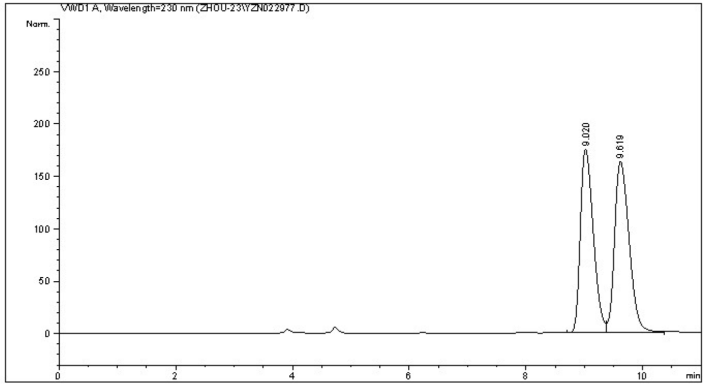

===
Area Percent Report
===

Sorted By : Signal
Multiplier: : 1.0000
Dilution: : 1.0000
Use Multiplier & Dilution Factor with ISTDs

Signal 1: VWD1 A, Wavelength=230 nm

Peak #	RetTime [min]	Type	Width [min]	Area mAU *s	Height [mAU]	Area %
1	9.020	BV	0.2407	2696.60937	175.18466	48.8336
2	9.619	VB	0.2705	2825.43018	163.10492	51.1664

Totals : 5522.03955 338.28958

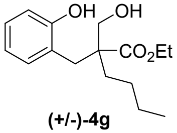

(+/-)-4g

===
*** End of Report ***

Instrument 1 10/17/2023 9:22:18 AM Page 1 of 1

Data File C:\CHEM32\1\DATA\ZHOU-23\YZN022978.D
Sample Name: HW-14-41C TM

===

Acq. Operator :
Acq. Instrument : Instrument 1 Location : -
Injection Date : 4/26/2023 8:05:34 AM
Acq. Method : C:\CHEM32\1\METHODS\DEF_LC.M
Last changed : 4/26/2023 8:05:08 AM
(modified after loading)
Analysis Method : C:\CHEM32\1\METHODS\DEF_LC.M
Last changed : 10/17/2023 9:22:15 AM
(modified after loading)
Sample Info : IB, Hexane/i-PrOH = 95/5, 0.8 mL/min, 30 oC, 230 nm

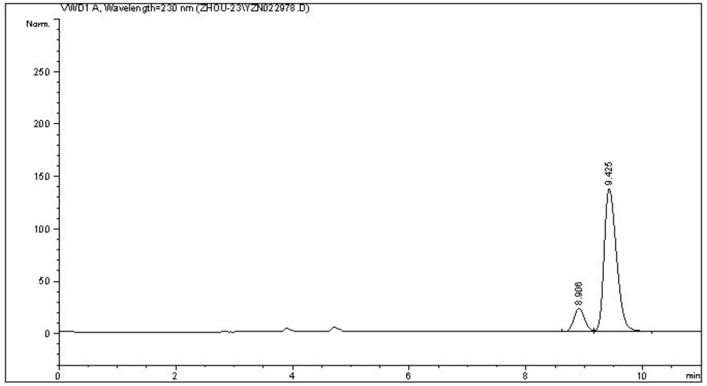

===
Area Percent Report
===

Sorted By : Signal
Multiplier: : 1.0000
Dilution: : 1.0000
Use Multiplier & Dilution Factor with ISTDs

Signal 1: VWD1 A, Wavelength=230 nm

Peak #	RetTime [min]	Type	Width [min]	Area mAU *s	Height [mAU]	Area %
1	8.906	BV	0.2014	284.40286	22.05935	12.5159
2	9.425	VB	0.2268	1987.93518	136.37880	87.4841

Totals : 2272.33804 158.43815

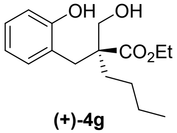

(+)-4g

===
*** End of Report ***

Instrument 1 10/17/2023 9:22:54 AM Page 1 of 1

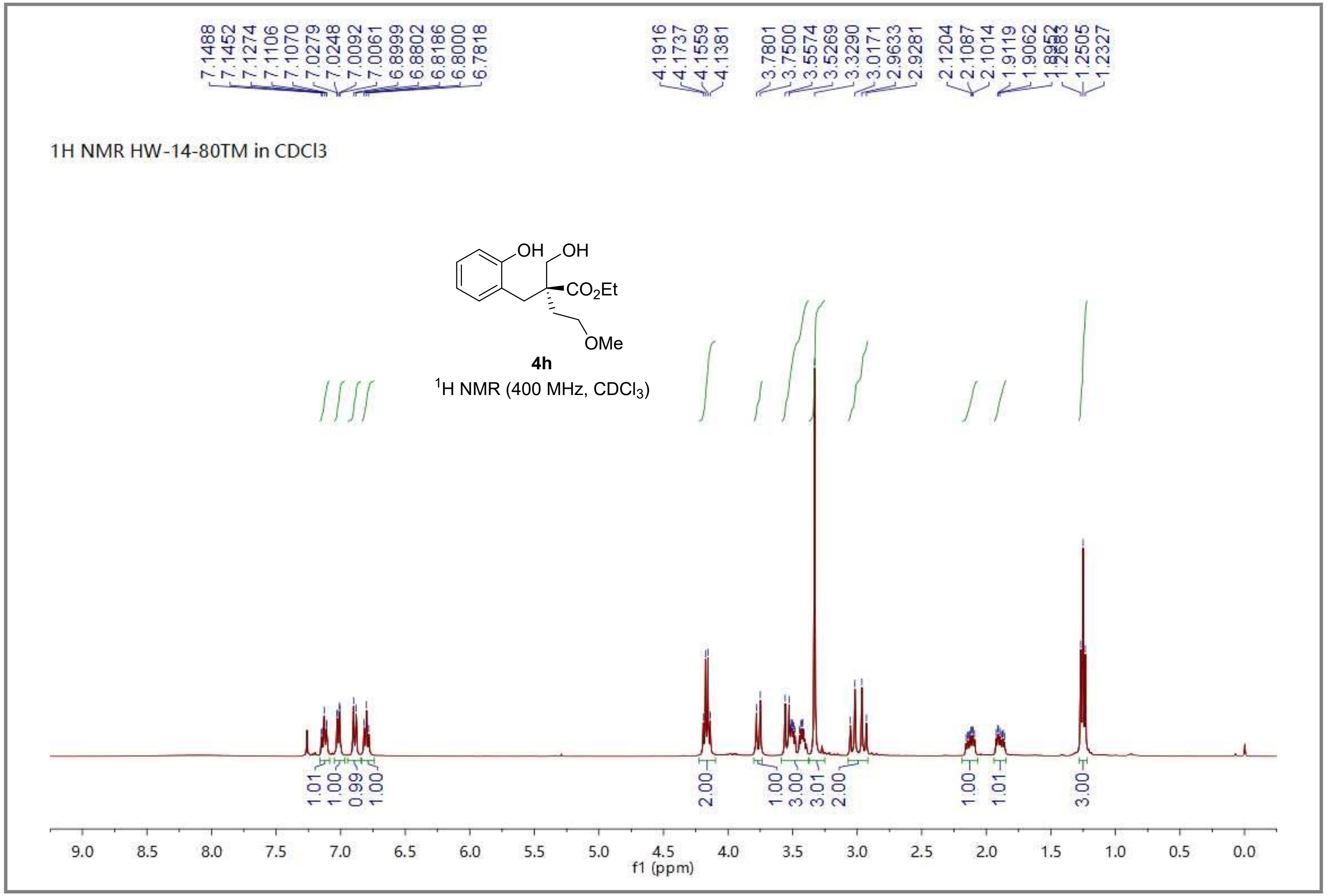

7.1488
7.1452
7.1274
7.1106
7.1070
7.0279
7.0248
7.0092
7.0061
6.8999
6.8802
6.8186
6.8000
6.7818
4.1916
4.1737
4.1559
4.1381
3.7801
3.7500
3.5574
3.5269
3.3290
3.0171
2.9633
2.9281
2.1204
2.1087
2.1014
1.9119
1.9062
1.8952
1.2683
1.2505
1.2327
1H NMR HW-14-80TM in CDCl3
OH
OH
CO_2Et
OMe
4h
^1H NMR (400 MHz, $CDCl_3$)
1.01
1.00
0.99
1.00
2.00
1.00
3.00
3.01
2.00
1.00
1.01
3.00
9.0
8.5
8.0
7.5
7.0
6.5
6.0
5.5
5.0
4.5
4.0
3.5
3.0
2.5
2.0
1.5
1.0
0.5
0.0
f1 (ppm)

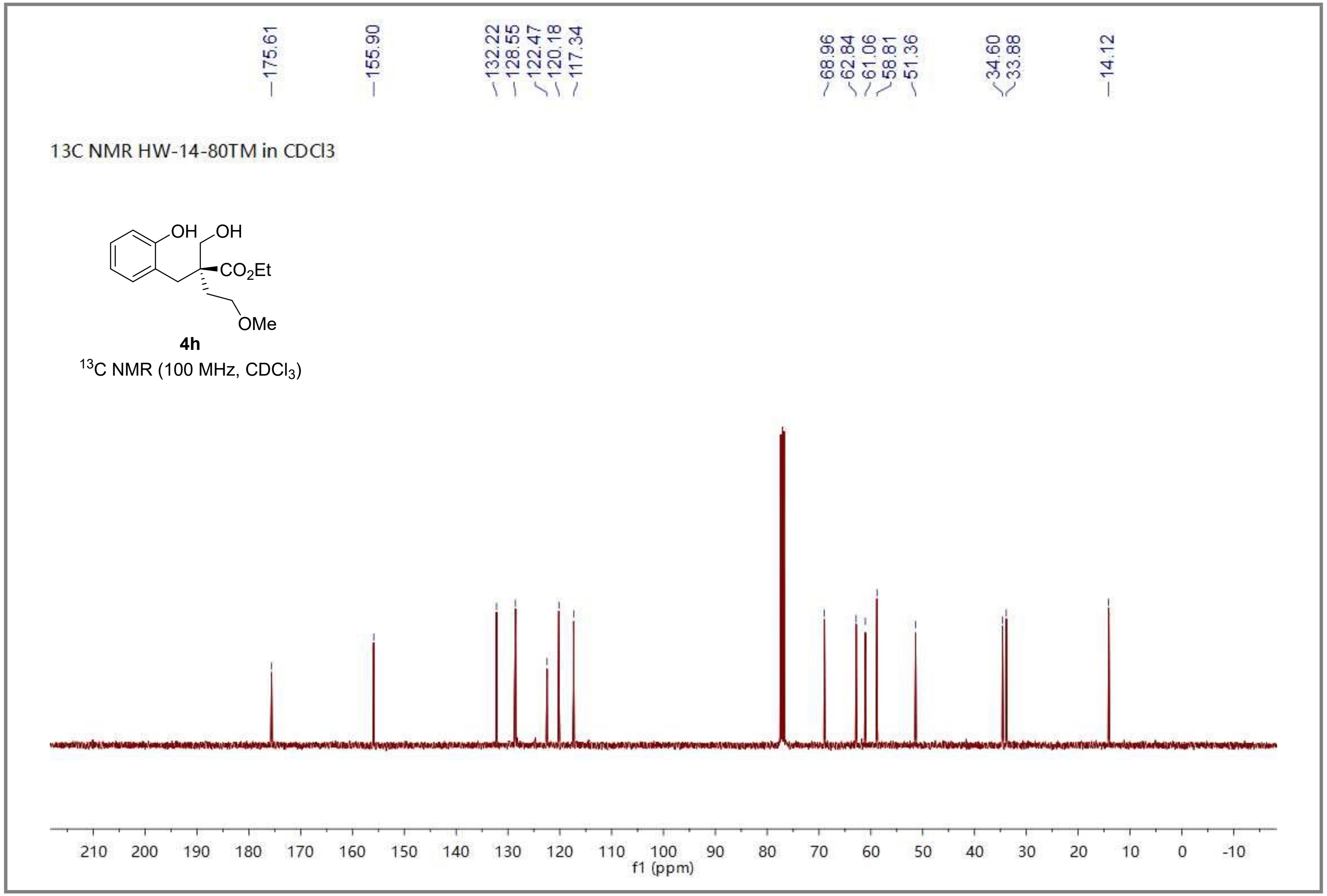

175.61
155.90
132.22
128.55
122.47
120.18
117.34
68.96
62.84
61.06
58.81
51.36
34.60
33.88
14.12
13C NMR HW-14-80TM in CDCl3
OH
OH
CO2Et
OMe
4h
13C NMR (100 MHz, CDCl3)
210 200 190 180 170 160 150 140 130 120 110 100 90 80 70 60 50 40 30 20 10 0 -10
f1 (ppm)

Data File C:\CHEM32\1\DATA\ZHOU-23\YZN023299.D
Sample Name: HW-14-80 TM+/-

===

Acq. Operator :
Acq. Instrument : Instrument 1 Location : -
Injection Date : 6/13/2023 3:10:26 AM
Acq. Method : C:\CHEM32\1\METHODS\DEF_LC.M
Last changed : 6/13/2023 3:09:51 AM
(modified after loading)
Analysis Method : C:\CHEM32\1\METHODS\DEF_LC.M
Last changed : 10/17/2023 9:29:58 AM
(modified after loading)
Sample Info : IB, Hexane/i-PrOH = 95/5, 0.8 mL/min, 30 oC, 230 nm

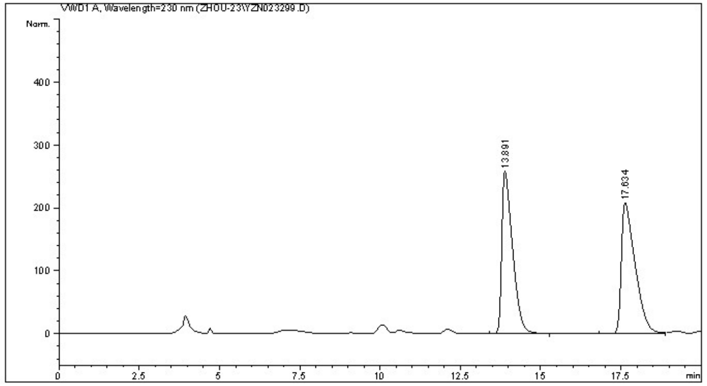

===
Area Percent Report
===

Sorted By : Signal
Multiplier: : 1.0000
Dilution: : 1.0000
Use Multiplier & Dilution Factor with ISTDs

Signal 1: VWD1 A, Wavelength=230 nm

Peak #	RetTime [min]	Type	Width [min]	Area mAU *s	Height [mAU]	Area %
1	13.891	BB	0.3526	6027.66406	258.00723	49.1641
2	17.634	BV	0.4410	6232.62939	208.18640	50.8359

Totals : 1.22603e4 466.19363

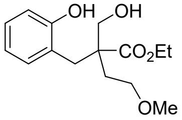

(+/-)-4h

===
*** End of Report ***

Instrument 1 10/17/2023 9:30:01 AM Page 1 of 1

Data File C:\CHEM32\1\DATA\ZHOU-23\YZN023300.D
Sample Name: HW-14-80 TM

===

Acq. Operator :
Acq. Instrument : Instrument 1 Location : -
Injection Date : 6/13/2023 3:38:30 AM
Acq. Method : C:\CHEM32\1\METHODS\DEF_LC.M
Last changed : 6/13/2023 3:37:15 AM
(modified after loading)
Analysis Method : C:\CHEM32\1\METHODS\DEF_LC.M
Last changed : 10/17/2023 9:30:47 AM
(modified after loading)
Sample Info : IB, Hexane/i-PrOH = 95/5, 0.8 mL/min, 30 oC, 230 nm

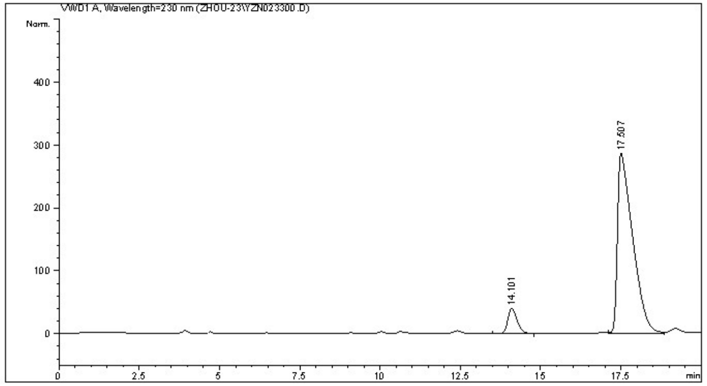

===
Area Percent Report
===

Sorted By : Signal
Multiplier: : 1.0000
Dilution: : 1.0000
Use Multiplier & Dilution Factor with ISTDs

Signal 1: VWD1 A, Wavelength=230 nm

Peak #	RetTime [min]	Type	Width [min]	Area mAU *s	Height [mAU]	Area %
1	14.101	BB	0.3105	798.60327	39.68063	7.7841
2	17.507	VV	0.4791	9460.86816	287.09534	92.2159

Totals : 1.02595e4 326.77596

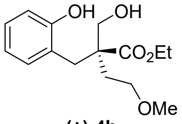

(+)-4h

===
*** End of Report ***

Instrument 1 10/17/2023 9:30:49 AM Page 1 of 1

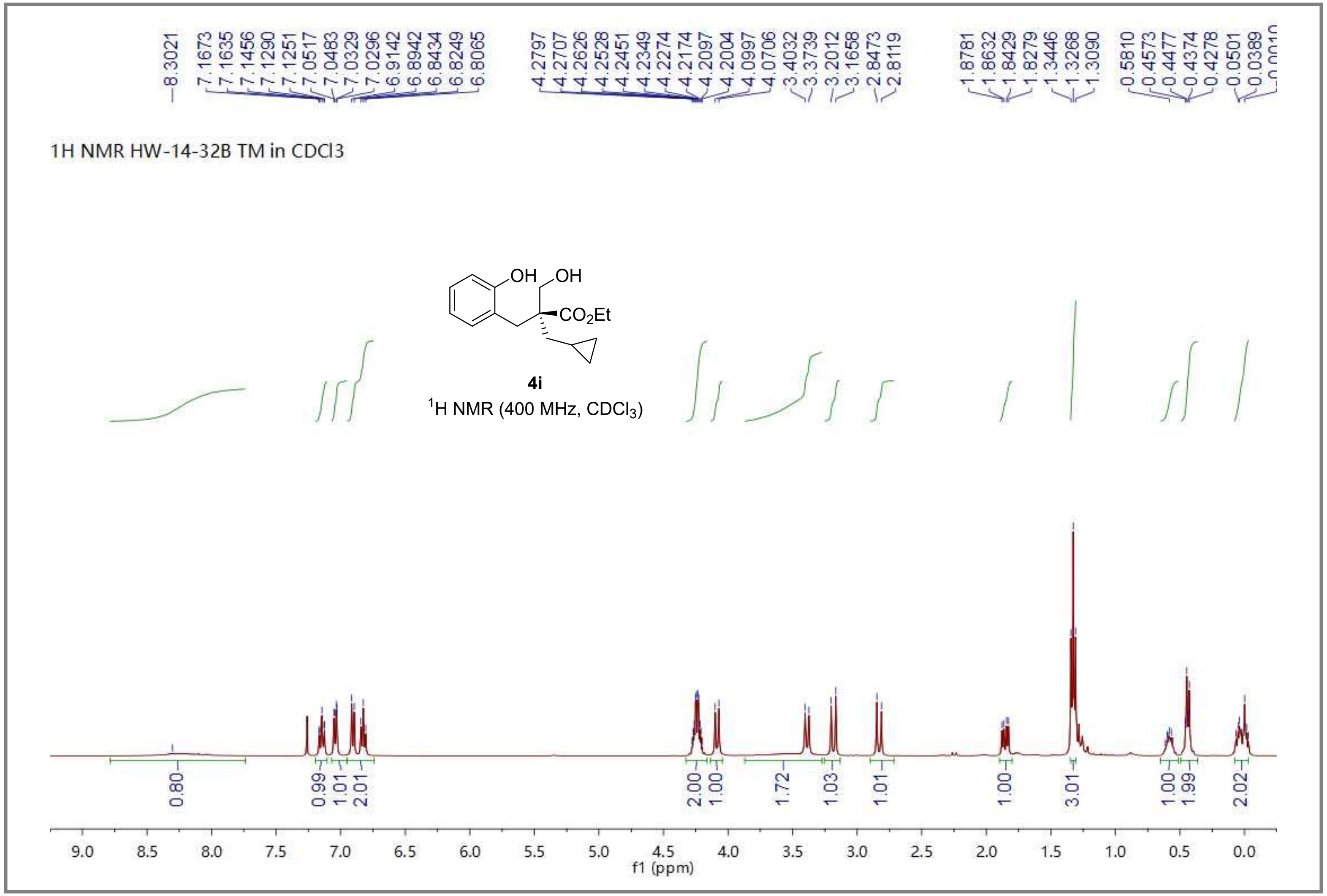
1H NMR HW-14-32B TM in CDCl3
OH
OH
CO2Et
4i
1H NMR (400 MHz, CDCl3)
f1 (ppm)

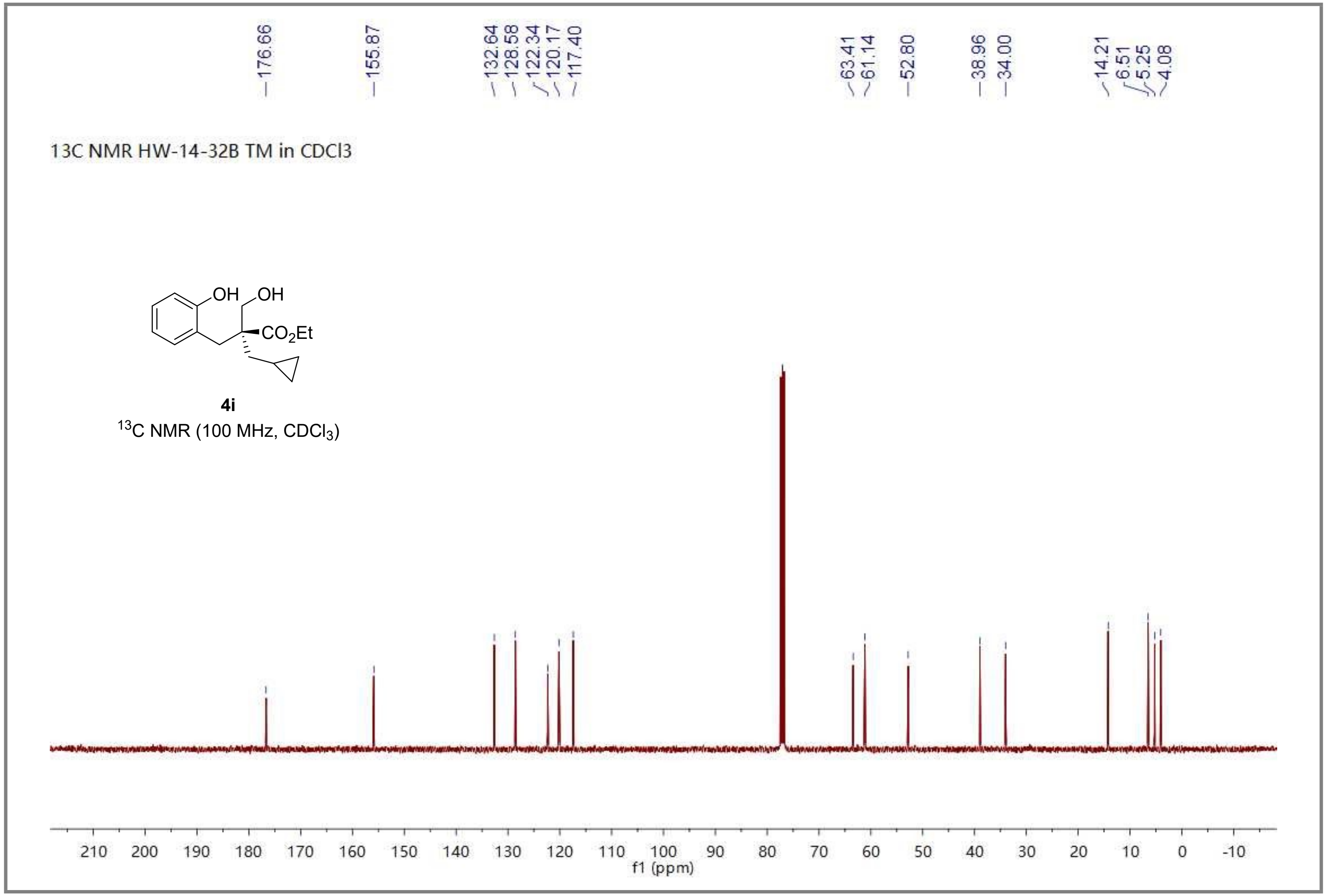
176.66
155.87
132.64
128.58
122.34
120.17
117.40
63.41
61.14
52.80
38.96
34.00
14.21
6.51
5.25
4.08
13C NMR HW-14-32B TM in CDCl3
OH
OH
CO2Et
4i
13C NMR (100 MHz, CDCl3)
210
200
190
180
170
160
150
140
130
120
110
100
90
80
70
60
50
40
30
20
10
0
-10
f1 (ppm)

Data File C:\CHEM32\1\DATA\ZHOU-23\YZN022951.D
Sample Name: HW-14-32B TM+/-

===

Acq. Operator :
Acq. Instrument : Instrument 1 Location : -
Injection Date : 4/23/2023 2:44:05 AM
Acq. Method : C:\CHEM32\1\METHODS\DEF_LC.M
Last changed : 4/23/2023 2:42:40 AM
(modified after loading)
Analysis Method : C:\CHEM32\1\METHODS\DEF_LC.M
Last changed : 10/17/2023 9:36:03 AM
(modified after loading)
Sample Info : IB, Hexane/i-PrOH = 95/5, 0.8 mL/min, 30 oC, 230 nm

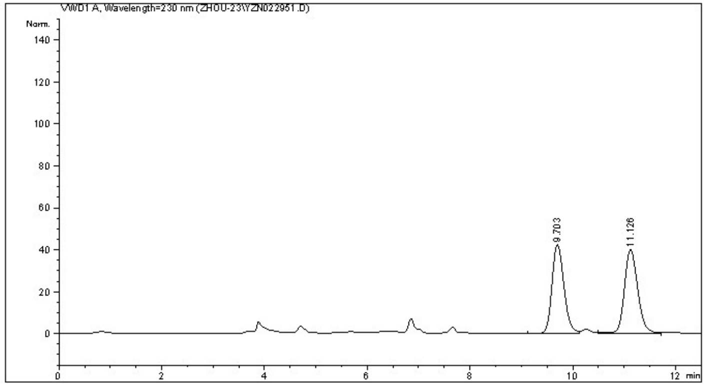

===

Area Percent Report

===

Sorted By : Signal
Multiplier: : 1.0000
Dilution: : 1.0000
Use Multiplier & Dilution Factor with ISTDs

Signal 1: VWD1 A, Wavelength=230 nm

Peak #	RetTime [min]	Type	Width [min]	Area mAU *s	Height [mAU]	Area %
1	9.703	VV	0.2542	691.68689	42.43552	48.3746
2	11.126	VV	0.2850	738.16943	40.04108	51.6254

Totals : 1429.85632 82.47660

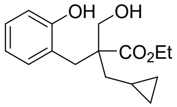

(+/-)-4i

===

*** End of Report ***

Instrument 1 10/17/2023 9:36:06 AM Page 1 of 1

Data File C:\CHEM32\1\DATA\ZHOU-23\YZN023014.D
Sample Name: HW-14-47A TM

===

Acq. Operator :
Acq. Instrument : Instrument 1 Location : -
Injection Date : 5/5/2023 5:59:42 AM
Acq. Method : C:\CHEM32\1\METHODS\DEF_LC.M
Last changed : 5/5/2023 5:54:33 AM
(modified after loading)
Analysis Method : C:\CHEM32\1\METHODS\DEF_LC.M
Last changed : 10/17/2023 9:37:14 AM
(modified after loading)
Sample Info : IB, Hexane/i-PrOH = 95/5, 0.8 mL/min, 30 oC, 230nm

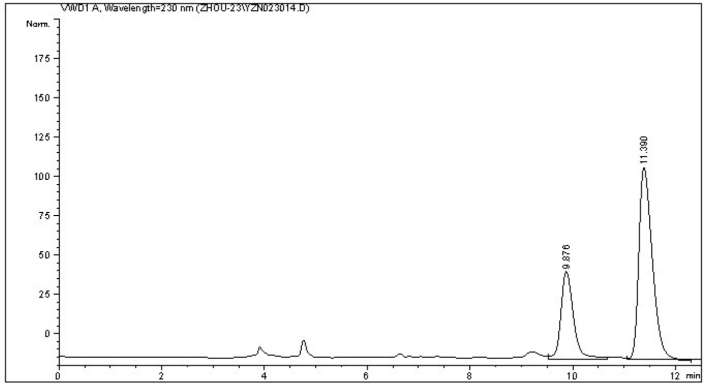

===

Area Percent Report

===

Sorted By : Signal
Multiplier: : 1.0000
Dilution: : 1.0000
Use Multiplier & Dilution Factor with ISTDs

Signal 1: VWD1 A, Wavelength=230 nm

Peak #	RetTime [min]	Type	Width [min]	Area mAU *s	Height [mAU]	Area %
1	9.876	VV	0.2597	961.53351	55.68395	30.2891
2	11.390	VV	0.2760	2212.98486	121.84767	69.7109

Totals : 3174.51837 177.53162

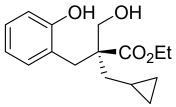

(+)-4i

===

*** End of Report ***

Instrument 1 10/17/2023 9:37:21 AM Page 1 of 1

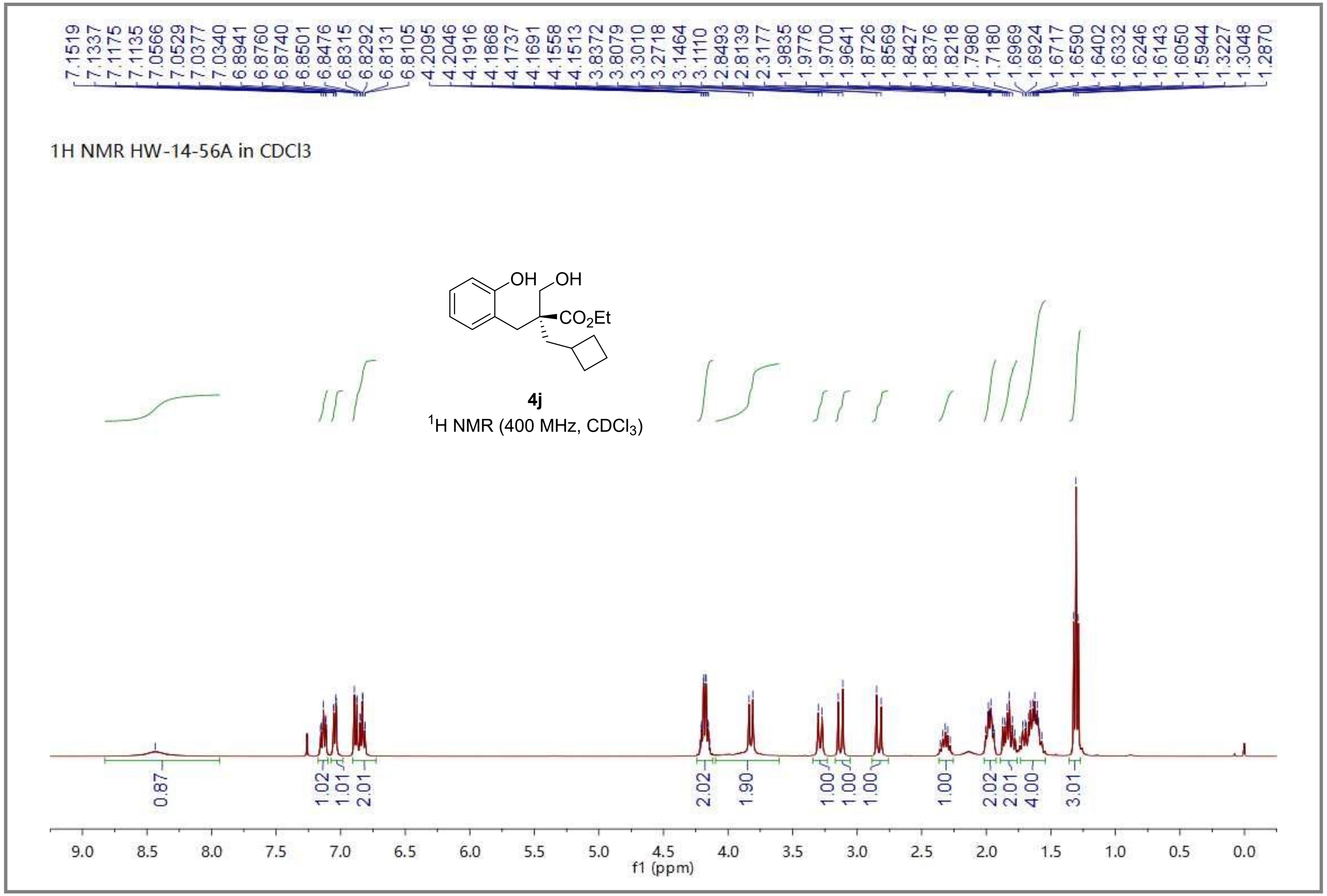

1H NMR HW-14-56A in CDCl3
7.1519
7.1337
7.1175
7.1135
7.0566
7.0529
7.0377
7.0340
6.8941
6.8760
6.8740
6.8501
6.8476
6.8315
6.8292
6.8131
6.8105
4.2095
4.2046
4.1916
4.1868
4.1737
4.1691
4.1558
4.1513
3.8372
3.8079
3.3010
3.2718
3.1464
3.1110
2.8493
2.8139
2.3177
1.9835
1.9776
1.9700
1.9641
1.8726
1.8569
1.8427
1.8376
1.8218
1.7980
1.7180
1.6969
1.6924
1.6717
1.6590
1.6402
1.6332
1.6246
1.6143
1.6050
1.5944
1.3227
1.3048
1.2870
OH
OH
CO2Et
4j
1H NMR (400 MHz, CDCl3)
0.87
1.02
1.01
2.01
2.02
1.90
1.00
1.00
1.00
1.00
2.02
2.01
4.00
3.01
9.0
8.5
8.0
7.5
7.0
6.5
6.0
5.5
5.0
4.5
4.0
3.5
3.0
2.5
2.0
1.5
1.0
0.5
0.0
f1 (ppm)

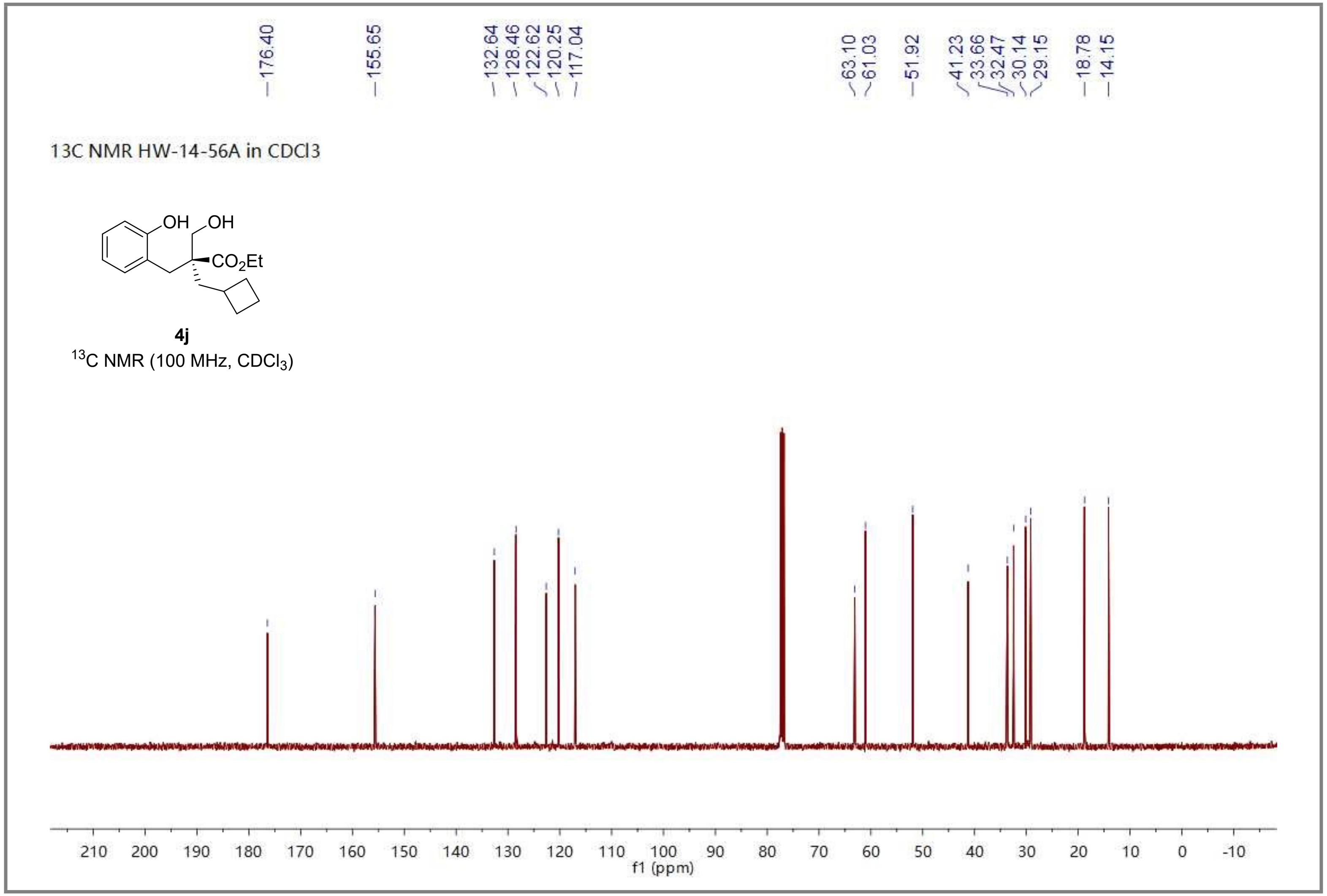
176.40
155.65
132.64
128.46
122.62
120.25
117.04
63.10
61.03
51.92
41.23
33.66
32.47
30.14
29.15
18.78
14.15
13C NMR HW-14-56A in CDCl3
OH
OH
CO2Et
4j
13C NMR (100 MHz, CDCl3)
210
200
190
180
170
160
150
140
130
120
110
100
90
80
70
60
50
40
30
20
10
0
-10
f1 (ppm)

Data File C:\CHEM32\1\DATA\ZHOU-23\YZN022979.D
Sample Name: HW-14-41A TM+/-

===

Acq. Operator :
Acq. Instrument : Instrument 1 Location : -
Injection Date : 4/26/2023 8:23:05 AM
Acq. Method : C:\CHEM32\1\METHODS\DEF_LC.M
Last changed : 4/26/2023 8:22:04 AM
(modified after loading)
Analysis Method : C:\CHEM32\1\METHODS\DEF_LC.M
Last changed : 10/17/2023 9:42:07 AM
(modified after loading)
Sample Info : IB, Hexane/i-PrOH = 95/5, 0.8 mL/min, 30 oC, 230 nm

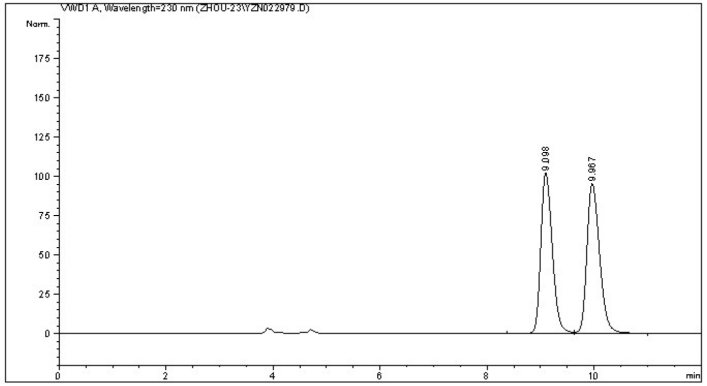

===
Area Percent Report
===

Sorted By : Signal
Multiplier: : 1.0000
Dilution: : 1.0000
Use Multiplier & Dilution Factor with ISTDs

Signal 1: VWD1 A, Wavelength=230 nm

Peak #	RetTime [min]	Type	Width [min]	Area mAU *s	Height [mAU]	Area %
1	9.098	VV	0.2332	1528.92163	101.94090	49.2315
2	9.967	VV	0.2573	1576.65430	95.21244	50.7685

Totals : 3105.57593 197.15334

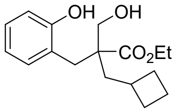

(+/-)-4j

===
*** End of Report ***

Instrument 1 10/17/2023 9:42:09 AM Page 1 of 1

Data File C:\CHEM32\1\DATA\ZHOU-23\YZN023148.D
Sample Name: HW-14-56A TM

===

Acq. Operator :
Acq. Instrument : Instrument 1 Location : -
Injection Date : 5/21/2023 2:23:55 AM
Acq. Method : C:\CHEM32\1\METHODS\DEF_LC.M
Last changed : 5/21/2023 2:15:33 AM
(modified after loading)
Analysis Method : C:\CHEM32\1\METHODS\DEF_LC.M
Last changed : 10/17/2023 9:43:13 AM
(modified after loading)
Sample Info : IB, Hexane/i-PrOH = 95/5, 0.8 mL/min, 30 oC, 230 nm

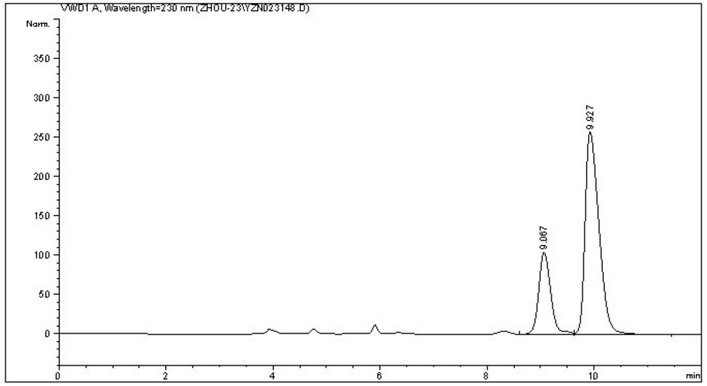

===
Area Percent Report
===

Sorted By : Signal
Multiplier: : 1.0000
Dilution: : 1.0000
Use Multiplier & Dilution Factor with ISTDs

Signal 1: VWD1 A, Wavelength=230 nm

Peak #	RetTime [min]	Type	Width [min]	Area mAU *s	Height [mAU]	Area %
1	9.067	VV	0.2464	1627.96777	104.17851	26.0059
2	9.927	VV	0.2798	4632.03223	257.48196	73.9941

Totals : 6260.00000 361.66048

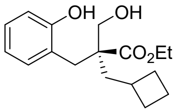

(+)-4j

===
*** End of Report ***

Instrument 1 10/17/2023 9:43:17 AM Page 1 of 1

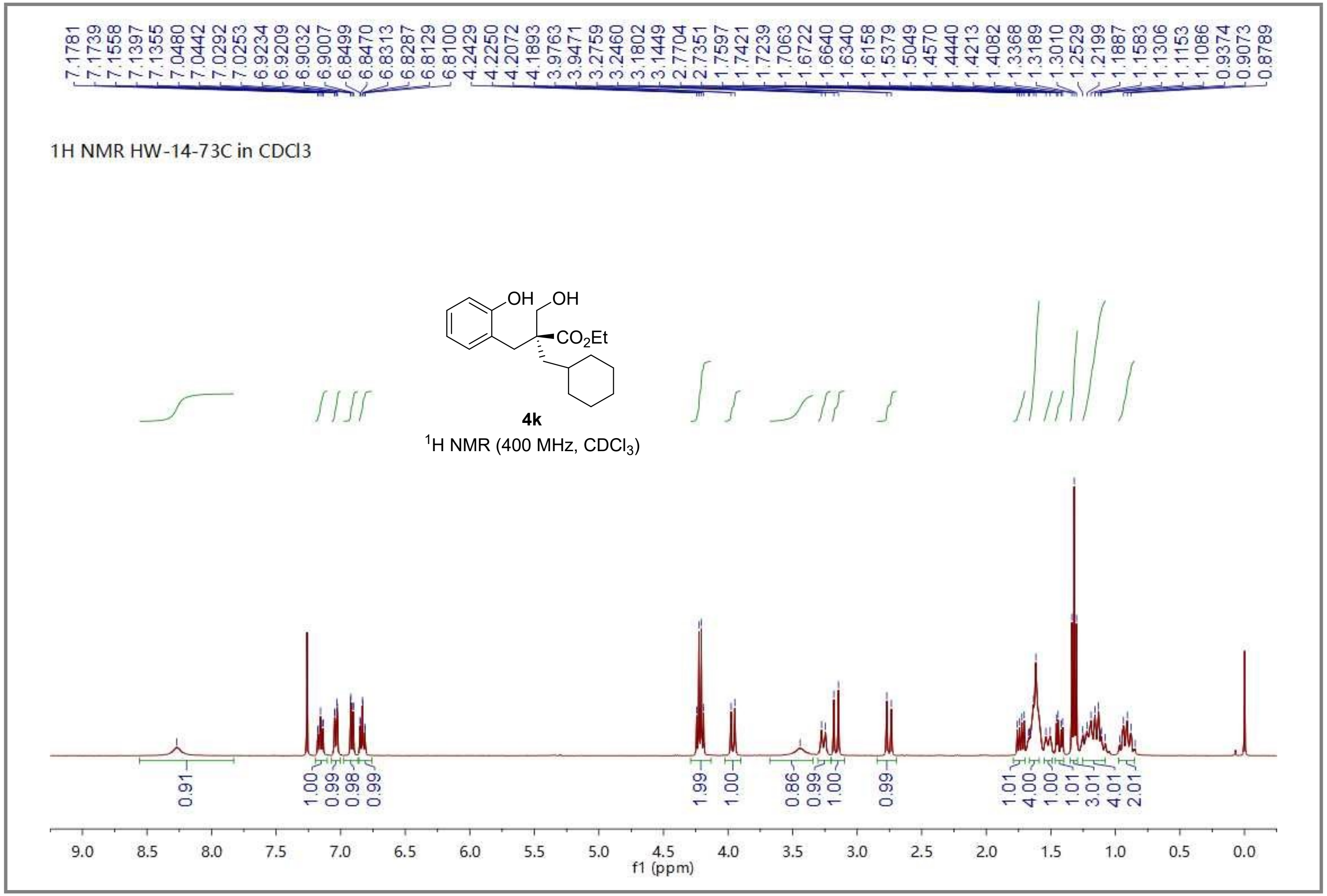
1H NMR HW-14-73C in CDCl3
4k
^{1}H NMR (400 MHz, $CDCl_3$)
f1 (ppm)

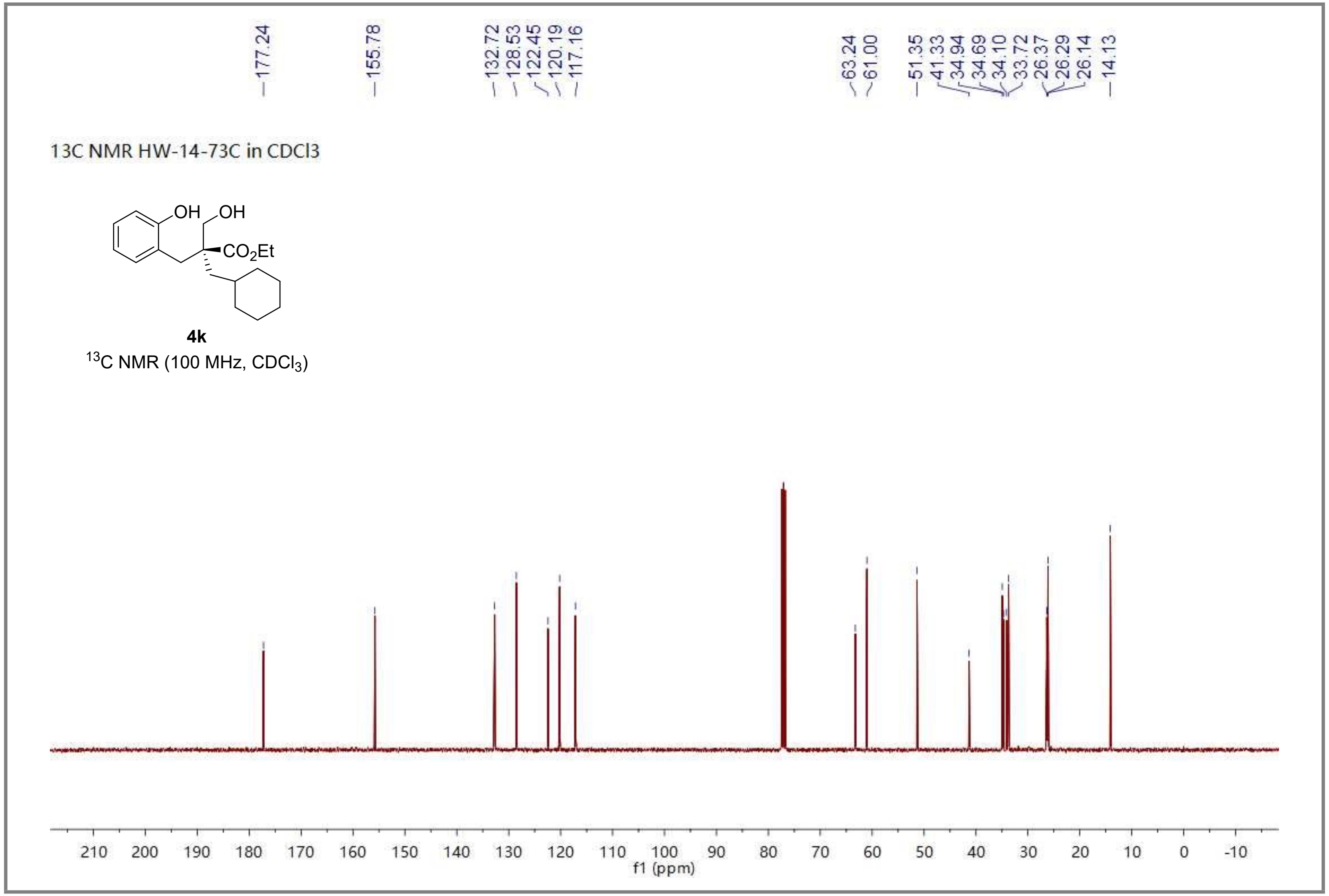
177.24
155.78
132.72
128.53
122.45
120.19
117.16
63.24
61.00
51.35
41.33
34.94
34.69
34.10
33.72
26.37
26.29
26.14
14.13
13C NMR HW-14-73C in CDCl3
OH
OH
CO2Et
4k
13C NMR (100 MHz, CDCl3)
210
200
190
180
170
160
150
140
130
120
110
100
90
80
70
60
50
40
30
20
10
0
-10
f1 (ppm)

Data File C:\CHEM32\1\DATA\ZHOU-23\YZN023156.D
Sample Name: HW-14-56B TM+/-

===

Acq. Operator :
Acq. Instrument : Instrument 1 Location : -
Injection Date : 5/22/2023 10:18:38 PM
Acq. Method : C:\CHEM32\1\METHODS\DEF_LC.M
Last changed : 5/22/2023 10:17:24 PM
(modified after loading)
Analysis Method : C:\CHEM32\1\METHODS\DEF_LC.M
Last changed : 10/17/2023 9:46:16 AM
(modified after loading)
Sample Info : AS-H, Hexane/i-PrOH = 90/10, 0.8 mL/min, 30 oC, 220 nm

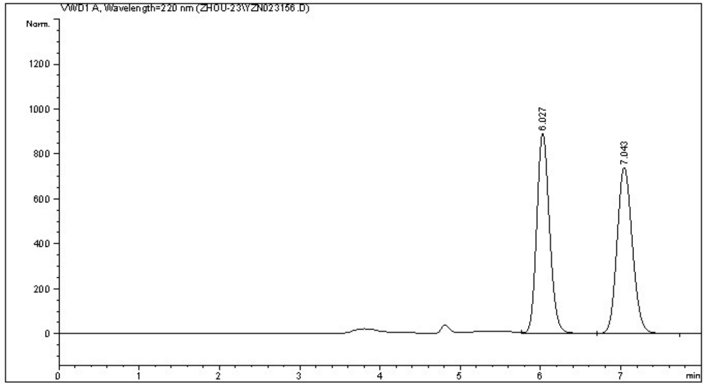

===
Area Percent Report
===

Sorted By : Signal
Multiplier: : 1.0000
Dilution: : 1.0000
Use Multiplier & Dilution Factor with ISTDs

Signal 1: VWD1 A, Wavelength=220 nm

Peak #	RetTime [min]	Type	Width [min]	Area mAU *s	Height [mAU]	Area %
1	6.027	VV	0.1705	9900.32520	890.14624	50.2350
2	7.043	VV	0.2074	9807.71289	738.23425	49.7650

Totals : 1.97080e4 1628.38049

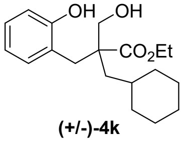

(+/-)-4k

===
*** End of Report ***

Instrument 1 10/17/2023 9:46:18 AM Page 1 of 1

Data File C:\CHEM32\1\DATA\ZHOU-23\YZN023225.D
Sample Name: HW-14-73C TM

===

Acq. Operator :
Acq. Instrument : Instrument 1 Location : -
Injection Date : 5/31/2023 2:12:13 AM
Acq. Method : C:\CHEM32\1\METHODS\DEF_LC.M
Last changed : 5/31/2023 2:11:14 AM
(modified after loading)
Analysis Method : C:\CHEM32\1\METHODS\DEF_LC.M
Last changed : 10/17/2023 9:47:08 AM
(modified after loading)
Sample Info : AS-H, Hexane/i-PrOH = 90/10, 0.8 mL/min, 30 oC, 230 nm

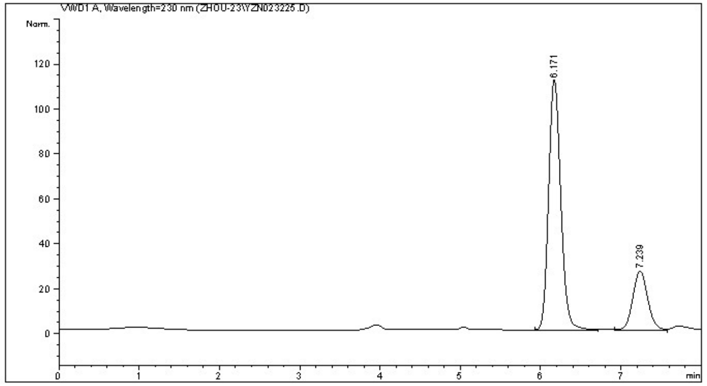

===
Area Percent Report
===

Sorted By : Signal
Multiplier: : 1.0000
Dilution: : 1.0000
Use Multiplier & Dilution Factor with ISTDs

Signal 1: VWD1 A, Wavelength=230 nm

Peak #	RetTime [min]	Type	Width [min]	Area mAU *s	Height [mAU]	Area %
1	6.171	BV	0.1701	1174.89648	110.97256	77.5276
2	7.239	VV	0.1976	340.55948	26.31386	22.4724

Totals : 1515.45596 137.28643

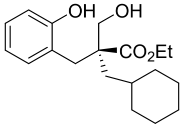

(+)-4k

===
*** End of Report ***

Instrument 1 10/17/2023 9:47:11 AM Page 1 of 1

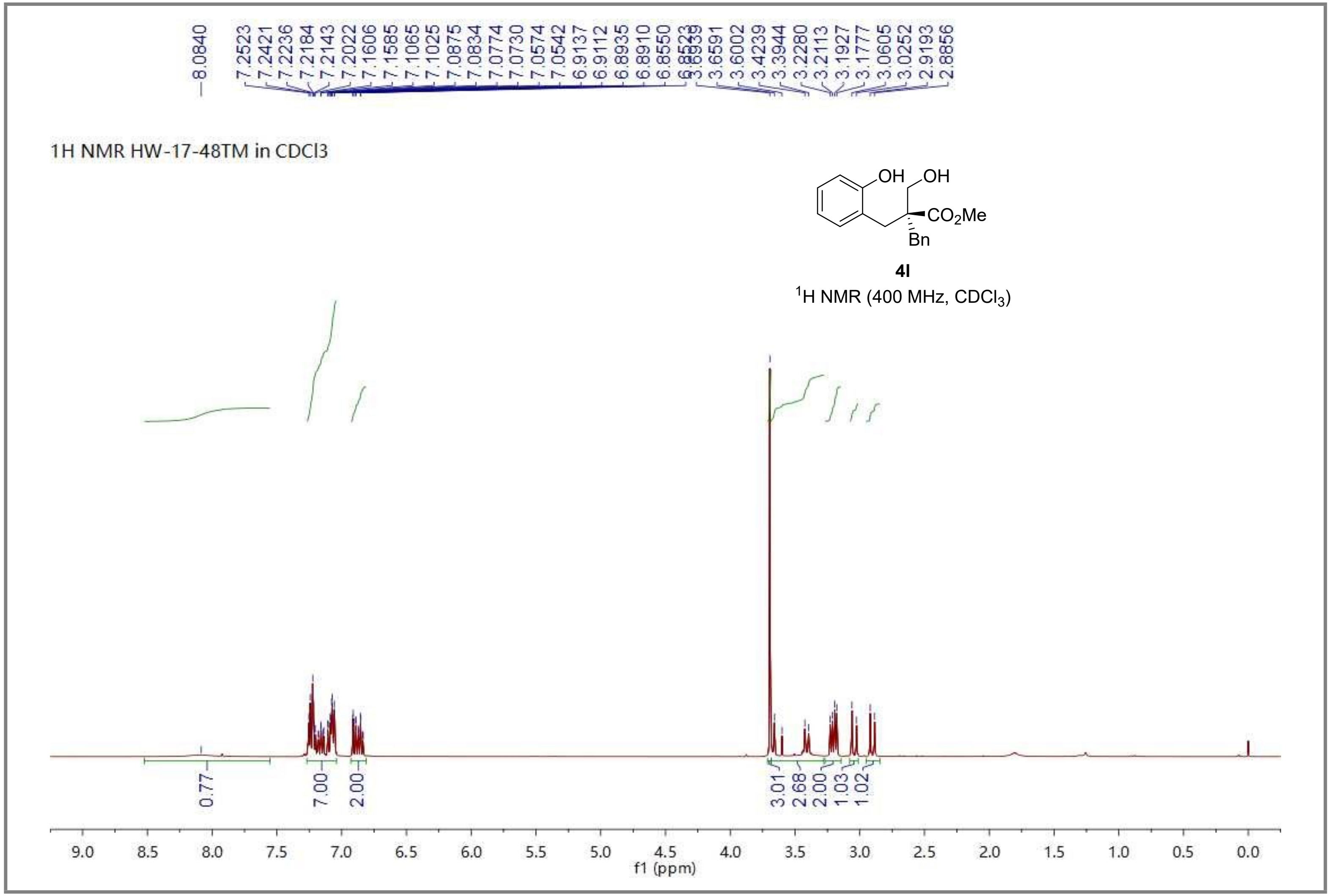

8.0840
7.2523
7.2421
7.2236
7.2184
7.2143
7.2022
7.1606
7.1585
7.1065
7.1025
7.0875
7.0834
7.0774
7.0730
7.0574
7.0542
6.9137
6.9112
6.8935
6.8910
6.8550
3.6591
3.6002
3.4239
3.3944
3.2280
3.2113
3.1927
3.1777
3.0605
3.0252
2.9193
2.8856
1H NMR HW-17-48TM in CDCl3
OH
OH
CO2Me
Bn
4l
1H NMR (400 MHz, CDCl3)
0.77
7.00
2.00
3.01
2.68
2.00
1.03
1.02
9.0
8.5
8.0
7.5
7.0
6.5
6.0
5.5
5.0
4.5
4.0
3.5
3.0
2.5
2.0
1.5
1.0
0.5
0.0
f1 (ppm)

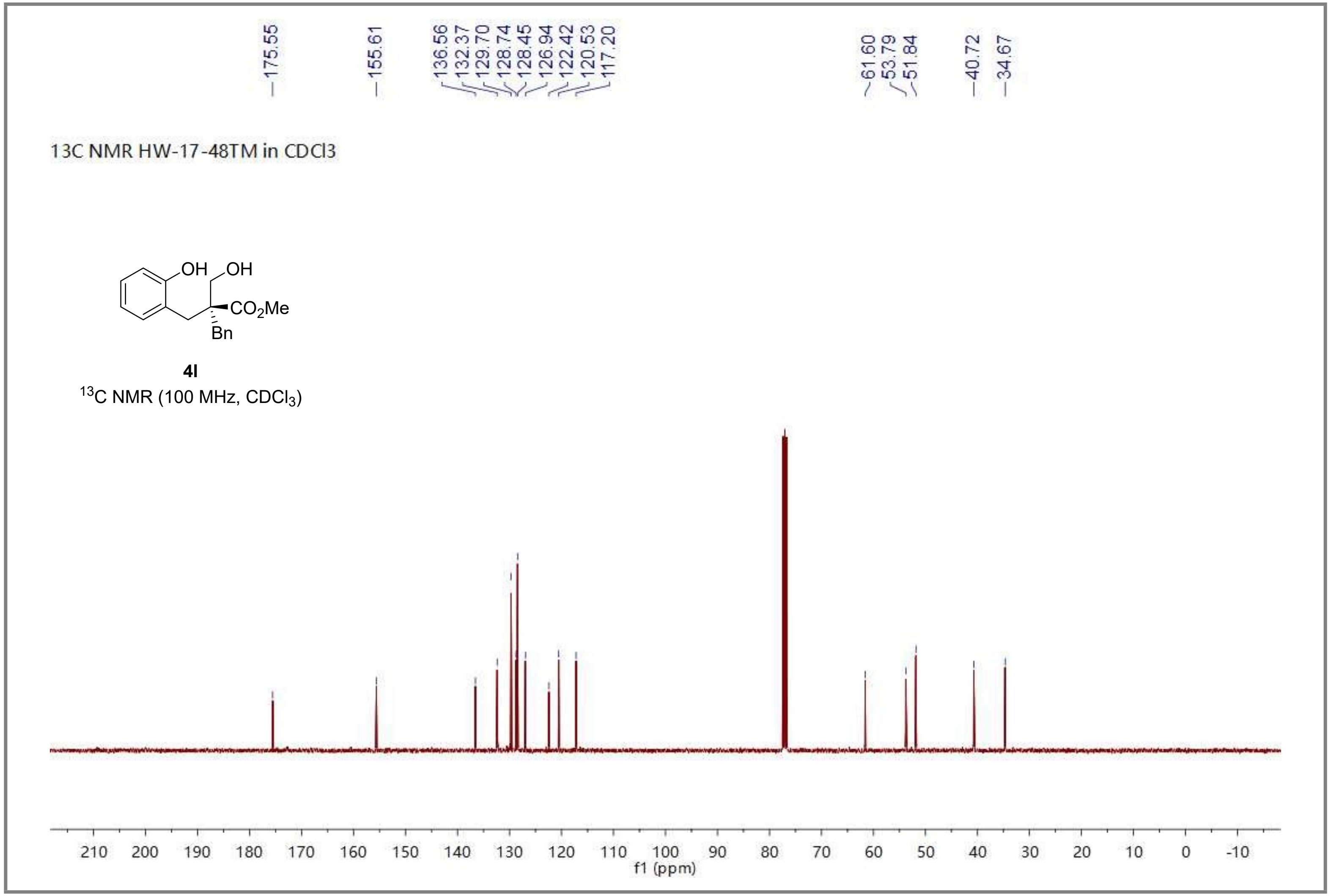
175.55
155.61
136.56
132.37
129.70
128.74
128.45
126.94
122.42
120.53
117.20
61.60
53.79
51.84
40.72
34.67
13C NMR HW-17-48TM in CDCl3
OH
OH
CO2Me
Bn
4l
13C NMR (100 MHz, CDCl3)
210 200 190 180 170 160 150 140 130 120 110 100 90 80 70 60 50 40 30 20 10 0 -10
f1 (ppm)

Data File D:\USERS\CHEMSTATION\1\DATA\ZHOU2024\YZ3009335 08-23-31.D
Sample Name: HW-17-48TM+/-

===

Acq. Operator : SYSTEM
Sample Operator : SYSTEM
Acq. Instrument : 1260II Location : -
Injection Date : 4/28/2024 8:23:31 AM Inj : 1
Inj Volume : No inj
Acq. Method : C:\Users\Public\Documents\ChemStation\1\Methods\def_LC.M
Last changed : 4/28/2024 8:08:31 AM by SYSTEM
(modified after loading)
Analysis Method : C:\Users\Public\Documents\ChemStation\1\Methods\def_LC.M
Last changed : 4/28/2024 10:00:18 AM by SYSTEM
(modified after loading)
Sample Info : IB, n-Hexane/i-PrOH = 97/3, 1.0 mL/min, 30 oC, 230 nm

Additional Info : Peak(s) manually integrated

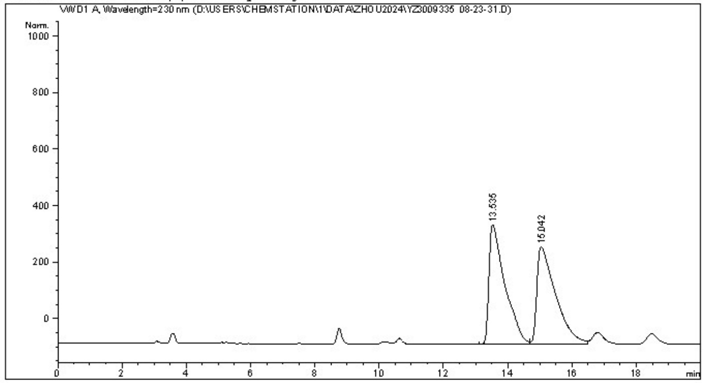

===
Area Percent Report
===

Sorted By : Signal
Multiplier : 1.0000
Dilution : 1.0000
Do not use Multiplier & Dilution Factor with ISTDs

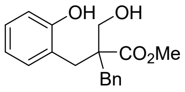

(+/-)-4l

Signal 1: VWD1 A, Wavelength=230 nm

Peak #	RetTime [min]	Type	Width [min]	Area [mAU*s]	Height [mAU]	Area %
1	13.535	BV	0.5013	8433.72168	245.18311	51.0353
2	15.042	VV	0.5842	8091.54639	199.93265	48.9647

1260II 4/28/2024 10:00:20 AM SYSTEM Page 1 of 2

Data File D:\USERS\CHEMSTATION\1\DATA\ZHOU2024\YZ3009336 08-47-37.D
Sample Name: HW-17-48TM

===

Acq. Operator : SYSTEM
Sample Operator : SYSTEM
Acq. Instrument : 1260II Location : -
Injection Date : 4/28/2024 8:47:37 AM Inj : 1
Inj Volume : No inj
Acq. Method : C:\Users\Public\Documents\ChemStation\1\Methods\def_LC.M
Last changed : 4/28/2024 8:08:31 AM by SYSTEM
(modified after loading)
Analysis Method : C:\Users\Public\Documents\ChemStation\1\Methods\def_LC.M
Last changed : 4/28/2024 9:57:35 AM by SYSTEM
(modified after loading)
Sample Info : IB, n-Hexane/i-PrOH = 97/3, 1.0 mL/min, 30 oC, 230 nm

Additional Info : Peak(s) manually integrated

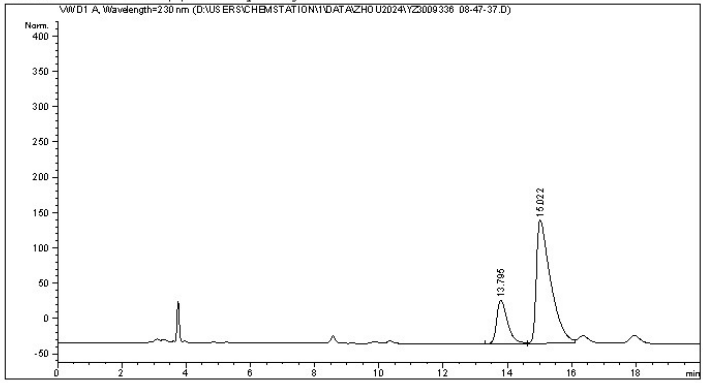

===
Area Percent Report
===

Sorted By : Signal
Multiplier : 1.0000
Dilution : 1.0000
Do not use Multiplier & Dilution Factor with ISTDs

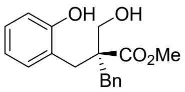

(+)-4l

Signal 1: VWD1 A, Wavelength=230 nm

Peak #	RetTime [min]	Type	Width [min]	Area [mAU*s]	Height [mAU]	Area %
1	13.795	BV	0.3533	844.73486	36.59961	20.7135
2	15.022	VV	0.4490	3233.44556	104.50420	79.2865

1260II 4/28/2024 9:58:13 AM SYSTEM Page 1 of 2

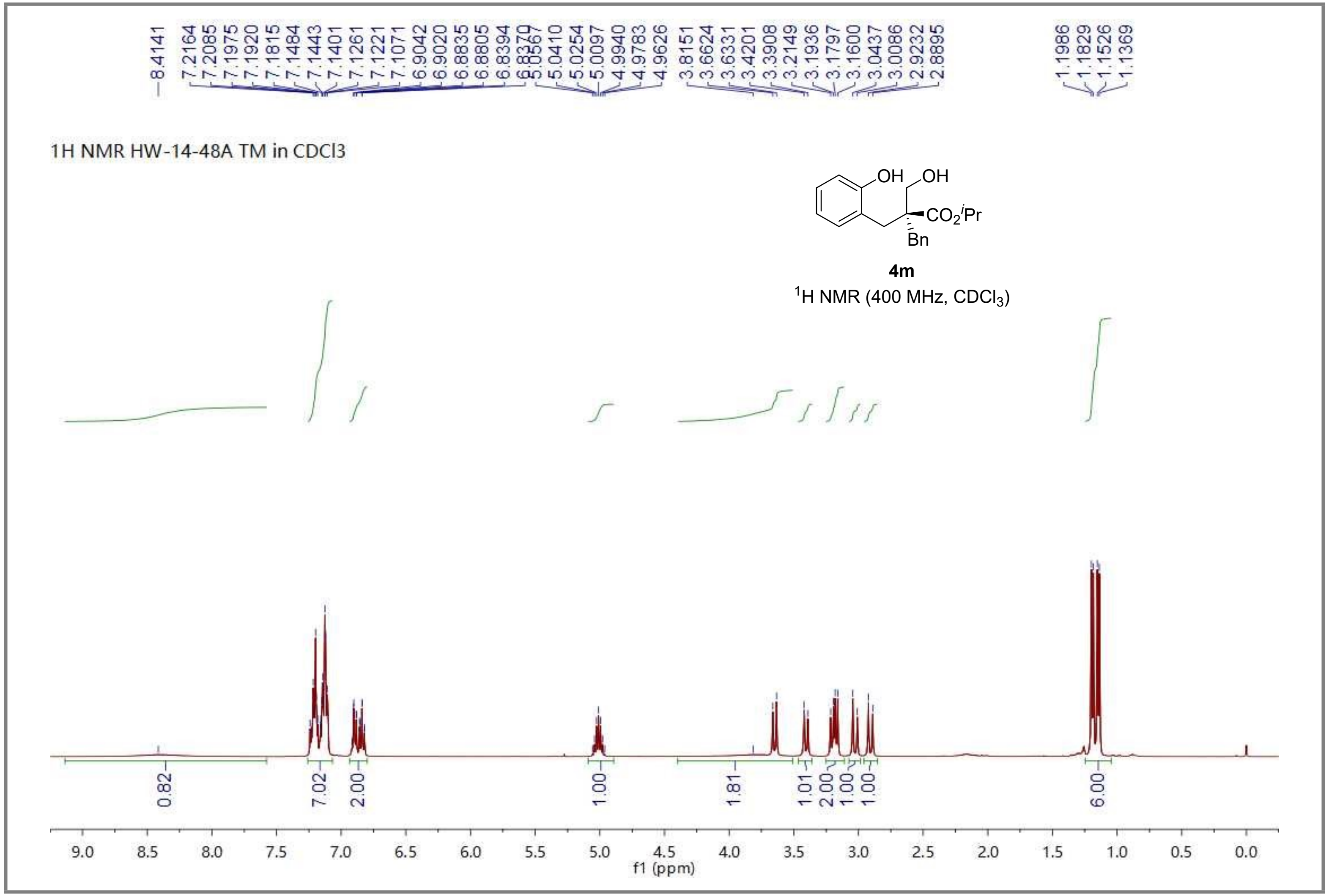

8.4141
7.2164
7.2085
7.1975
7.1920
7.1815
7.1484
7.1443
7.1401
7.1261
7.1221
7.1071
6.9042
6.9020
6.8835
6.8805
6.8394
6.8370
5.0567
5.0410
5.0254
5.0097
4.9940
4.9783
4.9626
3.8151
3.6624
3.6331
3.4201
3.3908
3.2149
3.1936
3.1797
3.1600
3.0437
3.0086
2.9232
2.8895
1.1986
1.1829
1.1526
1.1369
1H NMR HW-14-48A TM in CDCl3
OH
OH
CO2iPr
Bn
4m
1H NMR (400 MHz, CDCl3)
0.82
7.02
2.00
1.00
1.81
1.01
2.00
1.00
1.00
6.00
f1 (ppm)

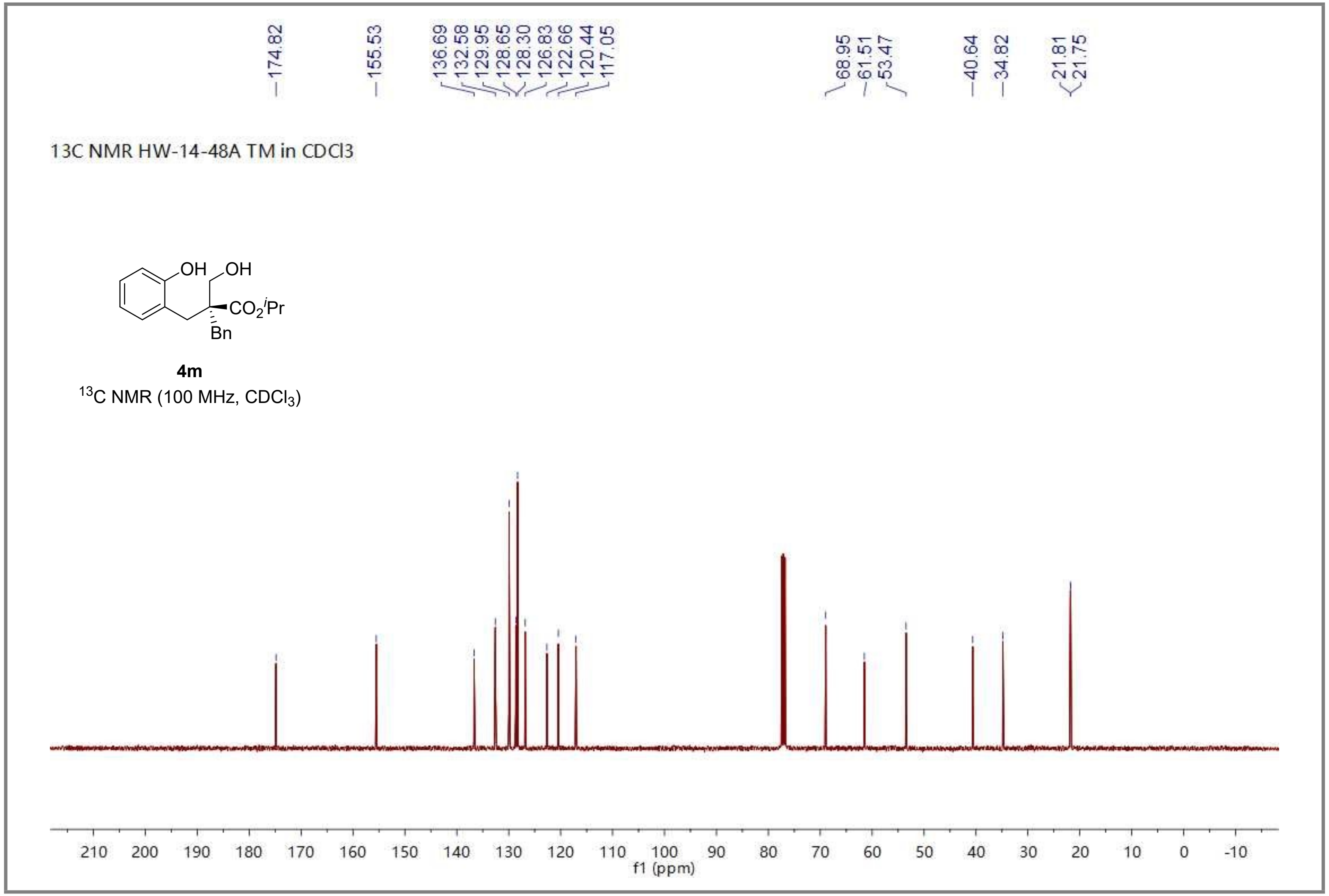
174.82
155.53
136.69
132.58
129.95
128.65
128.30
126.83
122.66
120.44
117.05
68.95
61.51
53.47
40.64
34.82
21.81
21.75
13C NMR HW-14-48A TM in CDCl3
OH
OH
$CO_2{}^iPr$
Bn
4m
^{13}C NMR (100 MHz, $CDCl_3$)
f1 (ppm)

Data File C:\CHEM32\1\DATA\ZHOU-23\YZN023017.D
Sample Name: HW-14-48A TM+/-

===

Acq. Operator :
Acq. Instrument : Instrument 1 Location : -
Injection Date : 5/5/2023 6:35:45 AM
Acq. Method : C:\CHEM32\1\METHODS\DEF_LC.M
Last changed : 5/5/2023 6:35:18 AM
(modified after loading)
Analysis Method : C:\CHEM32\1\METHODS\DEF_LC.M
Last changed : 10/17/2023 9:50:10 AM
(modified after loading)
Sample Info : IB, Hexane/i-PrOH = 95/5, 0.8 mL/min, 30 oC, 230nm

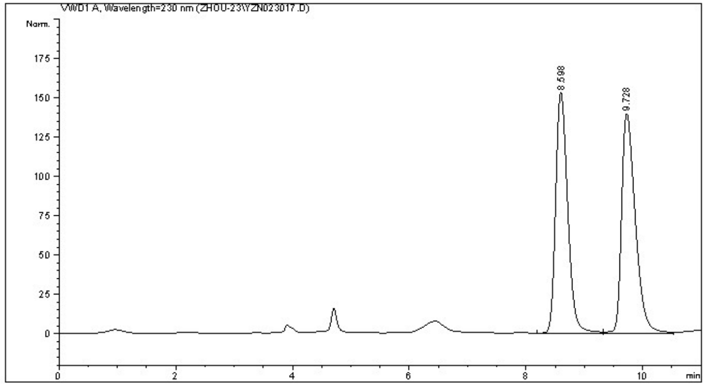

===
Area Percent Report
===

Sorted By : Signal
Multiplier: : 1.0000
Dilution: : 1.0000
Use Multiplier & Dilution Factor with ISTDs

Signal 1: VWD1 A, Wavelength=230 nm

Peak #	RetTime [min]	Type	Width [min]	Area mAU *s	Height [mAU]	Area %
1	8.598	VV	0.2257	2197.08643	153.03523	49.0022
2	9.728	VV	0.2555	2286.56616	139.36580	50.9978
Totals :				4483.65259	292.40103	

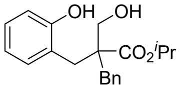

(+/-)-4m

===
*** End of Report ***

Instrument 1 10/17/2023 9:50:13 AM Page 1 of 1

Data File C:\CHEM32\1\DATA\ZHOU-23\YZN023018.D
Sample Name: HW-14-48A TM

===

Acq. Operator :
Acq. Instrument : Instrument 1 Location : -
Injection Date : 5/5/2023 6:50:22 AM
Acq. Method : C:\CHEM32\1\METHODS\DEF_LC.M
Last changed : 5/5/2023 6:49:58 AM
(modified after loading)
Analysis Method : C:\CHEM32\1\METHODS\DEF_LC.M
Last changed : 10/17/2023 9:51:19 AM
(modified after loading)
Sample Info : IB, Hexane/i-PrOH = 95/5, 0.8 mL/min, 30 oC, 230nm

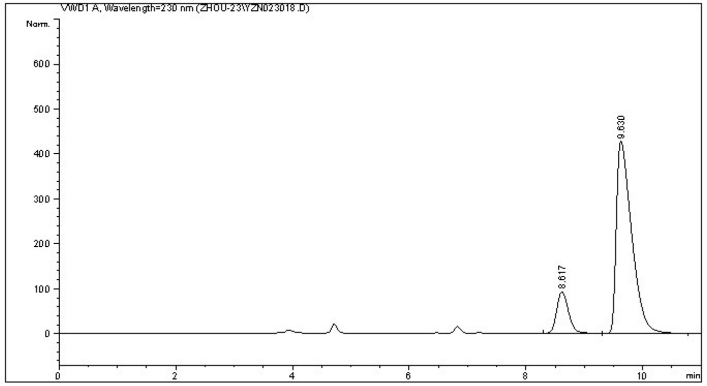

===
Area Percent Report
===

Sorted By : Signal
Multiplier: : 1.0000
Dilution: : 1.0000
Use Multiplier & Dilution Factor with ISTDs

Signal 1: VWD1 A, Wavelength=230 nm

Peak #	RetTime [min]	Type	Width [min]	Area mAU *s	Height [mAU]	Area %
1	8.617	VV	0.2231	1309.10754	92.63066	14.4020
2	9.630	VV	0.2769	7780.67188	426.52310	85.5980
Totals :				9089.77942	519.15376	

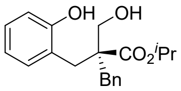

(+)-4m

===
*** End of Report ***

Instrument 1 10/17/2023 9:51:21 AM Page 1 of 1

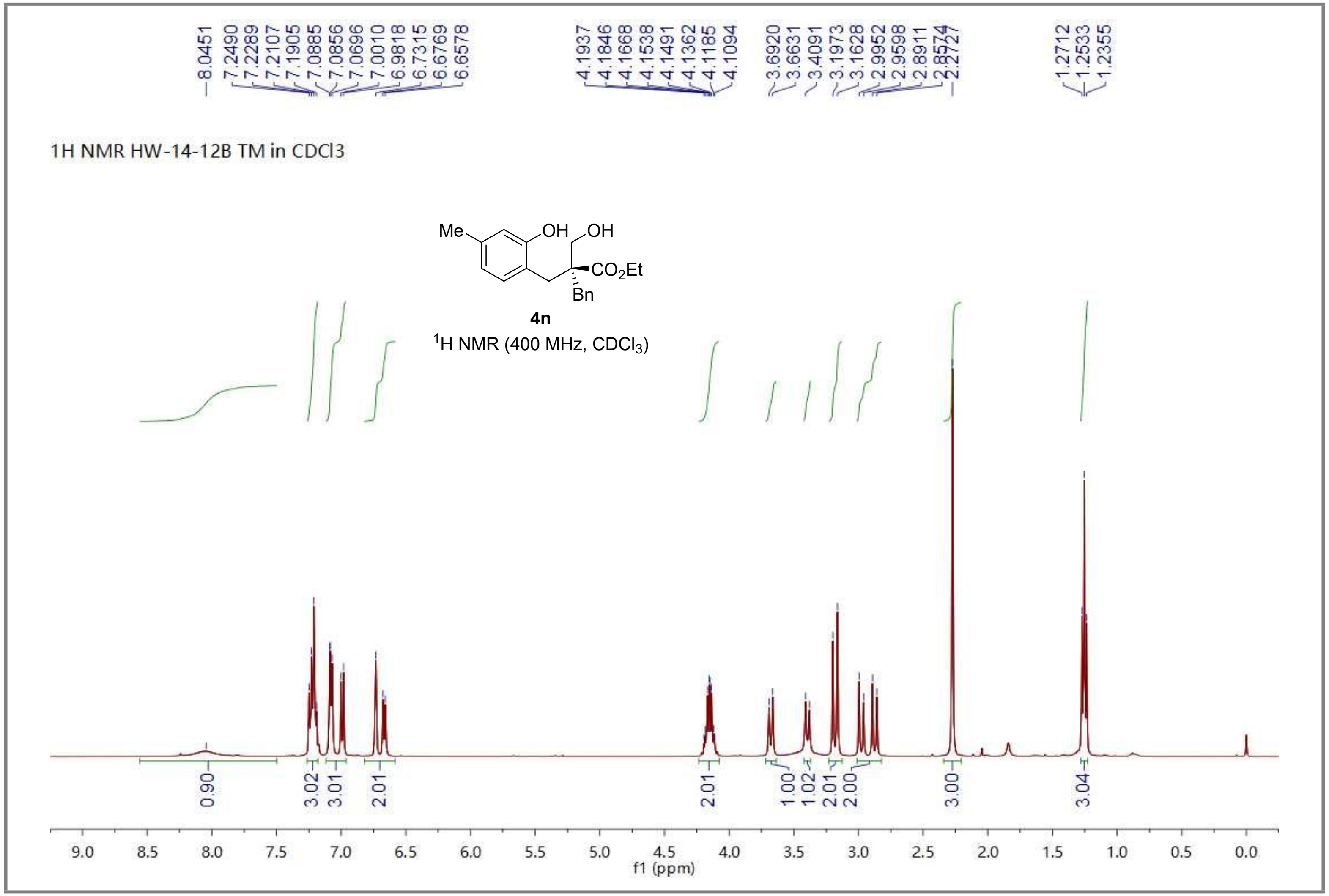
1H NMR HW-14-12B TM in CDCl3
Me
OH
OH
CO2Et
Bn
4n
1H NMR (400 MHz, CDCl3)
8.0451
7.2490
7.2289
7.2107
7.1905
7.0885
7.0856
7.0696
7.0010
6.9818
6.7315
6.6769
6.6578
4.1937
4.1846
4.1668
4.1538
4.1491
4.1362
4.1185
4.1094
3.6920
3.6631
3.4091
3.1973
3.1628
2.9952
2.9598
2.8911
2.8574
2.2727
1.2712
1.2533
1.2355
0.90
3.02
3.01
2.01
2.01
1.00
1.02
2.01
2.00
3.00
3.04
f1 (ppm)

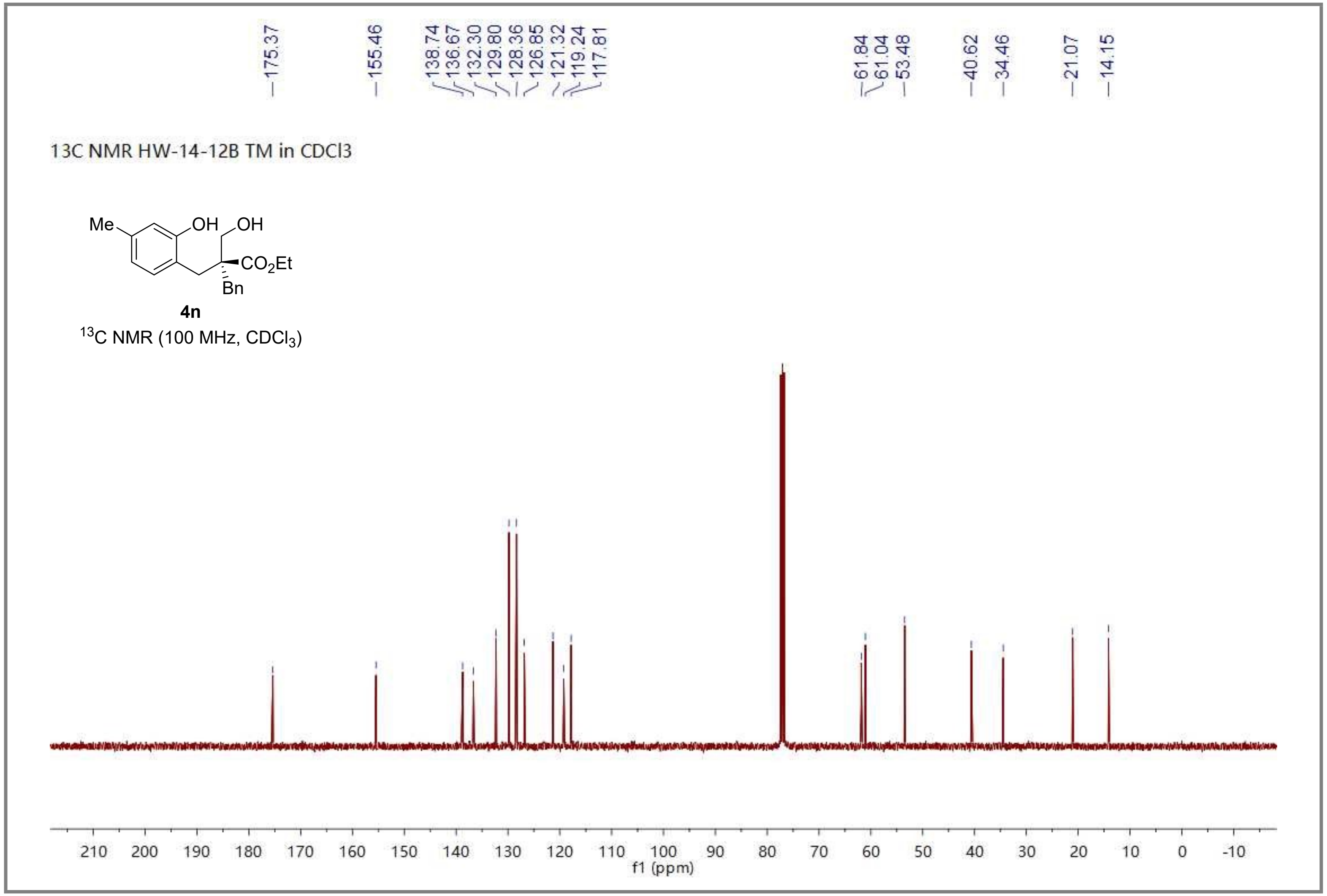
175.37
155.46
138.74
136.67
132.30
129.80
128.36
126.85
121.32
119.24
117.81
61.84
61.04
53.48
40.62
34.46
21.07
14.15
13C NMR HW-14-12B TM in CDCl3
Me
OH
OH
CO2Et
Bn
4n
13C NMR (100 MHz, CDCl3)
210 200 190 180 170 160 150 140 130 120 110 100 90 80 70 60 50 40 30 20 10 0 -10
f1 (ppm)

Data File C:\CHEM32\1\DATA\ZHOU-23\YZN024701.D
Sample Name: HW-14-12BTM+/-

===

Acq. Operator :
Acq. Instrument : Instrument 1 Location : -
Injection Date : 12/1/2023 2:40:00 AM
Acq. Method : C:\CHEM32\1\METHODS\DEF_LC.M
Last changed : 12/1/2023 2:31:04 AM
(modified after loading)
Analysis Method : C:\CHEM32\1\METHODS\DEF_LC.M
Last changed : 12/1/2023 3:30:06 AM
(modified after loading)
Sample Info : IA, Hexane/iPrOH = 95/5, 0.8 mL/min, 30 oC, 230 nm

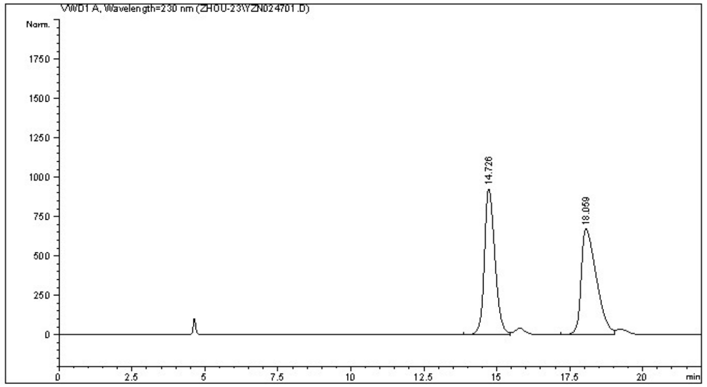

===

Area Percent Report

===

Sorted By : Signal
Multiplier: : 1.0000
Dilution: : 1.0000
Use Multiplier & Dilution Factor with ISTDs

Signal 1: VWD1 A, Wavelength=230 nm

Peak #	RetTime [min]	Type	Width [min]	Area mAU *s	Height [mAU]	Area %
1	14.726	BV	0.3848	2.32406e4	929.63464	48.8079
2	18.059	BV	0.5630	2.43758e4	671.90833	51.1921

Totals : 4.76164e4 1601.54297

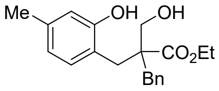

(+/-)-4n

===

*** End of Report ***

Instrument 1 12/1/2023 3:30:15 AM Page 1 of 1

Data File C:\CHEM32\1\DATA\ZHOU-23\YZN024702.D
Sample Name: HW-14-12BTM

===

Acq. Operator :
Acq. Instrument : Instrument 1 Location : -
Injection Date : 12/1/2023 3:04:06 AM
Acq. Method : C:\CHEM32\1\METHODS\DEF_LC.M
Last changed : 12/1/2023 3:03:19 AM
(modified after loading)
Analysis Method : C:\CHEM32\1\METHODS\DEF_LC.M
Last changed : 12/1/2023 3:29:08 AM
(modified after loading)
Sample Info : IA, Hexane/iPrOH = 95/5, 0.8 mL/min, 30 oC, 230 nm

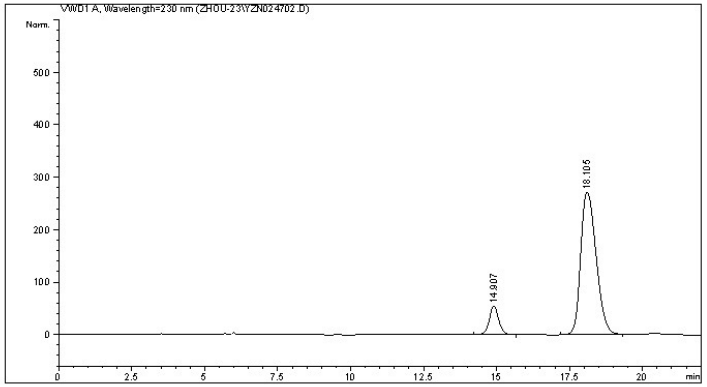

===

Area Percent Report

===

Sorted By : Signal
Multiplier: : 1.0000
Dilution: : 1.0000
Use Multiplier & Dilution Factor with ISTDs

Signal 1: VWD1 A, Wavelength=230 nm

Peak #	RetTime [min]	Type	Width [min]	Area mAU *s	Height [mAU]	Area %
1	14.907	BB	0.3381	1226.70667	55.15179	10.9720
2	18.105	BB	0.5808	9953.66699	271.37015	89.0280

Totals : 1.11804e4 326.52193

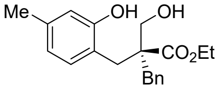

(+)-4n

===

*** End of Report ***

Instrument 1 12/1/2023 3:29:12 AM Page 1 of 1

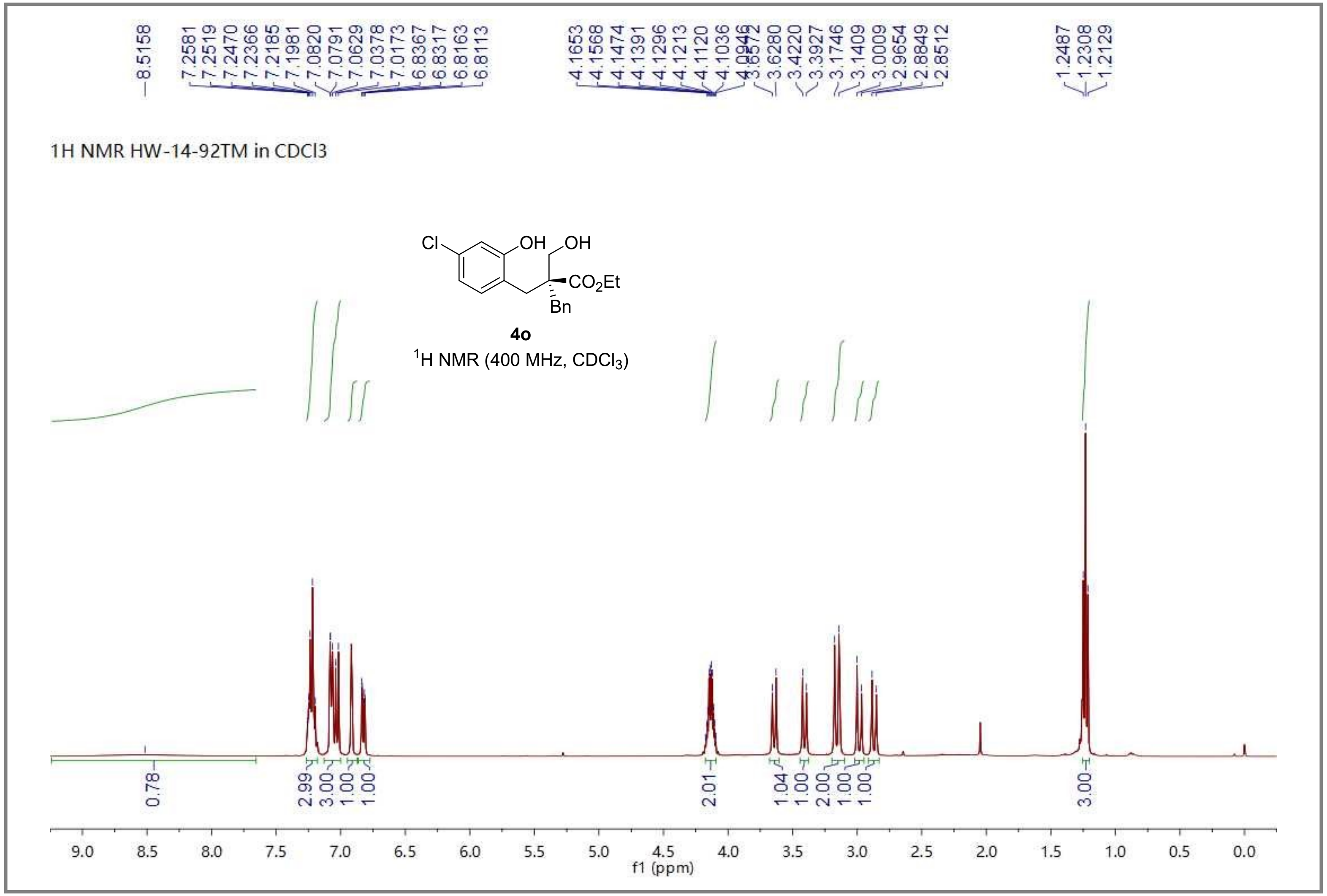
1H NMR HW-14-92TM in CDCl3
4o
^{1}H NMR (400 MHz, $CDCl_3$)
f1 (ppm)

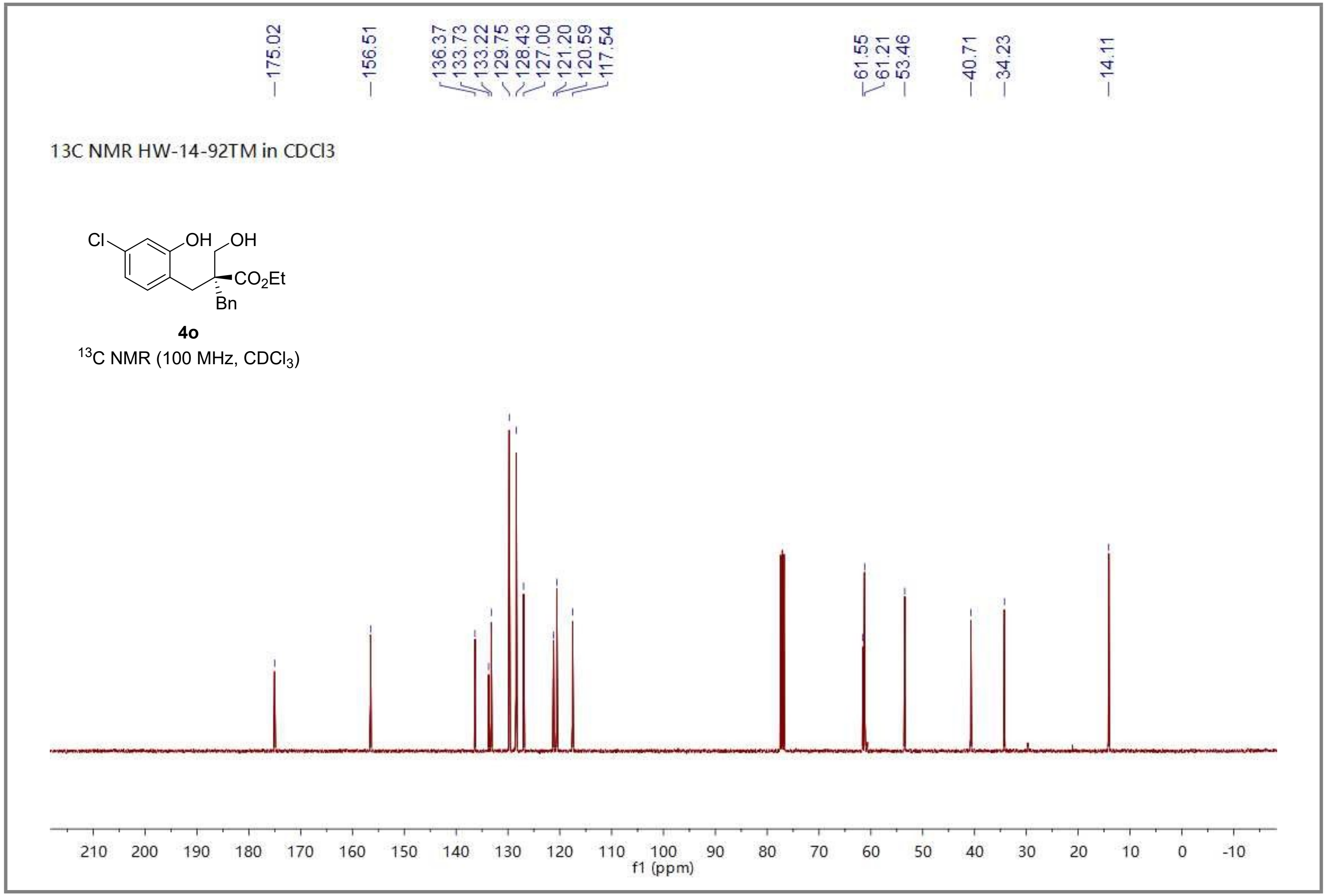

175.02
156.51
136.37
133.73
133.22
129.75
128.43
127.00
121.20
120.59
117.54
61.55
61.21
53.46
40.71
34.23
14.11
13C NMR HW-14-92TM in CDCl3
Cl
OH
OH
CO2Et
Bn
4o
13C NMR (100 MHz, CDCl3)
210
200
190
180
170
160
150
140
130
120
110
100
90
80
70
60
50
40
30
20
10
0
-10
f1 (ppm)

Data File C:\USERS\PUBLIC\DOCUMENTS\CHEMSTATION\1\DATA\2023\YZH005570 10-07-47.D
Sample Name: HW-14-92TM+/-

===

Acq. Operator : SYSTEM
Sample Operator : SYSTEM
Acq. Instrument : HPLC1260 II Location : 1
Injection Date : 11/29/2023 10:07:48 AM Inj : 1
Inj Volume : No inj
Acq. Method : C:\USERS\PUBLIC\DOCUMENTS\CHEMSTATION\1\METHODS\def_LC1.M
Last changed : 11/29/2023 10:07:11 AM by SYSTEM
(modified after loading)
Analysis Method : C:\USERS\PUBLIC\DOCUMENTS\CHEMSTATION\1\METHODS\def_LC1.M
Last changed : 11/29/2023 11:07:54 AM by SYSTEM
(modified after loading)
Sample Info : IB, Hexane/i-PrOH = 95/5, 0.8 mL/min, 30 oC, 230 nm

Additional Info : Peak(s) manually integrated

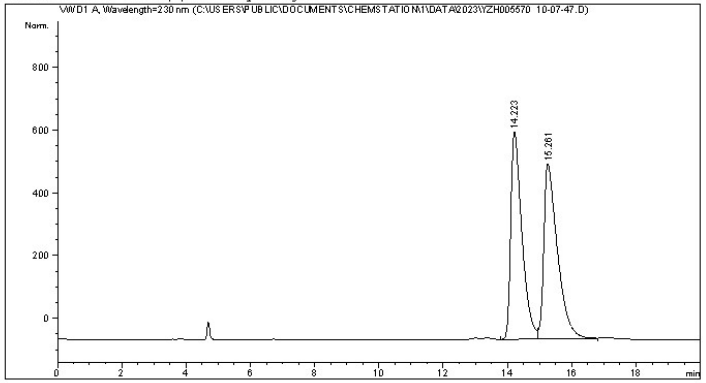

===
Area Percent Report
===

Sorted By : Signal
Multiplier : 1.0000
Dilution : 1.0000
Sample Amount: : 1.00000 [ng/ul] (not used in calc.)
Do not use Multiplier & Dilution Factor with ISTDs

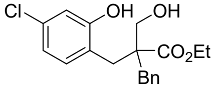

(+/-)-4o

Signal 1: VWD1 A, Wavelength=230 nm

Peak #	RetTime [min]	Type	Width [min]	Area [mAU*s]	Height [mAU]	Area %
1	14.223	BV	0.3595	1.27398e4	529.96002	49.1258
2	15.261	VB	0.4330	1.31932e4	447.39484	50.8742

HPLC1260 II 11/29/2023 11:07:58 AM SYSTEM Page 1 of 2

Data File C:\USERS\PUBLIC\DOCUMENTS\CHEMSTATION\1\DATA\2023\YZH005571 10-30-15.D
Sample Name: HW-14-92TM

===

Acq. Operator : SYSTEM
Sample Operator : SYSTEM
Acq. Instrument : HPLC1260 II Location : 1
Injection Date : 11/29/2023 10:30:15 AM Inj : 1
Inj Volume : No inj
Acq. Method : C:\USERS\PUBLIC\DOCUMENTS\CHEMSTATION\1\METHODS\def_LC1.M
Last changed : 11/29/2023 10:07:11 AM by SYSTEM
(modified after loading)
Analysis Method : C:\USERS\PUBLIC\DOCUMENTS\CHEMSTATION\1\METHODS\def_LC1.M
Last changed : 11/29/2023 11:08:45 AM by SYSTEM
(modified after loading)
Sample Info : IB, Hexane/i-PrOH = 95/5, 0.8 mL/min, 30 oC, 230 nm

Additional Info : Peak(s) manually integrated

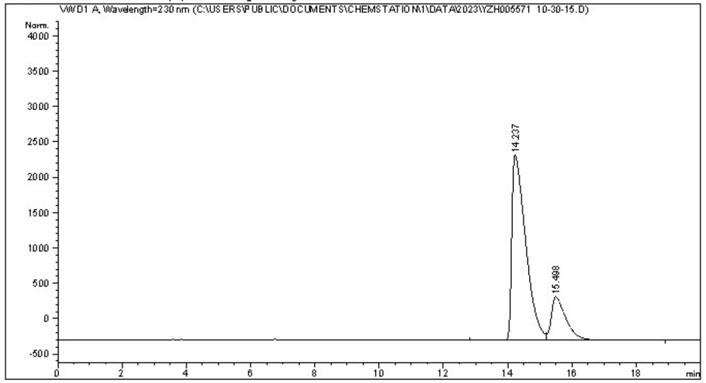

===
Area Percent Report
===

Sorted By : Signal
Multiplier : 1.0000
Dilution : 1.0000
Sample Amount: : 1.00000 [ng/ul] (not used in calc.)
Do not use Multiplier & Dilution Factor with ISTDs

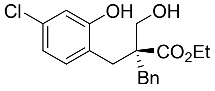

(+)-4o

Signal 1: VWD1 A, Wavelength=230 nm

Peak #	RetTime [min]	Type	Width [min]	Area [mAU*s]	Height [mAU]	Area %
1	14.237	VV R	0.4570	6.01549e4	2007.48206	79.6717
2	15.498	VV R	0.4721	1.53486e4	472.10394	20.3283

HPLC1260 II 11/29/2023 11:08:49 AM SYSTEM Page 1 of 2

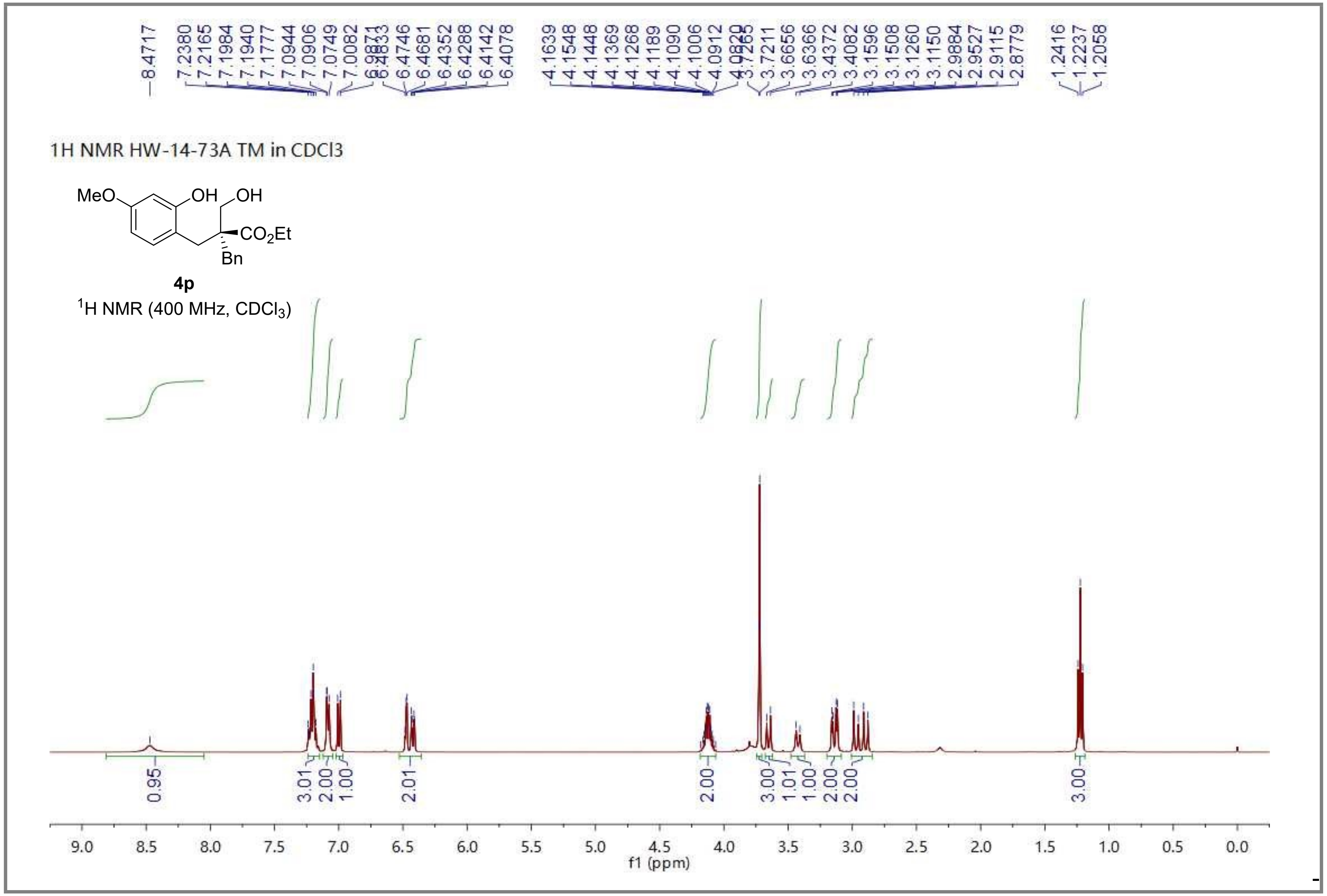
1H NMR HW-14-73A TM in CDCl3
MeO
OH
OH
CO2Et
Bn
4p
1H NMR (400 MHz, CDCl3)
8.4717
7.2380
7.2165
7.1984
7.1940
7.1777
7.0944
7.0906
7.0749
7.0082
6.9871
6.9833
6.4746
6.4681
6.4352
6.4288
6.4142
6.4078
4.1639
4.1548
4.1448
4.1369
4.1268
4.1189
4.1090
4.1006
4.0912
4.0820
3.7265
3.7211
3.6656
3.6366
3.4372
3.4082
3.1596
3.1508
3.1260
3.1150
2.9884
2.9527
2.9115
2.8779
1.2416
1.2237
1.2058
0.95
3.01
2.00
1.00
2.01
2.00
3.00
1.01
1.00
2.00
2.00
3.00
9.0
8.5
8.0
7.5
7.0
6.5
6.0
5.5
5.0
4.5
4.0
3.5
3.0
2.5
2.0
1.5
1.0
0.5
0.0
f1 (ppm)

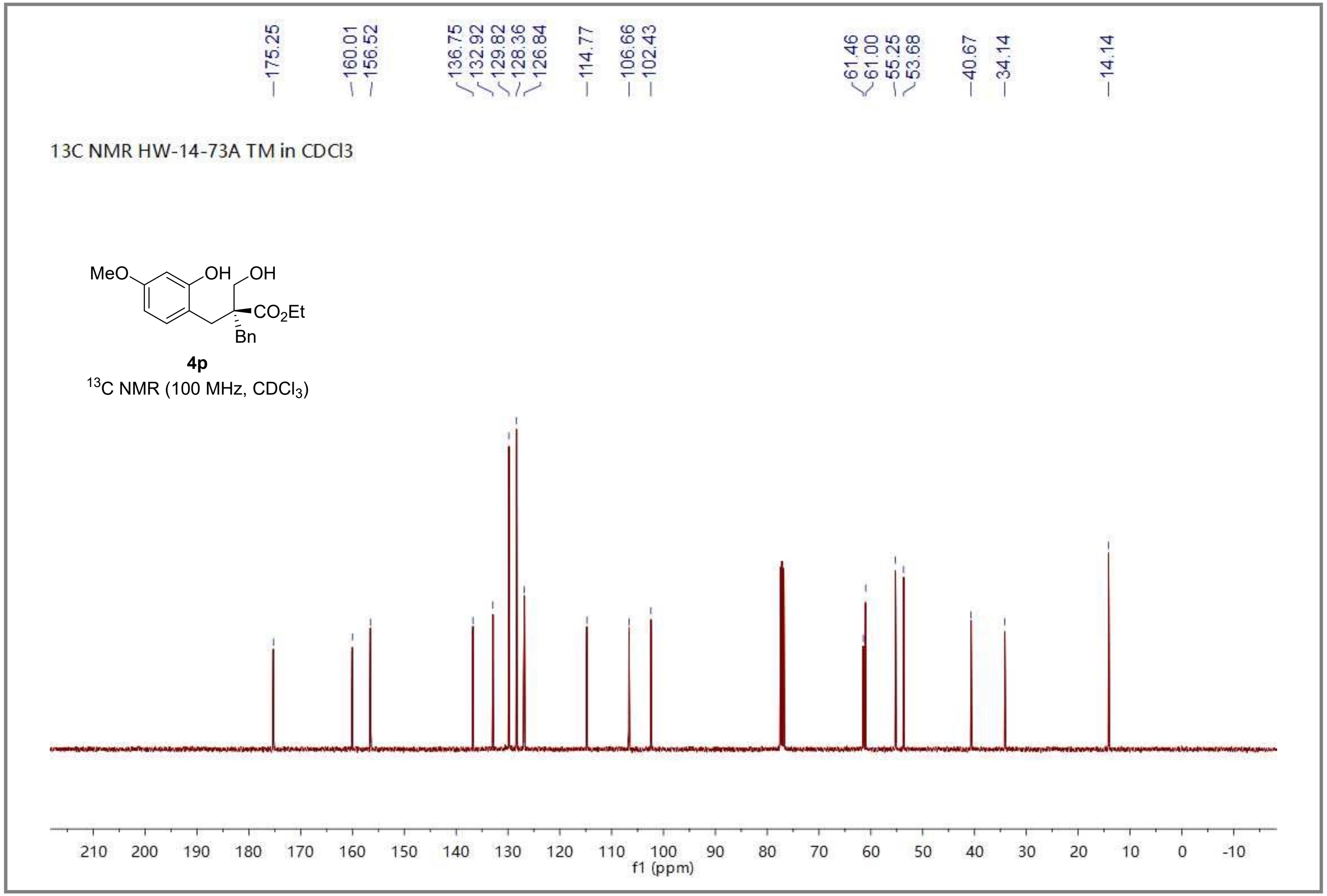
175.25
160.01
156.52
136.75
132.92
129.82
128.36
126.84
114.77
106.66
102.43
61.46
61.00
55.25
53.68
40.67
34.14
14.14
13C NMR HW-14-73A TM in CDCl3
MeO
OH
OH
CO_2Et
Bn
4p
^{13}C NMR (100 MHz, $CDCl_3$)
210 200 190 180 170 160 150 140 130 120 110 100 90 80 70 60 50 40 30 20 10 0 -10
f1 (ppm)

Data File C:\USERS\PUBLIC\DOCUMENTS\CHEMSTATION\1\DATA\2023\YZH004298 11-18-47.D
Sample Name: HW-14-73A TM+/-

===

Acq. Operator : SYSTEM
Sample Operator : SYSTEM
Acq. Instrument : HPLC1260 II Location : 1
Injection Date : 5/31/2023 11:18:47 AM Inj : 1
Inj Volume : No inj
Acq. Method : C:\USERS\PUBLIC\DOCUMENTS\CHEMSTATION\1\METHODS\def_LC1.M
Last changed : 5/31/2023 11:04:21 AM by SYSTEM
(modified after loading)
Analysis Method : C:\USERS\PUBLIC\DOCUMENTS\CHEMSTATION\1\METHODS\def_LC1.M
Last changed : 10/16/2023 10:16:44 PM by SYSTEM
(modified after loading)
Sample Info : IC, Hexane/i-PrOH = 80/20, 0.8 mL/min, 30 oC, 230 nm

Additional Info : Peak(s) manually integrated

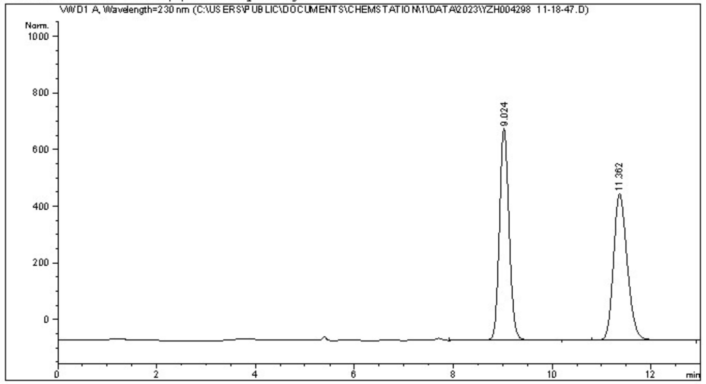

===

Area Percent Report

===

Sorted By : Signal
Multiplier : 1.0000
Dilution : 1.0000
Sample Amount: : 1.00000 [ng/ul] (not used in calc.)
Do not use Multiplier & Dilution Factor with ISTDs

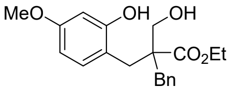

(+/-)-4p

Signal 1: VWD1 A, Wavelength=230 nm

Peak #	RetTime [min]	Type	Width [min]	Area [mAU*s]	Height [mAU]	Area %
1	9.024	VV R	0.2111	8211.14258	602.08978	50.5086
2	11.362	BB	0.2986	8045.78271	416.01419	49.4914

HPLC1260 II 10/16/2023 10:16:49 PM SYSTEM Page 1 of 2

Data File C:\USERS\PUBLIC\DOCUMENTS\CHEMSTATION\1\DATA\2023\YZH004299 11-33-55.D
Sample Name: HW-14-73A TM

===

Acq. Operator : SYSTEM
Sample Operator : SYSTEM
Acq. Instrument : HPLC1260 II Location : 1
Injection Date : 5/31/2023 11:33:56 AM Inj : 1
Inj Volume : No inj
Acq. Method : C:\USERS\PUBLIC\DOCUMENTS\CHEMSTATION\1\METHODS\def_LC1.M
Last changed : 5/31/2023 11:04:21 AM by SYSTEM
(modified after loading)
Analysis Method : C:\USERS\PUBLIC\DOCUMENTS\CHEMSTATION\1\METHODS\def_LC1.M
Last changed : 10/16/2023 10:18:10 PM by SYSTEM
(modified after loading)
Sample Info : IC, Hexane/i-PrOH = 80/20, 0.8 mL/min, 30 oC, 230 nm

Additional Info : Peak(s) manually integrated

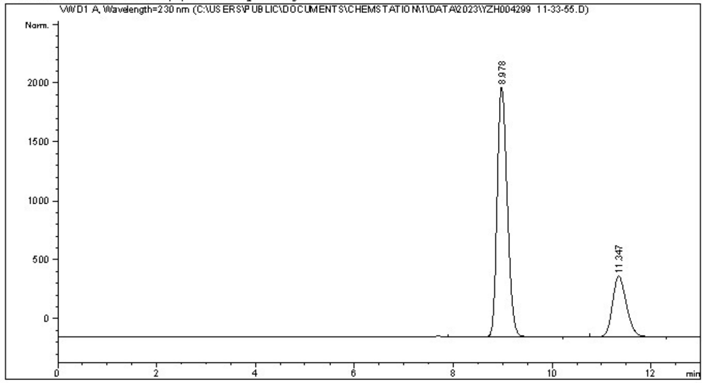

===

Area Percent Report

===

Sorted By : Signal
Multiplier : 1.0000
Dilution : 1.0000
Sample Amount: : 1.00000 [ng/ul] (not used in calc.)
Do not use Multiplier & Dilution Factor with ISTDs

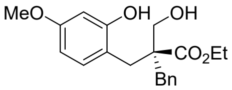

(+)-4p

Signal 1: VWD1 A, Wavelength=230 nm

Peak #	RetTime [min]	Type	Width [min]	Area [mAU*s]	Height [mAU]	Area %
1	8.978	VB R	0.2214	2.56802e4	1806.03650	74.7429
2	11.347	BB	0.3036	8677.82031	440.92514	25.2571

HPLC1260 II 10/16/2023 10:18:14 PM SYSTEM Page 1 of 2

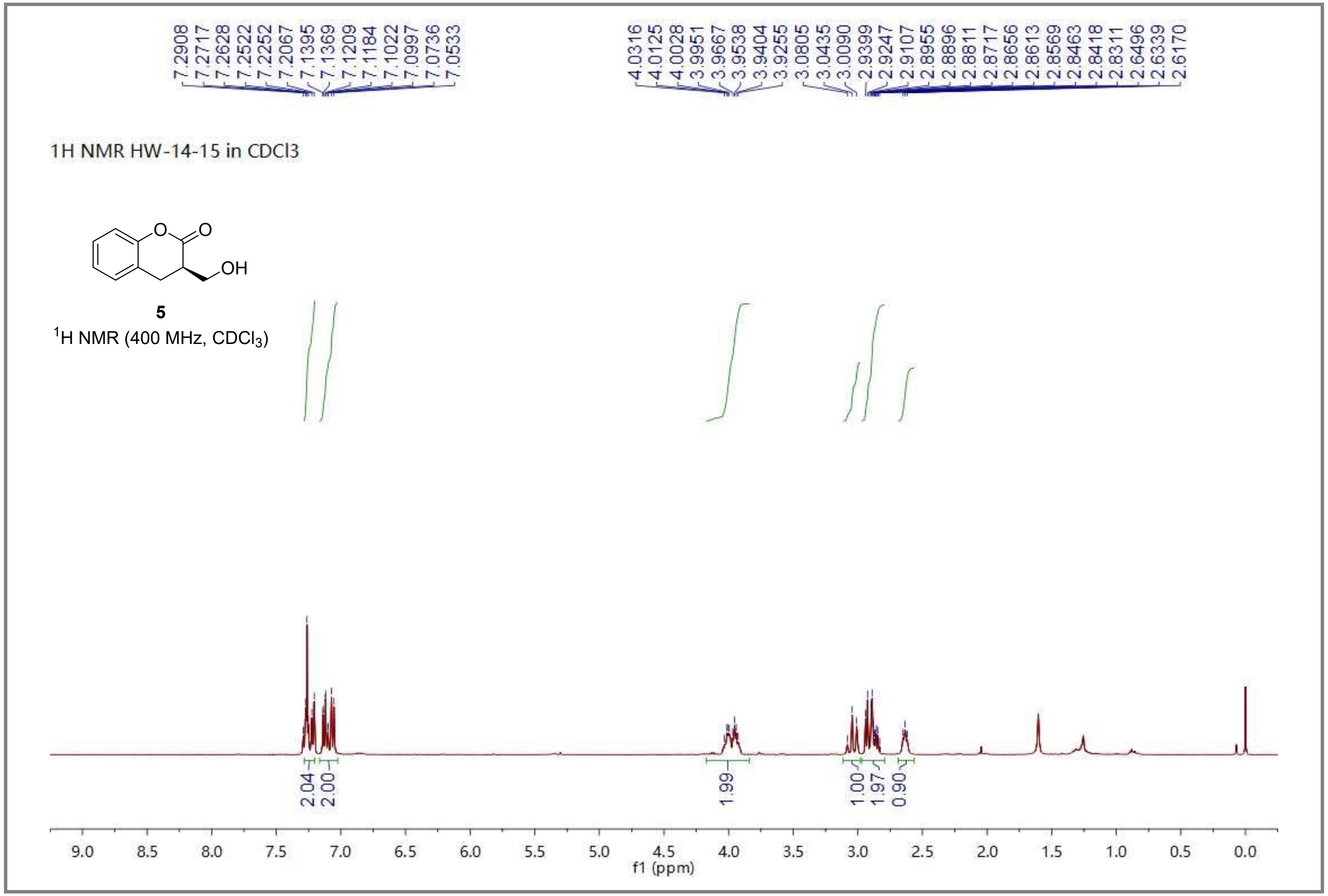

1H NMR HW-14-15 in CDCl3
5
^{1}H NMR (400 MHz, $CDCl_3$)
7.2908
7.2717
7.2628
7.2522
7.2252
7.2067
7.1395
7.1369
7.1209
7.1184
7.1022
7.0997
7.0736
7.0533
4.0316
4.0125
4.0028
3.9951
3.9667
3.9538
3.9404
3.9255
3.0805
3.0435
3.0090
2.9399
2.9247
2.9107
2.8955
2.8896
2.8811
2.8717
2.8656
2.8613
2.8569
2.8463
2.8418
2.8311
2.6496
2.6339
2.6170
2.04
2.00
1.99
1.00
1.97
0.90
f1 (ppm)

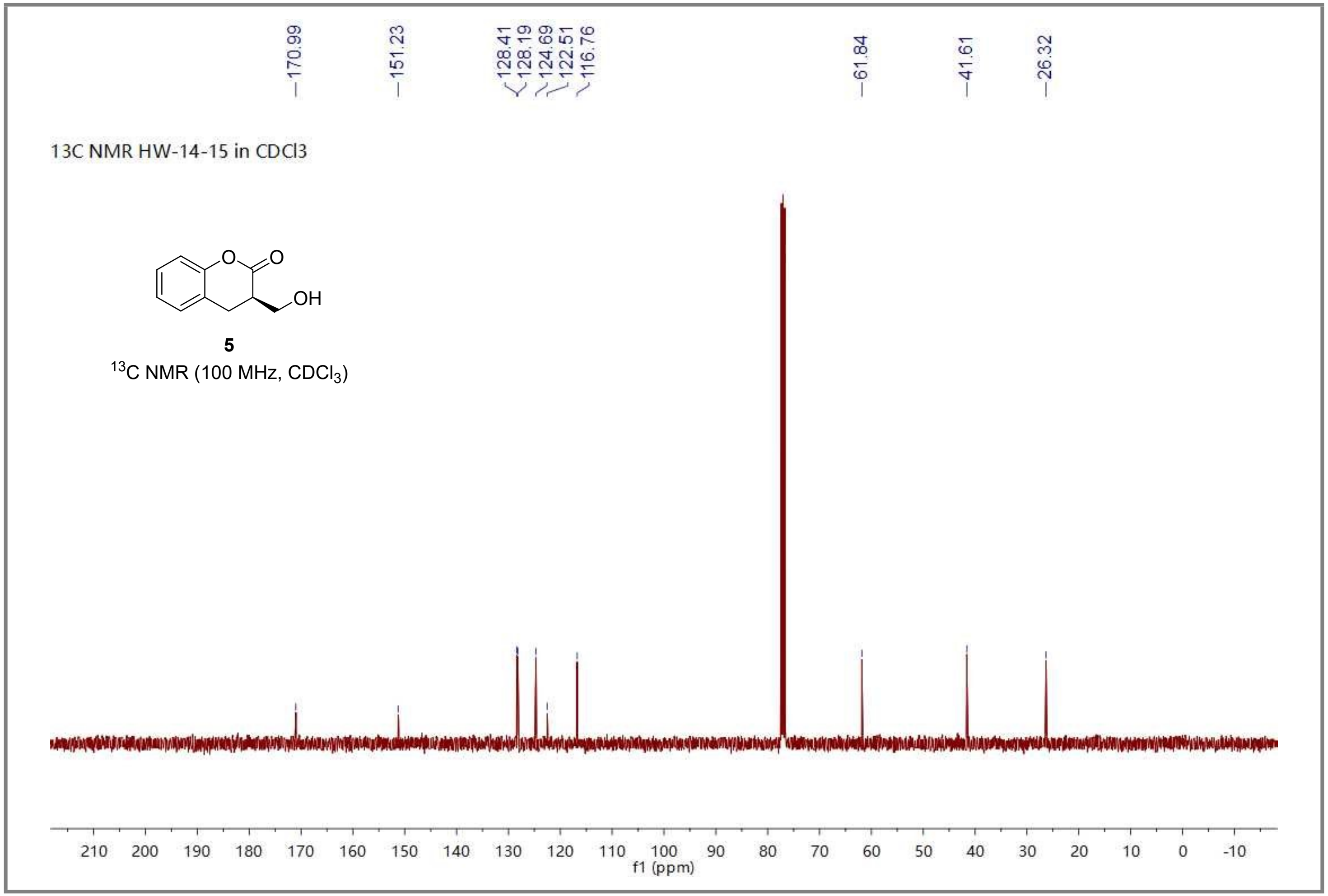

170.99
151.23
128.41
128.19
124.69
122.51
116.76
61.84
41.61
26.32
13C NMR HW-14-15 in CDCl3
O
O
OH
5
13C NMR (100 MHz, CDCl3)
210 200 190 180 170 160 150 140 130 120 110 100 90 80 70 60 50 40 30 20 10 0 -10
f1 (ppm)

Data File C:\CHEM32\1\DATA\ZHOU-23\YZN024770.D
Sample Name: HW-16-24+/-

===

Acq. Operator :
Acq. Instrument : Instrument 1 Location : -
Injection Date : 12/9/2023 2:58:54 AM
Acq. Method : C:\CHEM32\1\METHODS\DEF_LC.M
Last changed : 12/9/2023 2:41:50 AM
(modified after loading)
Analysis Method : C:\CHEM32\1\METHODS\DEF_LC.M
Last changed : 12/23/2023 3:47:04 AM
(modified after loading)
Sample Info : AS-H, Hexane/iPrOH = 90/10, 0.8 mL/min, 30 oC, 230 nm

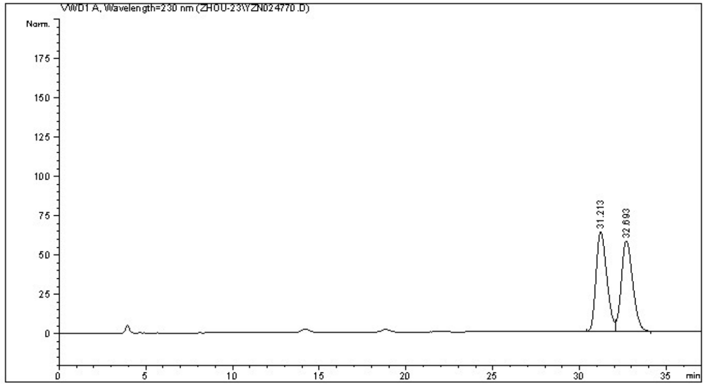

===
Area Percent Report
===

Sorted By : Signal
Multiplier: : 1.0000
Dilution: : 1.0000
Use Multiplier & Dilution Factor with ISTDs

Signal 1: VWD1 A, Wavelength=230 nm

Peak #	RetTime [min]	Type	Width [min]	Area mAU *s	Height [mAU]	Area %
1	31.213	BV	0.6762	2733.57471	63.41490	50.8524
2	32.693	VB	0.7076	2641.93481	57.67999	49.1476

Totals : 5375.50952 121.09489

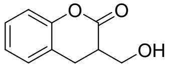

(+/-)-5

===
*** End of Report ***

Instrument 1 12/23/2023 3:47:17 AM Page 1 of 1

Data File C:\CHEM32\1\DATA\ZHOU-23\YZN024771.D
Sample Name: HW-16-24

===

Acq. Operator :
Acq. Instrument : Instrument 1 Location : -
Injection Date : 12/9/2023 3:41:38 AM
Acq. Method : C:\CHEM32\1\METHODS\DEF_LC.M
Last changed : 12/9/2023 3:41:03 AM
(modified after loading)
Analysis Method : C:\CHEM32\1\METHODS\DEF_LC.M
Last changed : 12/23/2023 3:47:04 AM
(modified after loading)
Sample Info : AS-H, Hexane/iPrOH = 90/10, 0.8 mL/min, 30 oC, 230 nm

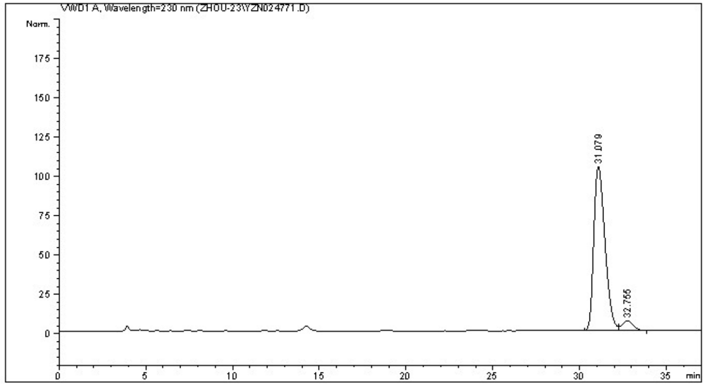

===
Area Percent Report
===

Sorted By : Signal
Multiplier: : 1.0000
Dilution: : 1.0000
Use Multiplier & Dilution Factor with ISTDs

Signal 1: VWD1 A, Wavelength=230 nm

Peak #	RetTime [min]	Type	Width [min]	Area mAU *s	Height [mAU]	Area %
1	31.079	BV	0.6996	4687.87695	104.49860	94.3382
2	32.755	VB	0.6432	281.34866	6.35522	5.6618

Totals : 4969.22562 110.85381

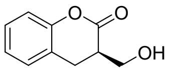

(-)-5

===
*** End of Report ***

Instrument 1 12/23/2023 3:48:13 AM Page 1 of 1

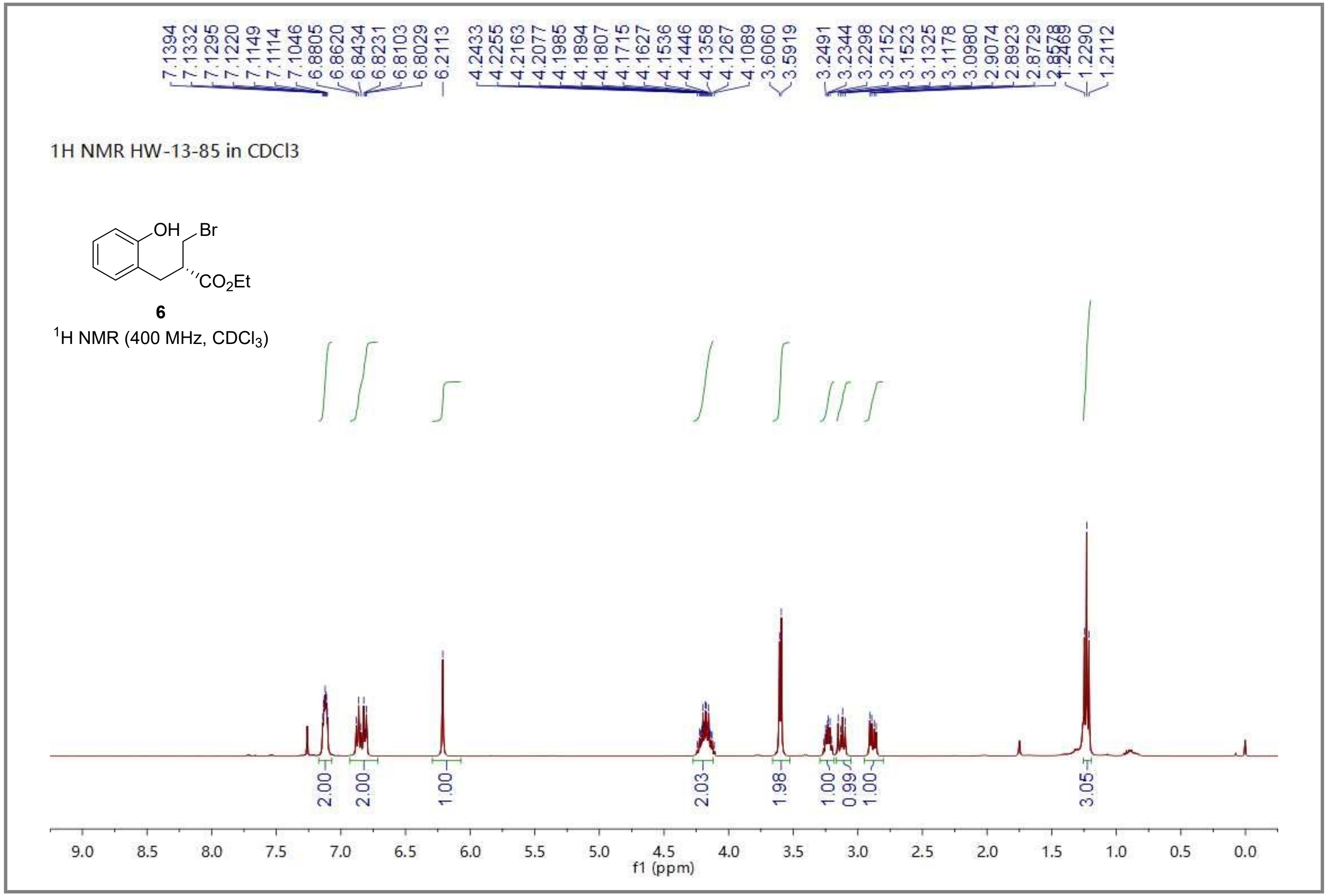

1H NMR HW-13-85 in CDCl3
OH
Br
CO2Et
6
1H NMR (400 MHz, CDCl3)
7.1394 7.1332 7.1295 7.1220 7.1149 7.1114 7.1046 6.8805 6.8620 6.8434 6.8231 6.8103 6.8029 6.2113 4.2433 4.2255 4.2163 4.2077 4.1985 4.1894 4.1807 4.1715 4.1627 4.1536 4.1446 4.1358 4.1267 4.1089 3.6060 3.5919 3.2491 3.2344 3.2298 3.2152 3.1523 3.1325 3.1178 3.0980 2.9074 2.8923 2.8729 2.8578 1.2469 1.2290 1.2112
2.00 2.00 1.00 2.03 1.98 1.00 0.99 1.00 3.05
9.0 8.5 8.0 7.5 7.0 6.5 6.0 5.5 5.0 4.5 4.0 3.5 3.0 2.5 2.0 1.5 1.0 0.5 0.0
f1 (ppm)

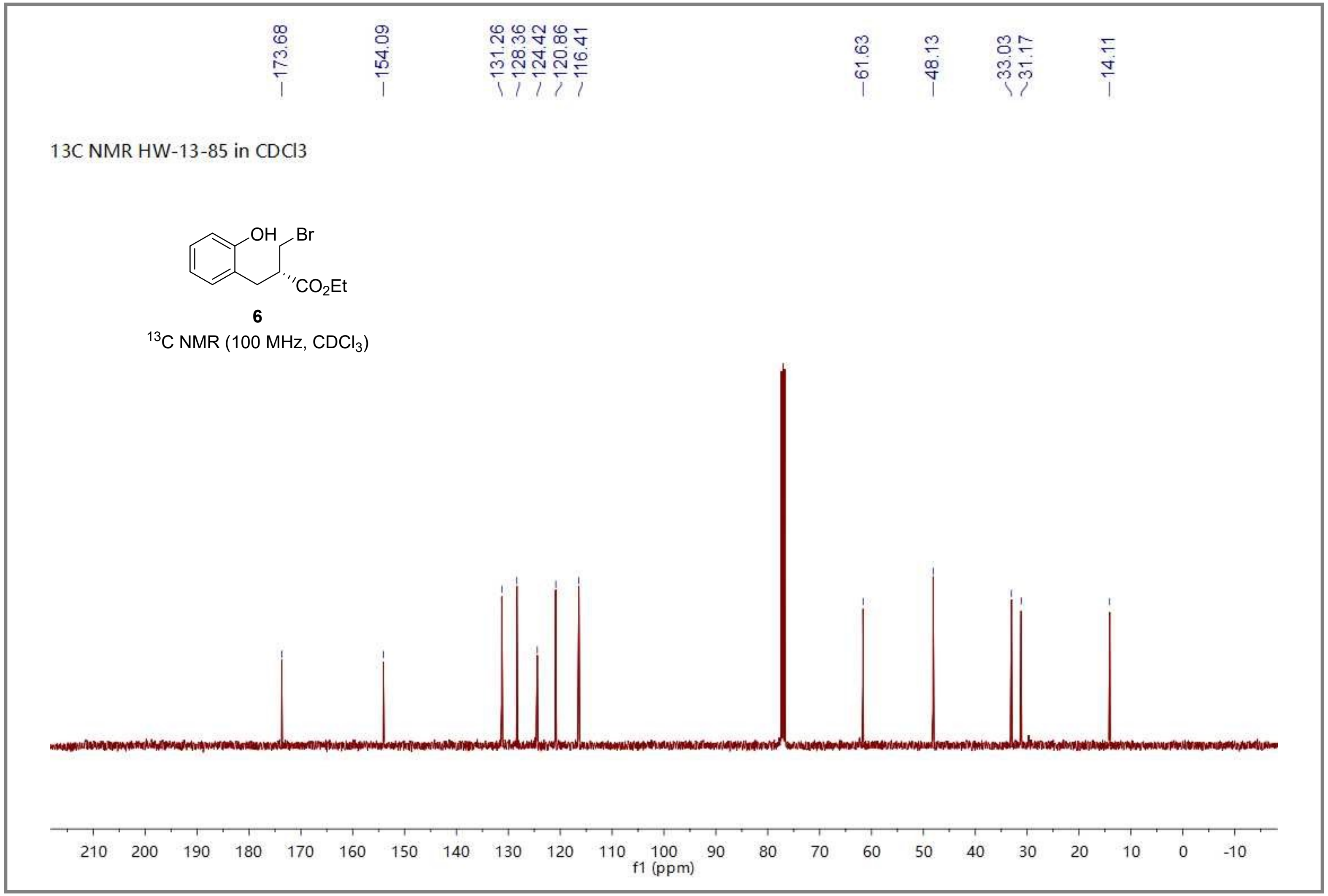
173.68
154.09
131.26
128.36
124.42
120.86
116.41
61.63
48.13
33.03
31.17
14.11
13C NMR HW-13-85 in CDCl3
OH
Br
CO2Et
6
13C NMR (100 MHz, CDCl3)
210 200 190 180 170 160 150 140 130 120 110 100 90 80 70 60 50 40 30 20 10 0 -10
f1 (ppm)

Data File C:\CHEM32\1\DATA\ZHOU-22\YZN022619.D
Sample Name: HW-13-90+/-

===

Acq. Operator :
Acq. Instrument : Instrument 1 Location : -
Injection Date : 3/13/2023 8:44:04 PM
Acq. Method : C:\CHEM32\1\METHODS\DEF_LC.M
Last changed : 3/13/2023 8:28:37 PM
(modified after loading)
Analysis Method : C:\CHEM32\1\METHODS\DEF_LC.M
Last changed : 12/23/2023 3:38:34 AM
(modified after loading)
Sample Info : OD-H, Hexane/i-PrOH = 75/25, 0.8 mL/min, 30 oC, 230nm

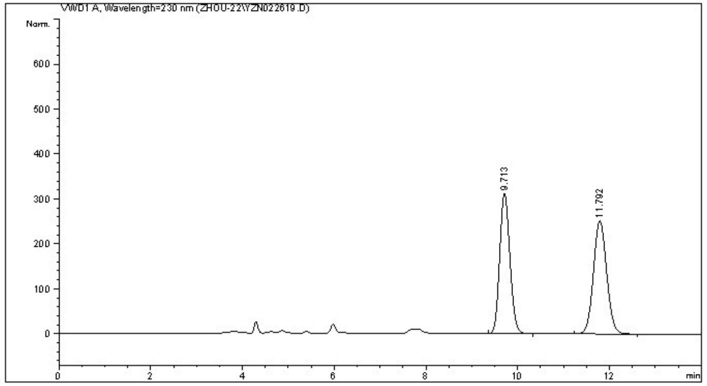

===
Area Percent Report
===

Sorted By : Signal
Multiplier: : 1.0000
Dilution: : 1.0000
Use Multiplier & Dilution Factor with ISTDs

Signal 1: VWD1 A, Wavelength=230 nm

Peak #	RetTime [min]	Type	Width [min]	Area mAU *s	Height [mAU]	Area %
1	9.713	VB	0.2424	4844.71729	311.80060	48.9922
2	11.792	BB	0.3125	5044.02588	251.65918	51.0078

Totals : 9888.74316 563.45978

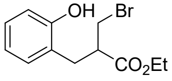

(+/-)-6

===
*** End of Report ***

Instrument 1 12/23/2023 3:38:38 AM Page 1 of 1

Data File C:\CHEM32\1\DATA\ZHOU-22\YZN022621.D
Sample Name: HW-13-96

===

Acq. Operator :
Acq. Instrument : Instrument 1 Location : -
Injection Date : 3/13/2023 9:20:49 PM
Acq. Method : C:\CHEM32\1\METHODS\DEF_LC.M
Last changed : 3/13/2023 9:20:02 PM
(modified after loading)
Analysis Method : C:\CHEM32\1\METHODS\DEF_LC.M
Last changed : 12/23/2023 3:38:34 AM
(modified after loading)
Sample Info : OD-H, Hexane/i-PrOH = 75/25, 0.8 mL/min, 30 oC, 230nm

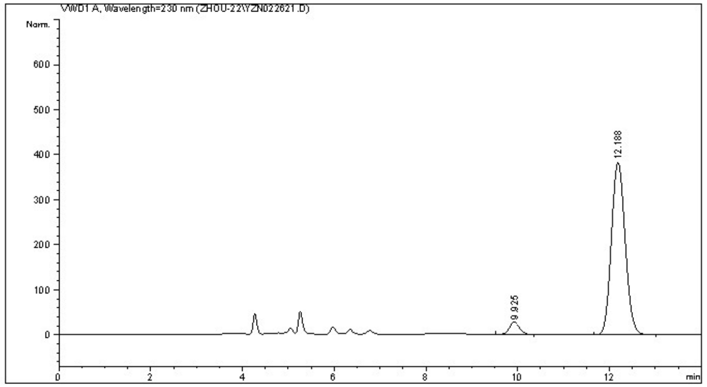

===
Area Percent Report
===

Sorted By : Signal
Multiplier: : 1.0000
Dilution: : 1.0000
Use Multiplier & Dilution Factor with ISTDs

Signal 1: VWD1 A, Wavelength=230 nm

Peak #	RetTime [min]	Type	Width [min]	Area mAU *s	Height [mAU]	Area %
1	9.925	VB	0.2418	426.03473	27.28893	5.0749
2	12.188	BB	0.3261	7968.93896	382.44785	94.9251

Totals : 8394.97369 409.73677

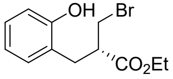

(-)-6

===
*** End of Report ***

Instrument 1 12/23/2023 3:39:19 AM Page 1 of 1

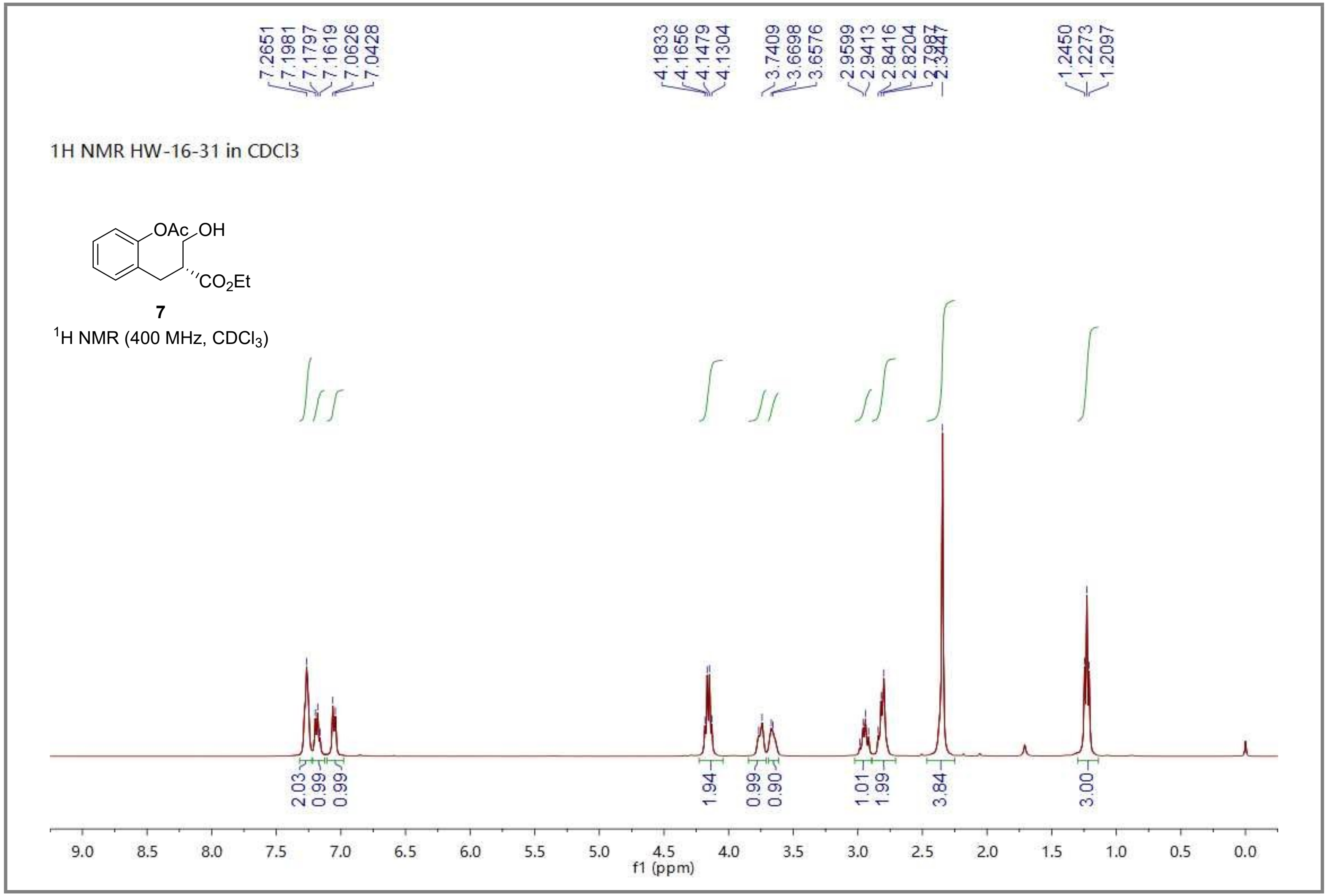

7.2651
7.1981
7.1797
7.1619
7.0626
7.0428
4.1833
4.1656
4.1479
4.1304
3.7409
3.6698
3.6576
2.9599
2.9413
2.8416
2.8204
2.7987
2.3447
1.2450
1.2273
1.2097
1H NMR HW-16-31 in CDCl3
OAc
OH
CO2Et
7
1H NMR (400 MHz, CDCl3)
2.03
0.99
0.99
1.94
0.99
0.90
1.01
1.99
3.84
3.00
9.0
8.5
8.0
7.5
7.0
6.5
6.0
5.5
5.0
4.5
4.0
3.5
3.0
2.5
2.0
1.5
1.0
0.5
0.0
f1 (ppm)

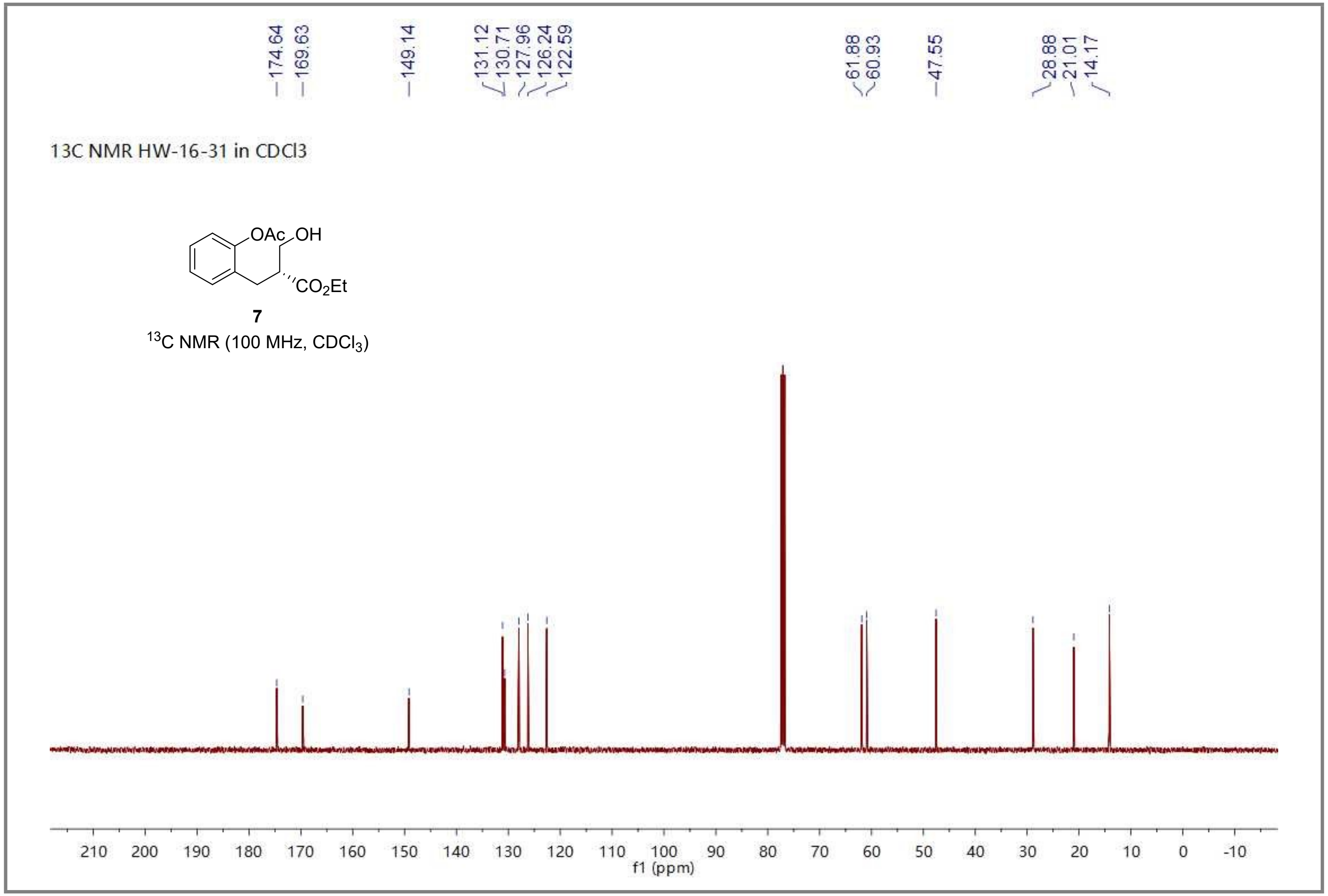
174.64
169.63
149.14
131.12
130.71
127.96
126.24
122.59
61.88
60.93
47.55
28.88
21.01
14.17
13C NMR HW-16-31 in CDCl3
OAc
OH
CO_2Et
7
^{13}C NMR (100 MHz, $CDCl_3$)
210 200 190 180 170 160 150 140 130 120 110 100 90 80 70 60 50 40 30 20 10 0 -10
f1 (ppm)

Data File C:\USERS\PUBLIC\DOCUMENTS\CHEMSTATION\1\DATA\2023\YZH005771 19-04-11.D
Sample Name: HW-16-31+/-

===

Acq. Operator : SYSTEM
Sample Operator : SYSTEM
Acq. Instrument : HPLC1260 II Location : 1
Injection Date : 12/15/2023 7:04:11 PM Inj : 1
Inj Volume : No inj
Acq. Method : C:\USERS\PUBLIC\DOCUMENTS\CHEMSTATION\1\METHODS\def_LC1.M
Last changed : 12/15/2023 7:01:30 PM by SYSTEM
(modified after loading)
Analysis Method : C:\USERS\PUBLIC\DOCUMENTS\CHEMSTATION\1\METHODS\def_LC1.M
Last changed : 12/22/2023 3:55:20 PM by SYSTEM
(modified after loading)
Sample Info : OJ-H, Hexane/i-PrOH = 70/30, 0.8 mL/min, 30 oC, 230 nm

Additional Info : Peak(s) manually integrated

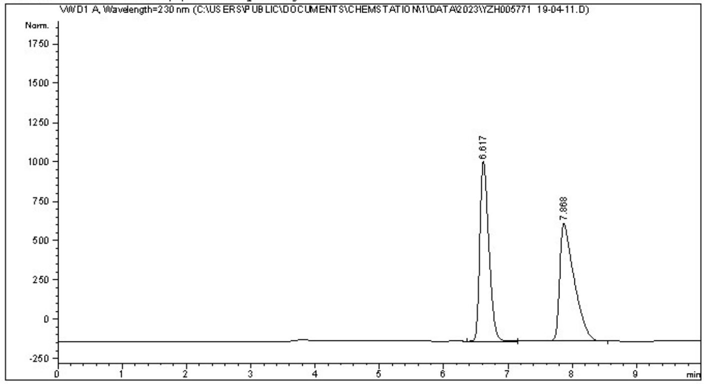

===
Area Percent Report
===

Sorted By : Signal
Multiplier : 1.0000
Dilution : 1.0000
Sample Amount: : 1.00000 [ng/ul] (not used in calc.)
Do not use Multiplier & Dilution Factor with ISTDs

Signal 1: VWD1 A, Wavelength=230 nm

Peak #	RetTime [min]	Type	Width [min]	Area [mAU*s]	Height [mAU]	Area %
1	6.617	BB	0.1490	8325.12598	859.45837	48.6079
2	7.868	VB R	0.2215	8801.97461	565.45422	51.3921

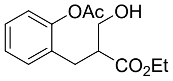

(+/-)-7

Data File C:\USERS\PUBLIC\DOCUMENTS\CHEMSTATION\1\DATA\2023\YZH005774 09-34-58.D
Sample Name: HW-16-31

===

Acq. Operator : SYSTEM
Sample Operator : SYSTEM
Acq. Instrument : HPLC1260 II Location : 1
Injection Date : 12/16/2023 9:34:58 AM Inj : 1
Inj Volume : No inj
Acq. Method : C:\USERS\PUBLIC\DOCUMENTS\CHEMSTATION\1\METHODS\def_LC1.M
Last changed : 12/16/2023 9:18:01 AM by SYSTEM
(modified after loading)
Analysis Method : C:\USERS\PUBLIC\DOCUMENTS\CHEMSTATION\1\METHODS\def_LC1.M
Last changed : 12/22/2023 3:55:20 PM by SYSTEM
(modified after loading)
Sample Info : OJ-H, Hexane/i-PrOH = 70/30, 0.8 mL/min, 30 oC, 230 nm

Additional Info : Peak(s) manually integrated

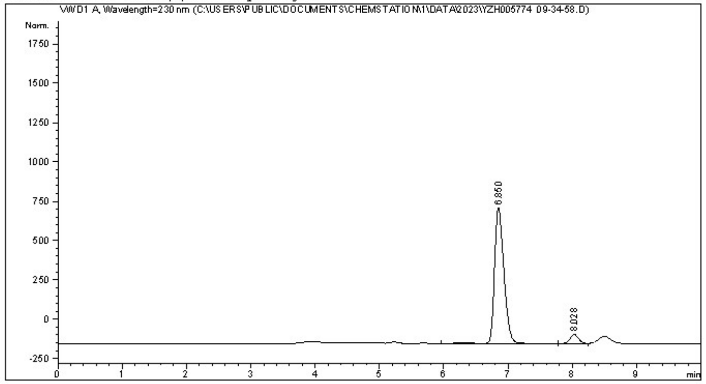

===
Area Percent Report
===

Sorted By : Signal
Multiplier : 1.0000
Dilution : 1.0000
Sample Amount: : 1.00000 [ng/ul] (not used in calc.)
Do not use Multiplier & Dilution Factor with ISTDs

Signal 1: VWD1 A, Wavelength=230 nm

Peak #	RetTime [min]	Type	Width [min]	Area [mAU*s]	Height [mAU]	Area %
1	6.850	VB R	0.1558	5621.48438	553.58759	94.0795
2	8.028	BV	0.1451	353.76694	37.84434	5.9205

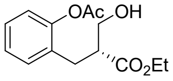

(-)-7

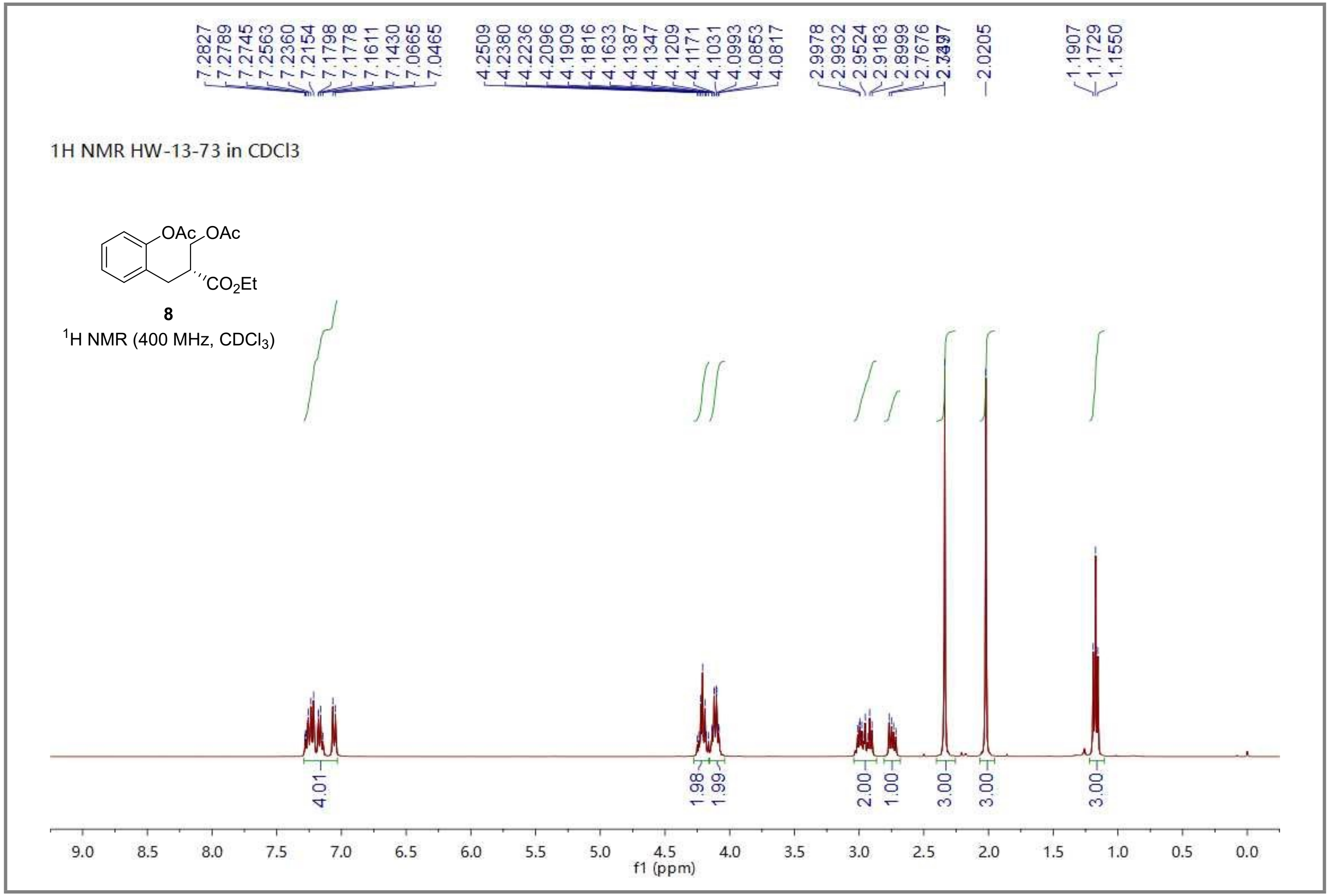
1H NMR HW-13-73 in CDCl3
OAc OAc
CO2Et
8
1H NMR (400 MHz, CDCl3)
7.2827 7.2789 7.2745 7.2563 7.2360 7.2154 7.1798 7.1778 7.1611 7.1430 7.0665 7.0465
4.2509 4.2380 4.2236 4.2096 4.1909 4.1816 4.1633 4.1387 4.1347 4.1209 4.1171 4.1031 4.0993 4.0853 4.0817
2.9978 2.9932 2.9524 2.9183 2.8999 2.7676
2.0205
1.1907 1.1729 1.1550
4.01 1.98 1.99 2.00 1.00 3.00 3.00 3.00
9.0 8.5 8.0 7.5 7.0 6.5 6.0 5.5 5.0 4.5 4.0 3.5 3.0 2.5 2.0 1.5 1.0 0.5 0.0
f1 (ppm)

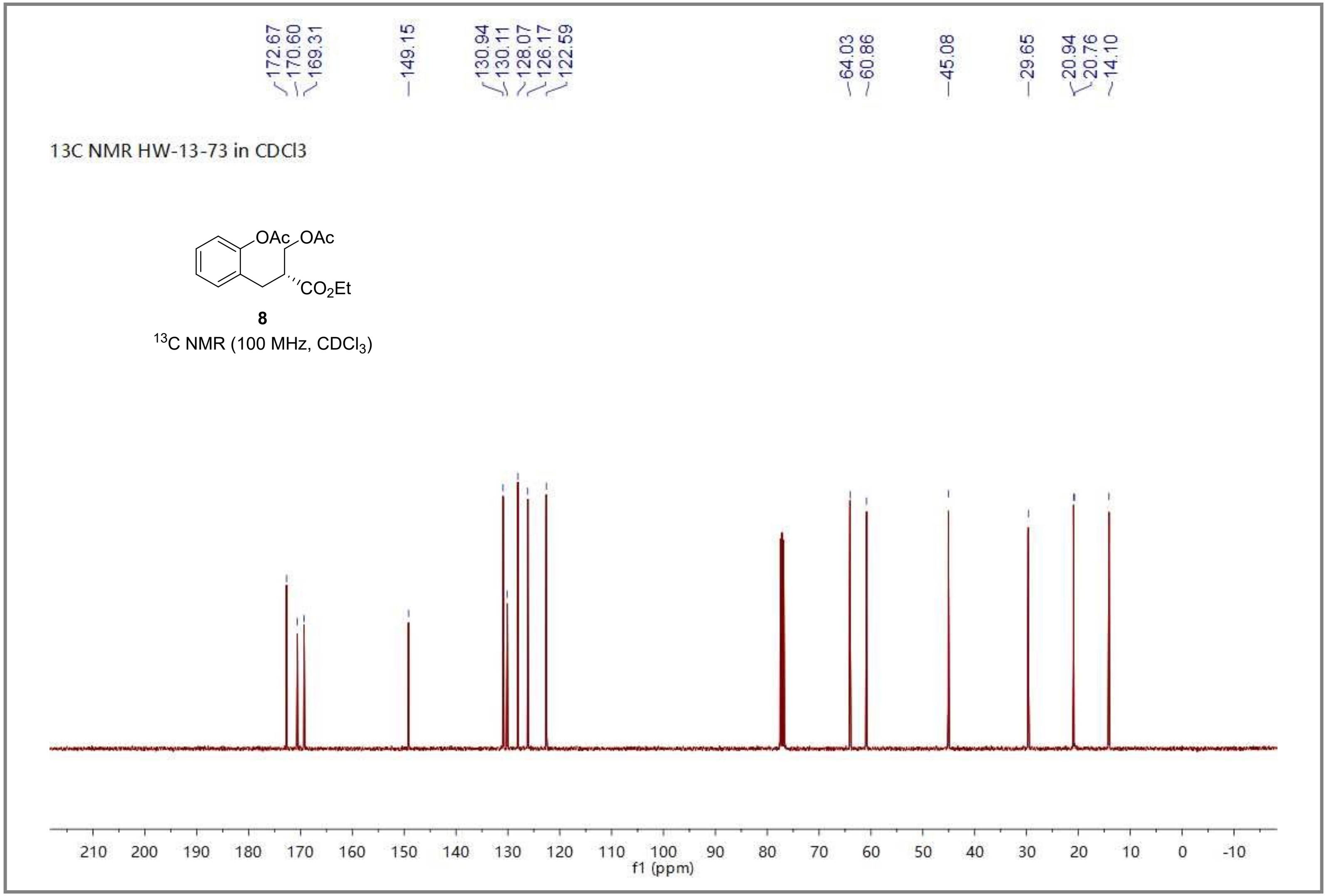
172.67
170.60
169.31
149.15
130.94
130.11
128.07
126.17
122.59
64.03
60.86
45.08
29.65
20.94
20.76
14.10
13C NMR HW-13-73 in CDCl3
OAc
OAc
CO2Et
8
13C NMR (100 MHz, CDCl3)
210 200 190 180 170 160 150 140 130 120 110 100 90 80 70 60 50 40 30 20 10 0 -10
f1 (ppm)

```
Data File C:\CHEM32\1\DATA\ZHOU-22\YZN022476.D
Sample Name: HW-13-73+/-

    =====================================================================
    Acq. Operator   :
    Acq. Instrument : Instrument 1                    Location :   -
    Injection Date  : 2/23/2023 3:08:54 AM
    Acq. Method     : C:\CHEM32\1\METHODS\DEF_LC.M
    Last changed    : 2/23/2023 2:29:51 AM
                      (modified after loading)
    Analysis Method : C:\CHEM32\1\METHODS\DEF_LC.M
    Last changed    : 12/23/2023 3:34:58 AM
                      (modified after loading)
    Sample Info     : OD-H, Hexane/i-PrOH = 95/5, 0.8 mL/min, 30 oC, 254 nm
```

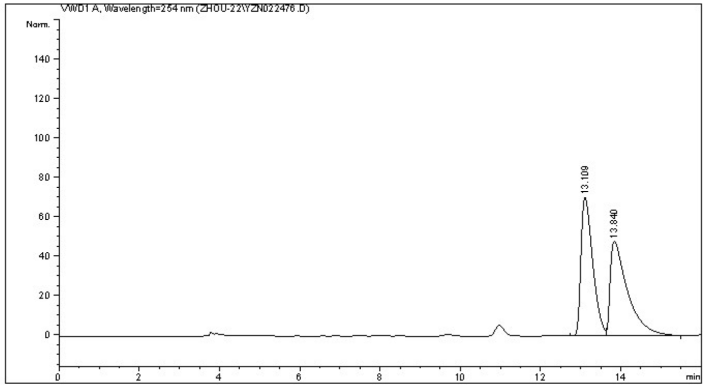


```
    =====================================================================
                               Area Percent Report
    =====================================================================

    Sorted By             :      Signal
    Multiplier:                  :      1.0000
    Dilution:                    :      1.0000
    Use Multiplier & Dilution Factor with ISTDs


    Signal 1: VWD1 A, Wavelength=254 nm

    Peak RetTime Type  Width     Area      Height     Area
      #   [min]        [min]  mAU   *s    [mAU   ]      %
    ----|-------|----|-------|----------|----------|--------|
       1  13.109 VV    0.3114 1430.85986   70.39180  49.5265
       2  13.840 VB    0.4342 1458.21667   48.03914  50.4735

    Totals :                  2889.07654  118.43094

    =====================================================================
                             *** End of Report ***
```

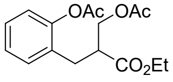

(+/-)-8

Instrument 1 12/23/2023 3:35:17 AM   Page 1 of 1

```
Data File C:\CHEM32\1\DATA\ZHOU-22\YZN022477.D
Sample Name: HW-13-73

    =====================================================================
    Acq. Operator   :
    Acq. Instrument : Instrument 1                    Location :   -
    Injection Date  : 2/23/2023 3:27:49 AM
    Acq. Method     : C:\CHEM32\1\METHODS\DEF_LC.M
    Last changed    : 2/23/2023 3:26:32 AM
                      (modified after loading)
    Analysis Method : C:\CHEM32\1\METHODS\DEF_LC.M
    Last changed    : 12/23/2023 3:37:20 AM
                      (modified after loading)
    Sample Info     : OD-H, Hexane/i-PrOH = 95/5, 0.8 mL/min, 30 oC, 254 nm
```

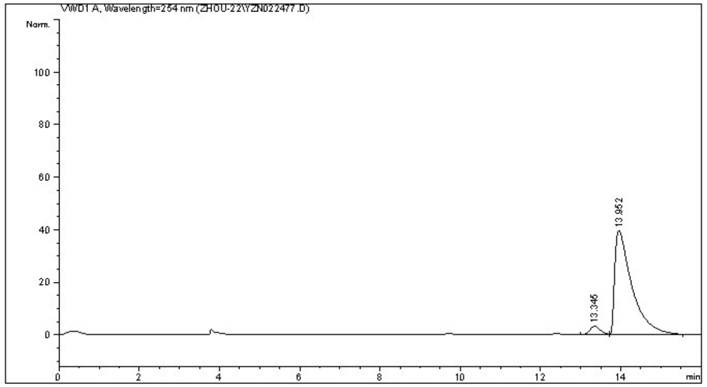


```
    =====================================================================
                               Area Percent Report
    =====================================================================

    Sorted By             :      Signal
    Multiplier:                  :      1.0000
    Dilution:                    :      1.0000
    Use Multiplier & Dilution Factor with ISTDs


    Signal 1: VWD1 A, Wavelength=254 nm

    Peak RetTime Type  Width     Area      Height     Area
      #   [min]        [min]  mAU   *s    [mAU   ]      %
    ----|-------|----|-------|----------|----------|--------|
       1  13.345 BV    0.2852   59.21564    3.20821   4.7798
       2  13.952 VB    0.4298 1179.64685   39.68708  95.2202

    Totals :                  1238.86249   42.89529

    =====================================================================
                             *** End of Report ***
```

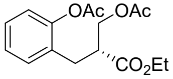

(-)-8

Instrument 1 12/23/2023 3:37:23 AM   Page 1 of 1
